# Atroposelective construction of indole-fused diazocines *via* gold(i)-catalysed 8-*endo*-dig cyclisation

**DOI:** 10.1039/d6sc00020g

**Published:** 2026-04-28

**Authors:** Silvia Meraviglia, Alessandra Romanelli, Paola Iannelli, Silvia Rizzato, Alessandro Contini, Giorgio Abbiati, Valentina Pirovano

**Affiliations:** a Dipartimento di Scienze Farmaceutiche, Sezione di Chimica Geneale e Organica “A. Marchesini” Università degli Studi di Milano Via C. Golgi 19 Milano 20133 Italy valentina.pirovano@unimi.it; b Dipartimento di Chimica Università degli Studi di Milano Via C. Golgi 19 Milano 20133 Italy

## Abstract

Axially chiral *N*-bridged biaryls embedded in medium-sized rings remain largely unexplored because of the combined challenges associated with C–N axial chirality and medium-ring formation. Here we report a gold(i)-catalysed atroposelective intramolecular cyclisation of indole-derived aryl propiolamides that enables direct access to indole-fused diazocines combining a C–N stereogenic axis with a conformationally constrained diazocine core. The transformation proceeds through a stereocontrolled 8-*endo*-dig hydroarylation promoted by a cationic gold complex bearing a BINOL-derived phosphoramidite ligand, affording the target scaffolds in high yields and excellent enantioselectivities across a broad substrate scope. DFT and QTAIM analyses reveal that stereocontrol originates from differential non-covalent interactions in key cyclisation intermediates. The resulting diazocines exhibit high barriers to racemisation and can be further diversified through downstream functionalisation without erosion of enantiopurity. Preliminary spectroscopic and DNA-interaction studies indicate that these rigid atropisomeric frameworks may be relevant for applications in molecular recognition. Overall, this work establishes a general catalytic strategy for the construction of medium-sized *N*-bridged atropisomers and expands the scope of gold-catalysed asymmetric cyclisations.

## Abstract 2

Gold(i)-catalysed atroposelective cyclisation affords indole-fused diazocines featuring a conformationally constrained diazocine core, combining high enantioselectivity, configurational stability, and potential for molecular recognition.

## Graphical abstract

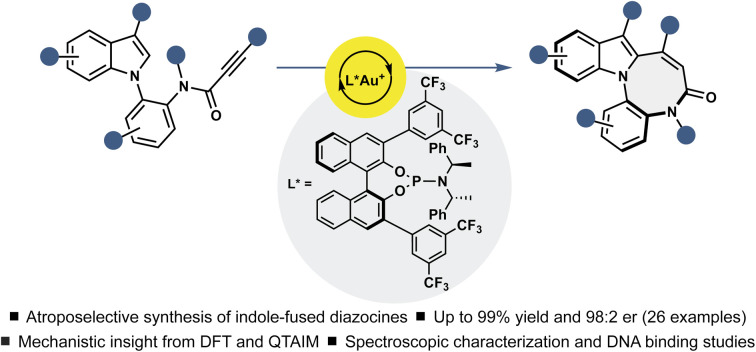

## Introduction

**Scheme 1:**
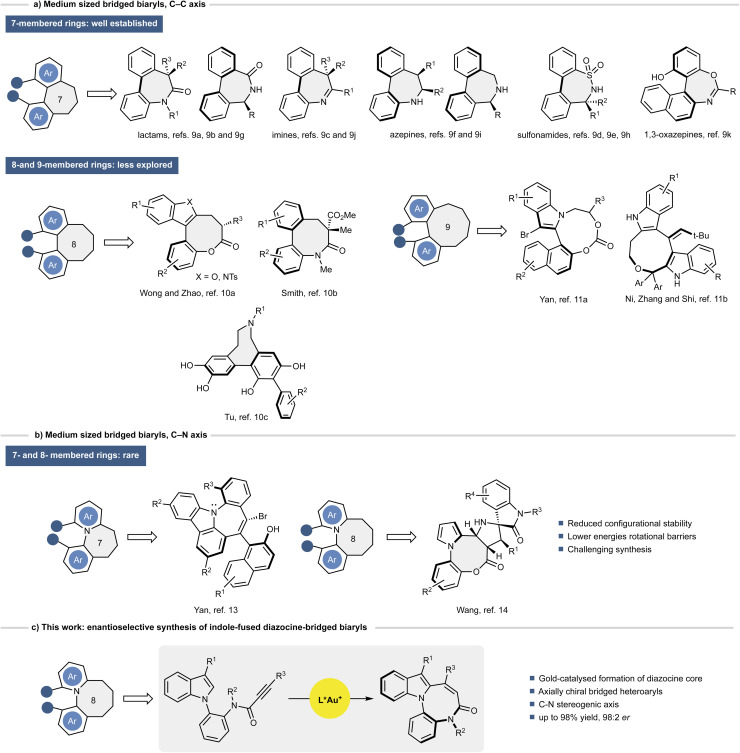
State of the art on the enantioselective synthesis of bridged biaryls (a) bearing a C–C stereogenic axis, (b) bearing a C–N stereogenic axis, and (c) our work.

Axially chiral (hetero)biaryls represent a central class of atropisomeric compounds with broad applications in asymmetric catalysis, medicinal chemistry, and functional materials.^1–3^ Within this class, bridged biaryls, where two ortho-substituted aryl units are covalently linked through a medium-sized ring, have attracted increasing attention owing to their enhanced configurational stability and well-defined three-dimensional architectures.^4^ In these systems, the cyclic tether restricts conformational mobility and can stabilize atropisomerism either by hindering rotation about the biaryl bond or by preventing ring-flip inversion processes, as highlighted in studies of medium-sized heterocyclic frameworks.^5^ Despite their attractive structural features and potential utility, the enantioselective synthesis of such scaffolds remains highly challenging, especially for medium-sized ring systems, which suffer from transannular strain, torsional tension, and unfavourable entropic factors.^6^ Considerable progress has been made in recent years in the atroposelective synthesis of biaryl systems using organocatalytic and transition-metal-catalysed approaches,^7^ including a limited number of bridged medium-ring architectures displaying conformationally or axially defined chirality.^8^ Nevertheless, most reported strategies focus on seven-membered rings,^9^ while catalytic enantioselective methods for the construction of larger bridged biaryls remain scarce. To date, only few approaches enabling the direct asymmetric synthesis of eight-membered bridged biaryls have been disclosed,^10^ and only two examples of nine-membered analogues have been reported (Scheme 1a).^11^ An even more demanding target is represented by axially chiral biaryls bearing a C–N stereogenic axis within a medium-sized ring. In contrast to C–C atropisomers, C–N axial chirality is associated with lower rotational barriers, greater conformational flexibility, and a strong dependence of configurational stability on both electronic effects and ring size.^12^ As a consequence, enantioselective methods to access such architectures are exceedingly rare. To date, only two asymmetric examples have been reported: an organocatalytic synthesis of azepine derivatives bearing a C–N chiral axis by Yan and co-workers,^13^ and a chiral phosphoric acid-catalysed [3 + 2] cycloaddition leading to eight-membered *N*-arylpyrrole lactones reported by Wang (Scheme 1b).^14^

Indole-fused diazocine-bridged biaryls combine a C–N stereogenic axis with a conformationally constrained diazocine core, offering rigid architectures of potential interest for chiral ligand design, medicinal chemistry, and functional materials. However, their synthesis remains highly demanding due to the need to simultaneously induce axial chirality and form a medium-sized ring. To date, this class of compounds has only been accessed by Liao and co-workers *via* a multi-step synthetic sequence.^15^

**Fig. 1:**
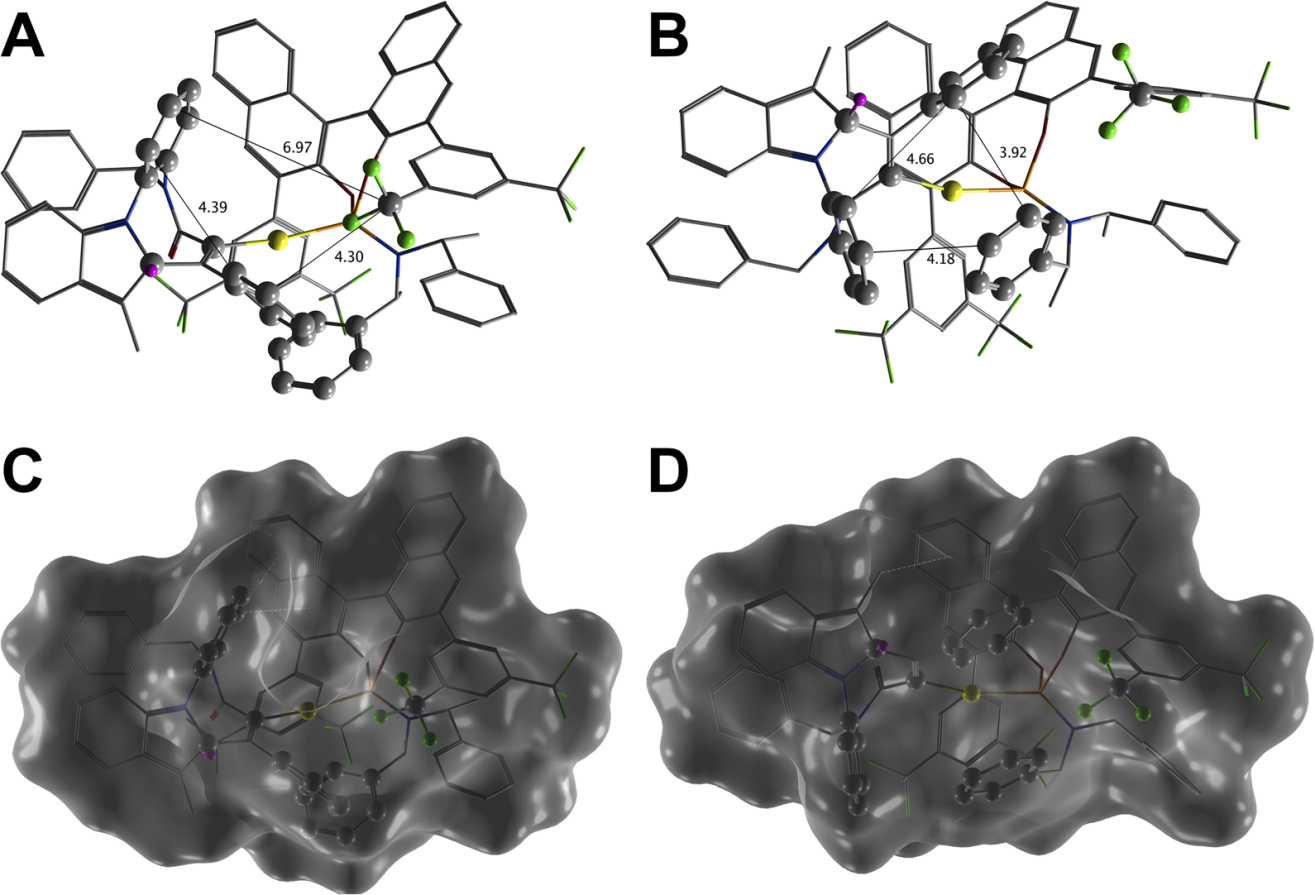
Structural comparison of the diastereomeric intermediates (*S*)-II and (*R*)-II illustrating the steric gating of the deprotonation step. (A and B) Three-dimensional geometries of (*S*)-II and (*R*)-II, respectively, with key interatomic distances (Å) defining the entrance to the C^2^-position. The transferring C^2^-proton is highlighted in magenta. (C and D) Corresponding molecular surfaces representations. The (*S*)-II isomer (C) exhibits an open chiral cage capable of accommodating an external base or proton shuttle, whereas the C^2^-proton in the (*R*)-II isomer (D) is heavily sterically shielded by the surrounding aromatic framework.

Inspired by the well-established ability of gold catalysts to activate alkynes and promote intramolecular cyclisations toward fused heterocycles,^16^ and building upon our group's ongoing interest in complex indole scaffolds,^17^ we envisioned that a gold(i)-catalysed strategy could overcome the challenges associated with diazocine ring formation. Herein, we report a gold(i)-catalysed atroposelective synthesis of indole-fused diazocines, achieved through a stereocontrolled 8-*endo*-dig intramolecular hydroarylation (Fig. 1c). This strategy provides efficient access to configurationally stable, medium-sized *N*-bridged atropisomers with high levels of enantioselectivity, and is supported by combined computational and spectroscopic studies that elucidate the origin of stereocontrol and highlight the functional potential of these rigid atropisomeric frameworks as molecular recognition scaffolds.

## Results and discussion

### Optimization of reaction conditions

We initiated our investigation by designing a suitable indole derivative for the enantioselective construction of the diazocine core. A literature survey on gold-catalysed carbocyclisations revealed that indoloazocines can be accessed from tryptamine-derived propiolamides, typically prepared *via* a Ugi multicomponent reaction.^18^ Inspired by these precedents, we synthesized model compound 1aa, featuring a propiolamide functional group on the *N*-aryl substituent. Compound 1aa was obtained in four steps from 3-methylindole and isolated as a mixture of rotamers, as evidenced by NMR analyses (see SI).

First, the reaction conditions were optimised by screening a series of chiral gold catalysts in dichloromethane at room temperature, using AgSbF_6_ for counterion exchange (Table 1, see SI for full screening table). The structure and electronic properties of the gold complexes had a strong influence on the outcome, both in terms of yield and enantiomeric ratio. Bidentate ligands such as (*R*)-DTBM-SegPhos (L_1_), (*R*)-DM-SegPhos (L_2_), and (*R*)-DM-BINAP (L_3_) failed or were inefficient in promoting the reaction (entries 1–3). The desired indoloazocine 2aa was obtained with L_2_ and L_3_, but only after 48 h and in very low enantiomeric ratios. Improved results were obtained using monodentate phosphoramidite ligands. SPINOL-based ligand L_4_ afforded 2aa in 82% yield, albeit as a racemate (entry 4), while BINOL-derived ligands L_5_ and L_6_ provided 75% and 71% yield, respectively, with modest enantiocontrol (entries 5 and 6). The best results were achieved with ligand L_7_, bearing a 3,5-bis(trifluoromethyl)phenyl substituent on the BINOL scaffold. After 24 h, 2aa was isolated in quantitative yield with an improved enantiomeric ratio of 8 : 92 (entry 7). Next, we examined the effect of the counterion by replacing AgSbF_6_ with AgNTf_2_, which did not significantly affect the reaction outcome (entry 8). A more pronounced effect was observed upon changing the solvent. When toluene was used, the enantiomeric ratio improved to 2 : 98; however, the reaction slowed considerably, and 2aa was obtained in only 38% yield after 48 h (entry 9). In contrast, chlorobenzene maintained both a high yield (94% after 24 h) and excellent enantioselectivity (entry 10). Based on these results, the conditions of entry 10, L_7_AuCl/AgSbF_6_ in chlorobenzene, were selected as optimal and employed in all subsequent studies. The structure and the absolute *S*-configuration of 2aa were confirmed through X-ray single-crystal diffraction analysis and those of the other products were assigned by analogy.^‡^

**Table 1:** Optimisation of reaction conditions^a^

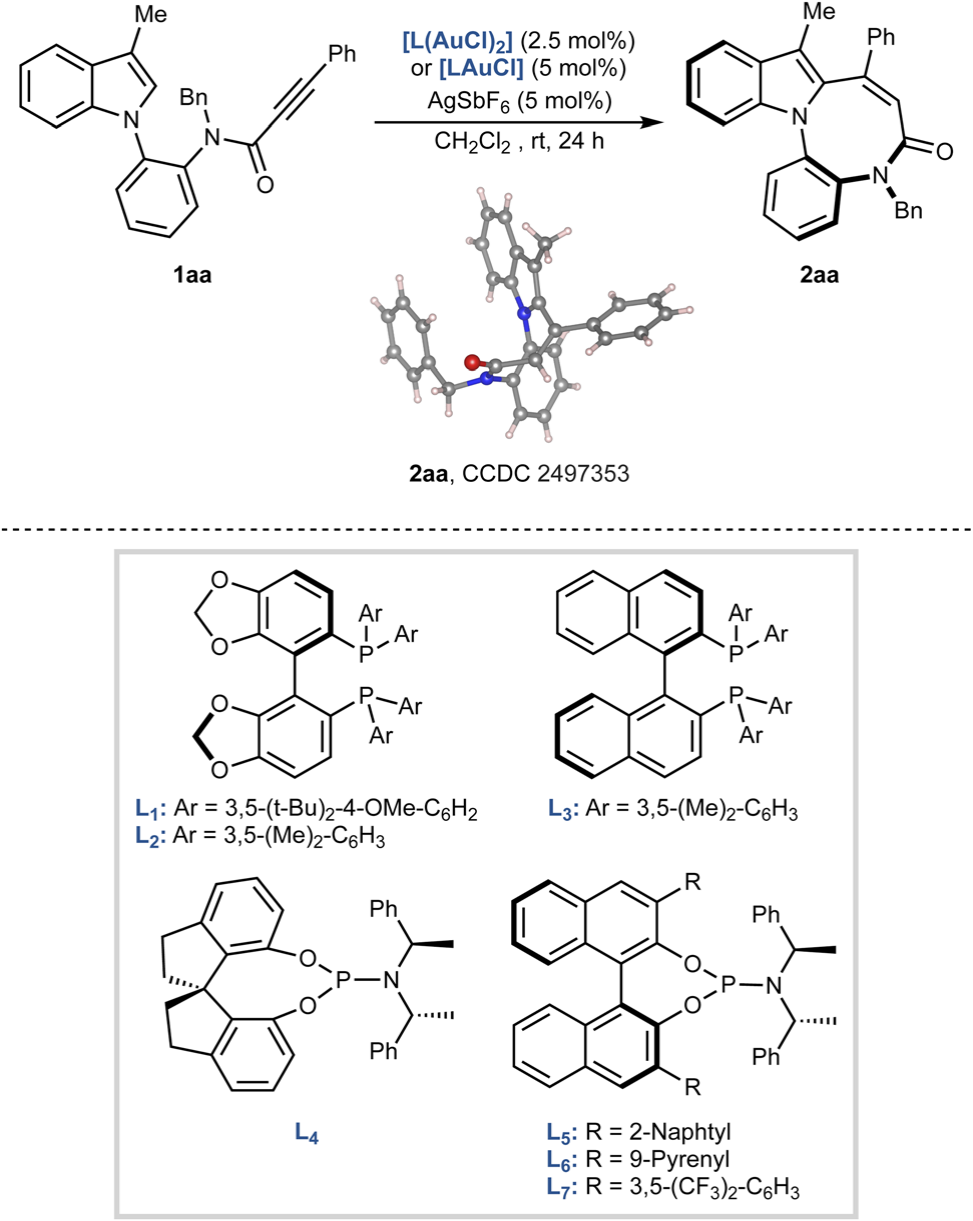				
Entry^a^	Au	Deviations	2aa, yield (%)^b^	2aa, er^c^
1	L_1_(Au_2_Cl_2_)	—	—	—
2	L_2_(Au_2_Cl_2_)	48 h	17	70 : 30
3	L_3_(Au_2_Cl_2_)	48 h	20	55 : 45
4	L_4_AuCl	—	82	52 : 48
5	L_5_AuCl	—	75	30 : 70
6	L_6_AuCl	48 h	71	25 : 75
7	L_7_AuCl	—	99	8 : 92
8	L_7_AuCl	AgNTf_2_	78	7 : 93
9	L_7_AuCl	Toluene, 48 h	38	2 : 98
10	L_7_AuCl	Chlorobenzene	94	2 : 98

a Unless otherwise stated, reactions were carried out with 1aa (0.1 mmol), gold catalyst (2.5 or 5 mol%), AgSbF_6_ (5 mol%) in anhydrous CH_2_Cl_2_ (1 mL, 0.1 M) at room temperature for 24 h.

b Isolated yields.

c Enantiomeric ratios (er) determined by chiral HPLC. See SI for full experimental details.

### Scope of the reaction

**Scheme 2:**
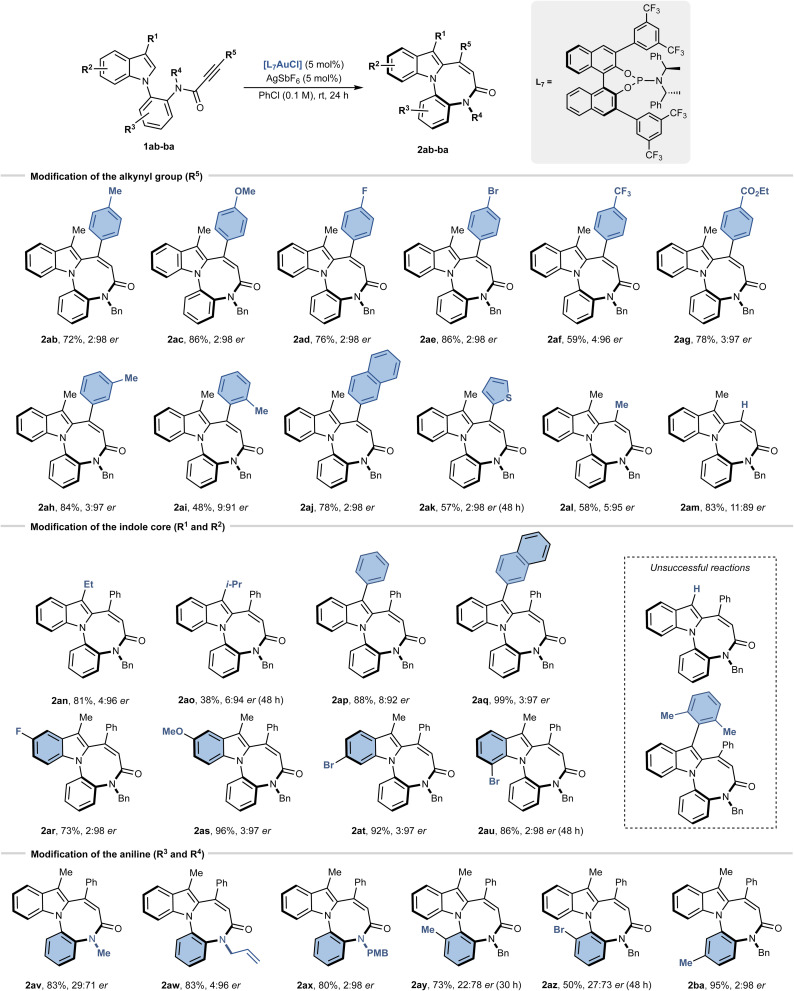
Scope of the reaction. Reaction conditions: 1aa–ba (0.1 mmol), [L_7_AuCl] (5 mol%), AgSbF_6_ (5 mol%) in chlorobenzene (1 mL) at rt 24–48 h. The yield was determined after chromatographic purification. Enantiomeric ratios (er) were determined by chiral HPLC. PMB = *p*-methoxybenzyl.

To assess the scope and generality of the transformation, a broad set of indole derivatives (1ab–1ba) was evaluated under the optimised reaction conditions (Scheme 2). Modification of the alkynyl substituent (R^5^) revealed broad tolerance to electronic effects: both electron-donating (2ab, Me; 2ac, OMe) and electron-withdrawing groups (2ad, F; 2ae, Br; 2af, CF_3_; 2ag, CO_2_Et) in the *para* position of aryl ring afforded the desired products in 59–86% yield and up to 2 : 98 er. A *meta*-methylphenyl substituent was equally effective (2ah, 84%, 3 : 97 er), whereas the *ortho*-substitution produced a drop in both yield and selectivity (2ai, 48%, 9 : 91 er).

Beyond phenyl, naphthyl (2aj) and thiophenyl (2ak) substituents were also well tolerated (78–57% yield, up to 2 : 98 er). Importantly, even less activated methyl- and terminal-substituted alkynes could be engaged, delivering 2al and 2am in synthetically meaningful yields (58% and 83%, respectively) while maintaining high levels of enantioselectivity (up to 5 : 95 er). At the pyrrole moiety, the 3-methyl substituent (R^1^) could be replaced with other alkyl and aryl groups. Ethyl substitution (2an) was well tolerated (81%, 4 : 96 er), whereas sterically hindered isopropyl (2ao) significantly slowed the reaction and reduced the yield (38%, 6 : 94 er). Remarkably, aromatic substituents at C^3^ (2ap, 2aq) provided excellent results (88–99%, up to 3 : 97 er). In contrast, unsubstituted indole (R^1^ = H) was completely unreactive, and very bulky arenes (R^1^ = 2,6-dimethylphenyl) gave only traces of product (Scheme 2). The complete lack of reactivity for the unsubstituted indole (R^1^ = H) can be rationalized by the dual role of a substituent at C^3^. On one hand, the presence of a substituent on the C^3^-position leads to the formation of a stable tertiary carbocation. Second, the presence of alkyl/aryl groups increases the nucleophilicity of the C^2^-position thus favouring the cyclisation.^19^ Substitution on the phenyl moiety of the indole ring (R^2^) displayed a broad compatibility: both electron-donating (2as) and electron-withdrawing (2ar, 2at, 2au) substituents at C^5^–C^7^ delivered the desired products in 73–96% yield and excellent er values. The second group on amide nitrogen (R^4^) was critical for enantioselectivity. Small aliphatic group such as methyl (2av) hampered the enantioselectivity (29 : 71 er), whereas unsaturated groups (allyl, 2aw; *p*-methoxybenzyl, PMB, 2ax) preserved it. Finally, substitution on the aniline ring (R^3^) was strongly position-dependent: *ortho*-substituents (2ay and 2az) slowed the reaction and eroded both yield and selectivity, whereas the *meta*-substituted analogue (2ba) allowed to retain the usual efficiency (95%, 2 : 98 er).

**Scheme 3:**
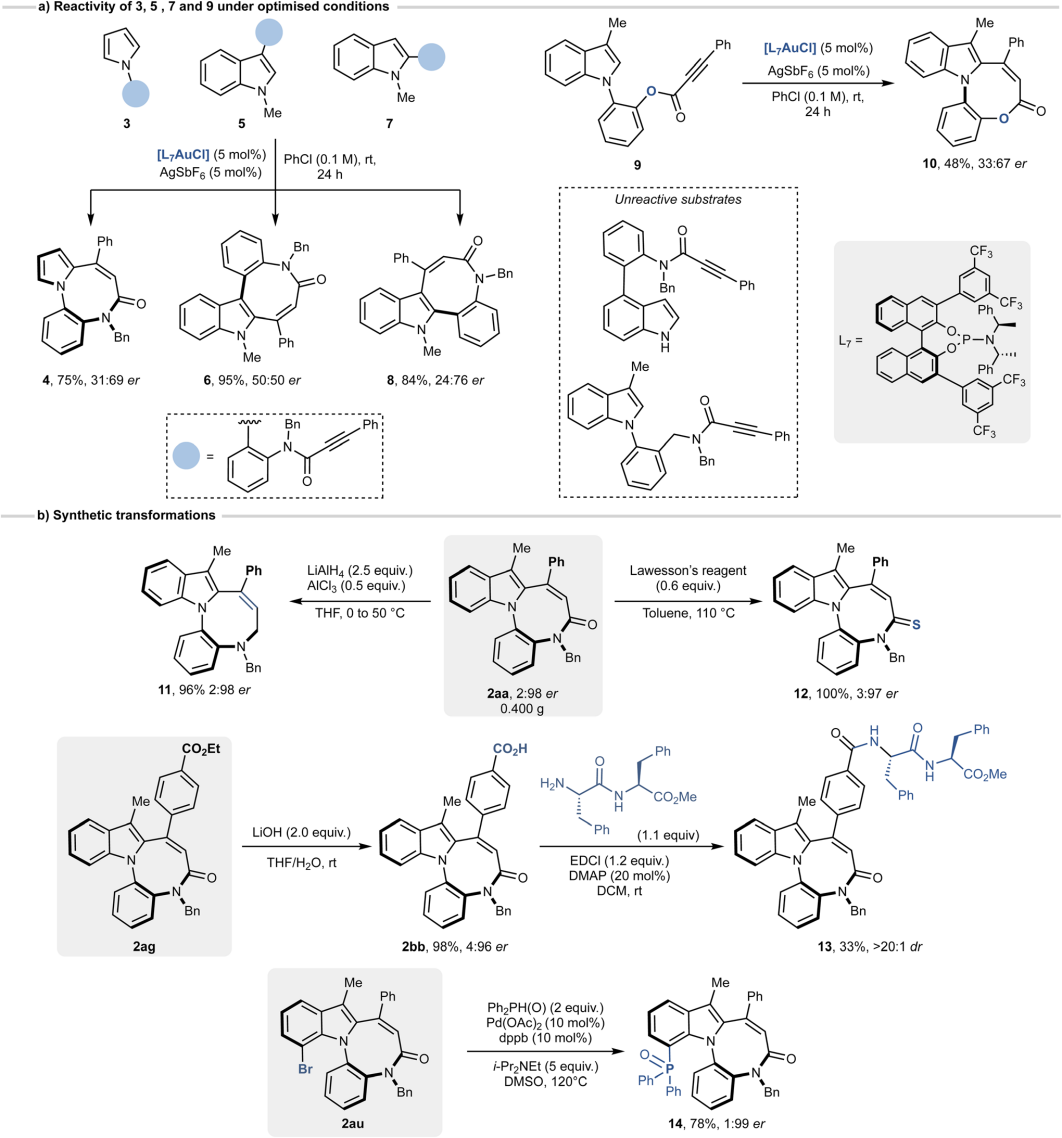
(a) Expansion of the scope to 3, 5, 7 and 9. (b) Post-functionalisations of selected substrates.

To expand the scope of the reaction, we evaluated whether the gold-catalysed cyclisation could be extended to substrates beyond 1ab–ba. We synthesised *N*-arylpyrrole 3 and indoles 5 and 7, each featuring an aryl propiolamide group at the C^3^- and C^2^-position, respectively (Scheme 3a). When subjected to the optimised reaction conditions, these substrates consistently afforded the corresponding diazocino-bridged biaryls (compounds 4–8) in high yields (75–95%). However, achieving enantiocontrol proved challenging. Pyrrole 4 was obtained with a poor enantiomeric ratio of 31 : 69, while indole 6, bearing a C^3^–C chiral axis, was formed as a racemate. Slightly better results were observed for compound 8 (C^2^–C chiral axis), which was obtained with an enantiomeric ratio of 24 : 76. To further probe the structural limitations of the transformation, we examined substrates targeting alternative ring sizes and tether functionalities. An ester analogue 9 underwent cyclisation to give the eight-membered product 10 in 48% yield, albeit with reduced enantioselectivity (33 : 67 er). In contrast, modified amide substrates intended to form nine-membered rings were completely unreactive under the optimised conditions. These findings emphasize the critical role of tether length and amide coordination in enabling efficient and stereocontrolled eight-membered ring formation.

Having synthesised a series of indole-fused diazocines, we next performed a set of post-functionalisations on selected substrates (Scheme 3b). The scalability of the reaction was confirmed on a 1.0 mmol scale, affording 2aa in 91% yield with unchanged enantiomeric ratio. Chemoselective derivatization further highlighted the synthetic utility of the diazocine scaffold. Reduction of the amide group of 2aa with LiAlH_4_ and AlCl_3_ afforded compound 11 in 96% yield, while the treatment with Lawesson's reagent gave the corresponding thioamide 12 in quantitative yield. Hydrolysis of the ethyl ester group of 2ag proceeded smoothly under basic conditions, giving the corresponding free acid 2bb in 98%. Subsequent coupling of 2bb with C-protected diphenylalanine furnished conjugate 13 in moderate yield, illustrating the feasibility of amide-bond formation for further functional applications. Finally, starting from the 7-bromo derivative 2au, we prepared phosphine oxide 14 in 78% yield, through a palladium catalysed reaction with diphenylphosphino oxide. In all cases, the enantiomeric ratios of products 11–14 were retained and matched those of the corresponding starting materials.

**Scheme 4:**
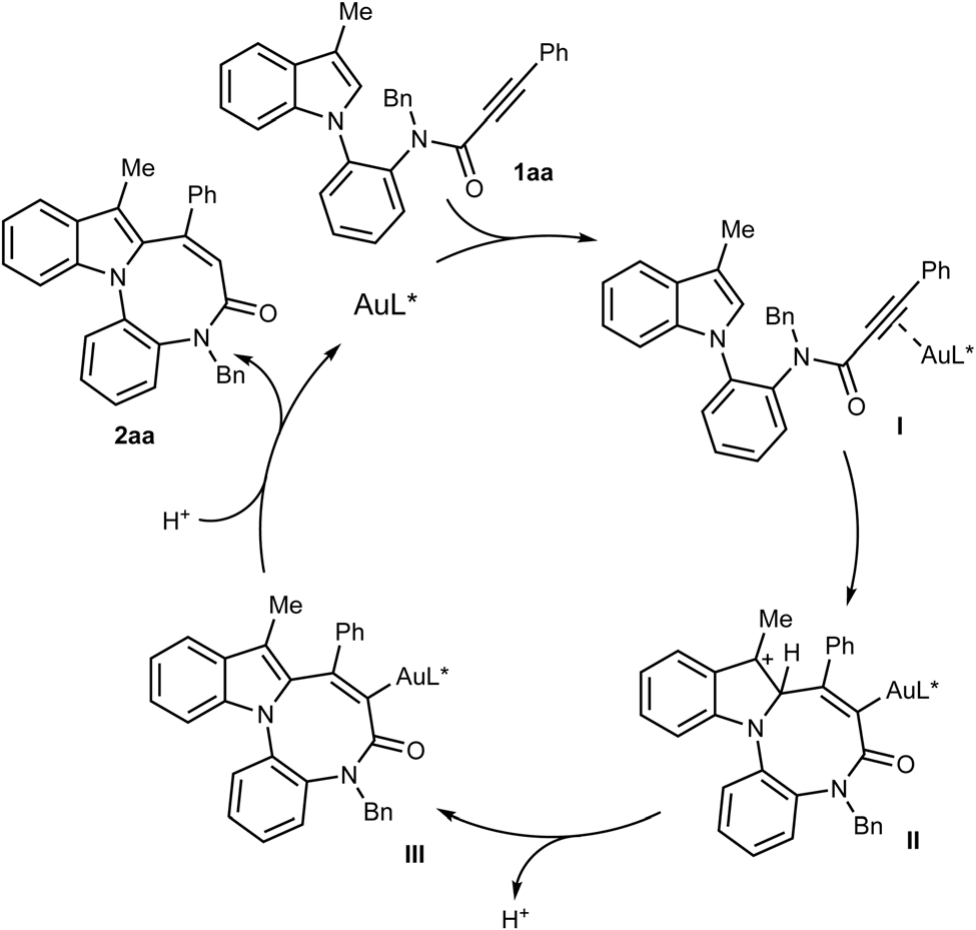
Proposed reaction mechanism.

On the basis of previous investigations on gold-catalysed intramolecular hydroarylation of propiolamides,^19,20^ and on gold-catalysed cyclisations of *N*-(2-aminophenyl)indoles^21^ or *N*-tethered allenylindoles,^22^ we propose that the cyclisation of 1aa proceeds as depicted in Scheme 4. Upon activation of the triple bond by the cationic gold(i) species (intermediate I), an intramolecular 8-*endo*-dig cyclization at the indole C^2^ position takes place to give intermediate II. Subsequent rearomatisation (intermediate III) and protodeauration finally afford the azocinoindole derivative 2aa.

### Computational studies

First, to investigate the configurational stability around the *N*-biaryl axis in products (*S*)-2aa–ba, the rotational barrier was assessed computationally. The calculated enantiomerization free energy barrier
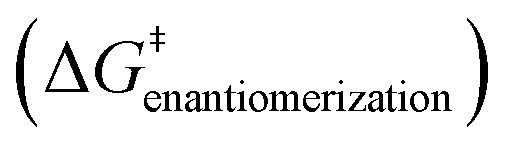
 for compound (*S*)-2aa was 36.9 kcal mol^−1^ (Table S6). This value is considerably higher than the 24–32 kcal mol^−1^ range previously reported for similar derivatives, for which good agreement between theoretical and experimental data was found.^9*b*^ The high calculated barrier for (*S*)-2aa suggested significant thermal stability, which was subsequently verified experimentally. Heating a solution of (*S*)-2aa in various solvents at temperatures up to 190 °C for 48 hours resulted in no observable change in its enantiomeric ratio, confirming the exceptional configurational stability of the *N*-biaryl axis (Table S4). Next, to gain further insight into the origin of the experimentally observed enantioselectivity, the diastereoisomeric cyclic intermediates II and III, reactants (1aa and the AuL catalyst), and product 2aa of Scheme 4 were modelled at DFT level. The calculated free energy for the formation of the initial cyclic intermediate II slightly favours the minor product's precursor, (*R*)-II, by 0.9 kcal mol^−1^ relative to (*S*)-II. To understand this stage at the electronic level, a Quantum Theory of Atoms in Molecules (QTAIM) analysis was performed^23^ to characterize their non-covalent interactions (NCIs).^24,25^

A full description of QTAIM results is provided in the SI. Briefly, despite a slightly higher computed free energy of formation, (*S*)-II features a more extensive and stabilizing network of 36 NCIs compared to 30 found for (*R*)-II (Fig. S1, S2 and Table S8). This enhanced stabilization is quantitatively supported by a more negative total potential energy density (Σ*V*(r) = −0.1452 a.u.) and higher total electron density (Σ*ρ* = 0.2573 a.u.) relative to (*R*)-II (Σ*V*(r) = −0.1065 a.u., Σ*ρ* = 0.1948 a.u.). Specifically, (*S*)-II benefits from a unique Au⋯H interaction, a more robust network of O⋯H and C⋯H hydrogen interactions, and a more compact carbon framework stabilised by London dispersion forces. Additionally, mechanistic studies on gold-catalysed indole functionalisations indicate that the reaction proceeds *via* a stepwise pathway that remains reversible up to the proton transfer step, followed by rearomatisation of the indole ring, and final protodeauration.^26^ Therefore, the final stereochemical outcome is likely determined during the proton abstraction and rearomatisation step that convertes intermediate II into III. A complete kinetic model for this proton transfer would require locating transition states explicitly accounting for the SbF_6_^−^ counterion. Moreover, as HSbF_6_ is a superacid, the proton abstraction cannot be directly mediated by SbF_6_^−^ itself; rather, it would require shuttle-molecules, such as trace water molecules, as reported for related reactions.^27^ While modelling such an explicit network at the DFT level is theoretically feasible, it introduces a very large number of degrees of freedom, rendering it impractical and beyond the scope of the present study.

Thus, we evaluated the steric preorganisation and morphological accessibility of the intermediates II (Fig. 1). In (*S*)-II, the spatial arrangement of the phosphoramidite ligand creates a relatively wide and accessible pocket above the Au atom (Fig. 1A and C), that is structurally suitable to accommodate the counterion together with potential proton-shuttles bridging the indole C^2^-proton and the vinyl gold moiety. In contrast, in the (*R*)-II isomer, the trajectory to the C^2^-proton is physically blocked by the ligand framework, narrowing the accessible gap to just 3.9–4.7 Å (Fig. 1B and D). This morphological divergence exemplifies the ligand-induced folding effect, previously identified as a key stereocontrolling element in related gold-catalysed indole functionalisations.^26*a*^ Accordingly, while the “open” folded conformation of (*S*)-II seamlessly accommodates the SbF_6_^−^ counterion, the lower-energy state of the (*R*)-II isomer adopts a sterically “closed” conformation. Consequently, reaction along the (*R*)-pathway requires an energetic penalty to access an open conformation able to host the counterion and the proton-shuttle machinery. This steric gating would favour the (*S*)-pathway, enabling selective proton abstraction and progression to the rearomatised intermediate III, perfectly aligning with the thermodynamic preferences of the resulting species. Calculations show that (*S*)-III is more stable than (*R*)-III, with a ΔΔ*G* of 2.1 kcal mol^−1^ favouring the (*S*)-isomer. This thermodynamic preference is corroborated by QTAIM analysis, which confirms that (*S*)-III maintains the stabilizing NCI network (Σ*V*(r) = −0.1300 a.u. *vs.* −0.1185 a.u. for (*R*)-III) observed in II (Fig. S3, S4 and Table S9). Collectively, the computational data indicate that the (*S*)-pathway benefits from a highly organized internal network of non-covalent interactions that reinforces the decisive steric gating mechanism.

### Optical properties and interaction with DNA

The diazocino-indole derivatives reported here feature a highly conjugated π system incorporating an indole moiety, a motif ubiquitous in nature and well known for its interaction with DNA.^28^ Recent molecular docking studies have shown that indoles engage in π–π stacking and preferentially intercalate between G–C base pair.^29^ Moreover, their environment-sensitive fluorescence has been widely exploited in the development of chemosensor^30^ and biomolecular labels.^31^ On this ground, and considering the ability of chiral small molecules to recognize specific DNA secondary structures,^32^ we investigated the spectroscopic features of our compounds to assess their potential application as fluorescent probes for DNA structure recognition. Notably, molecules capable of selectively binding defined DNA topologies may also serve as bioactive agents (*e.g.*, G_4_-DNA ligands as anticancer compounds)^33^ or molecular sensors.^34^ Accordingly, selected derivatives (2aa, 2aj, 2ak, 2ar, and 2as) were examined by UV-vis and fluorescence spectroscopy.

**Fig. 2:**
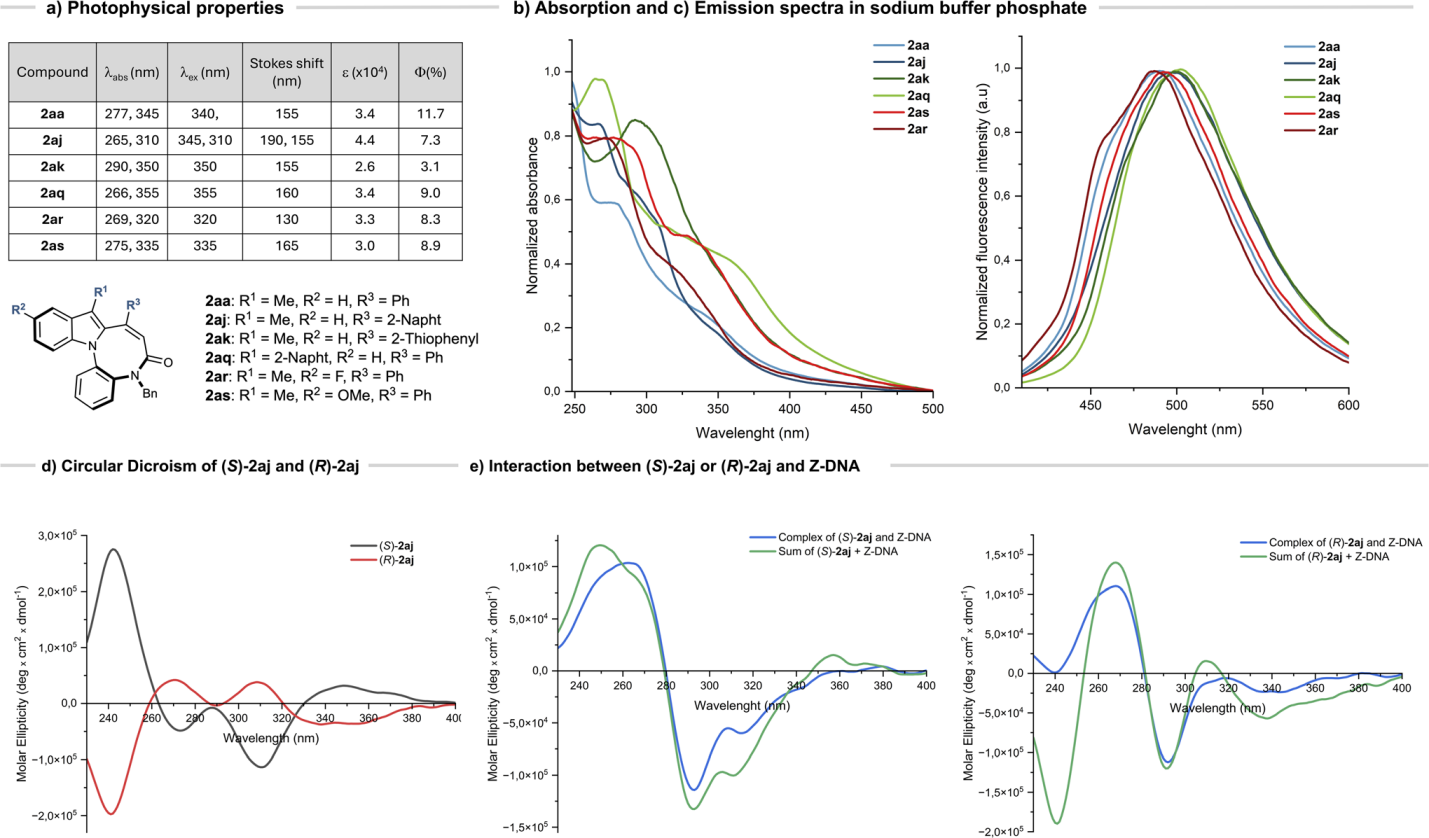
(a) Table of photophysical properties of compounds 2aa, 2aj, 2ak, 2aq, 2ar and 2as. (b) UV-Absorption spectra, 20 µM solutions in sodium phosphate buffer (10 mM, pH = 7.4 + 5% DMSO). (c) Fluorescence emission spectra, 20 µM solutions in sodium phosphate buffer (10 mM, pH = 7.4 + 5% DMSO). (d) CD of (*S*)-2aj and (*R*)-2aj, 10 µM solutions in phosphate buffer (10 mM, pH = 7.4 + 5% DMSO). (e) CD showing the interaction between (*S*)-2aj and (*R*)-2aj with Z-DNA (blue line). The sum spectrum (2aj + Z-DNA) is also reported (green line). Spectra were recorded in sodium phosphate buffer (10 mM pH = 7.4, 1 M Mg(ClO_4_)_2_ + 5% DMSO.

The molar extinction coefficients, as determined in phosphate buffer, as well as the absolute quantum yields and the excitation and emission fluorescence spectra are shown in Fig. 2a–c. The absorption profile of indole typically features three bands around 270, 280, and 290 nm.^30^

The compounds analysed show comparable patterns: a band centred at 275 nm is evident for 2aa and 2as in both buffer and dichloromethane (Fig. 2b and S5a). In 2ar, the absorption maximum at 269 nm is blue-shifted relative to 2aa, consistent with replacement of hydrogen by fluorine at the R^2^-position of the indole. Additionally, a broad band above 300 nm is observed, likely arising from the combined contributions of multiple aromatic rings. Compounds 2aq and 2as display distinct maxima at 315 and 325 nm, respectively. The ability of the synthesised compounds to emit fluorescence in aqueous conditions was then evaluated. Initial measurements were performed upon excitation at the absorption maxima identified in the UV spectra. Weak emissions were observed at 495 nm upon excitation at 275 nm. The excitation spectra recorded setting the emission at 495 nm revealed a broad peak, with one or two maxima between 310 and 355 nm, as reported in Fig. 2a. These values were employed for recording emission spectra (Fig. 2c). We next assessed, by fluorescence and circular dichroism (CD) spectroscopy, the ability of 2aj to interact with DNA. Compound 2aj was selected since it bears a planar naphthyl ring, which could further promote the interaction with DNA by intercalation. Moreover, 2aj is the only derivative with R^1^ = Me and R^2^ = H displaying two well-resolved excitation maxima (310 and 345 nm), whereas all other analogues show a wide excitation spectrum, with a unique maximum, around 345 nm (Fig. S6). Based on the chemical structure and literature data^35,36^ we hypothesise that the maximum at 310 is ascribed to the naphthyl chromophore. Excitation at either 310 or 345 nm afforded identical emission profiles, with *λ*_em(max)_ = 495 nm. Experiments were carried out on both enantiomers (*S* and *R*), to verify whether the chirality could affect the interaction mode with DNA. We selected from literature well characterized DNA sequences; (CG)_6_ was exploited for studies with B- and Z-DNA,^37^ while a 18-mer sequence derived from the *c*-MYC promoter, rich in guanine, was used to produce the quadruplex structure (G_4_).^38^ We compared the spectra of (*R*)- and (*S*)-2aj in buffer with those of the respective enantiomers incubated with DNA (Fig. S8 and S9). Upon interaction with all DNA sequences, the maximum emission wavelength did not change in a significative fashion, whereas the intensity of the signal changed and in all cases it increased. Notably, (*S*)-2aj incubated with B-DNA showed a more pronounced intensity change upon excitation at 310 nm than at 345 nm, suggesting a specific involvement of the naphthyl unit in binding. Furthermore, based on the higher fluorescence emission observed, we may speculate that (*R*)-2aj preferentially interacts with quadruplexes *vs.* duplexes, whereas (*S*)-2aj did not display a similarly marked trend (Fig. S8–S10). Overall, fluorescence data suggest that both (*R*)- and (*S*)-2aj interact with DNA though additional studies are required to confirm the preference of (*R*)-2aj for quadruplexes for a practical exploitation.

CD spectra were then recorded for (*R*)- and (*S*)-2aj, for the oligonucleotides, and for the complexes with DNA. CD spectrum of (*S*)-2aj exhibits one intense positive band at 250 nm and two negative bands at 270 and 310 nm, in line with its absorption features. The enantiomer (*R*)-2aj exhibits, as expected, a CD spectrum specular to that of (*S*)-2aj (Fig. 2d) while CD spectra of B-, Z- and G_4_-DNA are consistent with those reported in the literature (Fig. S11). Fig. 2e and S12–S13 show the CD spectra of the (*R*)- and (*S*)-2aj/DNA complexes alongside the ‘sum spectrum’, obtained by adding the spectra of the individual components. The marked differences observed suggest that (*R*)- and (*S*)-2aj interacts with B-DNA and Z-DNA, as the bands of the superimposed spectra exhibit different intensity and/or wavelength. Of note, the band centred at 240 nm in the (*R*)-2aj and (*S*)-2aj complexes with Z-DNA (Fig. 2e) differs significantly by the same band in the sum spectrum. As this band is due to the contribution of 2aj, we may hypothesise that 2aj adapts its structure to Z-DNA. The interaction of 2aj enantiomers with the quadruplex structure may be hypothesised based on the differences in the intensity of the signal around 240 nm; in this case the changes are not so evident, as the intensity in the signal of the DNA is very high compared to that of 2aj, reasonably due to the higher number of chromophores in the quadruplex. Together, the CD data corroborate the fluorescence results, indicating that (*R*) and (*S*)-2aj interacts with DNA and suggest that the secondary structure of the oligonucleotides is not affected.

## Conclusions

We have developed a gold(i)-catalysed atroposelective intramolecular cyclisation that enables the direct synthesis of axially chiral indole-fused diazocines. This strategy addresses long-standing challenges in the enantioselective construction of medium-sized *N*-bridged biaryls and provides efficient access to highly rigid, configurationally stable C–N atropisomers. The synergistic combination of a BINOL-derived phosphoramidite ligand and cationic gold activation is key to achieving high efficiency and excellent enantioselectivities across a broad substrate scope. Structural analyses and DFT calculations elucidate the origin of stereocontrol, indicating that enantioselectivity is governed by a steric gating mechanism operating within a ligand-induced chiral pocket. Complementary QTAIM analysis further supports this picture by showing that a highly organized network of intramolecular non-covalent interactions preferentially stabilizes the (*S*)-pathway and its associated intermediates, thereby reinforcing the observed atroposelectivity. The synthetic utility of the resulting diazocines is demonstrated by their scalable preparation, broad derivatisation potential, and comprehensive spectroscopic characterisation. In addition, both enantiomers of 2aj exhibit measurable interactions with B-, Z-, and G_4_-DNA, as evidenced by fluorescence and circular dichroism studies, suggesting their potential for applications in biomolecular labelling under aqueous conditions, without perturbing nucleic acid secondary structures. Overall, this work establishes the first general catalytic entry to indole-fused diazocine atropisomers and expands the boundaries of atroposelective cyclisation chemistry toward medium-sized *N*-bridged systems, opening new opportunities for the design of atropisomeric scaffolds relevant to asymmetric catalysis, molecular recognition, and medicinal chemistry.

## Author contributions

S. M.: conceptualisation, investigation, validation, data curation, writing – review & editing. A. R.: conceptualisation, investigation, resources, validation, writing – original draft. P. I.: investigation, data curation. S. R.: investigation, data curation, resources, writing – review & editing. A. C.: conceptualisation, formal analysis, data curation, resources, validation, writing – original draft. G. A.: conceptualisation, resources, funding aquistion, writing – review & editing. V. P.: conceptualisation, investigation, validation, funding acquisition, resources, supervision, writing – original draft.

## Conflicts of interest

There are no conflicts to declare.

## Supplementary Material

SC-017-D6SC00020G-s001

SC-017-D6SC00020G-s002

## Data Availability

CCDC 2497353 contains the supplementary crystallographic data for this paper.^39^ All data supporting the findings of this study are available within the article and the supplementary information (SI). Supplementary information: the SI includes full experimental procedures, compound characterisation data (NMR, HRMS, chiral HPLC), computational details (DFT energies, QTAIM analyses, and Cartesian coordinates), spectroscopic data (UV-vis, fluorescence, circular dichroism), and DNA interaction studies. Additional data are available from the corresponding author upon reasonable request. See DOI: https://doi.org/10.1039/d6sc00020g.
