# Supplementary material for: Atroposelective construction of indole-fused diazocines via gold(i)-catalysed 8-endo-dig cyclisation
Source: Chem Sci. 2026 Apr 28;17(23):11580–90. doi: 10.1039/d6sc00020g (PMC13141309; doi:10.1039/d6sc00020g)

Supplementary Information (SI) for Chemical Science.
This journal is © The Royal Society of Chemistry 2026

Electronic supplementary information

Atroposelective construction of indole-fused diazocines via gold(I)-catalysed 8-endo-dig cyclisation

Silvia Meraviglia,[a] Alessandra Romanelli,[a] Paola Iannelli,[a] Silvia Rizzato,[b] Alessandro Contini,*[a] Giorgio Abbiati[a] and Valentina Pirovano*[a]

[a]Dipartimento di Scienze Farmaceutiche, Sezione di Chimica Generale e Organica "A. Marchesini", Università degli Studi di Milano, Via C. Golgi 19, 20133, Milano (Italy). e-mail: valentina.pirovano@unimi.it.
[b] Dipartimento di Chimica, Università degli Studi di Milano, Via C. Golgi 19, 20133, Milano (Italy).

Table of contents

General remarks S-2
Screening of the reaction conditions S-3
Racemization experiments S-4
Synthesis of the starting materials S-5
Synthesis of differently substituted starting materials S-21
Synthesis of other stating materials S-24
Catalytic reactions S-26
Transformations of the final products S-57
Computational studies S-62
Spectroscopic characterization S-85
X-ray crystallographic data S-92
References S-93
NMR spectra of new compounds S-94

General remarks

All the reactions, that involve the use of reagents sensitive to oxygen or hydrolysis, were carried out under a nitrogen atmosphere. The glassware was previously dried with a heating gun and set with cycles of vacuum and nitrogen. All chemicals and solvents are commercially available and were used without further purification. Catalysts $L(AuCl)_2$ and LAuCl are known compounds and were prepared according to literature procedures.[1]

^{1}H NMR analyses were performed with 300, 400, 500 or 600 MHz spectrometer at room temperature or stated temperature. The coupling constants (J) are expressed in Hertz (Hz), the chemical shifts (δ) in ppm. The multiplicities of the proton spectrum were described by the following abbreviations: s (singlet), d (doublet), t (triplet), q (quartet), p (quintet), dt (double triplet), dd (double doublet), m (multiplet), br (broad). ^{13}C NMR analyses were performed with the same instruments at 75, 101, 126 and 151 MHz; APT sequence was used to distinguish the methine and methyl carbon signals from those arising from methylene and quaternary carbon atoms. All ^{13}C NMR spectra were recorded with complete proton decoupling.

High resolution mass spectra (HR-MS) were acquired on a Synapt G2-Si QTof mass spectrometer (Waters, Milford, MA, USA) equipped with a Zspray ESI-probe (Waters) for electrospray ionization in positive polarity and full scan mode. Data were processed using MassLynx v4.2 software (Waters).

HPLC chromatograms were recorded on Merck-Hitachi HPLC LaChrom, with HP Hewlett Packard detector series 1050 and Pump L-7100 or on LC-4000 Series HPLC with MD-4010 detector and PU-4180 pump, using Chiracel OD-H 5µ Cellulose (250 x 4.60 mm).

UV spectra were recorded on a Shimadzu UV-3600 spectrophotometer.

Fluorescence spectra were recorded on a Fluorolog Jobin Horiba instrument.

Photoluminescence quantum yields were measured using a C11347 Quantaurus – Absolute Photoluminescence Quantum Yield Spectrometer (Hamamatsu Photonics K.K), equipped with a 150 W Xenon lamp, an integrating sphere and a multichannel detector.

CD spectra were recorded on a Jasco J-810 instrument.

Screening of the reaction conditions

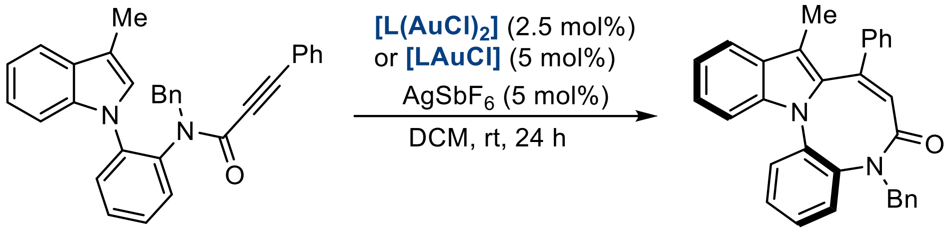

1aa **2aa**

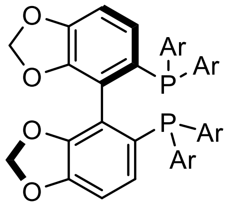

L_1: Ar = 3,5-(*t*-Bu)$_2$-4-OMe-C_6H_2
L_2: Ar = 3,5-(Me)$_2$-C_6H_3

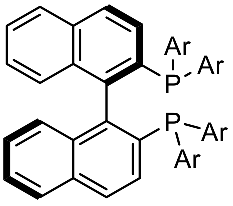

L_3: Ar = 3,5-(Me)$_2$-C_6H_3

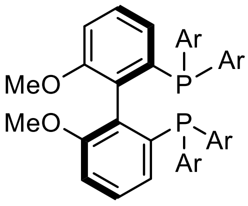

L_8: Ar = 3,5-(*t*-Bu)$_2$-4-OMe-C_6H_2

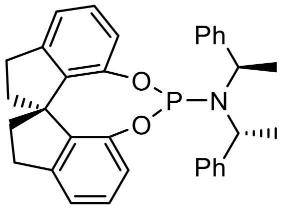

L_4

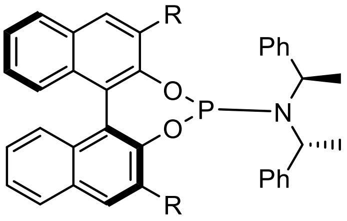

L_5: R = 2-Naphtyl
L_6: R = 9-Pyrenyl
L_7: R = 3,5-(CF_3)$_2$-C_6H_3
L_9: R = 2,4,6-(*i*Pr)$_3$-C_6H_2
L_{10}: R = 1-Naphtyl
L_{11}: R = 9-Antracenyl

Table S1: Screening of the chiral catalysts

Entry[a]	[Au]	Deviations	**2a** (%)[b]	e.r.[c]
1	$L_1(Au_2Cl_2)$	-	-	-
2	$L_2(Au_2Cl_2)$	48 h	17	70:30
3	$L_3(Au_2Cl_2)$	48 h	20	55:45
4	L_4AuCl	-	82	52:48
5	L_5AuCl	-	75	30:70
6	L_6AuCl	48 h	71	25:75
7	L_7AuCl	-	99	8:92
8	$L_8(Au_2Cl_2)$	-	-	-
9	L_9AuCl	48h	26	28:72
10	L_{10}AuCl	48h	56	35:65
11	L_{11}AuCl	48h	-	-

Table S2: Screening of the counterions

Entry[a]	[Au]	Deviations	**2a** (%)[b]	e.r.[c]
12	L_7AuCl	$AgNTf_2$	78	7:93
13	L_7AuCl	$AgBF_4$	66	5:95
14	L_7AuCl	AgOTf, 48 h	41	7:93
15	L_7AuCl	AgBArF, 48 h	-	-

Table S3: Screening of the solvents

Entry[a]	**[Au]**	Deviations	**2a** (%)[b]	e.r.[c]
16	$\mathbf{L_7}$AuCl	Toluene, 48 h	38	2:98
17	$\mathbf{L_7}$AuCl	Chlorobenzene	94	2:98
18	$\mathbf{L_7}$AuCl	$AgBF_4$, Toluene, 48h	-	-
19	$\mathbf{L_7}$AuCl	DCE	95	10:90

[a] Unless otherwise stated, reactions were carried out with **1aa** (0.1 mmol), gold catalyst (2.5 or 5 mol%), $AgSbF_6$ (5 mol%) in anhydrous DCM (1 ml, 0.1 M) at room temperature for 24 h. [b] Isolated yields. [c] Enantiomeric ratios (e.r.) determined by chiral HPLC.

Racemization experiments

5 mg of **2aa** were dissolved in 5 mL of 1,2-dichlorobenzene and heated at reflux (190 °C). Aliquots (50 µL) were diluted with 0.2 mL of the eluent (*i*PrOH/Hex 15/85) and directly analysed by chiral HPLC to determine the enantiomeric excess at the specified time.

Table S4: effect of heating on the enantiomeric excess for product **2aa**.

	t(s)	e.e.
1	0	96%
2	600	96%
3	1200	96%
4	1800	96%
5	3600	96%
6	10800	96%
7	21600	96%
8	86400	96%
9	172800	96%

Synthesis of the starting materials

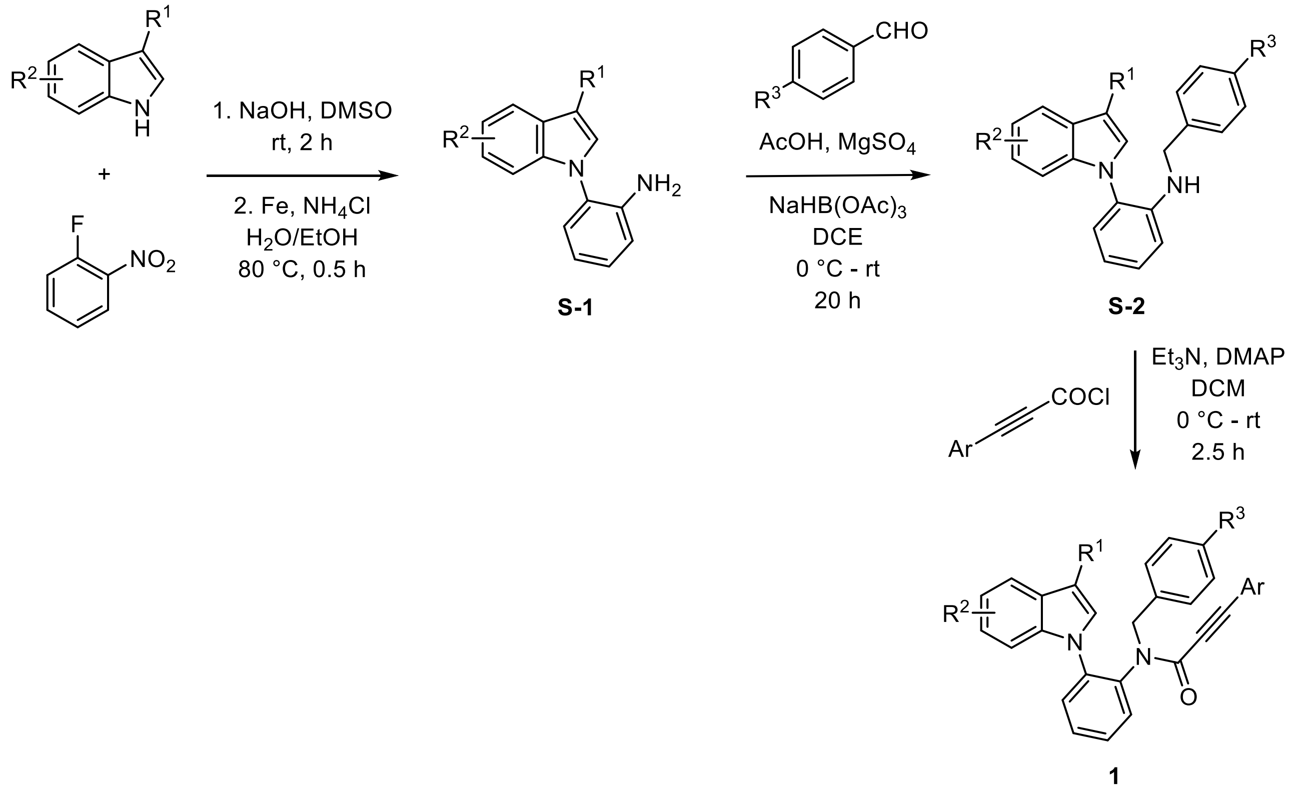

General procedure for the synthesis of 2-(1*H*-indol-1-yl)anilines (S-1a-i)

To the stirred solution of the appropriate 1*H*-indole (1 equiv.) in dry DMSO (0.85 M), 1-fluoro-2-nitrobenzene (1.1 equiv.) and NaOH (1.5 equiv.) were added under nitrogen atmosphere and the reaction mixture was stirred for 2 h. Upon completion of reaction, the reaction mixture was poured in H_2O and AcOEt. The aqueous layer was extracted with AcOEt and the combined organic layers were dried over Na_2SO_4 and concentrated in vacuo. The crude product was directly used for the next step.

A mixture of 1-(2- nitrophenyl)-1*H*-indole (1 equiv.), ammonium chloride (0.5 equiv.) and Fe (5 equiv.) in ethanol (0.1 M) and water (0.4 M) was stirred at 80 °C for 30 min. Upon completion of reaction, the reaction mixture was filtered through a bed of celite, concentrated under vacuum and extracted with H_2O and AcOEt. The combined organic layers were washed with Na_2SO_4 and concentrated under vacuum to yield **S-1**.

General procedure for the synthesis of *N*-benzyl-2-(1*H*-indol-1-yl)anilines (S-2a-j)

Under nitrogen atmosphere **S-1** (1 equiv.) and benzaldehyde or 4-methoxybenzaldehyde (1.2 equiv) were stirred in 1,2-dichloroethane (0.6 M). Then $MgSO_4$ (0.8 equiv) and acetic acid (0.1 equiv) were added. The reaction mixture was stirred at room temperature for 2h. Then the reaction mixture was cooled down to 0 °C and sodium triacetoxyborohydride (2 equiv.) was added portionwise. The mixture was reacted at room temperature for 17 hours. After completion of the reaction, the mixture was quenched with water, extracted with DCM, and the organic phases were combined and dried over anhydrous sodium sulfate. The organic phase was concentrated under reduced pressure. Purification of the crude by flash column chromatography yielded the corresponding benzylated derivatives **S-2.**

General procedure for the synthesis of *N*-benzyl-*N*-(2-(1*H*-indol-1-yl)phenyl)-3-arylpropiolamides (1aa-ak, 1an-av, 1ax-ba)

Under nitrogen atmosphere **S-2** (1 equiv.) and DMAP (0.05 equiv.) were dissolved in DCM (0.1 M) at 0 °C. Then Et_3N (2 equiv.) was added dropwise followed by a solution of different propioloyl chlorides (1.5 equiv) in DCM, which were prepared following a previously reported procedure[2] from the corresponding propiolic acids.[3] The reaction was left stirring for 0.5 h at 0 °C and then at rt for 2h. The reaction mixture was quenched with a solution of $NaHCO_3$ and extracted three times with DCM. The organic phases were washed with brine and evaporated under reduced pressure. Purification by flash column chromatography (SiO_2) yielded the corresponding derivatives **1**.

2-(3-methyl-1*H*-indol-1-yl)aniline (S-1a)

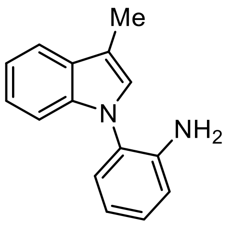

Synthetized from 3-methyl-1*H*-indole (2.04 g, 15.5 mmol), 1-fluoro-2-nitrobenzene (1.8 mL, 17.1 mmol), NaOH (930 mg, 23.3 mmol) and 18 mL of DMSO. The crude product (3.9 g, 15.5 mmol) directly used for the next step, adding Fe (4.32 g, 77.3 mmol), NH_4Cl (414 mg, 7.73 mmol), 116 mL of EtOH and 38 mL of water. The crude was purified by chromatography with Biotage® Selekt (Hex/AcOEt 98:2 → 80:20) to yield **S-1a** as an orange sticky oil (2.39 g, 69%).

^{1}H NMR (300 MHz, $CDCl_3$): 7.64 – 7.56 (m, 2H), 7.39 – 7.33 (m, 2H), 7.25 – 7.09 (m, 3H), 6.98 (dt, *J* = 2.2, 1.1 Hz, 2H), 2.36 (s, 3H). Data are in agreement with those reported in literature.[4]

2-(3-ethyl-1*H*-indol-1-yl)aniline (S-1b)

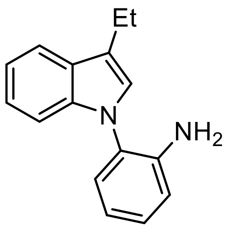

Synthetized from 3-ethyl-1*H*-indole[5] (658 mg, 4.53 mmol), 1-fluoro-2-nitrobenzene (526 µL, 4.98 mmol), NaOH (272 mg, 6.80 mmol) and 5.3 mL of DMSO. The crude product (1.19 g, 4.51 mmol) was directly used for the next step, adding Fe (1.26 g, 22.5 mmol), NH_4Cl (120 mg, 2.25 mmol), 45 mL of EtOH and 11 mL of water. Extraction of the reaction mixture yielded **S-1b** as an orange sticky oil (1.02 g, 95%). The crude was directly used for the next step without further purification.

^{1}H NMR (300 MHz, $CDCl_3$): 7.69 (m, 1H), 7.25 (m, 1H), 7.22 (m, 1H), 7.20 – 7.17 (m, 2H), 7.16 – 7.12 (m, 1H), 7.02 (t, *J* = 1.1 Hz, 1H), 6.93 – 6.78 (m, 2H), 3.61 (bs, 2H), 2.87 (qd, *J* = 7.5, 1.1 Hz, 2H), 1.40 (t, *J* = 7.5 Hz, 3H). **^{13}C NMR** (75 MHz, $CDCl_3$): 143.13 (C), 136.80 (C), 128.85 (CH), 128.60 (CH), 128.16 (C), 125.20 (C), 124.99 (CH), 122.18 (CH), 119.47 (C), 119.43 (CH), 119.13 (CH), 118.52 (CH), 116.20 (CH), 110.70 (CH), 18.35 (CH_2), 14.37 (CH_3). **HRMS** (ESI) calculated for $C_{16}H_{17}N_2$ $[M+H]^+$ requires m/z = 237.1387, found m/z 237.1385.

2-(3-isopropyl-1*H*-indol-1-yl)aniline (S-1c)

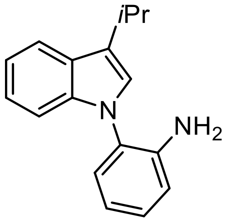

Synthetized from 3-isopropyl-1*H*-indole[6] (820 mg, 5.15 mmol), 1-fluoro-2-nitrobenzene (600 µL, 5.67 mmol), NaOH (309 mg, 7.73 mmol) and 6 mL of DMSO. The crude product (1.55 g, 5.15mmol) was directly used for the next step, adding Fe (1.44 g, 25.75 mmol), NH_4Cl (138 mg, 2.58 mmol), 51.5 mL of EtOH and 13 mL of water. Extraction of the reaction mixture yielded **S-1c** as a red sticky oil (1.17 g, 91%). The crude was directly used for the next step without further purification.

^{1}H NMR (300 MHz, $CDCl_3$): 7.74 (m, 1H), 7.30 – 7.05 (m, 5H), 7.01 (d, *J* = 1.0 Hz, 1H), 6.93 – 6.77 (m, 2H), 3.61 (bs, 2H), 3.30 (hept, *J* = 1.0 Hz, 1H), 1.43 (d, *J* = 6.9 Hz, 6H). **^{13}C NMR** (75 MHz, $CDCl_3$): 143.14 (C), 136.96 (C), 128.86 (CH), 128.62 (CH), 127.54 (C), 125.22 (C), 124.65 (C), 123.88 (CH), 122.09 (CH), 119.58 (CH), 119.35 (CH), 118.52 (CH), 116.19 (CH), 110.80 (CH), 25.56 (CH), 23.36 (CH_3). **HRMS** (ESI) calculated for $C_{17}H_{19}N_2$ $[M+H]^+$ requires m/z = 251.1543, found m/z 251.1545.

2-(3-phenyl-1*H*-indol-1-yl)aniline (S-1d)

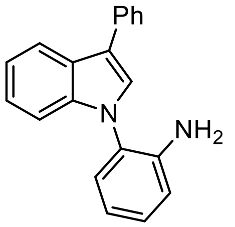

Synthetized from 3-phenyl-1*H*-indole[7] (402 mg, 2.1 mmol), 1-fluoro-2-nitrobenzene (240 µL, 2.3 mmol), NaOH (125 mg, 3.1 mmol) and 2.5 mL of DMSO. The crude product (647 mg, 2.1 mmol) was directly used for the next step, adding Fe (597 mg, 10.2 mmol), NH_4Cl (55 mg, 1.02 mmol), 20 mL of EtOH and 5.1 mL of water. Extraction of the reaction mixture yielded **S-1d** as an orange sticky oil (463 mg, 80%). The crude was directly used for the next step without further purification.

^{1}H NMR (300 MHz, $CDCl_3$): 8.01 (m, 1H), 7.77 – 7.67 (m, 2H), 7.52 – 7.43 (m, 2H), 7.41 (s, 1H), 7.32 (m, 1H), 7.29 – 7.26 (m, 2H), 7.26 – 7.23 (m, 2H), 7.20 (m, 1H), 6.89 (m, 2H), 3.66 (bs, 2H). Data are in agreement with those reported in literature.[8]

2-(3-(naphthalen-2-yl)-1*H*-indol-1-yl)aniline (S-1e)

Synthetized from 3-(naphthalen-2-yl)-1*H*-indole, which is a known compound, but it was prepared through a different procedure from those reported in literature: in a Schlenk vial, $Pd(OAc)_2$ (34 mg, 0.15 mmol), dppm (58 mg, 0.15 mmol), LiOH•H_2O (378 mg, 9 mmol), 2-bromonaphtalene (621 mg, 3 mmol), were added to a stirring solution of 1*H*-indole (351 mg, 3 mmol) in 6 mL of degassed water (0.5 M). The resulting suspension was heated up at 110 °C for 24 h. Then, 15 mL of HCl 1 N were added and the mixture was extracted with AcOEt. The organic phase was combined, dried over Na_2SO_4 and concentrated under vacuum. Purification of the crude by flash column chromatography (SiO_2, Hex/AcOEt 9:1 → 7:1) yielded 3-(naphthalen-2-yl)-1*H*-indole (372 mg, 51%), as a yellow solid.

^{1}H NMR (300 MHz, $CDCl_3$): 8.28 (s, 1H), 8.15 (s, 1H), 8.08 (dd, *J* = 8.1, 0.9 Hz, 1H), 7.97 – 7.77 (m, 4H), 7.55 – 7.41 (m, 4H), 7.35 – 7.18 (m, 2H). Data are in agreement with those reported in literature.[9]

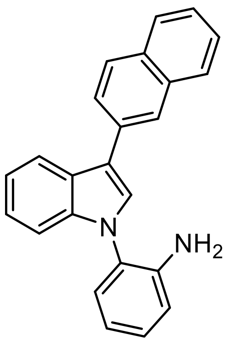

Then 3-(naphthalen-2-yl)-1*H*-indole (730 mg, 3 mmol) was reacted with 1-fluoro-2-nitrobenzene (348 µL, 3.3 mmol), NaOH (180 mg, 4.5 mmol) and 3.5 mL of DMSO through the standard procedure. The crude product was directly used for the next step, adding (1.09 g, 3 mmol), Fe (838 mg, 15 mmol), NH_4Cl (80 mg, 1.5 mmol), 30 mL of EtOH and 7.5 mL of water. Extraction of the reaction mixture yielded **S-1e** as a yellow solid (943 mg, 94%). The crude was directly used for the next step without further purification.

^{1}H NMR (300 MHz, $CDCl_3$): 8.19 (m, 1H), 8.14 (m, 1H), 7.98 – 7.80 (m, 4H), 7.53 (d, *J* = 0.7 Hz, 1H), 7.53 – 7.43 (m, 2H), 7.35 – 7.17 (m, 5H), 7.00 – 6.83 (m, 2H), 3.69 (bs, 2H). Data are in agreement with those reported in literature.[10]

2-(5-fluoro-3-methyl-1*H*-indol-1-yl)aniline (S-1f)

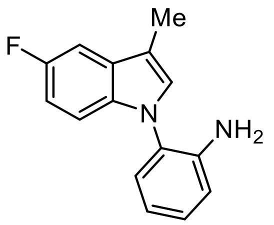

Synthetized form 5-fluoro-3-methyl-1*H*-indole through a different procedure. To a Schlenk vial, a solution of 5-fluoro-3-methyl-1*H*-indole[11] (392 mg, 2.6 mmol), CuI (50 mg, 0.26 mmol) and K_3PO_4 (1.17 g, 5.5 mmol) in 5.3 mL of toluene (0.5 M) was stirred under nitrogen atmosphere at room temperature. Then 2-iodoaniline (576 mg, 2.6 mmol) and DMEDA (57 µL, 0.5 mmol) were added. Then, the mixture was heated at 110 °C and stirred for 24 h. Successively, the mixture was cooled to room temperature, filtered over a pad of celite and concentrated under reduced pressure. The crude was purified by flash column chromatography (SiO_2, Hex/DCM 2:1) to yield **S-1f** (320 mg, 51%) as an orange solid.

^{1}H NMR (300 MHz, $CDCl_3$): 7.29 – 7.20 (m, 2H), 7.16 (dd, *J* = 7.8, 1.5 Hz, 1H), 7.06 – 6.97 (m, 2H), 6.96 – 6.75 (m, 3H), 3.76 (bs, 2H), 2.35 (d, *J* = 1.1 Hz, 3H). **^{13}C NMR** (101 MHz, $CDCl_3$): 158.04 (d, *J* = 235.2 Hz, C), 143.08 (C), 133.25 (C), 129.25 (d, *J* = 9.8, C), 129.06 (CH), 128.52 (CH), 127.74 (CH), 124.90 (C), 118.59 (CH), 116.26 (CH), 112.36 (d, *J* = 4.7 Hz, C), 111.37 (d, *J* = 9.5 Hz, CH), 110.43 (d, *J* = 26.2 Hz, CH), 103.95 (d, *J* = 23.3 Hz, CH), 9.62 (CH_3). **HRMS** (ESI) calculated for $C_{15}H_{14}FN_2$ $[M+H]^+$ requires m/z = 204.1136, found m/z 204.1136.

2-(5-methoxy-3-methyl-1*H*-indol-1-yl)aniline (S-1g)

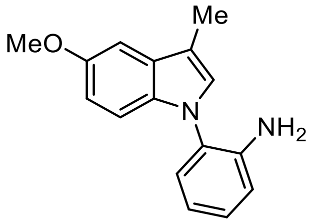

Synthetized from 5-methoxy-3-methyl-1*H*-indole[11] (424 mg, 2.6 mmol), 1-fluoro-2-nitrobenzene (305 µL, 2.9 mmol), NaOH (158 mg, 3.9 mmol) and 3.1 mL of DMSO. Purification of the crude by flash column chromatography (SiO_2, Hex/AcOEt 7:1) yielded 5-methoxy-3-methyl-1-(2-nitrophenyl)-1*H*-indole as a sticky orange oil (650 mg, 2.3 mmol), which was directly used for the next step, adding Fe (642 mg, 11.5 mmol), NH_4Cl (62 mg, 1.15 mmol), 23 mL of EtOH and 5.7 mL of water. Extraction of the reaction mixture yielded **S-1g** as an orange sticky oil (557 mg, 84%). The crude was directly used for the next step without further purification.

^{1}H NMR (300 MHz, $CDCl_3$): 7.29 – 7.15 (m, 2H), 7.10 (d, *J* = 2.4 Hz, 1H), 7.08 – 6.98 (m, 2H), 6.86 (td, *J* = 8.2, 2.0 Hz, 3H), 3.92 (s, 3H), 3.71 (bs, 3H), 2.39 (s, 1H). **^{13}C NMR** (75 MHz, $CDCl_3$): 154.27 (C), 142.77 (C), 131.96 (C), 129.33 (C), 128.76 (CH), 128.50 (CH), 126.79 (CH), 125.46 (C), 118.73 (CH), 116.39 (CH), 112.23 (CH), 112.01 (C), 111.48 (CH), 100.97 (CH), 56.00 (CH_3), 9.74 (CH_3). **HRMS** (ESI) calculated for $C_{16}H_{17}N_2O$ $[M+H]^+$ requires m/z = 253.1336, found m/z 253.1338.

2-(6-bromo-3-methyl-1*H*-indol-1-yl)aniline (S-1h)

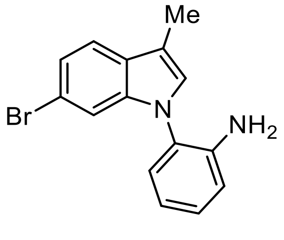

Synthetized from 6-bromo-3-methyl-1*H*-indole[11] (791 mg, 3.77 mmol), 1-fluoro-2-nitrobenzene (437 µL, 4.15 mmol), NaOH (226 mg, 5.66 mmol) and 4.4 mL of DMSO. The crude was purified by chromatography with Biotage® Selekt (Hex/AcOEt 95:5 → 60:40). The purified product (1 g, 3 mmol), was directly used for the next step, adding Fe (838 mg, 15 mmol), NH_4Cl (80 mg, 1.5 mmol), 30 mL of EtOH and 7.5 mL of water. Extraction of the reaction mixture yielded **S-1h** as an orange sticky oil (728 mg, 65%). The crude was directly used for the next step without further purification.

^{1}H NMR (300 MHz, $CDCl_3$): 7.48 (dd, *J* = 8.8, 1.0 Hz, 1H), 7.31 – 7.19 (m, 3H), 7.15 (dd, *J* = 7.7, 1.5 Hz, 1H), 6.97 (q, *J* = 1.1 Hz, 1H), 6.92 – 6.78 (m, 2H), 3.58 (bs, 2H), 2.37 (d, *J* = 1.1 Hz, 3H). **^{13}C NMR** (75 MHz, $CDCl_3$): 143.04 (C), 137.45 (C), 129.26 (CH), 128.51 (CH), 127.82 (C), 126.70 (CH), 124.35 (C), 122.78 (CH), 120.32 (CH), 118.60 (CH), 116.30 (CH), 116.00 (C), 113.51 (CH), 112.60 (C), 9.55 (CH_3**)**. **HRMS** (ESI) calculated for $C_{15}H_{14}BrN_2$ $[M+H]^+$ requires m/z = 301.0335, found m/z 301.0336.

2-(7-bromo-3-methyl-1*H*-indol-1-yl)aniline (S-1i)

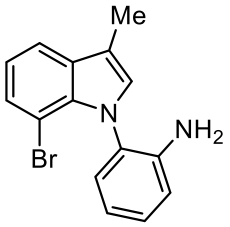

Synthetized from 7-bromo-3-methyl-1*H*-indole[11] (1 g, 4.76 mmol), 1-fluoro-2-nitrobenzene (552 µL, 5.24 mmol), NaOH (286 mg, 7.14 mmol) and 5.7 mL of DMSO. The crude product was directly used for the next step, adding Fe (1.30 g, 23.8 mmol), NH_4Cl (127 mg, 2.38 mmol), 48 mL of EtOH and 4.8 mL of water. Extraction of the reaction mixture yielded **S-1i** as an orange sticky oil (1.38 g, 97%). The crude was directly used for the next step without further purification.

^{1}H NMR (300 MHz, $CDCl_3$)**:** 7.57 (dd, *J* = 7.8, 1.1 Hz, 1H), 7.35 (dd, *J* = 7.6, 1.0 Hz, 1H), 7.26 (td, *J* = 7.5, 1.5 Hz, 1H), 7.16 (dd, *J* = 8.0, 1.5 Hz, 1H), 7.01 (t, *J* = 7.7 Hz, 1H), 6.91 (q, *J* = 1.1 Hz, 1H), 6.86 – 6.75 (m, 2H), 3.50 (s**b**, 2H), 2.36 (d, *J* = 1.1 Hz, 3H). **^{13}C NMR** (75 MHz, $CDCl_3$): 144.99 (C), 133.27 (C), 131.55 (C), 130.17 (CH9, 129.62 (CH), 128.78 (CH), 127.02 (CH), 125.75 (C), 120.61 (CH), 118.39 (CH), 117.91 (CH), 115.42 (CH), 112.39 (C), 104.11 (C), 9.54 (CH_3). **HRMS (ESI)** calculated for $C_{15}H_{14}BrN_2$ $[M+H]^+$ requires m/z = 301.0335, found m/z 301.0336.

3-methyl-2-(3-methyl-1*H*-indol-1-yl)aniline (S-1j)

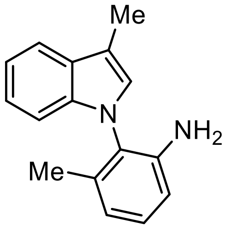

Synthetized from 3-methyl-1*H*-indole (700 mg, 5.34 mmol), 2-fluoro-1-methyl-3-nitrobenzene (911 mg, 5.87 mmol), NaOH (320 mg, 8.00 mmol) and 6.3 mL of DMSO. The crude product (1.12 g, 4.19 mmol) was directly used for the next step, adding Fe (1.17 g, 20.1 mmol), NH_4Cl (110 mg, 2.10 mmol), 42 mL of EtOH and 10 mL of water. Extraction of the reaction mixture yielded **S-1j** as a red sticky oil (980 mg, 73%). The crude was directly used for the next step without further purification.

^{1}H NMR (300 MHz, $CDCl_3$): 7.66 (m, 1H), 7.24 – 7.12 (m, 3H), 7.00 (m, 1H), 6.89 (d, J = 1.2 Hz, 1H), 6.73 (t, J = 7.2 Hz, 2H), 3.50 (s, 2H), 2.43 (d, J = 1.1 Hz, 3H), 1.92 (s, 3H). Data are in agreement with those reported in literature. [12]

3-bromo-2-(3-methyl-1*H*-indol-1-yl)aniline (S-1k)

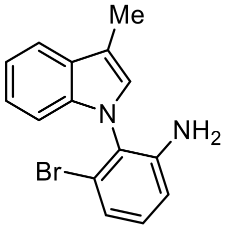

Synthetized from 3-methyl-1*H*-indole (1 g, 7.62 mmol), 1-bromo-2-fluoro-3-nitrobenzene (1.0 mL, 8.38 mmol), NaOH (460 mg, 11.4 mmol) and 9 mL of DMSO. The crude product was directly used for the next step, adding Fe (2.1 g, 38.1 mmol), NH_4Cl (204 mg, 3.81 mmol), 76 mL of EtOH and 7.6 mL of water. Extraction of the reaction mixture yielded **S-1k** as an orange sticky oil (2.1 g, 92%). The crude was directly used for the next step without further purification.

^{1}H NMR (300 MHz, $CDCl_3$): 7.71 – 7.59 (m, 1H), 7.29 – 7.15 (m, 2H), 7.09 (d, *J* = 3.3 Hz, 2H), 7.04 – 6.94 (m, 1H), 6.89 (q, *J* = 1.1 Hz, 1H), 6.78 (dd, *J* = 6.1, 3.4 Hz, 1H), 3.58 (bs, 2H), 2.41 (d, *J* = 1.1 Hz, 3H). **^{13}C NMR** (75 MHz, $CDCl_3$): 146.14 (C), 135.90 (C), 130.26 (CH), 128.85 (C), 125.30 (CH), 124.59 (C), 123.57 (C), 122.38 (CH), 122.14 (CH), 119.76 (CH), 119.08 (CH), 114.71 (CH), 112.93 (C), 110.16 (CH), 9.78 (CH_3). HRMS (ESI) calculated for $C_{15}H_{14}BrN_2$ $[M+H]^+$ requires m/z = 301.0335, found m/z 301.0336.

4-methyl-2-(3-methyl-1*H*-indol-1-yl)aniline (S-1l)

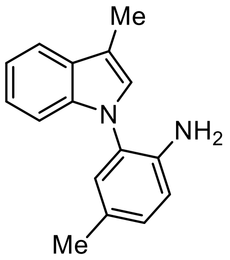

Synthetized form 3-methyl-1*H*-indole through a different procedure. To a solution of 3-methyl-1*H*-indole (1 g, 7.6 mmol) in 15 mL of toluene (0.5 M), CuI (145 mg, 0.76 mmol) and K_3PO_4 (3.4 g, 16 mmol) were added at rt under nitrogen atmosphere. Then 2-iodo-4-methylaniline (1.8 g, 7.6 mmol) and DMEDA (164 µL, 1.52 mmol) were added and the reaction mixture was stirred at 110°C for 48 h. Upon completion of the reaction, the mixture was cooled down to rt, AcOEt was added and the crude was filtered on celite. Purification of the crude with flash column chromatography (SiO_2 Hex/AcOEt 2:1) yielded **S-1l** as a red sticky oil (1.7 g, 94%).

^{1}H NMR (300 MHz, DMSO): 7.56 (m, 1H), 7.13 – 7.04 (m, 3H), 6.98 – 6.90 (m, 2H), 6.71 (s, 1H), 6.47 (m, 1H), 4.61 (s, 2H), 2.32 (s, 3H), 2.26 (s, 3H). Data are in agreement with those reported in literature.[13]

***N*-benzyl-2-(3-methyl-1*H*-indol-1-yl)aniline (S-2a)**

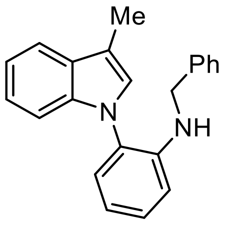

Synthetized from **S-1a** (1.5 g, 6.75 mmol), benzaldehyde (826 µL, 8.10 mmol), $MgSO_4$ (650 mg 5.40 mmol), acetic acid (39 µL, 0.68 mmol), $NaBH(OAc)_3$ (2.86 g, 13.5 mmol) and 11.3 mL of dichloroethane. Purification of the crude by Biotage® Selekt (Hex/AcOEt 95:5→80:20) yielded **S-2a** (1.86 g, 88%) as a white solid.

^{1}H NMR (300 MHz, $CDCl_3$): 7.68 (d, *J* = 6.4 Hz, 1H), 7.38 – 7.13 (m, 10H), 7.05 (s, 1H), 6.84 – 6.76 (m, 2H), 4.35 (s, 2H), 4.20 (bs, 1H), 2.45 (s, 3H). Data are in agreement with those reported in literature.[12]

***N*-benzyl-2-(3-ethyl-1*H*-indol-1-yl)aniline (S-2b)**

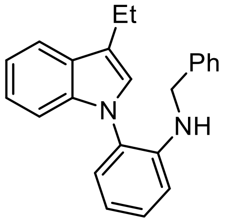

Synthetized from **S-1b** (473 mg, 2 mmol), benzaldehyde (243 µL, 2.4 mL), $MgSO_4$ (193 mg, 1.6 mmol), acetic acid (11 µL, 0.2 mmol), $NaBH(OAc)_3$ (848 mg, 4 mmol) and 3.4 mL of dichloroethane. Purification of the crude by Biotage® Selekt (Hex/AcOEt 95:5 → 85:15), yielded **S-2b** (598 mg, 92%) as a colorless glue.

^{1}H NMR (300 MHz, $CDCl_3$): 7.69 (d, *J* = 7.4 Hz, 1H), 7.35 – 7.13 (m, 10H), 7.03 (s, 1H), 6.82 – 6.74 (m, 2H), 4.32 (s, 2H), 4.18 (bs, 1H) 2.87 (q, *J* = 7.5 Hz, 2H), 1.40 (t, *J* = 7.5 Hz, 3H). Data are in agreement with those reported in literature.[12]

***N*-benzyl-2-(3-isopropyl-1*H*-indol-1-yl)aniline (S-2c)**

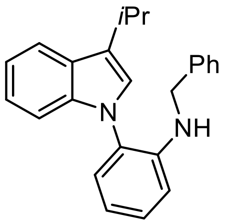

Synthetized from **S-1c** (250 mg, 1 mmol), benzaldehyde (122µL, 1.2 mmol), $MgSO_4$ (96 mg, 0.8 mmol), acetic acid (5.7 µL, 0.1 mmol), $NaBH(OAc)_3$ (422 mg, 2 mmol) and 1.7 mL of dichloroethane. Purification of the crude by flash column chromatography (SiO_2, Hex/DCM 98:2) yielded **S-2c** (210 mg, 62%) as a white solid.

^{1}H NMR (400 MHz, $CDCl_3$): 7.73 (m, 1H), 7.36 – 7.13 (m, 10H), 7.02 (d, *J* = 0.9 Hz, 1H), 6.87 – 6.78 (m, 2H), 5.64 – 3.39 (m, 2H), 3.30 (pd, *J* = 6.9, 1.0 Hz, 1H), 1.43 (dd, *J* = 7.1, 3.2 Hz, 6H). **^{13}C NMR** (101 MHz, $CDCl_3$): 143.99 (C), 138.78 (C), 137.23 (C), 129.13 (CH), 128.58 (CH), 128.51 (CH), 127.60 (C), 127.20 (CH), 127.06 (CH), 125.31 (C), 124.92 (C), 124.03 (CH), 122.17 (CH), 119.58 (CH), 119.46 (CH), 117.40 (CH), 112.25 (CH), 110.83 (CH), 47.86 (CH_2), 25.56 (CH_3), 23.35 (CH). **HRMS** (ESI) calculated for $C_{24}H_{25}N_2$ $[M+H]^+$ requires m/z = 341.4775, found m/z 341.4774.

***N*-benzyl-2-(3-phenyl-1*H*-indol-1-yl)aniline (S-2d)**

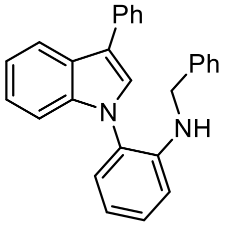

Synthetized from **S-1d** (463 mg, 1.60 mmol), benzaldehyde (200 µL, 2.0 mmol), $MgSO_4$ (157 mg, 1.3 mmol), acetic acid (9.3 µL, 0.163 mmol), $NaBH(OAc)_3$ (690 mg, 3.30 mmol) and 2.7 mL of dichloroethane. Purification of the crude by Biotage® Selekt (Hex/AcOEt 95:5 → 80:20), yielded **S-2d** (416, 68%) as a yellow sticky oil.

^{1}H NMR (300 MHz, $CDCl_3$): 8.03 (m, 1H), 7.76 – 7.71 (m, 2H), 7.53 – 7.46 (m, 2H), 7.49 (s, 1H), 7.38 – 7.19 (m, 11H) 6.86 –6.76 (m, 2H), 4.34 (s, 2H), 4.22 (bs,1H). Data are in agreement with those reported in literature.[12]

***N*-benzyl-2-(3-(naphthalen-2-yl)-1*H*-indol-1-yl)aniline (S-2e)**

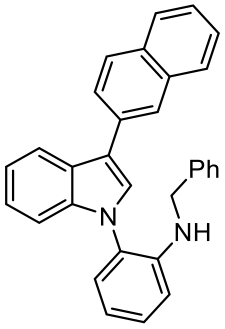

Synthetized from **S-1e** (334 mg, 1 mmol), benzaldehyde (122 µL, 1.2 mmol), $MgSO_4$ (96 mg, 0.8 mmol), acetic acid (5.7 µL, 0.1 mmol) $NaBH(OAc)_3$ (424 mg, 2 mmol) and 1.7 mL mL of dichloroethane. Purification of the crude by Biotage® Selekt (Hex/AcOEt 95:5 → 85:15 yielded **S-2e** (314 mg, 74%) as a white solid.

^{1}H NMR (300 MHz, $CDCl_3$): 8.20 (s, 1H), 8.16 (m, 1H), 7.98 – 7.84 (m, 4H), 7.55 (s, 1H), 7.54 – 7.44 (m, 2H), 7.36 – 7.20 (m, 10H), 6.90 – 6.80 (m, 2H), 4.49 (bs, 1H), 4.35 (s, 2H). **^{13}C NMR** (75 MHz, $CDCl_3$): 144.29 (C), 138.90 (C), 137.52 (C), 133.98 (C), 132.64 (C), 132.11 (C), 129.64 (CH), 128.61 (CH), 128.38 (CH), 127.80 (CH), 127.72 (CH), 127.17 (CH), 126.91 (CH), 126.75 (CH), 126.54 (C), 126.40 (CH), 126.21 (CH), 125.35 (CH), 125.31 (CH), 124.28 (C), 122.84 (CH), 120.93 (CH), 120.14 (CH), 118.85 (C), 117.07 (CH), 111.96 (CH), 111.30 (CH), 47.53 (CH_2). One CH is missing, probably overlapped. **HRMS** (ESI) calculated for $C_{31}H_{25}N_2$ $[M+H]^+$ requires m/z = 425.2013, found m/z 425.2014.

N-benzyl-2-(5-fluoro-3-methyl-1H-indol-1-yl)aniline (S-2f)

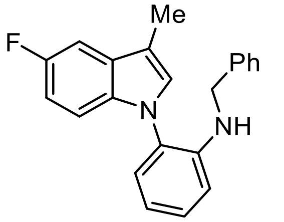

Synthetized from **S-1f** (320 mg, 1.3 mmol), benzaldehyde (163 µL, 1.6 mmol), $MgSO_4$ (128 mg, 1.06 mmol), acetic acid (7.6 µL, 0.13 mmol) $NaBH(OAc)_3$ (564 mg, 2.66 mmol) and 2.2 mL of dichloroethane. Purification of the crude by flash column chromatography (SiO_2, Hex/DCM 95:5 → 7:1) yielded **S-2f** (343 mg, 78%) as a white solid.

^{1}H NMR (400 MHz, $CDCl_3$): 7.38 – 7.23 (m, 7H), 7.19 (dd, *J* = 8.1, 1.6 Hz, 1H), 7.11 – 7.03 (m, 2H), 6.97 (td, *J* = 9.0, 2.5 Hz, 1H), 6.82 (ddd, *J* = 8.0, 6.6, 1.3 Hz, 2H), 4.34 (s, 2H), 4.17 (bs, 1H), 2.39 (d, *J* = 1.1 Hz, 3H). **^{13}C NMR** (101 MHz, $CDCl_3$): 158.10 (d, *J* = 235.2 Hz, C), 144.23 (C), 138.88 (C), 133.49 (C), 129.42 (C), 129.34 (CH), 128.63 (CH), 128.46 (CH), 127.90 (CH), 127.22 (CH), 126.98 (CH), 124.77 (C), 117.18 (CH), 112.57 (d, *J* = 4.7 Hz, C), 112.00 (CH), 111.43 (d, *J* = 9.5 Hz, CH), 110.47 (d, *J* = 26.2 Hz, CH), 104.00 (d, *J* = 23.3 Hz, CH), 47.65 (CH_2), 9.66 (CH_3). **HRMS** (ESI) calculated for $C_{22}H_{20}FN_2$ $[M+H]^+$ requires m/z = 331.1606, found m/z 331.1605.

N-benzyl-2-(5-methoxy-3-methyl-1H-indol-1-yl)aniline (S-2g)

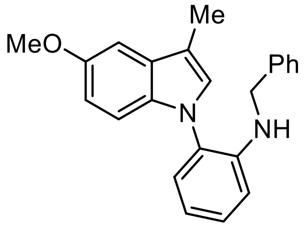

Synthetized from **S-1g** (391 mg, 1.55 mmol), benzaldehyde (190 µL, 1.86 mmol), $MgSO_4$ (149 mg, 1.2 mmol), acetic acid (9.5 µL, 0.16 mmol), $NaBH(OAc)_3$ (657 mg, 3.8 mmol) and 2.6 mL of dichloroethane. Purification of the crude by flash column chromatography (SiO_2, Hex/AcOEt 95:5) yielded **S-2g** (397 mg, 75%) as a pale-yellow solid.

^{1}H NMR (400 MHz, $CDCl_3$): 7.36 – 7.23 (m, 6H), 7.19 (dd, *J* = 7.6, 1.6 Hz, 1H), 7.10 (d, *J* = 2.4 Hz, 1H), 7.07 (dd, *J* = 8.8, 0.6 Hz, 1H), 7.01 (d, *J* = 1.2 Hz, 1H), 6.90 (dd, *J* = 8.8, 2.4 Hz, 1H), 6.81 – 6.77 (m, 2H), 4.34 (s, 2H), 4.22 (bs, 1H), 3.93 (s, 3H), 2.40 (d, *J* = 1.1 Hz, 3H). Data are in agreement with those reported in literature.[12]

N-benzyl-2-(6-bromo-3-methyl-1H-indol-1-yl)aniline (S-2h)

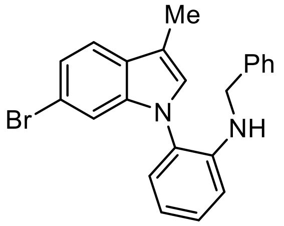

Synthetized from **S-1h** (300 mg, 1 mmol), benzaldehyde (122 µL, 1.2 mmol), $MgSO_4$ (96 mg, 0.8 mmol), acetic acid (5.7 µL, 0.1 mmol), $NaBH(OAc)_3$ (430 mg, 2 mmol) and 1.7 mL of dichloroethane. Purification of the crude by Biotage® Selekt (Hex/AcOEt 98:2 → 50:50) yielded **S-2h** (356 mg, 91%) as a pale-yellow solid.

^{1}H NMR (300 MHz, $CDCl_3$): 7.50 (dq, *J* = 8.3, 0.8 Hz, 1H), 7.39 – 7.21 (m, 8H), 7.15 (m, 1H), 6.99 (p, *J* = 1.0 Hz, 1H), 6.86 – 6.73 (m, 2H), 4.33 (d, *J* = 5.8 Hz, 2H), 4.06 (t, *J* = 5.9 Hz, 1H), 2.39 (d, *J* = 1.0 Hz, 3H). **^{13}C NMR** (75 MHz, $CDCl_3$): 144.18 (C), 138.89 (C), 137.58 (C), 129.51 (CH), 128.68 (CH), 128.41 (CH), 127.90 (C), 127.22 (CH), 127.04 (CH), 126.83 (CH), 124.15 (C), 122.85 (CH), 120.38 (CH), 117.05 (CH), 116.07 (C), 113.73 (CH), 112.80 (C), 111.83 (CH), 47.48 (CH_2), 9.60 (CH_3). **HRMS** (ESI) calculated for $C_{22}H_{20}BrN_2$ $[M+H]^+$ requires m/z = 391.0805, found m/z 391.0807.

N-benzyl-2-(7-bromo-3-methyl-1H-indol-1-yl)aniline (S-2i)

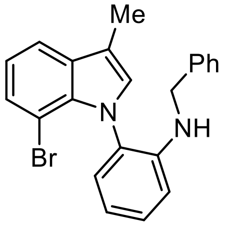

Synthetized from **S-1i** (1 g, 3.32 mmol), benzaldehyde (406 µL, 3.98 mmol), $MgSO_4$ (320 mg, 2.66 mmol), acetic acid (19 µL, 0.322 mmol), $NaBH(OAc)_3$ (1.41 g, 6.64 mmol) and 5.5 mL of dichloroethane. Purification of the crude by Biotage® Selekt (Hex/AcOEt 98:2 → 80:20) yielded **S-2i** (1.14 g, 88%) as a transparent sticky liquid.

^{1}H NMR (400 MHz, $CDCl_3$): 7.60 (m, 1H), 7.41 (d, *J* = 7.5 Hz, 1H), 7.36 – 7.21 (m, 6H), 7.18 (dd, *J* = 7.7, 1.6 Hz, 1H), 7.05 (t, *J* = 7.7 Hz, 1H), 6.97 – 6.92 (m, 1H), 6.77 (td, *J* = 7.5, 1.3 Hz, 1H), 6.69 (dd, *J* = 8.2, 1.3 Hz, 1H), 4.36 (d, *J* = 3.8 Hz, 2H), 3.97 (s, 1H), 2.39 (d, *J* = 1.1 Hz, 3H). **^{13}C NMR** (101 MHz, $CDCl_3$): 146.20 (C), 139.12 (C), 133.50 (C), 131.67 (C), 129.94 (CH), 129.79 (CH), 128.99 (CH), 128.55 (CH), 127.13 (CH), 127.06 (CH), 126.96 (CH), 125.39 (C), 120.69 (CH), 118.44 (CH), 116.24 (CH), 112.60 (C), 111.08 (CH), 104.31 (C), 47.60 (CH_2), 9.61 (CH_3). **HRMS** (ESI) calculated for $C_{22}H_{20}BrN_2$ $[M+H]^+$ requires m/z = 391.0805, found m/z 391.0803.

N-(4-methoxybenzyl)-2-(3-methyl-1H-indol-1-yl)aniline (S-2j)

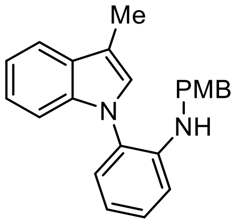

Synthetized from **S-1a** (300 mg, 1.34 mmol), 4-methoxybenzaldehyde (197 µL, 1.62 mmol), $MgSO_4$ (129 mg, 1.07 mmol), acetic acid (8 µL, 0.13 mmol), $NaBH(OAc)_3$ (569 mg, 2.68 mmol) and 2.2 mL of dichloroethane. Purification of the crude by Biotage® Selekt (Hex/AcOEt 9:1 → 8:2) yielded **S-2j** (434 mg, 95%) as a white solid.
^{1}H NMR (300 MHz, $CDCl_3$): 7.63 (m, 1H), 7.32 – 7.13 (m, 7H), 7.00 (s, 1H), 6.94 – 6.74 (m, 4H), 4.24 (m, 3H), 3.78 (s, 1H), 2.40 (m, 3H). **^{13}C NMR** (75 MHz, $CDCl_3$): 158.75 (C), 144.10 (C), 136.89 (C), 130.76 (C), 129.09 (CH), 129.01 (C), 128.47 (CH), 128.24 (CH), 126.21 (CH), 125.06 (C), 122.18 (CH), 119.54 (CH), 119.00 (CH), 117.14 (CH), 113.96 (CH), 112.59 (C), 112.07 (CH), 110.66 (CH), 55.25 (CH_3), 47.21 (CH_2), 9.67 (CH_3). **HRMS** (ESI) calculated for $C_{23}H_{23}N_2O$ $[M+H]^+$ requires m/z = 343.1805, found m/z 343.1803.

N-benzyl-3-methyl-2-(3-methyl-1H-indol-1-yl)aniline (S-2k)

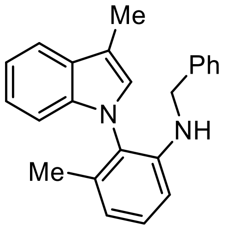

Synthetized from **S-1j** (300 mg, 1.28 mmol), benzaldehyde (156 µL, 1.53 mmol), $MgSO_4$ (123 mg, 1.02 mmol), acetic acid (7 µL, 0.13 mmol), $NaBH(OAc)_3$ (542 mg, 2.56 mmol) and 2 mL of dichloroethane. Purification of the crude by flash column chromatography (SiO_2, Hex/AcOEt 98:2) yielded **S-2k** (163 mg, 39%) as a white solid.
^{1}H NMR (300 MHz, $CDCl_3$): 7.68 (m, 1H), 7.34 – 7.12 (m, 8H), 7.02 (m, 1H), 6.91 (s, 1H), 6.67 (ddd, J = 7.6, 1.4, 0.7 Hz, 1H), 6.57 (d, J = 8.2 Hz, 1H), 4.27 (s, 2H), 4.07 (bs, 1H), 2.43 (d, J = 1.1 Hz, 3H), 1.88 (s, 3H). Data are in agreement with those reported in literature.[12]

N-benzyl-3-bromo-2-(3-methyl-1H-indol-1-yl)aniline (S-2l)

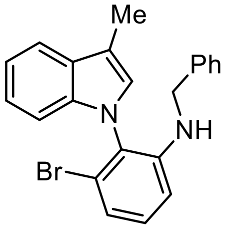

Synthetized from **S-1k** (1 g, 3.32 mmol), benzaldehyde (406 µL, 3.98 mmol), $MgSO_4$ (320 mg, 2.66 mmol), acetic acid (19 µL, 0.322 mmol), $NaBH(OAc)_3$ (1.41 g, 6.64 mmol) and 5.5 mL of dichloroethane. Purification of the crude by Biotage® Selekt (Hex/AcOEt 98:2 → 88:12) yielded **S-2l** (850 mg, 65%) as a transparent sticky liquid.
^{1}H NMR (300 MHz, $CDCl_3$): 7.66 (m, 1H), 7.34 – 7.13 (m, 7H), 7.13 – 6.98 (m, 3H), 6.90 (s, 1H), 6.62 (d, *J* = 8.1 Hz, 1H), 4.26 (d, *J* = 6.0 Hz, 2H), 4.14 (d, *J* = 6.1 Hz, 1H), 2.42 (s, 3H). **^{13}C NMR** (75 MHz, $CDCl_3$): 147.03 (C), 138.50 (C), 136.10 (C), 130.44 (CH), 128.92 (C), 128.61 (CH), 127.17 (CH), 126.67 (CH), 125.35 (CH), 124.58 (C), 123.21 (C), 122.45 (CH), 120.59 (CH), 119.83 (CH), 119.10 (CH), 113.16 (C), 110.46 (CH), 110.23 (CH), 47.21 (CH_2), 9.83 (CH_3). **HRMS** (ESI) calculated for $C_{22}H_{20}BrN_2$ $[M+H]^+$ requires m/z = 391.0805, found m/z 391.0805.

N-benzyl-4-methyl-2-(3-methyl-1H-indol-1-yl)aniline (S-2m)

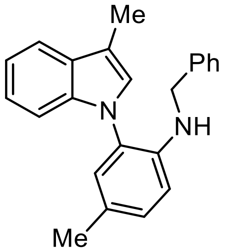

Synthetized from **S-1l** (201 mg, 0.85 mmol), benzaldehyde (104 µL, 1.02 mmol), $MgSO_4$ (82 mg, 0.68 mmol), acetic acid (4.9 µL, 0.09 mmol), $NaBH(OAc)_3$ (360 mg, 1.7 mmol) and 1.4 mL of dichloroethane. Purification of the crude by Biotage® Selekt (Hex/AcOEt 90:10 → 75:25) yielded **S-2m** (235 mg, 85%) as an orange sticky oil.
^{1}H NMR (300 MHz, $CDCl_3$): 7.63 (m, 1H), 7.34 – 7.17 (m, 7H), 7.13 (m,1H), 7.04 (ddd, J = 8.2, 2.1, 0.8 Hz, 1H), 7.02 – 6.97 (m, 2H), 6.67 (d, J = 8.3 Hz, 1H), 4.28 (s, 2H), 2.39 (d, J = 1.1 Hz, 3H), 2.27 (s, 3H). Data are in agreement with those reported in literature.[12]

***N*-benzyl-*N*-(2-(3-methyl-1*H*-indol-1-yl)phenyl)-3-phenylpropiolamide (1aa)**

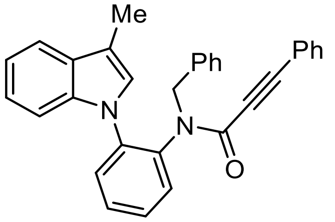

Synthetized from **S-2a** (250 mg, 0.8 mmol), DMAP (4.5 mg, 0.04 mmol), Et_3N (223 µL, 1.6 mmol), 3-phenylpropioloyl chloride (198 mg, 1.2 mmol) and DCM (8 mL). Purification by flash column chromatography (SiO_2, Hex/AcOEt 9:1 → 8:1) yielded **1aa** (352 mg, 87%) as a yellow solid.

^{1}H NMR (400 MHz, DMSO, 353 K): 7.66 – 7.53 (m, 3H), 7.51 – 7.31 (m, 5H), 7.28 – 7.18 (m, 5H), 7.18 – 7.09 (m, 4H), 7.05 (m, 2H), 5.05 (d, *J* = 14.7 Hz, 1H), 3.54 (d, *J* = 14.7 Hz, 1H), 2.36 (d, *J* = 1.2 Hz, 3H). **^{13}C NMR** (101 MHz, DMSO, 363 K): 153.99 (C), 137.45 (C), 137.34 (C), 136.90 (C), 136.47 (C), 132.72 (CH), 132.44 (CH), 131.01 (CH), 130.17 (CH), 129.41 (C), 129.30 (CH), 129.17 (CH), 128.84 (CH), 128.74 (CH), 128.41 (CH), 127.90 (CH), 126.44 (CH), 122.80 (CH), 120.24 (CH), 119.91 (C), 119.44 (CH), 113.01 (C), 110.47 (CH), 91.45 (C), 83.39 (C), 50.56 (CH_2), 9.66 (CH_3). **HRMS** (ESI) calculated for $C_{31}H_{24}N_2ONa$ $[M+Na]^+$ requires m/z = 463.1786, found m/z 463.1791.

***N*-benzyl-*N*-(2-(3-methyl-1*H*-indol-1-yl)phenyl)-3-(*p*-tolyl)propiolamide (1ab)**

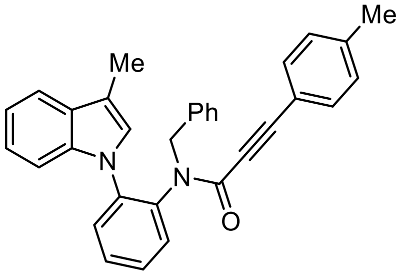

Synthetized from **S-2a** (200 mg, 0.64 mmol), DMAP (3.9 mg, 0.03 mmol), Et_3N (178 µL, 1.28 mmol), 3-(*p*-tolyl)propioloyl chloride (172 mg, 0.96 mmol) and 6.4 mL of DCM. Purification by flash column chromatography (SiO_2, Hex/AcOEt 8:1) yielded **1ab** (216 mg, 74%) as an orange solid.

^{1}H NMR (400 MHz, DMSO, 353 K): 7.66 – 7.54 (m, 2H), 7.53 – 7.38 (m, 2H), 7.33 (d, *J* = 7.8 Hz, 1H), 7.26 – 7.02 (m, 13H), 5.06 (d, *J* = 14.7 Hz, 1H), 3.53 (d, *J* = 14.7 Hz, 1H), 2.35 (s, 3H), 2.32 (s, 3H). **^{13}C NMR** (101 MHz, DMSO, 353 K): 154.10 (C), 141.36 (C), 137.46 (C), 137.30 (C), 136.84 (C), 136.51 (C), 132.74 (CH), 132.41 (CH), 130.14 (CH), 129.96 (CH), 129.35 (C), 129.15 (CH), 128.81 (CH), 128.74 (CH), 128.41 (CH), 127.89 (CH), 126.43 (CH), 122.80 (CH), 120.24 (CH), 119.45 (CH), 116.83 (C), 112.97 (C), 110.46 (CH), 91.91 (C), 83.05 (C), 50.44 (CH_2), 21.51 (CH_3), 9.71 (CH_3). Because of the presence of conformers with high energy barriers, ^{13}C-NMR analysis shows the signals of both. **HRMS** (ESI) calculated for $C_{32}H_{26}N_2ONa$ $[M+Na]^+$ requires m/z = 477.1943, found m/z 477.1942.

***N*-benzyl-3-(4-methoxyphenyl)-*N*-(2-(3-methyl-1*H*-indol-1-yl)phenyl)propiolamide (1ac)**

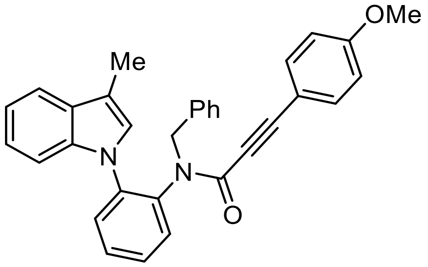

Synthetized from **S-2a** (200 mg, 0.64 mmol), DMAP (3.9 mg, 0.03 mmol), Et_3N (178 µL, 1.28 mmol), 3-(4-methoxyphenyl)propioloyl chloride (187 mg, 0.96 mmol) and 6.4 mL of DCM. Purification by flash column chromatography (SiO_2, Hex/AcOEt 5:1) yielded **1ac** (224 mg, 74%) as a pink solid.

^{1}H NMR (400 MHz, DMSO, 363 K): 7.64 – 7.53 (m, 2H), 7.52 – 7.30 (m, 3H), 7.25 – 7.02 (m, 11H), 6.97 – 6.90 (m, 2H), 5.05 (d, *J* = 14.8 Hz, 1H), 3.80 (s, 3H), 3.53 (d, *J* = 14.8 Hz, 1H), 2.35 (d, *J* = 1.1 Hz, 2H). **^{13}C NMR** (101 MHz, DMSO, 353 K): 161.70 (C), 154.25 (C), 137.55 (C), 137.30 (C), 136.86 (C), 136.58 (C), 134.37 (CH), 132.80 (CH), 130.09 (CH), 129.35 (C), 129.12 (CH), 128.73 (CH), 128.37 (CH), 127.86 (CH), 126.43 (CH), 122.79 (CH), 120.23 (CH), 119.44 (CH), 115.25 (CH), 114.59 (CH), 112.94 (C), 111.60 (C), 110.47 (CH), 92.26 (C), 82.72 (C), 55.96 (CH_3), 50.37 (CH_2), 9.70 (CH_3). Because of the presence of conformers with high energy barriers, ^{13}C-NMR analysis shows the signals of both. **HRMS** (ESI) calculated for $C_{32}H_{26}N_2O_2Na$ $[M+Na]^+$ requires m/z = 493.1892, found m/z 493.1892.

N-benzyl-3-(4-fluorophenyl)-N-(2-(3-methyl-1H-indol-1-yl)phenyl)propiolamide (1ad)

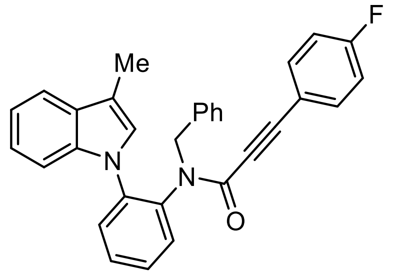

Synthetized from **S-2a** (200 mg, 0.64 mmol), DMAP (4 mg, 0.03 mmol), Et_3N (178 µL, 1.28 mmol), 3-(4-fluorophenyl)propioloyl chloride (175 mg, 0.96 mmol) and DCM (6.4 mL). Purification by flash column chromatography (SiO_2, Hex/AcOEt 9:1) yielded **1ad** (173 mg, 58%) as a yellow solid.

^{1}H NMR (500 MHz, DMSO, 363 K): 7.64 – 7.54 (m, 3H), 7.46 (t, *J* = 7.7 Hz, 1H), 7.34 (d, *J* = 7.9 Hz, 1H), 7.31 – 7.18 (m, 7H), 7.18 – 7.09 (m, 4H), 7.08 – 7.03 (m, 2H), 5.06 (d, *J* = 14.7 Hz, 1H), 3.56 (d, *J* = 14.7 Hz, 1H), 2.36 (d, *J* = 1.1 Hz, 3H). **^{13}C NMR** (126 MHz, DMSO, 363 K): 163.66 (d, *J* = 251.1 Hz, C), 153.88 (C), 137.33 (d, *J*= 6.0, C), 136.86 (C), 136.44 (C), 135.05 (d, *J* = 8.9 Hz, CH), 134.98 (CH), 132.68 (CH), 130.21 (CH), 129.38 (C), 129.16 (CH), 128.83 (CH), 128.75 (CH), 128.42 (CH), 127.91 (CH), 126.43 (CH), 122.81 (CH), 120.25 (CH), 119.46 (CH), 116.73 (d, *J* = 22.5 Hz, CH), 116.32 (C), 113.01 (C), 110.44 (CH), 90.42 (C), 83.19 (C), 50.54 (CH_2), 9.66 (CH_3). One C is missing, probably overlapped. **HRMS** (ESI) calculated for $C_{31}H_{23}N_2OFNa$ $[M+Na]^+$ requires m/z = 481.1692, found m/z 481.1693.

N-benzyl-3-(4-bromophenyl)-N-(2-(3-methyl-1H-indol-1-yl)phenyl)propiolamide (1ae)

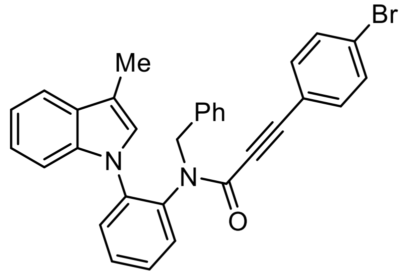

Synthetized from **S-2a** (300 mg, 0.96 mmol), DMAP (6 mg, 0.05 mmol), Et_3N (268 µL, 1.92 mmol), 3-(4-bromophenyl)propioloyl chloride (351 mg, 1.44 mmol) and 9.6 mL of DCM. Purification by flash column chromatography (SiO_2, Hex/AcOEt 9:1) yielded **1ae** (317 mg, 64%) as a yellow solid.

^{1}H NMR (500 MHz, DMSO, 363 K): 7.65 – 7.54 (m, 5H), 7.45 (t, *J* = 7.45, 1H), 7.34 (d, *J* = 8.0 Hz, 1H), 7.29 – 7.02 (m, 11H), 5.06 (d, *J* = 14.7 Hz, 1H), 3.57 (d, *J* = 14.7 Hz, 1H), 2.36 (s, 3H). **^{13}C NMR** (126 MHz, DMSO, 363 K): 153.77 (C), 137.29 (C), 136.86 (C), 136.39 (C), 134.17 (CH), 132.63 (CH), 132.50 (CH), 130.25 (CH), 129.39 (C), 129.18 (CH), 128.85 (CH), 128.75 (CH), 128.43 (CH), 127.93 (CH), 126.44 (CH), 124.80 (C), 122.83 (CH), 120.26 (CH), 119.46 (CH), 119.10 (C), 113.03 (C), 110.44 (CH), 90.20 (C), 84.26 (C), 50.61 (CH_2), 9.66 (CH_3). One C is missing, probably overlapped. **HRMS** (ESI) calculated for $C_{31}H_{23}N_2ONaBr$ $[M+Na]^+$ requires m/z = 541.0891, found m/z 541.0892.

N-benzyl-N-(2-(3-methyl-1H-indol-1-yl)phenyl)-3-(4-(trifluoromethyl)phenyl)propiolamide (1af)

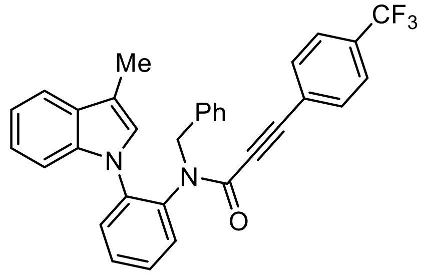

Synthetized from **S-2a** (300 mg, 0.96 mmol), DMAP (5.9 mg, 0.05 mmol), Et_3N (279 µL, 1.94 mmol), 3-(4-(trifluoromethyl)phenyl)propioloyl chloride (335 mg, 1.44 mmol) and 9.5 mL of DCM. Purification by flash column chromatography (SiO_2, Hex/AcOEt 8:1) yielded **1af** (337 mg, 69%) as an orange solid.

^{1}H NMR (500 MHz, DMSO, 363 K): 7.74 (t, *J* = 8.2 Hz, 2H), 7.66 – 7.55 (m, 3H), 7.53 – 7.35 (m, 3H), 7.28 – 7.04 (m, 10H), 5.08 (d, *J* = 14.6 Hz, 1H), 3.61 (d, *J* = 14.6 Hz, 1H), 2.37 (s, 3H). **^{13}C NMR** (126 MHz, DMSO, 363 K): 153.54 (C), 137.32 (C), 137.16 (C), 136.88 (C), 136.31 (C), 133.13 (CH), 132.59 (CH), 131.01 (q, *J* = 31.8 Hz, C), 130.33 (CH), 129.42 (C), 129.21 (CH), 128.88 (CH), 128.76 (CH), 128.46 (CH), 127.96 (CH), 126.43 (CH), 126.13 (q, *J* = 3.9 Hz, CH), 124.14 (C), 124.13 (q, *J* = 272.5 Hz, C), 122.84 (CH), 120.29 (CH), 119.48 (CH), 113.11 (C), 110.43 (CH), 89.36 (C) , 85.03 (C), 50.71 (CH_2), 9.62 (CH_3). **HRMS** (ESI) calculated for $C_{32}H_{23}N_2OF_3Na$ $[M+Na]^+$ requires m/z = 531.1660, found m/z 531.1661.

ethyl 4-(3-(benzyl(2-(3-methyl-1*H*-indol-1-yl)phenyl)amino)-3-oxoprop-1-yn-1-yl)benzoate (1ag)

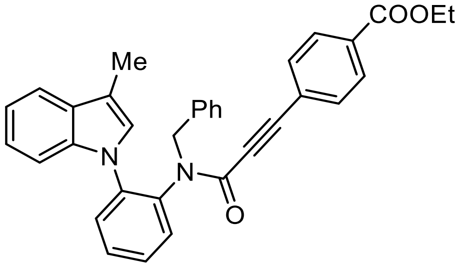

Synthetized from **S-2a** (300 mg, 0.96 mmol), DMAP (5.9 mg, 0.05 mmol), Et_3N (270 µL), ethyl 4-(3-chloro-3-oxoprop-1-yn-1-yl)benzoate (1.36 g, 5.76 mmol) and DCM (9.5 mL). Purification by flash column chromatography (SiO_2, Hex/AcOEt 9:1) yielded **1ag** (257 mg, 52%) as a yellow solid.

^{1}H NMR (500 MHz, DMSO, 363 K): 7.94 (d, *J* = 8.0 Hz, 2H), 7.68 – 7.54 (m, 3H), 7.47 (t, *J* = 7.4 Hz, 1H), 7.35 (t, *J* = 6.8 Hz, 2H), 7.24 (d, *J* = 6.5 Hz, 3H), 7.19 – 7.04 (m, 7H), 5.07 (d, *J* = 14.6 Hz, 1H), 4.35 (q, *J* = 7.1 Hz, 2H), 3.60 (d, *J* = 14.6 Hz, 2H), 2.36 (s, 3H), 1.34 (t, *J* = 7.1 Hz, 3H). **^{13}C NMR** (126 MHz, DMSO, 363 K): 165.35 (C), 153.62 (C), 137.30 (C), 137.20 (C), 136.86 (C), 136.34 (C), 132.61 (CH), 132.07 (C), 130.33 (CH), 129.80 (CH), 129.39 (C), 129.21 (CH), 128.87 (CH), 128.77 (CH), 128.49 (CH), 127.96 (CH), 126.45 (CH), 124.29 (C), 122.84 (CH), 120.27 (CH), 119.47 (CH), 113.05 (C), 110.44 (CH), 90.06 (C), 85.33 (C), 61.53 (CH_2), 50.68 (CH_2), 14.45 (CH_3), 9.65 (CH_3). One CH is missing, probably overlapped. **HRMS** (ESI) calculated for $C_{34}H_{28}N_2O_3Na$ $[M+Na]^+$ requires m/z = 535.1998, found m/z 535.2003.

***N*-benzyl-*N*-(2-(3-methyl-1*H*-indol-1-yl)phenyl)-3-(*m*-tolyl)propiolamide (1ah)**

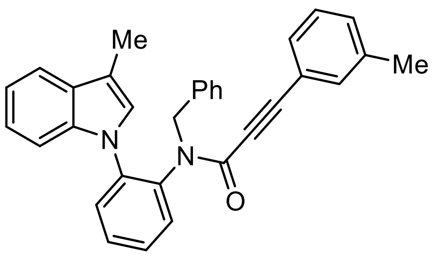

Synthetized from **S-2a** (200 mg, 0.64 mmol), DMAP (4 mg, 0.03 mmol), Et_3N (178 µL, 1.28 mmol), 3-(4-fluorophenyl)propioloyl chloride (172 mg, 0.96 mmol) and DCM (6.4 mL). Purification by flash column chromatography (SiO_2, Hex/AcOEt 9:1 → 8:1) yielded **1ah** (226 mg, 78%) as a pale-yellow solid.

^{1}H NMR (400 MHz, DMSO, 353 K): 7.67 – 7.56 (m, 2H), 7.46 (t, *J* = 7.4 Hz, 1H), 7.34 (d, *J* = 8.2 Hz, 1H), 7.30 – 7.08 (m, 10H), 7.08 – 6.97 (m, 4H), 5.06 (d, *J* = 14.7 Hz, 1H), 3.54 (d, *J* = 14.7 Hz, 1H), 2.36 (s, 3H), 2.27 (s, 3H). **^{13}C NMR** (101 MHz, DMSO, 353 K): 154.02 (C), 138.84 (C), 137.40 (C), 137.28 (C), 136.83 (C), 136.47 (C), 132.81 (CH), 132.73 (CH), 131.81 (CH), 130.16 (CH), 129.55 (CH), 129.36 (C), 129.17 (CH), 128.83 (CH), 128.75 (CH), 128.43 (CH), 127.90 (CH), 126.42 (CH), 122.82 (CH), 120.25 (CH), 119.73 (C), 119.46 (CH), 112.99 (C), 110.46 (CH), 91.74 (C), 83.13 (C), 50.47 (CH_2), 21.01 (CH_3), 9.70 (CH_3). One CH is missing, probably overlapped. Because of the presence of conformers with high energy barriers, ^{13}C-NMR analysis shows the signals of both. **HRMS** (ESI) calculated for $C_{32}H_{26}N_2ONa$ $[M+Na]^+$ requires m/z = 477.1943, found m/z 477.1944.

***N*-benzyl-*N*-(2-(3-methyl-1*H*-indol-1-yl)phenyl)-3-(*o*-tolyl)propiolamide (1ai)**

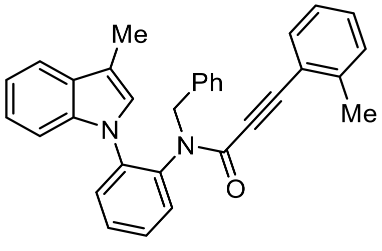

Synthetized from **S-2a** (300 mg, 0.96 mmol), DMAP (6 mg, 0.05 mmol), Et_3N (268 µL, 1.92 mmol), 3-(4-bromophenyl)propioloyl chloride (257 mg, 1.44 mmol) and 9.6 mL of DCM. Purification by flash column chromatography (SiO_2, Hex/AcOEt 9:1 → 5:1) yielded **1ai** (367 mg, 84%) as a yellow solid.

^{1}H NMR (500 MHz, DMSO, 363 K): 7.63 (dt, *J* = 7.8, 1.0 Hz, 1H), 7.59 – 7.53 (m, 2H), 7.42 (d, *J* = 6.1 Hz, 1H), 7.39 – 7.31 (m, 2H), 7.29 – 7.17 (m, 7H), 7.17 – 7.09 (m, 3H), 7.06 – 7.02 (m, 2H), 5.05 (d, *J* = 14.7 Hz, 1H), 3.50 (d, *J* = 14.7 Hz, 1H), 2.36 (s, 3H), 2.02 (s, 3H). **^{13}C NMR** (126 MHz, DMSO, 363 K): 154.12 (C), 141.31 (C), 137.33 (C), 137.26 (C), 136.80 (C), 136.42 (C), 133.17 (CH), 132.87 (CH), 130.97 (CH), 130.22 (CH), 130.17 (CH), 129.41 (C), 129.14 (CH), 128.84 (CH), 128.71 (CH), 128.53 (CH), 127.88 (CH), 126.47 (CH), 126.39 (CH), 122.82 (CH), 120.27 (CH), 119.84 (C), 119.46 (CH), 113.07 (C), 110.43 (CH), 90.38 (C), 87.10 (C), 50.49 (CH_2), 19.97 (CH_3), 9.66 (CH_3). **HRMS** (ESI) calculated for $C_{32}H_{26}N_2ONa$ $[M+Na]^+$ requires m/z = 477.1943, found m/z 477.1944.

***N*-benzyl-*N*-(2-(3-methyl-1*H*-indol-1-yl)phenyl)-3-(naphthalen-2-yl)propiolamide (1aj)**

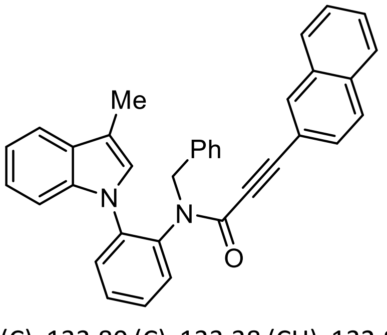

Synthetized from **S-2a** (188 mg, 0.64 mmol), DMAP (3.9 mg, 0.03 mmol), Et_3N (178 µL, 1.28 mmol), 3-(naphthalen-2-yl)propioloyl chloride (550 mg, 2,56 mmol) and 6.4 mL of DCM. Purification by flash column chromatography (SiO_2, Hex/AcOEt 8:1) yielded **1aj** (160 mg, 51%) as a yellow solid.

^{1}H NMR (500 MHz, DMSO, 363 K): 7.90 (dt, *J* = 13.3, 6.7 Hz, 4H), 7.70 – 7.53 (m, 5H), 7.53 – 7.46 (m, 1H), 7.40 (d, *J* = 8.1 Hz, 1H), 7.33 – 6.94 (m, 10H), 5.10 (d, *J* = 14.7 Hz, 1H), 3.60 (d, *J* = 14.7 Hz, 1H), 2.37 (s, 3H). **^{13}C NMR** (126 MHz, DMSO, 363 K): 154.04 (C), 137.48 (C), 137.35 (C), 136.94 (C), 136.51 (C), 133.80 (C), 133.38 (CH), 132.80 (C), 132.76 (CH), 130.21 (CH), 129.42 (C), 129.20 (CH), 129.00 (CH), 128.86 (CH), 128.76 (CH), 128.47 (CH), 128.43 (CH), 128.40 (CH), 128.21 (CH), 127.92 (CH), 127.90 (CH), 127.58 (CH), 126.47 (CH), 122.83 (CH), 120.26 (CH), 119.46 (CH), 117.16 (C), 113.04 (C), 110.51 (CH), 91.92 (C), 83.71 (C), 50.61 (CH_2), 9.68 (CH_3). Because of the presence of conformers with high energy barriers, ^{13}C-NMR analysis shows the signals of both. **HRMS** (ESI) calculated for $C_{35}H_{26}N_2ONa$ $[M+Na]^+$ requires m/z = 513.1943, found m/z 513.1947.

***N*-benzyl-*N*-(2-(3-methyl-1*H*-indol-1-yl)phenyl)-3-(thiophen-2-yl)propiolamide (1ak)**

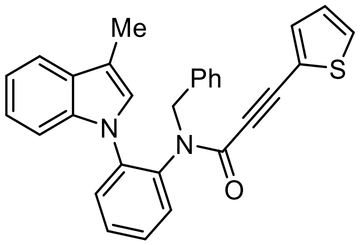

Synthetized from **S-2a** (300 mg, 0.96 mmol), DMAP (5.9 mg, 0.05 mmol), Et_3N (270 µL, 1.94 mmol), 3-(thiophen-2-yl)propioloyl chloride (655 mg, 3.84 mmol) and 9.6 mL of DCM. Purification by flash column chromatography (SiO_2, Hex/AcOEt 8:1) yielded **1ak** (198 mg, 56%) as a yellow solid.

^{1}H NMR (400 MHz, DMSO, 363 K): 7.75 – 7.50 (m, 4H), 7.42 (m, 1H), 7.31 (d, *J* = 7.1 Hz, 1H), 7.27 – 7.17 (m, 4H), 7.17 – 7.01 (m, 7H), 5.06 (d, *J* = 14.7 Hz, 1H), 3.60 (d, *J* = 14.7 Hz, 1H), 2.35 (dd, *J* = 3.0, 1.1 Hz, 3H). **^{13}C NMR** (101 MHz, DMSO, 363 K): 153.86 (C), 137.19 (C), 137.07 (C), 136.36 (C), 136.11 (C), 132.56 (C), 131.54 (C), 130.22 (C), 128.71 (CH), 128.60 (CH), 128.52 (CH), 127.80 (CH), 126.59 (CH), 122.84 (CH), 122.79 (CH), 120.23 (CH), 120.27 (CH), 119.42 (CH), 110.46 (CH), 110.25 (CH), 87.38 (C), 85.36 (C), 50.61 (CH_2), 9.64 (CH_3). Because of the presence of conformers with high energy barriers, ^{13}C-NMR analysis shows the signals of both. **HRMS** (ESI) calculated for $C_{29}H_{22}N_2ONaS$ $[M+Na]^+$ requires m/z = 469.1351, found m/z 469.1342.

***N*-benzyl-*N*-(2-(3-ethyl-1*H*-indol-1-yl)phenyl)-3-phenylpropiolamide (1an)**

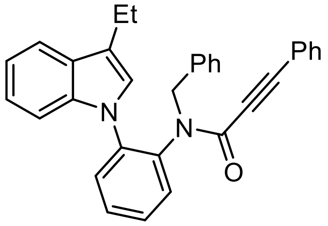

Synthetized from **S-2b** (588 mg, 1.8 mmol), DMAP (10 mg, 0.09 mmol), Et_3N (502 µL, 3.6 mmol), 3-phenylpropioloyl chloride (444 mg, 2.7 mmol) and 18 mL of DCM. Purification by flash column chromatography (SiO_2, Hex/AcOEt 7:1) yielded **1an** (437 mg, 53%) as a pale-yellow solid.

^{1}H NMR (400 MHz, DMSO, 353 K): 7.65 (dd, *J* = 6.5, 2.4 Hz, 1H), 7.58 (m, 1H), 7.56 – 7.32 (m, 6H), 7.29 – 7.08 (m, 9H), 7.07 – 7.00 (m, 2H), 5.05 (d, *J* = 14.7 Hz, 1H), 3.50 (d, *J* = 14.7 Hz, 1H), 2.81 (q, *J* = 7.5 Hz, 2H), 1.33 (t, *J* = 7.4 Hz, 3H). **^{13}C NMR** (101 MHz, DMSO, 353 K): 153.96 (C), 137.39 (C), 136.85 (C), 136.42 (C), 132.72 (CH), 132.43 (CH), 131.04 (CH), 130.20 (CH), 129.32 (CH), 129.10 (CH), 128.79 (CH), 128.75 (CH), 128.45 (C), 128.42 (CH), 127.89 (CH), 125.42 (CH), 122.84 (CH), 120.26 (CH), 120.16 (C), 119.86 (C), 119.53 (CH), 110.51 (CH), 91.42 (C), 83.32 (C), 50.44 (CH_2), 18.11 (CH_2), 14.80 (CH_3). One C is missing, probably overlapped. **HRMS** (ESI) calculated for $C_{32}H_{26}N_2ONa$ $[M+Na]^+$ requires m/z = 477.1943, found m/z 477.1944.

N-benzyl-N-(2-(3-isopropyl-1H-indol-1-yl)phenyl)-3-phenylpropiolamide (1ao)

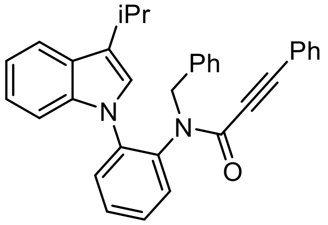

Synthetized from **S-2c** (210 mg, 0.62 mmol), DMAP (4 mg, 0.03 mmol), Et_3N (173 µL, 1.24 mmol), 3-phenylpropioloyl chloride (153 mg, 0.93 mmol) and DCM (6.2 mL). Purification by flash column chromatography (SiO_2 Hex/AcOEt 9:1 → 5:1) yielded **1ao** (223 mg, 50%) as a yellow solid.

^{1}H NMR (600 MHz, DMSO, 353 K): 7.70 (dd, *J* = 6.9, 2.2 Hz, 1H), 7.65 – 7.48 (m, 3H), 7.48 – 7.34 (m, 4H), 7.26 – 7.09 (m, 9H), 7.05 – 7.01 (m, 2H), 5.04 (d, *J* = 14.7 Hz, 1H), 3.44 (d, *J* = 14.7 Hz, 1H), 3.25 (p, *J* = 6.9 Hz, 1H), 1.38 (d, *J* = 7.1 Hz, 6H). **^{13}C NMR** (151 MHz, DMSO, 353 K): 153.97 (C), 137.55 (C), 137.42 (C), 136.86 (C), 136.41 (C), 132.75 (CH), 132.42 (CH), 131.01 (CH), 130.18 (CH), 129.30 (CH), 129.03 (CH), 128.75 (CH), 128.72 (CH), 128.40 (CH), 127.86 (CH), 127.87 (C) 125.40 (C), 124.26 (CH), 122.78 (CH), 120.25 (CH), 119.93 (CH), 119.88 (C), 110.57 (CH), 91.41 (C), 83.33 (C), 50.40 (CH_2), 25.34 (CH), 23.64 (CH_3). **HRMS** (ESI) calculated for $C_{33}H_{28}N_2ONa$ $[M+Na]^+$ requires m/z = 491.2099, found m/z 491.2103.

N-benzyl-3-phenyl-N-(2-(3-phenyl-1H-indol-1-yl)phenyl)propiolamide (1ap)

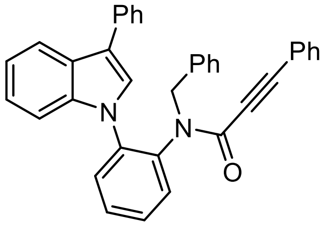

Synthetized from **S-2d** (412 mg, 1.1 mmol), DMAP (6 mg, 0.06 mmol), Et_3N (307 µL, 2.2 mmol), 3-phenylpropioloyl chloride (272 mg, 1.65 mmol) and DCM (11 mL). Purification by flash column chromatography (SiO_2, Hex/AcOEt 8:1 → 7:1 + 5% of DCM → 5:1 + 5% of DCM) yielded **1ap** (367 mg, 66%) as a pale-yellow solid.

^{1}H NMR (400 MHz, DMSO, 353 K): 7.96 (d, *J* = 7.7 Hz, 1H), 7.75 – 7.59 (m, 4H), 7.57 – 7.30 (m, 9H), 7.30 – 7.14 (m, 8H), 7.08 (d, *J* = 6.8 Hz, 2H), 5.09 (d, *J* = 14.7 Hz, 1H), 3.71 (d, *J* = 14.7 Hz, 1H). **^{13}C NMR** (101 MHz, DMSO, 353 K): 153.99 (C), 137.96 (C), 137.68 (C), 136.41 (C), 134.98 (C), 132.70 (CH), 132.47 (CH), 131.11 (CH), 130.40 (CH), 129.34 (CH), 129.09 (CH), 128.82 (CH), 128.78 (CH), 127.95 (CH), 127.57 (CH), 126.96 (CH), 126.69 (CH), 126.48 (C), 123.31 (CH), 121.52 (CH), 120.19 (CH), 119.78 (C), 119.05 (C), 111.15 (CH), 91.62 (C), 83.28 (C), 50.83 (CH_2). One C and two CH are missing, probably overlapped. **HRMS** (ESI) calculated for $C_{36}H_{26}N_2ONa$ $[M+Na]^+$ requires m/z = 525.1943, found m/z 525.1945.

N-benzyl-N-(2-(3-(naphthalen-2-yl)-1H-indol-1-yl)phenyl)-3-phenylpropiolamide (1aq)

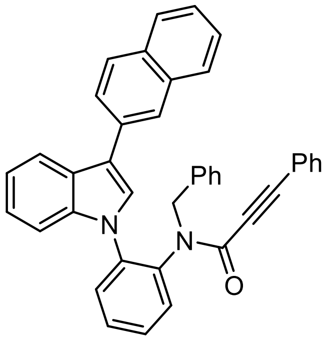

Synthetized from **S-2e** (296 mg, 0.70 mmol), DMAP (3.9 mg, 0.04 mmol), Et_3N (194 µL, 1.39 mmol), 3-phenylpropioloyl chloride (172 mg, 1.05 mmol) and DCM (7 mL). Purification by flash column chromatography (SiO_2, Hex/AcOEt 7:1) yielded **1aq** (299 mg, 78%) as a yellow solid.

^{1}H NMR (500 MHz, DMSO, 363 K): 8.26 (d, *J* = 1.8 Hz, 1H), 8.13 (d, *J* = 8.0 Hz, 1H), 8.04 – 7.85 (m, 4H), 7.79 – 7.65 (m, 3H), 7.60 – 7.46 (m, 5H), 7.40 (m, 3H), 7.35 – 7.17 (m, 7H), 7.16 – 7.08 (m, 2H), 5.12 (d, *J* = 14.7 Hz, 1H), 3.79 (d, *J* = 14.7 Hz, 1H). **^{13}C NMR** (126 MHz, DMSO, 363 K): 154.00 (C), 138.13 (C), 137.79 (C), 136.46 (C), 134.17 (C), 132.69 (CH), 132.56 (C), 132.48 (CH), 132.23 (C), 131.10 (CH), 130.41 (CH), 129.50 (CH), 129.33 (CH), 129.17 (CH), 128.85 (CH), 128.81 (CH), 128.79 (CH), 128.21 (CH), 128.21 (CH), 127.98 (CH), 127.95 (CH), 126.72 (CH), 126.59 (C), 126.50 (CH), 125.90 (CH), 125.24 (CH), 123.42 (CH), 121.67 (CH), 120.44 (CH), 119.82 (C), 118.88 (C), 111.25 (CH), 91.63 (C), 83.36 (C), 50.94 (CH_2). One C is missing, probably overlapped. **HRMS** (ESI) calculated for $C_{40}H_{38}N_2ONa$ $[M+Na]^+$ requires m/z = 575.2099, found m/z 575.2111.

N-benzyl-N-(2-(5-fluoro-3-methyl-1H-indol-1-yl)phenyl)-3-phenylpropiolamide (1ar)

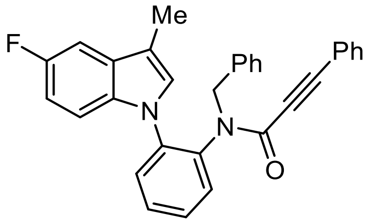

Synthetized from **S-2f** (323 mg, 0.98 mmol), DMAP (6 mg, 0.05 mmol), Et_3N (273 mg, 1.96 mmol), 3-phenylpropioloyl chloride (241 mg, 1.47 mmol) and DCM (9.8 mL). Purification by Biotage® Selekt (Hex/AcOEt 98:2 → 80:20) yielded **1ar** (285 mg, 64%) as a yellow solid.

^{1}H NMR (400 MHz, DMSO, 363 K): 7.63 – 7.31 (m, 8H), 7.28 – 6.99 (m, 9H), 6.91 (t, J = 9.2 Hz, 1H), 5.03 (d, J = 14.7 Hz, 1H), 3.64 (d, J = 14.8 Hz, 1H), 2.32 (d, J = 1.1 Hz, 3H). **^{13}C NMR** (101 MHz, DMSO, 363 K): 158.05 (d, J = 233.9 Hz, C), 153.98 (C), 137.52 (C), 136.70 (C), 136.41 (C), 134.54 (C), 133.99 (C), 132.70 (CH), 132.45 (CH), 131.05 (CH), 130.27 (CH), 129.31 (CH), 129.12 (CH), 128.81 (CH), 128.72 (CH), 128.67 (CH), 128.40 (CH), 127.90 (CH), 119.84 (C), 113.03 (d, J = 9.6 Hz, C), 111.60 (d, J = 9.7 Hz, CH), 110.71 (d, J = 26.2 Hz, CH), 104.37 (d, J = 23.1 Hz, CH), 91.51 (C), 77.19 (C), 50.75 (CH_2), 9.58 (CH_3). **HRMS** (ESI) calculated for $C_{31}H_{23}N_2OFNa$ $[M+Na]^+$ requires m/z = 481.1692, found m/z 481.1695.

N-benzyl-N-(2-(5-methoxy-3-methyl-1H-indol-1-yl)phenyl)-3-phenylpropiolamide (1as)

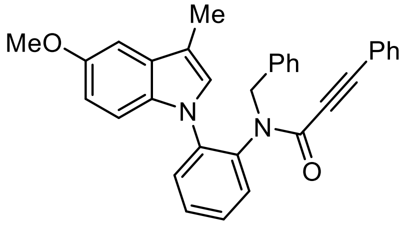

Synthetized from **S-2g** (200 mg, 0.58 mmol), DMAP (3 mg, 0.03 mmol), Et_3N (162 µL, 1.16 mmol), 3-phenylpropioloyl chloride (143 mg, 0.87 mmol) and DCM (5.8 mL). Purification by flash column chromatography (SiO_2 Hex/AcOEt 7:1) yielded **1as** (190 mg, 70%) as a yellow solid.

^{1}H NMR (400 MHz, DMSO, 353K): 7.57 (ddd, J = 11.6, 8.3, 6.6 Hz, 2H), 7.50 – 7.30 (m, 5H), 7.27 – 7.18 (m, 5H), 7.15 – 6.99 (m, 5H), 6.76 (dd, J = 8.9, 2.5 Hz, 1H), 5.07 (d, J = 14.7 Hz, 1H), 3.83 (s, 3H), 3.53 (d, J = 14.7 Hz, 1H), 2.32 (d, J = 1.2 Hz, 3H). **^{13}C NMR** (101 MHz, DMSO, 353 K): 154.74 (C), 153.98 (C), 137.17 (C), 136.94 (C), 136.47 (C), 132.70 (CH), 132.44 (CH), 131.03 (CH), 130.17 (CH), 129.94 (C), 129.31 (CH), 128.97 (CH), 128.84 (CH), 128.76 (CH), 128.20 (CH), 127.90 (CH), 127.04 (CH), 119.86 (C), 112.74 (C), 112.62 (CH), 111.20 (CH), 102.13 (CH), 91.39 (C), 83.33 (C), 56.22 (CH_3), 50.43 (CH_2), 9.78 (CH_3). One C is missing, probably overlapped. **HRMS** (ESI) calculated for $C_{32}H_{26}N_2O_2Na$ $[M+Na]^+$ requires m/z = 493.1892, found m/z 493.1895.

N-benzyl-N-(2-(6-bromo-3-methyl-1H-indol-1-yl)phenyl)-3-phenylpropiolamide (1at)

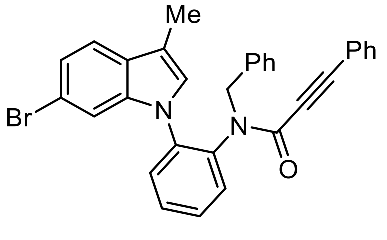

Synthetized from **S-2h** (200 mg, 0.87 mmol), DMAP (3 mg, 0.03 mmol), Et_3N (162 µL), 3-phenylpropioloyl chloride (215 mg, 1.3 mmol) and DCM (3.8 mL). Purification by flash column chromatography (SiO_2 Hex/AcOEt 7:1) yielded **1at** (195 mg, 98%) as a yellow solid.

^{1}H NMR (600 MHz, DMSO, 353 K): 7.62 (t, J = 7.7 Hz, 1H), 7.58 – 7.43 (m, 5H), 7.39 (t, J = 8.1 Hz, 3H), 7.28 – 7.15 (m, 6H), 7.13 (s, 1H), 7.10 – 7.05 (m, 2H), 5.03 (d, J = 14.7 Hz, 1H), 3.77 (d, J = 14.8 Hz, 1H), 2.33 (d, J = 1.2 Hz, 3H). **^{13}C NMR** (151 MHz, DMSO, 353 K): 153.91 (C), 138.05 (C), 137.64 (C), 136.39 (C), 132.65 (CH), 132.46 (CH), 131.06 (CH), 130.41 (CH), 129.35 (CH), 129.24 (CH), 129.01 (CH), 128.85 (CH), 128.73 (CH), 128.37 (C), 127.92 (CH), 127.53 (CH), 123.16 (CH), 121.21 (CH), 119.75 (C), 115.85 (C), 113.34 (CH), 113.16 (C), 91.50 (C), 83.24 (C), 50.90 (CH2), 9.54 (CH3). One C is missing, probably overlapped. **HRMS** (ESI) calculated for $C_{31}H_{23}N_2ONaBr$ $[M+Na]^+$ requires m/z = 541.0891, found m/z 541.0881.

N-benzyl-N-(2-(7-bromo-3-methyl-1H-indol-1-yl)phenyl)-3-phenylpropiolamide (1au)

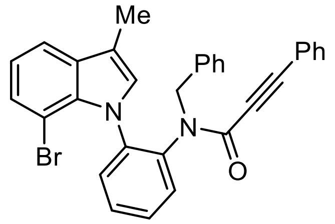

Synthetized from **S-2i** (783 mg, 2.00 mmol), DMAP (11 mg, 0.10 mmol), Et_3N (0.6 mL, 4.00 mmol), 3-phenylpropioloyl chloride (494 mg, 3.00 mmol) and DCM (20 mL). Purification by flash column chromatography (SiO_2, Hex/AcOEt 9:1 → 8:2) yielded **1au** (585 mg, 56%) as an orange solid.
^{1}H NMR (400 MHz, DMSO, 363 K): 7.65 (d, *J* = 7.8 Hz, 1H), 7.48 (d, *J* = 17.7 Hz, 4H), 7.40 (d, *J* = 7.5 Hz, 4H), 7.31 – 6.90 (m, 9H), 4.94 (d, *J* = 14.6 Hz, 1H), 3.89 (d, *J* = 14.7 Hz, 1H), 2.35 – 2.28 (m, 3H). ^{13}C NMR (101 MHz, DMSO) : 154.17 (C), 138.38 (C), 136.86 (C), 136.58 (C), 133.62 (C), 132.99 (CH), 132.62 (CH), 132.30 (CH), 132.18 (CH), 132.10 (C), 131.37 (CH), 129.78 (CH), 129.46 (CH), 129.00 (CH), 128.81 (CH), 128.57 (CH), 127.93 (CH), 127.88 (CH), 121.76 (CH), 119.54 (CH), 119.45 (C), 113.21 (C), 103.36 (C), 92.00 (C), 82.89 (C), 49.99 (CH_2), 9.78 (CH_3). Because of the presence of conformers with high energy barriers, ^{13}C-NMR analysis shows the signals of both. **HRMS** (ESI) calculated for $C_{31}H_{23}N_2ONaBr$ [M+Na]$^+$ requires m/z = 541.0891, found m/z 541.0886.

N-methyl-N-(2-(3-methyl-1H-indol-1-yl)phenyl)-3-phenylpropiolamide (1av)

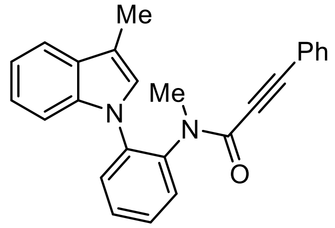

Synthetized according to the general procedure, from *N*-methyl-2-(3-methyl-1*H*-indol-1-yl)aniline[14] (276 mg, 1.17 mmol), DMAP (6.5 mg, 0.06 mmol), Et_3N (326 µL, 2-34 mmol), 3-phenylpropioloyl chloride (288 mg, 1.76 mmol) and DCM (11 mL). Purification of the crude by Biotage® Selekt (Hex/AcOEt 97:3 → 74:26) yielded **1av** (249 mg, 58%) as a yellow solid.
^{1}H NMR (400 MHz, DMSO, 363 K): 7.73 (m, 1H), 7.66 – 7.53 (m, 5H), 7.47 (t, *J* = 7.4 Hz, 1H), 7.39 (t, *J* = 7.6 Hz, 2H), 7.20 (d, *J* = 7.5 Hz, 1H), 7.10 (dq, *J* = 15.7, 7.8 Hz, 4H), 2.84 (s, 3H), 2.34 (d, *J* = 1.2 Hz, 3H). **^{13}C NMR** (101 MHz, DMSO, 363 K): 153.54 (C), 147.60 (C), 147.10 (C), 139.54 (C), 137.38 (C), 136.82 (C), 132.36 (CH), 131.18 (CH), 130.85 (CH), 130.02 (CH), 129.26 (CH), 129.04 (CH), 128.92 (CH), 126.57 (CH), 122.66 (CH), 120.11 (CH), 119.32 (CH), 112.71 (C), 110.39 (CH), 90.42 (C), 83.43 (C), 35.56 (CH_3), 9.63 (CH_3). **HRMS** (ESI) calculated for $C_{25}H_{20}N_2ONa$ [M+Na]$^+$ requires m/z = 387.1474, found m/z 387.1472.

N-(4-methoxybenzyl)-N-(2-(3-methyl-1H-indol-1-yl)phenyl)-3-phenylpropiolamide (1ax)

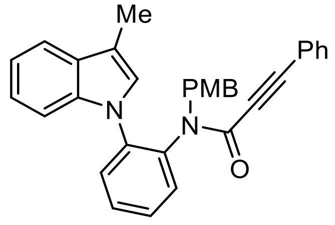

Synthetized from **S-2j** (400mg, 1.17 mmol), DMAP (6.5 mg, 0.06 mmol), Et_3N (326 µL, 2.34 mmol), 3-phenylpropioloyl chloride (288 mg, 1.76 mmol) and DCM (11 mL). Purification of the crude by Biotage® Selekt (Hex/AcOEt 97:3 → 74:26) yielded **1ax** (551 mg, 75%) as a yellow solid.
^{1}H NMR (400 MHz, DMSO, 363 K): 7.65 – 7.28 (m, 8H), 7.24 – 7.07 (m, 6H), 7.01 – 6.93 (m, 2H), 6.77 (d, *J* = 8.0 Hz, 2H), 4.99 (d, *J* = 14.5 Hz, 1H), 3.71 (s, 3H), 3.53 (d, *J* = 14.5 Hz, 1H), 2.35 (s, 3H). **^{13}C NMR** (101 MHz, DMSO, 363 K): 159.38 (C), 153.91 (C), 137.41 (C), 137.34 (C), 136.93 (C), 132.79 (CH), 132.41 (CH), 130.96 (CH), 130.27 (CH), 130.13 (CH), 129.40 (C), 129.28 (CH), 129.09 (CH), 128.50 (C), 128.39 (CH), 126.44 (CH), 122.75 (CH), 120.21 (CH), 119.94 (C), 119.40 (CH), 114.39 (CH), 112.92 (C), 110.48 (CH), 91.33 (C), 83.46 (C), 55.60 (CH_3), 49.99 (CH_2), 9.65 (CH_3). **HRMS** (ESI) calculated for $C_{32}H_{26}N_2O_2Na$ [M+Na]$^+$ requires m/z = 493.1892, found m/z = 493.1898.

***N*-benzyl-*N*-(3-methyl-2-(3-methyl-1*H*-indol-1-yl)phenyl)-3-phenylpropiolamide (1ay)**

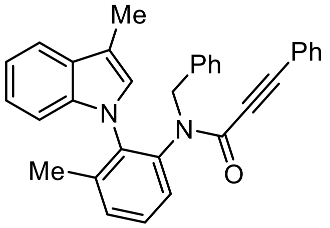

Synthetized from **S-2k** (163 mg, 0.5 mmol), DMAP (2.8 mg, 0.03 mmol), Et_3N (135 µL, 1 mmol), 3-phenylpropioloyl chloride (123 mg, 0.75 mmol) and DCM (5 mL). Purification by flash column chromatography (SiO_2, Hex/AcOEt 7:1) yielded **1ay** (224 mg, 98%) as a yellow solid.

^{1}H NMR (500 MHz, DMSO): 7.64 (ddt, *J* = 31.0, 7.9, 1.0 Hz, 1H), 7.57 – 7.46 (m, 2H), 7.46 – 7.37 (m, 3H), 7.36 – 7.12 (m, 8H), 7.11 – 7.02 (m, 1H), 7.02 – 6.88 (m, 3H), 5.03 (dd, *J* = 46.9, 14.7 Hz, 1H), 3.51 (dd, *J* = 404.9, 14.7 Hz, 1H), 2.36 (dd, *J* = 6.6, 1.1 Hz, 3H), 1.88 (d, *J* = 19.9 Hz, 3H). **^{13}C NMR** (75 MHz, DMSO): 153.83 (C), 139.14 (C), 138.78 (C), 136.96 (C), 136.81 (C), 136.24 (C), 135.65 (C), 132.56 (CH), 132.31 (CH), 131.79 (CH), 131.12 (CH), 130.05 (CH), 129.45 (CH), 128.87 (CH), 128.70 (CH), 128.00 (CH), 127.13 (CH), 123.21 (CH), 119.76 (CH), 119.60 (C), 119.43 (CH), 112.70 (C), 110.18 (CH), 91.72 (C), 83.44 (C), 50.92 (CH_2), 17.50 (CH_3), 9.96 (CH_3). Because of the presence of conformers with high energy barriers, NMR analyses show the signals of both. **HRMS** (ESI) calculated for $C_{32}H_{26}N_2ONa$ $[M+Na]^+$ requires m/z = 477.1943., found m/z 477.1948.

***N*-benzyl-*N*-(3-bromo-2-(3-methyl-1*H*-indol-1-yl)phenyl)-3-phenylpropiolamide (1az)**

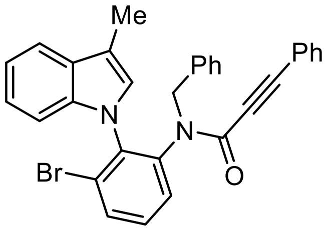

Synthetized from **S-2l** (783 mg, 2.00 mmol), DMAP (11 mg, 0.10 mmol), Et_3N (0.6 mL, 4.00 mmol), 3-phenylpropioloyl chloride (494 mg, 3.00 mmol) and DCM (20 mL). Purification by flash column chromatography (SiO_2, Hex/AcOEt 9:1) yielded **1au** (639 mg, 61%) as an orange solid.

^{1}H NMR (400 MHz, DMSO): 7.96 (ddd, *J* = 10.5, 8.2, 1.4 Hz, 1H), 7.63 (ddt, *J* = 21.8, 7.8, 1.0 Hz, 1H), 7.56 – 7.35 (m, 5H), 7.32 – 7.15 (m, 7H), 7.09 (ddd, *J* = 7.9, 5.9, 2.0 Hz, 1H), 7.06 – 6.84 (m, 3H), 5.02 (dd, *J* = 31.6, 14.7 Hz, 1H), 3.52 (dd, *J* = 325.5, 14.7 Hz, 1H), 2.36 (dd, *J* = 6.6, 1.2 Hz, 3H). **^{13}C NMR** (101 MHz, DMSO): 153.60 (C), 141.19 (C), 136.77 (C), 136.54 (C), 135.91 (C), 134.59 (CH), 132.59 (CH), 132.38 (CH), 131.43 (CH), 130.93 (CH), 129.60 (CH), 129.11 (C), 128.94 (CH), 128.77 (CH), 128.15 (CH), 125.35 (CH), 124.98 (C), 123.29 (CH), 120.11 (CH), 119.51 (CH), 119.41 (C), 113.46 (C), 110.79 (CH), 92.25 (C), 83.19 (C), 50.89 (CH_2), 10.00 (CH_3). Because of the presence of conformers with high energy barriers, NMR analyses show the signals of both. **HRMS** (ESI) calculated for $C_{31}H_{23}N_2ONaBr$ $[M+Na]^+$ requires m/z = 541.0891, found m/z 541.0890.

***N*-benzyl-*N*-(4-methyl-2-(3-methyl-1*H*-indol-1-yl)phenyl)-3-phenylpropiolamide (1ba)**

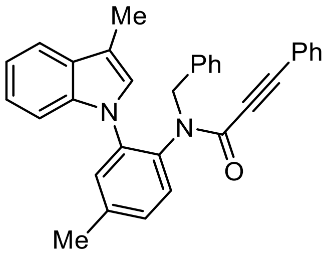

Synthetized from **S-2m** (235 mg, 0.72 mmol), DMAP (4 mg, 0.036 mmol), Et_3N (201 µL, 1.44 mmol), 3-phenylpropioloyl chloride (178 mg, 1.08 mmol) and DCM (7.2 mL). Purification by flash column chromatography (SiO_2, Hex/AcOEt 7:1) yielded **1ba** (264 mg, 81%) as a yellow solid.

^{1}H NMR (500 MHz, DMSO, 353 K): 7.62 (m, 1H), 7.47 (d, *J* = 7.6 Hz, 1H), 7.44 – 7.37 (m, 3H), 7.24 (dtd, *J* = 15.0, 8.1, 3.9 Hz, 7H), 7.18 – 7.10 (m, 3H), 7.09 – 7.03 (m, 3H), 5.04 (d, *J* = 14.6 Hz, 1H), 3.51 (d, *J* = 14.7 Hz, 1H), 2.41 (s, 3H), 2.35 (s, 3H). **^{13}C NMR** (126 MHz, DMSO, 353 K): 154.07 (C), 140.24 (C), 137.27 (C), 136.55 (C), 136.46 (C), 134.71 (C), 132.43 (CH), 132.30 (CH), 131.00 (CH), 129.48 (CH), 129.34 (CH), 129.30 (C), 129.01 (CH), 128.81 (CH), 128.76 (CH), 127.88 (CH), 126.48 (CH), 122.76 (CH), 120.17 (CH), 119.95 (C), 119.43 (CH) 112.82 (C), 110.54 (CH), 91.35 (C), 83.43 (C), 50.49 (CH_2), 20.90 (CH_3), 9.71 (CH_3). **HRMS** (ESI) calculated for $C_{32}H_{26}N_2ONa$ $[M+Na]^+$ requires m/z = 477.1943., found m/z 477.1945.

Synthesis of differently substituted starting materials

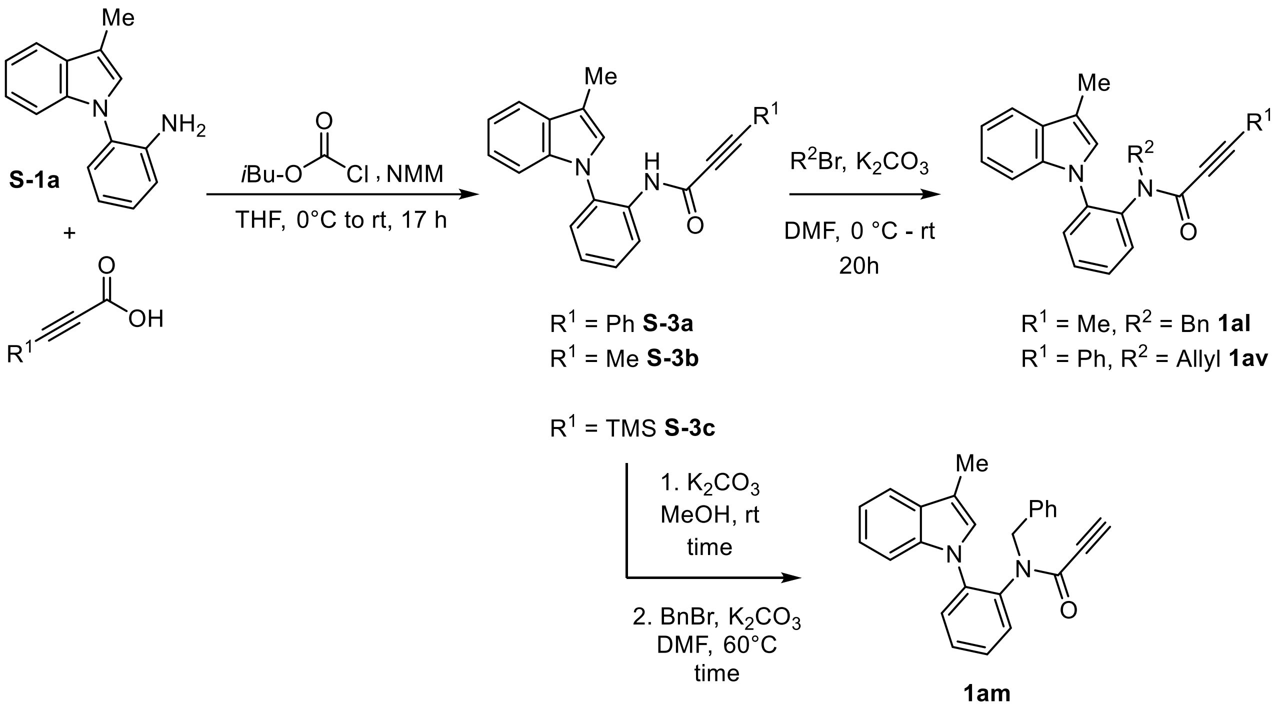

General procedure for the synthesis of *N*-(2-(3-methyl-1*H*-indol-1-yl)phenyl)-propiolamides (S-3a-c)

Under nitrogen atmosphere, the appropriate propiolic acid (1.2 eqiv.) and 4-methylmorpholine (1.8 equiv) were dissolved in THF (0.2 M) at 0°C. Then isobutyl chloroformate (1.5 equiv.) was added dropwise and the reaction mixture was stirred at 0°C for 0.5 h. Subsequently **S-1a** (1 equiv.) in THF (0.15 M) was added to the solution at 0 °C, and the mixture was stirred at 0 °C for 1 h and at room temperature for 16 h. The reaction was quenched with water and extracted with DCM. The organic layer was washed with brine, dried over Na_2SO_4, and concentrated under reduced pressure. Purification of the crude by flash column chromatography (SIO_2) yielded the corresponding amides **S-4**.

General procedure for the protection of the amidic group

Under nitrogen atmosphere, **S-3a-c** (1 equiv.) was dissolved in DMF (0.2M). Then allyl or benzyl bromide (1.3 equiv.) and K_2CO_3 (2 equiv.) were added at 0 °C and the reaction was allowed to warm up to rt. After 2 h the mixture was quenched with H_2O and extracted three times with AcOEt. The organic layer was washed with brine, dried over Na_2SO_4, and concentrated under vacuum. Purification of the crude by flash column chromatography yielded the corresponding protected product **1**.

***N*-(2-(3-methyl-1*H*-indol-1-yl)phenyl)-3-phenylpropiolamide (S-3a)**

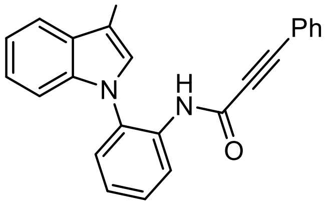

Synthetized from **S-1a** (200 mg, 0.89 mmol), 3-phenylpropiolic acid (158 mg, 1.08 mmol), 4-methylmorpholine (177 µL, 1.8 mmol), isobutylchloroformiate (176 µL) in 5.6 mL of THF. Purification of the crude by flash column chromatography (SiO_2, Hex/AcOEt 9:1) yielded **S-3a** (160 mg, 51%) as a pale yellow solid.

^{1}H NMR (300 MHz, $CDCl_3$): 8.51 (d, *J* = 8.3 Hz, 1H), 7.67 (m, 1H), 7.52 – 7.37 (m, 5H), 7.35 – 7.18 (m, 6H), 7.13 (dd, *J* = 7.5, 1.9 Hz, 1H), 7.02 (d, *J* = 1.3 Hz, 1H), 2.44 (d, *J* = 1.2 Hz, 3H). **^{13}C NMR** (75 MHz, $CDCl_3$): 151.00 (C), 137.21 (C), 133.79 (C), 132.66 (CH), 130.36 (CH), 129.42 (C), 128.77 (CH), 128.70 (C), 128.48 (CH), 128.06 (CH), 126.01 (CH), 125.12 (CH), 123.05 (CH), 122.15 (CH), 120.27 (CH), 119.69 (C), 119.38 (CH), 114.16 (C), 110.41 (CH), 86.24 (C), 83.16 (C), 9.68 (CH_3). **HRMS** (ESI) calculated for $C_{24}H_{18}N_2ONa$ $[M+Na]^+$ requires m/z = 373.1317, found m/z 373.1312.

N-(2-(3-methyl-1H-indol-1-yl)phenyl)but-2-ynamide (S-3b)

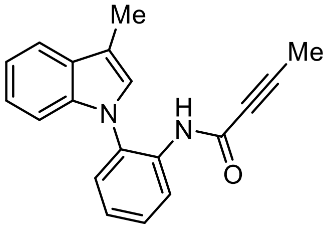

Synthetized from **S-1a** (445 mg, 2 mmol), 3-methylpropiolic acid (202 mg, 2.4 mmol), 4-methylmorpholine (397 µL, 3.6 mmol), isobutylchloroformiate (394 µL, 03 mmol) in 12.5 mL of THF. Purification of the crude by flash column chromatography (SiO_2, Hex/AcOEt 5:1) yielded **S-3b** (362 mg, 60%) as a pale yellow solid.

^{1}H NMR (300 MHz, $CDCl_3$): 8.46 (d, J = 8.3 Hz, 1H), 7.72 – 7.62 (m, 1H), 7.43 (ddd, J = 8.6, 6.8, 2.3 Hz, 1H), 7.30 – 7.16 (m, 5H), 7.11 – 7.05 (m, 1H), 6.97 (q, J = 1.2 Hz, 1H), 2.42 (d, J = 1.1 Hz, 3H), 1.86 (s, 3H). **^{13}C NMR** (75 MHz, $CDCl_3$): 151.04 (C), 137.32 (C), 133.92 (C), 129.37 (C), 128.78 (CH), 128.42 (C), 128.12 (CH), 126.03 (CH), 124.89 (CH), 122.99 (CH), 122.01 (CH), 120.20 (CH), 119.25 (CH), 114.05 (C), 110.38 (CH), 85.21 (C), 75.09 (C), 9.66 (CH_3), 3.80 (CH_3). **HRMS** (ESI) calculated for $C_{19}H_{17}N_2O$ $[M+H]^+$ requires m/z = 289.1336, found m/z 289.1336.

N-(2-(3-methyl-1H-indol-1-yl)phenyl)-3-(trimethylsilyl)propiolamide (S-3c)

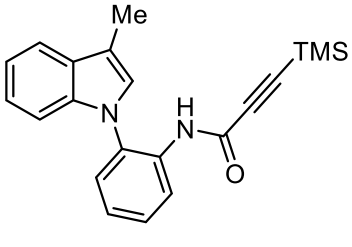

Synthetized from **S-1a** (725 mg, 3.3 mmol), 3-(trimethylsilyl)propiolic acid (382 mg, 2.7 mmol), 4-methylmorpholine (643 µL, 5.8 mmol), isobutylchloroformiate (652 µL, 4.9 mmol) in 16.5 mL of THF Purification of the crude by flash column chromatography (SiO_2, Hex/AcOEt 95:5) yielded **S-3c** (748 mg, 65%) as a pale yellow solid.

^{1}H NMR (400 MHz, $CDCl_3$): 8.44 (d, J = 8.3 Hz, 1H), 7.74 – 7.65 (m, 1H), 7.46 (ddd, J = 8.5, 7.4, 1.7 Hz, 1H), 7.37 – 7.22 (m, 5H), 7.13 (dd, J = 7.6, 1.7 Hz, 1H), 7.01 (d, J = 1.3 Hz, 1H), 2.45 (d, J = 1.2 Hz, 3H), 0.17 (s, 9H). **^{13}C NMR** (101 MHz, $CDCl_3$): 151.17 (C), 138.07 (C), 134.52 (C), 130.26 (C), 129.61 (CH), 129.51 (C), 128.83 (CH), 126.81 (CH), 126.01 (CH), 123.93 (CH), 123.09 (CH), 121.15 (CH), 120.20 (CH), 115.01 (C), 111.30 (CH), 98.34 (C), 94.28 (C), 10.49 (CH_3), 0.00 (CH_3). **HRMS** (ESI) calculated for $C_{21}H_{23}N_2OSi$ $[M+H]^+$ requires m/z = 347.1575, found m/z 347.1577.

N-benzyl-N-(2-(3-methyl-1H-indol-1-yl)phenyl)but-2-ynamide (1al)

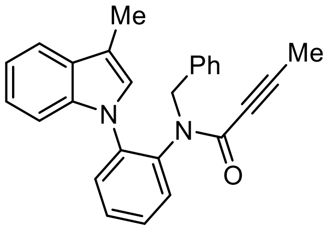

Synthetized from **S-3b** (277 mg, 0.96 mmol), benzyl bromide (148 µL, 1.25mmol), K_2CO_3 (265 mg, 1.92 mmol) in 4.8 mL of DMF. Purification of the crude by Biotage® Selekt (Hex/AcOEt 87:13 → 75:25) yielded **1al** (289 mg, 80%) as a pale yellow solid.

^{1}H NMR (600 MHz, DMSO, 353 K): 7.62 (dd, J = 8.0, 1.4 Hz, 1H), 7.57 – 7.31 (m, 3H), 7.18 (dt, J = 33.8, 7.3 Hz, 7H), 7.03 – 6.99 (m, 3H), 5.00 (d, J = 14.7 Hz, 1H), 3.51 (d, J = 14.7 Hz, 1H), 2.35 (s, 3H), 1.89 (d, J = 137.0 Hz, 3H). **^{13}C NMR** (151 MHz, DMSO, 353 K): 154.02 (C), 137.38 (C), 137.18 (C), 136.69 (C), 136.59 (C), 132.33 (CH), 129.88 (CH), 129.36 (C), 128.91 (CH), 128.76 (CH), 128.72 (CH), 128.26 (CH), 127.83 (CH), 126.41 (CH), 122.80 (CH), 120.22 (CH), 119.42 (CH), 112.80 (C), 110.51 (CH), 91.27 (C), 74.78 (C), 50.48 (CH_2), 9.69 (CH_3), 3.63 (CH_3).). Because of the presence of conformers with high energy barriers, NMR analyses show the signals of both. **HRMS** (ESI) calculated for $C_{26}H_{23}N_2O$ $[M+H]^+$ requires m/z = 379.1810, found m/z 379.1812.

N-benzyl-N-(2-(3-methyl-1H-indol-1-yl)phenyl)propiolamide (1am)

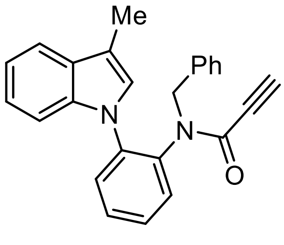

To a stirring solution of **S-3c** (228 mg, 0.66 mmol) in 5.3 mL of MeOH, K_2CO_3 (137 mg, 0.99 mmol) was added and the reaction was stirred at rt for 2h. MeOH was removed under reduced pressure, then the mixture was extracted three times with H_2O/AcOEt. The organic layer was washed with brine, dried over Na_2SO_4 and concentrated under vacuum. The crude (181 mg, 0.66 mmol) was dissolved in 3.5 mL of DMF and directly used for the next step without further purification. Benzyl bromide (108 µL, 0.91 mmol) and K_2CO_3 (193 mg, 1.4 mmol) were added, and the solution was left stirring for 2h. Afterward water was added and then extracted three times with AcOEt. The organic layer was then dried over Na_2SO_4 and concentrated under reduced pressure. Purification of the crude by column chromatography (SiO_2, Hex/AcOEt 9:1 → 3:1) yielded **1am** (95 mg, 39%) as a white thick solid.

^{1}H NMR (500 MHz, DMSO): 7.63 (m, 1H), 7.59 – 7.46 (m, 2H), 7.37 (m, 1H), 7.28 – 7.08 (m, 6H), 7.07 – 6.97 (m, 3H), 4.98 (d, J = 14.6 Hz, 1H), 3.44 (d, J = 14.7 Hz, 1H), 3.10 (s, 1H), 2.36 (s, 3H). **^{13}C NMR** (126 MHz, DMSO): 153.28 (C), 137.11 (C), 136.87 (C), 136.62 (C), 136.21 (C), 132.58 (CH), 130.28 (CH), 129.36 (C), 129.09 (CH), 128.81 (CH), 128.75 (CH), 128.46 (CH), 127.94 (CH), 126.36 (CH), 122.92 (CH), 120.29 (CH), 119.49 (CH), 113.03 (C), 110.50 (CH), 83.84 (CH), 77.16 (C), 50.42 (CH_2), 9.70 (CH_3). Because of the presence of conformers with high energy barriers, ^{13}C-NMR analysis shows the signals of both. **HRMS** (ESI) calculated for $C_{25}H_{20}N_2ONa$ $[M+Na]^+$ requires m/z = 387.1468, found m/z 387.1466.

N-allyl-N-(2-(3-methyl-1H-indol-1-yl)phenyl)-3-phenylpropiolamide (1aw)

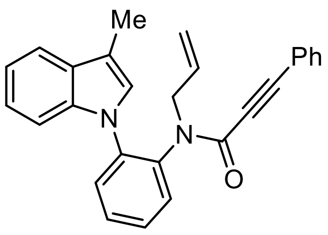

Synthetized from **S-3a** (220 mg, 0.63 mmol), allyl bromide (60 µL, 0.82 mmol), K_2CO_3 (174 mg, 1.26 mmol) in 3.2 mL of DMF. Purification of the crude by Biotage® Selekt (Hex/AcOEt 94:6→70:30) yielded **1aw** (190 mg, 77%) as a pale-yellow solid.

^{1}H NMR (300 MHz, $CDCl_3$): 7.62 (m, 1H), 7.58 – 7.49 (m, 3H), 7.49 – 7.32 (m, 3H), 7.32 – 7.15 (m, 6H), 7.02 (q, J = 1.2 Hz, 1H), 5.55 (dddd, J = 16.9, 10.1, 7.6, 5.3 Hz, 1H), 5.02 – 4.76 (m, 2H), 4.49 (ddt, J = 14.9, 5.3, 1.5 Hz, 1H), 2.90 (ddt, J = 14.9, 7.8, 1.1 Hz, 1H), 2.37 (dd, J = 4.7, 1.1 Hz, 3H). **^{13}C NMR** (75 MHz, $CDCl_3$): 154.29 (C), 136.99 (C), 136.79 (C), 136.73 (C), 132.70 (CH), 132.58 (CH), 131.52 (CH), 130.24 (CH), 129.39 (CH), 129.25 (C), 128.55 (CH), 128.45 (CH), 127.37 (CH), 125.48 (CH), 122.53 (CH), 120.16 (C), 119.99 (CH), 119.19 (CH), 118.91 (CH_2), 113.60 (C), 109.89 (CH), 91.71 (C), 82.70 (C), 49.44 (CH_2), 9.66 (CH_3). Because of the presence of conformers with high energy barriers, ^{13}C-NMR analysis shows the signals of both. **HRMS** (ESI) calculated for $C_{27}H_{22}N_2NaO$ $[M+Na]^+$ requires m/z = 413.1625, found m/z 413.1631

Synthesis of other stating materials

N-benzyl-2-(1*H*-pyrrol-1-yl)aniline (S-4)

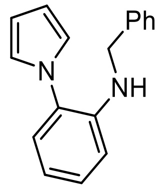

Synthetized from 2-(1*H*-pyrrol-1-yl)aniline[15] (250 mg, 1.58 mmol), benzaldehyde (193 µL, 1.90 mmol) $MgSO_4$ (152 mg, 1.26 mmol), acetic acid (9 µL, 0.16 mmol) sodium triacetoxyborohydride (670 mg, 3.16 mmol) in 2.6 mL of 1,2-dichloroethane. Purification of the crude by Biotage® Selekt (Hex/AcOEt 3% → 20%) yielded **S-4** (259 mg, 66%) as a transparent liquid**.**

^{1}H NMR (300 MHz, $CDCl_3$): 7.32 – 7.20 (m, 6H), 7.17 (m, 1H), 6.86 (t, *J* = 2.1 Hz, 2H), 6.80 (t, *J* = 7.5 Hz, 2H), 6.36 (t, *J* = 2.1 Hz, 2H), 4.30 (bs, 3H). Data are in agreement with those reported in literature.[4]

N-(2-(1*H*-pyrrol-1-yl)phenyl)-*N*-benzyl-3-phenylpropiolamide (3)

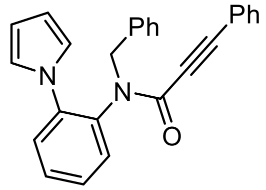

Synthetized from *N*-benzyl-2-(1*H*-pyrrol-1-yl)aniline **S-4** (250 mg, 1 mmol), DMAP (5.6 mg, 0.05 mmol), Et_3N (279 µl, 2 mmol), 3-phenylpropioloyl chloride (247 mg, 1.5 mmol) in 10 mL of DCM. Purification by Biotage® Selekt (Hex/AcOEt 97:3 → 80:20) yielded the corresponding **3** (318 mg, 88%) as a yellow solid.

^{1}H NMR (400 MHz, DMSO, 363 K): 7.48 (dt, *J* = 27.3, 7.3 Hz, 4H), 7.36 (t, *J* = 7.6 Hz, 2H), 7.31 – 7.20 ^{1}H NMR (400 MHz, DMSO, 363 K): 7.48 (dt, *J* = 27.3, 7.3 Hz, 4H), 7.36 (t, *J* = 7.6 Hz, 2H), 7.31 – 7.20 (m, 4H), 7.19 – 7.12 (m, 4H), 6.92 (t, *J* = 2.2 Hz, 2H), 6.32 (t, *J* = 2.2 Hz, 2H), 5.22 (d, *J* = 14.7 Hz, 1H), 3.69 (d, *J* = 14.7 Hz, 1H). **^{13}C NMR** (101 MHz, DMSO, 363 K): 154.11 (C), 138.70 (C), 136.51 (C), 135.56 (C), 132.40 (CH), 132.33 (CH), 130.98 (CH), 130.25 (CH), 129.28 (CH), 128.97 (CH), 128.81 (CH), 127.95 (CH), 127.91 (CH), 127.33 (CH), 122.09 (CH), 119.82 (C), 110.47 (CH), 91.19 (C), 83.12 (C), 50.58 (CH_2). **HRMS** (ESI) calculated for $C_{26}H_{20}N_2ONa$ $[M+Na]^+$ requires m/z = 399.1473 found m/z 399.1472.

Synthesis of *N*-benzyl-*N*-(2-(1-methyl-1*H*-indol-3-yl)phenyl)-3-phenylpropiolamide (5)

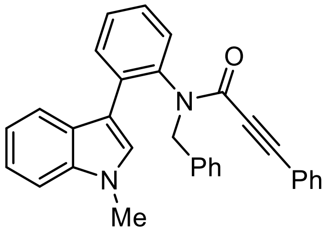

Synthetized according to the general procedure from 2-(1-methyl-1*H*-indol-3-yl)aniline[16] (333 mg, 1.5 mmol), benzaldehyde (184 µL, 1.8 mmol), $MgSO_4$ (144 mg, 1.2 mmol), acetic acid (8.6 µL, 0.15 mmol) and sodium triacetoxyborohydride (636 mg, 3 mmol) in 2.5 mL of 1,2-dichloroethane. Purification of the crude by flash column chromatography (SiO_2, Hex/AcOEt 95:5) yielded *N*-benzyl-2-(1-methyl-1*H*-indol-3-yl)aniline (377 mg, 81%) as a white solid. The product was directly used for the next step adding DMAP (6 mg, 0.6 mmol), Et_3N (315 µL, 2.26 mmol), 3-phenylpropioloyl chloride[2] (292 mg, 1.8 mL) and 12 mL of DCM. Purification by Biotage® Selekt (Hex/AcOEt 5:95 → 60:40) yielded **5** (377 mg, 71%) as a white solid.

^{1}H NMR (400 MHz, DMSO): 7.64 (dd, *J* = 7.7, 1.5 Hz, 1H), 7.57 – 7.48 (m, 3H), 7.43 (m, 1H), 7.37 – 7.18 (m, 8H), 7.12 (ddd, *J* = 9.7, 8.2, 1.6 Hz, 5H), 7.04 (m, 1H), 5.21 (d, *J* = 14.6 Hz, 1H), 3.94 (d, *J* = 14.6 Hz, 1H), 3.86 (d, *J* = 8.1 Hz, 3H). **^{13}C NMR** (101 MHz, DMSO): 154.42 (C), 139.20 (C), 137.15 (C), 136.99 (C), 134.06 (C), 132.48 (CH), 131.80 (CH), 131.46 (CH), 130.94 (CH), 129.29 (CH), 129.15 (CH), 128.91 (CH), 128.83 (CH), 128.32 (CH), 127.86 (CH), 127.11 (CH), 127.05 (C), 122.17 (CH), 120.17 (CH), 119.95 (C), 119.32 (CH), 111.54 (C), 110.65 (CH), 90.63 (C), 83.58 (C), 51.14 (CH_2), 33.13 (CH_3). **HRMS** (ESI) calculated for $C_{31}H_{24}N_2ONa$ $[M+Na]^+$ requires m/z = 463.1786, found m/z 463.1793.

2-(1-methyl-1*H*-indol-2-yl)aniline (S-5)

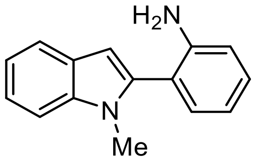

2-iodo-1-methyl-1*H*-indole[17] (451 mg, 1.86 mmol) and $Pd(PPh_3)_4$ (108 mg, 0.093 mmol) were dissolved in 22 mL of toluene, and the mixture was left stirring for 30 minutes at rt. Then a solution of 2-(4,4,5,5-tetramethyl-1,3,2-dioxaborolan-2-yl)aniline (488 mg, 2.23 mmol) in 14 mL of EtOH, followed by 13 mL of $NaHCO_3$ ss. The reaction was heated to reflux for 2h and then diluted with water and AcOEt. The reaction mixture was extracted with AcOEt, dried with $NaSO_4$ and concentrated under reduced pressure. Purification by column chromatography (SiO_2, Hex/AcOEt 7:1) yielded **S-5** (371 mg, 90%) as a white solid.

^{1}H NMR (400 MHz, $CDCl_3$): 7.68 (dt, J = 7.8, 1.1 Hz, 1H), 7.40 (dq, J = 8.2, 1.0 Hz, 1H), 7.32–7.23 (m, 2H), 7.23–7.13 (m, 2H), 6.92–6.79 (m, 2H), 6.57 (d, J = 0.8 Hz, 1H), 3.84 (bs, 2H), 3.62 (s, 3H). Data are in agreement with those reported in literature.[18]

***N*-benzyl-*N*-(2-(1-methyl-1*H*-indol-2-yl)phenyl)-3-phenylpropiolamide (7)**

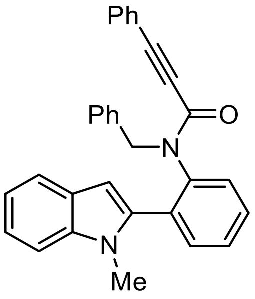

Under nitrogen atmosphere, phenylpropiolic acid (197 mg, 1.35 mmol) and 4-methylmorpholine (222 µL, 2.02 mmol) were dissolved in 2 mL of THF. Then isobutyl chloroformate (221 µL, 1.68 mmol) in 1 mL of THF was added at 0 °C, and the mixture was stirred at 0 °C for 0.5 h. **S-5** (250 mg, 1.12 mmol) in 1 mL of THF was added to the solution at 0 °C, and the mixture was stirred at 0 °C for 1 h and at room temperature for 16 h. The reaction was quenched with water and extracted with DCM. The organic layer was washed with brine, dried over Na_2SO_4, and concentrated under reduced pressure. Purification of the crude by flash column chromatography (SiO_2, Hex/AcOEt 9:1) yielded *N*-(2-(1-methyl-1*H*-indol-2-yl)phenyl)-3-phenylpropiolamide (139 mg, 0.39 mmol) which was directly dissolved in 4.8 mL of DMF and used for the next step. Benzyl bromide (108 mg, 0.78 mmol) and K_2CO_3 (108 mg, 0.78 mmol) were added at 0 °C and the reaction was heated to 60°C. After 2h the end of the reaction was checked by TLC, water was added and the aqueous phase was extracted three times with AcOEt. The organic layer was washed with brine, dried over Na_2SO_4, and concentrated under reduced pressure. Purification of the crude by flash column chromatography (SiO_2, Hex/AcOEt 7:1) yielded **7** (116 mg, 27%) as a pale yellow solid.

^{1}H NMR (600 MHz, DMSO, 353 K): 7.64 – 7.54 (m, 3H), 7.54 – 7.40 (m, 4H), 7.39 – 7.30 (m, 2H), 7.28 (d, J = 7.9 Hz, 1H), 7.24 – 7.16 (m, 5H), 7.15 – 7.07 (m, 3H), 6.60 (s, 1H), 5.09 (d, J = 14.8 Hz, 1H), 4.06 (d, J = 14.8 Hz, 1H), 3.50 (s, 3H). **^{13}C NMR** (151 MHz, DMSO, 353 K): 154.30 (C), 140.84 (C), 138.12 (C), 136.73 (C), 132.82 (CH), 132.41 (CH), 131.74 (C), 131.66 (CH), 130.96 (CH), 129.65 (CH), 129.32 (CH), 128.94 (CH), 128.79 (CH), 128.66 (CH), 127.96 (C), 127.77 (CH), 122.06 (CH), 120.75 (CH), 120.07 (CH), 119.97 (C), 110.56 (CH), 102.81 (CH), 91.30 (C), 83.69 (C), 51.73 (CH_2), 31.08 (CH_3). One C is missing, probably overlapped. **HRMS** (ESI) calculated for $C_{31}H_{24}N_2ONa$ $[M+Na]^+$ requires m/z = 463.1783 found m/z 463.1791.

2-(3-methyl-1*H*-indol-1-yl)phenyl 3-phenylpropiolate (9)

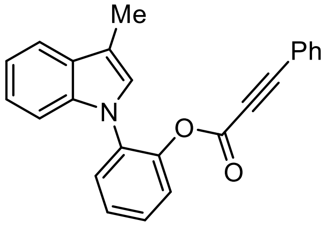

Synthetized according to the general procedure from 2-(3-methyl-1*H*-indol-1-yl)phenol[12] (223 mg, 1 mmol), DMAP (5.6 mg, 0.05 mmol), Et_3N (279 µl, 2 mmol), 3-phenylpropioloyl chloride (247 mg, 1.5 mmol) in 10 mL of DCM. Purification by Biotage® Selekt (Hex/AcOEt 97:3 → 80:20) yielded **13** (230 mg, 66%) as a yellow solid.

^{1}H NMR (300 MHz, $CDCl_3$): 7.59 (m, 1H), 7.55 – 7.32 (m, 9H), 7.30 – 7.10 (m, 3H), 7.03 (q, J = 1.2 Hz, 1H), 2.36 (t, J = 1.1 Hz, 3H). **^{13}C NMR** (75 MHz, $CDCl_3$): 151.48 (C), 144.93 (C), 136.71 (C), 133.18 (CH), 132.31 (C), 131.04 (CH), 129.38 (C), 128.60 (CH), 128.23 (CH), 128.04 (CH), 127.41 (CH), 125.91 (CH), 123.79 (CH), 122.43 (CH), 119.87 (CH), 119.10 (C), 119.00 (CH), 113.21 (C), 110.66 (CH), 89.27 (C), 79.58 (C), 9.67 (CH_3). **HR-MS** (ESI) calculated for $C_{24}H_{17}NO_2$ $[M+H]^+$ requires m/z = 352.1333 found m/z 352.1335.

Catalytic reactions

General procedure for the asymmetric synthesis of axially chiral indole-fused diazocine bridged biaryls 2aa-ba

Under nitrogen atmosphere, chiral catalyst **L_7**AuCl (5 mol%) and $AgSbF_6$ (5 mol%) were stirred in chlorobenzene (Ph-Cl, 0.1 M) for 10 minutes. Then **1** (1 equiv.) was added in one portion and the reaction was left stirring at room temperature for 24 hours. The solvent was eliminated under reduced pressure and purification by column chromatography (SiO_2) yielded the corresponding diazocino-indolo derivative **2aa-ba.**

General procedure for the synthesis of racemic samples of 2aa-ba

Under nitrogen atmosphere, AuJohnPhosSbF_6(CH_3CN) (5 mol%) and **1** were stirred in 1,2-dichloroethane (0.1 M) at room temperature for 24 hours. The solvent was removed under reduced pressure and purification by column chromatography yielded the racemic product **2aa-ba.**

(*S,Z*)-5-benzyl-9-methyl-8-phenylbenzo[2,3][1,4]diazocino[1,8-*a*]indol-6(5*H*)-one (2aa)

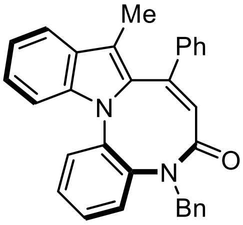

Synthetized from **1aa** (44 mg, 0.1 mmol), **L_7**AuCl (6.0 mg, 0.005 mmol), $AgSbF_6$ (1.7 mg, 0.005 mmol) and 1 mL of Ph-Cl. Purification by Biotage® Selekt (Hex/AcOEt 95:5 → 60:40) gave **2aa** (41 mg, 94% yield) as a white solid.

^{1}H NMR (400 MHz, $CDCl_3$): 7.60 (dt, *J* = 7.9, 1.0 Hz, 1H), 7.47 – 7.37 (m, 2H), 7.36 – 7.32 (m, 2H), 7.32 - 7.25 (m, 5H), 7.19 (ddd, *J* = 8.0, 7.0, 1.0 Hz, 1H), 7.07 (ddd, *J* = 8.3, 7.0, 1.2 Hz, 1H), 6.96 (m, 1H), 6.82 – 6.74 (m, 2H), 6.74 – 6.69 (m, 2H), 6.63 (s, 1H), 6.59 (dt, *J* = 8.2, 0.9 Hz, 1H), 5.57 (d, *J* = 14.6 Hz, 1H), 4.39 (d, *J* = 14.6 Hz, 1H), 1.97 (s, 3H).

^{13}C NMR (101 MHz, $CDCl_3$): 168.38 (C), 139.82 (C), 139.02 (C), 137.53 (C), 136.35 (C), 136.02 (C), 135.36 (C), 134.49 (C), 128.99 (C), 128.69 (CH), 128.54 (CH), 128.51 (CH), 128.38 (CH), 128.13 (CH), 127.99 (CH), 127.56 (CH), 127.31 (CH), 127.22 (CH), 125.00 (CH), 122.88 (CH), 120.23 (CH), 118.73 (CH), 112.78 (C), 110.99 (CH), 52.46 (CH_2), 9.60 (CH_3). One CH is missing, probably overlapped.

HRMS (ESI) calculated for $C_{31}H_{24}N_2ONa$ $[M+H]^+$ requires m/z = 463.1786, found m/z 463.1784.

$[\alpha]^D{}_{25}$ = +300.0 (c = 0.25, $CHCl_3$).

R_t (OD-H column, Hex/*i*-PrOH 85:15, 0.8 mL/min, 266.4 nm): tr(major) = 11.2 min, tr(minor) = 7.7 min, 2:98 e.r.

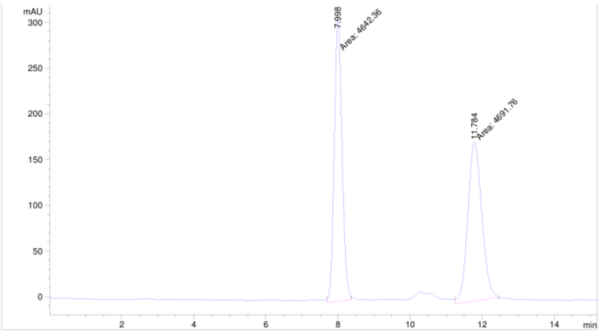

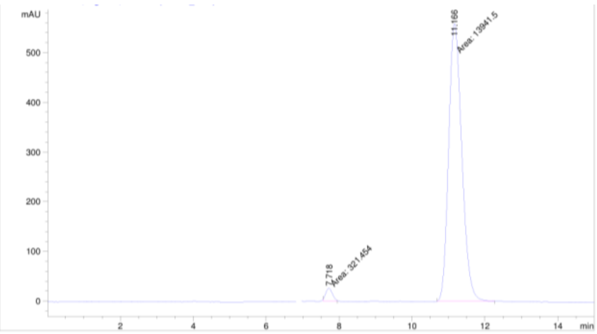

Peak #	RetTime [min]	Type	Width [min]	Area [mAU*s]	Height [mAU]	Area %
1	7.998	MM	0.2519	4642.36377	307.18948	49.7354
2	11.784	MM	0.4496	4691.75830	173.92520	50.2646

Peak #	RetTime [min]	Type	Width [min]	Area [mAU*s]	Height [mAU]	Area %
1	7.718	MM	0.2117	321.45370	25.31201	2.2538
2	11.166	MM	0.4164	1.39415e4	557.95154	97.7462

(*S,Z*)-5-benzyl-9-methyl-8-(p-tolyl)benzo[2,3][1,4]diazocino[1,8-*a*]indol-6(5*H*)-one (2ab)

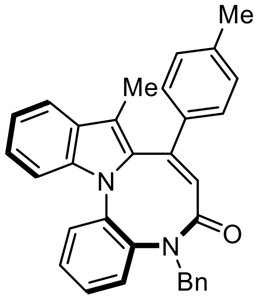

Synthetized from **1ab** (45 mg, 0.1 mmol), **L$_7$**AuCl (6.0 mg, 0.005 mmol), AgSbF$_6$ (1.7 mg, 0.005 mmol) and 1 mL of Ph-Cl. Purification by Biotage® Selekt (Hex/AcOEt 97:3 → 70:30) gave **2ab** (32 mg, 72% yield) as a white solid.

^{1}H NMR (300 MHz, CDCl$_3$): 7.57 (dt, *J* = 7.8, 0.9 Hz, 1H), 7.44 – 7.34 (m, 2H), 7.30 (dd, *J* = 3.8, 1.0 Hz, 2H), 7.19 – 7.11 (m, 3H), 7.10 – 7.00 (m, 3H), 6.92 (d, *J* = 7.2 Hz, 1H), 6.80 – 6.60 (m, 4H), 6.59 – 6.52 (m, 2H), 5.53 (d, *J* = 14.6 Hz, 1H), 4.35 (d, *J* = 14.6 Hz, 1H), 2.31 (s, 3H), 1.95 (s, 3H).

^{13}C NMR (75 MHz, CDCl$_3$): 168.53 (C), 139.85 (C), 138.78 (C), 137.44 (C), 136.17 (C), 136.02 (C), 135.97 (C), 135.36 (C), 134.50 (C), 129.20 (CH), 128.94 (C), 128.44 (CH), 128.32 (CH), 128.08 (CH), 127.94 (CH), 127.49 (CH), 127.17 (CH), 127.14 (CH), 124.11 (CH), 122.76 (CH), 120.14 (CH), 118.67 (CH), 112.62 (CH), 110.92 (C), 52.41 (CH$_2$), 21.16 (CH$_3$), 9.63 (CH$_3$). One CH is missing, probably overlapped.

HRMS (ESI) calculated for $C_{32}H_{26}N_2ONa$ [M+Na]$^+$ requires m/z = 477.1943, found m/z 477.1951.

$[\alpha]^D_{25}$ = +283.0 (c = 0.25, CHCl$_3$).

R$_t$ (OD-H column, Hex/*i*-PrOH 80:20, 0.8 mL/min, 266.4 nm): tr(major) = 8.8 min, tr(minor) = 6.4 min, 2:98 e.r.

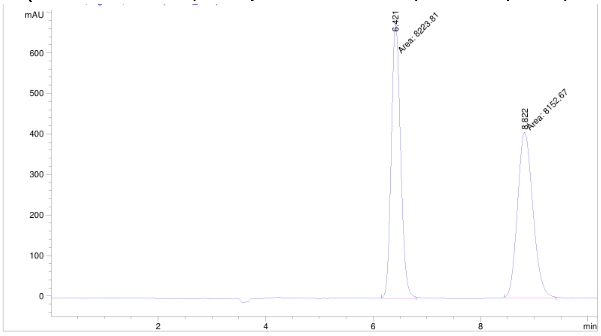

Peak #	RetTime [min]	Type	Width [min]	Area [mAU*s]	Height [mAU]	Area %
1	6.421	MM	0.2015	8223.81055	680.15985	50.2172
2	8.822	MM	0.3322	8152.67383	409.07816	49.7828

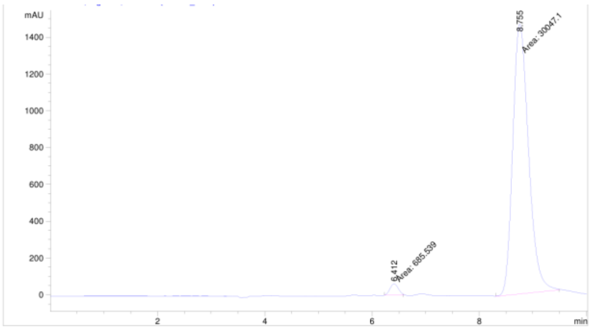

Peak #	RetTime [min]	Type	Width [min]	Area [mAU*s]	Height [mAU]	Area %
1	6.412	MM	0.1831	685.53851	62.40894	2.2307
2	8.755	MM	0.3410	3.00471e4	1468.75415	97.7693

(*S,Z*)-5-benzyl-8-(4-methoxyphenyl)-9-methylbenzo[2,3][1,4]diazocino[1,8-*a*]indol-6(5*H*)-one (2ac)

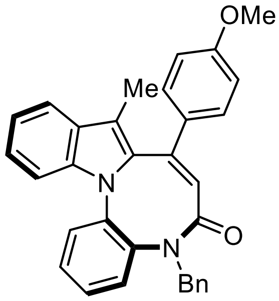

Synthetized from **1ac** (47 mg, 0.1 mmol), **L₇**AuCl (6.0 mg, 0.005 mmol), $AgSbF_6$ (1.7 mg, 0.005 mmol) and 1 mL of Ph-Cl. Purification by Biotage® Selekt (Hex/AcOEt 95:5 → 60:40) gave **2ac** (40 mg, 86% yield) as a white solid.

^{1}H NMR (400 MHz, $CDCl_3$): 7.60 (dt, *J* = 7.9, 1.0 Hz, 1H), 7.48 – 7.36 (m, 3H), 7.35 – 7.30 (m, 2H), 7.27 – 7.14 (m, 3H), 7.06 (m, 1H), 6.95 (m, 1H), 6.86 – 6.66 (m, 5H), 6.58 (dt, *J* = 8.3, 0.9 Hz, 1H), 6.55 (s, 1H), 5.55 (d, *J* = 14.6 Hz, 1H), 4.38 (d, *J* = 14.6 Hz, 1H), 3.80 (s, 3H), 1.99 (s, 3H).

^{13}C NMR (101 MHz, $CDCl_3$): 168.64 (C), 160.16 (C), 139.96 (C), 137.46 (C), 135.98 (C), 135.83 (C), 135.42 (C), 134.55 (C), 134.07 (CH), 133.91 (CH), 131.38 (C), 128.98 (CH), 128.96 (C), 128.88 (CH), 128.59 (CH), 128.47 (CH), 128.44 (CH), 128.30 (CH), 128.10 (CH), 127.96 (CH), 127.51 (CH), 127.18 (CH), 123.13 (CH), 122.80 (CH), 120.17 (CH), 118.69 (CH), 113.88 (CH), 112.64 (C), 110.93 (CH), 55.32 (CH_3), 52.44 (CH_2), 9.63 (CH_3).

HRMS (ESI) calculated for $C_{32}H_{26}N_2O_2Na$ $[M+Na]^+$ requires m/z = 493.1892, found m/z 493.1890.

$[\alpha]^D_{25}$ = +239.9 (c = 0.25, $CHCl_3$).

R_t (OD-H column, Hex/*i*-PrOH 80:20, 0.8 mL/min, 266.4 nm): tr(major) = 11.2 min, tr(minor) = 8.1 min, 2:98 e.r.

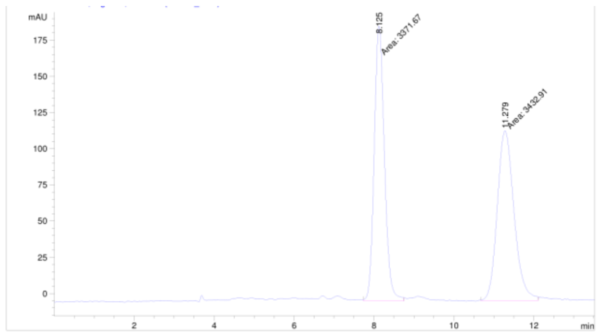

Peak #	RetTime [min]	Type	Width [min]	Area [mAU*s]	Height [mAU]	Area %
1	8.125	MM	0.2958	3371.66943	189.97729	49.5500
2	11.279	MM	0.4868	3432.90820	117.54296	50.4500

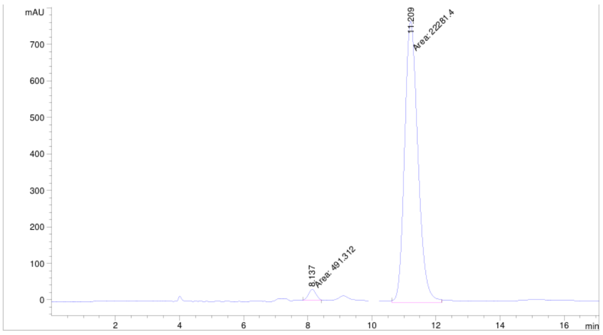

Peak #	RetTime [min]	Type	Width [min]	Area [mAU*s]	Height [mAU]	Area %
1	8.137	MM	0.2732	491.31247	29.96806	2.1575
2	11.209	MM	0.4818	2.22814e4	770.78455	97.8425

(*S,Z*)-5-benzyl-8-(4-fluorophenyl)-9-methylbenzo[2,3][1,4]diazocino[1,8-*a*]indol-6(5*H*)-one (2ad)

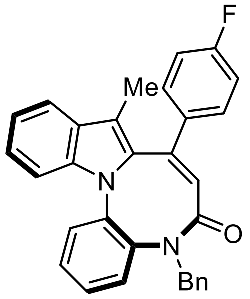

Synthetized from **1ad** (46 mg, 0.1 mmol), **L_7**AuCl (6.0 mg, 0.005 mmol), $AgSbF_6$ (1.7 mg, 0.005 mmol) and 1 mL of Ph-Cl. Purification by flash column chromatography (SiO_2, Hex/AcOEt 7:1 → 5:1) gave **2ad** (35 mg, 76% yield) as a white solid.

^{1}H NMR (600 MHz, $CDCl_3$): 7.48 (d, *J* = 7.9 Hz, 1H), 7.38 – 7.28 (m, 2H), 7.26 – 7.18 (m, 2H), 7.18 – 7.11 (m, 2H), 7.07 (m, 1H), 6.95 (ddd, *J* = 8.2, 7.0, 1.2 Hz, 1H), 6.90 – 6.81 (m, 3H), 6.65 (t, *J* = 7.7 Hz, 2H), 6.59 (dd, *J* = 7.8, 1.4 Hz, 2H), 6.48 – 6.44 (m, 2H), 5.45 (d, *J* = 14.6 Hz, 1H), 4.27 (d, *J* = 14.6 Hz, 1H), 1.85 (s, 3H).

^{13}C NMR (151 MHz, $CDCl_3$): 168.18 (C),163.09 (d, *J* = 248.6 Hz, C), 139.80 (C), 137.56 (C), 135.94 (C), 135.29 (C), 135.10 (d, *J* = 3.3 Hz, C), 134.23 (C), 129.06 (d, *J* = 8.2 Hz, CH), 128.92 (C), 128.60 (CH), 128.58 (CH), 128.31 (CH), 128.14 (CH), 127.99 (CH), 127.58 (CH), 127.26 (CH), 124.71 (CH), 123.04 (CH), 120.33 (CH), 118.76 (CH),115.51 (d, *J* = 21.6 Hz, CH), 112.86 (C), 111.01 (CH), 52.46 (CH_2), 9.60 (CH_3). One C is missing, probably overlapped.

HRMS (ESI) calculated for $C_{31}H_{23}N_2OFNa$ $[M+Na]^+$ requires m/z = 481.1692, found m/z 481.1693.

$[\alpha]^D_{25}$ = +273.6 (c = 0.25, $CHCl_3$).

R_t (OD-H column, Hex/*i*-PrOH 80:20, 0.8 mL/min, 266.4 nm): tr(major) = 9.4 min, tr(minor) = 6.3 min, 2:98 e.r.

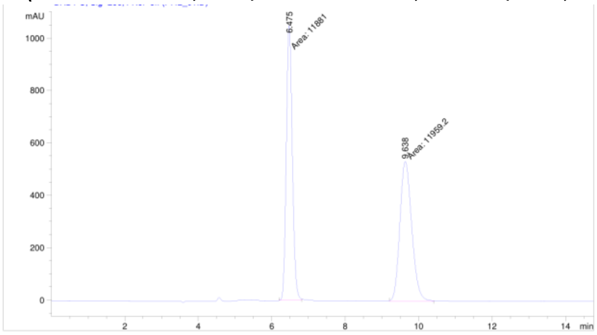

Peak #	RetTime [min]	Type	Width [min]	Area [mAU*s]	Height [mAU]	Area %
1	6.475	MM	0.1885	1.18810e4	1050.40710	49.8361
2	9.638	MM	0.3733	1.19592e4	533.88208	50.1639

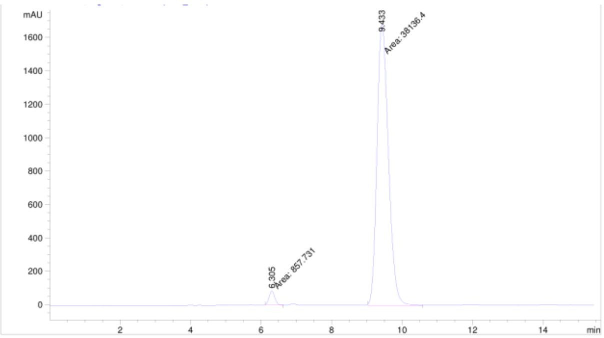

Peak #	RetTime [min]	Type	Width [min]	Area [mAU*s]	Height [mAU]	Area %
1	6.305	MM	0.1765	857.73138	80.98854	2.1996
2	9.433	MM	0.3767	3.81364e4	1687.15894	97.8004

(S,Z)-5-benzyl-8-(4-bromophenyl)-9-methylbenzo[2,3][1,4]diazocino[1,8-*a*]indol-6(5*H*)-one (2ae)

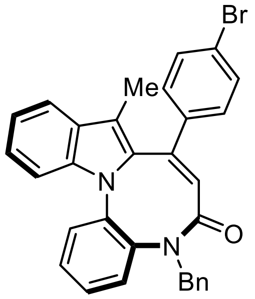

Synthetized from **1ae** (52 mg, 0.1 mmol), **L₇**AuCl (6.0 mg, 0.005 mmol), $AgSbF_6$ (1.7 mg, 0.005 mmol) and 1 mL of Ph-Cl. Purification by Biotage® Selekt (Hex/AcOEt 97:3 → 70:30) gave **2ae** (45 mg, 86% yield) as a white solid.

^{1}H NMR (300 MHz, $CDCl_3$): 7.57 (dt, *J* = 7.9, 1.0 Hz, 1H), 7.44 – 7.34 (m, 4H), 7.33 – 7.20 (m, 2H), 7.21 – 7.09 (m, 3H), 7.04 (ddd, *J* = 8.2, 7.0, 1.2 Hz, 1H), 6.93 (t, *J* = 7.2 Hz, 1H), 6.78 – 6.63 (m, 4H), 6.59 (d, *J* = 0.7 Hz, 1H), 6.54 (dt, *J* = 8.2, 0.9 Hz, 1H), 5.53 (d, *J* = 14.5 Hz, 1H), 4.35 (d, *J* = 14.5 Hz, 1H), 1.94 (s, 3H).

^{13}C NMR (75 MHz, $CDCl_3$): 168.00 (C), 139.65 (C), 137.86 (C), 137.54 (C), 135.84 (C), 135.21 (C), 135.20 (C), 133.85 (C), 131.68 (CH), 128.84 (C), 128.80 (CH), 128.62 (CH), 128.29 (CH), 128.12 (CH), 128.12 (CH), 127.96 (CH), 127.58 (CH), 127.26 (CH), 125.24 (CH), 123.07 (CH), 122.97 (C), 120.33 (CH), 118.77 (CH), 112.91 (C), 110.99 (CH), 52.43 (CH_2), 9.67 (CH_3).

HRMS (ESI) calculated for $C_{31}H_{23}N_2ONaBr$ $[M+Na]^+$ requires m/z = 541.0891, found m/z 541.0898.

$[\alpha]^D_{25}$ = +270.2 (c = 0.25, $CHCl_3$).

R_t (OD-H column, Hex/*i*-PrOH 80:20, 0.8 mL/min, 266.4 nm): tr(major) = 13.2 min, tr(minor) = 7.5 min, 2:98 e.r.

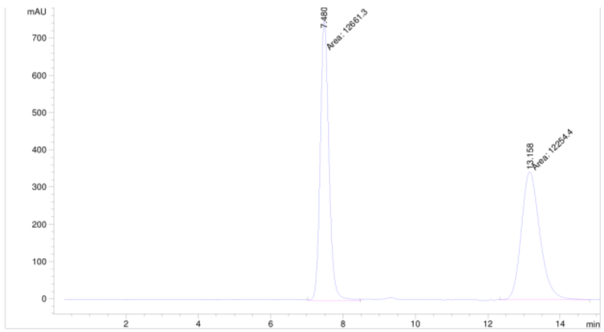

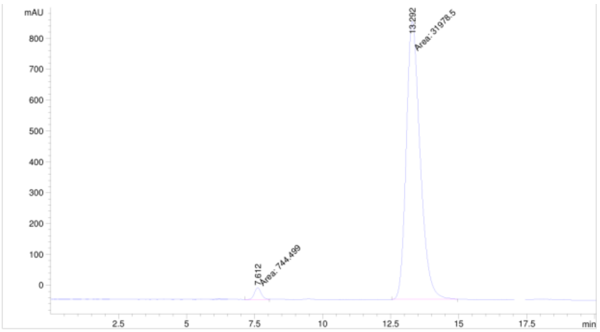

Peak #	RetTime [min]	Type	Width [min]	Area [mAU*s]	Height [mAU]	Area %
1	7.480	MM	0.2802	1.26613e4	753.07410	50.8166
2	13.158	MM	0.5948	1.22544e4	343.37863	49.1834

Peak #	RetTime [min]	Type	Width [min]	Area [mAU*s]	Height [mAU]	Area %
1	7.612	MM	0.3140	744.49933	39.51443	2.2752
2	13.292	MM	0.5920	3.19785e4	900.28973	97.7248

(*S,Z*)-5-benzyl-9-methyl-8-(4-(trifluoromethyl)phenyl)benzo[2,3][1,4]diazocino[1,8-*a*]indol-6(5*H*)-one (2af)

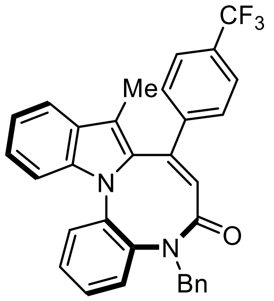

Synthetized from **1af** (51 mg, 0.1 mmol), **L₇**AuCl (6.0 mg, 0.005 mmol), $AgSbF_6$ (1.7 mg, 0.005 mmol) and 1 mL of Ph-Cl. Purification by Biotage® Selekt (Hex/AcOEt 92:8 → 70:30) gave **2af** (30 mg, 59% yield) as a white solid.

^{1}H NMR (300 MHz, $CDCl_3$): 7.60 – 7.48 (m, 3H), 7.47 – 7.28 (m, 6H), 7.17 (ddd, *J* = 8.0, 7.0, 1.0 Hz, 1H), 7.05 (ddd, *J* = 8.3, 7.0, 1.2 Hz, 1H), 6.97 – 6.90 (m, 1H), 6.78 – 6.64 (m, 5H), 6.54 (dt, *J* = 8.2, 1.0 Hz, 1H), 5.55 (d, *J* = 14.5 Hz, 1H), 4.36 (d, *J* = 14.5 Hz, 1H), 1.93 (d, *J* = 0.6 Hz, 3H).

^{13}C NMR (75 MHz, $CDCl_3$) : 167.72 (C), 142.46 (C), 139.54 (C), 137.61 (C), 135.83 (C), 135.12 (C), 135.02 (C), 133.70 (C), 130,57 (q, J = 32.7, C), 128.82 (C), 128.70 (CH), 128.31 (CH), 128.14 (CH), 127.98 (CH), 127.62 (CH), 127.52 (CH), 127.30 (CH), 126.73 (CH), 125.48 (q, J = 3.8, CH), 123.93 (q, J = 271.94, C) 123.19 (CH), 122.12 (CH), 120.41 (CH), 118.82 (CH), 113.02 (C), 111.03 (CH), 52.45 (CH_2), 9.64 (CH_3).

HRMS (ESI) calculated for $C_{32}H_{23}N_2OF_3Na$ $[M+Na]^+$ requires m/z = 531.1660, found m/z 531.1661.

[α]^D$_{25}$ = +272.3 (c = 0.25, $CHCl_3$).

R_t (OD-H column, Hex/*i*-PrOH 80:20, 0.8 mL/min, 266.4 nm): tr(major) = 9.8 min, tr(minor) = 5.9 min, 4:96 e.r.

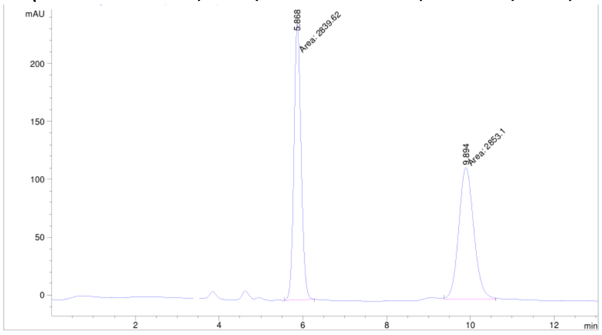

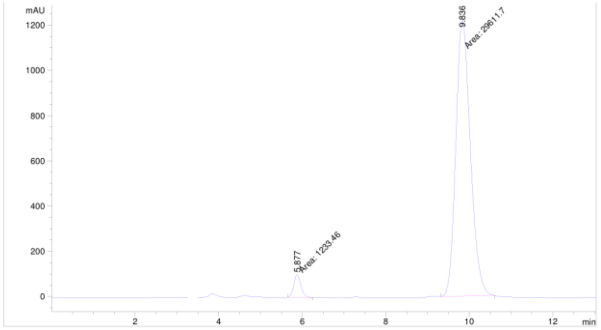

Peak #	RetTime [min]	Type	Width [min]	Area [mAU*s]	Height [mAU]	Area %
1	5.868	MM	0.1974	2839.61987	239.69127	49.8816
2	9.894	MM	0.4169	2853.10278	114.04664	50.1184

Peak #	RetTime [min]	Type	Width [min]	Area [mAU*s]	Height [mAU]	Area %
1	5.877	MM	0.2028	1233.45850	101.38798	3.9989
2	9.836	MM	0.4035	2.96117e4	1223.25952	96.0011

ethyl (*S,Z*)-4-(5-benzyl-9-methyl-6-oxo-5,6-dihydrobenzo[2,3][1,4]diazocino[1,8-*a*]indol-8-yl)benzoate (2ag)

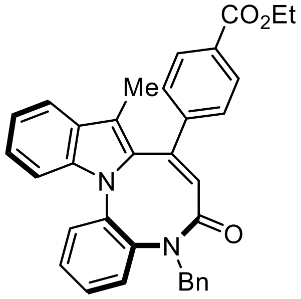

Synthetized from **1ag** (51 mg, 0.1 mmol), **L₇**AuCl (6.0 mg, 0.005 mmol), $AgSbF_6$ (1.7 mg, 0.005 mmol) and 1 mL of Ph-Cl. Purification by Biotage® Selekt (Hex/AcOEt 96:4 → 60:40) gave **2ag** (40 mg, 78% yield) as a white solid.

^{1}H NMR (400 MHz, $CDCl_3$): 8.00 – 7.92 (m, 2H), 7.59 (dt, *J* = 8.0, 1.0 Hz, 1H), 7.50 – 7.39 (m, 2H), 7.37 – 7.28 (m, 4H), 7.19 (ddd, *J* = 8.0, 7.1, 1.0 Hz, 1H), 7.07 (ddd, *J* = 8.2, 7.0, 1.2 Hz, 1H), 6.96 (td, *J* = 7.2, 1.4 Hz, 1H), 6.82 – 6.67 (m, 5H), 6.57 (dt, *J* = 8.2, 0.9 Hz, 1H), 5.56 (d, *J* = 14.5 Hz, 1H), 4.44 – 4.32 (m, 3H), 1.94 (s, 3H), 1.39 (t, *J* = 7.1 Hz, 3H).

^{13}C NMR (101 MHz, $CDCl_3$): 167.88 (C), 166.13 (C), 143.28 (C), 139.62 (C), 137.64 (C), 135.92 (C), 135.47 (C), 135.22 (C), 133.92 (C), 130.54 (C), 129.76 (CH), 128.90 (C), 128.68 (CH), 128.66 (CH), 128.39 (CH), 128.16 (CH), 128.00 (CH), 127.63 (CH), 127.30 (CH), 127.23 (CH), 126.53 (CH), 123.13 (CH), 120.37 (CH), 118.82 (CH), 113.02 (C), 111.04 (CH), 61.08 (CH_2), 52.48 (CH_2), 14.31 (CH_3), 9.62 (CH_3).

HRMS (ESI) calculated for $C_{34}H_{28}N_2O_3Na$ $[M+Na]^+$ requires m/z = 535.1998, found m/z 535.2001.

$[\alpha]^D_{25}$ = +328.7 (c = 0.225, $CHCl_3$).

R_t (OD-H column, Hex/*i*-PrOH 80:20, 0.8 mL/min, 266.4 nm): tr(major) = 11.3 min, tr(minor) = 7.8 min, 3:97 e.r.

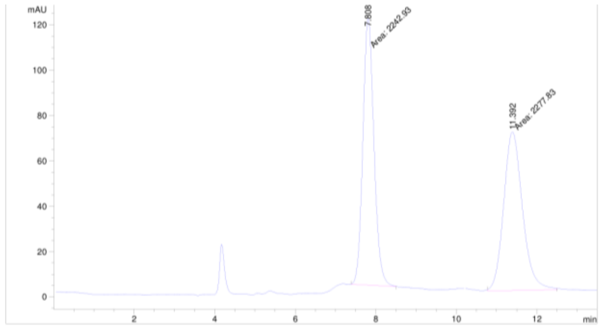

Peak #	RetTime [min]	Type	Width [min]	Area [mAU*s]	Height [mAU]	Area %
1	7.808	MM	0.3176	2242.93140	117.69518	49.6140
2	11.392	MM	0.5425	2277.82739	69.98175	50.3860

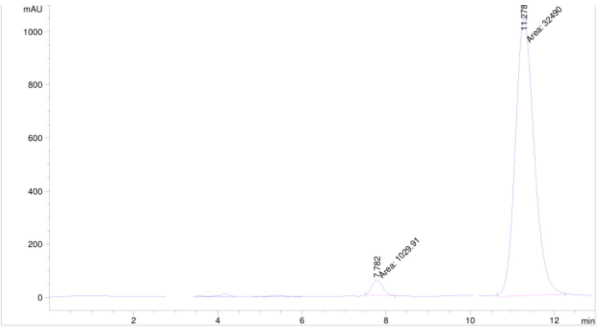

Peak #	RetTime [min]	Type	Width [min]	Area [mAU*s]	Height [mAU]	Area %
1	7.782	MM	0.2994	1029.91284	57.34080	3.0725
2	11.278	MM	0.5183	3.24900e4	1044.68909	96.9275

(*S,Z*)-5-benzyl-9-methyl-8-(m-tolyl)benzo[2,3][1,4]diazocino[1,8-*a*]indol-6(5*H*)-one (2ah)

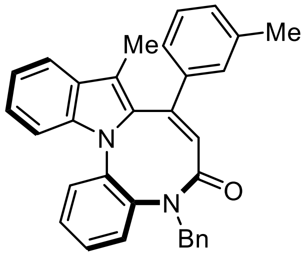

Synthetized from **1ah** (45 mg, 0.1 mmol), **L_7**AuCl (6.0 mg, 0.005 mmol), $AgSbF_6$ (1.7 mg, 0.005 mmol) and 1 mL of Ph-Cl. Purification by Biotage® Selekt (Hex/AcOEt 97:3 → 70:30) gave **2ah** (38 mg, 84% yield) as a white solid.

^{1}H NMR (300 MHz, $CDCl_3$): 7.58 (d, *J* = 7.8 Hz, 1H), 7.47 – 7.27 (m, 4H), 7.23 – 7.01 (m, 6H), 6.94 (t, *J* = 7.2 Hz, 1H), 6.72 (dt, *J* = 14.7, 7.3 Hz, 4H), 6.61 – 6.52 (m, 2H), 5.54 (d, *J* = 14.6 Hz, 1H), 4.36 (d, *J* = 14.6 Hz, 1H), 2.28 (s, 3H), 1.95 (s, 3H).

^{13}C NMR (75 MHz, $CDCl_3$): 168.42 (C), 139.84 (C), 138.94 (C), 138.18 (C), 137.50 (C), 136.37 (C), 135.98 (C), 135.34 (C), 134.55 (C), 129.46 (CH), 128.96 (C), 128.49 (CH), 128.39 (CH), 128.37 (CH), 128.10 (CH), 128.01 (CH), 127.96 (CH), 127.83 (CH), 127.49 (CH), 127.19 (CH), 124.81 (CH), 124.52 (CH), 122.79 (CH), 120.15 (CH), 118.71 (CH), 112.71 (C), 110.97 (CH), 52.41 (CH_2), 21.36 (CH_3), 9.62 (CH_3).

HRMS (ESI) calculated for $C_{32}H_{26}N_2ONa$ $[M+Na]^+$ requires m/z = 477.1943, found m/z 477.1945.

$[\alpha]^D_{25}$ = +294.4 (c = 0.25, $CHCl_3$).

R_t (OD-H column, Hex/*i*-PrOH 80:20, 0.8 mL/min, 266.4 nm): tr(major) = 7.4 min, tr(minor) = 5.8 min, 3:97 e.r.

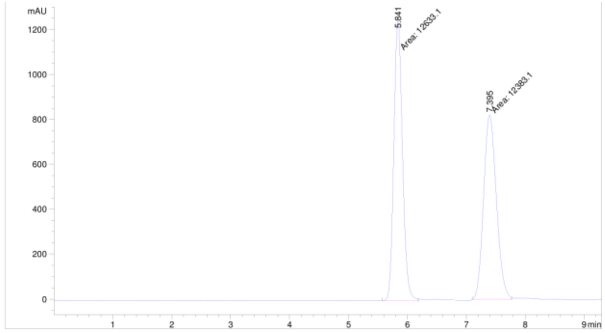

Peak #	RetTime [min]	Type	Width [min]	Area [mAU*s]	Height [mAU]	Area %
1	5.841	MM	0.1688	1.26331e4	1247.27844	50.4997
2	7.395	MM	0.2515	1.23831e4	820.70654	49.5003

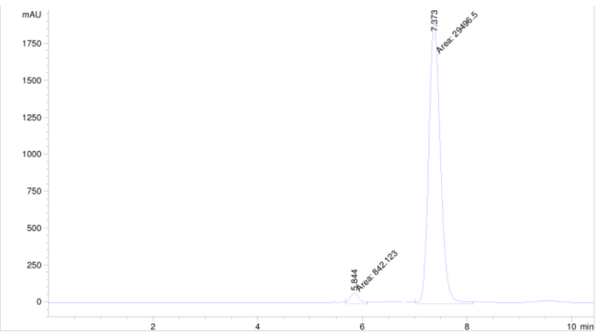

Peak #	RetTime [min]	Type	Width [min]	Area [mAU*s]	Height [mAU]	Area %
1	5.844	MM	0.1995	842.12317	70.36741	2.7757
2	7.373	MM	0.2594	2.94965e4	1895.53735	97.2243

(*S,Z*)-5-benzyl-9-methyl-8-(*o*-tolyl)benzo[2,3][1,4]diazocino[1,8-*a*]indol-6(5*H*)-one (2ai)

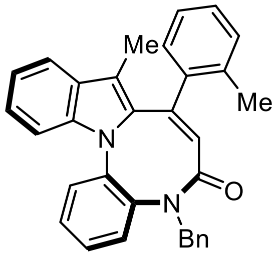

Synthetized from **1ai** (45 mg, 0.1 mmol), **L$_7$**AuCl (6.0 mg, 0.005 mmol), AgSbF$_6$ (1.7 mg, 0.005 mmol) and 1 mL of Ph-Cl. Purification by Biotage® Selekt (Hex/AcOEt 97:3 → 70:30) gave **2ai** (22 mg, 48% yield) as a white solid.

^{1}H NMR (400 MHz, CDCl$_3$): 7.55 – 7.47 (m, 2H), 7.46 – 7.40 (m, 2H), 7.36 (td, *J* = 7.6, 1.6 Hz, 1H), 7.27 (m, 1H), 7.22 (dd, *J* = 5.6, 3.4 Hz, 2H), 7.15 (ddd, *J* = 8.0, 7.0, 1.0 Hz, 1H), 7.05 (ddt, *J* = 7.0, 5.6, 1.2 Hz, 2H), 6.98 (ddd, *J* = 8.7, 4.4, 1.8 Hz, 1H), 6.85 – 6.73 (m, 4H), 6.55 (dt, *J* = 8.2, 0.9 Hz, 1H), 6.38 (d, *J* = 0.7 Hz, 1H), 5.61 (d, *J* = 14.6 Hz, 1H), 4.42 (d, *J* = 14.6 Hz, 1H), 1.84 (s, 3H), 1.74 (s, 3H).

^{13}C NMR (101 MHz, CDCl$_3$): 167.99 (C), 139.94 (C), 139.49 (C), 137.67 (C), 137.10 (C), 136.61 (C), 136.37 (C), 135.39 (C), 134.42 (C), 130.47 (CH), 130.19 (CH), 129.30 (C), 128.85 (CH), 128.44 (CH), 128.42 (CH), 128.28 (CH), 128.19 (CH), 128.07 (CH), 127.62 (CH), 127.55 (CH), 127.21 (CH), 126.04 (CH), 122.90 (CH), 120.31 (CH), 118.73 (CH), 112.97 (C), 111.33 (CH), 52.48 (CH$_2$), 20.43 (CH$_3$), 8.91 (CH$_3$).

HRMS (ESI) calculated for $C_{32}H_{26}N_2ONa$ [M+Na]$^+$ requires m/z = 477.1943, found m/z 477.1938.

[α]^D$_{25}$ = +412.5 (c = 0.25, CHCl$_3$).

R$_t$ (OD-H column, Hex/*i*-PrOH 80:20, 0.8 mL/min, 266.4 nm): tr(major) = 10.8 min, tr(minor) = 7.8 min, 9:91 e.r.

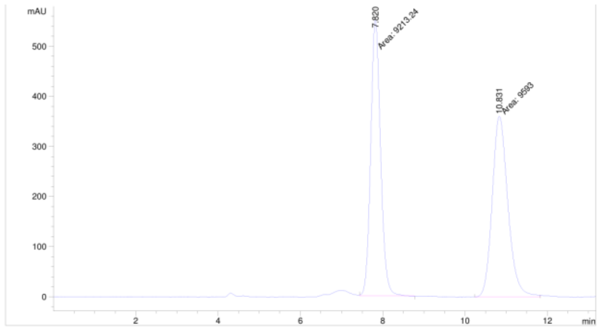

Peak #	RetTime [min]	Type	Width [min]	Area [mAU*s]	Height [mAU]	Area %
1	7.820	MM	0.2789	9213.24316	550.63885	48.9903
2	10.831	MM	0.4431	9593.00391	360.79956	51.0097

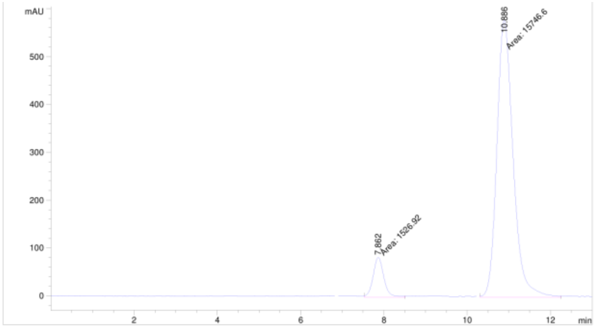

Peak #	RetTime [min]	Type	Width [min]	Area [mAU*s]	Height [mAU]	Area %
1	7.862	MM	0.3036	1526.92395	83.81598	8.8397
2	10.886	MM	0.4530	1.57466e4	579.30188	91.1603

(*S,Z*)-5-benzyl-9-methyl-8-(naphthalen-2-yl)benzo[2,3][1,4]diazocino[1,8-*a*]indol-6(5*H*)-one ((*S*)-2aj)

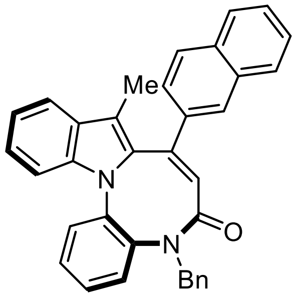

Synthetized from **1aj** (49 mg, 0.1 mmol), **L_7**AuCl (6.0 mg, 0.005 mmol), $AgSbF_6$ (1.7 mg, 0.005 mmol) and 1 mL of Ph-Cl. Purification by Biotage® Selekt (Hex/AcOEt 95:5 → 60:40) gave **2aj** (38 mg, 78% yield) as a white solid.

^{1}H NMR (400 MHz, $CDCl_3$): 7.83 – 7.73 (m, 4H), 7.63 (dt, *J* = 7.9, 1.0 Hz, 1H), 7.48 (m, 3H), 7.43 – 7.38 (m, 3H), 7.35 (m, 1H), 7.21 (ddd, *J* = 7.9, 7.1, 1.0 Hz, 1H), 7.10 (ddd, *J* = 8.2, 7.0, 1.2 Hz, 1H), 6.97 (m, 1H), 6.86 – 6.70 (m, 5H), 6.62 (dt, *J* = 8.3, 0.9 Hz, 1H), 5.60 (d, *J* = 14.5 Hz, 1H), 4.41 (d, *J* = 14.5 Hz, 1H), 1.98 (s, 3H).

^{13}C NMR (101 MHz, $CDCl_3$): 168.40 (C), 139.86 (C), 137.61 (C), 136.28 (C), 136.19 (C), 135.99 (C), 135.36 (C), 134.42 (C), 133.35 (C), 133.24 (C), 128.99 (C), 128.58 (CH), 128.43 (CH), 128.21 (CH), 128.15 (CH), 128.01 (CH), 127.58 (CH), 127.56 (CH), 127.26 (CH), 126.92 (CH), 126.59 (CH), 126.44 (CH), 125.35 (CH), 124.72 (CH), 122.94 (CH), 120.25 (CH), 118.83 (CH), 112.94 (C), 111.03 (CH), 52.49 (CH2), 9.68 (CH3). 2 CH are missing, probably overlapped.

HRMS (ESI) calculated for $C_{35}H_{26}N_2ONa$ $[M+Na]^+$ requires m/z = 513.1943, found m/z 513.1942.

$[\alpha]^D_{25}$ = +308.9 (c = 0.25, $CHCl_3$).

R_t (OD-H column, Hex/*i*-PrOH 80:20, 0.8 mL/min, 310 nm): tr(major) = 12.5 min, tr(minor) = 9.2 min, 2:98 e.r.

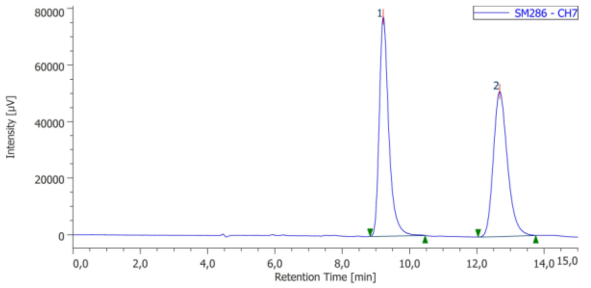

#	Peak Name	CH	tR [min]	Area [µV·sec]	Height [µV]	Area%
1	Unknown	7	9,217	1526137	77492	50,575
2	Unknown	7	12,677	1491419	51324	49,425

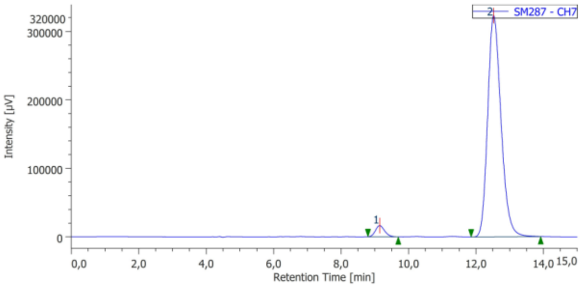

#	Peak Name	CH	tR [min]	Area [µV·sec]	Height [µV]	Area%
1	Unknown	7	9,140	312795	16392	3,401
2	Unknown	7	12,520	8885295	322838	96,599

(*R,Z*)-5-benzyl-9-methyl-8-(naphthalen-2-yl)benzo[2,3][1,4]diazocino[1,8-*a*]indol-6(5*H*)-one ((*R*)-2aj)

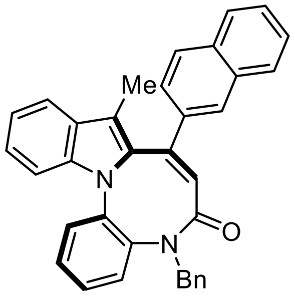

Synthetized from **1aj** following the general procedure, using the same catalyst but with opposite configuration.

$[\alpha]^D_{25}$ = -298.3 (c = 0.152, $CHCl_3$).

R_t (OD-H column, Hex/*i*-PrOH 80/20, 0.8 mL/min, 310 nm): tr(major) = 13.1 min, tr(minor) = 9.4 min, 98:2 e.r.

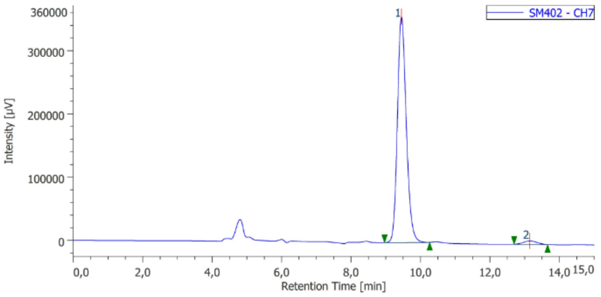

#	Peak Name	CH	tR [min]	Area [µV·sec]	Height [µV]	Area%
1	Unknown	7	9,447	6346985	357070	97,702
2	Unknown	7	13,140	149312	5544	2,298

(*S,E*)-5-benzyl-9-methyl-8-(thiophen-2-yl)benzo[2,3][1,4]diazocino[1,8-*a*]indol-6(5*H*)-one (2ak)

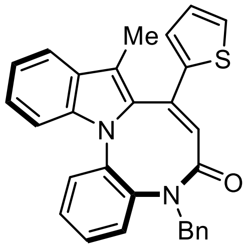

Synthetized from **1ak** (45 mg, 0.1 mmol), **L**$_7$AuCl (6.0 mg, 0.005 mmol), AgSbF$_6$ (1.7 mg, 0.005 mmol) and 1 mL of Ph-Cl. Purification by Biotage® Selekt (Hex/AcOEt 92:8 → 88:12) gave (*S*)-**2ak** (26 mg, 57% yield) as a white solid.

^{1}H NMR (300 MHz, CDCl$_3$): 7.63 (m, 1H), 7.55 (dd, *J* = 7.9, 1.6 Hz, 1H), 7.44 (td, *J* = 7.7, 1.5 Hz, 1H), 7.39 – 7.03 (m, 12H), 6.96 (dd, *J* = 5.1, 3.8 Hz, 1H), 6.76 (s, 1H), 6.34 (s, 1H), 5.40 (d, *J* = 14.4 Hz, 1H), 3.49 (d, *J* = 14.4 Hz, 1H), 2.36 (d, *J* = 1.1 Hz, 3H).

^{13}C NMR (75 MHz, CDCl$_3$): 164.06 (C), 141.08 (C), 136.65 (C), 136.54 (C), 136.39 (C), 136.15 (C), 135.46 (C), 131.49 (CH), 129.37 (C), 129.11 (C), 129.07 (CH), 128.57 (CH), 128.45 (CH), 128.35 (CH), 128.12 (CH), 127.83 (CH), 127.60 (CH), 127.49 (CH), 125.11 (CH), 122.68 (CH), 120.13 (CH), 119.31 (CH), 115.52 (CH), 113.77 (C), 109.74 (CH), 50.85 (CH$_2$), 9.73 (CH$_3$).

HRMS (ESI) calculated for $C_{29}H_{22}N_2ONaS$ [M+Na]$^+$ requires m/z = 469.1351, found m/z 469.1357.

[α]^D$_{25}$ = +112.8 (c = 0.25, CHCl$_3$).

R$_t$ (OD-H column, Hex/*i*-PrOH 80/20, 0.8 mL/min, 310 nm): tr(major) = 11.3 min, tr(minor) = 9.0 min, 2:98 e.r.

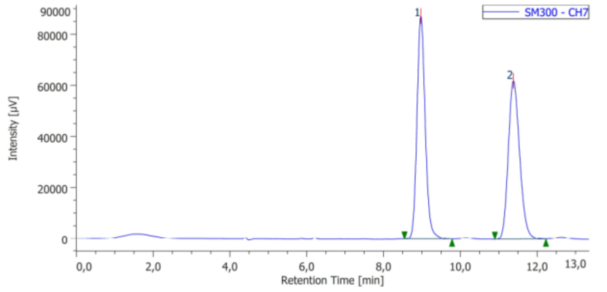

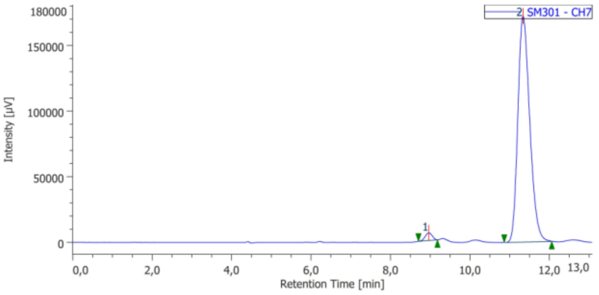

#	Peak Name	CH	tR [min]	Area [µV·sec]	Height [µV]	Area%
1	Unknown	7	8,970	1310834	87316	50,513
2	Unknown	7	11,380	1284207	62110	49,487

#	Peak Name	CH	tR [min]	Area [µV·sec]	Height [µV]	Area%
1	Unknown	7	8,963	71777	5816	1,971
2	Unknown	7	11,333	3569776	172091	98,029

(*S,Z*)-5-benzyl-8,9-dimethylbenzo[2,3][1,4]diazocino[1,8-*a*]indol-6(5*H*)-one (2al)

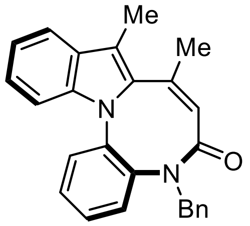

Synthetized from **1al** (38 mg, 0.1 mmol), L_7AuCl (6.0 mg, 0.005 mmol), $AgSbF_6$ (1.7 mg, 0.005 mmol) and 1 mL of Ph-Cl. Purification by Biotage® Selekt (Hex/AcOEt 93:7 → 70:30) gave **2al** (22 mg, 58% yield) as a white solid.

^{1}H NMR (300 MHz, $CDCl_3$): 7.57 (d, *J* = 7.9 Hz, 1H), 7.44 – 7.37 (m, 2H), 7.33 (m, 1H), 7.24 (m, 1H), 7.14 (m, 1H), 6.99 (ddd, *J* = 8.2, 7.0, 1.2 Hz, 1H), 6.89 (t, *J* = 7.4 Hz, 1H), 6.69 (t, *J* = 7.6 Hz, 2H), 6.61 (d, *J* = 7.5 Hz, 2H), 6.51 (d, *J* = 8.2 Hz, 1H), 6.21 (d, *J* = 1.9 Hz, 1H), 5.47 (d, *J* = 14.6 Hz, 1H), 4.28 (d, *J* = 14.6 Hz, 1H), 2.40 (s, 3H), 2.04 (d, *J* = 1.5 Hz, 3H).

^{13}C NMR (75 MHz, $CDCl_3$): 168.08 (C), 139.93 (C), 136.97 (C), 135.99 (C), 135.39 (C), 134.10 (C), 129.03 (C), 128.27 (CH), 128.19 (CH), 128.01 (CH), 127.84 (CH), 127.53 (CH), 127.07 (CH), 126.15 (CH), 122.58 (CH), 120.11 (CH), 118.37 (CH), 110.78 (CH), 110.34 (C), 52.24 (CH_2), 23.59 (CH_3), 9.59 (CH_3). One C and one CH are missing, probably overlapped.

HRMS (ESI) calculated for $C_{26}H_{22}N_2ONa$ $[M+Na]^+$ requires m/z = 401.1630, found m/z 401.1636.

$[\alpha]^D_{25}$ = +200.7 (c = 0.5, $CHCl_3$).

R_t (OD-H column, Hex/*i*-PrOH 80/20, 0.8 mL/min, 266.4 nm): tr(major) = 7.7 min, tr(minor) = 6.3 min, 5:95 e.r.

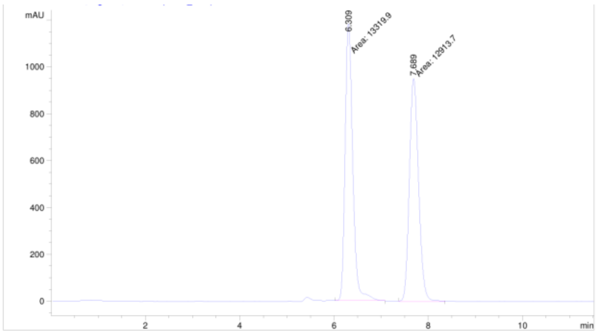

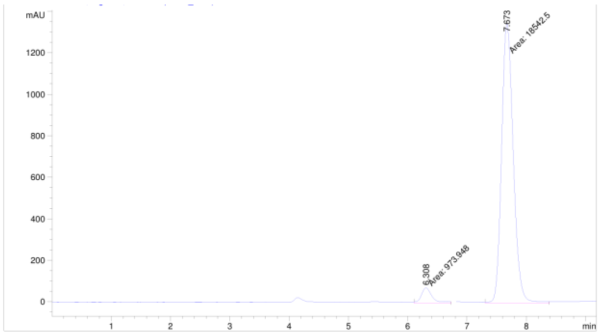

Peak #	RetTime [min]	Type	Width [min]	Area [mAU*s]	Height [mAU]	Area %
1	6.309	MM	0.1876	1.33199e4	1183.33813	50.7741
2	7.689	MM	0.2258	1.29137e4	953.30365	49.2259

Peak #	RetTime [min]	Type	Width [min]	Area [mAU*s]	Height [mAU]	Area %
1	6.308	MM	0.2184	973.94788	74.32731	4.9904
2	7.673	MM	0.2293	1.85425e4	1347.79138	95.0096

(*S,Z*)-5-benzyl-9-methylbenzo[2,3][1,4]diazocino[1,8-*a*]indol-6(5*H*)-one (2am)

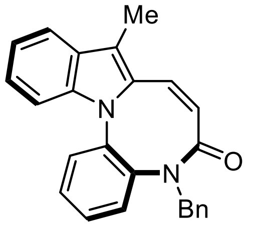

Synthetized from **1am** (36 mg, 0.1 mmol), **L₇**AuCl (6.0 mg, 0.005 mmol), $AgSbF_6$ (1.7 mg, 0.005 mmol) and 1 mL of Ph-Cl. Purification by Biotage® Selekt (Hex/AcOEt 90:10 → 60:40) gave **2am** (30 mg, 83% yield) as a white solid.

^{1}H NMR (300 MHz, $CDCl_3$): 7.56 (d, *J* = 7.9 Hz, 1H), 7.47 – 7.30 (m, 2H), 7.30 – 7.20 (m, 2H), 7.20 – 7.10 (m, 1H), 7.09 – 6.96 (m, 3H), 6.90 – 6.76 (m, 4H), 6.66 (d, *J* = 12.5 Hz, 1H), 6.52 (d, *J* = 8.2 Hz, 1H), 6.25 (d, *J* = 12.5 Hz, 1H), 5.58 (d, *J* = 14.5 Hz, 1H), 4.40 (d, *J* = 14.5 Hz, 1H), 2.34 (s, 3H).

^{13}C NMR (75 MHz, $CDCl_3$): 167.78 (C), 138.92 (C), 138.83 (C), 137.36 (C), 135.34 (C), 133.97 (C), 129.51 (C), 128.52 (CH), 128.27 (CH), 128.24 (CH), 128.11 (CH), 128.05 (CH), 127.90 (CH), 127.19 (CH), 127.02 (CH), 123.22 (CH), 123.02 (CH), 120.64 (CH), 118.78 (CH), 113.74 (C), 111.90 (CH), 52.63 (CH_2), 9.21 (CH_3).

HRMS (ESI) calculated for $C_{25}H_{20}N_2ONa$ $[M+Na]^+$ requires m/z = 387.1468, found m/z 387.1475.

$[\alpha]^D_{25}$ = +397.9 (c = 0.25, $CHCl_3$).

R_t (OD-H column, Hex/*i*-PrOH 80/20, 0.8 mL/min, 266.4 nm): tr(major) = 9.7 min, tr(minor) = 7.8 min, 11:89 e.r.

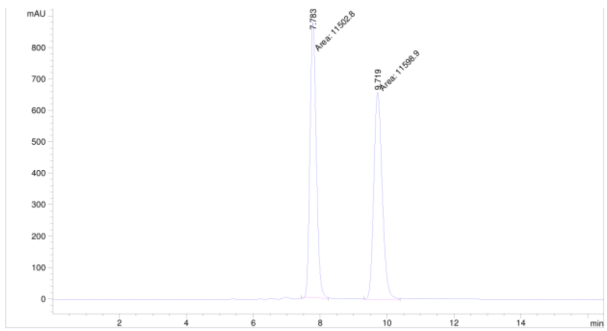

Peak #	RetTime [min]	Type	Width [min]	Area [mAU*s]	Height [mAU]	Area %
1	7.783	MM	0.2186	1.15028e4	877.11920	49.7919
2	9.719	MM	0.2933	1.15989e4	659.04687	50.2081

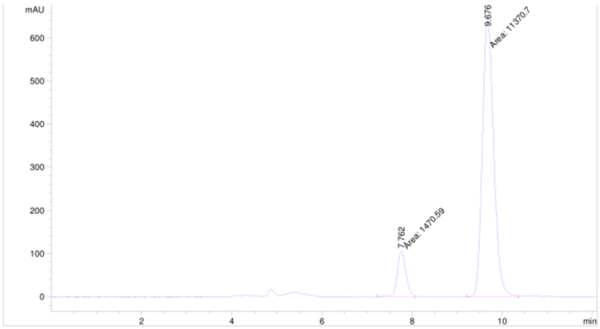

Peak #	RetTime [min]	Type	Width [min]	Area [mAU*s]	Height [mAU]	Area %
1	7.762	MM	0.2277	1470.58972	107.63534	11.4520
2	9.676	MM	0.2928	1.13707e4	647.18317	88.5480

(*S,Z*)-5-benzyl-9-ethyl-8-phenylbenzo[2,3][1,4]diazocino[1,8-*a*]indol-6(5*H*)-one (2an)

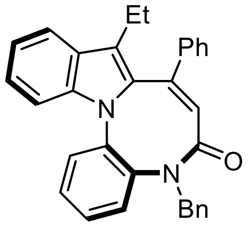

Synthetized from **1an** (45 mg, 0.1 mmol), **L_7**AuCl (6.0 mg, 0.005 mmol), $AgSbF_6$ (1.7 mg, 0.005 mmol) and 1 mL of Ph-Cl. Purification by flash column chromatography (SiO_2, Hex/AcOEt 5:1) gave **2an** (36 mg, 81% yield) as a white solid.

^{1}H NMR (400 MHz, $CDCl_3$): 7.64 (d, *J* = 7.9 Hz, 1H), 7.41 (m, 4H), 7.30 – 7.26 (m, 5H), 7.16 (t, *J* = 7.7 Hz, 1H), 7.04 (t, *J* = 7.9 Hz, 1H), 6.90 (t, *J* = 7.3 Hz, 1H), 6.76 – 6.58 (m, 6H), 5.58 (d, *J* = 14.6 Hz, 1H), 4.32 (d, *J* = 14.6 Hz, 1H), 2.50 (ddd, *J* = 14.4, 7.4, 2.2 Hz, 1H), 2.37 (ddd, *J* = 14.4, 7.4, 2.2 Hz, 1H), 1.08 (td, *J* = 7.5, 2.2 Hz, 3H).

^{13}C NMR (101 MHz, $CDCl_3$): 168.34 (C), 139.99 (C), 139.35 (C), 137.48 (C), 136.62 (C), 135.72 (C), 135.37 (C), 133.73 (C), 128.71 (CH), 128.59 (CH), 128.56 (CH), 128.40 (CH), 128.33 (CH), 128.00 (CH), 127.91 (C), 127.76 (CH), 127.26 (CH), 127.16 (CH), 125.40 (CH), 122.74 (CH), 120.07 (CH), 118.99 (CH), 118.92 (C), 110.83 (CH), 52.57 (CH_2), 18.13 (CH_2), 14.76 (CH_3). One CH is missing, probably overlapped.

HRMS (ESI) calculated for $C_{32}H_{26}N_2ONa$ $[M+Na]^+$ requires m/z = 477.1943, found m/z 477.1942.

$[\alpha]^D_{25}$ = +166.1 (c = 0.25, $CHCl_3$).

R_t (OD-H column, Hex/*i*-PrOH 80/20, 0.8 mL/min, 266.4 nm): tr(major) = 7.1 min, tr(minor) = 6.5 min, 4:96 e.r.

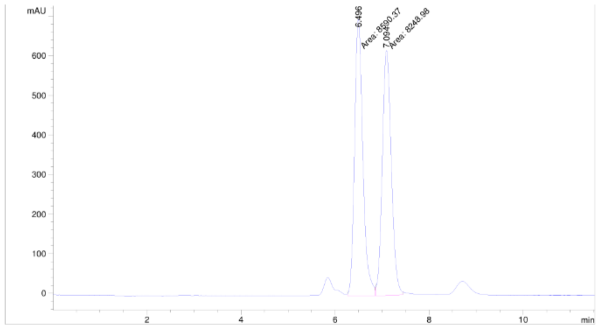

Peak #	RetTime [min]	Type	Width [min]	Area [mAU*s]	Height [mAU]	Area %
1	6.496	MM	0.2047	8590.37402	699.39581	51.0137
2	7.094	MM	0.2221	8248.97852	619.11664	48.9863

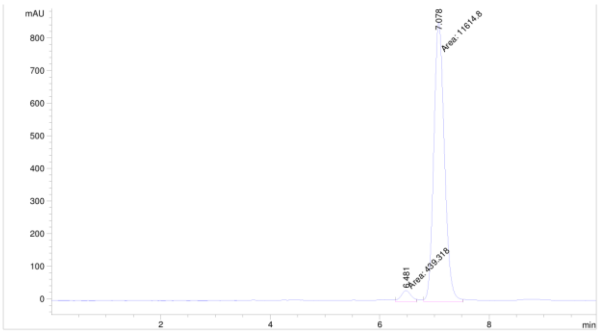

Peak #	RetTime [min]	Type	Width [min]	Area [mAU*s]	Height [mAU]	Area %
1	6.481	MM	0.2144	439.31818	34.15166	3.6446
2	7.078	MM	0.2261	1.16148e4	856.11853	96.3554

(*S,Z*)-5-benzyl-9-isopropyl-8-phenylbenzo[2,3][1,4]diazocino[1,8-*a*]indol-6(5*H*)-one (2ao)

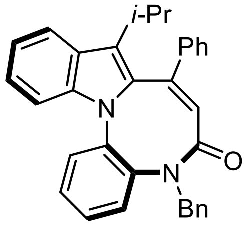

Synthetized from **1ao** (47 mg, 0.1 mmol), **L_7**AuCl (6.0 mg, 0.005 mmol), $AgSbF_6$ (1.7 mg, 0.005 mmol) and 1 mL of Ph-Cl. Purification by Biotage® Selekt (Hex/AcOEt 90:10 → 60:40) gave **2ao** (18 mg, 38% yield) as a white solid.

^{1}H NMR (300 MHz, $CDCl_3$): 7.74 (d, *J* = 8.0 Hz, 1H), 7.57 – 7.32 (m, 5H), 7.26 (s, 4H), 7.09 (t, *J* = 7.5 Hz, 1H), 6.96 (t, *J* = 7.6 Hz, 1H), 6.82 (t, *J* = 7.3 Hz, 1H), 6.69 – 6.52 (m, 6H), 5.55 (d, *J* = 14.6 Hz, 1H), 4.22 (d, *J* = 14.6 Hz, 1H), 2.77 (p, *J* = 7.2 Hz, 1H), 1.30 (d, *J* = 7.2 Hz, 3H), 1.12 (d, *J* = 7.0 Hz, 3H).

^{13}C NMR (75 MHz, $CDCl_3$): 168.37 (C), 140.10 (C), 139.54 (C), 137.75 (C), 136.86 (C), 135.51 (C), 135.34 (C), 132.67 (C), 128.66 (CH), 128.56 (CH), 128.53 (CH), 128.31 (CH), 127.85 (CH), 127.81 (CH), 127.52 (CH), 127.07 (CH), 127.01 (CH), 126.50 (C), 125.58 (CH), 122.42 (CH), 122.37 (C), 120.48 (CH), 119.68 (CH), 110.77 (CH), 52.58 (CH_2), 26.68 (CH), 23.40 (CH_3), 21.29 (CH_3). One CH is missing, probably overlapped.

HRMS (ESI) calculated for $C_{33}H_{28}N_2ONa$ $[M+Na]^+$ requires m/z = 491.2099, found m/z 491.2098.

[α]$^D_{25}$ = +109.1 (c = 0.165, $CHCl_3$).

R_t (OD-H column, Hex/*i*-PrOH 80/20, 0.8 mL/min, 266.4 nm): tr(major) = 6.7 min, tr(minor) = 6.4 min, 6:94 e.r.

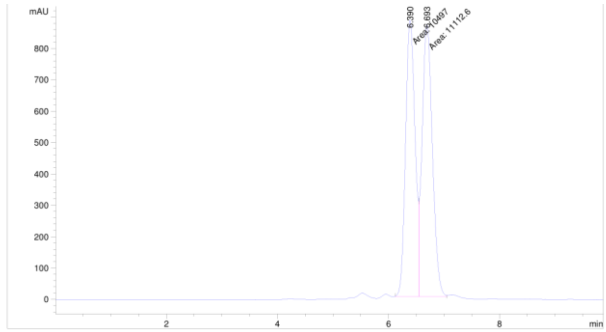

Peak #	RetTime [min]	Type	Width [min]	Area [mAU*s]	Height [mAU]	Area %
1	6.390	MF	0.1985	1.04970e4	881.34497	48.5758
2	6.693	FM	0.2135	1.11126e4	867.49384	51.4242

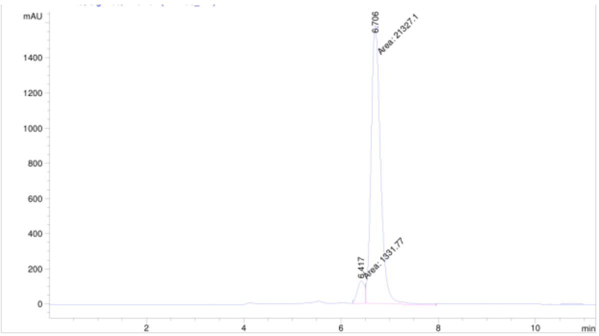

Peak #	RetTime [min]	Type	Width [min]	Area [mAU*s]	Height [mAU]	Area %
1	6.417	MM	0.1723	1331.77380	128.79959	5.8775
2	6.706	MM	0.2248	2.13271e4	1581.52661	94.1225

(*S,Z*)-5-benzyl-8,9-diphenylbenzo[2,3][1,4]diazocino[1,8-*a*]indol-6(5*H*)-one (2ap)

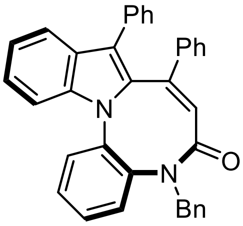

Synthetized from **1ap** (50 mg, 0.1 mmol), **L₇**AuCl (6.0 mg, 0.005 mmol), $AgSbF_6$ (1.7 mg, 0.005 mmol) and 1 mL of Ph-Cl. Purification by Biotage® Selekt (Hex/AcOEt 97:3 → 70:30) gave **2ap** (44 mg, 88% yield) as a white solid.

^{1}H NMR (300 MHz, $CDCl_3$): 7.83 (d, *J* = 8.0 Hz, 1H), 7.56 – 7.32 (m, 6H), 7.29 – 7.15 (m, 3H), 7.14 – 6.93 (m, 7H), 6.86 (m, 1H), 6.74 – 6.59 (m, 6H), 5.70 (d, *J* = 14.5 Hz, 1H), 4.30 (d, *J* = 14.5 Hz, 1H).

^{13}C NMR (75 MHz, $CDCl_3$): 168.49 (C), 139.66 (C), 137.95 (C), 137.46 (C), 137.02 (C), 135.32 (C), 135.21 (C), 134.29 (C), 133.81 (C), 129.10 (CH), 128.82 (CH), 128.74 (CH), 128.37 (CH), 128.24 (CH), 128.09 (CH), 128.04 (CH), 127.84 (CH), 127.81 (CH), 127.27 (CH), 127.04 (CH), 126.83 (C), 126.21 (CH), 126.18 (CH), 123.30 (CH), 121.15 (CH), 119.68 (CH), 118.58 (C), 111.07 (CH), 52.43 (CH_2). One CH is missing, probably overlapped.

HRMS (ESI) calculated for $C_{36}H_{26}N_2ONa$ $[M+Na]^+$ requires m/z = 525.1943, found m/z 525.1944.

[α]^D$_{25}$ = +220.7 (c = 0.25, $CHCl_3$).

R_t (OD-H column, Hex/*i*-PrOH 80/20, 0.8 mL/min, 266.4 nm): tr(major) = 7.5 min, tr(minor) = 6.4 min, 8:92 e.r.

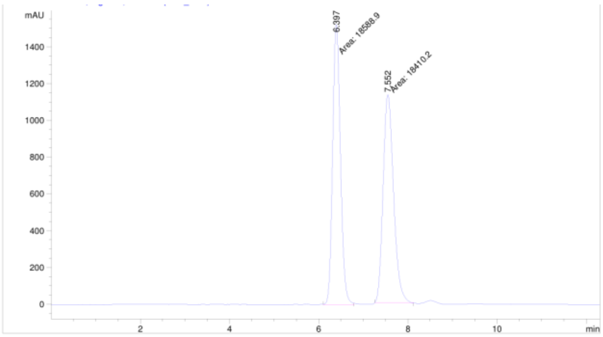

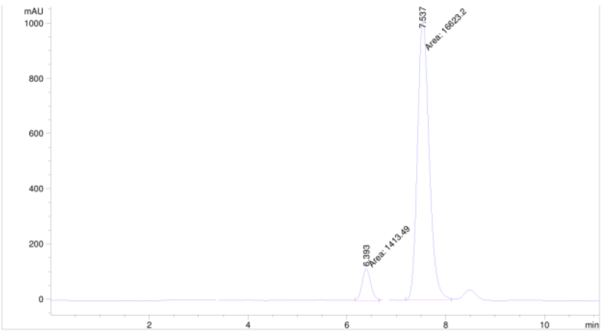

Peak #	RetTime [min]	Type	Width [min]	Area [mAU*s]	Height [mAU]	Area %
1	6.397	MM	0.2031	1.85889e4	1525.68384	50.2415
2	7.552	MM	0.2714	1.84102e4	1130.43066	49.7585

Peak #	RetTime [min]	Type	Width [min]	Area [mAU*s]	Height [mAU]	Area %
1	6.393	MM	0.2044	1413.48950	115.25904	7.8368
2	7.537	MM	0.2742	1.66232e4	1010.47015	92.1632

(*S,Z*)-5-benzyl-9-(naphthalen-2-yl)-8-phenylbenzo[2,3][1,4]diazocino[1,8-*a*]indol-6(5*H*)-one (2aq)

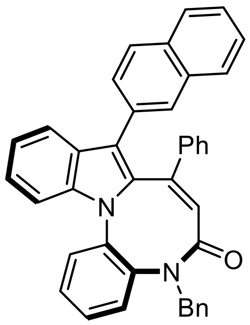

Synthetized from **1aq** (55 mg, 0.1 mmol), **L_7**AuCl (6.0 mg, 0.005 mmol), $AgSbF_6$ (1.7 mg, 0.005 mmol) and 1 mL of Ph-Cl. Purification by Biotage® Selekt (Hex/AcOEt 95:5 → 60:40) gave **2aq** (54 mg, 99% yield) as a white solid.

^{1}H NMR (300 MHz, $CDCl_3$): 7.99 – 7.86 (m, 2H), 7.82 (dd, *J* = 7.4, 1.9 Hz, 1H), 7.77 – 7.65 (m, 3H), 7.56 – 7.32 (m, 6H), 7.24 (m, 1H), 7.16 – 7.02 (m, 3H), 6.99 – 6.80 (m, 4H), 6.75 (s, 1H), 6.72 – 6.62 (m, 5H), 5.71 (d, *J* = 14.5 Hz, 1H), 4.34 (d, *J* = 14.5 Hz, 1H).

^{13}C NMR (75 MHz, $CDCl_3$): 168.50 (C), 139.74 (C), 138.02 (C), 137.58 (C), 137.05 (C), 135.30 (C), 135.25 (C), 134.57 (C), 133.39 (C), 132.00 (C), 131.50 (C), 128.89 (CH), 128.76 (CH), 128.41 (CH), 128.29 (CH), 128.08 (CH), 127.92 (CH), 127.89 (CH), 127.85 (CH), 127.64 (CH), 127.54 (CH), 127.30 (CH), 127.04 (C), 126.90 (CH), 126.40 (CH), 125.70 (CH), 125.28 (CH), 123.42 (CH), 121.30 (CH), 119.74 (CH), 118.36 (C), 111.17 (CH), 52.45 (CH_2). Three CH are missing, probably overlapped.

HRMS (ESI) calculated for $C_{40}H_{28}N_2ONa$ $[M+Na]^+$ requires m/z = 575.2099, found m/z 575.2104.

$[\alpha]^D_{25}$ = +222.6 (c = 0.25, $CHCl_3$).

R_t (OD-H column, Hex/*i*-PrOH 80/20, 0.8 mL/min, 266.4 nm): tr(major) = 8.5 min, tr(minor) = 7.5 min, 3:97 e.r.

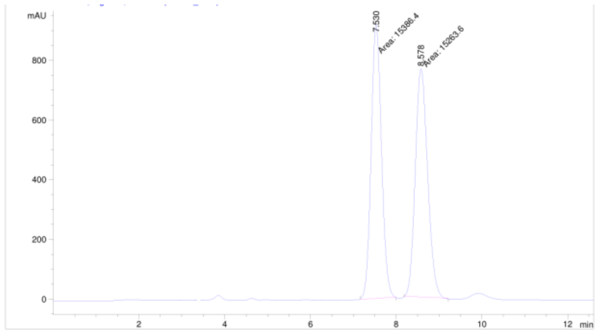

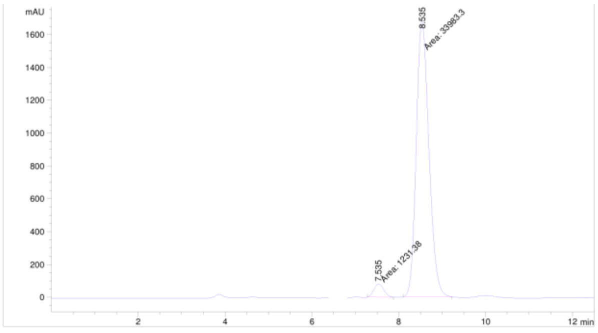

Peak #	RetTime [min]	Type	Width [min]	Area [mAU*s]	Height [mAU]	Area %
1	7.530	MM	0.2792	1.53864e4	918.37909	50.2004
2	8.578	MM	0.3324	1.52636e4	765.20721	49.7996

Peak #	RetTime [min]	Type	Width [min]	Area [mAU*s]	Height [mAU]	Area %
1	7.535	MM	0.2585	1231.37744	79.40237	3.4968
2	8.535	MM	0.3372	3.39833e4	1679.84229	96.5032

(*S,Z*)-5-benzyl-11-fluoro-9-methyl-8-phenylbenzo[2,3][1,4]diazocino[1,8-*a*]indol-6(5*H*)-one (2ar)

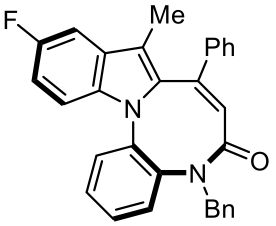

Synthetized from **1ar** (46 mg, 0.1 mmol), **L$_7$**AuCl (6.0 mg, 0.005 mmol), AgSbF$_6$ (1.7 mg, 0.005 mmol) and 1 mL of Ph-Cl. Purification by flash column chromatography (SiO_2, Hex/AcOEt 5:1) gave **2ar** (34 mg, 73% yield) as a white solid.

^{1}H NMR (400 MHz, DMSO): 7.74 (dt, *J* = 8.1, 1.0 Hz, 1H), 7.52 (ddd, *J* = 8.1, 6.2, 2.8 Hz, 1H), 7.45 – 7.28 (m, 6H), 7.27 – 7.21 (m, 2H), 6.96 (m, 1H), 6.87 (m, 1H), 6.81 (s, 1H), 6.74 (dd, *J* = 8.5, 7.0 Hz, 2H), 6.64 – 6.55 (m, 2H), 6.43 (dd, *J* = 8.9, 4.4 Hz, 1H), 5.43 (d, *J* = 14.9 Hz, 1H), 4.46 (d, *J* = 15.0 Hz, 1H), 1.85 (s, 3H).

^{13}C NMR (101 MHz, DMSO): 167.66 (C), 158.06 (d, *J* = 233.7 Hz, C), 139.97 (C), 138.19 (C), 136.71 (C), 136.18 (C), 135.16 (C), 134.89 (C), 134.24 (C), 129.72 (CH), 129.31 (CH), 129.20 (CH), 129.12 (CH), 129.08 (C), 128.64 (CH), 128.32 (CH), 128.24 (CH), 127.82 (CH), 127.39 (CH), 127.22 (CH), 126.82 (CH), 112.35 (d, *J* = 9.8 Hz, CH), 111.27 (d, *J* = 26.3 Hz, CH), 111.19 (d, *J* = 4.7 Hz, C), 103.94 (d, *J* = 23.4 Hz, CH), 51.86 (CH_2), 9.73 (CH_3).

HRMS (ESI) calculated for $C_{31}H_{23}N_2OFNa$ $[M+Na]^+$ requires m/z = 481.1692, found m/z 481.1692.

[α]$^{D}_{25}$ = +303.8 (c = 0.25, $CHCl_3$).

R$_t$ (OD-H column, Hex/*i*-PrOH 80/20, 0.8 mL/min, 266.4 nm): tr(major) = 9.3 min, tr(minor) = 7.8 min, 2:98 e.r.

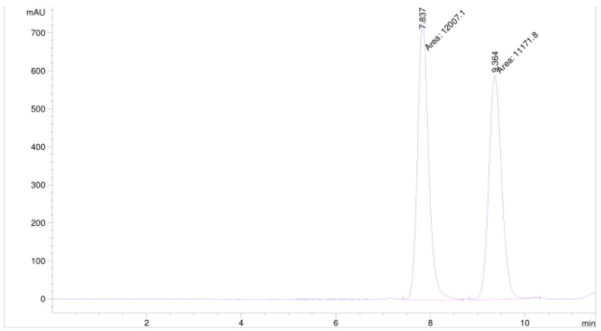

Peak #	RetTime [min]	Type	Width [min]	Area [mAU*s]	Height [mAU]	Area %
1	7.837	MM	0.2721	1.20071e4	735.36456	51.8018
2	9.364	MM	0.3150	1.11718e4	591.17645	48.1982

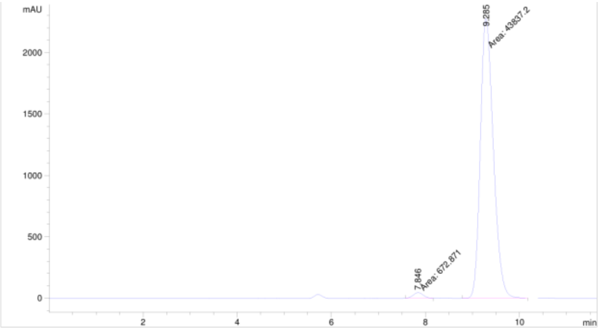

Peak #	RetTime [min]	Type	Width [min]	Area [mAU*s]	Height [mAU]	Area %
1	7.846	MM	0.2451	672.87061	45.76122	1.5117
2	9.285	MM	0.3209	4.38372e4	2277.07349	98.4883

(*S,Z*)-5-benzyl-11-methoxy-9-methyl-8-phenylbenzo[2,3][1,4]diazocino[1,8-*a*]indol-6(5*H*)-one (2as)

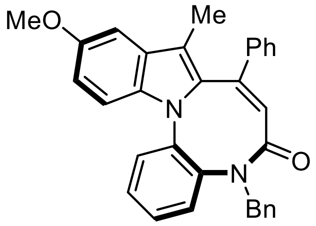

Synthetized from **1as** (47 mg, 0.1 mmol), **L₇**AuCl (6.0 mg, 0.005 mmol), $AgSbF_6$ (1.7 mg, 0.005 mmol) and 1 mL of Ph-Cl. Purification by flash column chromatography (SiO_2, Hex/AcOEt 7:1 → 3:1) gave **2as** (45 mg, 96% yield) as a white solid.

^{1}H NMR (300 MHz, $CDCl_3$): 7.49 – 7.19 (m, 9H), 7.03 – 6.91 (m, 2H), 6.79 (dd, *J* = 8.5, 6.8 Hz, 2H), 6.74 – 6.68 (m, 3H), 6.57 (d, *J* = 0.7 Hz, 1H), 6.43 (d, *J* = 8.9 Hz, 1H), 5.57 (d, *J* = 14.6 Hz, 1H), 4.35 (d, *J* = 14.6 Hz, 1H), 3.91 (s, 3H), 1.90 (s, 3H).

^{13}C NMR (75 MHz, $CDCl_3$): 168.35 (C), 154.68 (C), 139.65 (C), 139.07 (C), 136.34 (C), 136.21 (C), 135.34 (C), 135.28 (C), 132.90 (C), 129.35 (C), 128.63 (CH), 128.47 (CH), 128.45 (CH), 128.37 (CH), 128.26 (CH), 128.16 (CH), 128.03 (CH), 127.43 (CH), 127.31 (CH), 127.22 (CH), 124.86 (CH), 113.19 (CH), 112.49 (C), 111.96 (CH), 100.07 (CH), 55.94 (CH_3), 52.39 (CH_2), 9.68 (CH_3).

HRMS (ESI) calculated for $C_{32}H_{26}N_2O_2Na$ $[M+Na]^+$ requires m/z = 493.1892, found m/z 493.1891.

$[\alpha]^D_{25}$ = +298.4 (c = 0.25, $CHCl_3$).

R_t (OD-H column, Hex/*i*-PrOH 80/20, 0.8 mL/min, 266.4 nm): tr(major) = 9.0 min, tr(minor) = 7.6 min, 3:97 e.r.

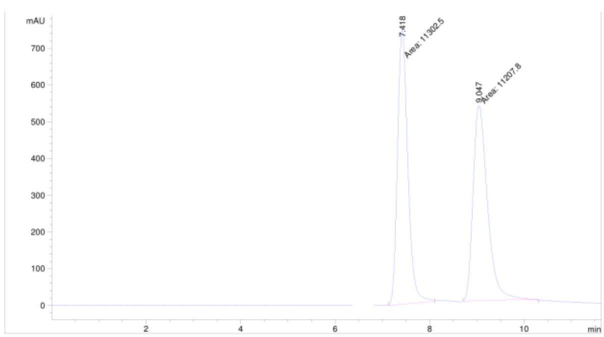

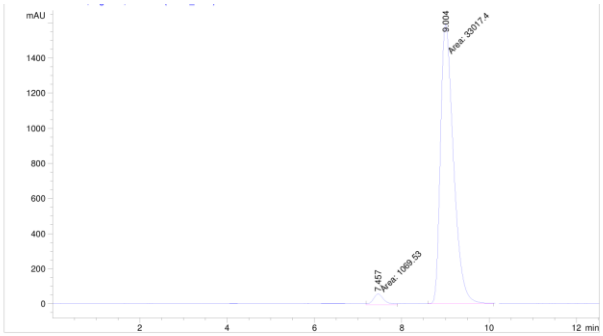

Peak #	RetTime [min]	Type	Width [min]	Area [mAU*s]	Height [mAU]	Area %
1	7.418	MM	0.2518	1.13025e4	747.99347	50.2103
2	9.047	MM	0.3510	1.12078e4	532.21649	49.7897

Peak #	RetTime [min]	Type	Width [min]	Area [mAU*s]	Height [mAU]	Area %
1	7.457	MM	0.2890	1069.53003	61.67268	3.1377
2	9.004	MM	0.3457	3.30174e4	1591.95276	96.8623

(*S,Z*)-5-benzyl-12-bromo-9-methyl-8-phenylbenzo[2,3][1,4]diazocino[1,8-*a*]indol-6(5*H*)-one (2at)

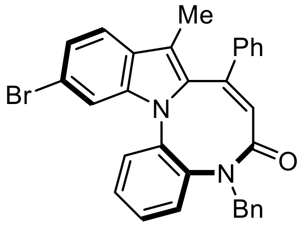

Synthetized from **1at** (54 mg, 0.1 mmol), **L_7**AuCl (6.0 mg, 0.005 mmol), $AgSbF_6$ (1.7 mg, 0.005 mmol) and 1 mL of Ph-Cl. Purification by flash column chromatography (SiO_2, Hex/AcOEt 7:1 → 3:1) gave **2at** (50 mg, 92% yield) as a white solid.

^{1}H NMR (300 MHz, $CDCl_3$): 7.55 – 7.15 (m, 11H), 7.03 (t, *J* = 7.4 Hz, 1H), 6.75 (t, *J* = 7.6 Hz, 2H), 6.66 – 6.58 (m, 3H), 6.44 (s, 1H), 5.75 (d, *J* = 14.2 Hz, 1H), 4.15 (d, *J* = 14.2 Hz, 1H), 1.90 (s, 3H).

^{13}C NMR (75 MHz, $CDCl_3$): 167.90 (C), 139.57 (C), 138.59 (C), 137.92 (C), 135.87 (C), 135.46 (C), 134.97 (C), 134.89 (C), 128.96 (CH), 128.79 (CH), 128.56 (CH), 128.22 (CH), 128.12 (CH), 128.01 (CH), 127.73 (CH), 127.63 (CH), 127.53 (C), 127.16 (CH), 125.29 (CH), 123.35 (CH), 119.89 (CH), 116.55 (C), 113.70 (CH), 112.52 (C), 52.27 (CH_2), 9.43 (CH_3). One CH is missing, probably overlapped.

HRMS (ESI) calculated for $C_{31}H_{23}N_2ONaBr$ $[M+Na]^+$ requires m/z = 541.0891, found m/z 541.0899.

$[\alpha]^D_{25}$ = +216.5 (c = 0.25, $CHCl_3$).

R_t (OD-H column, Hex/*i*-PrOH 80/20, 0.8 mL/min, 266.4 nm): tr(major) = 10.3 min, tr(minor) = 8.8 min, 3:97 e.r.

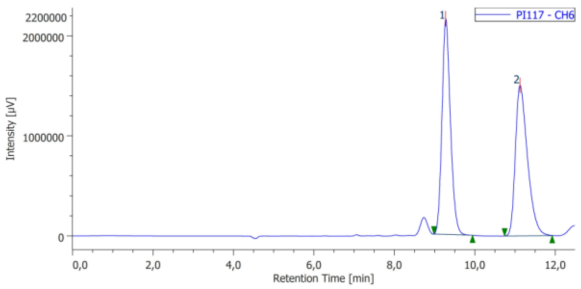

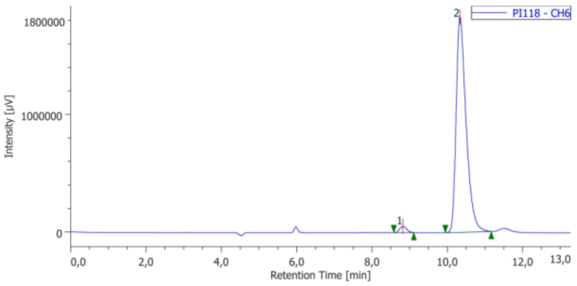

#	Peak Name	CH	tR [min]	Area [µV·sec]	Height [µV]	Area%
1	Unknown	6	9,273	31448277	2155719	50,007
2	Unknown	6	11,120	31439195	1504090	49,993

#	Peak Name	CH	tR [min]	Area [µV·sec]	Height [µV]	Area%
1	Unknown	6	8,820	697230	53776	2,034
2	Unknown	6	10,333	33583498	1832970	97,966

(*R,Z*)-5-benzyl-13-bromo-9-methyl-8-phenylbenzo[2,3][1,4]diazocino[1,8-*a*]indol-6(5*H*)-one (2au)

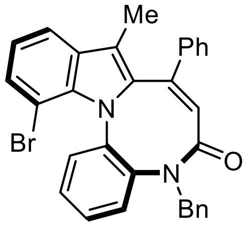

Synthetized from **1au** (54 mg, 0.1 mmol), **L₇**AuCl (6.0 mg, 0.005 mmol), $AgSbF_6$ (1.7 mg, 0.005 mmol) and 1 mL of Ph-Cl. Purification by Biotage® Selekt (Hex/AcOEt 95:5 → 75:25) gave **2au** (46 mg, 86% yield) as a white solid.

^{1}H NMR (400 MHz, $CDCl_3$): 7.59 (dd, *J* = 7.9, 1.0 Hz, 1H), 7.39 – 7.24 (m, 8H), 7.23 – 7.15 (m, 2H), 7.12 – 7.03 (m, 6H), 6.56 (s, 1H), 5.14 (d, *J* = 14.8 Hz, 1H), 4.80 (d, *J* = 14.8 Hz, 1H), 1.92 (s, 3H).

^{13}C NMR (101 MHz, $CDCl_3$): 168.42 (C), 141.01 (C), 138.93 (C), 137.24 (C), 136.98 (C), 135.69 (C), 135.49 (C), 134.78 (C), 132.51 (C), 130.51 (CH), 128.91 (CH), 128.69 (CH), 128.60 (CH), 128.35 (CH), 128.16 (CH), 128.06 (CH), 127.68 (CH), 127.39 (CH), 127.35 (CH), 126.85 (CH), 125.83 (CH), 121.56 (CH), 118.50 (CH), 113.64 (C), 105.17 (C), 51.98 (CH_2), 9.70 (CH_3).

HRMS (ESI) calculated for $C_{31}H_{23}N_2ONaBr$ $[M+Na]^+$ requires m/z = 541.0891, found m/z 541.0887.

$[\alpha]^D_{25}$ = +309.4 (c = 0.25, $CHCl_3$).

R_t (OD-H column, Hex/*i*-PrOH 80/20, 0.8 mL/min, 310 nm): tr(major) = 12,3 min, tr(minor) = 7.3 min, 2:98 e.r.

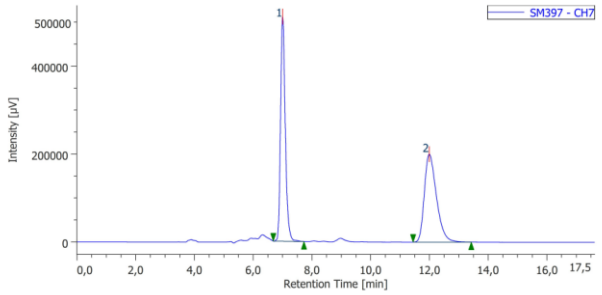

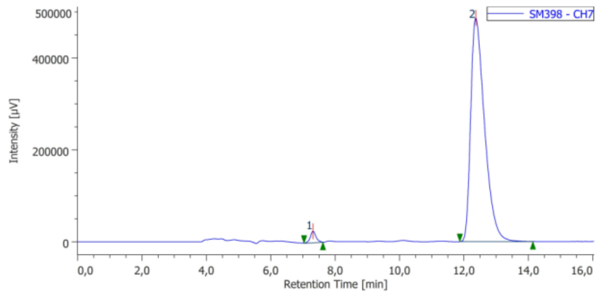

#	Peak Name	CH	tR [min]	Area [µV·sec]	Height [µV]	Area%
1	Unknown	7	7,000	5737502	510580	50,026
2	Unknown	7	11,990	5731529	200067	49,974

#	Peak Name	CH	tR [min]	Area [µV·sec]	Height [µV]	Area%
1	Unknown	7	7,307	311698	25146	2,089
2	Unknown	7	12,363	14609791	486534	97,911

(*S,Z*)-5-allyl-9-methyl-8-phenylbenzo[2,3][1,4]diazocino[1,8-*a*]indol-6(5*H*)-one (2av)

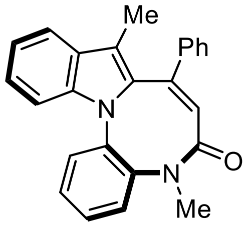

Synthetized from **1av** (36 mg, 0.1 mmol), **L₇**AuCl (6.0 mg, 0.005 mmol), $AgSbF_6$ (1.7 mg, 0.005 mmol) and 1 mL of Ph-Cl. Purification by Flash column chromatography (SiO_2, Hex/AcOEt 4:1) gave **2av** (30 mg, 83% yield) as a white solid.

^{1}H NMR (300 MHz, DMSO): 7.68 (d, *J* = 7.8 Hz, 1H), 7.63 – 7.41 (m, 4H), 7.35 – 7.07 (m, 8H), 6.66 (s, 1H), 3.07 (s, 3H), 1.82 (s, 3H).

^{13}C NMR (75 MHz, DMSO): 167.55 (C), 141.88 (C), 138.41 (C), 137.61 (C), 135.18 (C), 134.91 (C), 134.48 (C), 129.87 (CH), 129.23 (CH), 129.15 (CH), 129.08 (CH), 129.05 (C), 128.92 (CH), 127.74 (CH), 127.22 (CH), 126.61 (CH), 123.59 (CH), 120.77 (CH), 119.44 (CH), 111.09 (C), 110.83 (CH), 36.64 (CH_3), 9.66 (CH_3).

HRMS (ESI) calculated for $C_{25}H_{20}N_2ONa$ $[M+Na]^+$ requires m/z = 387.1473, found m/z 387.1470.

$[\alpha]^D_{25}$ = +134.8 (c = 0.165, $CHCl_3$).

R_t (OD-H column, Hex/*i*-PrOH 80/20, 0.8 mL/min, 266.4 nm): tr(major) = 11.5 min, tr(minor) = 8.2 min, 29:71 e.r.

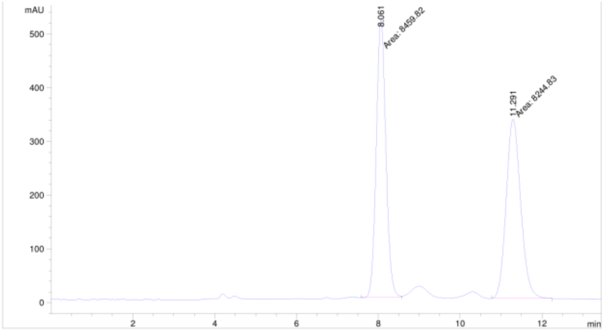

Peak #	RetTime [min]	Type	Width [min]	Area [mAU*s]	Height [mAU]	Area %
1	8.061	MM	0.2723	8459.82422	517.75079	50.6435
2	11.291	MM	0.4123	8244.82715	333.30499	49.3565

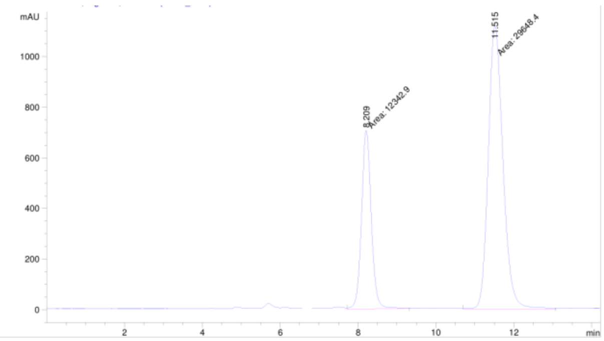

Peak #	RetTime [min]	Type	Width [min]	Area [mAU*s]	Height [mAU]	Area %
1	8.209	MM	0.2910	1.23429e4	706.83362	29.3940
2	11.515	MM	0.4413	2.96484e4	1119.77734	70.6060

(*S,Z*)-5,9-dimethyl-8-phenylbenzo[2,3][1,4]diazocino[1,8-*a*]indol-6(5*H*)-one (2aw)

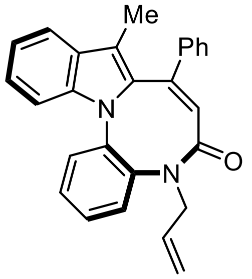

Synthetized from **1aw** (39 mg, 0.1 mmol), **L$_7$**AuCl (6.0 mg, 0.005 mmol), $AgSbF_6$ (1.7 mg, 0.005 mmol) and 1 mL of Ph-Cl. Purification by flash column chromatography (SiO_2, Hex/AcOEt 5:1) gave **2aw** (32 mg, 83% yield) as a white solid.

^{1}H NMR (300 MHz, $CDCl_3$): 7.61 (m, 1H), 7.53 – 7.32 (m, 4H), 7.28 (s, 5H), 7.24 – 7.13 (m, 3H), 6.54 (s, 1H), 5.26 (m, 1H), 4.82 (dq, *J* = 9.0, 5.6 Hz, 3H), 3.83 (dd, *J* = 15.1, 7.8 Hz, 1H), 1.92 (s, 3H).

^{13}C NMR (75 MHz, $CDCl_3$): 167.83 (C), 140.15 (C), 138.89 (C), 137.59 (C), 136.30 (C), 136.27 (C), 134.75 (C), 132.19 (CH), 129.31 (C), 128.75 (CH), 128.67 (CH), 128.62 (CH), 128.50 (CH), 128.33 (CH), 127.61 (CH), 127.26 (CH), 125.12 (CH), 122.98 (CH), 120.36 (CH), 119.08 (CH), 118.54 (CH_2), 112.83 (C), 110.85 (CH), 51.46 (CH_2), 9.51 (CH_3).

HRMS (ESI) calculated for $C_{27}H_{22}N_2ONa$ $[M+Na]^+$ requires m/z = 413.1630, found m/z 413.1632.

$[\alpha]^D_{25}$ = +487.7 (c = 0.25, $CHCl_3$).

R_t (OD-H column, Hex/*i*-PrOH 80/20, 0.8 mL/min, 266.4 nm): tr(major) = 8.2 min, tr(minor) = 6.3 min, 4:96 e.r.

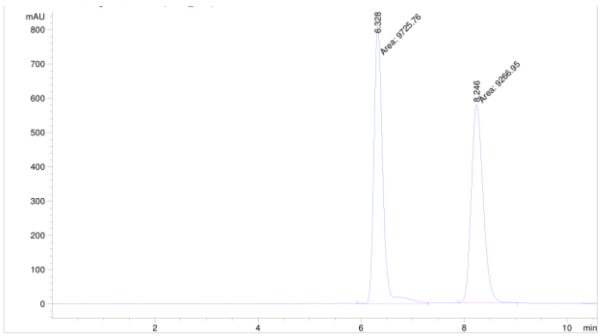

Peak #	RetTime [min]	Type	Width [min]	Area [mAU*s]	Height [mAU]	Area %
1	6.328	MM	0.1991	9725.75879	814.08203	51.2078
2	8.246	MM	0.2668	9266.95313	578.99548	48.7922

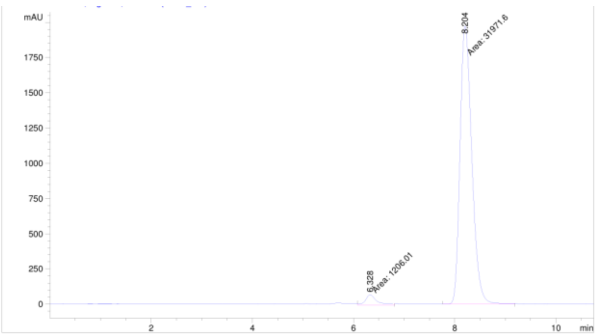

Peak #	RetTime [min]	Type	Width [min]	Area [mAU*s]	Height [mAU]	Area %
1	6.328	MM	0.2702	1206.00659	74.40267	3.6350
2	8.204	MM	0.2699	3.19716e4	1974.42468	96.3650

(*S,Z*)-5-(4-methoxybenzyl)-9-methyl-8-phenylbenzo[2,3][1,4]diazocino[1,8-*a*]indol-6(5*H*)-one (2ax)

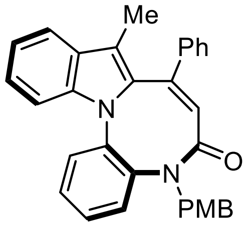

Synthetized from **1ax** (47 mg, 0.1 mmol), **L₇**AuCl (6.0 mg, 0.005 mmol), $AgSbF_6$ (1.7 mg, 0.005 mmol) and 1 mL of Ph-Cl. Purification by Biotage® Selekt (Hex/AcOEt 95:5 → 60:40) gave **2ax** (38 mg, 80% yield) as a white solid.

^{1}H NMR (300 MHz, $CDCl_3$): 7.55 (dt, *J* = 7.9, 0.8 Hz, 1H), 7.46 – 7.34 (m, 2H), 7.33 – 7.29 (m, 2H), 7.28 – 7.22 (m, 5H), 7.13 (ddd, *J* = 8.0, 7.0, 1.0 Hz, 1H), 7.01 (ddd, *J* = 8.3, 7.0, 1.2 Hz, 1H), 6.62 – 6.52 (m, 3H), 6.50 (d, *J* = 8.2 Hz, 1H), 6.30 – 6.14 (m, 2H), 5.57 (d, *J* = 14.3 Hz, 1H), 4.19 (d, *J* = 14.3 Hz, 1H), 3.61 (s, 3H), 1.93 (s, 3H).

^{13}C NMR (75 MHz, $CDCl_3$): 168.14 (C), 158.68 (C), 139.67 (C), 138.97 (C), 137.39 (C), 136.24 (C), 136.07 (C), 134.35 (C), 129.28 (CH), 128.83 (C), 128.62 (CH), 128.50 (CH), 128.47 (CH), 128.25 (CH), 127.69 (CH), 127.36 (C), 127.23 (CH), 125.07 (CH), 125.02 (CH), 122.59 (CH), 120.05 (CH), 118.61 (CH), 113.36 (CH), 112.55 (C), 110.90 (CH), 54.86 (CH_3), 51.78 (CH_2), 9.54 (CH_3).

HRMS (ESI) calculated for $C_{32}H_{26}N_2O_2Na$ $[M+Na]^+$ requires m/z = 493.1892, found m/z 493.1894.

$[\alpha]^D_{25}$ = +259.7 (c = 0.245, $CHCl_3$).

R_t (OD-H column, Hex/*i*-PrOH 80/20, 0.8 mL/min, 266.4 nm): tr(major) = 11.5 min, tr(minor) = 8.8 min, 2:98 e.r.

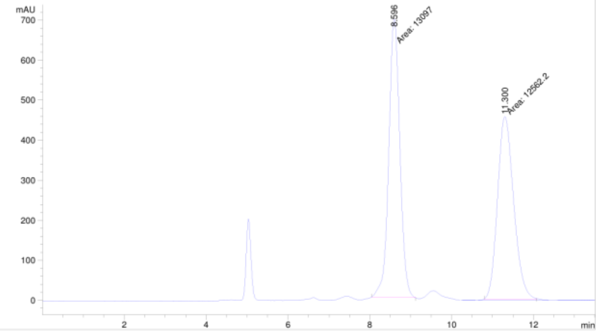

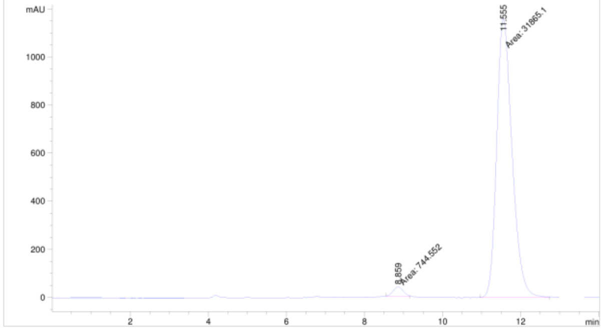

Peak #	RetTime [min]	Type	Width [min]	Area [mAU*s]	Height [mAU]	Area %
1	8.596	MM	0.3135	1.30970e4	696.38330	51.0421
2	11.300	MM	0.4579	1.25622e4	457.19772	48.9579

Peak #	RetTime [min]	Type	Width [min]	Area [mAU*s]	Height [mAU]	Area %
1	8.859	MM	0.3083	744.55176	40.25227	2.2832
2	11.555	MM	0.4578	3.18651e4	1160.11206	97.7168

(*S,Z*)-5-benzyl-1,9-dimethyl-8-phenylbenzo[2,3][1,4]diazocino[1,8-*a*]indol-6(5*H*)-one (2ay)

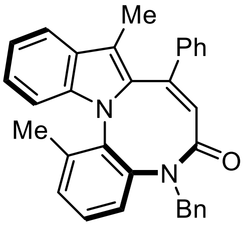

Synthetized from **1ay** (45 mg, 0.1 mmol), **L₇**AuCl (6.0 mg, 0.005 mmol), $AgSbF_6$ (1.7 mg, 0.005 mmol) and 1 mL of Ph-Cl. Purification by Biotage® Selekt (Hex/AcOEt 96:4 → 60:40) gave **2ay** (33 mg, 73% yield) as a white solid.

^{1}H NMR (400 MHz, $CDCl_3$): 7.60 (dt, *J* = 7.9, 1.0 Hz, 1H), 7.41 – 7.23 (m, 7H), 7.22 – 7.10 (m, 2H), 7.07 – 6.95 (m, 2H), 6.87 – 6.78 (m, 2H), 6.77 – 6.72 (m, 2H), 6.65 – 6.58 (m, 1H), 6.28 (dt, *J* = 8.2, 0.9 Hz, 1H), 5.44 (d, *J* = 14.6 Hz, 1H), 4.46 (d, *J* = 14.6 Hz, 1H), 2.00 (s, 3H), 1.84 (s, 3H).

^{13}C NMR (101 MHz, $CDCl_3$): 168.51 (C), 141.05 (C), 138.49 (C), 137.79 (C), 136.87 (C), 136.00 (C), 135.52 (C), 134.33 (C), 133.73 (C), 130.21 (CH), 128.73 (CH), 128.59 (CH), 128.49 (C), 128.39 (CH), 128.15 (CH), 128.13 (CH), 127.28 (CH), 127.24 (CH), 124.72 (CH), 124.53 (CH), 122.94 (CH), 119.84 (CH), 118.88 (CH), 112.22 (C), 111.32 (CH), 52.32 (CH_2), 18.31 (CH_3), 9.57 (CH_3).

HRMS (ESI) calculated for $C_{32}H_{26}N_2ONa$ $[M+Na]^+$ requires m/z = 477.1943, found m/z 477.1947.

[α]^{D}$_{25}$ = +251.2 (c = 0.25, $CHCl_3$).

R_t (OD-H column, Hex/*i*-PrOH 80/20, 0.8 mL/min, 266.4 nm): tr(major) = 9.5 min, tr(minor) = 7.3 min, 22:78 e.r.

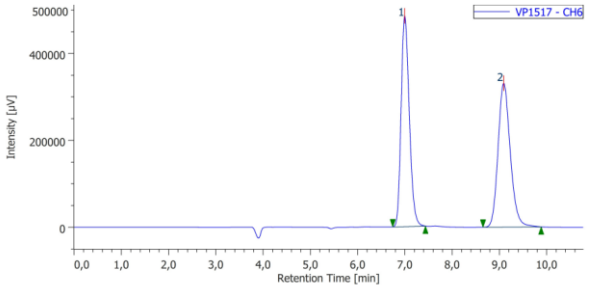

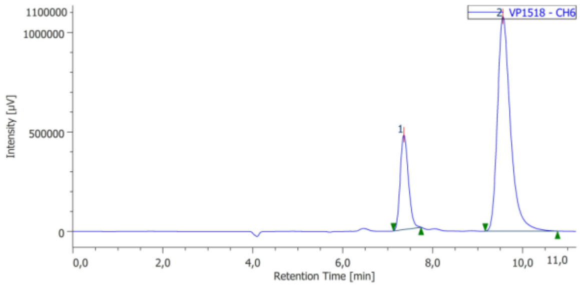

#	Peak Name	CH	tR [min]	Area [µV·sec]	Height [µV]	Area%
1	Unknown	6	6,993	5901816	484834	49,666
2	Unknown	6	9,087	5981189	331001	50,334

#	Peak Name	CH	tR [min]	Area [µV·sec]	Height [µV]	Area%
1	Unknown	6	7,353	5981314	475391	21,560
2	Unknown	6	9,557	21761574	1077833	78,440

(*R,Z*)-5-benzyl-1-bromo-9-methyl-8-phenylbenzo[2,3][1,4]diazocino[1,8-*a*]indol-6(5*H*)-one (2az)

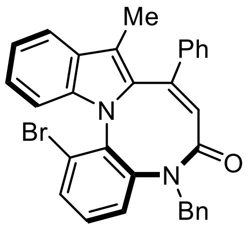

Synthetized from **1az** (54 mg, 0.1 mmol), $\mathbf{L_7}$AuCl (6.0 mg, 0.005 mmol), $AgSbF_6$ (1.7 mg, 0.005 mmol) and 1 mL of Ph-Cl. Purification by Biotage® Selekt (Hex/AcOEt 95:5 → 75:25) gave **2az** (27 mg, 50% yield) as a white solid.

^{1}H NMR (400 MHz, $CDCl_3$): 7.59 (td, *J* = 7.7, 1.2 Hz, 2H), 7.51 – 7.42 (m, 2H), 7.38 (dd, *J* = 8.1, 1.5 Hz, 1H), 7.36 – 7.26 (m, 4H), 7.18 (ddd, *J* = 8.0, 7.1, 1.0 Hz, 1H), 7.11 – 6.98 (m, 2H), 6.89 – 6.81 (m, 2H), 6.80 – 6.73 (m, 2H), 6.56 (d, *J* = 0.6 Hz, 1H), 6.35 (dt, *J* = 8.2, 0.9 Hz, 1H), 5.43 (d, *J* = 14.6 Hz, 1H), 4.47 (d, *J* = 14.6 Hz, 1H), 1.99 (s, 3H).

^{13}C NMR (101 MHz, $CDCl_3$): 168.33 (C), 142.68 (C), 138.46 (C), 136.70 (C), 136.67 (C), 135.38 (C), 135.12 (C), 133.52 (C), 132.93 (CH), 129.53 (CH), 128.88 (CH), 128.75 (C), 128.58 (CH), 128.29 (CH), 128.20 (CH), 127.61 (CH), 127.49 (CH), 126.20 (CH), 124.26 (CH), 123.79 (C), 122.99 (CH), 120.21 (CH), 118.85 (CH), 113.03 (C), 111.94 (CH), 52.32 (CH_2), 9.49 (CH_3).

HRMS (ESI) calculated for $C_{31}H_{23}N_2ONaBr$ $[M+Na]^+$ requires m/z = 541.0891, found m/z 541.0893.

$[\alpha]^D{}_{25}$ = +287.4 (c = 0.25, $CHCl_3$).

R_t (OD-H column, Hex/*i*-PrOH 80/20, 0.8 mL/min, 310 nm): tr(major) = 12.4 min, tr(minor) = 9.2 min, 27:73 e.r.

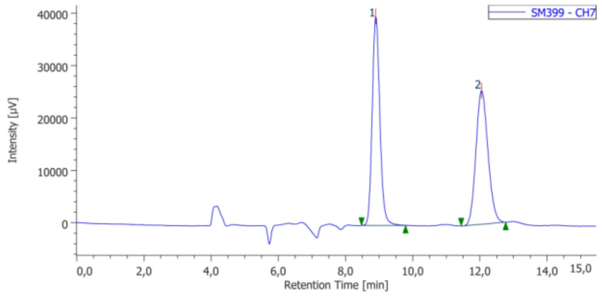

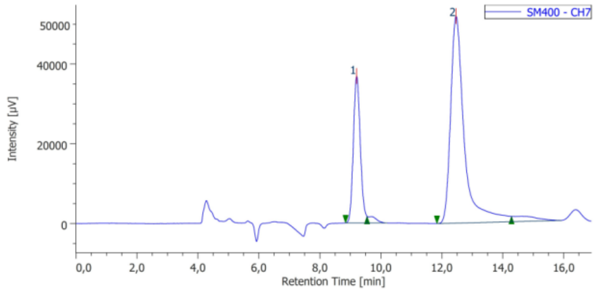

#	Peak Name	CH	tR [min]	Area [µV·sec]	Height [µV]	Area%
1	Unknown	7	8,897	630643	39855	49,822
2	Unknown	7	12,043	635137	25487	50,178

#	Peak Name	CH	tR [min]	Area [µV·sec]	Height [µV]	Area%
1	Unknown	7	9,203	585353	36826	26,599
2	Unknown	7	12,463	1615328	51854	73,401

(*S,Z*)-5-benzyl-2,9-dimethyl-8-phenylbenzo[2,3][1,4]diazocino[1,8-*a*]indol-6(5*H*)-one (2ba)

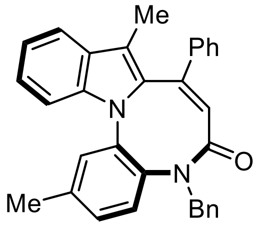

Synthetized from **1ba** (45 mg, 0.1 mmol), **L₇**AuCl (6.0 mg, 0.005 mmol), $AgSbF_6$ (1.7 mg, 0.005 mmol) and 1 mL of Ph-Cl. Purification by Biotage® Selekt (Hex/AcOEt 94:6 → 60:40) gave **2ba** (43 mg, 95% yield) as a white solid.

^{1}H NMR (400 MHz, $CDCl_3$): 7.59 (dt, *J* = 8.0, 1.0 Hz, 1H), 7.30 (s, 6H), 7.23 – 7.12 (m, 3H), 7.07 (ddd, *J* = 8.2, 7.0, 1.2 Hz, 1H), 7.00 – 6.91 (m, 1H), 6.81 – 6.68 (m, 4H), 6.62 (d, *J* = 9.1 Hz, 2H), 5.54 (d, *J* = 14.6 Hz, 1H), 4.37 (d, *J* = 14.6 Hz, 1H), 2.34 (s, 3H), 1.97 (s, 3H).

^{13}C NMR (101 MHz, $CDCl_3$): 168.50 (C), 139.09 (C), 138.77 (C), 137.48 (C), 137.17 (C), 136.23 (C), 135.60 (C), 135.51 (C), 134.46 (C), 129.33 (CH), 128.96 (C), 128.63 (CH), 128.50 (CH), 128.10 (CH), 127.97 (CH), 127.34 (CH), 127.15 (CH), 127.13 (CH), 125.06 (CH), 122.77 (CH), 120.12 (CH), 118.70 (CH), 112.62 (C), 111.03 (CH), 52.41 (CH_2), 20.97 (CH_3), 9.60 (CH_3). One CH is missing, probably overlapped.

HRMS (ESI) calculated for $C_{32}H_{26}N_2ONa$ $[M+Na]^+$ requires m/z = 477.1943, found m/z 477.1939.

$[\alpha]^D_{25}$ = +304.6 (c = 0.25, $CHCl_3$).

R_t (OD-H column, Hex/*i*-PrOH 80/20, 0.8 mL/min, 266.4 nm): tr(major) = 8.3 min, tr(minor) = 6.4 min, 2:98 e.r.

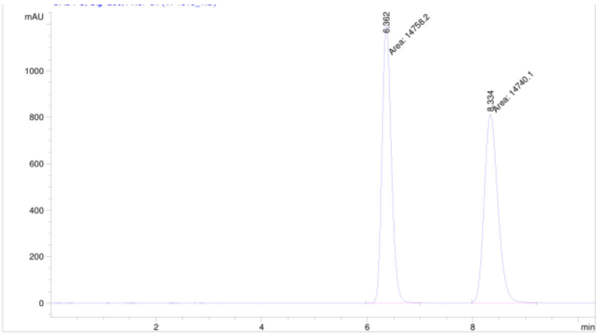

Peak #	RetTime [min]	Type	Width [min]	Area [mAU*s]	Height [mAU]	Area %
1	6.362	MM	0.2054	1.47582e4	1197.80017	50.0306
2	8.334	MM	0.3016	1.47401e4	814.53912	49.9694

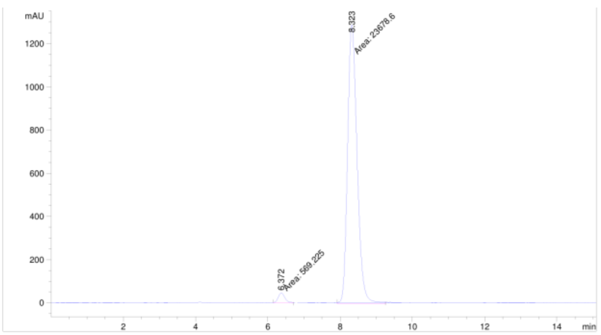

Peak #	RetTime [min]	Type	Width [min]	Area [mAU*s]	Height [mAU]	Area %
1	6.372	MM	0.2092	569.22528	45.34773	2.3475
2	8.323	MM	0.3054	2.36786e4	1292.10889	97.6525

(*R,Z*)-5-benzyl-8-phenylbenzo[*b*]pyrrolo[1,2-*d*][1,4]diazocin-6(5*H*)-one (4)

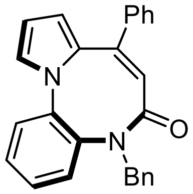

Synthetized from **3** (37 mg, 0.1 mmol), **L**$_7$AuCl (6.0 mg, 0.005 mmol), AgSbF$_6$ (1.7 mg, 0.005 mmol) and 1 mL of Ph-Cl. Purification by Biotage® Selekt (Hex/AcOEt 96:4 → 60:40) gave **4** (28 mg, 75% yield) as a white solid.

^{1}H NMR (300 MHz, CDCl$_3$): 7.40 – 7.34 (m, 2H), 7.33 – 7.21 (m, 6H), 7.21 – 7.07 (m, 4H), 6.83 (dd, *J* = 7.7, 1.8 Hz, 2H), 6.37 (d, *J* = 0.7 Hz, 1H), 6.29 (dd, *J* = 2.8, 1.7 Hz, 1H), 6.24 (dd, *J* = 3.7, 2.8 Hz, 1H), 6.13 (dd, *J* = 3.6, 1.7 Hz, 1H), 5.62 (d, *J* = 14.4 Hz, 1H), 4.32 (d, *J* = 14.4 Hz, 1H).

^{13}C NMR (75 MHz, CDCl$_3$): 168.58 (C), 140.54 (C), 138.56 (C), 137.87 (C), 136.96 (C), 135.81 (C), 132.88 (C), 128.69 (CH), 128.66 (CH), 128.44 (CH), 128.32 (CH), 128.29 (CH), 128.09 (CH), 128.06 (CH), 127.69 (CH), 127.20 (CH), 123.31 (CH), 122.15 (CH), 122.12 (CH), 122.09 (CH), 112.10 (CH), 109.81 (CH), 52.71 (CH$_2$).

HRMS (ESI) calculated for $C_{26}H_{20}N_2ONa$ [M+Na]$^+$ requires m/z = 399.1473, found m/z 399.1466.

[α]$^D_{25}$ = +67.6 (c = 0.25, CHCl$_3$).

R$_t$ (OD-H column, Hex/*i*-PrOH 80/20, 0.8 mL/min, 266.4 nm): tr(major) = 9.2 min, tr(minor) = 8.5 min, 31:69 e.r.

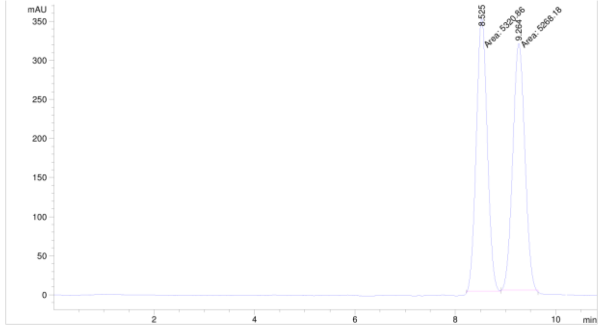

Peak #	RetTime [min]	Type	Width [min]	Area [mAU*s]	Height [mAU]	Area %
1	8.525	MM	0.2539	5320.85645	349.22531	50.2487
2	9.264	MM	0.2778	5268.18066	316.05753	49.7513

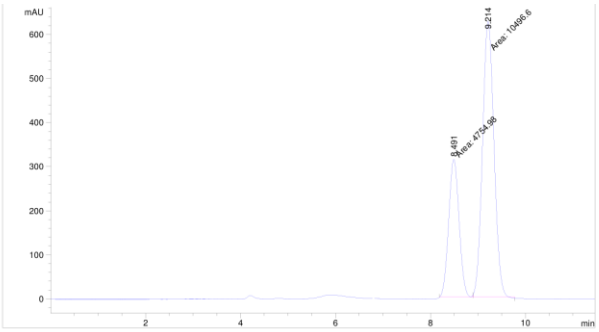

Peak #	RetTime [min]	Type	Width [min]	Area [mAU*s]	Height [mAU]	Area %
1	8.491	MM	0.2531	4754.97900	313.05817	31.1770
2	9.214	MM	0.2793	1.04966e4	626.45020	68.8230

(*Z*)-5-benzyl-9-methyl-8-phenyl-5,9-dihydro-6H-benzo[2,3]azocino[5,4-*b*]indol-6-one (6)

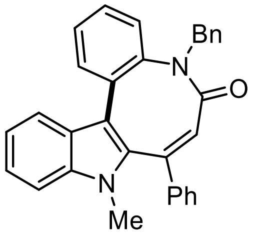

Synthetized from **5** (44 mg, 0.1 mmol), **L**$_7$AuCl (6.0 mg, 0.005 mmol), $AgSbF_6$ (1.7 mg, 0.005 mmol) and 1 mL of Ph-Cl. Purification by flash column chromatography (SiO_2, Hex/AcOEt 4:1) gave **6** (42 mg, 95% yield) as a white solid.

^{1}H NMR (400 MHz, DMSO): 7.52 (ddd, *J* = 8.0, 2.7, 1.2 Hz, 2H), 7.48 (dd, *J* = 7.5, 1.7 Hz, 1H), 7.41 – 7.26 (m, 6H), 7.25 (dt, *J* = 8.0, 1.0 Hz, 1H), 7.13 (ddd, *J* = 7.9, 6.9, 0.9 Hz, 1H), 7.11 – 7.03 (m, 2H), 6.94 (m, 1H), 6.90 (s, 1H), 6.75 (dd, *J* = 8.5, 6.9 Hz, 2H), 6.69 (dd, *J* = 8.1, 1.5 Hz, 2H), 5.22 (d, *J* = 15.2 Hz, 1H), 4.52 (d, *J* = 15.2 Hz, 1H), 3.33 (s, 3H).

^{13}C NMR (101 MHz, DMSO): 168.39 (C), 141.62 (C), 138.05 (C), 137.94 (C), 136.83 (C), 136.14 (C), 134.22 (C), 133.86 (C), 131.31 (CH), 129.43 (CH), 129.35 (CH), 128.36 (CH), 128.16 (CH), 128.11 (CH), 127.78 (CH), 127.38 (CH), 127.34 (CH), 127.05 (CH), 126.89 (CH), 125.94 (C), 123.33 (CH), 120.58 (CH), 119.75 (CH), 115.32 (C), 110.47 (CH), 53.04 (CH_2), 31.81 (CH_3).

HRMS (ESI) calculated for $C_{31}H_{24}N_2ONa$ $[M+Na]^+$ requires m/z = 463.1781, found m/z 463.1793.

R$_t$ (OD-H column, Hex/*i*-PrOH 80/20, 0.8 mL/min, 266.4 nm): tr(major) = 8.2 min, tr(minor) = 7.3 min, 50:50 e.r.

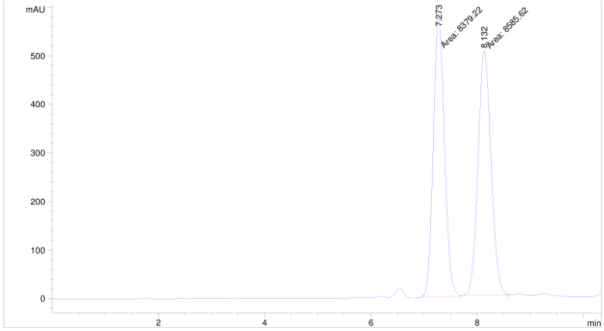

Peak #	RetTime [min]	Type	Width [min]	Area [mAU*s]	Height [mAU]	Area %
1	7.273	MM	0.2438	8379.21973	572.77014	49.3917
2	8.132	MM	0.2840	8585.62109	503.78625	50.6083

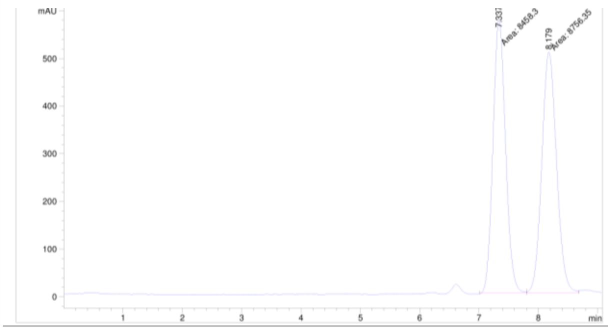

Peak #	RetTime [min]	Type	Width [min]	Area [mAU*s]	Height [mAU]	Area %
1	7.337	MM	0.2457	8458.29687	573.68610	49.1343
2	8.179	MM	0.2885	8756.35156	505.85257	50.8657

(*Z*)-5-benzyl-13-methyl-8-phenyl-5,13-dihydro-6*H*-benzo[2,3]azocino[4,5-*b*]indol-6-one (8)

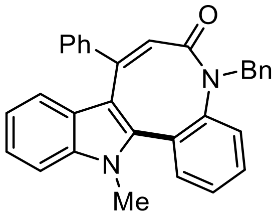

Synthetized from **7** (44 mg, 0.1 mmol), **L$_7$**AuCl (6.0 mg, 0.005 mmol), $AgSbF_6$ (1.7 mg, 0.005 mmol) and 1 mL of Ph-Cl. Purification by flash column chromatography (SiO_2, Hex/AcOEt 4:1) gave **8** (37 mg, 84% yield) as a white solid.

^{1}H NMR (400 MHz, DMSO): 7.71 (d, *J* = 8.1 Hz, 1H), 7.60 – 7.48 (m, 2H), 7.46 – 7.34 (m, 2H), 7.34 – 7.10 (m, 7H), 6.94 (dt, *J* = 10.1, 7.6 Hz, 3H), 6.77 (d, *J* = 7.9 Hz, 1H), 6.62 – 6.53 (m, 3H), 5.59 (d, *J* = 14.9 Hz, 1H), 4.28 (d, *J* = 14.9 Hz, 1H), 3.04 (s, 3H).

^{13}C NMR (101 MHz, DMSO): 168.93 (C), 142.25 (C), 139.50 (C), 138.90 (C), 137.58 (C), 137.06 (C), 137.03 (C), 131.37 (CH), 130.45 (CH), 129.90 (C), 128.92 (CH), 128.79 (CH), 128.42 (CH), 128.05 (CH), 128.03 (CH), 127.74 (CH), 127.52 (CH), 127.40 (CH), 125.75 (C), 123.23 (CH), 122.63 (CH), 120.22 (CH), 120.11 (CH), 113.77 (C), 110.75 (CH), 52.69 (CH_2), 30.61 (CH_3).

HRMS (ESI) calculated for $C_{31}H_{24}N_2ONa$ $[M+Na]^+$ requires m/z = 463.1781, found m/z 463.1790.

R$_t$ (OD-H column, Hex/*i*-PrOH 80/20, 0.8 mL/min, 266.4 nm): tr(major) = 10.1 min, tr(minor) = 8.3 min, 24:76 e.r.

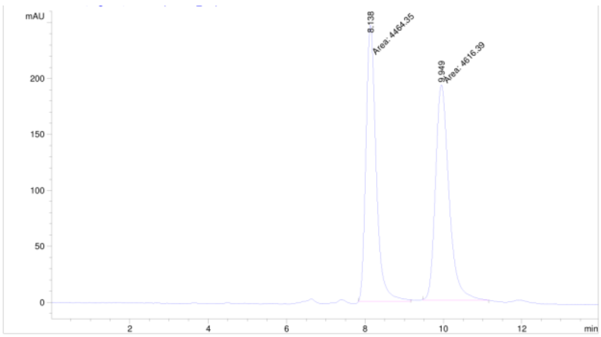

Peak #	RetTime [min]	Type	Width [min]	Area [mAU*s]	Height [mAU]	Area %
1	8.138	MM	0.3009	4464.34619	247.26408	49.1628
2	9.949	MM	0.3993	4616.38525	192.68591	50.8372

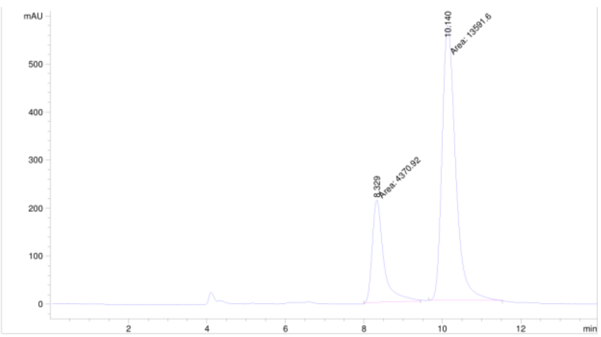

Peak #	RetTime [min]	Type	Width [min]	Area [mAU*s]	Height [mAU]	Area %
1	8.329	MM	0.3429	4370.91553	212.43823	24.3335
2	10.140	MM	0.3953	1.35916e4	573.02081	75.6665

(*S,Z*)-9-methyl-8-phenyl-6*H*-benzo[2,3][1,4]oxazocino[4,5-*a*]indol-6-one (10)

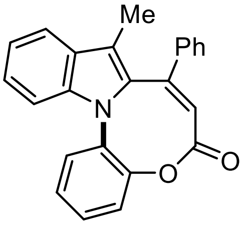

Synthetized from **9** (35 mg, 0.1 mmol), **L$_7$**AuCl (6.0 mg, 0.005 mmol), AgSbF$_6$ (1.7 mg, 0.005 mmol) and 1 mL of Ph-Cl. Purification by Biotage® Selekt (Hex/AcOEt 90:10 → 50:50) gave **8** (17 mg, 48% yield) as a white solid.

^{1}H NMR (300 MHz, CDCl$_3$): 7.62 (dt, *J* = 7.2, 1.3 Hz, 1H), 7.48 (dd, *J* = 7.9, 1.8 Hz, 1H), 7.44 – 7.38 (m, 2H), 7.37 – 7.28 (m, 6H), 7.24 – 7.17 (m, 2H), 6.46 (s, 1H), 1.90 (s, 3H).

^{13}C NMR (75 MHz, CDCl$_3$): 166.37 (C), 149.02 (C), 140.31 (C), 138.18 (C), 137.67 (C), 133.51 (C), 131.03 (C), 129.64 (C), 129.59 (CH), 129.34 (CH), 128.71 (CH), 128.60 (CH), 127.56 (CH), 127.28 (CH), 123.80 (CH), 122.50 (CH), 120.78 (CH), 119.41 (CH), 118.91 (CH), 114.55 (C), 110.91 (CH), 9.57 (CH$_3$).

HRMS (ESI) calculated for $C_{24}H_{17}NO_2Na$ $[M+Na]^+$ requires m/z = 374.1152, found m/z 374.1153.

$[\alpha]^D_{25}$ = +10 030.4 (c = 0.5, CHCl$_3$).

R$_t$ (OD-H column, Hex/*i*-PrOH 90/10, 0.8 mL/min, 310 nm): tr(major) = 11.6 min, tr(minor) = 8.3 min, 33:67 e.r.

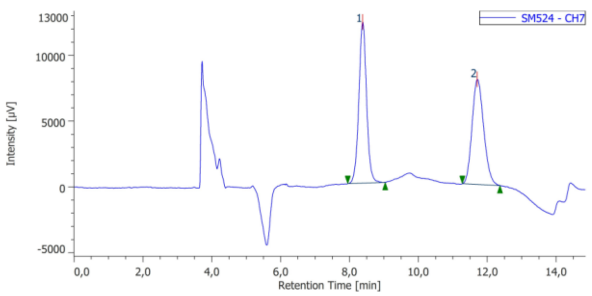

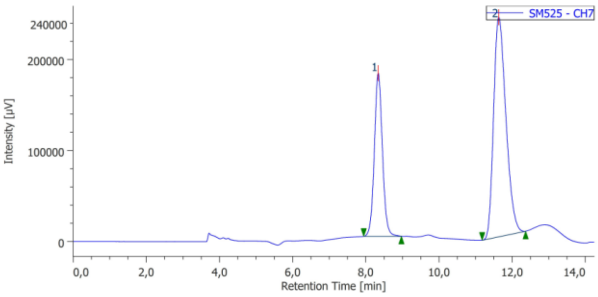

#	Peak Name	CH	tR [min]	Area [µV·sec]	Height [µV]	Area%
1	Unknown	7	8,380	190670	12232	50,410
2	Unknown	7	11,707	187566	7978	49,590

#	Peak Name	CH	tR [min]	Area [µV·sec]	Height [µV]	Area%
1	Unknown	7	8,333	2745073	179319	32,778
2	Unknown	7	11,630	5629680	241006	67,222

Transformations of the final products

(*S,Z*)-5-benzyl-9-methyl-8-phenyl-5,6-dihydrobenzo[2,3][1,4]diazocino[1,8-*a*]indole (11)

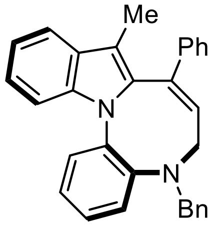

Under nitrogen atmosphere, **2aa** (44 mg, 0.1 mmol) was dissolved in 1 mL of THF and the solution was cooled down to 0°C. Then $LiAlH_4$ (9.5 mg, 0.25 mmol) and anhydrous $AlCl_3$ (6.7 mg, 0.05 mmol) were added and the solution was stirred at 50 °C for 2 hours. Then water was added, and the salts were filtered. The water phase was extracted with AcOEt, and the organic phases were combined and dried over anhydrous sodium sulfate. The organic phase was concentrated under reduced pressure to afford **11** as a white solid (42 mg,96% yield).

^{1}H NMR (400 MHz, CD_2Cl_2): 7.63 (m, 1H), 7.43 – 7.36 (m, 2H), 7.36 – 7.30 (m, 3H), 7.29 – 7.12 (m, 9H), 7.08 – 6.98 (m, 2H), 6.92 (ddd, *J* = 7.8, 7.1, 1.4 Hz, 1H), 6.37 (dd, *J* = 8.7, 6.9 Hz, 1H), 4.56 (q, *J* = 16.1 Hz, 2H), 4.06 (dd, *J* = 13.0, 8.7 Hz, 1H), 3.65 (dd, *J* = 13.0, 6.9 Hz, 1H), 2.00 (s, 3H).

^{13}C NMR (101 MHz, CD_2Cl_2): 147.03 (C), 139.49 (C), 139.11 (C), 139.07 (C), 139.02 (C), 136.42 (C), 130.19 (CH), 129.96 (C), 129.53 (C), 128.86 (CH), 128.42 (CH), 127.97 (CH), 127.11 (CH), 126.88 (CH), 126.80 (CH), 126.70 (CH), 122.07 (CH), 121.23 (CH), 120.54 (CH), 119.52 (CH), 118.42 (CH), 111.26 (CH), 59.36 (CH_2), 52.61 (CH_2), 9.00 (CH_3). One C and one CH are missing, probably overlapped.

HRMS (ESI) calculated for $C_{31}H_{27}N_2$ $[M+H]^+$ requires m/z = 427.2174, found m/z 427.2173.

$[\alpha]^D_{25}$ = +628.8 (c = 0.25, $CHCl_3$).

R_t (OD-H column, Hex/*i*-PrOH 98/2, 0.8 mL/min, 266.4 nm): tr(major) = 7.8 min, tr(minor) = 7.3 min, 2:98 e.r.

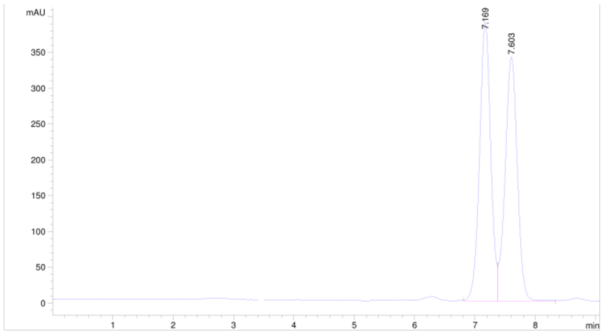

Peak #	RetTime [min]	Type	Width [min]	Area [mAU*s]	Height [mAU]	Area %
1	7.169	BV	0.1983	5027.85645	390.37512	51.1921
2	7.603	VB	0.2158	4793.68457	341.38568	48.8079

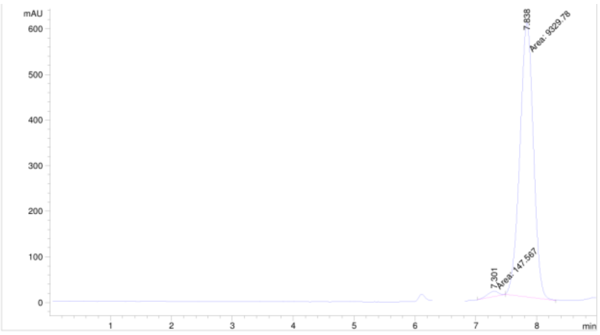

Peak #	RetTime [min]	Type	Width [min]	Area [mAU*s]	Height [mAU]	Area %
1	7.301	MM	0.2081	147.56688	11.81622	1.5570
2	7.838	MM	0.2584	9329.78125	601.70428	98.4430

(*S,Z*)-5-benzyl-9-methyl-8-phenylbenzo[2,3][1,4]diazocino[1,8-a]indole-6(5*H*)-thione (12)

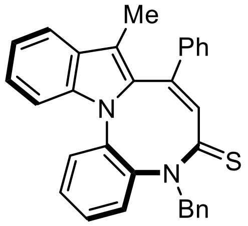

A mixture of **2aa** (35.2 mg, 0.08 mmol) and Lawesson reagent (19.4 mg, 0.048 mmol) in 1.3 mL of toluene was heated under inert atmosphere at 110°C for 2 hours. Then the solvent was removed under vacuum. Purification of the crude by column Biotage® Selekt (Hex/AcOEt 98:2 → 80:20) yielded **12** (36.5 mg, 100%) as a yellow solid.

^{1}H NMR (400 MHz, $CDCl_3$): 7.59 (d, *J* = 7.9 Hz, 1H), 7.46 – 7.30 (m, 4H), 7.28 (d, *J* = 4.3 Hz, 5H), 7.18 (ddd, *J* = 8.0, 7.0, 1.0 Hz, 1H), 7.09 – 6.92 (m, 3H), 6.76 (d, *J* = 4.4 Hz, 4H), 6.53 (d, *J* = 8.2 Hz, 1H), 6.40 (d, *J* = 14.3 Hz, 1H), 4.71 (d, *J* = 14.3 Hz, 1H), 1.96 (s, 3H).

^{13}C NMR (101 MHz, $CDCl_3$): 199.35 (C), 141.37 (C), 139.02 (C), 137.31 (C), 135.40 (C), 134.06 (C), 133.58 (C), 131.26 (CH), 129.80 (C), 129.28 (CH), 129.25 (C), 128.46 (CH), 128.35 (CH), 128.32 (CH), 128.20 (CH), 128.16 (CH), 128.08 (CH), 127.65 (CH), 127.46 (CH), 126.15 (CH), 122.83 (CH), 120.31 (CH), 118.78 (CH), 113.08 (C), 110.74 (CH), 59.23 (CH_2), 9.49 (CH_3).

HRMS (ESI) calculated for $C_{31}H_{24}N_2NaS$ $[M+Na]^+$ requires m/z = 479.1558, found m/z 479.1556.

$[\alpha]^D_{25}$ = +457.2 (c = 0.25, $CHCl_3$).

R_t (OD-H column, Hex/*i*-PrOH 95:5, 0.8 mL/min, 266.4 nm): tr(major) = 11.3 min, tr(minor) = 8.7 min, 3:97 e.r.

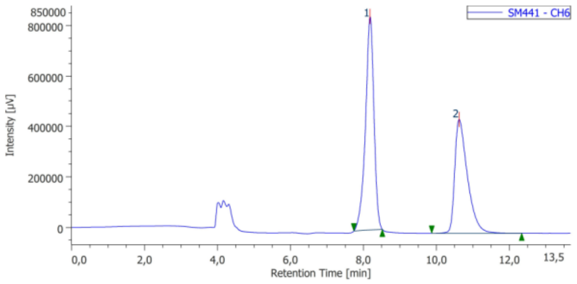

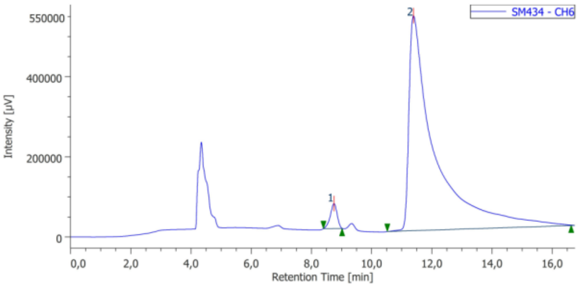

#	Peak Name	CH	tR [min]	Area [µV·sec]	Height [µV]	Area%
1	Unknown	6	8,180	13643352	845575	55,684
2	Unknown	6	10,620	10857886	449585	44,316

#	Peak Name	CH	tR [min]	Area [µV·sec]	Height [µV]	Area%
1	Unknown	6	8,747	974608	62219	2,947
2	Unknown	6	11,387	32094733	535993	97,053

(*S,Z*)-4-(5-benzyl-9-methyl-6-oxo-5,6-dihydrobenzo[2,3][1,4]diazocino[1,8-*a*]indol-8-yl)benzoic acid (2bb)

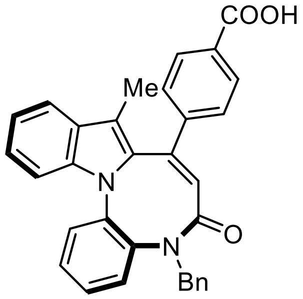

Under nitrogen atmosphere, **2ag** (20.0 mg, 0.04 mmol) was dissolved in 0.4 mL THF. Then a solution of LiOH (3.4 mg, 0.08 mmol) in 0.2 mL of water was added and the mixture was left stirring for 24 hours. Then HCl 1M was added and the acqueous phase was extracted three times with AcOEt. The combined organic phases were dried with anhydrous sodium sulfate and concentrated under reduced pressure to yield **2bb** as a yellow solid (19.6 mg, 98%), without further purification.

^{1}H NMR (400 MHz, $CDCl_3$): 8.04 – 7.97 (m, 2H), 7.59 (dt, *J* = 7.9, 1.0 Hz, 1H), 7.51 – 7.40 (m, 2H), 7.40 – 7.32 (m, 4H), 7.18 (ddd, *J* = 8.0, 7.0, 1.0 Hz, 1H), 7.07 (ddd, *J* = 8.2, 7.0, 1.2 Hz, 1H), 6.95 (m, 1H), 6.77 (t, *J* = 7.7 Hz, 2H), 6.71 (d, *J* = 7.7 Hz, 3H), 6.56 (dt, *J* = 8.3, 0.9 Hz, 1H), 5.56 (d, *J* = 14.5 Hz, 1H), 4.39 (d, *J* = 14.6 Hz, 1H), 1.94 (s, 3H).

^{13}C NMR (101 MHz, $CDCl_3$): 170.87 (C), 167.94 (C), 144.19 (C), 139.52 (C), 137.64 (C), 135.89 (C), 135.42 (C), 135.12 (C), 133.77 (C), 130.40 (CH), 129.31 (C), 128.87 (C), 128.75 (CH), 128.68 (CH), 128.38 (CH), 128.16 (CH), 128.01 (CH), 127.63 (CH), 127.37 (CH), 127.32 (CH), 126.82 (CH), 123.18 (CH), 120.41 (CH), 118.84 (CH), 113.11 (C), 111.03 (CH) , 52.53 (CH_2), 9.62 (CH_3).

HRMS (ESI) calculated for $C_{32}H_{23}N_2O_3Na$ $[M+Na]^+$ requires m/z = 483.1709, found m/z 483.1708.

R_t (OD-H column, Hex/*i*-PrOH 80:20 + 0.1% HCOOH, 0.8 mL/min, 266.4 nm): tr(major) = 12.3 min, tr(minor) = 9.0 min, 3:97 e.r.

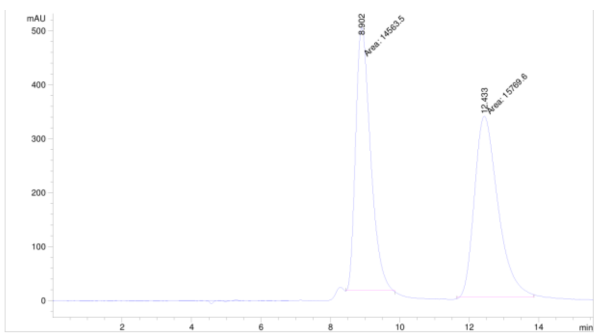

Peak #	RetTime [min]	Type	Width [min]	Area [mAU*s]	Height [mAU]	Area %
1	8.902	MM	0.4978	1.45635e4	487.62900	48.0119
2	12.433	MM	0.7845	1.57696e4	335.02237	51.9881

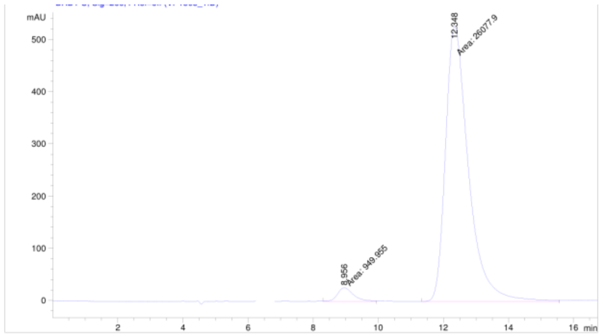

Peak #	RetTime [min]	Type	Width [min]	Area [mAU*s]	Height [mAU]	Area %
1	8.956	MM	0.5927	949.95502	26.71444	3.5147
2	12.348	MM	0.8235	2.60779e4	527.78174	96.4853

methyl (4-((*S,Z)*-5-benzyl-9-methyl-6-oxo-5,6-dihydrobenzo[2,3][1,4]diazocino[1,8-*a*]indol-8-yl)benzoyl)-*L*-phenylalanyl-*L*-phenylalaninate (13)

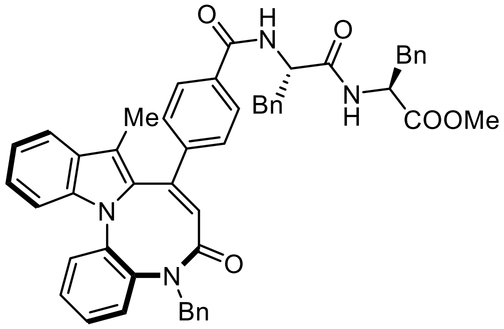

A mixture of **2bb** (19.6 mg, 0.040 mmol), methyl *L*-phenylalanyl-*L*-phenylalaninate (19.3 mg, 0.044 mmol), EDCI (9,2 mg, 0.48 mmol) and DMAP (1.0 mg, 0.008 mmol) in 0.8 mL of DCM were stirred for 48 hours at room temperature under nitrogen atmosphere. After the completion of the reaction, a solution of NH_4Cl was added and the water phase was extracted three times with DCM. The organic phases were collected, dried over Na_2SO_4 and concentrated under reduced pressure. Purification of the crude by columne Biotage® Selekt (Hex/AcOEt 93:7 → 30:70) yielded **13** (10.4 mg, 33%) as a white solid.

^{1}H NMR (400 MHz, $CDCl_3$): 7.61 – 7.55 (m, 3H), 7.49 – 7.38 (m, 2H), 7.37 – 7.29 (m, 5H), 7.26 – 7.22 (m, 4H), 7.21 – 7.14 (m, 4H), 7.07 (ddd, *J* = 8.2, 7.0, 1.2 Hz, 1H), 6.96 (ddd, *J* = 9.5, 6.0, 4.7 Hz, 3H), 6.77 (t, *J* = 7.7 Hz, 2H), 6.73 – 6.65 (m, 4H), 6.56 (dd, *J* = 8.3, 1.1 Hz, 1H), 6.22 (d, *J* = 7.6 Hz, 1H), 5.55 (d, *J* = 14.5 Hz, 1H), 4.85 – 4.72 (m, 2H), 4.38 (d, *J* = 14.5 Hz, 1H), 3.72 (s, 3H), 3.32 – 2.91 (m, 4H), 1.94 (s, 3H).

^{13}C NMR (101 MHz, $CDCl_3$): 171.24, (C) 170.24 (C), 167.85 (C), 166.38 (C), 142.38 (C), 139.63 (C), 137.63 (C), 136.31 (C), 135.89 (C), 135.48 (C), 135.25 (C), 135.20 (C), 133.83 (C), 133.62 (C), 129.39 (CH), 129.12 (CH), 128.87 (C), 128.74 (CH), 128.67 (CH), 128.57 (CH), 128.37 (CH), 128.15 (CH), 127.99 (CH), 127.62 (CH), 127.47 (CH), 127.29 (CH), 127.16 (CH), 127.12 (CH), 126.38 (CH), 123.14 (CH), 120.38 (CH), 118.83 (CH), 112.99 (C), 111.03 (CH), 54.54 (CH_3), 53.45 (CH), 52.47 (CH_2), 52.37 (CH), 38.07 (CH_2), 37.85 (CH_2), 9.66 (CH_3). Two CH are missing, probably overlapped.

HRMS (ESI) calculated for $C_{51}H_{44}N_4O_5Na$ $[M+Na]^+$ requires m/z = 815.3209, found m/z 815.3215.

[α]^D$_{25}$ = +175.7 (c = 0.25, $CHCl_3$).

(*R,Z*)-5-benzyl-13-(diphenylphosphoryl)-9-methyl-8-phenylbenzo[2,3][1,4]diazocino[1,8-*a*]indol-6(5*H*)-one (14)

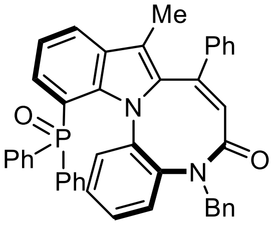

2au (31 mg, 0.06 mmol), diphenylphosphine oxide (24 mg, 0.12 mmol), $Pd(OAc)_2$ (1.3 mg, 0.006 mmol) and dppb (2.6 mg, 0.006 mmol) were dissolved in DMSO, which was previously degassed, through three freez-pump cycles. Then *i*Pr_2NEt (52 µl, 0.3 mmol) was added and the mixture was heated at 120 °C for 24 h. After the completion of the reaction, HCl 1 N was added, and the suspension was extracted three times with AcOEt. The combined organic phases were washed with $NaHCO_3$ ss and brine, dried with Na_2SO_4 and concentered under reduced pressure. Purification of the crude by colum Biotage® Selekt (Hex/AcOEt 92:8 → 34:66) yielded **14** (29.2 mg, 76%) as a yellow solid.

^{1}H NMR (300 MHz, $CDCl_3$): 7.80 (ddd, *J* = 7.8, 2.1, 1.2 Hz, 1H), 7.56 (td, *J* = 6.2, 2.1 Hz, 1H), 7.51 – 7.39 (m, 5H), 7.32 (td, *J* = 7.6, 3.1 Hz, 4H), 7.27 – 7.08 (m, 11H), 7.07 – 6.95 (m, 2H), 6.89 (ddd, *J* = 15.9, 7.4, 1.2 Hz, 1H), 6.48 (s, 1H), 6.37 – 6.25 (m, 2H), 5.41 (d, *J* = 15.1 Hz, 1H), 5.24 (d, *J* = 15.1 Hz, 1H), 1.90 (s, 3H).

^{13}C NMR (75 MHz, $CDCl_3$): 168.34 (C), 142.27 (C), 139.50 (d, *J* = 3.0 Hz, C), 139.30 (C), 138.62 (C), 137.93 (C), 137.64 (C), 135.30 (C), 135.04 (d, *J* = 210.5 Hz, C), 133.66 (d, *J* = 207.0 Hz, C), 133.65 (C), 131.96 (CH), 131.94 (d, *J* = 21.7 Hz, CH), 131.60 (CH), 131.57 (CH), 131.36 (CH), 128.45 (CH), 128.38, 128.31 (CH), 128.24 (CH), 128.14 (CH), 128.07 (CH), 128.02 (CH), 127.31 (CH), 127.26 (CH), 126.65 (d, *J* = 3.5 Hz, CH), 126.53 (CH), 123.45 (d, *J* = 2.8 Hz, CH), 119.07 (d, *J* = 12.7 Hz, CH), 115.75 (C), 114.19 (C). 113.19 (C), 51.97 (CH_2), 9.64 (CH_3).

^{31}P NMR (162 MHz, $CDCl_3$): 31.28.

HRMS (ESI) calculated for $C_{43}H_{33}N_2O_2PNa$ $[M+Na]^+$ requires m/z = 663.2172, found m/z 663.2173.

$[\alpha]^D_{25}$ = +228.9 (c = 0.25, $CHCl_3$).

R_t (OD-H column, Hex/*i*-PrOH 95:5, 0.8 mL/min, 310 nm): tr(major) =29.4 min, tr(minor) = 32.0 min, 1:99 e.r.

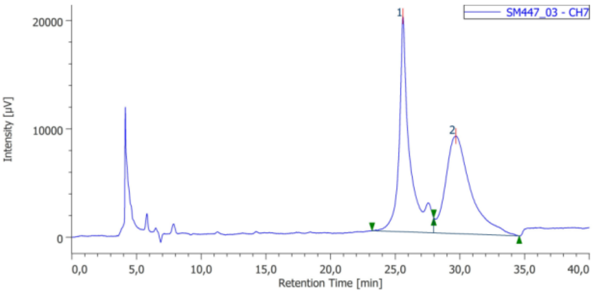

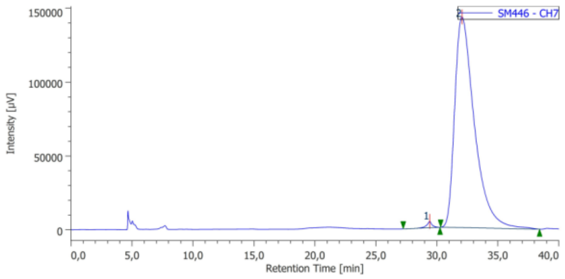

#	Peak Name	CH	tR [min]	Area [µV·sec]	Height [µV]	Area%	Height%
1	Unknown	7	25,590	1116412	19877	47,908	68,811
2	Unknown	7	29,677	1213906	9009	52,092	31,189

#	Peak Name	CH	tR [min]	Area [µV·sec]	Height [µV]	Area%	Height%
1	Unknown	7	29,407	126354	4259	0,782	2,903
2	Unknown	7	32,060	16030314	142481	99,218	97,097

Computational studies

Methods

DFT calculations. Geometry optimizations, vibrational analyses and single point (SP) energy calculations were performed using the Gaussian 16 software.[19] The enantiomerization energy of reference compound **2a**[20] was initially calculated to test suitable computational protocols to be used in this study. Reference compounds **2a** and **2a**-**TS** were optimized at the M06[21]/def2-SVP level using the SMD[22] solvent model for mesitylene and the "ultrafine" integration grid, and vibrational analysis was performed on optimized geometries at the same level. Single point (SP) energies were then calculated at the PBE1PBE[23]/TZ2P level, using the GD3BJ empirical correction for dispersive interactions.[24] A free energy of enantiomerization ($\Delta G^{\ddagger}_{enantiomerization}$) of 29.9 kcal/mol (Table S5) was obtained, a value that is comparable to those calculated ($\Delta G^{\ddagger}_{enantiomerization}$ = 29.8 kcal/mol) and measured ($\Delta G^{\ddagger}_{enantiomerization}$ = 29.9 kcal/mol) in reference.[20] However, since the adopted theoretical level was too computational demanding for our subsequent mechanistic study, a different protocol was evaluated. Geometries of reference **2a** and **2a-TS** were optimized in the gas phase at the M06/LANL2DZ[25] level with the "ultrafine" integration grid. Vibrational analyses were then performed at the same level and SP energies were computed at the PBE1PBE-GD3BJ/TZ2P level, using the CPCM[26] solvent model for mesitylene. A $\Delta G^{\ddagger}_{enantiomerization}$ = 30.0 kcal/mol (Table S5) was obtained, with a considerable reduction of computational time. The CPCM-PBE1PBE-GD3BJ/TZ2P//M06/LANL2DZ protocol was then adopted for all the other DFT calculations described herein.

The enantiomerization energy of compound **2aa** synthetized here was also evaluated. The initial conformation of **2aa** was taken from the X-ray coordinates, while three alternative conformations were generated for the enantiomerization transition state (TS), hereafter referred as **TS-2aa**. All geometries were optimized, frequency were calculated, and SP energy were evaluated using the protocol described above and toluene as the solvent. Two conformations converged to the same structure, so energies of the **TS-2aa** – Conformations 1 and 2 are reported in Table S6.

A mechanistic analysis of the catalytic cycle leading to **2aa** was done. An initial conformational search was performed for reactant **1aa** and the cationic $\mathbf{L_7}$AuCl using the MMFF94x force field in the gas phase, as implemented in the MOE software package.[27] The lowest-energy conformers (28 for **1aa**, 10 for $\mathbf{L_7}$AuCl) were selected for subsequent DFT optimization at the CPCM-PBE1PBE-GD3BJ/TZ2P//M06/LANL2DZ level, using chlorobenzene as the solvent. The SbF_6^- counterion was added to each conformer of the cationic s prior to the DFT calculations. The lowest-energy geometries of both the substrate and catalyst were used to manually construct an initial set of conformations for intermediates **II** and **III**, as the presence of the phosphorous-Au-carbon bonds precluded an automated conformational search. Multiple initial geometries were built for each intermediate in both atropisomeric configurations. DFT optimization of these structures yielded a final set of 20 conformers for intermediate *(S)*-**II**, 5 for *(R)*-**II**, 12 for *(S)*-**III**, and 3 for *(R)*-**III**. Further manual searching via systematic dihedral angle variation failed in identifying other low-energy structures. Product **2aa** was optimized at the same level, starting from the crystallographic geometry described in this article. To achieve a correct stoichiometry in the computation of relative energies, the $HSbF_6$ and SbF_6^- species were also optimized at the same level of theory. The nature of all stationary points as true energy minima was confirmed by frequency calculations at the same level of theory. All the absolute energies and thermochemical corrections are listed in Table S7. The lowest-energy minima of each compound were used to compute relative Gibbs free energies (ΔG). The energies of the isolated atropisomeric intermediates **II** and **III** were adjusted by adding the energy of the SbF_6^- counterion or the $HSbF_6$ acid, respectively. However, to assess the influence of the counterion, the geometries of the intermediate **II** atropisomers were reoptimized with the SbF_6^- counterion explicitly included. The SbF_6^- counterion was initially placed near the most acidic proton (the presumed site of abstraction leading to intermediate **III**) of the lowest-energy geometry, and various starting orientations were tested during reoptimization. The results from the explicit counterion model were comparable to those from the isolated ion calculations. The computed ΔΔG between the two atropisomers, favouring *(R)*, dropped from 0.9 to 0.2 kcal/mol. Although this result is slightly closer to the experimental outcome, the simpler isolated-ion model was retained for the mechanistic discussion. This decision was made to keep the model as simple as possible for subsequent QTAIM analysis. Additionally, despite careful setup,

the vast conformational space of the ion pair makes it computationally challenging to guarantee that a true global minimum was located.

QTAIM analysis. The Quantum Theory of Atoms in Molecules (QTAIM)[28] analysis was performed using the Multiwfn 3.8 program[29] on the lowest energy compounds optimized previously. A wavefunction file was produced during the SP calculations described above and loaded into Multiwfn. A topological analysis was then conducted by searching for BCPs starting from atomic nuclei, midpoints of atomic pairs, and the centres of triangles and pyramids formed by atoms, ensuring the location of all CPs. The analysis focused on nuclear attractors (3,−3), CPs corresponding to nuclear positions, and Bond Critical Points (BCPs; 3,-1), that are found along the path of maximum electron density between two nuclei and are characteristic of both covalent and non-covalent bonding interactions. The paths connecting the (3,−3) and (3,−1) CPs were generated to visualize the complete molecular graph. Finally, several key topological parameters were calculated at each non-covalent BCP to characterize the nature and strength of the interactions. The most relevant parameters for this analysis are electron density (ρ), that measures the amount of electron charge at each BCP. For similar types of non-covalent interactions, a higher value of ρ indicates a greater accumulation of charge between the atoms and thus a stronger interaction. Laplacian of Electron Density ($\nabla^2\rho$); the sign of ($\nabla^2\rho$) indicates the nature of the interaction. For the non-covalent interactions studied here, a $\nabla^2\rho > 0$ is expected. Potential Energy Density (V(r)); this is always a negative (stabilizing) value. A more negative V(r) corresponds to a greater stabilization from the interaction, providing a direct measure of its strength. Total Energy Density (H(r)); this is the sum of the kinetic and potential energy densities at the BCP. While a negative value (H(r) < 0) would indicate significant covalent character, for the weak interactions analysed here, a positive H(r) is expected. In these cases, a smaller positive value suggests a more stabilizing interaction.

DFT and QTAIM study

Table S5: Absolute (a.u.) and relative (kcal/mol) energies for the isomerization of the reference compound **2a** described in *Angew. Chem. Int. Ed.,* 2018, **57**, 11040 –11044, using two alternative theoretical levels.

	E(SCF)	ZPEc	Hc	Gc	E(SCF)+ZPE	E(SP)	E(SP)+ZPE	E(SP)+Hc	E(SP)+Gc	ΔE	ΔH	ΔG
					**SMD-PBE1PBE-GD3BJ/Def2TZVP//SMD-M06/def2-SVP (solvent = mesytilene)**							
**2a**	–1439.85699	0.510874	0.540256	0.451211	–1439.34612	–1440.768081	–1440.257207	–1440.227825	–1440.31687	0.0	0.0	0.0
**2a-TS**	–1439.81276	0.510867	0.539228	0.453483	–1439.30189	–1440.722708	–1440.211841	–1440.18348	–1440.269225	28.5	27.8	29.9
					**CPCM-PBE1PBE-GD3BJ/Def2TZVP//M06/LANL2DZ (solvent = mesytilene)**							
**2a**	–1440.5968	0.517795	0.546924	0.457949	–1440.079005	–1440.763902	–1440.246107	–1440.216978	–1440.305953	0.0	0.0	0.0
**2a-TS**	–1440.551429	0.517658	0.545735	0.460336	–1440.033771	–1440.71848	–1440.200822	–1440.172745	–1440.258144	28.4	27.8	30.0

Table S6: Absolute (a.u.) and relative (kcal/mol) energies for the isomerization of the compound **2aa** synthetized here.

	E(SCF)	ZPEc	Hc	Gc	E(SCF)+ZPE	E(SP)	E(SP)+ZPE	E(SP)+Hc	E(SP)+Gc	ΔE	ΔH	ΔG
					**CPCM-PBE1PBE-GD3BJ/Def2TZVP//M06/LANL2DZ (solvent = toluene)**							
**2aa**	–1440.5968	0.517795	0.546924	0.457949	–1440.079005	–1440.763902	–1440.246107	–1440.216978	–1440.305953	0.0	0.0	0.0
**TS-2aa** – Conformation 1	–1379.22781	0.47054	0.497488	0.412812	–1378.75727	–1379.386304	–1378.915764	–1378.888816	–1378.973492	36.7	36.4	36.9
**TS-2aa** – Conformation 2	–1379.22908	0.471193	0.497639	0.41663	–1378.757887	–1379.387802	–1378.916609	–1378.890163	–1378.971172	36.2	35.5	38.3

Table S7: Absolute energies and correction (a.u.) for the lowest-energy conformations of reactants, intermediates and products of the catalytic cycle leading to product **2aa**, obtained at the CPCM-PBE1PBE-GD3BJ/Def2TZVP//M06/LANL2DZ level (solvent = chlorobenzene) as the solvent.

	E(SCF)	ZPEc	Hc	Gc	E(SCF)+ZPE	E(SP)	E(SP)+ZPE	E(SP)+Hc	E(SP)+Gc
**1aa**	–1379.22355	0.468369	0.497393	0.405091	–1378.755181	–1379.383894	–1378.915525	–1378.886501	–1378.9788
**AuL·SbF$_6$**	–4149.587553	0.764422	0.833990	0.656246	–4148.823131	–4719.448120	–4718.683698	–4718.614130	–4718.791874
**SbF$_6^-$**	–604.563143	0.012611	0.022087	–0.021131	–604.550532	–839.3564359	–839.3438249	–839.3343489	–839.3775669
**SbF$_6$H**	–604.970408	0.022377	0.033106	–0.012939	–604.948031	–839.7293541	–839.7069771	–839.6962481	–839.7422931
**Int (*S*)-II**	–4924.195639	1.222919	1.309410	1.100166	–4922.972720	–5259.520107	–5258.297188	–5258.210697	–5258.419941
**Int (*R*)-II**	–4924.199284	1.222987	1.309564	1.099302	–4922.976297	–5259.520629	–5258.297642	–5258.211065	–5258.421327
**Int (*S*)-III**	–4923.825000	1.210533	1.297408	1.084126	–4922.614467	–5259.119542	–5257.909009	–5257.822134	–5258.035416
**Int (*R*)-III**	–4923.83256	1.210906	1.297309	1.087311	–4922.621654	–5259.119335	–5257.908429	–5257.822026	–5258.03202
**2aa**	–1379.285039	0.470959	0.498525	0.413035	–1378.81408	–1379.449212	–1378.978253	–1378.950687	–1379.03618

Detailed discussion on QTAIM results

The analysis focused on localising (3,−1) bond critical points (BCPs). BCPs associated with NCIs are characterised by positive values for both the Laplacian of electron density ($\nabla^2\rho$) and the total energy density (H(r)). The electron density (ρ) and the potential energy density (V(r)) at the BCP quantify the interactions strength. QTAIM results are summarised in Tables S8 (intermediates **II**) and S9 (intermediates **III**), while the full list of non-covalent BCPs and their description is provided in Tables S10-S13. Optimised geometries with labelled BCPs are presented in Figures S1-S4.

The QTAIM analysis of intermediate (*S*)-**II** reveals that this is the more stabilised isomer by NCIs. Indeed, it features a more extensive network of 36 NCIs compared to 30 found for (*R*)-**II** (Table S8, Figures S1, S2). This enhanced stabilisation is quantitatively demonstrated by its more negative total potential energy density (ΣV(r) = −0.1452 a.u.) and higher total electron density (Σρ = 0.2573 a.u.) relative to (*R*)-**II** (ΣV(r) = −0.1065 a.u., Σρ = 0.1948 a.u.). The superior stability of the (*S*)-**II** NCIs network is a result of several key factors. Considering the central Au atom of the catalyst, (*S*)-**II** benefits from a unique and stabilizing Au···H interaction, not present in the other isomer. Concerning classical O···H, and F···H and weak C···H interactions, (*S*)-**II** exhibits a more robust NCI network. Its C···H interactions show a greater accumulation of electron density (Σρ = 0.0737 a.u. vs. 0.0438 a.u.) and are substantially more stabilising (ΣV(r) = −0.0379 a.u. vs. −0.0237 a.u.). The same trend holds for O···H interactions (Σρ = 0.0441 a.u., ΣV(r) = −0.0280 a.u.; Σρ = 0.0364 a.u. ΣV(r) = −0.0209 a.u., for (*S*)-**II** and (*R*)-**II**, respectively). While (*R*)-**II** has a slightly more stabilising F···H network (Σρ = 0.0295 a.u., ΣV(r) = −0.0162 a.u.; Σρ = 0.0381 a.u. ΣV(r) = −0.0224 a.u., for (*S*)- and (*R*)-**II**, respectively), it is not enough to compensate for the deficits elsewhere. Moreover, (*S*)-**II** displays a more extensive network of intramolecular C···C contacts that are also cumulatively stronger (ΣV(r) = −0.0116 vs. −0.0101 for (*R*)-**II**). This suggests that the carbon framework of (*S*)-**II** adopts a more compact and well-organised conformation stabilised by London dispersion forces. Conversely, (*R*)-**II** exhibits significantly stronger H···H interactions (ΣV(r) = −0.0101 a.u. vs. −0.0063 a.u. for (*S*)-**II**). While these can be stabilising, a high value can also indicate steric hindrance where hydrogen atoms are forced into close proximity, leading to repulsive effects that are not fully captured by the ΣV(r) alone but might contribute to overall strain.[30] Finally, two distinct F···O interactions are only found in (*S*)-**II**. NCIs between F and similarly electronegative atoms has been controversial,[31]but more recent literature provided crystallographic evidence of an intramolecular C–F···O=C contact observed in F-substituted benzoyl chlorides.[32] In our case these interactions, with a combined ΣV(r) = −0.0054 a.u., might act as directional anchor points, locking the substrate into a favourable orientation relative to the catalyst.

Table S8. NCIs identified by QTAIM analysis on intermediates **II**. Σρ and ΣV(r) are the sum of the total electron density (ρ) and the potential energy density V(r) for each NCI, given in a.u.

	**Intermediate (*S*)-II**			**Intermediate (*R*)-II**		
**Type**	**Count**	**Σρ**	**ΣV(r)**	**Count**	**Σρ**	**ΣV(r)**
Au···C	1	0.0117	−0.0066	1	0.0079	−0.0043
Au···H	1	0.0127	−0.0078	0	0.0000	0.0000
C···C	1	0.0252	−0.0116	3	0.0207	−0.0101
C···F	4	0.0118	−0.0059	1	0.0064	−0.0042
C···H	10	0.0737	−0.0379	9	0.0498	−0.0237
C···O	2	0.0265	−0.0194	2	0.0162	−0.0108
F···H	6	0.0295	−0.0162	8	0.0381	−0.0224
F···O	2	0.0093	−0.0054	0	0.0000	0.0000
H···H	3	0.0128	−0.0063	2	0.0192	−0.0101
O··· H	4	0.0441	−0.0280	4	0.0364	−0.0209
Total	36	0.2573	−0.1452	30	0.1948	−0.1065

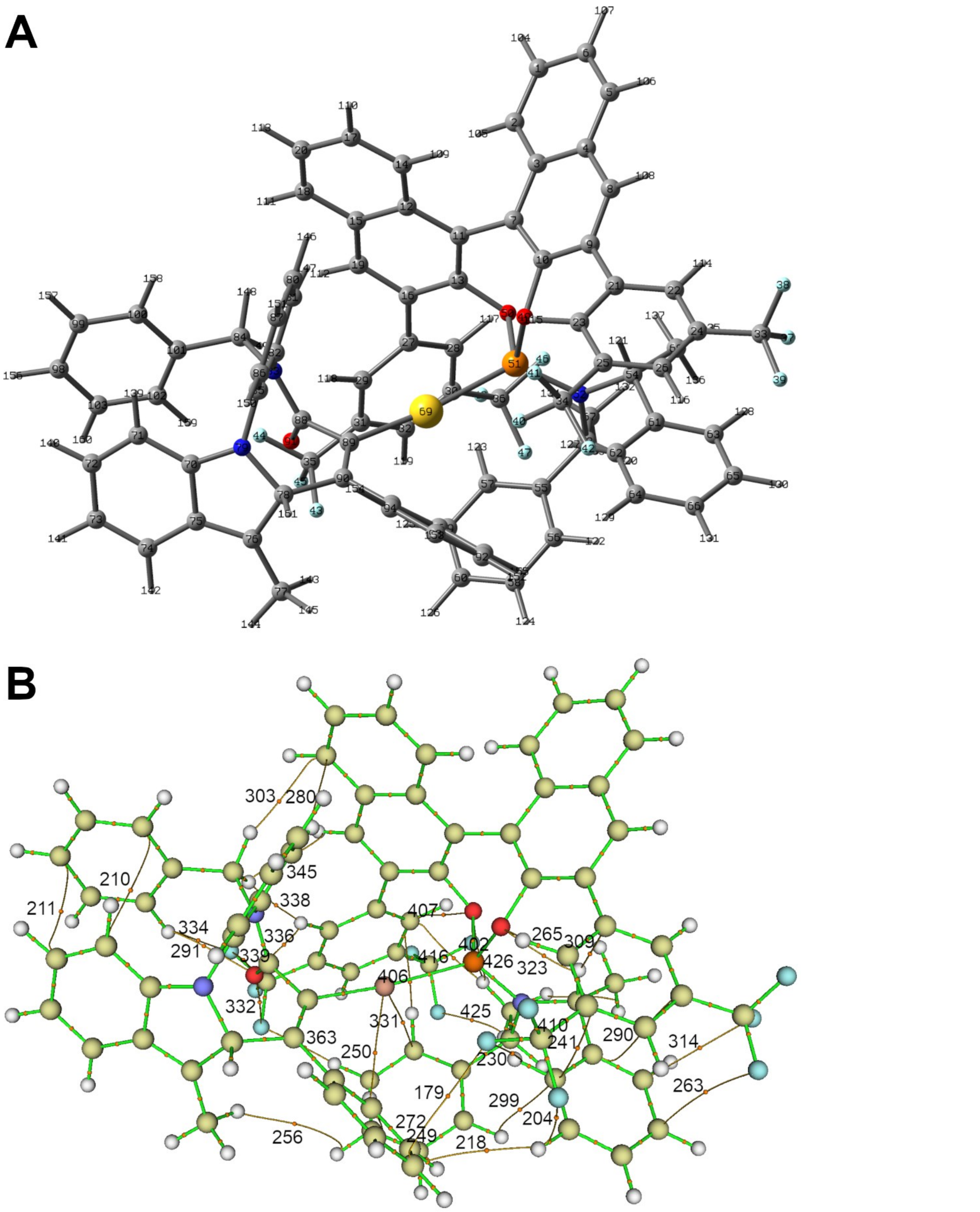

Figure S1: DFT optimized geometry and (3, –1) BCPs for intermediate *(S)*-**II**. (A) Molecular geometry with atom labelling. (B) (3, –1) BCPs of identified NCIs (labelled by BCP number), and corresponding bond paths, obtained by QTAIM analysis.

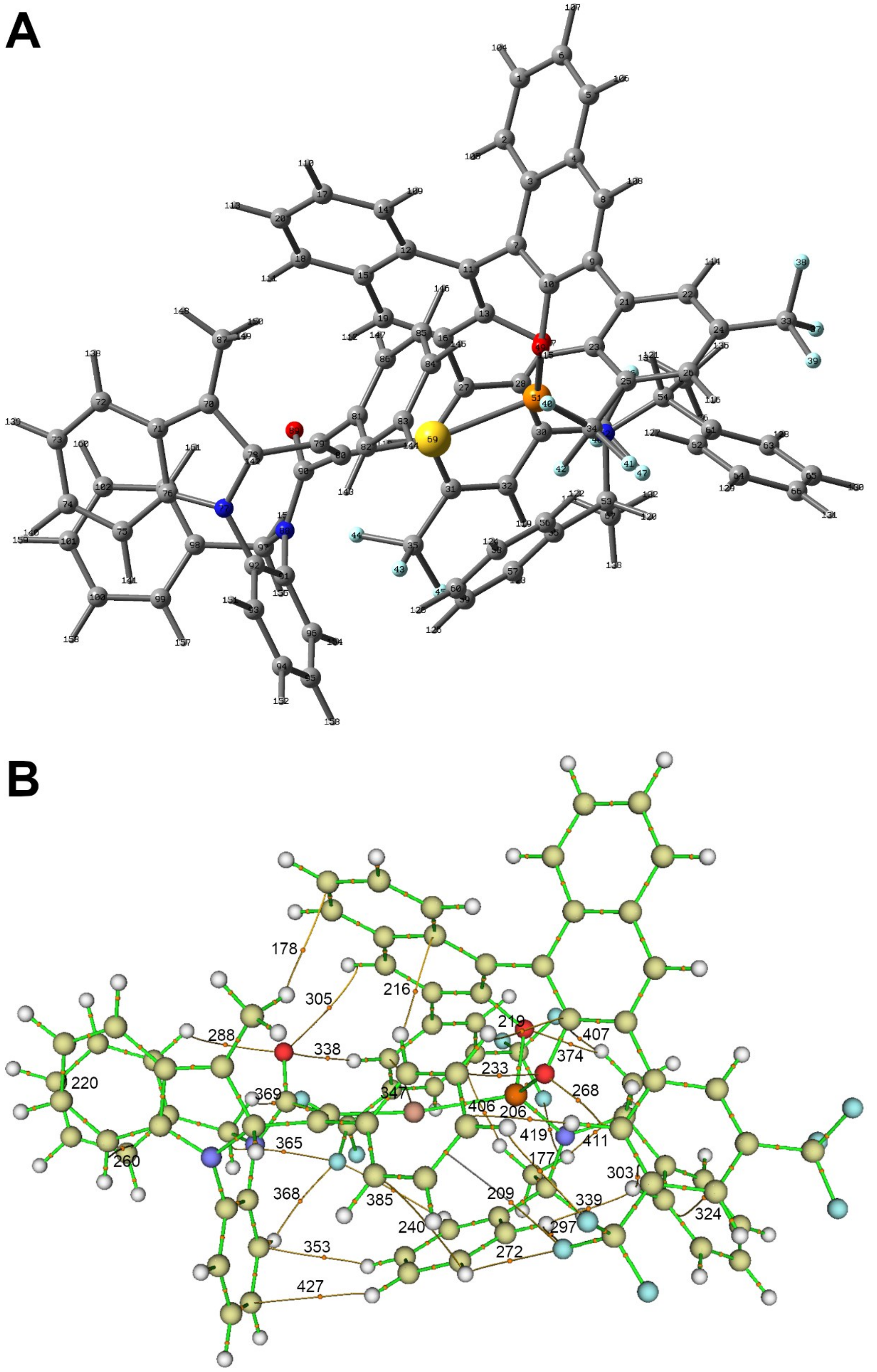

Figure S2: DFT optimized geometry and (3, –1) BCPs for intermediate (*R*)-**II**. (A) Molecular geometry with atom labelling. (B) (3, –1) BCPs of identified NCIs (labelled by BCP number), and corresponding bond paths, obtained by QTAIM analysis.

For Intermediate **III**, the comprehensive analysis confirms that (*S*)-**III** has 29 interactions and (*R*)-**III** has 33 (Table S9, Figures S3, S4). Despite (*R*)-**III** having more individual interactions, (*S*)-**III** is more stabilised by the overall quality of its NCI network. Indeed, the ΣV(r) for (*S*)-**III** is −0.1300 a.u., thus more negative than the −0.1185 for (*R*)-**III** (Table S9). Similarly to intermediate **II**, (*S*)-**III** maintains its unique Au···H contact. Moreover, the hydrogen bonding network is the most significant differentiator. (*S*)-**III** possesses a markedly more stabilising network of C···H bonds (ΣV(r) = −0.0408 a.u. vs. −0.0251 a.u. for (*R*)-**III**) and H···O bonds (ΣV(r) = −0.0341 a.u. vs. −0.0237 a.u.). Although (*R*)-**III** has more than double the number of F···H interactions, their collective strength (ΣV(r) = −0.0193 a.u.) is not sufficient to overcome the stabilisation provided by the more potent C···H and H···O bonds in (*S*)-**III**. Additionally, (*S*)-**III** maintains a significantly more stabilising network of C···C contacts, with double the number of interactions and a much more favourable energy (ΣV(r) = −0.0113 a.u. vs. −0.0065 a.u. for (*R*)-**III**). In contrast, (*R*)-**III** again shows stronger H···H contacts (ΣV(r) = −0.0107 vs. −0.0011). In intermediate **III**, the specific halogen bonding pattern shifts. The F···O bonds are no longer present. Instead, (*R*)-**III** features a unique F···N halogen bond (ΣV(r) = −0.0021 a.u.), where the positive σ-hole of the fluorine atom interacts with the lone pair of a nitrogen. Although this also is a recognised stabilising interaction,[33] its contribution does not compensate for the advantages (*S*)-**III** gains from its C···C, C···H, and H···O interaction networks.

Table S9. NCIs identified by QTAIM analysis on intermediates **III**. Σρ and ΣV(r) are the sum of the total electron density (ρ) and the potential energy density V(r) for each NCI, given in a.u.

	**Intermediate (*S*)-III**			**Intermediate (*R*)-III**		
**Type**	**Count**	**Σρ**	**ΣV(r)**	**Count**	**Σρ**	**ΣV(r)**
Au···C	1	0.0093	−0.0048	1	0.0147	−0.0096
Au···H	1	0.0152	−0.0096	0	0.0000	0.0000
C···C	4	0.0246	−0.0113	2	0.0133	−0.0065
C···F	4	0.0132	−0.0065	1	0.0025	−0.0011
C···H	10	0.0780	−0.0408	10	0.0521	−0.0251
C···O	1	0.0117	−0.0083	3	0.0295	−0.0204
F···H	4	0.0239	−0.0134	9	0.0339	−0.0193
F···N	0	0.0000	0.0000	1	0.0039	−0.0021
H···H	1	0.0025	−0.0011	2	0.0201	−0.0107
O··· H	3	0.0477	−0.0341	3	0.0362	−0.0237
Total	29	0.2260	−0.1300	32	0.2061	−0.1185

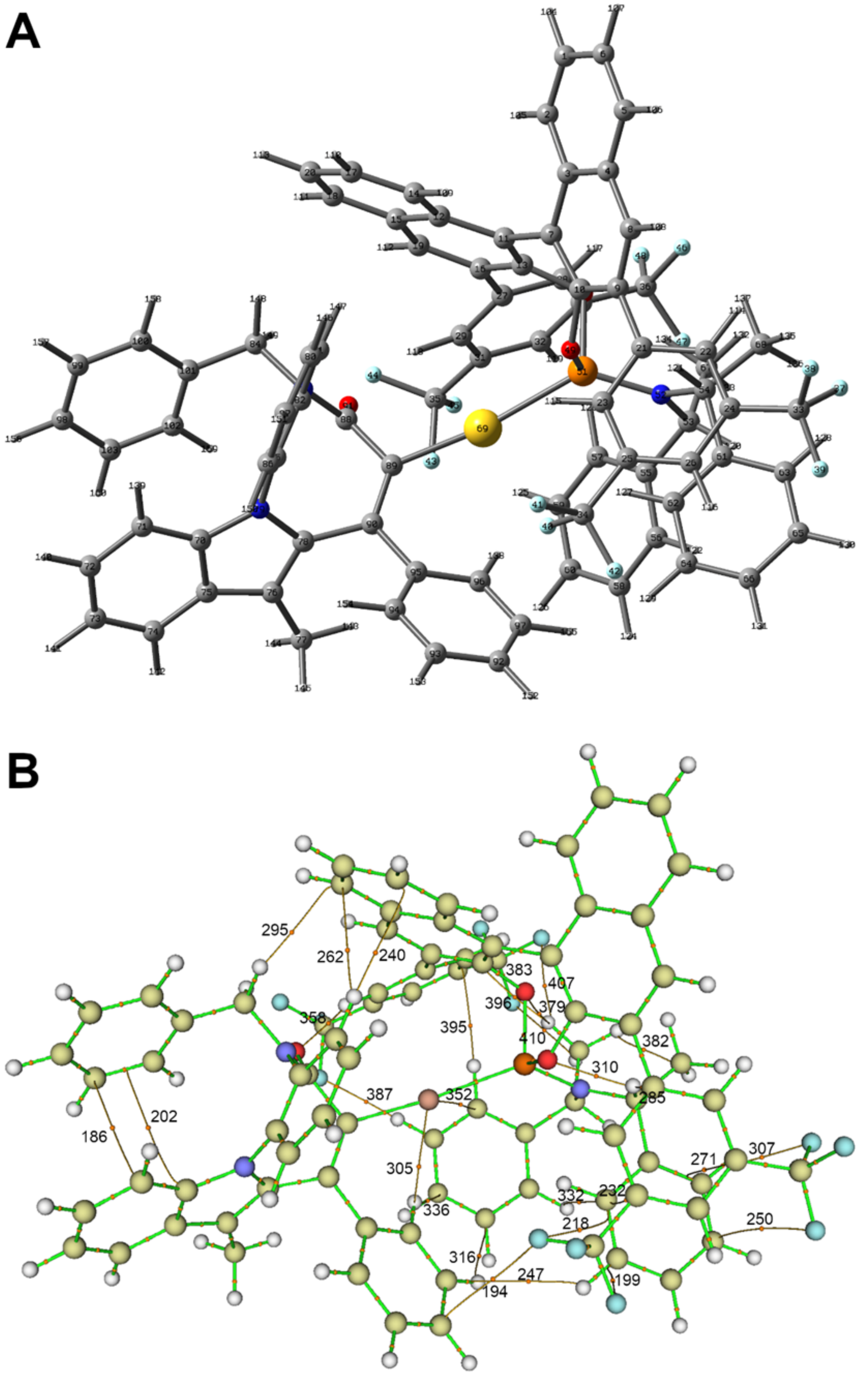

Figure S3: DFT optimized geometry and (3, –1) BCPs for intermediate (*S*)-**III**. (A) Molecular geometry with atom labelling. (B) (3, –1) BCPs of identified NCIs (labelled by BCP number), and corresponding bond paths, obtained by QTAIM analysis.

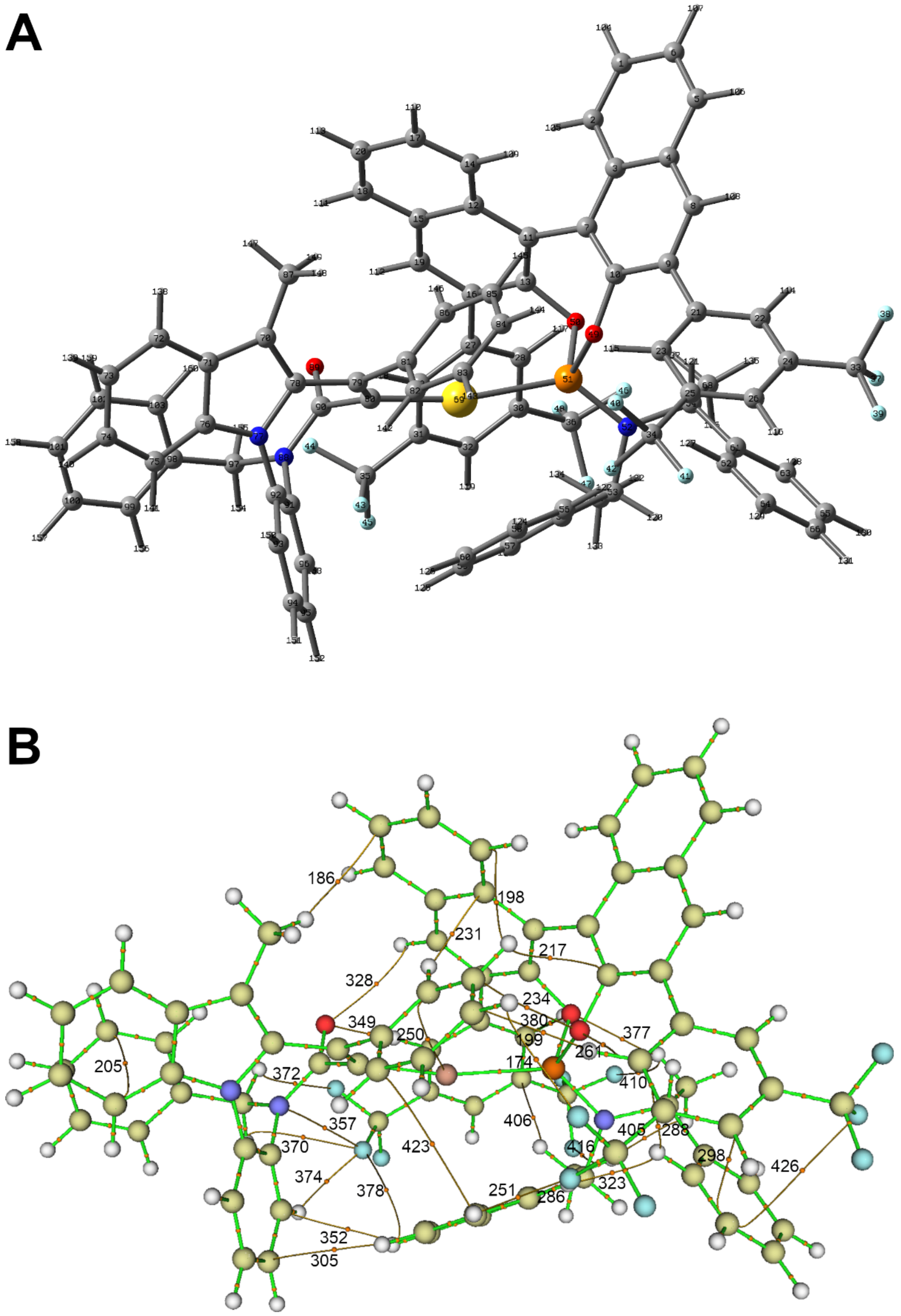

Figure S4: DFT optimized geometry and (3, –1) BCPs for intermediate (*R*)-**III**. (A) Molecular geometry with atom labelling. (B) (3, –1) BCPs of identified NCIs (labelled by BCP number), and corresponding bond paths obtained by QTAIM analysis.

Table S10: Full list of (3, −1) BCPs corresponding to NCIs (H(r) and $\nabla^2\rho > 0$) for intermediate (*S*)-**II**. The following BCP features are reported in a.u.: Density (ρ): electron density at the BCP. V(r): potential energy density. H(r): total energy density. $\nabla^2\rho$: Laplacian of electron density.

Type	Involved Atoms	#BCP	Density (ρ)	V(r)	H(r)	∇²ρ
Au-C	69(Au) -- 57(C)	331	1.16606271E-02	-6.57376016E-03	5.02149488E-04	3.03169001E-02
Au-H	138(H) -- 69(Au)	250	1.27034933E-02	-7.79655736E-03	8.80136923E-04	3.82337850E-02
C-C	71(C) -- 100(C)	210	6.18872929E-03	-2.89233833E-03	9.88665365E-04	1.94786762E-02
	72(C) -- 98(C)	211	7.61905766E-03	-3.69755980E-03	1.05064623E-03	2.31954090E-02
	25(C) -- 62(C)	241	6.88859964E-03	-3.21791498E-03	9.34058301E-04	2.03441263E-02
	24(C) -- 61(C)	290	4.54816758E-03	-1.83031034E-03	8.72378266E-04	1.43002675E-02
C-F	92(C) -- 40(F)	179	4.52843701E-03	-2.31081356E-03	1.15309133E-03	1.84679849E-02
	39(F) -- 65(C)	263	2.40422446E-03	-1.06205336E-03	5.63943521E-04	8.75976161E-03
	37(F) -- 63(C)	314	4.90658673E-03	-2.57215642E-03	1.19366853E-03	1.98379740E-02
C-H	97(C) -- 129(H)	218	4.24412894E-03	-2.08010973E-03	8.72228834E-04	1.52982696E-02
	155(H) -- 58(C)	249	5.19764988E-03	-2.59478447E-03	8.92894712E-04	1.75222956E-02
	138(H) -- 60(C)	272	9.06753036E-03	-4.47128137E-03	1.06782260E-03	2.64277063E-02
	147(H) -- 18(C)	280	5.67256423E-03	-2.65446682E-03	7.89416639E-04	1.69332004E-02
	62(C) -- 122(H)	299	5.18718355E-03	-2.40077087E-03	8.28720141E-04	1.62328446E-02
	148(H) -- 18(C)	303	6.59750669E-03	-3.28019440E-03	8.73763480E-04	2.01108855E-02
	21(C) -- 121(H)	309	1.43339855E-02	-7.98372849E-03	1.45255041E-03	4.35553172E-02
	123(H) -- 28(C)	406	7.15067026E-03	-3.55948739E-03	9.18354740E-04	2.15847875E-02
	68(C) -- 132(H)	410	8.35416936E-03	-4.89051232E-03	1.35169050E-03	3.03755733E-02
	134(H) -- 28(C)	416	7.88439827E-03	-4.00568081E-03	9.35573586E-04	2.35073119E-02
C-O	23(C) -- 49(O)	265	1.48500106E-02	-1.10681961E-02	2.51900421E-03	6.44248180E-02
	50(O) -- 28(C)	407	1.16290933E-02	-8.31731213E-03	2.26915488E-03	5.14224876E-02
F-H	42(F) -- 129(H)	204	4.78905097E-03	-2.53994716E-03	1.25366974E-03	2.01891466E-02
	40(F) -- 127(H)	230	4.41210228E-03	-2.38668719E-03	1.17807873E-03	1.89713786E-02
	159(H) -- 44(F)	334	3.13286744E-03	-1.71332487E-03	8.03169505E-04	1.32786555E-02
	125(H) -- 43(F)	363	7.93478224E-03	-4.46480263E-03	1.96041299E-03	3.35425145E-02
	133(H) -- 47(F)	425	4.58253859E-03	-2.49029326E-03	1.18701139E-03	1.94572642E-02
	134(H) -- 46(F)	426	4.66936711E-03	-2.61893396E-03	1.20205969E-03	2.00922134E-02
F-O	91(O) -- 43(F)	332	4.36611019E-03	-2.49831184E-03	1.38004538E-03	2.10336104E-02
	91(O) -- 44(F)	339	4.96248382E-03	-2.93560981E-03	1.57827920E-03	2.43686729E-02

H-H	143(H) -- 126(H)	256	6.90670532E-04	-2.09280043E-04	2.12146150E-04	2.53428937E-03
	149(H) -- 118(H)	338	6.92268720E-03	-3.47313524E-03	8.52510361E-04	2.07126239E-02
	149(H) -- 112(H)	345	5.15150788E-03	-2.57276667E-03	9.42619180E-04	1.78320201E-02
O-H	91(O) -- 159(H)	291	8.24702383E-03	-4.60626167E-03	1.52093824E-03	3.05925526E-02
	49(O) -- 121(H)	323	1.69127709E-02	-1.22246094E-02	2.41185545E-03	6.81932810E-02
	91(O) -- 118(H)	336	1.21989014E-02	-7.38274492E-03	2.18712857E-03	4.70280082E-02
	50(O) -- 134(H)	402	6.73216102E-03	-3.81197228E-03	1.38909716E-03	2.63606665E-02

Table S11: Full list of (3, –1) BCPs corresponding to NCIs (H(r) and $\nabla^2\rho > 0$) for intermediate (*R*)-**II**. The following BCP features are reported in a.u.: Density (ρ): electron density at the BCP. V(r): potential energy density. H(r): total energy density. $\nabla^2\rho$: Laplacian of electron density.

Type	Involved Atoms	#BCP	Density (ρ)	V(r)	H(r)	∇²ρ
Au-C	69(Au) -- 29(C)	347	7.90899921E-03	-4.33195919E-03	8.60064811E-04	2.42101212E-02
C-C	74(C) -- 101(C)	220	7.80205364E-03	-3.80336021E-03	1.05042771E-03	2.36168625E-02
	75(C) -- 99(C)	260	6.24865051E-03	-2.94837442E-03	1.02679384E-03	2.00078484E-02
	26(C) -- 62(C)	324	6.68157571E-03	-3.32489697E-03	1.03373389E-03	2.15694590E-02
C-F	97(C) -- 43(F)	365	6.44462370E-03	-4.15259697E-03	1.77100276E-03	3.07784100E-02
C-H	150(H) -- 20(C)	178	5.23032758E-03	-2.29700707E-03	6.90436455E-04	1.47115199E-02
	84(C) -- 115(H)	206	7.21463589E-03	-3.40711100E-03	9.82154357E-04	2.14856788E-02
	147(H) -- 12(C)	216	6.00622923E-03	-2.84484784E-03	8.65964052E-04	1.83071038E-02
	146(H) -- 10(C)	219	3.42715701E-03	-1.60797392E-03	7.06901187E-04	1.20871052E-02
	82(C) -- 124(H)	240	2.30050858E-03	-8.01300155E-04	5.28678330E-04	7.43462726E-03
	25(C) -- 127(H)	303	8.94508452E-03	-4.69191132E-03	1.12517581E-03	2.77690518E-02
	96(C) -- 125(H)	353	7.60402298E-03	-3.89187054E-03	1.03074391E-03	2.38134335E-02
	134(H) -- 30(C)	406	5.90016610E-03	-2.83849825E-03	8.70880504E-04	1.83210370E-02
	126(H) -- 95(C)	427	3.18457858E-03	-1.36098771E-03	6.74561980E-04	1.08404467E-02
C-O	85(C) -- 49(O)	233	4.01058061E-03	-1.84136814E-03	9.20253269E-04	1.47274987E-02
	23(C) -- 49(O)	268	1.22064612E-02	-8.92943384E-03	2.39330629E-03	5.48641857E-02
F-H	145(H) -- 40(F)	177	6.77970868E-03	-4.01406629E-03	1.73102669E-03	2.99044787E-02
	42(F) -- 124(H)	272	5.56572810E-03	-3.56764877E-03	1.68506930E-03	2.77511495E-02
	42(F) -- 122(H)	297	6.17946292E-03	-3.77488081E-03	1.64661366E-03	2.82724325E-02
	154(H) -- 43(F)	368	2.94273730E-03	-1.62534711E-03	8.30448985E-04	1.31449803E-02
	156(H) -- 44(F)	369	8.22252556E-03	-4.99042197E-03	1.99655445E-03	3.59341235E-02

	43(F) -- 123(H)	385	2.04704712E-03	-1.00121765E-03	5.74704944E-04	8.60251014E-03
	137(H) -- 46(F)	407	1.46540504E-03	-6.71867867E-04	4.06613886E-04	5.94038256E-03
	132(H) -- 47(F)	419	4.85039732E-03	-2.74779644E-03	1.26142295E-03	2.10825694E-02
H-H	122(H) -- 127(H)	339	8.10642728E-03	-4.11790786E-03	1.10936082E-03	2.53465180E-02
	136(H) -- 132(H)	411	1.10872314E-02	-6.01686274E-03	1.44084555E-03	3.55942154E-02
O-H	161(H) -- 89(O)	288	9.12545346E-03	-5.21182541E-03	1.63972557E-03	3.39651063E-02
	89(O) -- 112(H)	305	6.61083194E-03	-3.63965623E-03	1.41048424E-03	2.58424989E-02
	89(O) -- 118(H)	338	1.05985609E-02	-5.76082425E-03	2.10218958E-03	3.98608137E-02
	50(O) -- 137(H)	374	1.00542567E-02	-6.31284656E-03	1.96511782E-03	4.09723288E-02

Table S12: List of (3, –1) BCPs corresponding to NCIs (H(r) and $\nabla^2\rho$ at BCP > 0) for intermediate (*S*)-**III**. The following BCP features are reported in a.u.: Density (ρ): electron density at the BCP. V(r): potential energy density. H(r): total energy density. $\nabla^2\rho$: Laplacian of electron density.

Type	Involved Atoms	#BCP	Density (ρ)	V(r)	H(r)	$\nabla^2\rho$
Au-C	69(Au) -- 57(C)	352	9.33129668E-03	-4.81398267E-03	6.46106036E-04	2.44272410E-02
Au-H	138(H) -- 69(Au)	305	1.51598948E-02	-9.64750464E-03	7.12721519E-04	4.43021238E-02
C-C	71(C) -- 103(C)	186	6.26819116E-03	-2.95820811E-03	9.31503884E-04	1.92848635E-02
	70(C) -- 102(C)	202	6.27148355E-03	-2.94191686E-03	9.56474785E-04	1.94194657E-02
	25(C) -- 62(C)	232	6.91484387E-03	-3.21040151E-03	9.36365596E-04	2.03325308E-02
	24(C) -- 63(C)	271	5.14610922E-03	-2.15399986E-03	8.97855337E-04	1.57988421E-02
C-F	40(F) -- 92(C)	194	1.99982431E-03	-8.62270105E-04	5.01612729E-04	7.46198225E-03
	42(F) -- 64(C)	199	4.31654124E-03	-2.22156665E-03	1.07596517E-03	1.74939879E-02
	40(F) -- 62(C)	218	4.38982803E-03	-2.34337097E-03	1.15557076E-03	1.86180500E-02
	39(F) -- 65(C)	250	2.53839262E-03	-1.11998207E-03	5.91955871E-04	9.21557524E-03
C-H	147(H) -- 17(C)	240	5.79180771E-03	-3.10464119E-03	1.00771005E-03	2.04802451E-02
	147(H) -- 18(C)	262	6.26161069E-03	-3.27021752E-03	9.64406656E-04	2.07961233E-02
	21(C) -- 121(H)	285	1.50360696E-02	-8.41237312E-03	1.46810333E-03	4.53943192E-02
	148(H) -- 18(C)	295	8.17216998E-03	-4.16674553E-03	9.63228279E-04	2.43728083E-02
	155(H) -- 58(C)	316	6.20257066E-03	-3.11159149E-03	9.24953839E-04	1.98459967E-02
	62(C) -- 122(H)	332	5.83288972E-03	-2.77407569E-03	8.74901062E-04	1.80955113E-02
	138(H) -- 60(C)	336	7.75660279E-03	-3.77757667E-03	9.53324590E-04	2.27369034E-02
	68(C) -- 132(H)	382	7.89250781E-03	-4.62509414E-03	1.32761129E-03	2.91212669E-02
	123(H) -- 30(C)	395	7.36587480E-03	-3.71914154E-03	9.49270963E-04	2.24707339E-02

	134(H) -- 28(C)	396	7.69757134E-03	-3.88413821E-03	9.02524474E-04	2.27567486E-02
C-O	50(O) -- 28(C)	383	1.16729729E-02	-8.33831835E-03	2.26920864E-03	5.15069425E-02
F-H	37(F) -- 128(H)	307	5.53765329E-03	-3.05650279E-03	1.40913599E-03	2.34990991E-02
	125(H) -- 43(F)	387	9.46292464E-03	-5.50208301E-03	2.39201323E-03	4.11444379E-02
	134(H) -- 46(F)	407	4.42862251E-03	-2.45968536E-03	1.13722594E-03	1.89365489E-02
	133(H) -- 47(F)	410	4.42584766E-03	-2.38722010E-03	1.14199025E-03	1.86848024E-02
H-H	129(H) -- 155(H)	247	2.51346893E-03	-1.07671236E-03	6.28411183E-04	9.33413891E-03
O-H	49(O) -- 121(H)	310	1.81286252E-02	-1.32991841E-02	2.38679008E-03	7.22910572E-02
	91(O) -- 118(H)	358	2.27562087E-02	-1.69559429E-02	2.55838497E-03	8.82908514E-02
	50(O) -- 134(H)	379	6.77177040E-03	-3.82928051E-03	1.38918041E-03	2.64305654E-02

Table S13: List of (3, –1) BCPs corresponding to NCIs (H(r) and $\nabla^2\rho$ at BCP > 0) for intermediate (*R*)-**III**. The following BCP features are reported in a.u.: Density (ρ): electron density at the BCP. V(r): potential energy density. H(r): total energy density. $\nabla^2\rho$: Laplacian of electron density.

Type	Involved Atoms	#BCP	Density (ρ)	V(r)	H(r)	$\nabla^2\rho$
Au-C	86(C) -- 69(Au)	250	1.46994179E-02	-9.62708881E-03	6.74456809E-04	4.39116754E-02
C-C	75(C) -- 102(C)	205	6.63548949E-03	-3.17418705E-03	9.46150734E-04	2.02659541E-02
	26(C) -- 64(C)	298	6.62495502E-03	-3.28034791E-03	1.02001020E-03	2.12814733E-02
C-F	37(F) -- 64(C)	426	2.45170677E-03	-1.08439340E-03	6.29300610E-04	9.37197850E-03
C-H	149(H) -- 20(C)	186	2.28746866E-03	-8.58573713E-04	4.14786679E-04	6.75258828E-03
	145(H) -- 14(C)	198	3.09650458E-03	-1.39967915E-03	6.85470295E-04	1.10824789E-02
	84(C) -- 115(H)	199	6.06536719E-03	-2.76303166E-03	8.55194765E-04	1.78936848E-02
	145(H) -- 10(C)	217	3.75325096E-03	-1.75547607E-03	7.16723086E-04	1.27556890E-02
	146(H) -- 12(C)	231	7.14476398E-03	-3.53866569E-03	9.83654980E-04	2.20239026E-02
	25(C) -- 127(H)	288	9.06990037E-03	-4.75396334E-03	1.12906933E-03	2.80484079E-02
	126(H) -- 95(C)	305	4.81582422E-03	-2.24367426E-03	8.07453218E-04	1.54343228E-02
	96(C) -- 125(H)	352	7.33284671E-03	-3.77934624E-03	1.03506030E-03	2.33978674E-02
	134(H) -- 30(C)	406	7.37145100E-03	-3.62891382E-03	9.62824479E-04	2.22182511E-02
	82(C) -- 124(H)	423	1.19662565E-03	-3.39151869E-04	2.84750346E-04	3.63461025E-03
C-O	85(C) -- 49(O)	234	3.80779834E-03	-1.73479429E-03	8.89917805E-04	1.40585196E-02
	23(C) -- 49(O)	261	1.23545098E-02	-9.00450991E-03	2.40009534E-03	5.52188024E-02
	50(O) -- 28(C)	380	1.33300402E-02	-9.69523565E-03	2.37583235E-03	5.77876014E-02
F-H	144(H) -- 40(F)	174	4.78890993E-03	-2.65221478E-03	1.29782407E-03	2.09914517E-02
	42(F) -- 124(H)	251	5.27149436E-03	-3.32117472E-03	1.58550382E-03	2.59687294E-02

	42(F) -- 122(H)	286	5.44911411E-03	-3.27867161E-03	1.46858826E-03	2.48633925E-02
	154(H) -- 43(F)	370	3.82229219E-03	-2.25885295E-03	1.08634204E-03	1.77261481E-02
	155(H) -- 44(F)	372	3.87460241E-03	-2.11105853E-03	1.01146973E-03	1.65359919E-02
	153(H) -- 43(F)	374	3.54397041E-03	-2.01186555E-03	9.84371707E-04	1.59224358E-02
	125(H) -- 43(F)	378	1.11623278E-03	-4.97314449E-04	3.55075727E-04	4.82986361E-03
	137(H) -- 46(F)	410	1.92269875E-03	-9.25335486E-04	5.60588308E-04	8.18604841E-03
	132(H) -- 47(F)	416	4.07078363E-03	-2.27619865E-03	1.13726051E-03	1.82028787E-02
F-N	88(N) -- 43(F)	357	3.90541167E-03	-2.14050934E-03	1.24234412E-03	1.85007903E-02
H-H	127(H) -- 122(H)	323	9.22202844E-03	-4.71615871E-03	1.25068423E-03	2.88701087E-02
	136(H) -- 132(H)	405	1.09072233E-02	-5.95376116E-03	1.43229918E-03	3.52734381E-02
O-H	89(O) -- 112(H)	328	5.82201404E-03	-3.10171765E-03	1.26020456E-03	2.24885070E-02
	89(O) -- 118(H)	349	2.12474336E-02	-1.50285967E-02	2.56127008E-03	8.06045474E-02
	50(O) -- 137(H)	377	9.12564140E-03	-5.60402593E-03	1.82232126E-03	3.69946738E-02

Cartesian Coordinates

Gaussian root section for opt freq of minima:
opt freq M06/LANL2DZ Int=Ultrafine 10f 6d gfinput gfprint

Gaussian root section for opt freq of TSs:
opt=(calcfc,ts,noeigentest) freq M06/LANL2DZ Int=Ultrafine 10f 6d gfinput gfprint

Gaussian root section for SP calculations:
#P PBE1PBE/Def2TZVP EmpiricalDispersion=GD3BJ Geom=Checkpoint Guess=Read SCRF=(CPCM,Solvent=chlorobenzene) Int=Ultrafine

To output the wavefunction for QTAIM analyses the "output=wfn" keyword was added to the root section, and the wavefunction output file name was added following the coordinate section, leaving a blank line between the coordinates and the .wfn file name. Multiwfn software can be downloaded for free at http://sobereva.com/multiwfn/download.html

1aa

0 1

C	-1.03979300	2.30061900	0.09063500
C	0.16093500	2.47313400	-0.61134100
C	0.25123800	3.57197700	-1.46849500
C	-0.82289000	4.48245000	-1.61859000
C	-2.00689800	4.31638200	-0.89968200
C	-2.12109800	3.21606500	-0.02877000
C	-3.17107700	2.78189400	0.86550100
C	-4.49089400	3.45786200	1.05699500
C	-2.70668300	1.64837100	1.49053300
N	-1.41673300	1.32838500	1.02412900
C	0.67577100	-2.09103600	2.37787400
C	0.47562800	-1.90063100	1.00811100
C	-0.19872400	-0.76634100	0.52598600
N	-0.46490300	-0.64940800	-0.87301000
C	-1.87669000	-0.70964500	-1.31856600
C	-0.68519600	0.18808700	1.44587400
C	-0.49655500	-0.01794700	2.82290900
C	0.17922000	-1.14790900	3.29108900
C	0.53718900	-0.67468300	-1.83641900
C	1.88576900	-0.53808000	-1.32612000
C	3.03145300	-0.40359800	-0.92126800
O	0.29386000	-0.77910100	-3.06038800
C	6.87342800	0.06041300	0.80624000
C	5.72652500	0.07897700	1.61517900
C	4.46162800	-0.07420800	1.04067400
C	4.33391800	-0.24766600	-0.35508200
C	5.48958800	-0.26473800	-1.16284900
C	6.75184600	-0.11153500	-0.58140400
C	-3.55218900	-4.54472700	-0.23963400
C	-2.58809800	-4.45376400	-1.25704700
C	-2.05929600	-3.20767500	-1.61267600
C	-2.49051200	-2.04094900	-0.95733200
C	-3.45321200	-2.13725100	0.05780700
C	-3.98507100	-3.38459100	0.41673300
H	0.99950500	1.79303800	-0.48628600
H	1.16560000	3.72981200	-2.03265100
H	-0.71392500	5.32208400	-2.29875200
H	-2.82627400	5.02393900	-1.00312900
H	-5.11542700	2.92089100	1.77834800
H	-5.04566400	3.51997000	0.11213700
H	-4.36051700	4.48405900	1.42412700
H	-3.19033400	1.01358400	2.22164700
H	1.19240900	-2.97794000	2.73028200
H	0.81043600	-2.64572100	0.29088400
H	-2.43486300	0.11697300	-0.86109700
H	-1.85700500	-0.55900800	-2.40284900
H	-0.86948500	0.73500600	3.51241400
H	0.31994500	-1.29140600	4.35780400
H	7.85536300	0.18019300	1.25393900
H	5.82108400	0.21388500	2.68816000
H	3.56319300	-0.06111300	1.65362900
H	5.38419600	-0.39738600	-2.23544700
H	7.63976800	-0.12481000	-1.20571400
H	-3.96322700	-5.51176100	0.03566400
H	-2.25615700	-5.35139300	-1.77085500
H	-1.31577800	-3.12520800	-2.40414100
H	-3.78632500	-1.23115800	0.56297300
H	-4.73151700	-3.45209400	1.20300400

(*S*)-2aa

0 1

C	-1.00549300	1.91434800	-0.59302500
N	0.70637900	-2.10506600	-0.87844500
O	-0.25716000	-2.06544000	-2.97447700
C	0.14855000	2.50376000	0.03743200
C	0.68152600	3.80726500	0.03230100
H	0.19711800	4.59860100	-0.53480000
C	1.83753800	4.06286400	0.77170000
H	2.26202600	5.06270900	0.78556500
C	2.47281600	3.03245000	1.50782800
H	3.37834000	3.25703000	2.06468700
C	1.96714700	1.73081200	1.52175200
H	2.47416900	0.93615200	2.06279700
C	0.80242500	1.48591600	0.78220800
N	0.08890400	0.30125100	0.59469600
C	-1.01950800	0.57619600	-0.24209000
C	-2.02728600	-0.44946900	-0.56284600
C	-1.70074900	-1.63062100	-1.14138900
H	-2.47247900	-2.36667300	-1.35577300
C	-0.36808900	-1.94192500	-1.73172300
C	0.60648000	-2.10024800	0.54968700
C	0.34972500	-0.91903500	1.26886600
C	0.34474400	-0.94436700	2.67292100
H	0.12598400	-0.02416400	3.20817800
C	0.61314500	-2.13079900	3.36150100
H	0.61144600	-2.13901900	4.44686900
C	0.86619100	-3.31160300	2.64624000
H	1.06037500	-4.23964700	3.17453600
C	0.85657900	-3.29151800	1.24805600
H	1.04037800	-4.19940200	0.67888200
C	-3.44090700	-0.13519700	-0.23041600
C	-3.74689500	0.63274300	0.91035900
H	-2.93597500	1.00992900	1.53034600
C	-5.07637700	0.90259700	1.25282700
H	-5.29681300	1.48815000	2.14052500
C	-6.12302900	0.42005600	0.45306000
H	-7.15478300	0.63495400	0.71506300
C	-5.82837500	-0.32998300	-0.69505600
H	-6.63269900	-0.68967900	-1.33002300
C	-4.49878800	-0.60319100	-1.03514100
H	-4.27477800	-1.15333500	-1.94614800
C	2.05527500	-2.30853300	-1.45101900
H	1.90464500	-2.47744000	-2.52317900
H	2.49936700	-3.21228400	-1.01410600
C	2.94226200	-1.11080400	-1.20685800
C	2.65087000	0.11029000	-1.84049900
H	1.78231900	0.17683300	-2.49487900
C	3.46695100	1.22657100	-1.63161900
H	3.22883600	2.17068800	-2.11318600
C	4.58656900	1.13186700	-0.78898500
H	5.21807700	2.00100600	-0.62763000
C	4.87838900	-0.07980800	-0.14922800
H	5.74151500	-0.15540600	0.50607600
C	4.05341900	-1.19679600	-0.35571700
H	4.27431800	-2.14032600	0.14211700
C	-1.98219200	2.63368700	-1.46724000
H	-1.46532500	3.17170400	-2.27211800
H	-2.69375000	1.94219400	-1.92994800
H	-2.55949600	3.37249700	-0.89509800

AuL·SbF_6

0 1

C	-1.35842600	4.67946500	5.04506000
C	-0.78781900	3.84881300	4.09803600
C	-1.56158500	3.32343800	3.02276500
C	-2.95319900	3.65327200	2.97070700
C	-3.51299200	4.52328300	3.94864000
C	-2.73230800	5.03246400	4.96750100
C	-1.00090900	2.45125100	2.02253900
C	-3.76265500	3.10289500	1.94419400
C	-3.25366600	2.21124900	1.01692800
C	-1.86545700	1.87304200	1.09870300
C	0.46021000	2.16495100	2.02078000
C	1.44331000	3.20059400	1.85103600
C	0.90816900	0.85816400	2.12999300
C	1.09596100	4.54599800	1.54518700
C	2.83198100	2.84957300	1.89328100
C	2.28379800	0.48653200	2.18011300
C	2.07645800	5.48982900	1.29790600
C	3.81966200	3.84400100	1.65269900
C	3.22103900	1.50005100	2.09405400
C	3.45211000	5.14087000	1.35377700
C	-4.12853900	1.65696700	-0.04043900
C	-5.43024800	1.24402200	0.27678900
C	-3.69925700	1.55737800	-1.37988000
C	-6.28699700	0.75518800	-0.71781300
C	-4.56770400	1.07176600	-2.35664200
C	-5.87588000	0.67611900	-2.04495700
C	2.67641300	-0.94030100	2.19460300
C	3.67490600	-1.39189600	1.31325300
C	2.03694200	-1.87420200	3.02890000

C	4.00231900	-2.75327600	1.26191800
C	2.38040100	-3.22701400	2.96407900
C	3.36585000	-3.68580300	2.08241200
C	-7.64607700	0.29509200	-0.30653400
C	-4.10630300	0.88373300	-3.76257100
C	1.67887900	-4.19799300	3.85418800
C	4.99339200	-3.20117400	0.23725600
F	-8.44519500	1.34863000	0.13207400
F	-8.33621800	-0.33046000	-1.32628400
F	-7.59317700	-0.59924400	0.75693200
F	-2.86106200	1.41536600	-4.01343500
F	-4.98157100	1.42199400	-4.69157200
F	-4.02091500	-0.48287500	-4.08016300
F	0.35159400	-3.84436400	4.07953900
F	1.65939000	-5.48064600	3.32781600
F	2.26190300	-4.30034500	5.11119300
F	6.11643400	-2.40014300	0.19078400
F	5.42297200	-4.50082500	0.43920000
F	4.44035100	-3.16926800	-1.04818800
O	-1.42484600	0.95513500	0.13305100
O	-0.04982300	-0.18452900	2.05138800
P	-0.47911000	-0.46858800	0.37977000
N	-1.52668700	-1.79128700	0.40388900
C	-2.72875700	-1.76762100	1.31158300
C	-1.11395600	-3.07199100	-0.28250200
C	-3.90695600	-2.45075100	0.63149100
C	-4.33473800	-1.97277500	-0.61946700
C	-4.59098700	-3.53245300	1.20821900
C	-5.40244500	-2.57124400	-1.29340100
C	-5.67519500	-4.12576500	0.54293000
C	-6.08020200	-3.65323200	-0.71101700
C	-0.51948500	-2.77097600	-1.65712500
C	-1.31244800	-2.11472900	-2.61674100
C	0.81884600	-3.06635300	-1.97851900
C	-0.77707000	-1.72556400	-3.85024500
C	1.36287000	-2.67275600	-3.21442100
C	0.56968100	-1.98943100	-4.14707100
C	-2.40609700	-2.29438300	2.71209800
C	-0.19709000	-3.90164600	0.61653600
Au	1.40964400	-0.31507700	-1.03389200
H	-0.75243500	5.06425300	5.85923500
H	0.26130100	3.58267100	4.17782000
H	-4.56976000	4.77108000	3.88232200
H	-3.16262800	5.69198000	5.71416300
H	-4.80595100	3.40868300	1.88250600
H	0.04863700	4.82390000	1.47275700
H	1.79370900	6.50564100	1.03972800
H	4.86669100	3.55173100	1.66223800
H	4.28207700	1.26189900	2.11532200
H	4.20791200	5.88742500	1.13471200
H	-5.76882400	1.26952300	1.31077300
H	-2.69030200	1.85407800	-1.64624900
H	-6.54293800	0.29685300	-2.81473300
H	4.18008200	-0.67967800	0.65772400
H	1.25567200	-1.54252000	3.70678900
H	3.61692900	-4.74051300	2.02496000
H	-2.98603700	-0.69797700	1.40817500
H	-2.06124900	-3.60995400	-0.42887000
H	-3.81457300	-1.12336200	-1.06356200
H	-4.29275900	-3.91700000	2.17910200
H	-5.70442600	-2.20155800	-2.27047300
H	-6.19949100	-4.95690600	1.00458200
H	-6.91488300	-4.11685600	-1.22721200
H	-2.35558600	-1.90065100	-2.39420800
H	1.45704300	-3.59348900	-1.27394600
H	-1.40623400	-1.21349500	-4.57202700
H	2.40389000	-2.88823900	-3.43354100
H	0.99537300	-1.67104600	-5.09277300
H	-3.24711000	-2.09923300	3.38735400
H	-2.21407300	-3.37264500	2.71495000
H	-1.51929200	-1.78853100	3.10690000
H	-0.67755100	-4.11845200	1.57507200
H	0.05904200	-4.85554400	0.14195800
H	0.73466800	-3.35570600	0.82678700
F	3.20806700	0.08398700	-2.25752300
Sb	4.13246900	1.84747200	-2.01112800
F	4.78161900	1.06504800	-0.37333800
F	5.62839900	1.15157200	-2.95287200
F	3.30527800	2.43389600	-3.62368700
F	2.51468400	2.30635600	-1.04826000
F	4.94851600	3.51982800	-1.62082600

Intermediate (*S*)-II

1 1

C	3.23695500	4.81971000	5.16340400
C	2.48943400	4.08207000	4.26324100
C	3.02358600	2.90761400	3.65914800
C	4.36339600	2.52541300	3.98979900
C	5.10525300	3.29324900	4.93087300
C	4.55498100	4.41943300	5.51102600
C	2.28281700	2.12377100	2.70601000
C	4.93247200	1.37495900	3.38317200
C	4.25432100	0.64983900	2.42055600
C	2.94122700	1.09018100	2.05117300
C	0.84393600	2.41119400	2.44684500
C	-0.13406100	2.31172500	3.49582300
C	0.41123600	2.73729500	1.16867900
C	0.18308900	1.84398300	4.80501200
C	-1.49442700	2.66097500	3.21089400
C	-0.94052600	3.08185200	0.86196300
C	-0.78863400	1.76425400	5.78659000
C	-2.46943500	2.59763000	4.24869800
C	-1.85970100	3.06050600	1.89809400
C	-2.12695800	2.16096400	5.51453900
C	4.84974400	-0.56665700	1.82086300
C	6.19506300	-0.59216900	1.42505400
C	4.07203700	-1.71864400	1.61370400
C	6.73485600	-1.73254500	0.81568700
C	4.62341800	-2.84343300	0.99933600
C	5.96146300	-2.87342100	0.59665700
C	-1.35910400	3.46040100	-0.50667300
C	-0.58891700	4.33901300	-1.29104200
C	-2.57586500	2.98655500	-1.02263900
C	-1.04038200	4.72418900	-2.55447000
C	-3.02833600	3.40582100	-2.27901600
C	-2.26584700	4.27335500	-3.06240600
C	8.17214200	-1.68934900	0.41112900
C	3.71967200	-4.01101100	0.78291900
C	-4.34124000	2.90386100	-2.79050000
C	-0.23168500	5.63860200	-3.41646800
F	8.46719700	-0.56004300	-0.35192600
F	9.01795900	-1.61800400	1.51400700
F	8.56504700	-2.78639000	-0.32471100
F	2.47583800	-3.61094700	0.29706800
F	3.45005400	-4.70221800	1.96107700
F	4.22147700	-4.93960100	-0.10898600
F	-4.23015800	1.70515000	-3.49199000
F	-5.25552500	2.68315600	-1.77524600
F	-4.92986900	3.79869800	-3.67084400
F	0.94167300	6.05306900	-2.81834500
F	0.12468200	5.02307100	-4.61695900
F	-0.93246800	6.77984300	-3.77353300
O	2.30405600	0.35668300	1.03621300
O	1.34236700	2.66370400	0.11928000
P	1.58776200	0.98483800	-0.40602100
N	2.84180200	1.07764400	-1.54336000
C	2.55411900	1.48652700	-2.96170300
C	4.26313600	0.82564100	-1.18488100
C	1.37441300	0.74028800	-3.57043500
C	1.61269200	-0.40593200	-4.34881700
C	0.04955300	1.19104700	-3.41828800
C	0.55508200	-1.07571200	-4.97794900
C	-1.01648700	0.50353300	-4.01725500
C	-0.76319600	-0.62549900	-4.80903400
C	4.76539800	-0.41596300	-1.91513400
C	3.95145000	-1.56503900	-1.95635700
C	6.04904700	-0.47765900	-2.48102500
C	4.41106200	-2.74575400	-2.54856800
C	6.51445600	-1.66326000	-3.07110000
C	5.69826500	-2.80088400	-3.10720600
C	2.40765800	3.00718800	-3.07389100
C	5.13481900	2.07280500	-1.31900200
Au	-0.46725700	-0.31050000	-0.56627300
C	-5.52744000	-3.16237800	0.60236400

C	-6.58035200	-3.01238700	1.52839600
C	-7.86409600	-2.95513400	1.01418500
C	-8.14454000	-3.01674000	-0.39624900
C	-7.12873700	-3.16051200	-1.31012000
C	-5.78602900	-3.25724300	-0.82151100
C	-4.57517900	-3.43724900	-1.46861400
C	-4.26670700	-3.49701100	-2.91298000
C	-3.49866400	-3.57796400	-0.42969200
N	-4.19898300	-3.28294800	0.83668600
C	-2.10365800	-2.20596700	4.33865400
C	-2.50223200	-1.21870700	3.43000600
C	-3.20886000	-1.56920600	2.27088700
N	-3.63473200	-0.57536500	1.34341900
C	-4.45007500	0.56185500	1.82868800
C	-3.50798000	-2.93158600	2.04215100
C	-3.09296300	-3.91984000	2.93899100
C	-2.39404500	-3.55720200	4.09750600
C	-3.31885600	-0.64075600	-0.01082300
C	-2.15872400	-1.47625000	-0.41648200
C	-2.20782800	-2.80403500	-0.66723600
O	-3.90257300	0.09887000	-0.84773000
C	1.05216100	-5.15192300	-2.23665200
C	0.26915000	-5.64986100	-1.18506200
C	-0.78650900	-4.88700500	-0.67380500
C	-1.07813900	-3.61024900	-1.19739800
C	-0.28175900	-3.12010400	-2.25071200
C	0.77622200	-3.88183400	-2.76097200
C	-8.71529400	-0.01978500	2.18287100
C	-7.89249600	-0.17714000	3.30891200
C	-6.50461800	-0.02029100	3.18475200
C	-5.93127600	0.28788100	1.93906300
C	-6.75870800	0.43258400	0.81105900
C	-8.14517700	0.27951900	0.93434600
H	2.81789800	5.71828100	5.60483100
H	1.49010200	4.41206000	3.99582100
H	6.11736600	2.98180300	5.17718800
H	5.12593600	5.00638500	6.22275800
H	5.92085300	1.05040300	3.70495700
H	1.20227700	1.54423500	5.02875400
H	-0.52672300	1.40920300	6.77827500
H	-3.48682800	2.91679900	4.02926400
H	-2.88770400	3.37022200	1.71342300
H	-2.87140400	2.12328800	6.30321900
H	6.81587200	0.29330200	1.55347800
H	3.02598200	-1.72032900	1.90407900
H	6.38056000	-3.74584800	0.10509000
H	0.35197700	4.72174300	-0.90673700
H	-3.17356700	2.27017300	-0.46315800
H	-2.61655000	4.60235100	-4.03762000
H	3.45328300	1.17256500	-3.51122800
H	4.25311300	0.56599300	-0.12287200
H	2.63587000	-0.75740400	-4.47844200
H	-0.16067500	2.10143100	-2.85600600
H	0.75903000	-1.93538900	-5.61010500
H	-2.03518600	0.85461700	-3.87321000
H	-1.58327400	-1.14091000	-5.30059500
H	2.94882900	-1.54020600	-1.52520900
H	6.70181500	0.38997700	-2.46178800
H	3.76932000	-3.62362500	-2.56221700
H	7.51394700	-1.69550300	-3.49381200
H	6.05896700	-3.71793100	-3.56259800
H	3.31122900	3.51558500	-2.72699600
H	2.21542500	3.29677600	-4.11268600
H	1.57809600	3.36958000	-2.45457700
H	6.12271900	1.89130100	-0.87686000
H	5.28537400	2.37196000	-2.36342700
H	4.66670000	2.90229100	-0.77517100
H	-0.51389400	-2.15102300	-2.69317100
H	-6.38685500	-2.95747400	2.59389100
H	-8.70001400	-2.86640100	1.70109900
H	-9.17603900	-2.95496600	-0.72422200
H	-7.32944500	-3.21569000	-2.37530600
H	-3.76877600	-2.55435800	-3.19362100
H	-5.15678300	-3.61652500	-3.53432300
H	-3.55153400	-4.29734300	-3.13757300
H	-1.54775600	-1.92001400	5.22598900
H	-2.23993800	-0.17547100	3.60172800
H	-4.04665600	0.86159600	2.80439300
H	-4.29036700	1.38410800	1.12130700
H	-3.32837700	-4.95975700	2.72806300
H	-2.07358100	-4.31929800	4.79948600
H	1.86943300	-5.74625500	-2.63478300
H	0.49036900	-6.62272600	-0.75732200
H	-1.35447900	-5.27371700	0.17313800
H	1.37097900	-3.48299900	-3.58007700
H	-9.79359600	-0.10680600	2.28200900
H	-8.33242000	-0.39182400	4.27833200
H	-5.86379900	-0.10934900	4.06191300
H	-6.30527100	0.67987500	-0.14685700
H	-8.78270700	0.41762000	0.06575800
H	-3.24704600	-4.65682200	-0.42491700

Intermediate (*S*)-II · SbF_6

0 1

C	-5.73393100	-2.96914500	5.47980000
C	-4.77440900	-2.69950800	4.52084300
C	-4.75115300	-1.44826500	3.83870200
C	-5.76737300	-0.49106900	4.15262500
C	-6.73354200	-0.78622600	5.15536900
C	-6.71927200	-2.00107400	5.81203000
C	-3.77536100	-1.13718200	2.82627300
C	-5.79611600	0.74718200	3.46085700
C	-4.90872900	1.03074400	2.43907100
C	-3.93089800	0.03852700	2.09916600
C	-2.63964700	-2.06833800	2.57464900
C	-1.68932700	-2.38522200	3.60541300
C	-2.46587600	-2.63301200	1.31829900
C	-1.69070200	-1.74667900	4.87992300
C	-0.67258500	-3.35801800	3.33569700
C	-1.45045400	-3.59298700	1.02519100
C	-0.76487600	-2.09070500	5.84860300
C	0.24914800	-3.71609300	4.36161400
C	-0.58889700	-3.95132200	2.04872000
C	0.20401500	-3.10076800	5.59820900
C	-4.96356400	2.33790800	1.74310200
C	-6.19695200	2.87040100	1.33736000
C	-3.79594200	3.07061200	1.46843700
C	-6.25398300	4.09062500	0.65314300
C	-3.87300800	4.28954500	0.79082300
C	-5.09689400	4.81848000	0.37371700
C	-1.31635900	-4.20704600	-0.31448500
C	-2.43973700	-4.66540000	-1.02607700
C	-0.04060100	-4.39934100	-0.86823500
C	-2.27269900	-5.30163600	-2.25777100
C	0.11130100	-5.06967300	-2.08721900
C	-0.99992500	-5.52215600	-2.80021400
C	-7.59524200	4.59070800	0.23274000
C	-2.59604000	5.02512300	0.54489400
C	1.48641500	-5.29702800	-2.63139900
C	-3.44909500	-5.78262700	-3.04033100
F	-8.30445300	3.64261000	-0.50745100
F	-8.41025800	4.87997400	1.32523800
F	-7.53913500	5.73431000	-0.53503500
F	-1.59849800	4.18815400	0.06455500
F	-2.08901500	5.58646100	1.71356900
F	-2.73025600	6.06157700	-0.36085800
F	1.94715300	-4.24811800	-3.41793800
F	2.42432300	-5.49102600	-1.63410200
F	1.53460400	-6.42338400	-3.44588700
F	-4.65814300	-5.52897300	-2.42118600
F	-3.51155600	-5.18572600	-4.29977900
F	-3.40015900	-7.15012800	-3.28056900
O	-3.07819900	0.35728300	1.03427500
O	-3.29117200	-2.18423500	0.27430500
P	-2.72335600	-0.61769800	-0.35444400
N	-3.90463600	-0.19118400	-1.50043100
C	-3.85907500	-0.76647300	-2.88896900
C	-5.03495600	0.71668500	-1.18179300
C	-2.47192900	-0.70879900	-3.51377100
C	-2.13782200	0.37111900	-4.34959300
C	-1.51876600	-1.72316600	-3.30530700
C	-0.88583200	0.43014400	-4.97536000
C	-0.25031100	-1.64925400	-3.89920700

C	0.06392800	-0.57649500	-4.74495400
C	-4.89498700	2.01853900	-1.96530600
C	-3.63022500	2.63265100	-2.05333700
C	-6.00353200	2.67382900	-2.52583900
C	-3.47946800	3.87376300	-2.67981200
C	-5.85547000	3.92183300	-3.15179000
C	-4.59433100	4.52701000	-3.22919300
C	-4.45491700	-2.17825300	-2.91308200
C	-6.39513300	0.02743900	-1.27978400
Au	-0.29483000	-0.45779400	-0.53992600
C	5.62928200	-0.28463400	0.33249200
C	6.56495800	-0.89034700	1.19832800
C	7.71592000	-1.38710000	0.62263400
C	7.96993200	-1.32734300	-0.79276000
C	7.06382400	-0.74556800	-1.64655100
C	5.86559600	-0.19668200	-1.09175700
C	4.82288300	0.51463900	-1.66087400
C	4.58444500	0.88309500	-3.07085700
C	3.92478200	0.98747700	-0.55771200
N	4.46110700	0.31577800	0.63568300
C	2.44167700	0.43169700	4.32976800
C	2.19604000	-0.61183800	3.42887300
C	2.88387900	-0.65985200	2.20940400
N	2.68476800	-1.74188100	1.30017000
C	2.94449400	-3.11748100	1.77885700
C	3.81074900	0.35909100	1.90548800
C	4.04829400	1.41154200	2.79434400
C	3.36541500	1.44043200	4.01782200
C	2.35745300	-1.55098100	-0.03595700
C	1.75347500	-0.25517600	-0.44484100
C	2.42212300	0.87998800	-0.75655300
O	2.41637000	-2.51396400	-0.85274100
C	0.57328500	4.40410800	-2.43058100
C	1.55915000	4.52762100	-1.44151300
C	2.15966400	3.38920500	-0.89122700
C	1.78029200	2.09921200	-1.31870500
C	0.79413200	1.98818800	-2.32018800
C	0.19028700	3.12733400	-2.86455800
C	7.05281400	-4.45847600	1.73662100
C	6.47590700	-4.01029400	2.93395700
C	5.15162500	-3.54819100	2.93969200
C	4.39820600	-3.53080800	1.75434400
C	4.98564200	-3.97029000	0.55326100
C	6.30719300	-4.43150700	0.54690700
H	-5.73997900	-3.93213800	5.98072200
H	-4.03829900	-3.45659000	4.26853600
H	-7.48945900	-0.03951800	5.38670900
H	-7.46094700	-2.22534000	6.57178500
H	-6.52945300	1.49510000	3.75872700
H	-2.42412900	-0.97287800	5.08468400
H	-0.78040400	-1.59078100	6.81198800
H	0.98564900	-4.49002200	4.15363600
H	0.16858900	-4.71443200	1.87370200
H	0.90814900	-3.37798800	6.37610300
H	-7.11364600	2.31110800	1.51938500
H	-2.82824100	2.67612800	1.76491700
H	-5.14420300	5.75721000	-0.16920800
H	-3.43407400	-4.52924900	-0.61137000
H	0.84365600	-4.00151900	-0.37439200
H	-0.88299500	-6.04223200	-3.74772800
H	-4.50518200	-0.09415600	-3.47228700
H	-4.90266600	0.98895000	-0.13127400
H	-2.87083100	1.15916500	-4.52000400
H	-1.76153600	-2.59074800	-2.69096700
H	-0.65097500	1.25689400	-5.63968100
H	0.48931500	-2.41922100	-3.69587400
H	1.03945800	-0.52632500	-5.21923600
H	-2.75138300	2.14188000	-1.63158800
H	-6.99242600	2.22777700	-2.47147400
H	-2.49459500	4.33279700	-2.72415800
H	-6.72646700	4.41806500	-3.57002100
H	-4.47926300	5.49405900	-3.70915900
H	-5.49647700	-2.17265300	-2.58006100
H	-4.40871800	-2.59616400	-3.92496200
H	-3.90610100	-2.84455100	-2.23683900
H	-7.17521800	0.67273400	-0.85558300
H	-6.67738100	-0.20393900	-2.31428900
H	-6.37044600	-0.90305700	-0.69974300
H	0.53003500	1.00312500	-2.70442200
H	6.39593600	-0.92046500	2.26886500
H	8.47199700	-1.83280000	1.26277000
H	8.90209600	-1.73163700	-1.17210500
H	7.25574800	-0.67408400	-2.71254700
H	3.59962400	0.51439100	-3.39175300
H	5.35268700	0.48871000	-3.73992600
H	4.56865200	1.97744100	-3.15940900
H	1.89688600	0.46542500	5.26886600
H	1.45494800	-1.37683600	3.65702600
H	2.54774800	-3.18904300	2.80013500
H	2.37046800	-3.79091700	1.13103700
H	4.74839800	2.19290400	2.51217100
H	3.54562600	2.25278100	4.71390600
H	0.11541600	5.29247700	-2.85792900
H	1.86413000	5.50956900	-1.09392700
H	2.90230400	3.52181600	-0.10650000
H	-0.56050500	3.01010400	-3.64465300
H	8.07209900	-4.83490200	1.73197100
H	7.04849600	-4.03380900	3.85675200
H	4.69330700	-3.21302600	3.86993200
H	4.39332300	-3.95346000	-0.35943700
H	6.75236900	-4.77874200	-0.38109700
H	4.18305500	2.05956500	-0.44971600
F	4.83519500	3.66953100	0.84455400
Sb	6.62221600	3.89670300	0.10377600
F	5.88448400	3.17331700	-1.53716100
F	6.16192500	5.65896200	-0.46469600
F	7.22955700	4.51607100	1.80507500
F	8.35792800	3.98757100	-0.67960800
F	6.93856900	2.07314400	0.66671700

Intermediate (*R*)-II

0 1

C	4.61190900	0.51368700	6.62481600
C	3.85229400	0.68753400	5.48204000
C	4.04866800	-0.13999900	4.33795300
C	5.07761500	-1.13127500	4.39511500
C	5.83532300	-1.30148600	5.58786600
C	5.60754300	-0.49791900	6.68778100
C	3.28013000	0.01773000	3.13047700
C	5.33402900	-1.92899100	3.25133000
C	4.65002900	-1.74198900	2.06355500
C	3.63576000	-0.73059900	2.00902200
C	2.16434600	1.00069800	3.09404600
C	1.00191700	0.87574500	3.93230100
C	2.19036700	2.03102600	2.16913000
C	0.80507800	-0.23014000	4.80914000
C	-0.05080800	1.84032500	3.80411500
C	1.18099900	3.03676500	2.06892800
C	-0.37207300	-0.36766600	5.52307400
C	-1.25068300	1.67477900	4.55156700
C	0.07898700	2.92596800	2.89562700
C	-1.41187700	0.59308300	5.39711600
C	4.96108700	-2.60773900	0.90314900
C	6.30042800	-2.88123700	0.58264900
C	3.95091700	-3.22242100	0.14057400
C	6.62011200	-3.76077400	-0.45674800
C	4.29159800	-4.11818300	-0.87859600
C	5.62385100	-4.40943500	-1.18375100
C	1.28092100	4.05348800	0.99974000
C	2.50937500	4.68317700	0.72094100
C	0.16967700	4.34737800	0.19457400
C	2.61920500	5.57316400	-0.34603400
C	0.30702900	5.23201900	-0.88760600
C	1.52221500	5.85301800	-1.17235900
C	8.06156900	-3.96395900	-0.78262000
C	3.18652700	-4.76162200	-1.64829700
C	-0.88182800	5.47063900	-1.75459300
C	3.92319000	6.22312100	-0.66610800
F	8.71754300	-2.75969900	-1.01381300
F	8.75619100	-4.57076900	0.26290800
F	8.25909800	-4.75085000	-1.90035500
F	2.42101300	-5.62315000	-0.86985300
F	3.63943700	-5.49864400	-2.72646800
F	2.29475400	-3.82045000	-2.15945800

F	-1.37585600	4.28284300	-2.30825600
F	-1.94988600	6.02724600	-1.05869600
F	-0.62331500	6.31186400	-2.81857600
F	4.89913200	5.98603800	0.28126500
F	4.44453700	5.77937400	-1.88576600
F	3.80876900	7.60125400	-0.79578400
O	3.01256900	-0.59878500	0.77089600
O	3.16210600	1.97868200	1.14789100
P	2.57035500	0.85763700	-0.09306600
N	3.71983100	0.97067300	-1.32794000
C	3.26607900	1.32178000	-2.71833300
C	5.18090700	0.87205800	-1.00324700
C	1.89724600	0.71072400	-3.01070800
C	1.72123700	-0.68244000	-2.88311700
C	0.79584100	1.49183800	-3.39500600
C	0.47352400	-1.27623700	-3.10342300
C	-0.45262500	0.89679000	-3.63100200
C	-0.62436900	-0.48359800	-3.47684800
C	5.90704200	0.08813300	-2.08748800
C	5.43415400	-1.19366100	-2.42305200
C	7.03788300	0.58398500	-2.75357800
C	6.05918200	-1.95600600	-3.41286600
C	7.67890600	-0.18529600	-3.73775900
C	7.18965900	-1.45263800	-4.07590800
C	3.33160500	2.83117200	-2.94717000
C	5.79768000	2.23348700	-0.66364600
Au	0.10723000	0.87797800	-0.13974600
C	-4.43987900	-1.11235100	1.89004200
C	-5.68975200	-0.51642800	1.83547100
C	-6.74737100	-0.42238100	2.79388700
C	-7.93676700	0.14186900	2.39900700
C	-8.11229200	0.62705800	1.05510200
C	-7.11447300	0.57423400	0.10446600
C	-5.88589000	0.00076700	0.50032300
N	-4.77551400	-0.20682300	-0.23362100
C	-3.82562200	-1.03033700	0.52453100
C	-2.36269100	-0.63981800	0.40822900
C	-1.92769200	0.61618100	0.14704100
C	-1.42229400	-1.77757100	0.61905700
C	-1.51406800	-2.95811400	-0.14342100
C	-0.59296400	-3.99525600	0.05165000
C	0.40844500	-3.88292100	1.02910000
C	0.48656700	-2.72263500	1.81369900
C	-0.42196400	-1.67796600	1.60753100
C	-3.79212900	-1.81968500	3.01326400
N	-3.53776800	2.24256100	-0.86155600
O	-2.54977700	2.66414300	1.16771700
C	-2.72475000	1.86778100	0.19513200
C	-3.95546200	1.36422700	-1.90436100
C	-4.59587000	0.14551500	-1.60501100
C	-5.06798200	-0.69563500	-2.61969400
C	-4.90949900	-0.31380700	-3.95743200
C	-4.28460000	0.90390500	-4.26757200
C	-3.81371300	1.73989400	-3.24803000
C	-4.10142600	3.61527900	-0.85152600
C	-5.49905400	3.68572500	-0.28114700
C	-6.60463600	3.88279100	-1.12423600
C	-7.89290200	4.02929700	-0.58797900
C	-8.08141700	3.97975300	0.80055700
C	-6.98149000	3.76855700	1.64798800
C	-5.69759200	3.61944600	1.11115700
H	4.45083600	1.15961500	7.48234100
H	3.10486100	1.47418700	5.44745600
H	6.60433000	-2.07009500	5.61171600
H	6.19019100	-0.62609000	7.59434000
H	6.08277600	-2.71668600	3.32217900
H	1.58551800	-0.97996100	4.90361400
H	-0.50828500	-1.22225400	6.17864200
H	-2.04451800	2.40494200	4.41789200
H	-0.74013500	3.63591800	2.81389100
H	-2.33180200	0.46757800	5.95995100
H	7.09873200	-2.37916000	1.12613400
H	2.90501400	-2.98945200	0.33648500
H	5.87642500	-5.09861000	-1.98317500
H	3.37584300	4.46631100	1.33786500
H	-0.78790700	3.86171700	0.40274600
H	1.61951100	6.53323200	-2.01430800
H	3.99674600	0.83228400	-3.37844200
H	5.22267300	0.25830000	-0.08625200
H	2.56436600	-1.30917800	-2.59633700
H	0.88836600	2.56964900	-3.50026800
H	0.35850700	-2.34844000	-2.97811100
H	-1.29500600	1.51775300	-3.92058500
H	-1.60063100	-0.93411500	-3.63787700
H	4.56247300	-1.58410000	-1.89645700
H	7.43219000	1.56603100	-2.50892000
H	5.66237000	-2.93467800	-3.67560200
H	8.55767400	0.20785700	-4.23995200
H	7.68237000	-2.04346900	-4.84171000
H	4.34926200	3.20178800	-2.80102000
H	3.02975600	3.08745600	-3.96876600
H	2.67571500	3.36286500	-2.23971400
H	6.80218500	2.09665200	-0.24619200
H	5.88167600	2.88744000	-1.53824600
H	5.17976400	2.73867500	0.08648000
H	-6.61490100	-0.81632200	3.79677500
H	-8.77025600	0.20863200	3.08975400
H	-9.07745000	1.04238700	0.77952700
H	-7.26495500	0.92962300	-0.90906100
H	-3.90963300	-2.05722700	0.11219800
H	-2.28733900	-3.06235500	-0.90287200
H	-0.65717800	-4.89457700	-0.55357100
H	1.10169800	-4.70531100	1.18789700
H	1.23826100	-2.63359000	2.59715000
H	-0.37915300	-0.78455700	2.23217900
H	-4.37732600	-1.75545500	3.93427900
H	-3.67817100	-2.87899800	2.74350100
H	-2.78473900	-1.42090600	3.19347000
H	-5.56054700	-1.62534800	-2.35129800
H	-5.27538700	-0.96081500	-4.74740200
H	-4.16075900	1.20164500	-5.30444400
H	-3.32356100	2.68123400	-3.48564400
H	-4.08542300	3.99779500	-1.87770400
H	-3.42317400	4.22731100	-0.24977500
H	-6.45130200	3.94183000	-2.20113000
H	-8.73996700	4.19755700	-1.24684100
H	-9.07466200	4.11435600	1.22009300
H	-7.12637500	3.72939400	2.72413800
H	-4.83329700	3.46963700	1.75533500
F	-4.38258200	-3.43995000	-1.35781200
Sb	-5.92732800	-4.25696300	-0.49790800
F	-6.75457800	-4.38964100	-2.21619100
F	-6.63911900	-2.45967600	-0.34764600
F	-5.00291900	-3.92262900	1.17117400
F	-7.44127700	-4.95556900	0.42471000
F	-5.10904600	-5.97193100	-0.64681500

Intermediate (*R*)-II · SbF_6

0 1

C	4.61190900	0.51368700	6.62481600
C	3.85229400	0.68753400	5.48204000
C	4.04866800	-0.13999900	4.33795300
C	5.07761500	-1.13127500	4.39511500
C	5.83532300	-1.30148600	5.58786600
C	5.60754300	-0.49791900	6.68778100
C	3.28013000	0.01773000	3.13047700
C	5.33402900	-1.92899100	3.25133000
C	4.65002900	-1.74198900	2.06355500
C	3.63576000	-0.73059900	2.00902200
C	2.16434600	1.00069800	3.09404600
C	1.00191700	0.87574500	3.93230100
C	2.19036700	2.03102600	2.16913000
C	0.80507800	-0.23014000	4.80914000
C	-0.05080800	1.84032500	3.80411500
C	1.18099900	3.03676500	2.06892800
C	-0.37207300	-0.36766600	5.52307400
C	-1.25068300	1.67477900	4.55156700
C	0.07898700	2.92596800	2.89562700
C	-1.41187700	0.59308300	5.39711600
C	4.96108700	-2.60773900	0.90314900
C	6.30042800	-2.88123700	0.58264900
C	3.95091700	-3.22242100	0.14057400
C	6.62011200	-3.76077400	-0.45674800

C	4.29159800	-4.11818300	-0.87859600
C	5.62385100	-4.40943500	-1.18375100
C	1.28092100	4.05348800	0.99974000
C	2.50937500	4.68317700	0.72094100
C	0.16967700	4.34737800	0.19457400
C	2.61920500	5.57316400	-0.34603400
C	0.30702900	5.23201900	-0.88760600
C	1.52221500	5.85301800	-1.17235900
C	8.06156900	-3.96395900	-0.78262000
C	3.18652700	-4.76162200	-1.64829700
C	-0.88182800	5.47063900	-1.75459300
C	3.92319000	6.22312100	-0.66610800
F	8.71754300	-2.75969900	-1.01381300
F	8.75619100	-4.57076900	0.26290800
F	8.25909800	-4.75085000	-1.90035500
F	2.42101300	-5.62315000	-0.86985300
F	3.63943700	-5.49864400	-2.72646800
F	2.29475400	-3.82045000	-2.15945800
F	-1.37585600	4.28284300	-2.30825600
F	-1.94988600	6.02724600	-1.05869600
F	-0.62331500	6.31186400	-2.81857600
F	4.89913200	5.98603800	0.28126500
F	4.44453700	5.77937400	-1.88576600
F	3.80876900	7.60125400	-0.79578400
O	3.01256900	-0.59878500	0.77089600
O	3.16210600	1.97868200	1.14789100
P	2.57035500	0.85763700	-0.09306600
N	3.71983100	0.97067300	-1.32794000
C	3.26607900	1.32178000	-2.71833300
C	5.18090700	0.87205800	-1.00324700
C	1.89724600	0.71072400	-3.01070800
C	1.72123700	-0.68244000	-2.88311700
C	0.79584100	1.49183800	-3.39500600
C	0.47352400	-1.27623700	-3.10342300
C	-0.45262500	0.89679000	-3.63100200
C	-0.62436900	-0.48359800	-3.47684800
C	5.90704200	0.08813300	-2.08748800
C	5.43415400	-1.19366100	-2.42305200
C	7.03788300	0.58398500	-2.75357800
C	6.05918200	-1.95600600	-3.41286600
C	7.67890600	-0.18529600	-3.73775900
C	7.18965900	-1.45263800	-4.07590800
C	3.33160500	2.83117200	-2.94717000
C	5.79768000	2.23348700	-0.66364600
Au	0.10723000	0.87797800	-0.13974600
C	-4.43987900	-1.11235100	1.89004200
C	-5.68975200	-0.51642800	1.83547100
C	-6.74737100	-0.42238100	2.79388700
C	-7.93676700	0.14186900	2.39900700
C	-8.11229200	0.62705800	1.05510200
C	-7.11447300	0.57423400	0.10446600
C	-5.88589000	0.00076700	0.50032300
N	-4.77551400	-0.20682300	-0.23362100
C	-3.82562200	-1.03033700	0.52453100
C	-2.36269100	-0.63981800	0.40822900
C	-1.92769200	0.61618100	0.14704100
C	-1.42229400	-1.77757100	0.61905700
C	-1.51406800	-2.95811400	-0.14342100
C	-0.59296400	-3.99525600	0.05165000
C	0.40844500	-3.88292100	1.02910000
C	0.48656700	-2.72263500	1.81369900
C	-0.42196400	-1.67796600	1.60753100
C	-3.79212900	-1.81968500	3.01326400
N	-3.53776800	2.24256100	-0.86155600
O	-2.54977700	2.66414300	1.16771700
C	-2.72475000	1.86778100	0.19513200
C	-3.95546200	1.36422700	-1.90436100
C	-4.59587000	0.14551500	-1.60501100
C	-5.06798200	-0.69563500	-2.61969400
C	-4.90949900	-0.31380700	-3.95743200
C	-4.28460000	0.90390500	-4.26757200
C	-3.81371300	1.73989400	-3.24803000
C	-4.10142600	3.61527900	-0.85152600
C	-5.49905400	3.68572500	-0.28114700
C	-6.60463600	3.88279100	-1.12423600
C	-7.89290200	4.02929700	-0.58797900
C	-8.08141700	3.97975300	0.80055700

C	-6.98149000	3.76855700	1.64798800
C	-5.69759200	3.61944600	1.11115700
H	4.45083600	1.15961500	7.48234100
H	3.10486100	1.47418700	5.44745600
H	6.60433000	-2.07009500	5.61171600
H	6.19019100	-0.62609000	7.59434000
H	6.08277600	-2.71668600	3.32217900
H	1.58551800	-0.97996100	4.90361400
H	-0.50828500	-1.22225400	6.17864200
H	-2.04451800	2.40494200	4.41789200
H	-0.74013500	3.63591800	2.81389100
H	-2.33180200	0.46757800	5.95995100
H	7.09873200	-2.37916000	1.12613400
H	2.90501400	-2.98945200	0.33648500
H	5.87642500	-5.09861000	-1.98317500
H	3.37584300	4.46631100	1.33786500
H	-0.78790700	3.86171700	0.40274600
H	1.61951100	6.53323200	-2.01430800
H	3.99674600	0.83228400	-3.37844200
H	5.22267300	0.25830000	-0.08625200
H	2.56436600	-1.30917800	-2.59633700
H	0.88836600	2.56964900	-3.50026800
H	0.35850700	-2.34844000	-2.97811100
H	-1.29500600	1.51775300	-3.92058500
H	-1.60063100	-0.93411500	-3.63787700
H	4.56247300	-1.58410000	-1.89645700
H	7.43219000	1.56603100	-2.50892000
H	5.66237000	-2.93467800	-3.67560200
H	8.55767400	0.20785700	-4.23995200
H	7.68237000	-2.04346900	-4.84171000
H	4.34926200	3.20178800	-2.80102000
H	3.02975600	3.08745600	-3.96876600
H	2.67571500	3.36286500	-2.23971400
H	6.80218500	2.09665200	-0.24619200
H	5.88167600	2.88744000	-1.53824600
H	5.17976400	2.73867500	0.08648000
H	-6.61490100	-0.81632200	3.79677500
H	-8.77025600	0.20863200	3.08975400
H	-9.07745000	1.04238700	0.77952700
H	-7.26495500	0.92962300	-0.90906100
H	-3.90963300	-2.05722700	0.11219800
H	-2.28733900	-3.06235500	-0.90287200
H	-0.65717800	-4.89457700	-0.55357100
H	1.10169800	-4.70531100	1.18789700
H	1.23826100	-2.63359000	2.59715000
H	-0.37915300	-0.78455700	2.23217900
H	-4.37732600	-1.75545500	3.93427900
H	-3.67817100	-2.87899800	2.74350100
H	-2.78473900	-1.42090600	3.19347000
H	-5.56054700	-1.62534800	-2.35129800
H	-5.27538700	-0.96081500	-4.74740200
H	-4.16075900	1.20164500	-5.30444400
H	-3.32356100	2.68123400	-3.48564400
H	-4.08542300	3.99779500	-1.87770400
H	-3.42317400	4.22731100	-0.24977500
H	-6.45130200	3.94183000	-2.20113000
H	-8.73996700	4.19755700	-1.24684100
H	-9.07466200	4.11435600	1.22009300
H	-7.12637500	3.72939400	2.72413800
H	-4.83329700	3.46963700	1.75533500
F	-4.38258200	-3.43995000	-1.35781200
Sb	-5.92732800	-4.25696300	-0.49790800
F	-6.75457800	-4.38964100	-2.21619100
F	-6.63911900	-2.45967600	-0.34764600
F	-5.00291900	-3.92262900	1.17117400
F	-7.44127700	-4.95556900	0.42471000
F	-5.10904600	-5.97193100	-0.64681500

Intermediate (*S*)-III

0 1

C	3.32978600	2.37031000	6.48799600
C	2.59367400	2.12656500	5.34307200
C	2.98671900	1.11334700	4.42113700
C	4.17960800	0.37353100	4.69863900
C	4.91008400	0.63149700	5.89291300
C	4.49514800	1.60971400	6.77559300
C	2.25072700	0.84676700	3.21358800

C	4.61502700	-0.61402600	3.77705300
C	3.95763500	-0.83049200	2.57943800
C	2.79890000	-0.03657600	2.28882300
C	0.93619000	1.50723500	2.97783500
C	-0.17381000	1.30283900	3.86753200
C	0.75148200	2.31085400	1.86155500
C	-0.12449400	0.40697000	4.97574100
C	-1.40076700	1.99803100	3.61209800
C	-0.46649900	3.00462900	1.58491000
C	-1.21472300	0.25037000	5.81352900
C	-2.49681700	1.84087700	4.50821600
C	-1.51597500	2.83730600	2.47249900
C	-2.40931900	0.98869300	5.59260700
C	4.43681100	-1.87028700	1.63786300
C	5.80429500	-1.97341400	1.34100000
C	3.54686400	-2.75915200	1.00905900
C	6.26246400	-2.91948900	0.41630400
C	4.02429200	-3.70725300	0.10053800
C	5.38325600	-3.80253200	-0.21093000
C	-0.61030900	3.88988900	0.40692400
C	0.41123400	4.79326700	0.05961100
C	-1.79904000	3.88020100	-0.34296400
C	0.23677800	5.66568100	-1.01546700
C	-1.96280100	4.78548000	-1.40156900
C	-0.95368800	5.68111500	-1.75424300
C	7.72389700	-2.94847900	0.11912100
C	3.03483600	-4.64030000	-0.51807400
C	-3.25065900	4.76654300	-2.16201300
C	1.31234800	6.61754300	-1.41658200
F	8.21547300	-1.68711800	-0.22412900
F	8.47573200	-3.34148900	1.22461200
F	8.05947700	-3.80079300	-0.91164300
F	1.90582700	-3.98201900	-0.98528100
F	2.56876800	-5.58141900	0.39809400
F	3.55626300	-5.35624900	-1.58077000
F	-3.35984900	3.66499700	-3.00612800
F	-4.35382200	4.73169300	-1.33058800
F	-3.39683900	5.88417000	-2.97449100
F	2.44219700	6.53294900	-0.62456900
F	1.73169800	6.40381600	-2.73114300
F	0.89282800	7.94199100	-1.37496300
O	2.20112100	-0.25884600	1.04264200
O	1.79597300	2.37122900	0.92262700
P	1.76176400	0.94153500	-0.13760500
N	3.15717000	1.11375400	-1.09693200
C	3.13870900	2.00657000	-2.30510600
C	4.42985100	0.41547300	-0.79332300
C	1.92082100	1.77737300	-3.18832900
C	2.00855500	0.86878900	-4.25765100
C	0.71269700	2.46784600	-2.98330200
C	0.91670600	0.66101400	-5.10990900
C	-0.39123100	2.24144600	-3.81698900
C	-0.28943400	1.34175800	-4.88679900
C	4.71382500	-0.63259300	-1.86577400
C	3.66837800	-1.47610800	-2.29043600
C	6.00062000	-0.83841000	-2.38869200
C	3.90474900	-2.50436300	-3.20788500
C	6.24149400	-1.86958600	-3.31077400
C	5.19620600	-2.70730800	-3.72136200
C	3.31439500	3.47306100	-1.89867200
C	5.57711300	1.37743500	-0.48949200
Au	-0.53116200	0.28373100	-0.69093400
C	-5.92712200	-3.16885200	-0.27744400
C	-6.75907300	-3.82825300	0.63552400
C	-7.99650300	-4.27705400	0.16771000
C	-8.39752700	-4.06445200	-1.17383100
C	-7.56588100	-3.39350900	-2.07352800
C	-6.31032100	-2.93912700	-1.62762300
C	-5.23002500	-2.23827000	-2.27798300
C	-5.17868800	-1.82674800	-3.71388200
C	-4.24461400	-2.04439500	-1.33114200
N	-4.66248500	-2.60745500	-0.09920300
C	-2.22811300	-2.85011600	3.34627400
C	-2.55955100	-1.60262600	2.80827200
C	-3.38934900	-1.51042100	1.68264900
N	-3.80358300	-0.21348900	1.23681600
C	-4.75395900	0.50361500	2.10852900
C	-3.87124100	-2.68357300	1.07396100
C	-3.52962500	-3.93522900	1.60971000
C	-2.72315100	-4.02026500	2.74933100
C	-3.38600000	0.36202900	0.05255700
C	-2.45034700	-0.42936700	-0.79032100
C	-2.86874800	-1.51853700	-1.48902500
O	-3.73967900	1.53351200	-0.25912300
C	-0.25517700	-3.92982400	-3.93873500
C	-1.27575600	-4.52060800	-3.17903900
C	-2.13190400	-3.72513500	-2.41022800
C	-1.97786100	-2.32341600	-2.36753100
C	-0.96532200	-1.74000500	-3.15560700
C	-0.10905400	-2.53567400	-3.92643500
C	-8.67593900	-1.30943700	2.11993100
C	-7.91843300	-1.28164000	3.29801900
C	-6.63808500	-0.70563000	3.29220200
C	-6.11591100	-0.14909500	2.11578400
C	-6.87494400	-0.19337800	0.93319600
C	-8.14766300	-0.77199700	0.93482500
H	3.01995100	3.15395700	7.17228100
H	1.71341300	2.72514100	5.12937800
H	5.80765100	0.05022200	6.09098900
H	5.05845500	1.80831800	7.68171800
H	5.48144600	-1.22247800	4.03253900
H	0.78137500	-0.16301000	5.15768800
H	-1.15780800	-0.43772300	6.65128000
H	-3.40489800	2.40986200	4.32060300
H	-2.45536300	3.36049900	2.29998500
H	-3.24920800	0.87314700	6.27021700
H	6.50910900	-1.27987800	1.79761900
H	2.48175600	-2.69024200	1.21287000
H	5.73940900	-4.52640700	-0.93710700
H	1.33352100	4.81149700	0.63280000
H	-2.58637000	3.14934600	-0.13197600
H	-1.08756900	6.37691500	-2.57835900
H	4.01936500	1.68772100	-2.88186500
H	4.24142600	-0.14121100	0.12832100
H	2.94331000	0.33507600	-4.42788100
H	0.62636600	3.20211400	-2.18237200
H	1.00782400	-0.02131900	-5.95039300
H	-1.32649000	2.76194200	-3.63108500
H	-1.14111300	1.17418300	-5.53875700
H	2.65777600	-1.33108500	-1.90565900
H	6.82803000	-0.20622600	-2.07918300
H	3.08319200	-3.14743100	-3.51303200
H	7.24423900	-2.01627100	-3.70133600
H	5.38221100	-3.50732500	-4.43153300
H	4.25580600	3.61939400	-1.36119200
H	3.30502700	4.12043500	-2.78260900
H	2.50929000	3.79385000	-1.22634500
H	6.44368600	0.82044500	-0.10957100
H	5.90178400	1.94025100	-1.37344100
H	5.25821400	2.08563100	0.28479000
H	-0.87169800	-0.65437200	-3.19502500
H	-6.46741200	-3.96231500	1.67407800
H	-8.66900500	-4.79032500	0.84976700
H	-9.36731700	-4.42887300	-1.50115200
H	-7.87740400	-3.22671500	-3.10205700
H	-4.28643400	-1.22500800	-3.91781900
H	-6.05699600	-1.22811600	-3.98750900
H	-5.15455600	-2.70079800	-4.37870400
H	-1.58258400	-2.90662100	4.21796000
H	-2.17911900	-0.68409700	3.25263100
H	-4.34292400	0.53555300	3.12684100
H	-4.81376400	1.52561100	1.71469100
H	-3.89342600	-4.83121800	1.11307300
H	-2.46503300	-4.99308800	3.15625600
H	0.40823000	-4.54723600	-4.53834100
H	-1.40191300	-5.59948300	-3.18415000
H	-2.92196300	-4.19117900	-1.82319700
H	0.66075100	-2.05800900	-4.52954600
H	-9.66479200	-1.75954100	2.11619000
H	-8.32010100	-1.70625400	4.21386400
H	-6.04046900	-0.68652800	4.20356700
H	-6.45944900	0.22617600	0.01756100
H	-8.72321500	-0.81821700	0.01466100

Intermediate (*R*)-III

0 1

C	3.95399000	-0.03830000	6.64509000
C	3.13954200	0.13765000	5.54122700
C	3.48805600	-0.42026500	4.27651100
C	4.71955000	-1.13951600	4.17605100
C	5.53330700	-1.32052800	5.32980000
C	5.15953500	-0.78390200	6.54625100
C	2.66581700	-0.25033400	3.10715400
C	5.11540700	-1.65939500	2.91786600
C	4.37025000	-1.44335600	1.77219700
C	3.15112700	-0.69655400	1.87858300
C	1.34111800	0.41481400	3.23105000
C	0.28207700	-0.15001100	4.02357900
C	1.07362100	1.55953800	2.49703200
C	0.40268400	-1.40427100	4.68910400
C	-0.98446300	0.51937000	4.05782600
C	-0.16459100	2.26889700	2.55816400
C	-0.67620500	-1.96029300	5.35287900
C	-2.07713400	-0.07397200	4.75112300
C	-1.16600600	1.73728500	3.35123000
C	-1.92960800	-1.29145900	5.38794900
C	4.84109500	-2.00738200	0.48625600
C	6.19873200	-1.89457000	0.14406900
C	3.98509000	-2.70434100	-0.38714100
C	6.69232000	-2.48125100	-1.02498500
C	4.50304600	-3.29650400	-1.54530000
C	5.85822800	-3.20607100	-1.87320900
C	-0.41386000	3.43205000	1.67891500
C	0.57679000	4.39849500	1.42270700
C	-1.65643200	3.54539800	1.03224900
C	0.32361400	5.44272000	0.53096800
C	-1.88535100	4.59795300	0.13606200
C	-0.90908000	5.55883200	-0.12465100
C	8.13183700	-2.27704300	-1.35792600
C	3.56103700	-4.00332200	-2.46139000
C	-3.18874400	4.62254300	-0.58890300
C	1.37063200	6.46305500	0.23921300
F	8.46961400	-0.92882500	-1.40727600
F	8.97678100	-2.83717000	-0.40058500
F	8.49080600	-2.82558300	-2.57373200
F	2.93169800	-5.08725900	-1.86284300
F	4.17681600	-4.48539800	-3.60258200
F	2.53767600	-3.16016300	-2.89735300
F	-3.33456600	3.51753100	-1.43747000
F	-4.28332500	4.58818400	0.26095900
F	-3.34272300	5.74041400	-1.39095200
F	2.63233200	6.08840200	0.66809200
F	1.48000900	6.72300400	-1.12673100
F	1.10283700	7.69447900	0.82960300
O	2.47700900	-0.50439800	0.67536400
O	2.00614700	1.93549100	1.50671200
P	1.70262000	0.94247700	0.06782100
N	2.79977400	1.52829200	-1.08105800
C	2.24378000	2.12494800	-2.34178800
C	4.25829200	1.64829500	-0.76375300
C	1.08806700	1.27582300	-2.87554800
C	1.24517300	-0.11864000	-3.01005700
C	-0.14973000	1.84238600	-3.22027100
C	0.18585100	-0.92750100	-3.43560600
C	-1.20976200	1.03580800	-3.65946800
C	-1.05413200	-0.35160100	-3.75454000
C	5.09134800	1.26277800	-1.97888400
C	4.90094700	-0.00767900	-2.55054200
C	6.04970500	2.11749100	-2.54483400
C	5.63381900	-0.41302500	-3.66889400
C	6.79761800	1.71035700	-3.66074000
C	6.58933300	0.44838600	-4.23041900
C	1.86932800	3.59345000	-2.12682800
C	4.61451800	3.00393400	-0.14648300
Au	-0.69959300	0.39696600	-0.10418100
C	-4.51957200	-3.32938300	1.19543900
C	-5.82644700	-3.75459100	0.76297000
C	-6.77158800	-4.65479700	1.29349200
C	-7.97606300	-4.84667300	0.61404600
C	-8.25720600	-4.14236800	-0.58244800
C	-7.34014700	-3.23863100	-1.12327200
C	-6.12854300	-3.06333700	-0.44237600
N	-5.05034200	-2.22442700	-0.73076400
C	-4.06925300	-2.39686900	0.27678000
C	-2.75473700	-1.73603200	0.20569600
C	-2.59425400	-0.38945000	0.07205500
C	-1.57658000	-2.64478800	0.30065400
C	-1.43193600	-3.72348600	-0.59154200
C	-0.30328400	-4.55027000	-0.52951000
C	0.68204300	-4.32850600	0.44755600
C	0.52916600	-3.27543700	1.36103800
C	-0.59206800	-2.43943400	1.28567800
C	-3.78792600	-3.80524200	2.41184900
N	-4.70560500	0.64957600	-0.69705300
O	-3.76845600	1.26978600	1.30961700
C	-3.72676200	0.55094500	0.26705600
C	-4.70264000	-0.09521300	-1.91816200
C	-4.90242500	-1.48792700	-1.93405500
C	-4.97748800	-2.16488200	-3.16337500
C	-4.89184700	-1.46253200	-4.36931300
C	-4.71011300	-0.07059700	-4.35389900
C	-4.61216700	0.60215200	-3.13193400
C	-5.85258300	1.55814400	-0.48202900
C	-7.13396100	0.77790500	-0.30621800
C	-8.22221400	0.96540800	-1.17000300
C	-9.41345600	0.24727000	-0.97760000
C	-9.51529200	-0.66824200	0.07788500
C	-8.42168800	-0.87067500	0.93536300
C	-7.23860200	-0.15145300	0.74399300
H	3.67353000	0.40123900	7.59727000
H	2.22776000	0.72000400	5.63099800
H	6.46012000	-1.88114000	5.23143100
H	5.78419800	-0.91850600	7.42350000
H	6.02607700	-2.25425400	2.86286300
H	1.34940200	-1.93615900	4.65720700
H	-0.57022700	-2.92422700	5.84095100
H	-3.03544400	0.43867100	4.74007400
H	-2.14220100	2.21506500	3.38791100
H	-2.76947700	-1.74751200	5.90243000
H	6.86793700	-1.31726200	0.77944900
H	2.91965000	-2.77007700	-0.16596900
H	6.24598500	-3.66170400	-2.77846100
H	1.54800300	4.31517900	1.90039900
H	-2.43030100	2.78996900	1.20417900
H	-1.09082200	6.36331700	-0.83190900
H	3.07336300	2.06757600	-3.06202500
H	4.44111400	0.87978900	0.00668700
H	2.19614300	-0.58237600	-2.75713000
H	-0.31009900	2.91303300	-3.13573600
H	0.32583300	-2.00211600	-3.50002300
H	-2.16461400	1.49381600	-3.90157600
H	-1.89180500	-0.97410800	-4.06040300
H	4.16563300	-0.67709800	-2.10445800
H	6.22656400	3.10115500	-2.11948300
H	5.45301200	-1.39186700	-4.10958900
H	7.54037200	2.37989800	-4.08404500
H	7.16444700	0.13713100	-5.09693300
H	2.70866500	4.15106000	-1.70141100
H	1.59015500	4.07840600	-3.06876600
H	1.02145200	3.67474800	-1.42942000
H	5.64219900	2.98538900	0.23505400
H	4.53792100	3.82406800	-0.86909500
H	3.93785600	3.21497400	0.68809100
H	-6.56336900	-5.19533300	2.21386400
H	-8.71318400	-5.54221200	1.00541700
H	-9.20918400	-4.29858100	-1.08228000
H	-7.57270800	-2.67138200	-2.02095900
H	-2.20145900	-3.89424200	-1.34347500
H	-0.18353000	-5.36471000	-1.23839400
H	1.54656000	-4.98547300	0.50379400
H	1.27189900	-3.10929200	2.14114400
H	-0.73117900	-1.64122100	2.01569800
H	-4.48944900	-4.19071900	3.16144300
H	-3.07986000	-4.61048200	2.17007100
H	-3.21078900	-2.99543100	2.87456100
H	-5.11255200	-3.24328700	-3.15346800
H	-4.96291700	-1.99520900	-5.31250600
H	-4.64196300	0.48331100	-5.28542400

H	-4.47076400	1.67974800	-3.09992200
H	-5.93026300	2.25550100	-1.32628500
H	-5.61647300	2.13679500	0.41756000
H	-8.13753200	1.67550700	-1.99150600
H	-10.25428700	0.40072900	-1.64825700
H	-10.43234800	-1.23140900	0.22691900
H	-8.48898200	-1.59985500	1.73806800
H	-6.38482600	-0.30902200	1.40270900

SbF_6^-

-1 1

Sb	0.00001800	-0.00009400	-0.00010000
F	1.26595200	-0.28601400	1.42063900
F	-1.05307400	1.11428500	1.16299000
F	-1.26647300	0.28700700	-1.42024400
F	1.05300000	-1.11436200	-1.16274400
F	-0.99581100	-1.54248300	0.57627400
F	0.99630200	1.54209800	-0.57634800

SbF_6H

0 1

Sb	0.17533400	-0.00032500	-0.00566600
F	-0.62589200	-0.01344000	-1.74657100
F	2.00664200	-0.01138400	-0.43585900
F	0.19492500	0.01650700	1.88867600
F	-2.09566300	0.00923600	0.33523500
F	-0.09612600	-1.88143200	0.03548500
F	-0.08075700	1.88314600	0.00197000
H	-2.67017400	-0.00713000	-0.42144400

TS-2aa – Conf1

0 1

C	-1.58419300	1.11782300	-0.99285200
C	-0.25096800	1.59086600	-0.88135000
C	-0.03665100	-1.84974500	0.22550000
C	1.14299300	-1.60020500	-0.40682400
C	1.18243000	-0.29553400	-1.10447700
N	0.70236100	0.72699000	-0.28870700
C	1.03693100	0.90524300	1.13145100
C	0.15480200	2.83227900	-1.38012400
H	1.20502900	3.09194400	-1.27608200
C	-2.47920200	2.00443900	-1.63199000
H	-3.51912100	1.76900100	-1.74923700
C	-2.07585200	3.24990700	-2.12178100
H	-2.81882000	3.88008400	-2.60215900
C	-0.75324800	3.68562500	-2.00519600
H	-0.43957300	4.64680500	-2.39737800
C	-3.42798000	-0.36426700	-0.15533100
C	-1.31838200	-1.14677800	0.36899000
C	-2.22653400	-1.74333000	1.23744700
C	-3.53634200	-1.26319200	0.93799500
C	-1.95135400	-2.82598100	2.23601900
H	-0.98933300	-2.69321500	2.74177500
H	-1.95624200	-3.82218300	1.77149800
H	-2.72704500	-2.82941800	3.00937200
O	1.43435000	-0.13617800	-2.31629800
H	1.54428000	-0.00353800	1.47689500
H	0.11020400	1.01379300	1.71476400
N	-2.03852400	-0.14472200	-0.43186200
H	-0.10739400	-2.83582700	0.68478200
C	-4.78047100	-1.64970200	1.46688300
H	-4.82701300	-2.33194100	2.31186600
C	-5.94501900	-1.17875000	0.86740500
H	-6.91933400	-1.46464000	1.25065500
C	-5.84930200	-0.37269600	-0.28416800
H	-6.75430200	-0.07316600	-0.80467000
C	-4.61539300	0.02779500	-0.80729300
H	-4.63273100	0.55086900	-1.75115600
C	2.26463400	-2.55324900	-0.46945600
C	2.47063700	-3.50206100	0.55599200
C	3.17917900	-2.51220200	-1.54364000
C	3.54324800	-4.39652700	0.49952900
H	1.80520800	-3.52126500	1.41744400
C	4.25397100	-3.40728200	-1.59492100
H	3.02042700	-1.79325700	-2.34225200
C	4.44042300	-4.35506000	-0.57899200
H	3.68907900	-5.11623200	1.29991700
H	4.94345000	-3.36791900	-2.43310200
H	5.27547800	-5.04800000	-0.62164400
C	1.93141900	2.10985100	1.32801600
C	1.47948600	3.24426200	2.01530900
C	3.22646200	2.10566700	0.78043200
C	2.31095700	4.36593600	2.15894000
H	0.47175600	3.25374300	2.42673800
C	4.05852600	3.22124800	0.92100700
H	3.57261400	1.22771300	0.23447700
C	3.60091600	4.35599700	1.61199800
H	1.95207200	5.24304100	2.68942100
H	5.05763200	3.21129100	0.49542000
H	4.24528800	5.22359700	1.71992700

TS-2aa – Conf2

0 1

C	-1.46898900	1.73203100	-0.33801400
C	-0.49743500	2.29497500	0.53005500
C	1.07868100	-0.71975000	-0.69631100
C	2.08204200	0.19325900	-0.59018000
C	1.59091100	1.56579300	-0.34829500
N	0.70870100	1.58804700	0.73682100
C	1.00934500	0.96362300	2.03763900
C	-0.65494100	3.55106800	1.12518600
H	0.16036700	3.91261900	1.74710200
C	-2.59028800	2.55721500	-0.58074200
H	-3.38448600	2.24701000	-1.22996500
C	-2.75092900	3.80898800	0.01971800
H	-3.64407200	4.38184700	-0.21193600
C	-1.79123500	4.32406400	0.89584000
H	-1.91055800	5.29956000	1.35451600
C	-2.57676300	-0.29536400	-1.31436400
C	-0.38581700	-0.66778900	-0.67619700
C	-1.01980100	-1.90028200	-0.77478000
C	-2.37922800	-1.69161000	-1.15060300
C	-0.40040100	-3.25817500	-0.64741600
H	0.07747900	-3.58187100	-1.58285000
H	-1.17015600	-3.99848000	-0.40262800
H	0.34561000	-3.29681500	0.15438800
O	1.77724300	2.54443200	-1.09740600
H	0.86914300	1.74302100	2.80328100
H	2.07457300	0.70225100	2.04160600
N	-1.38923900	0.38818100	-0.88399800
H	1.40497600	-1.72892900	-0.94625700
C	-3.38022200	-2.63110300	-1.45295100
H	-3.19553600	-3.69171100	-1.30325900
C	-4.58968200	-2.18644400	-1.97852600
H	-5.37900800	-2.88938600	-2.22528200
C	-4.75860200	-0.81386000	-2.24404400
H	-5.66699500	-0.46968500	-2.73002400
C	-3.77169700	0.12797700	-1.93393700
H	-3.94976600	1.13673500	-2.27166100
C	3.50886800	-0.11548600	-0.77058100
C	4.01538300	-1.40221200	-0.48424200
C	4.40713500	0.88370800	-1.20131600
C	5.37412400	-1.68848700	-0.64589100
H	3.34558100	-2.17619200	-0.11179100
C	5.76743200	0.59459800	-1.35792300
H	4.02228200	1.87272500	-1.43473000
C	6.25782500	-0.69074700	-1.08594200
H	5.74770800	-2.68292700	-0.41881600
H	6.44463100	1.37219200	-1.69885800
H	7.31382400	-0.91203400	-1.20916100
C	0.17795700	-0.24837400	2.40030700
C	0.78621100	-1.50728100	2.52760200
C	-1.20121200	-0.12942000	2.64467200
C	0.02956000	-2.63520300	2.87528700
H	1.85870800	-1.60030800	2.35726900
C	-1.96407200	-1.25692100	2.97294900
H	-1.67885900	0.84696900	2.57677300
C	-1.35098100	-2.51410500	3.08795400
H	0.51407800	-3.60204300	2.98265700
H	-3.03123500	-1.15537900	3.14651400
H	-1.94153700	-3.38635200	3.35287500

Spectroscopic characterization

Samples preparation

Samples were dissolved 1) in CH_2Cl_2 or 2) sodium phosphate buffer 10 mM, pH = 7.4 + 5% DMSO (Buffer B) or 3) sodium phosphate buffer 10 mM pH = 7.4, 1M $Mg(ClO_4)_2$ + 5% DMSO (buffer Z) or 4) potassium phosphate buffer 10 mM, pH = 7.1, 100 mM KCl + 5% DMSO (buffer Q).

The complexes between (*S*)-**2aj** or (*R*)-**2aj** and oligonucleotides were prepared as follows:
A solution of $(CG)_6$ (10μM) and (*S*)-**2aj** or (*R*)-**2aj** (10μM) in buffer B ((*S/R*)-**2aj** + B-DNA) was equilibrated 48 hours at 4°C.
A solution of $(CG)_6$ (10μM) and (*S*)-**2aj** or (*R*)-**2aj** (10μM) in buffer Z ((*S/R*)-**2aj** + Z-DNA) was equilibrated 48 hours at 4°C.
A solution of Pu_{18} (AGGGTGGGGAGGGTGGGG) (10μM) and (*S*)-**2aj** or (*R*)-**2aj** (10μM) in buffer Q ((*S/R*)-**2aj** + G_4-DNA) was equilibrated 48 hours at 4°C.

Samples for UV spectroscopy were analysed in CH_2Cl_2 at 30 μM concentration or in buffer A at different concentrations (2.5- 30 μM).
Fluorescence analyses were carried out at different concentrations of samples (2.5- 30 μM) in buffer A. Further analyses were carried out on samples at 10μM concentration in buffer B, or buffer Z or buffer Q. λ_{ex}= 310 or 345 nm; λ_{em}= 505 nm.
The fluorescence of complexes between (*S*)-**2aj** or (*R*)-**2aj** and oligonucleotides (prepared as described earlier) were also carried out. λ_{ex}= 310 or 345 nm; λ_{em}= 505 nm.
Quantum yields analyses were performed at 10 μM concentration in buffer B.
CD analyses were performed on samples of (*S*)-**2aj** or (*R*)-**2aj** at 10μM in buffers B, Z and Q and on samples of the complexes between (*S*)-**2aj** or (*R*)-**2aj** and oligonucleotides (prepared as described earlier).

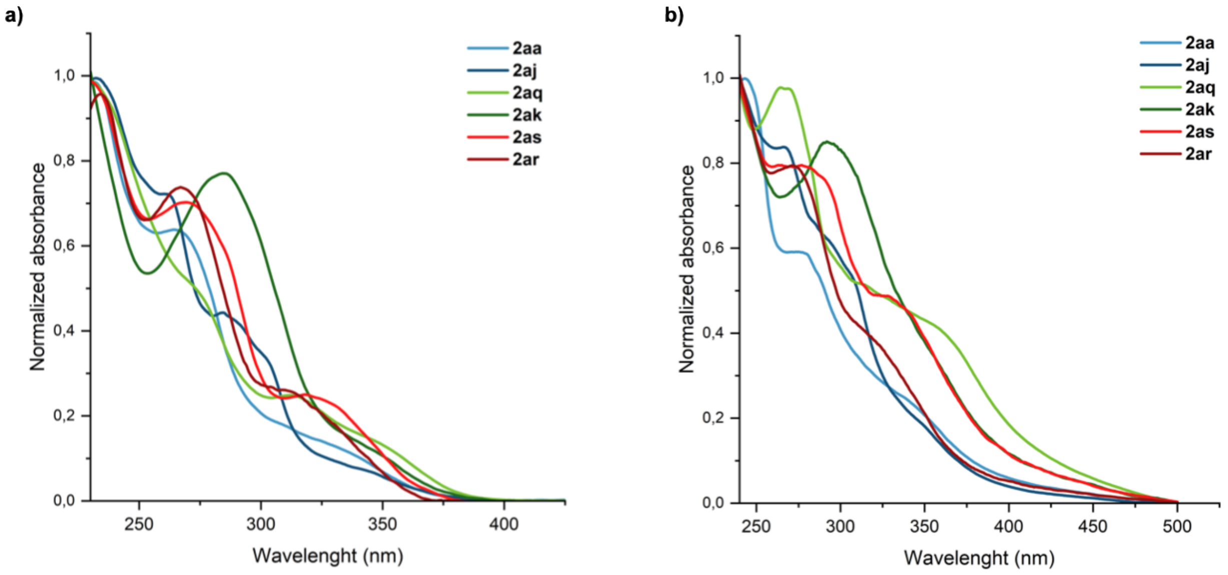

Figure S5: a) UV-absorption spectra in CH_2Cl_2 (30 μM). b) UV-absorption spectra (20 μM) in buffer B.

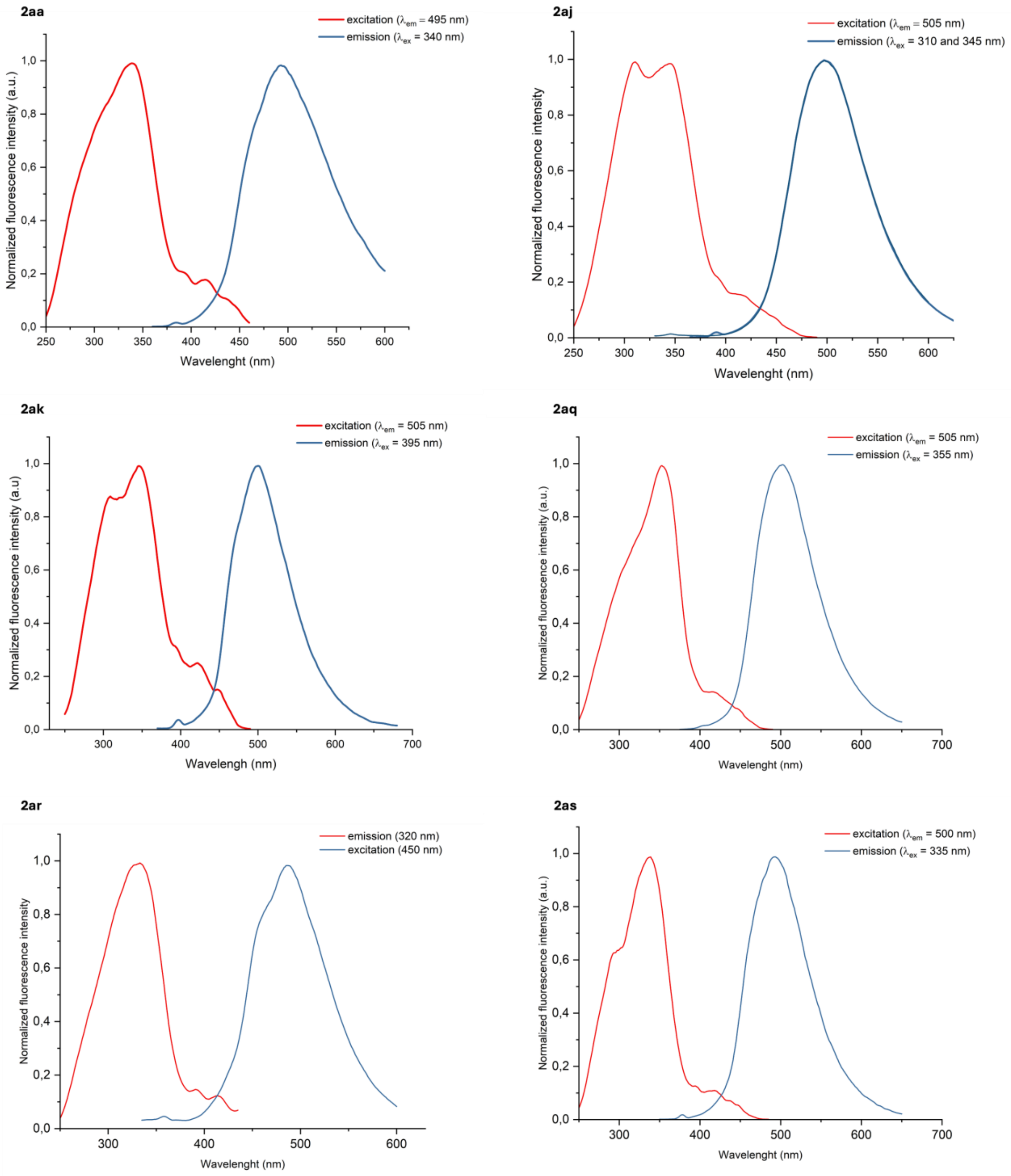

Figure S6: Fluorescence excitation and emission spectra (20 μM) in buffer B.

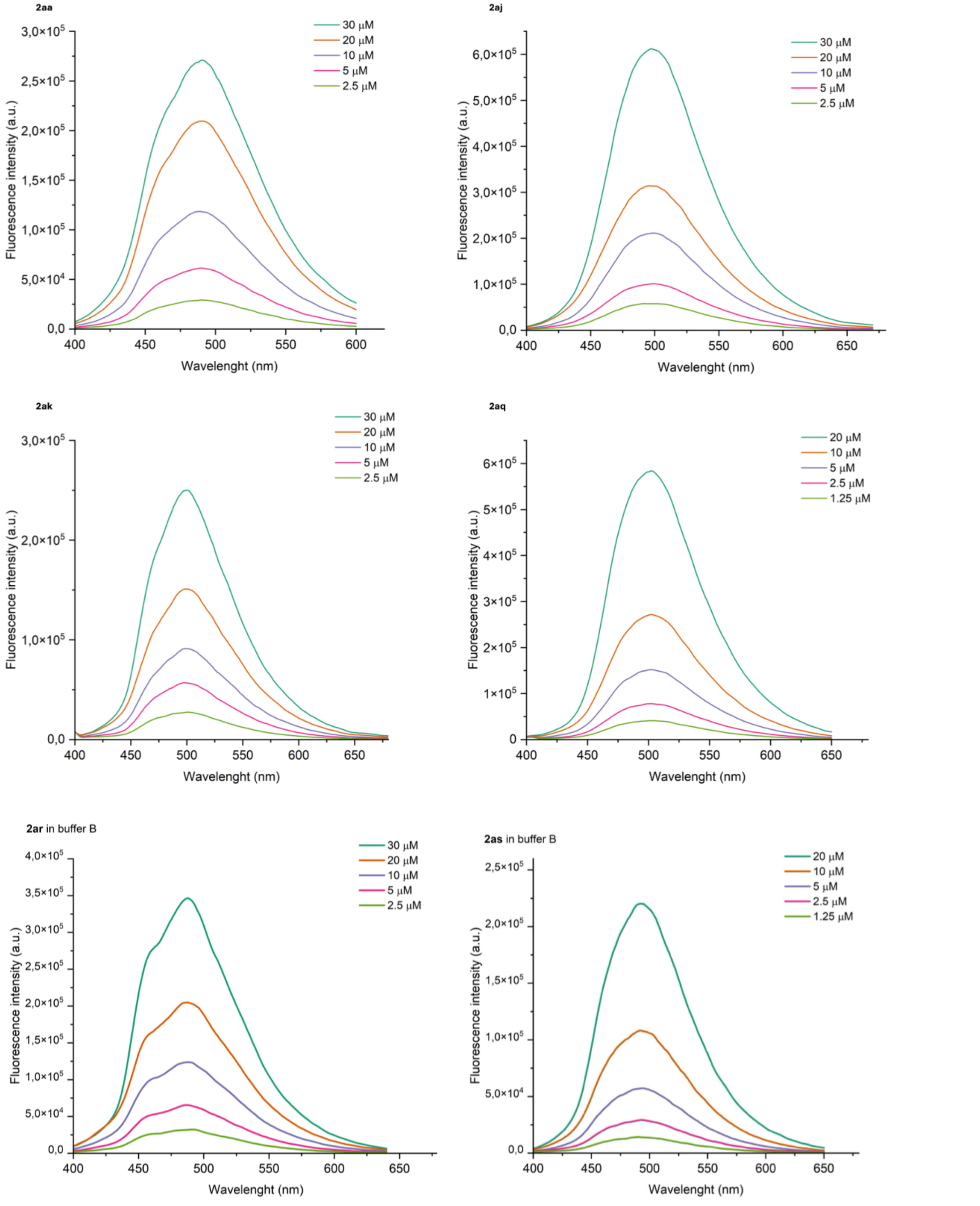

Figure S7: Fluorescence emission spectra of (*S*)-**2aa, aj, ak, aq, ar, as**, excited at their λ_{ex} (see figure S6), at different concentrations in buffer B.

Fluorescence spectra of (*R/S*)-2aj with DNA

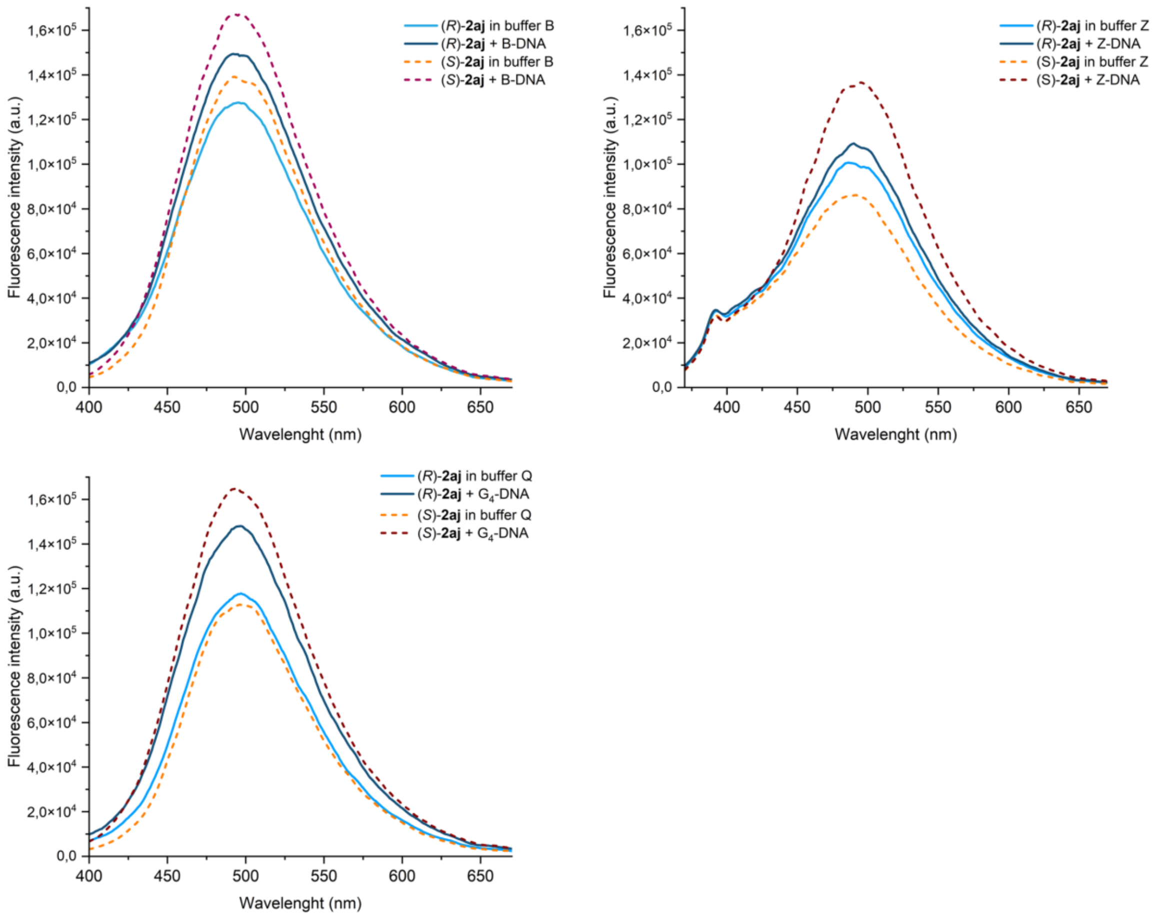

Figure S8: Fluorescence spectra of (*R/S*)-**2aj** complexes with DNA in the appropriate buffer with B-, Z- and G_4-DNA, λ_{ex} 345 nm.

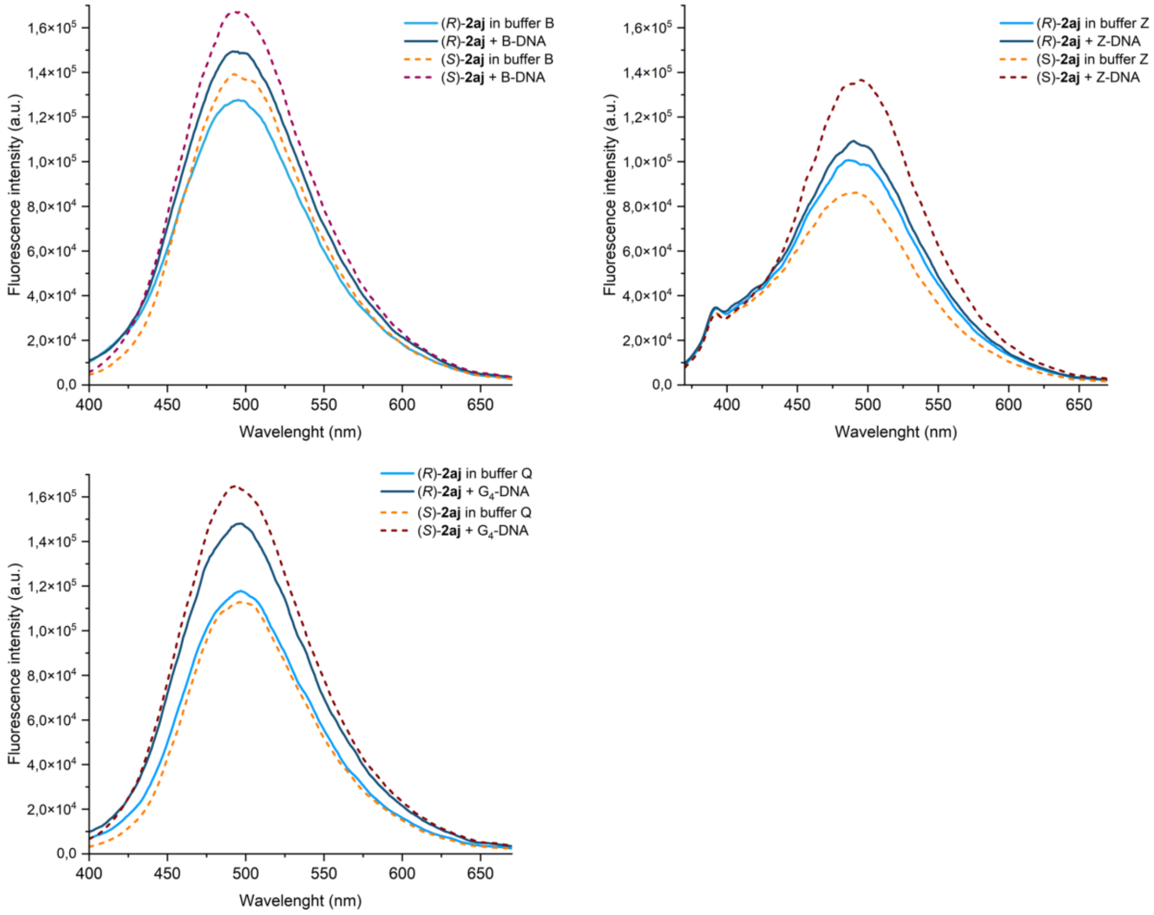

Figure S9: Fluorescence spectra of (*R/S*)-**2aj** complexes with DNA in the appropriate buffer with B-, Z- and G_4-DNA, λ_{ex} 310 nm.

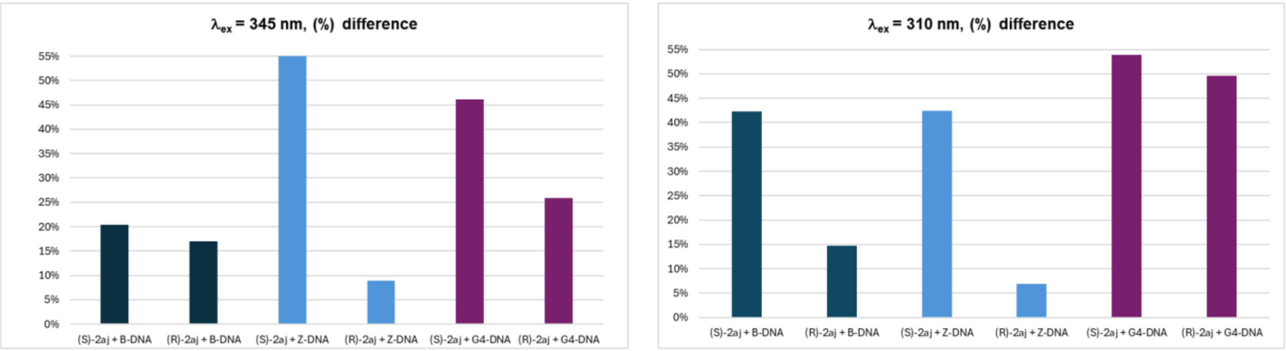

Figure S10: Fluorescence intensity percentage difference at λ_{ex} 345 and 310 nm. (%)difference= $(x_1-x_2)/x_1$ where x_1=fluorescence intensity of **2aj** in the appropriate buffer and x_2 = fluorescence intensity of the **2aj**/DNA complex.

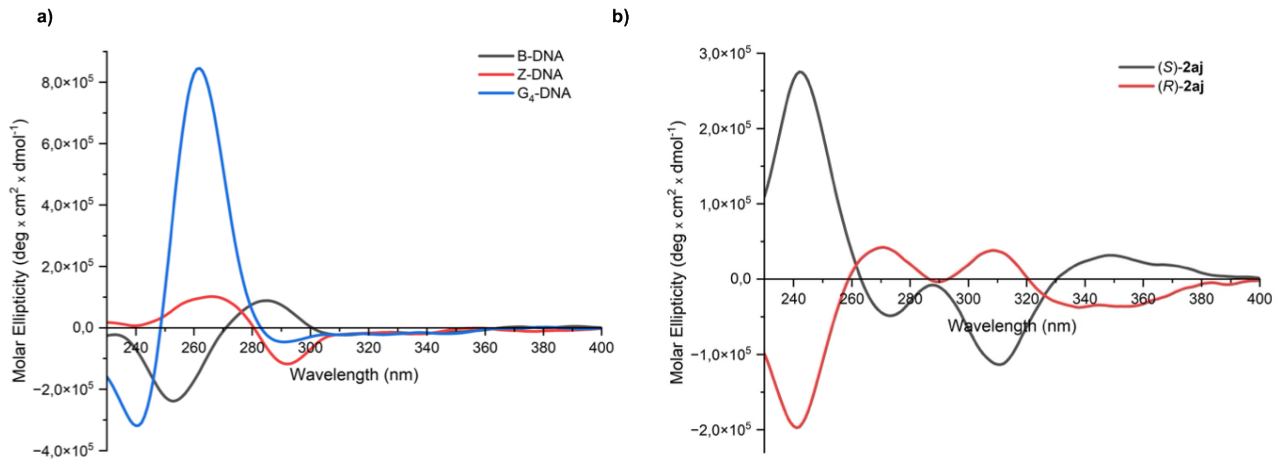

Figure S11: a) CD spectra B-, Z- and G_4-DNA in buffer B; b) CD spectra of (*S*)-**2aj** and (*R*)-**2aj** in buffer B.

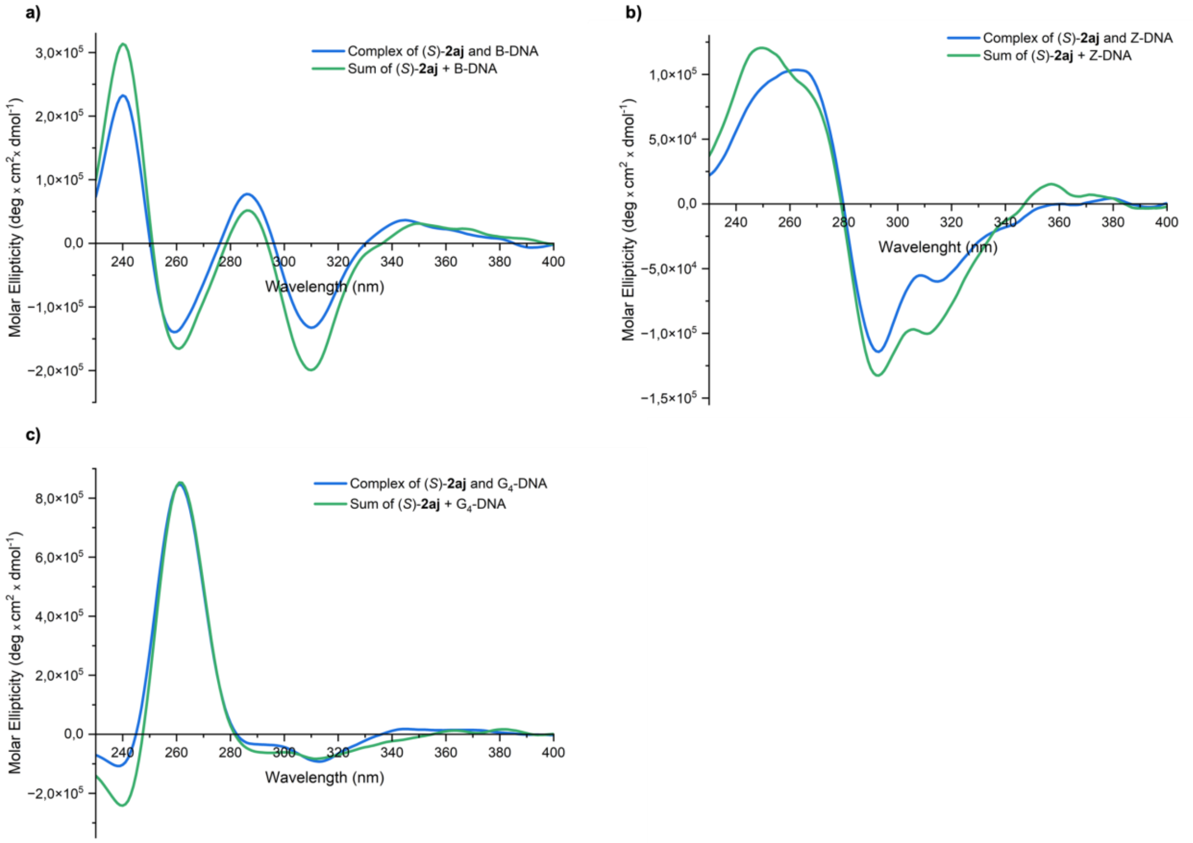

Figure S12: CD spectra showing the interactions of (*S*)-**2aj** with DNA. Comparison of the CD spectra of the complexes (blue lines) with CD spectra obtained after the sum of the (*S*)-**2aj** spectrum and the DNA spectrum (green lines).

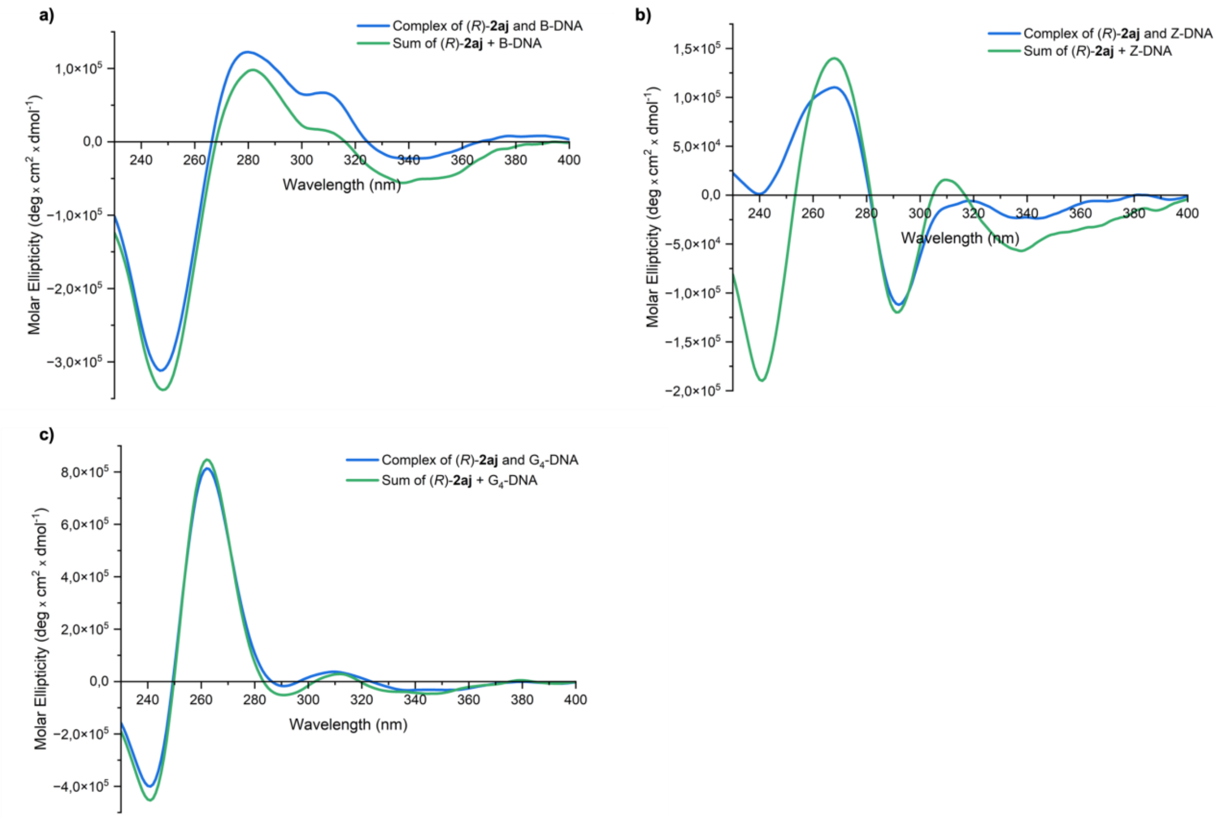

Figure S13: CD spectra showing the interactions of (*R*)-**2aj** with DNA. Comparison of the CD spectra of the complexes (blue lines) with CD spectra obtained after the sum of the (*R*)-**2aj** spectrum and the DNA spectrum (green lines).

X-ray crystallographic data

Crystallographic details of 2aa.

Crystals of **2aa** were obtained by slow evaporation from a deuterated DCM solution in an NMR tube. Single-crystal X-ray diffraction data for **2aa** were collected at room temperature on a Rigaku XtaLAB Synergy-S 4-circle diffractometer with a Hybrid Photon counting (HPC) detector by using a single graphite-monochromatized wavelength X-ray source (Cu Kα radiation: λ = 1.54184 Å) from a micro-focus sealed X-ray tube. Pre-experiment, data collection, data reduction and analytical absorption correction were performed with the program suite CrysAlisPro.[34] The structure was solved by direct methods by using SHELXT[35] and refined by full-matrix least squares refinements on F^2 (SHELX 2018)[36] with the WINGX interface.[37] The compound crystallizes in the orthorhombic, non-centrosymmetric chiral space group $P2_12_12_1$ (No. 19) (Figure S14), with two full molecules in the asymmetric unit (Z' = 2).

In one of the molecules, a benzene ring exhibits rotational disorder over two orientations with an occupancy ratio of 0.53:0.47. Disordered atoms of both components were refined isotropically. All other non-H atoms were refined anisotropically. Hydrogen atoms were placed in calculated positions (HFIX 43 for aromatic rings and vinyl groups, HFIX 23 for methyl groups) and were included in the refinement in the riding model approximation, with U_{iso} set to 1.2 U_{eq} (parent atom). The H atoms of the methyl groups were allowed to rotate with a fixed angle around the C—C bond to best fit the experimental electron density (HFIX 137) with U_{iso} set to1.5 U_{eq} (parent atom). The structure pictures were generated using the VESTA programs.[38]

Crystal data **2aa**: $C_{31}H_{24}N_2O$, M_r = 440.52. Orthorhombic, space group $P2_12_12_1$ (No. 19), a = 8.7601(1) Å, b = 15.8369(2) Å, c = 34.4002(4) Å, V = 4772.44(10)Å^3. Z = 8, D_c = 1.226 g·cm^{-3}, F(000) = 1856, T = 298.2(1) K, Cu $K\alpha$ radiation, λ = 1.54184 Å, μ = 0.579 mm^{-1}. Total number of reflections recorded to ϑ_{max} = 86.696°, was 66145 of which 10359 were unique (R_{int} = 0.0447); 9758 were 'observed' with $I > 2\sigma(I)$. Final R-values: wR_2 = 0.1365 and R_1 = 0.0469 for all data; wR_2 = 0.1322 and R_1 = 0.0492 for the 'observed' data. Final $GooF$ = 1.060, largest difference hole and peak = -0.271 and 0.270 e.Å^{-3}.

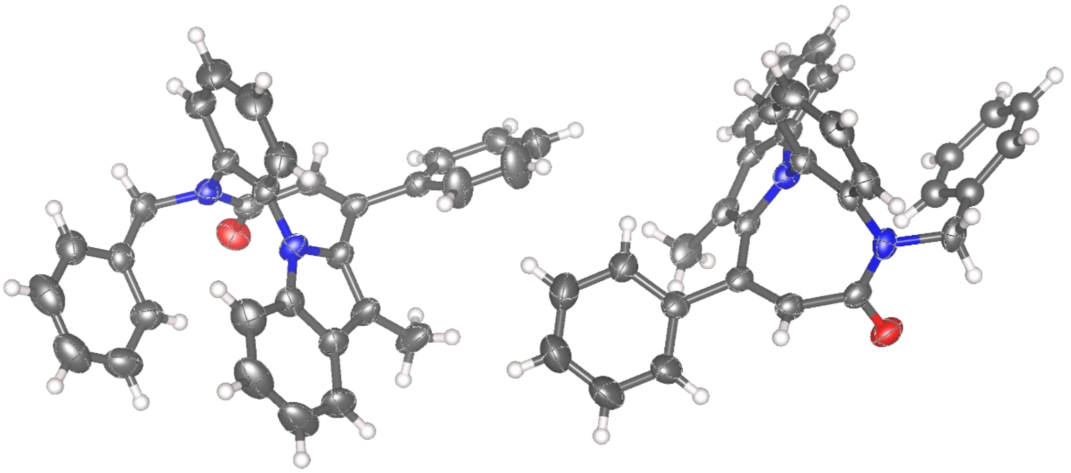

Figure S14: The two independent molecules present in the asymmetric unit of the crystal structure of **2aa** (disordered atoms have been removed). Thermal ellipsoids at RT were drawn at the 30 % probability level and H atoms are shown as small spheres of arbitrary radius. Atoms are represented with the usual colour code (C: black; N: blue; O: red; H: white). Labelling scheme has not been reported for clarity.

References

[1] a) I. Alonso, B. Trillo, F. Lopez, S. Montserrat, G. Ujaque, L. Castedo, A. Lledos and J. L. Mascareñas, *J. Am. Chem. Soc.*, 2009, **131**, 13020–13030; b)A. Z. Gonzalez and F. D. Toste, *Org. Lett.*, 2010, **12**, 1–3; c) J. Himmelstrup, M. Buendia, X. Sun and S. Kramer, *Chem. Commun.*, 2019, **55**, 12988–12991; d) J. Yu and C. Czekelius, *Eur. J. Org. Chem.*, 2022, **2022**, e202200027.

[2] M. Deimling, M. Kirchhof, B. Schwager, Y. Qawasmi, A. Savin, T. Mühlhäuser, W. Frey, T. Sottmann, B. Claasen, A. Baro and S. Laschat, *Chem. Eur. J.*, 2019, **25**, 9464–9476.

[3] J. Meesin, P. Katrun, C. Pareseecharoen, M. Pohmakotr, V. Reutrakul, D. Soorukram, C. Kuhakarn, *J. Org. Chem.*, 2016, **81**, 2744−2752.

[4] J. Ahn, S. B. Lee, I. Song, S. Chun, D. C. Oh, S. Hong, *J. Org. Chem.,* 2021, **86**, 7390.

[5] T. Tomakinian, R. Guillot, C. Kouklovsky, G. Vincent, *Angew. Chem. Int. Ed.,* 2014, **53**, 11881 – 11885.

[6] L. Yu, S. Huang, T. Cai, K. Du, C. Wu, H. Dong, R. Shen, *J. Org. Chem.,* 2022, **87**, 15114 – 15119.

[7] L. Joucla, N. Batail, L. Djakovitch, *Adv. Synth. Catal***.,** 2010, **352**, 2929 – 2936.

[8] C. Rapelli, B. Sridhar, R. Subba, *Org. Biomol. Chem.,* 2020, **18**, 6710 – 6715.

[9] J. Chen, J. Wu, *Angew. Chem. Int. Ed.* 2017, **56**, 3951 – 3955.

[10] T. Bzeih, D. Lamaa, G. Frison, A. Hachem, N. Jaber, J. Bignon, P. Retailleau, M. Alami, A. Hamze, *Org. Lett.,* 2017, **19**, 6700 – 6703.

[11] X. Gao, R. Chang, J. Rao, D. Hao, Z. Zhang, C. Zhou, Z. Guo, *J. Org. Chem.,* 2022, **87**, 8198 – 8202.

[12] E. Brambilla, S. Meraviglia, E. Moneta, D. Nava, S. Rizzato, G. Abbiati, V. Pirovano, *Adv. Synth. Catal*., 2023, **365**, 3958–3966.

[13] Y. Fan, Y. Jiang, D. An, D. Sha, J. Antilla, S. Zhang, *Org. Lett.,* 2014, **16**, 6112−6115.

[14] A. Chandrasekhar, S. Sankararaman, *Org. Biomol. Chem.,* 2020, **18**, 1612 – 1622.

[15] S. Gorle, S. Chandra, R. Vaikunta, R. Raghavendra, R. Akula, *Synth. Commun***.,** 2023, **53**, 414 – 425.

[16] S. Badigenchala, V. Rajeshkumar, G. Sekar, *Org. Biomol. Chem*., 2016, **14**, 2297 – 2305.

[17] V. Weingand, T. Wurm, V. Vethacke, M. Dietl, D. Ehjeij, M. Rudolph, F. Rominger, J. Xie, S. Hashmi*, Chem. Eur. J.,* 2018, **24**, 3725 – 3728.

[18] A. Kim, C. Lee, J. Song, S. Lee, Y. Kwon, *Nat. Commun*., 2023, **14**, 5502.

[19] M. J. Frisch *et al.*, *Gaussian 16*, Revision A.03, Gaussian, Inc., Wallingford CT, 2016.

[20] C. G. Newton, E. Braconi, J. Kuziola, M. D. Wodrich, N. Cramer, *Angew. Chem. Int*. *Ed*., 2018, **57**, 11040 –11044.

[21] a) Y. Zhao, D. G. Truhlar, *Acc. Chem. Res.,* 2008, **41**, 157-167; b) Y. Zhao, D. G. Truhlar, *Theor. Chem. Acc*., 2008, **120**, 215-241.

[22] A. V. Marenich, C. J. Cramer, D. G. Truhlar, *J. Phys. Chem. B,* 2009, **113**, 6378-6396.

[23] C. Adamo, V. Barone*,* J. *Chem. Phys*., 1999, **110**, 6158-6170.

[24] S. Grimme, S. Ehrlich, L. Goerigk, *J. Comp. Chem.,* 2011, **32**, 1456–1465.

[25] a) W. R. Wadt, P. J. Hay, *J. Chem. Phys.,* 1985, **82**, 270-283; b) W. R. Wadt, P. J. Hay, *J. Chem. Phys*., 1985, **82**, 284-298; c) W. R. Wadt, P. J. Hay, *J. Chem. Phys*., 1895, **82**, 299.

[26] M. Cossi, N. Rega, G. Scalmani, V. Barone, *J. Comp. Chem.,* 2003, **24**, 669-681.

[27] Molecular Operating Environment (MOE), 2024.0601 Chemical Computing Group ULC, 910-1010 Sherbrooke St. W., Montreal, QC H3A 2R7, 2025.

[28] a) R. F. W. Bader, *Atoms in Molecules: A Quantum Theory*, Oxford University Press, Oxford, 1990; b) R. F. W. Bader, M. T. Carroll, J. R. Cheeseman, C. Chang, *J. Am. Chem. Soc.,* 1987, **109**, 7968–7979; c) R. F. W. Bader, T. S. Slee, D. Cremer, E. Kraka, *J. Am. Chem. Soc.,* 1983, **105**, 5061–5068.

[29] a) T. Lu, F. Chen, *J. Comput. Chem.,* 2012, **33**, 580-592; b) T. Lu, *J. Chem. Phys.,* 2024, **161**, 082503.

[30] A. S. Hazrah, S. Nanayakkara, N. A. Seifert, E. Kraka and W. Jäger, *Phys. Chem. Chem. Phys.,* 2022, **24**, 3722.

[31] J. P. M. Lommerse, A. J. Stone, R. Taylor and F. H. Allen, *J. Am. Chem. Soc.,* 1996, **118**, 3108.

[32] D. Dey, S. Bhandary, A. Sirohiwal, V. R. Hathwar and D. Chopra, *Chem. Commun*., 2016, **52**, 7225.

[33] A. M. Hua, S. L. Bidwell, S. I. Baker, H. P. Hratchian and R. D. Baxter, *ACS Catal.,* 2019, **9**, 3322.

[34] Rigaku Oxford Diffraction, *CrysAlisPro*, version 171.42.91a, Rigaku Corporation, Oxford, UK, 2021.

[35] G. M. Sheldrick, *Acta Crystallogr., Sect. A: Found. Adv.,* 2015, **71**, 3–8.

[36] G. M. Sheldrick, *Acta Crystallogr. C. Struct. Chem.,* 2015, **71**, 3–8.

[37] L. J. Farrugia, *J. Appl. Crystallogr.,* 2012, **45**, 849–854.

[38] K. Momma, F. Izumi, *J. Appl. Cryst.,* 2011, **44**, 1272–1276.

NMR spectra of new compounds

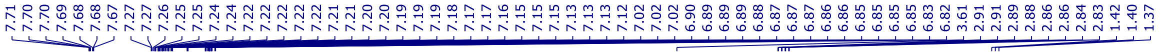

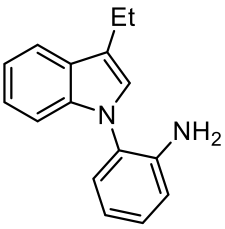

S-1b, ^{1}H-NMR ($CDCl_3$, 300 MHz)

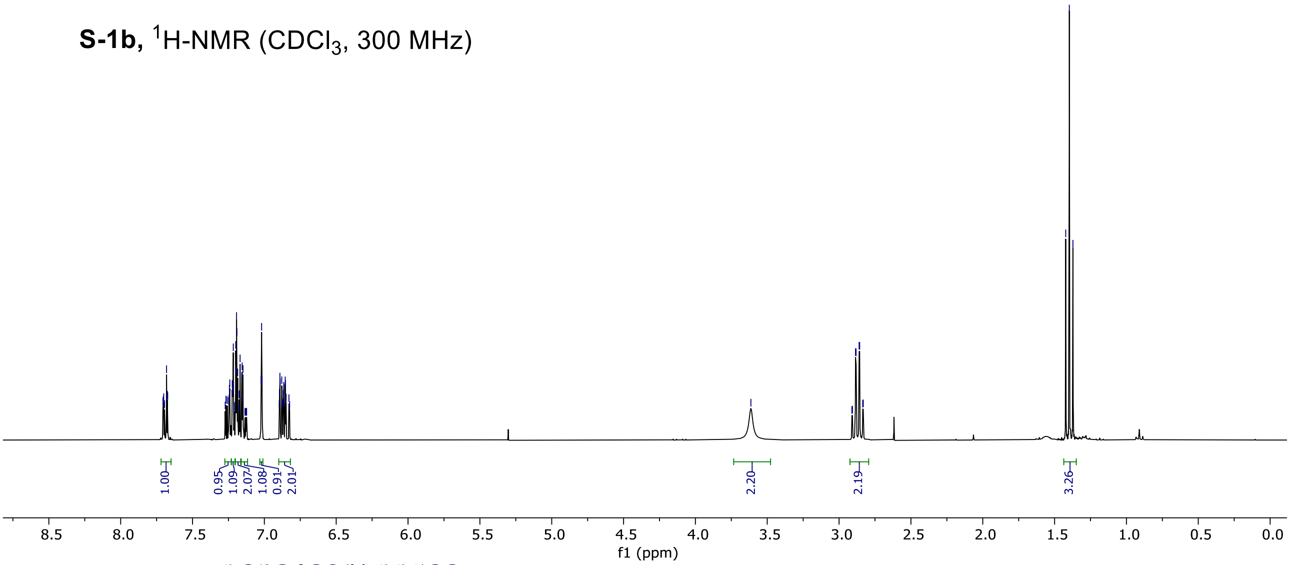

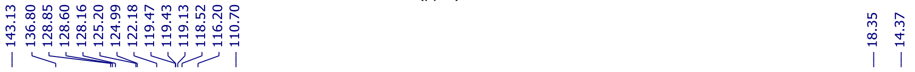

S-1b, ^{13}C-NMR ($CDCl_3$, 75 MHz)

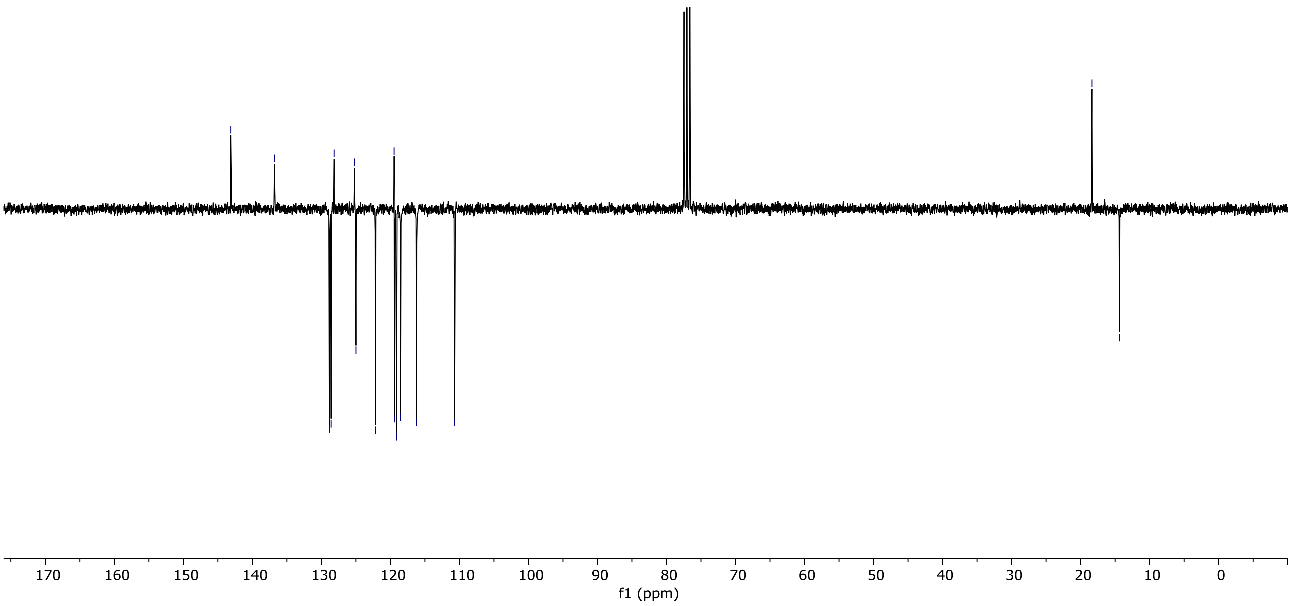

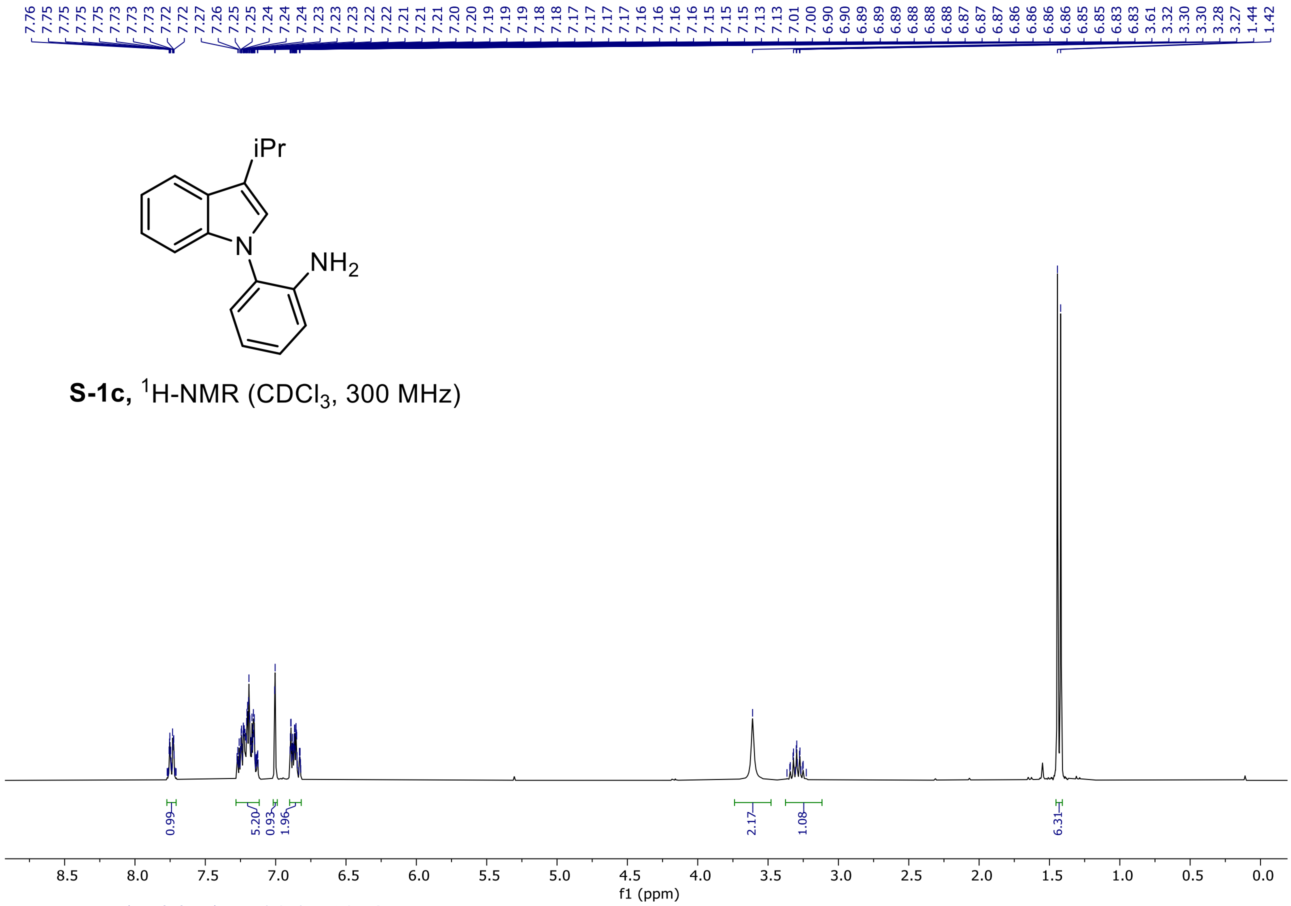

S-1c, ¹H-NMR ($CDCl_3$, 300 MHz)

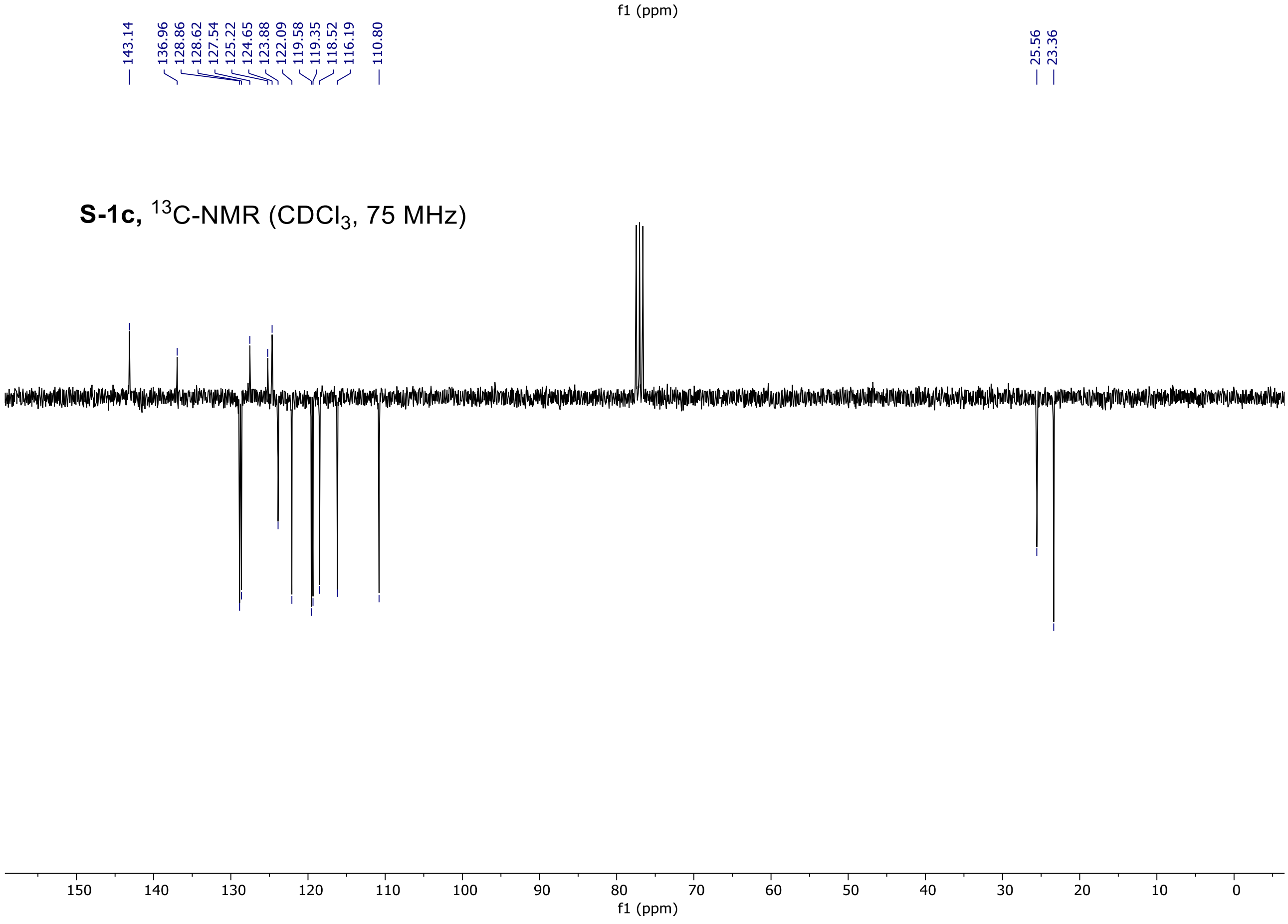

S-1c, ¹³C-NMR ($CDCl_3$, 75 MHz)

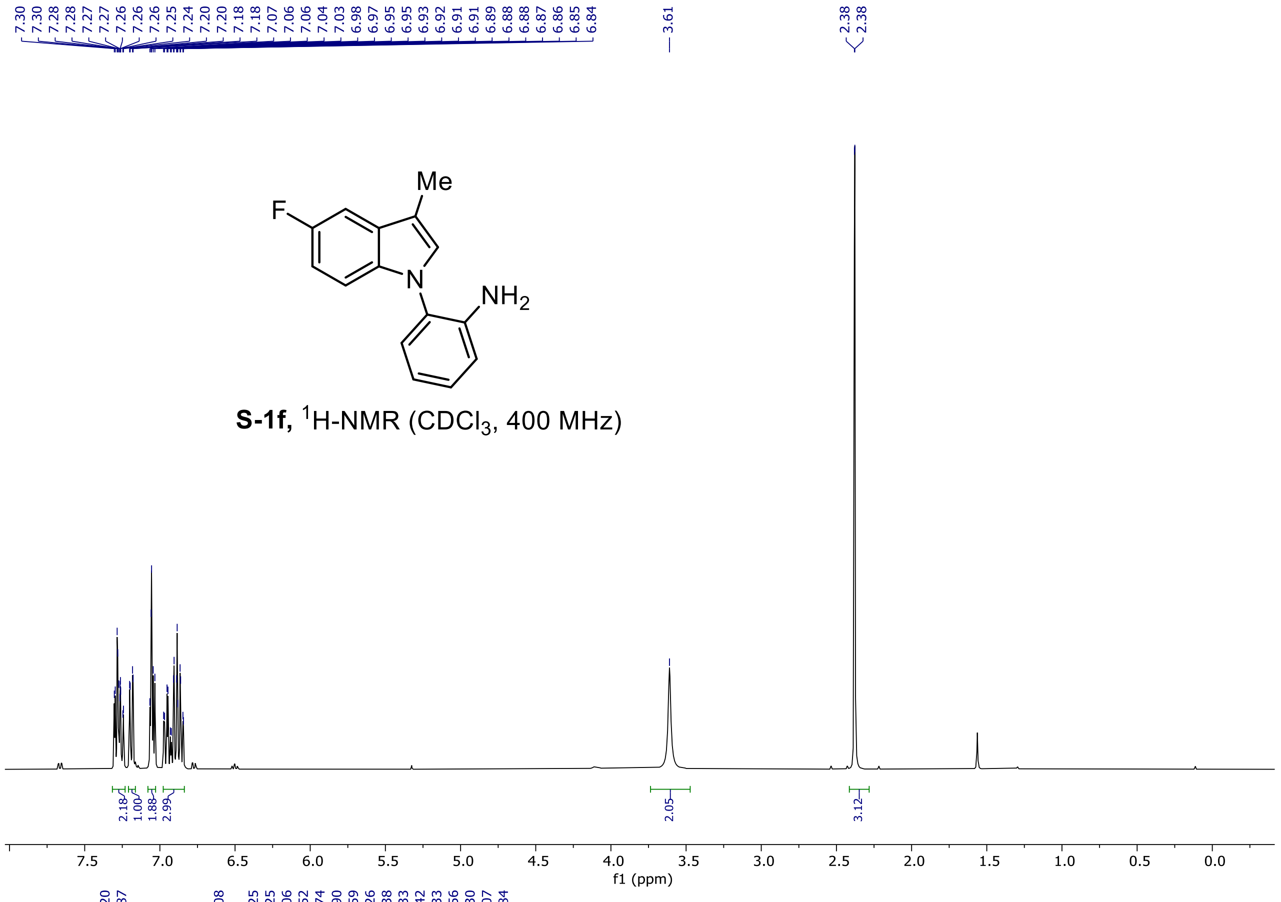

S-1f, ^{1}H-NMR ($CDCl_3$, 400 MHz)

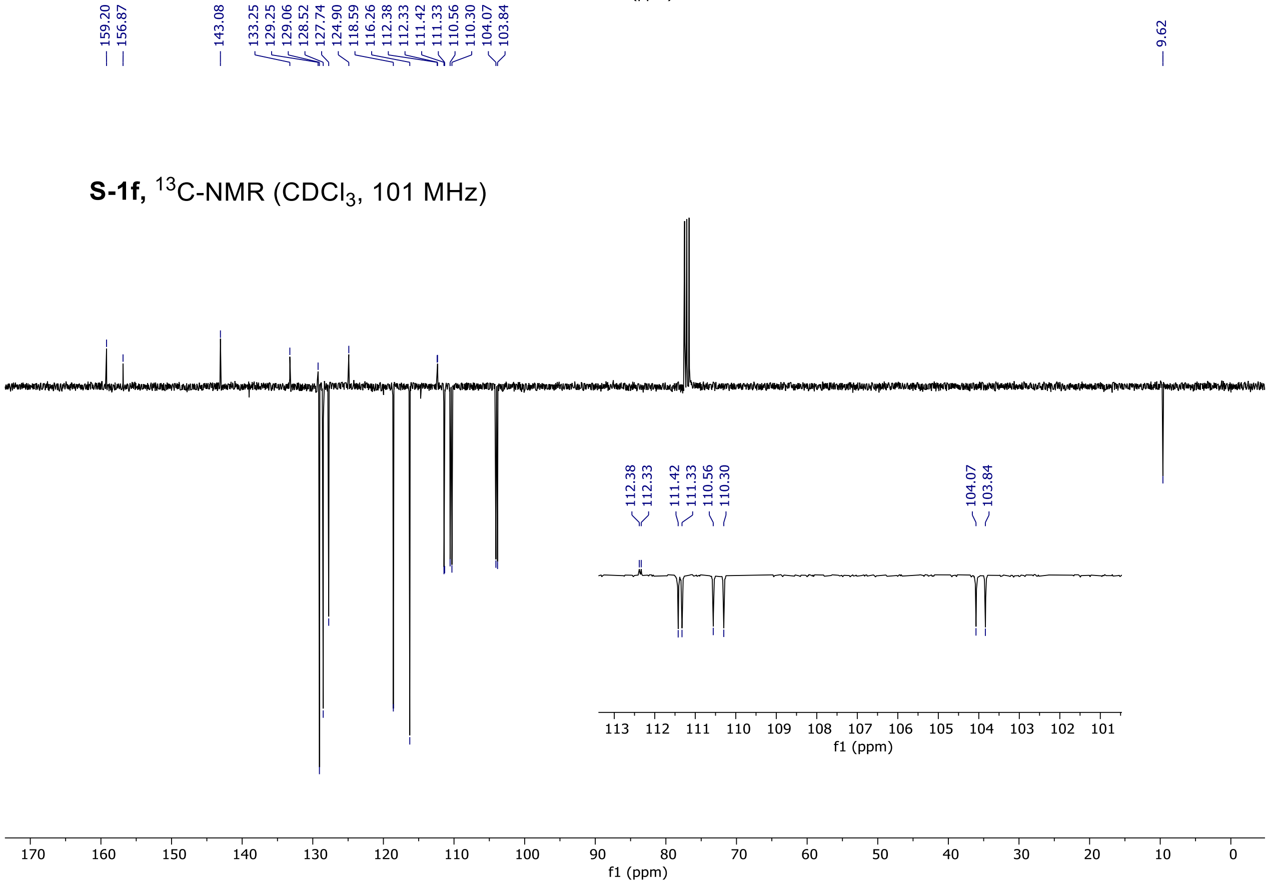

S-1f, ^{13}C-NMR ($CDCl_3$, 101 MHz)

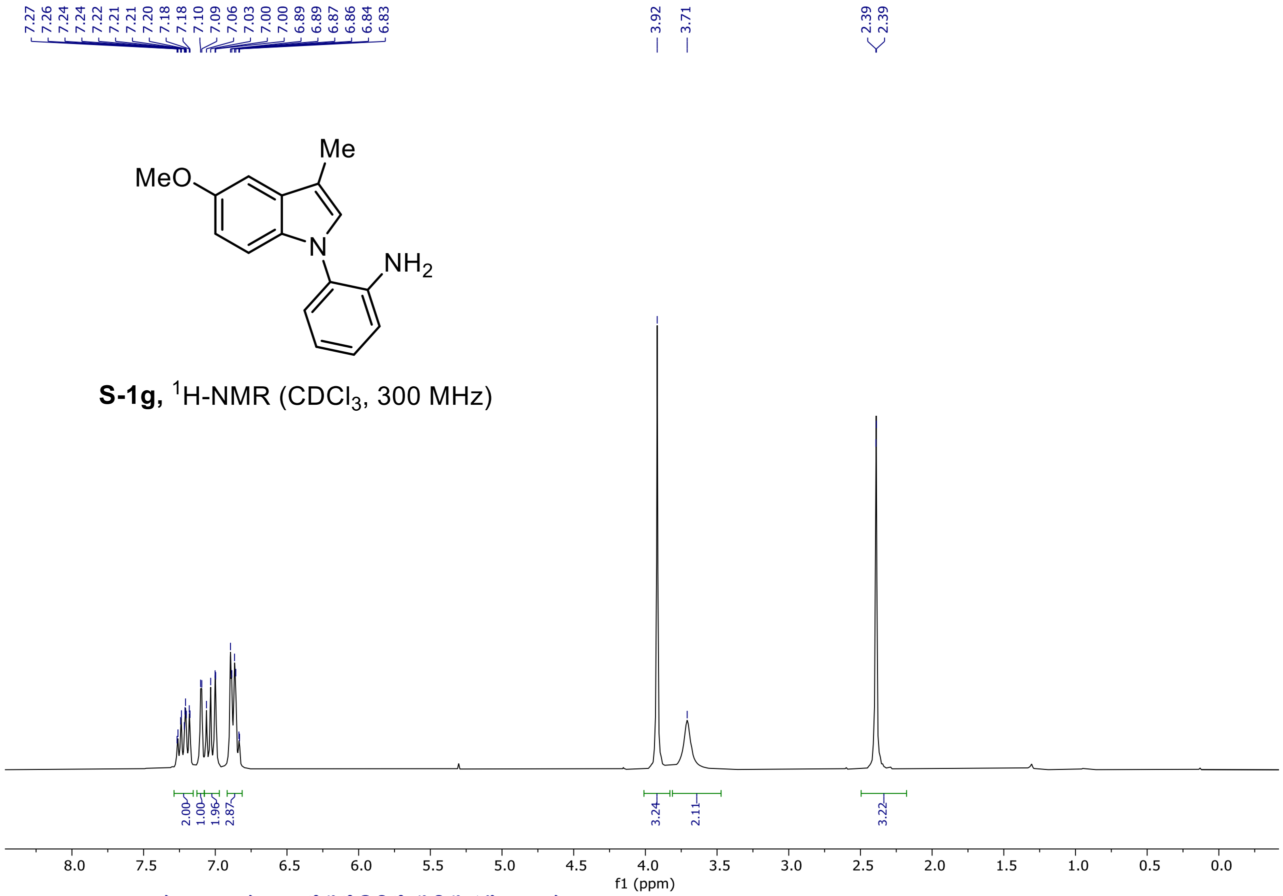

S-1g, ^{1}H-NMR ($CDCl_3$, 300 MHz)

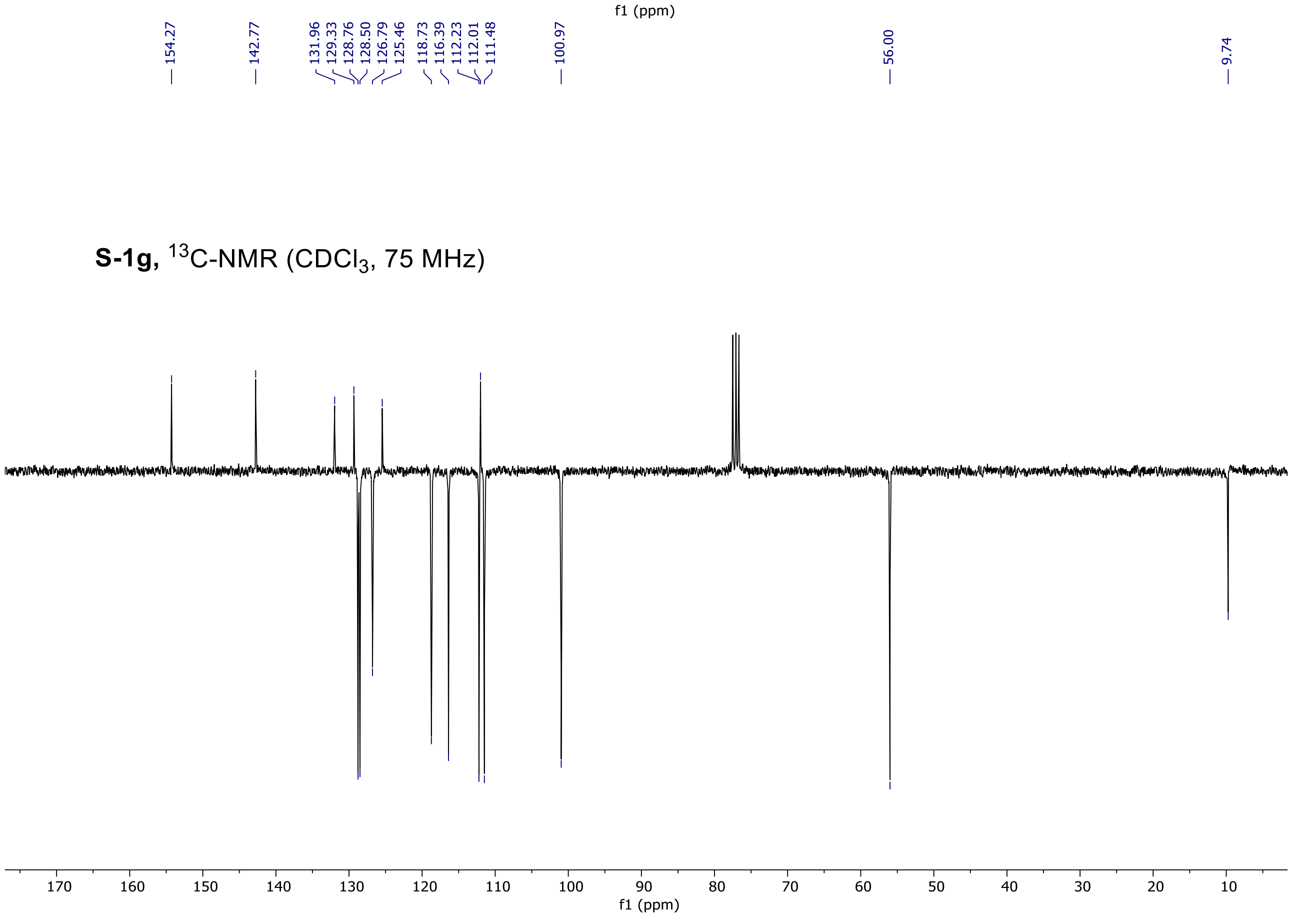

S-1g, ^{13}C-NMR ($CDCl_3$, 75 MHz)

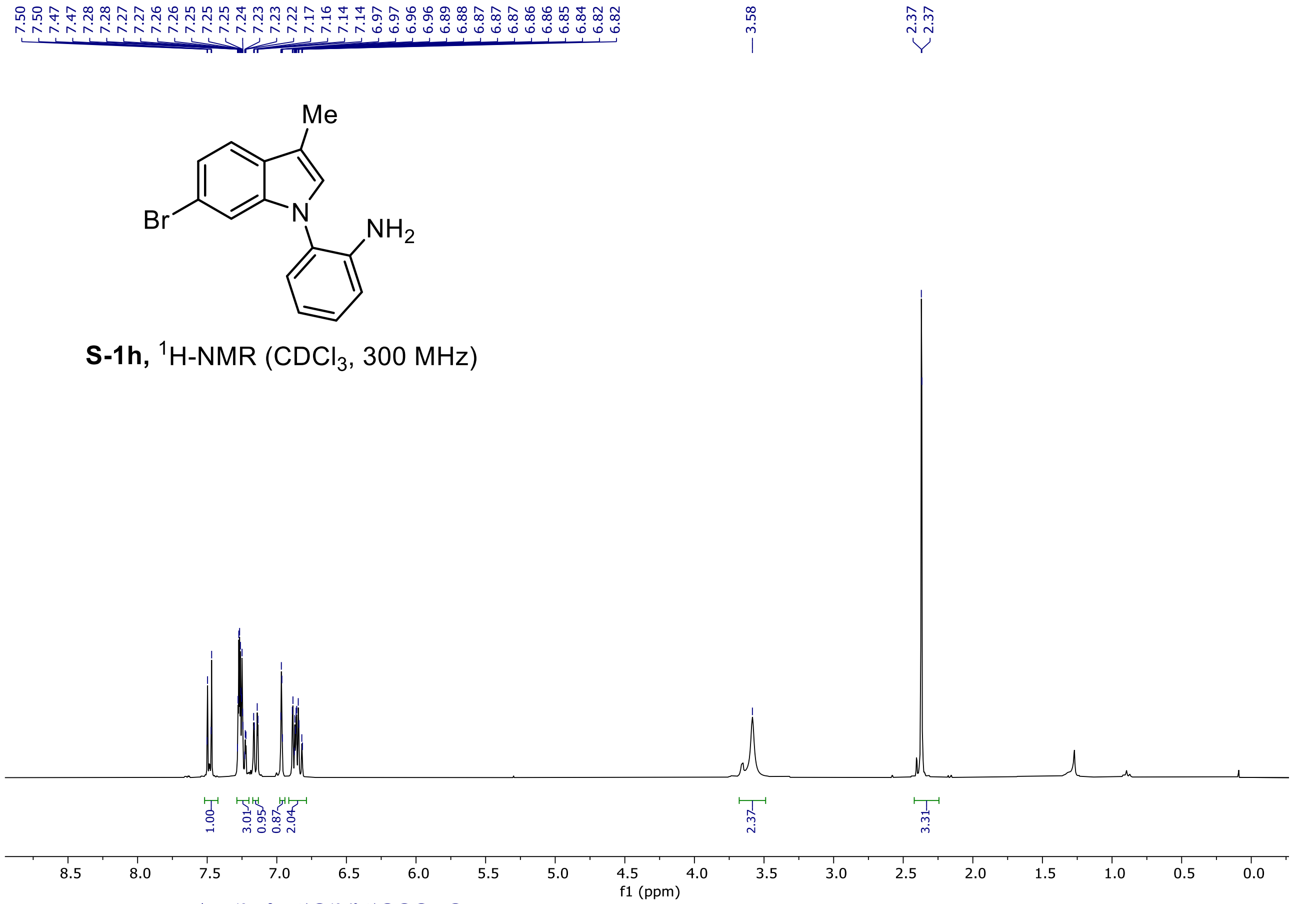

S-1h, ^{1}H-NMR ($CDCl_3$, 300 MHz)

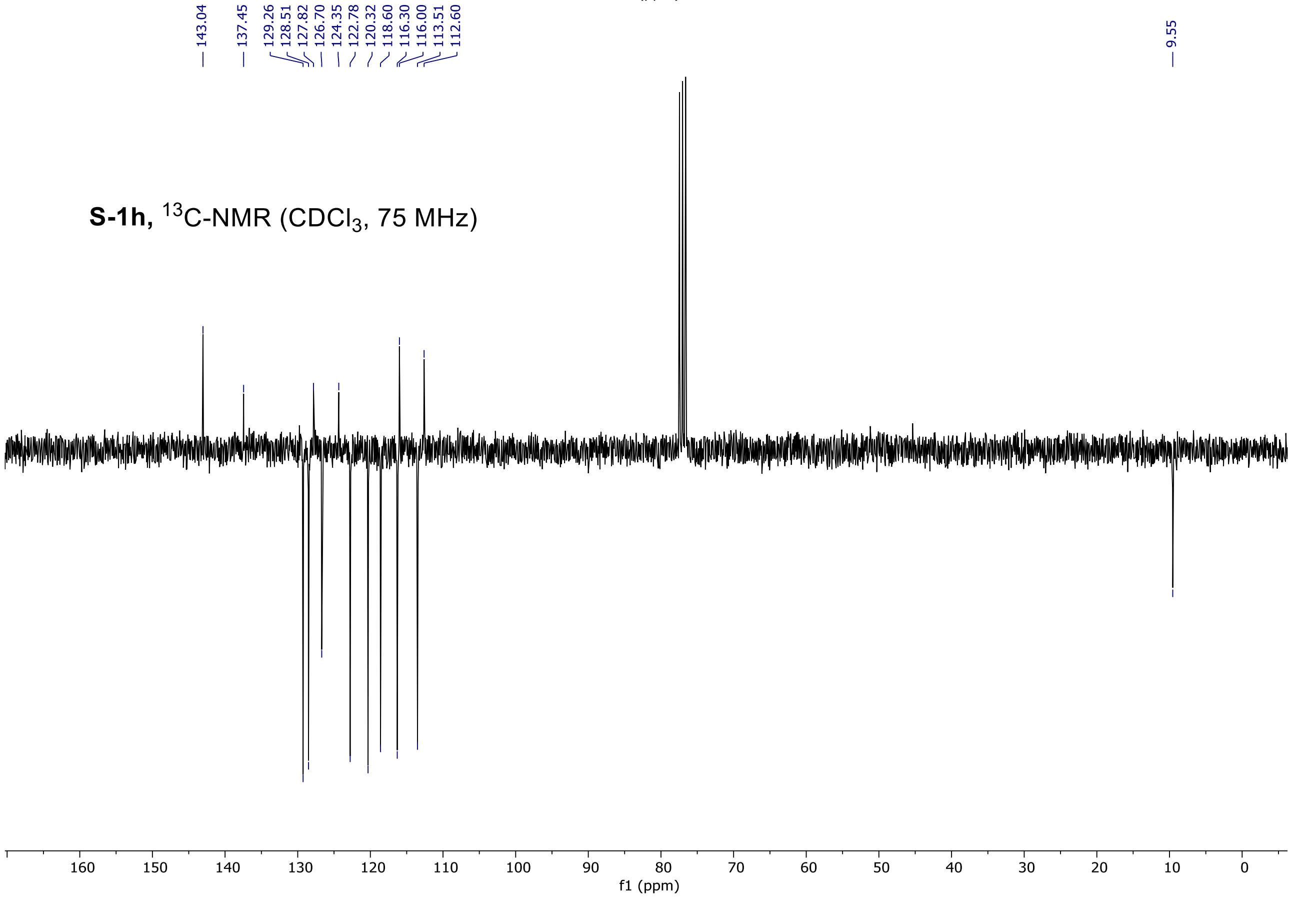

S-1h, ^{13}C-NMR ($CDCl_3$, 75 MHz)

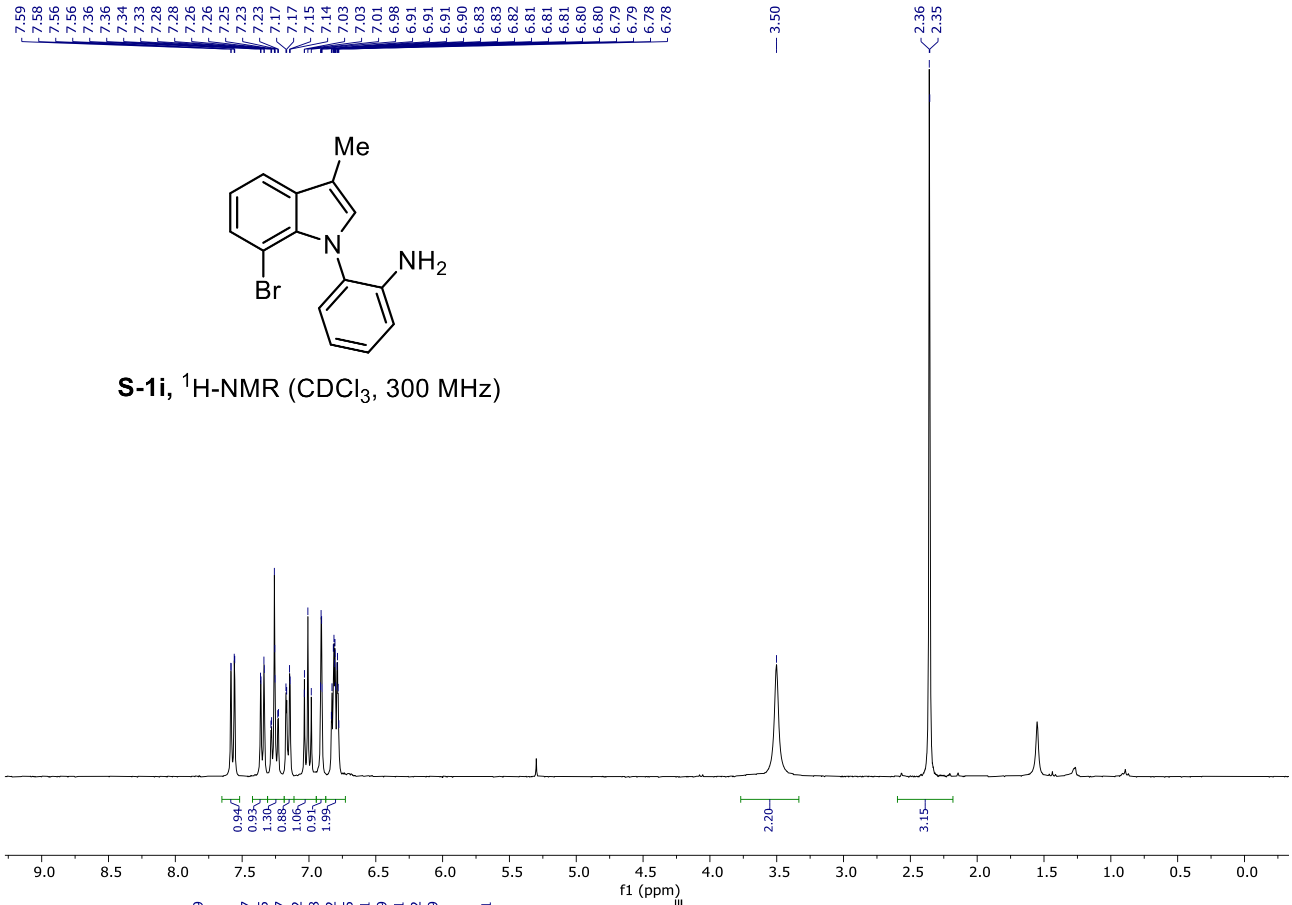

S-1i, ^{1}H-NMR ($CDCl_3$, 300 MHz)

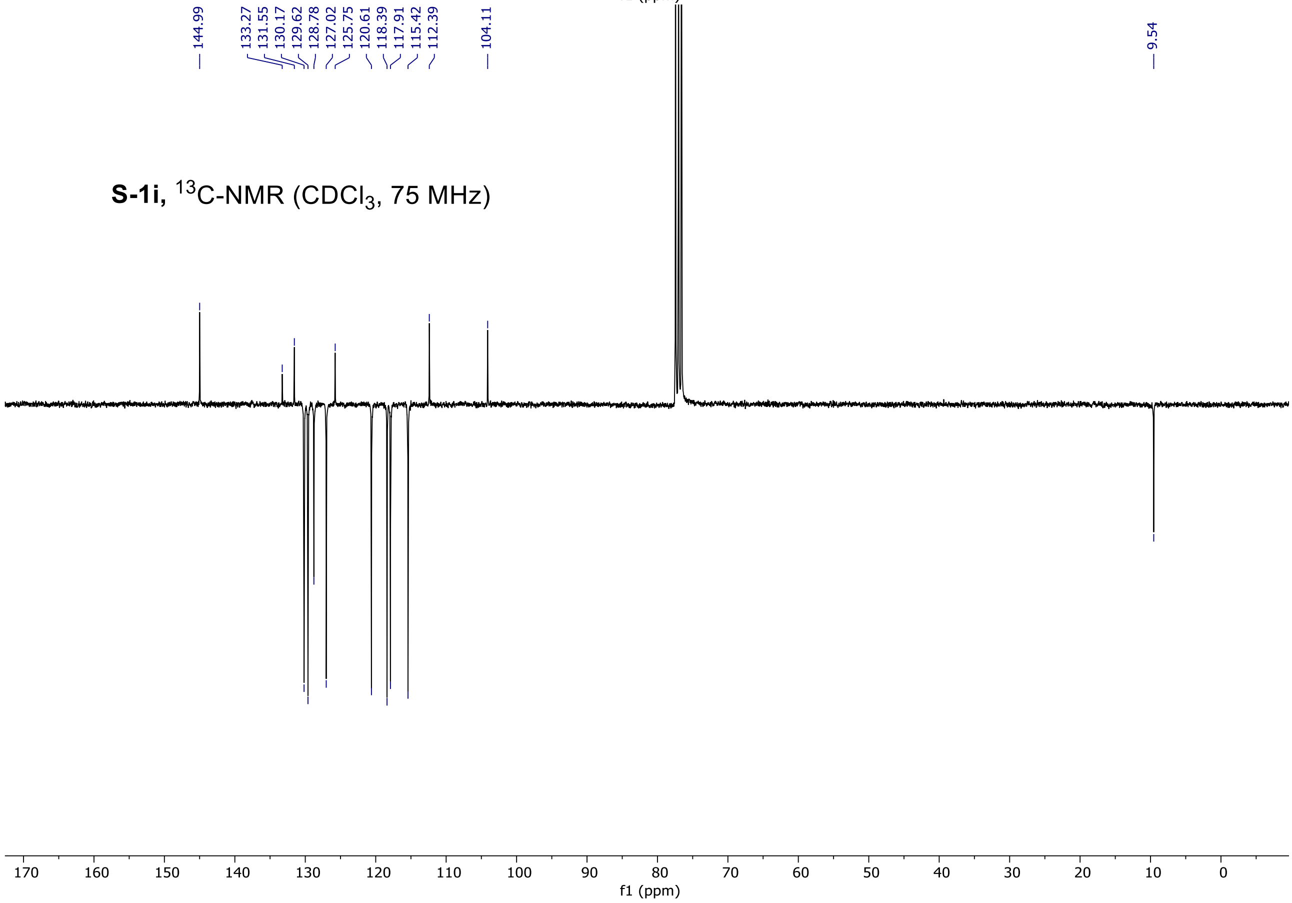

S-1i, ^{13}C-NMR ($CDCl_3$, 75 MHz)

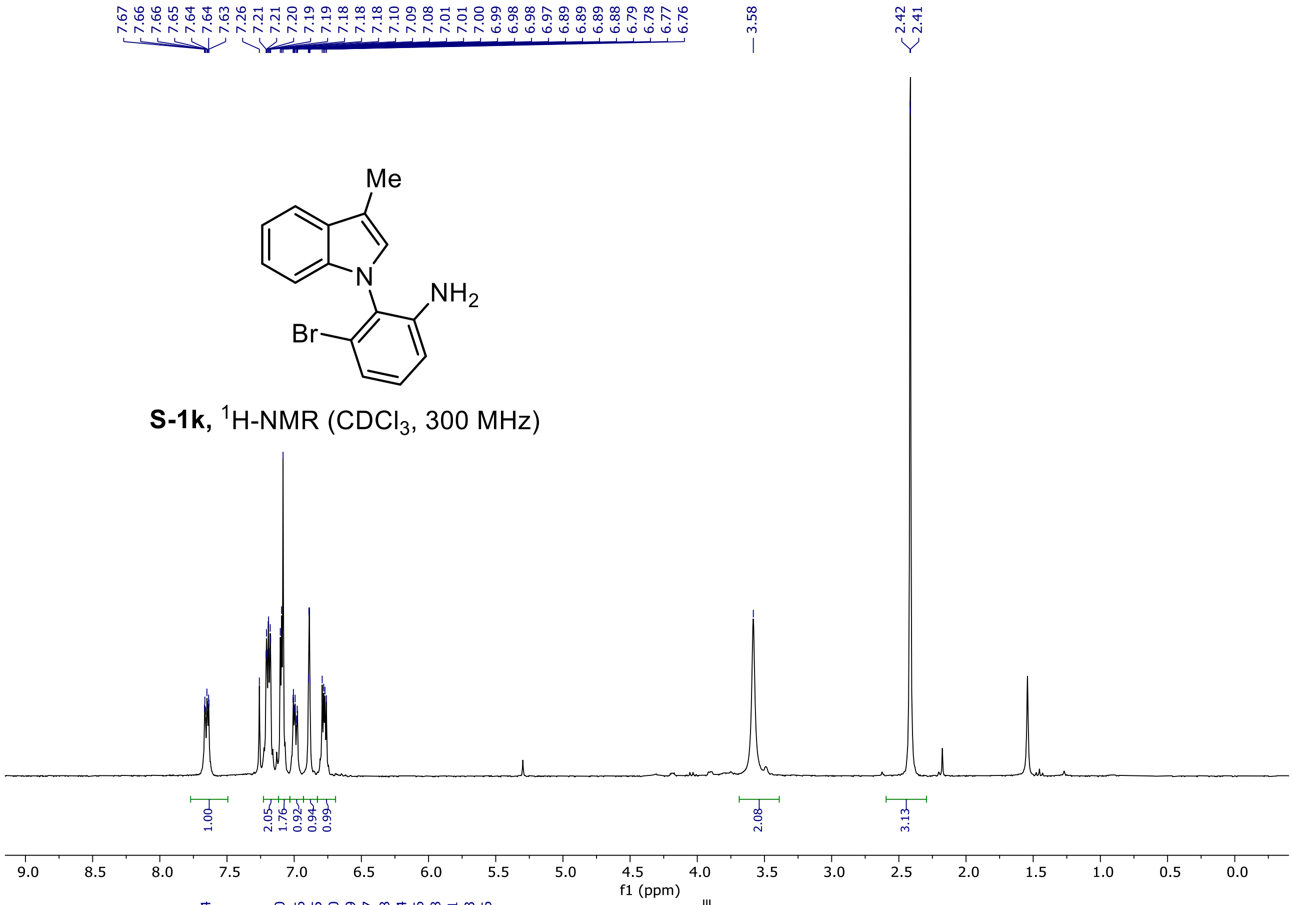
7.67 7.66 7.65 7.65 7.64 7.64 7.63 7.26 7.21 7.21 7.20 7.19 7.19 7.18 7.18 7.18 7.10 7.09 7.08 7.01 7.01 7.00 6.99 6.98 6.98 6.97 6.89 6.89 6.89 6.88 6.79 6.78 6.77 6.76
3.58
2.42 2.41
Me
N
NH2
Br
S-1k, 1H-NMR (CDCl3, 300 MHz)
1.00
2.05 1.76 0.92 0.94 0.99
2.08
3.13
9.0 8.5 8.0 7.5 7.0 6.5 6.0 5.5 5.0 4.5 4.0 3.5 3.0 2.5 2.0 1.5 1.0 0.5 0.0
f1 (ppm)

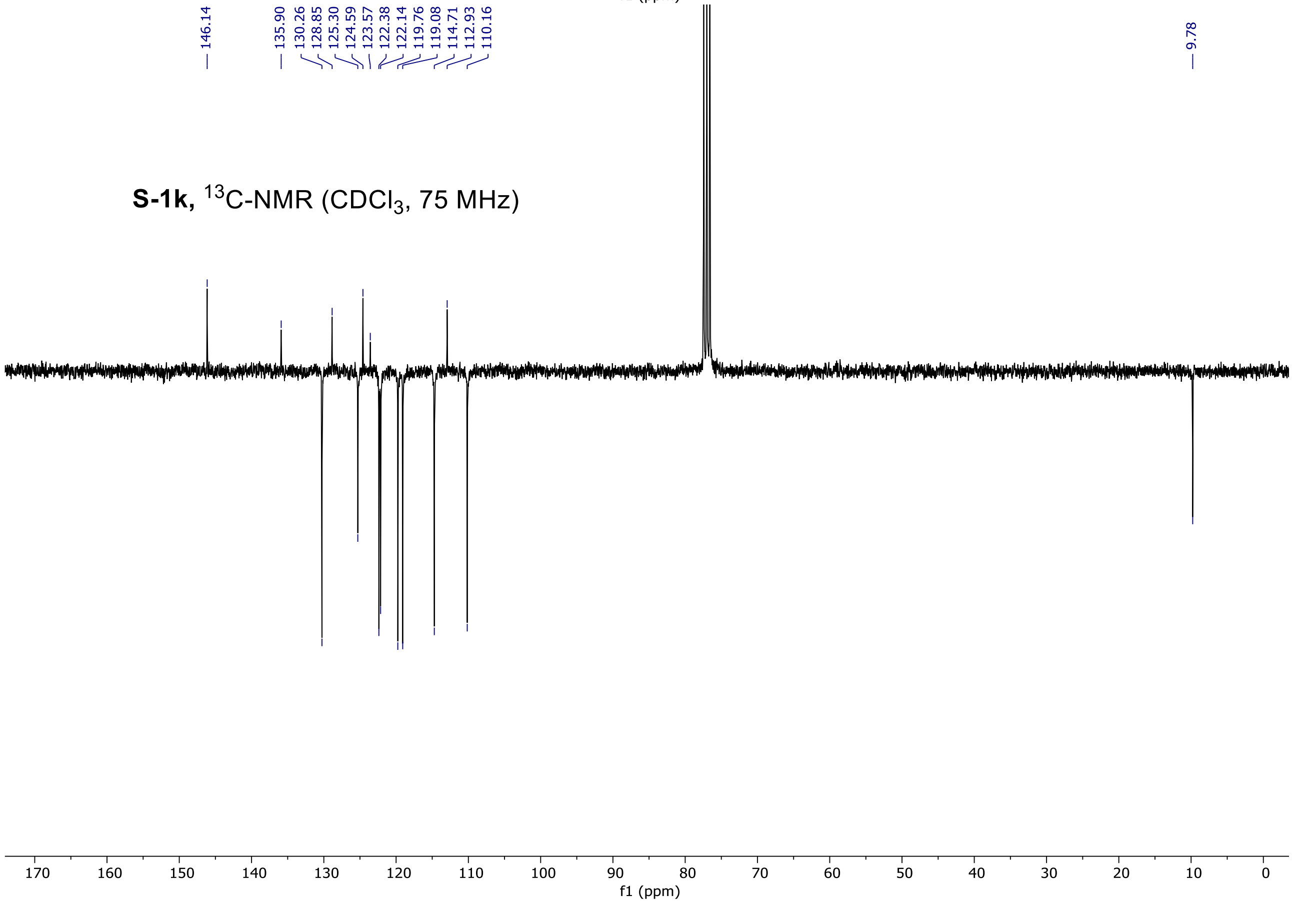
146.14
135.90 130.26 128.85 125.30 124.59 123.57 122.38 122.14 119.76 119.08 114.71 112.93 110.16
9.78
S-1k, 13C-NMR (CDCl3, 75 MHz)
170 160 150 140 130 120 110 100 90 80 70 60 50 40 30 20 10 0
f1 (ppm)

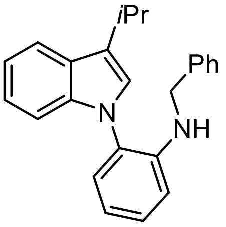

S-2c, ^{1}H-NMR ($CDCl_3$, 400 MHz)

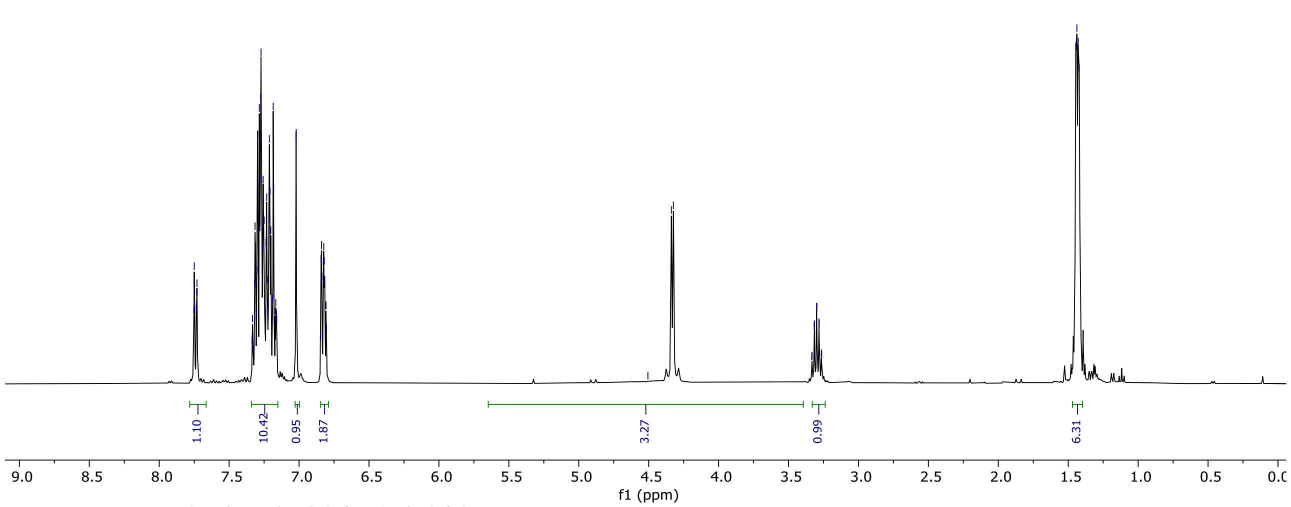

S-2c, ^{13}C-NMR ($CDCl_3$, 101 MHz)

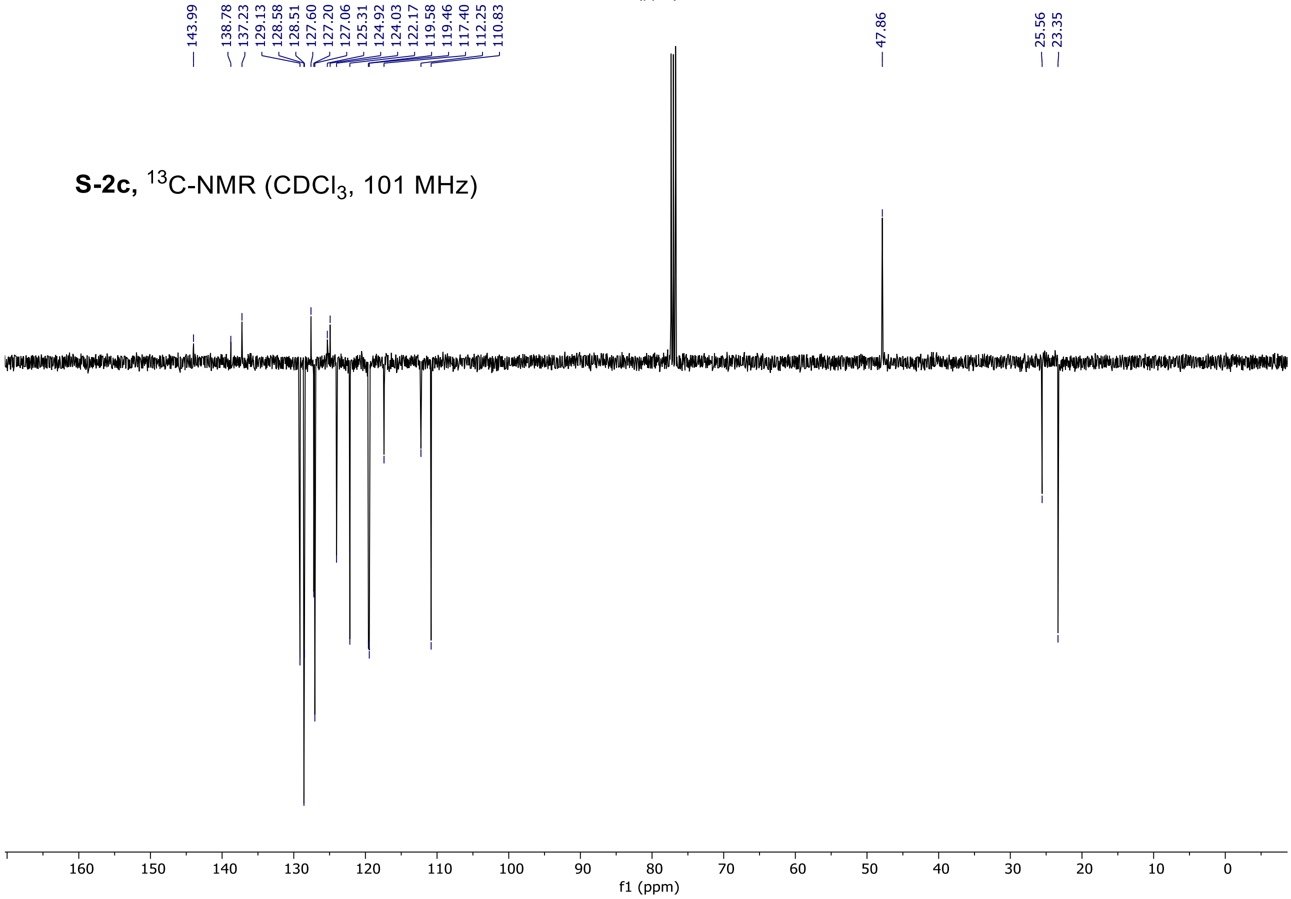

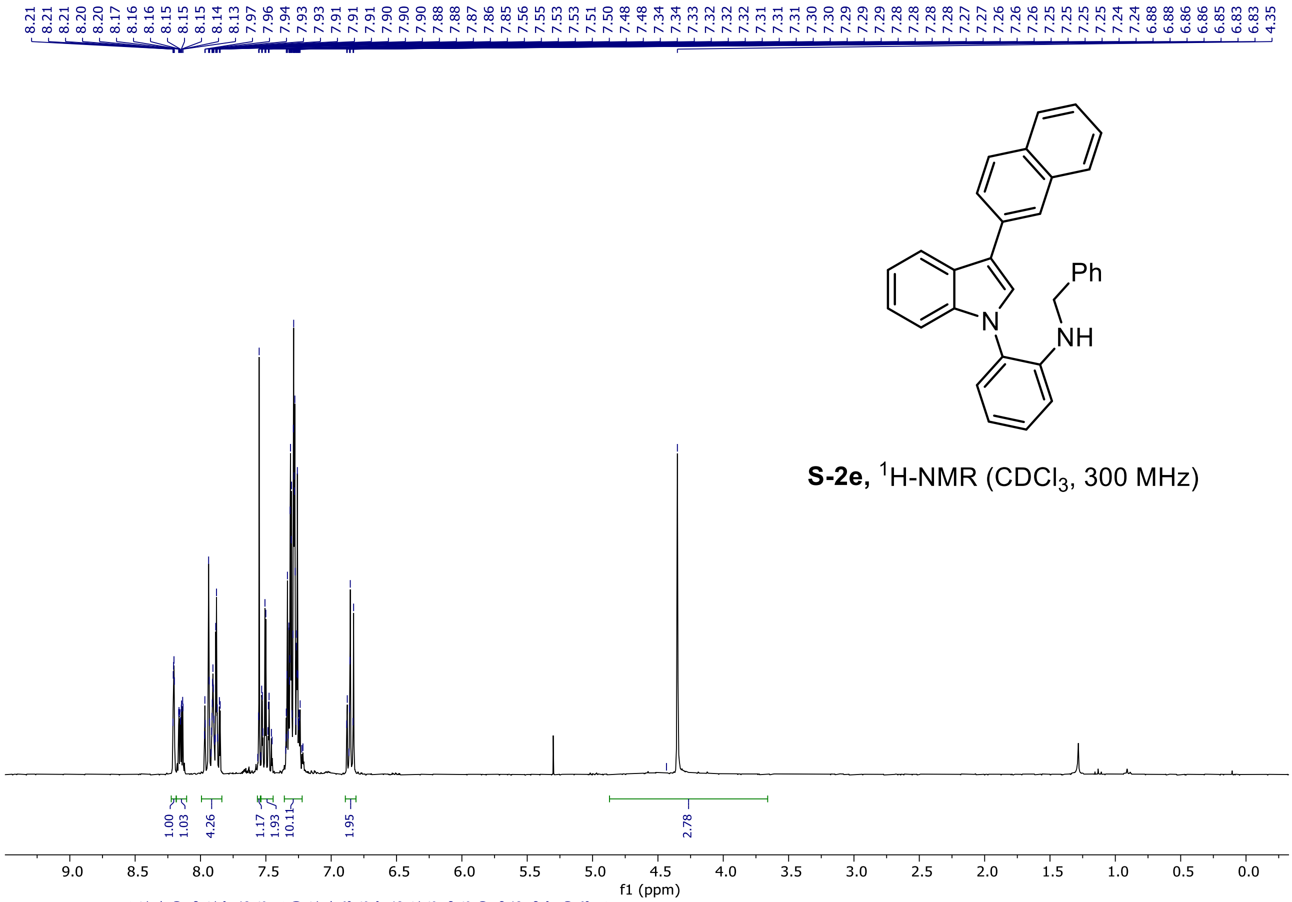

S-2e, ^{1}H-NMR ($CDCl_3$, 300 MHz)

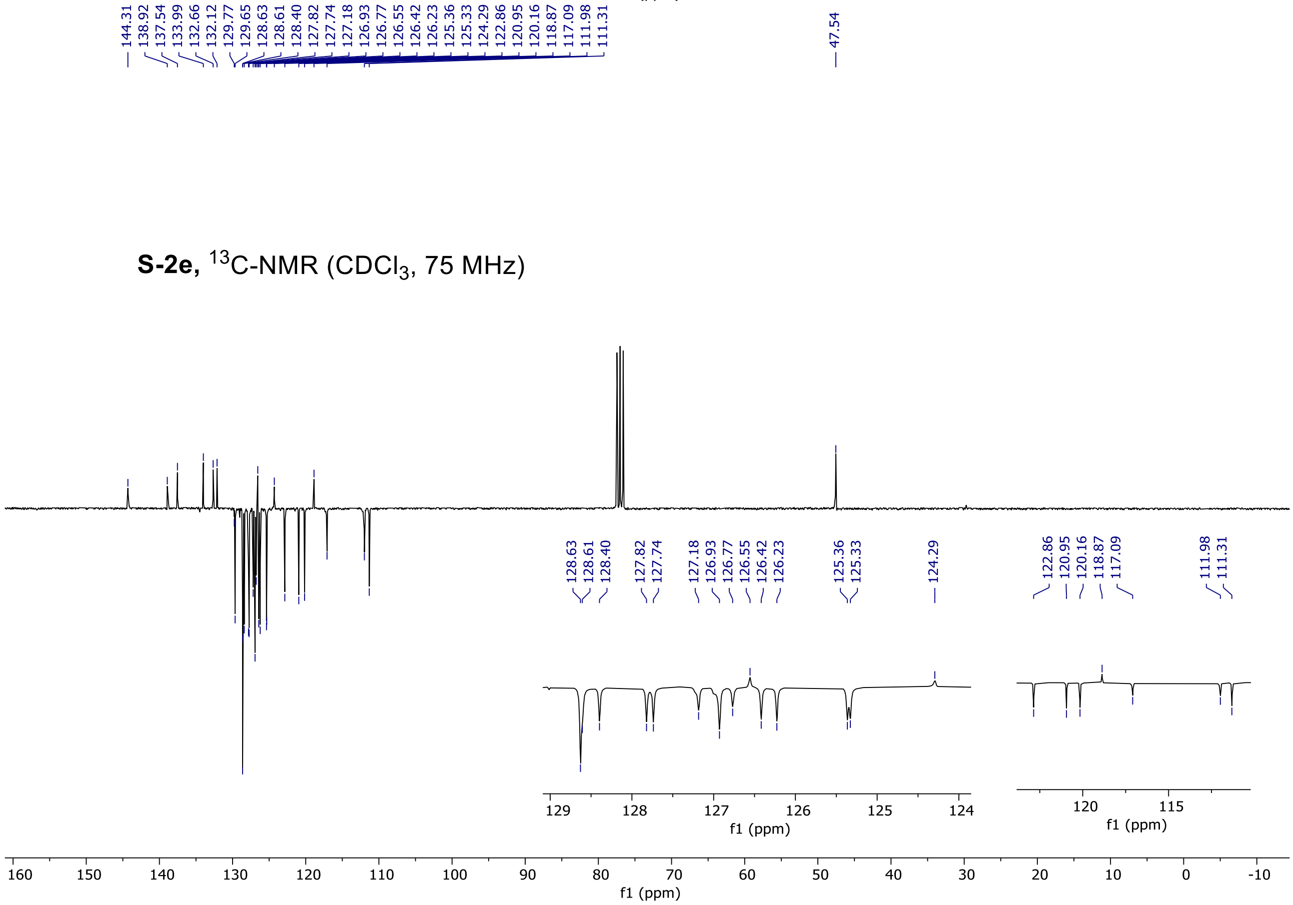

S-2e, ^{13}C-NMR ($CDCl_3$, 75 MHz)

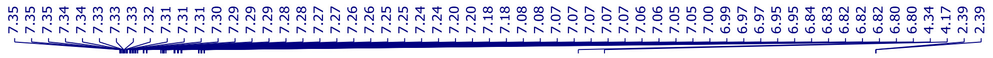

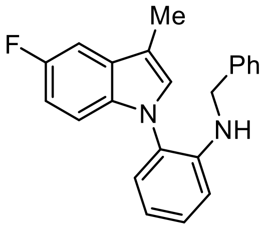

S-2f, ^{1}H-NMR ($CDCl_3$, 400 MHz)

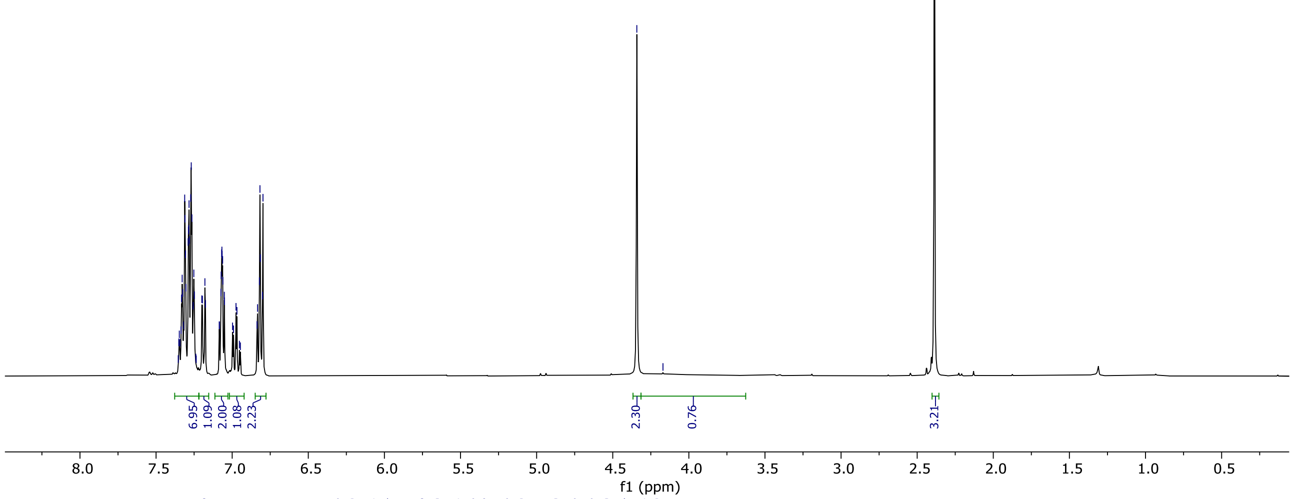

S-2f, ^{13}C-NMR ($CDCl_3$, 101 MHz)

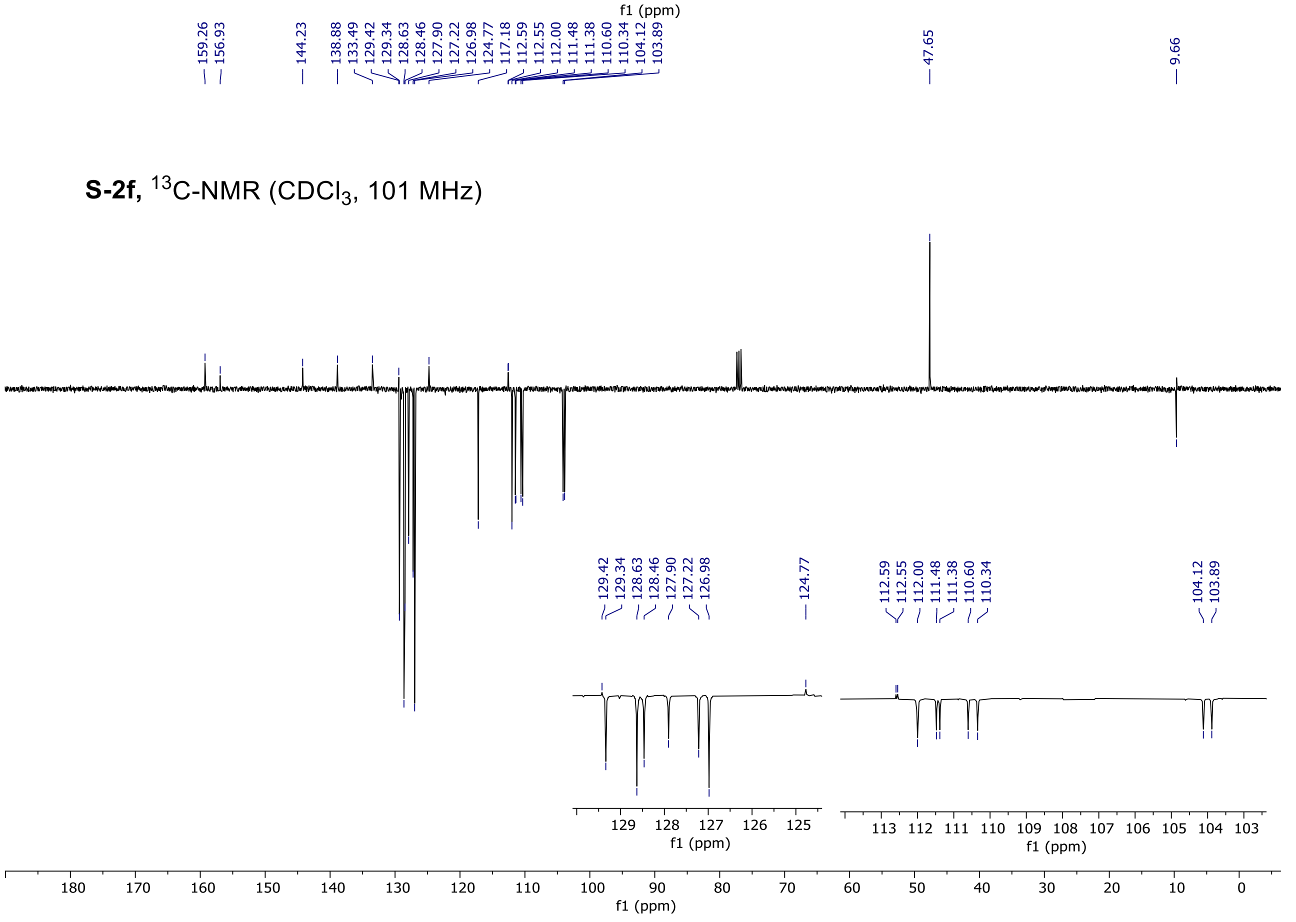

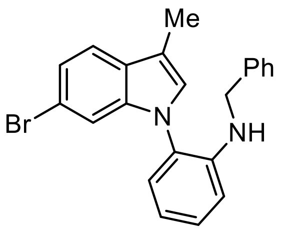

S-2h, ^{1}H-NMR ($CDCl_3$, 300 MHz)

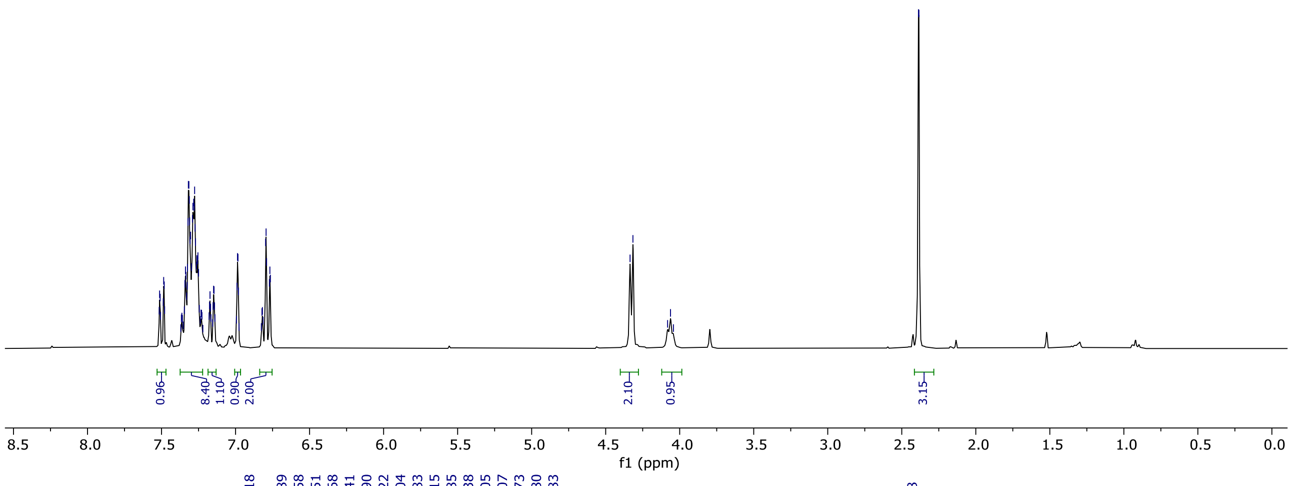

S-2h, ^{13}C-NMR ($CDCl_3$, 75 MHz)

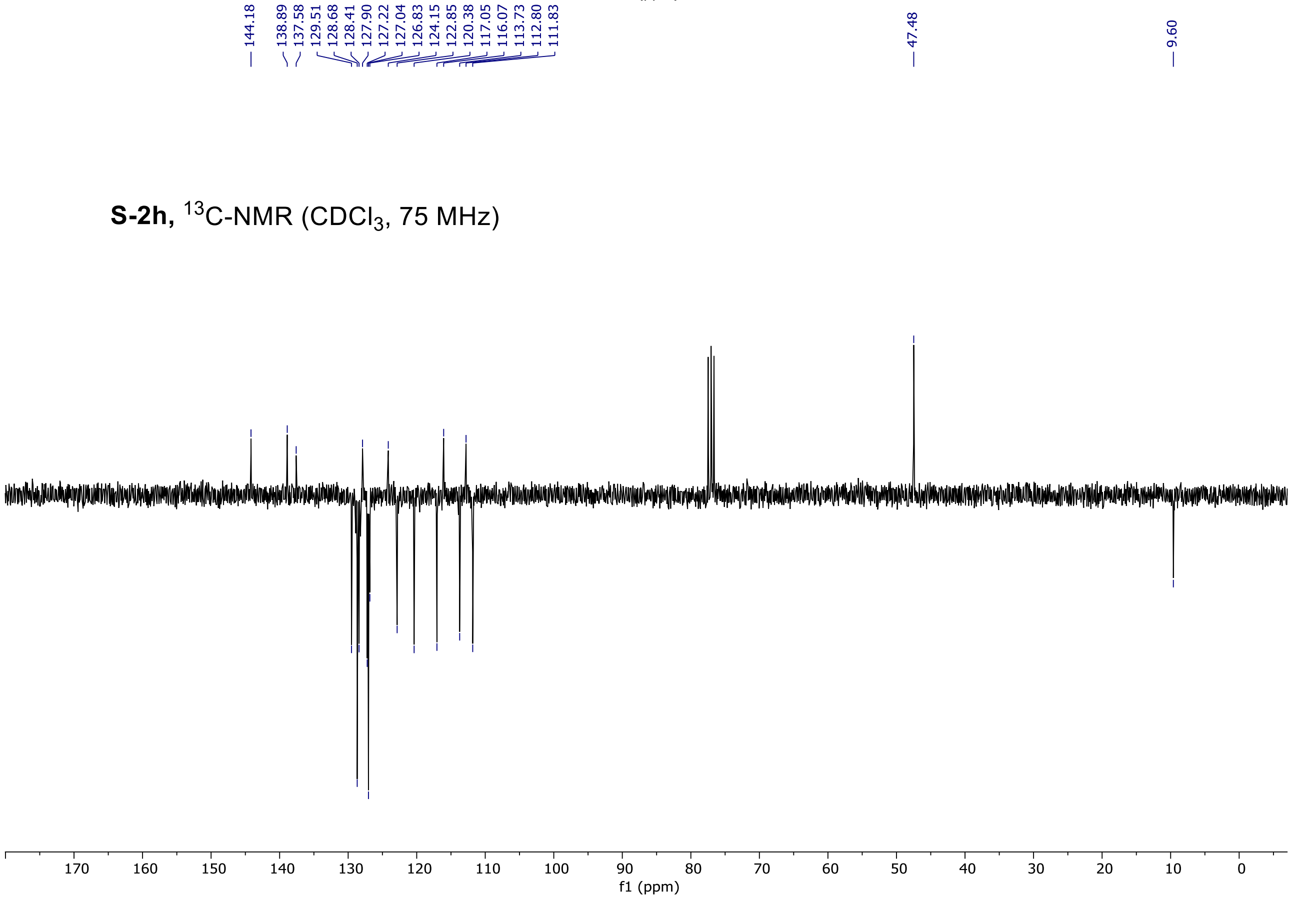

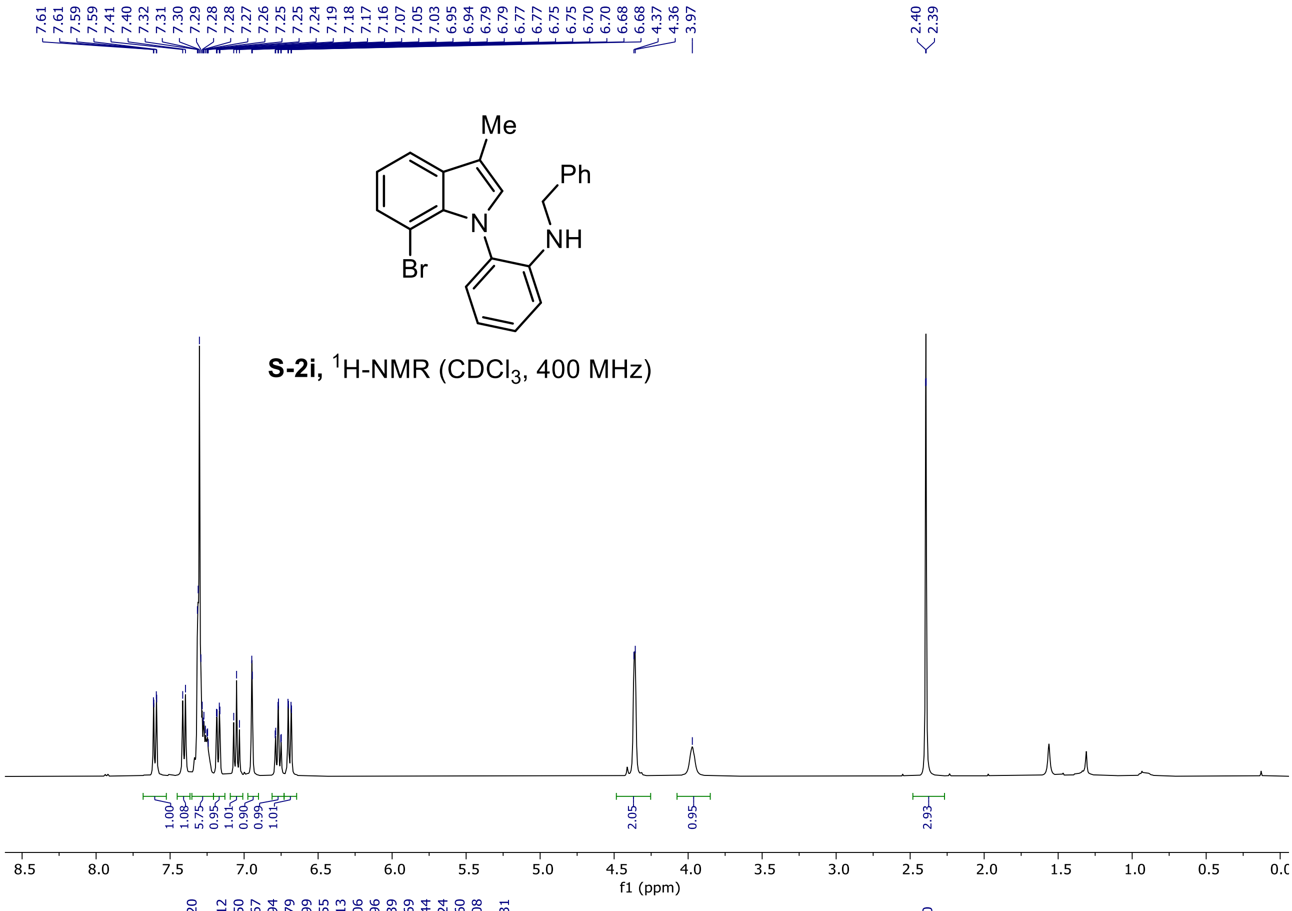

S-2i, ^{1}H-NMR ($CDCl_3$, 400 MHz)

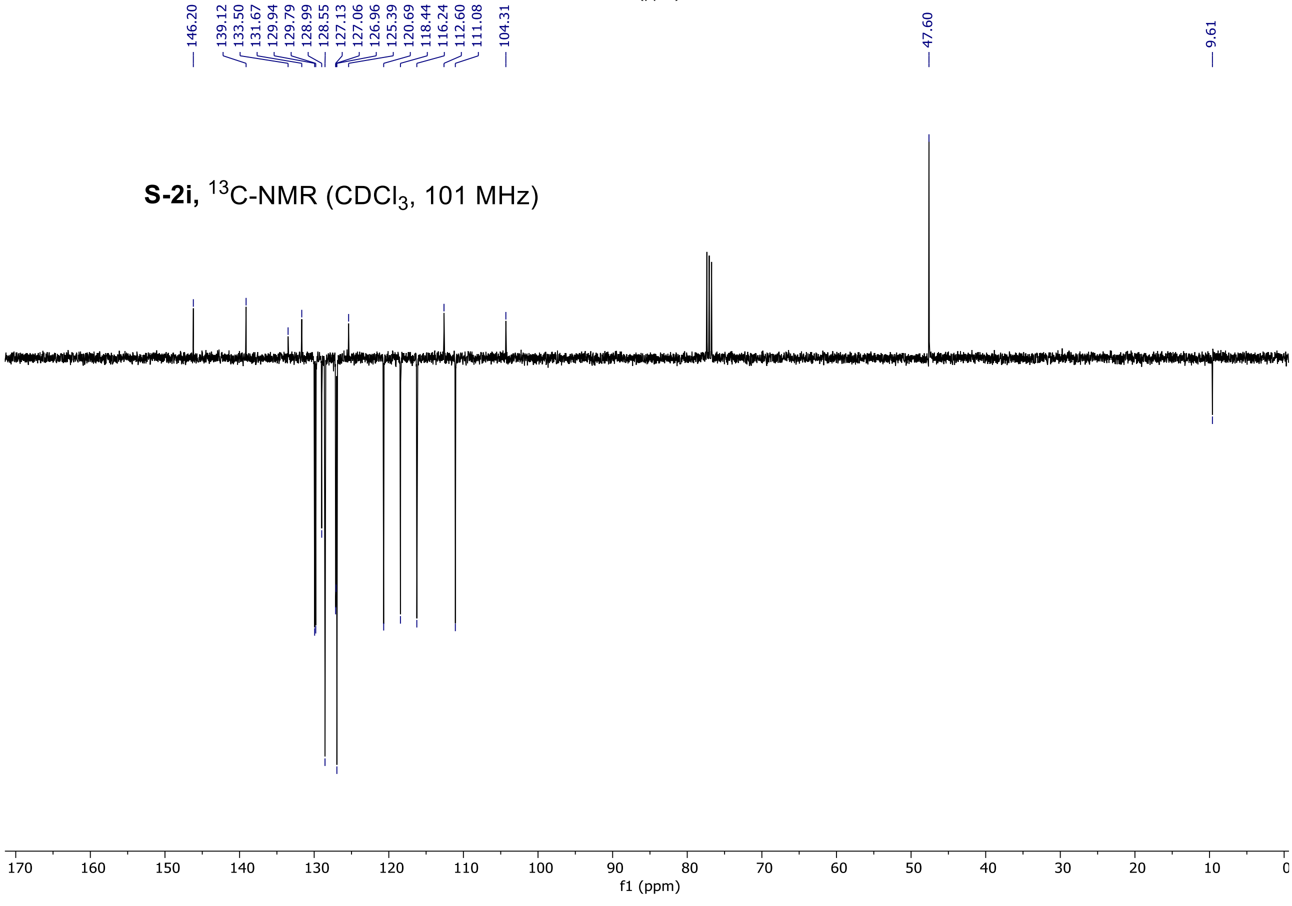

S-2i, ^{13}C-NMR ($CDCl_3$, 101 MHz)

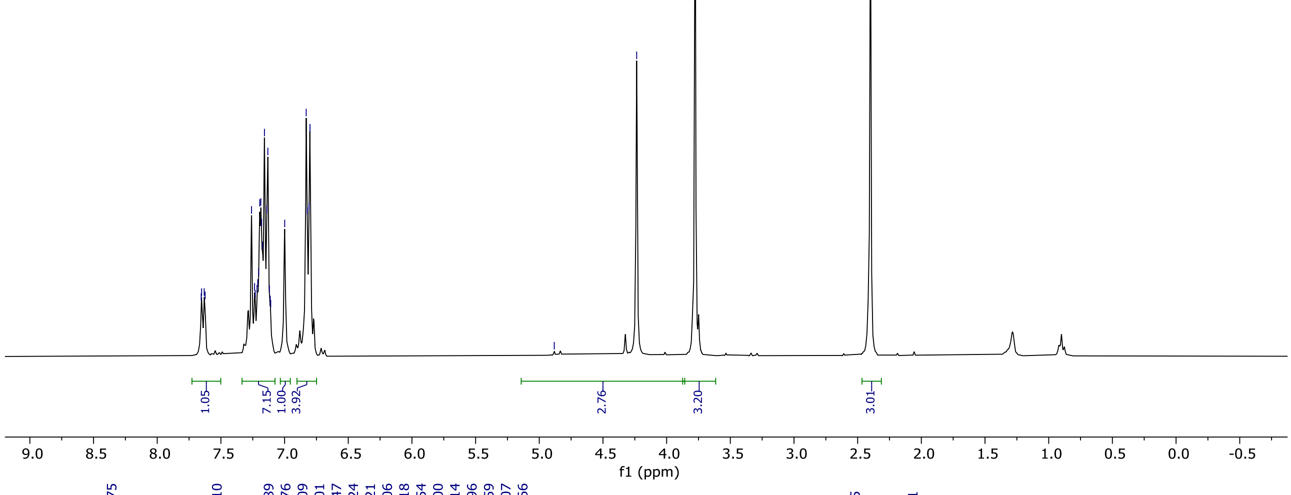

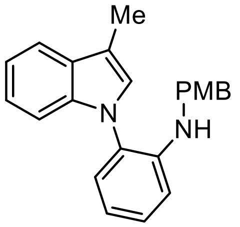

S-2j, ^{1}H-NMR ($CDCl_3$, 300 MHz)

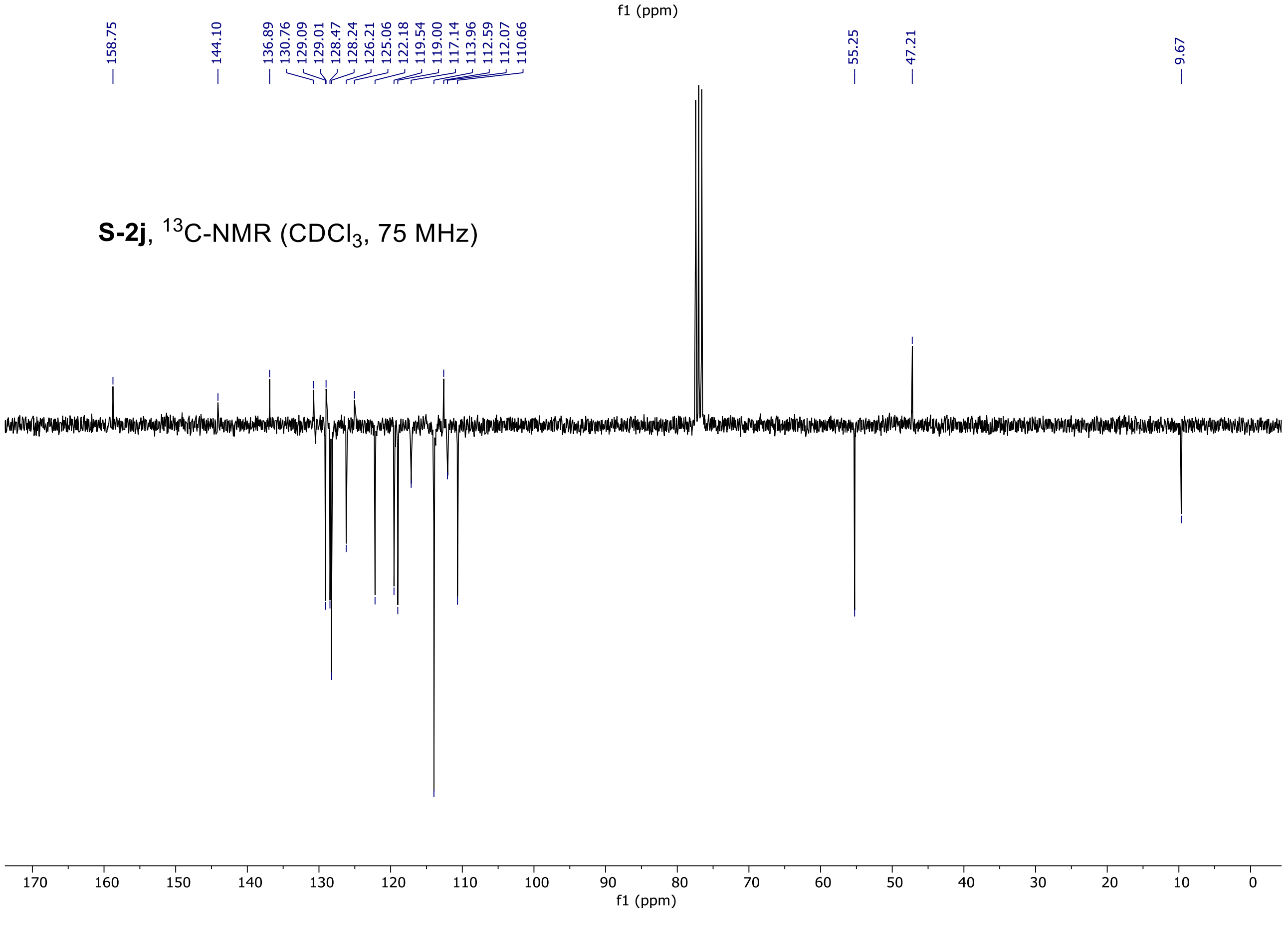

S-2j, ^{13}C-NMR ($CDCl_3$, 75 MHz)

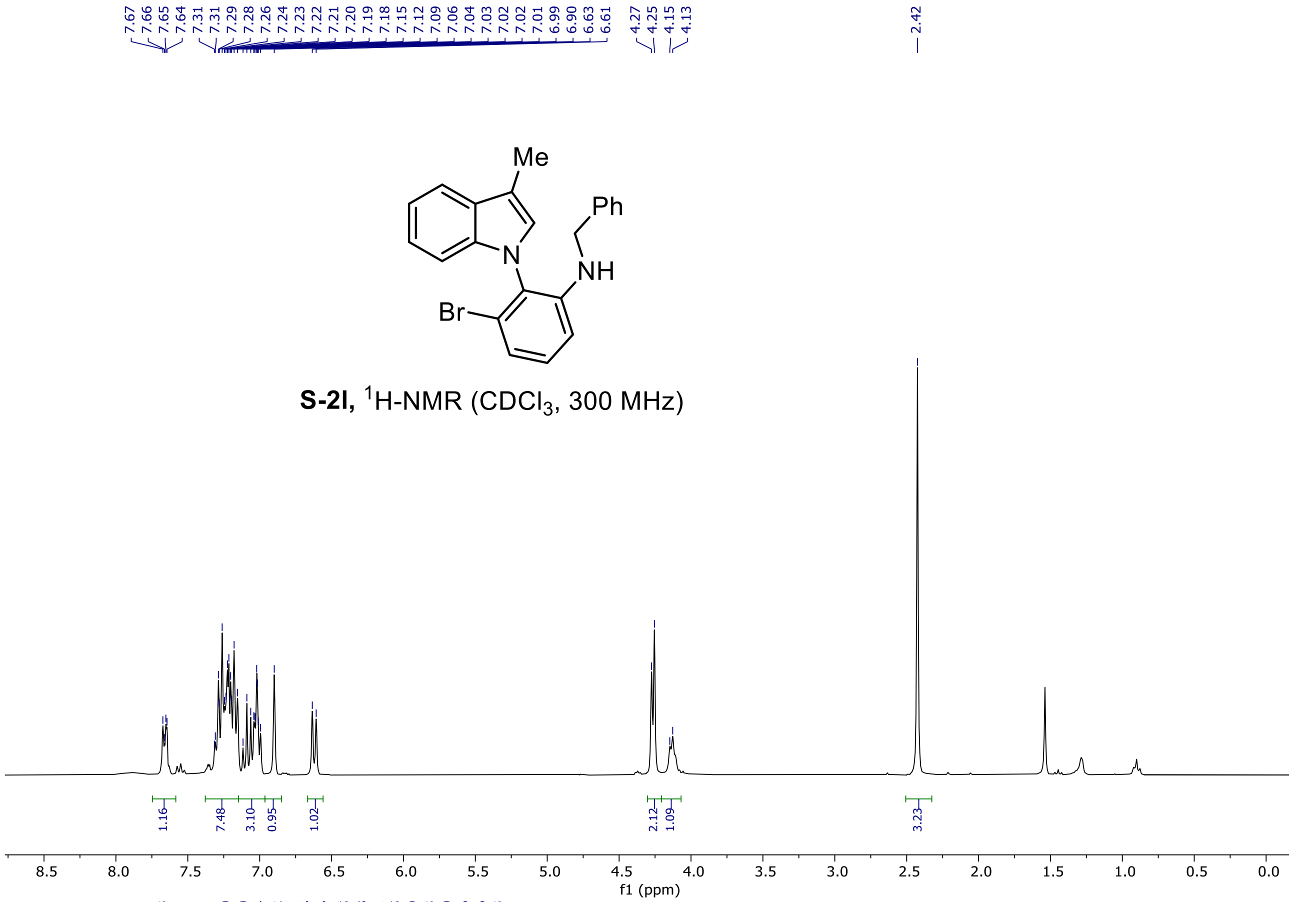

S-2l, ^{1}H-NMR ($CDCl_3$, 300 MHz)

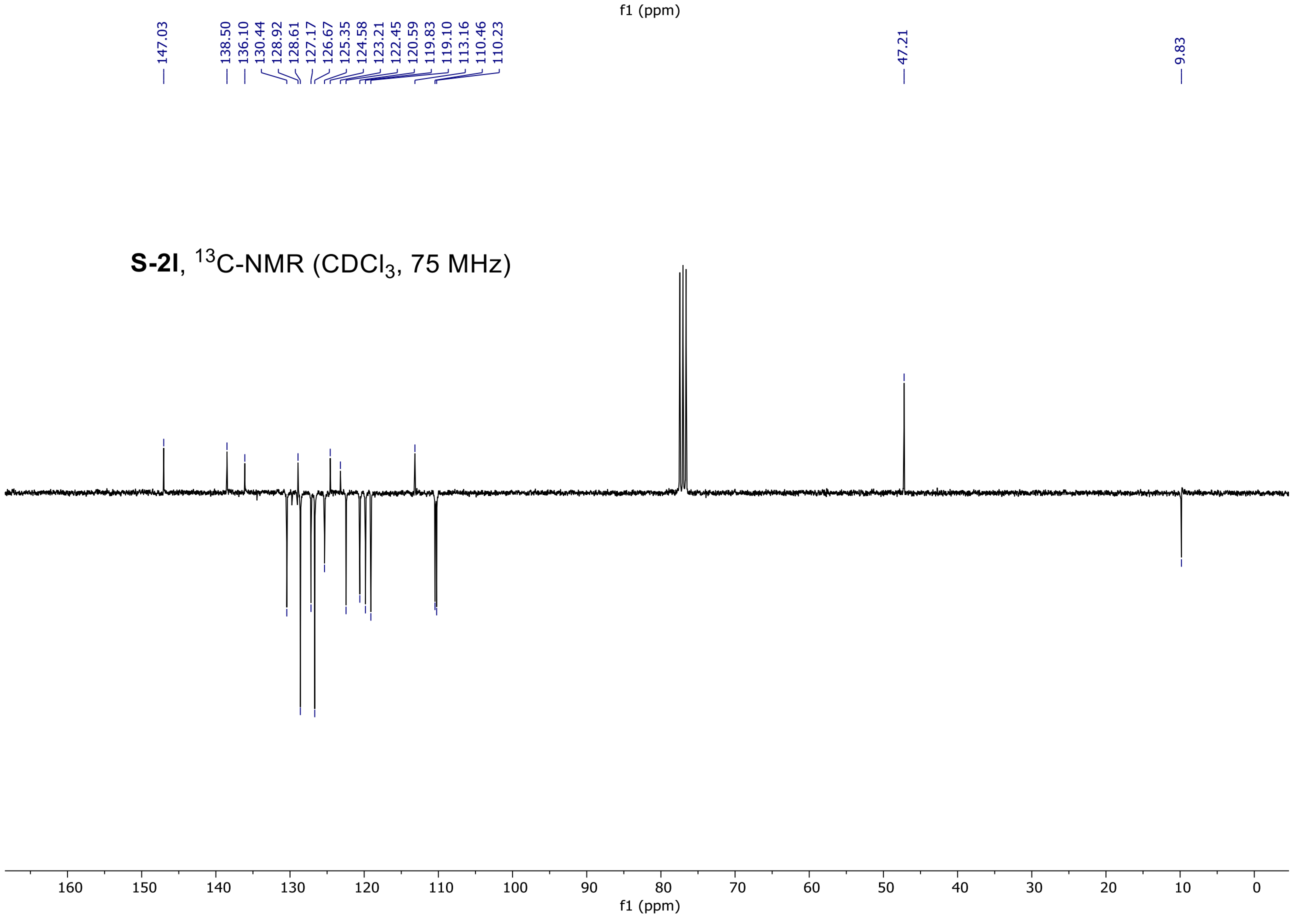

S-2l, ^{13}C-NMR ($CDCl_3$, 75 MHz)

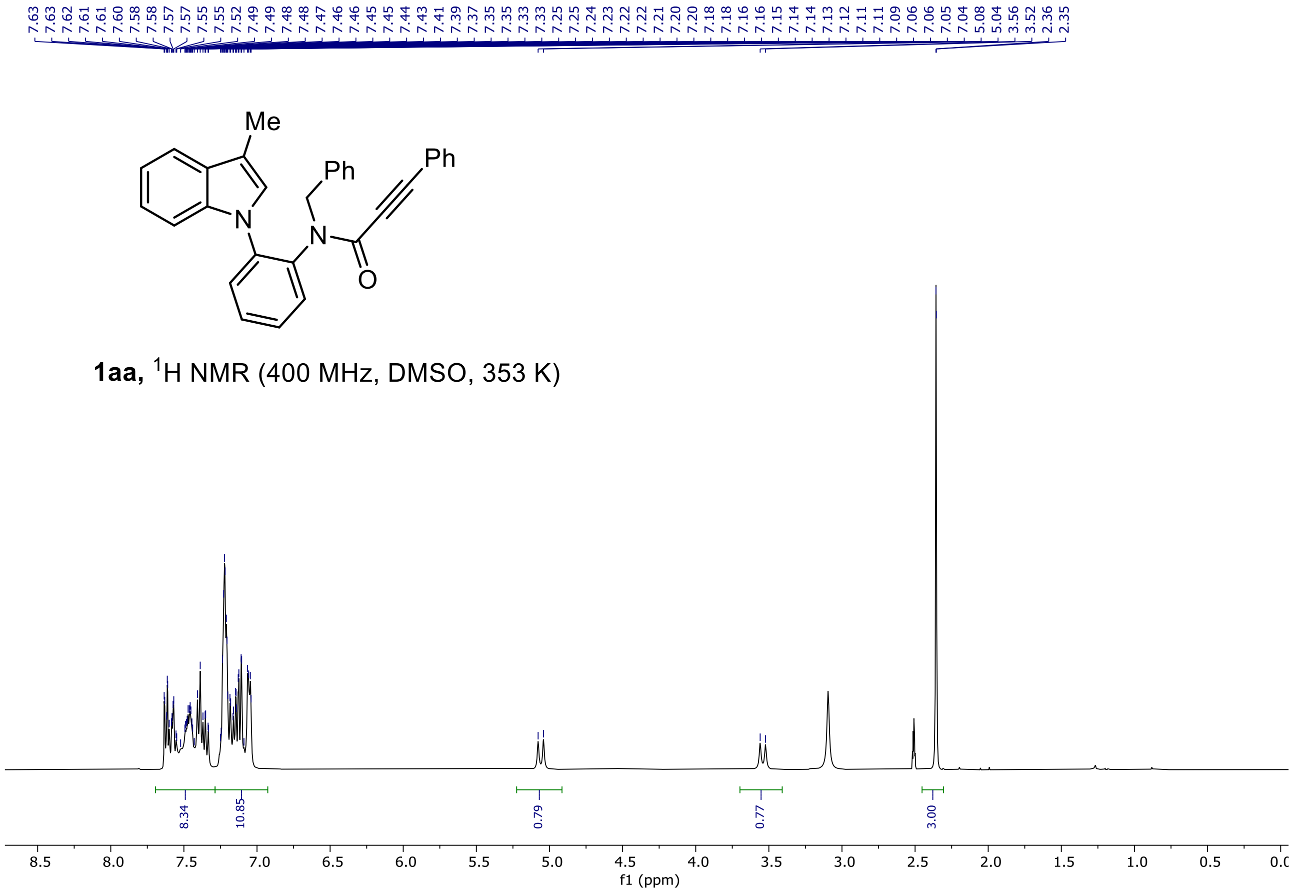
Me
Ph
Ph
N
N
O
1aa, ^{1}H NMR (400 MHz, DMSO, 353 K)
f1 (ppm)

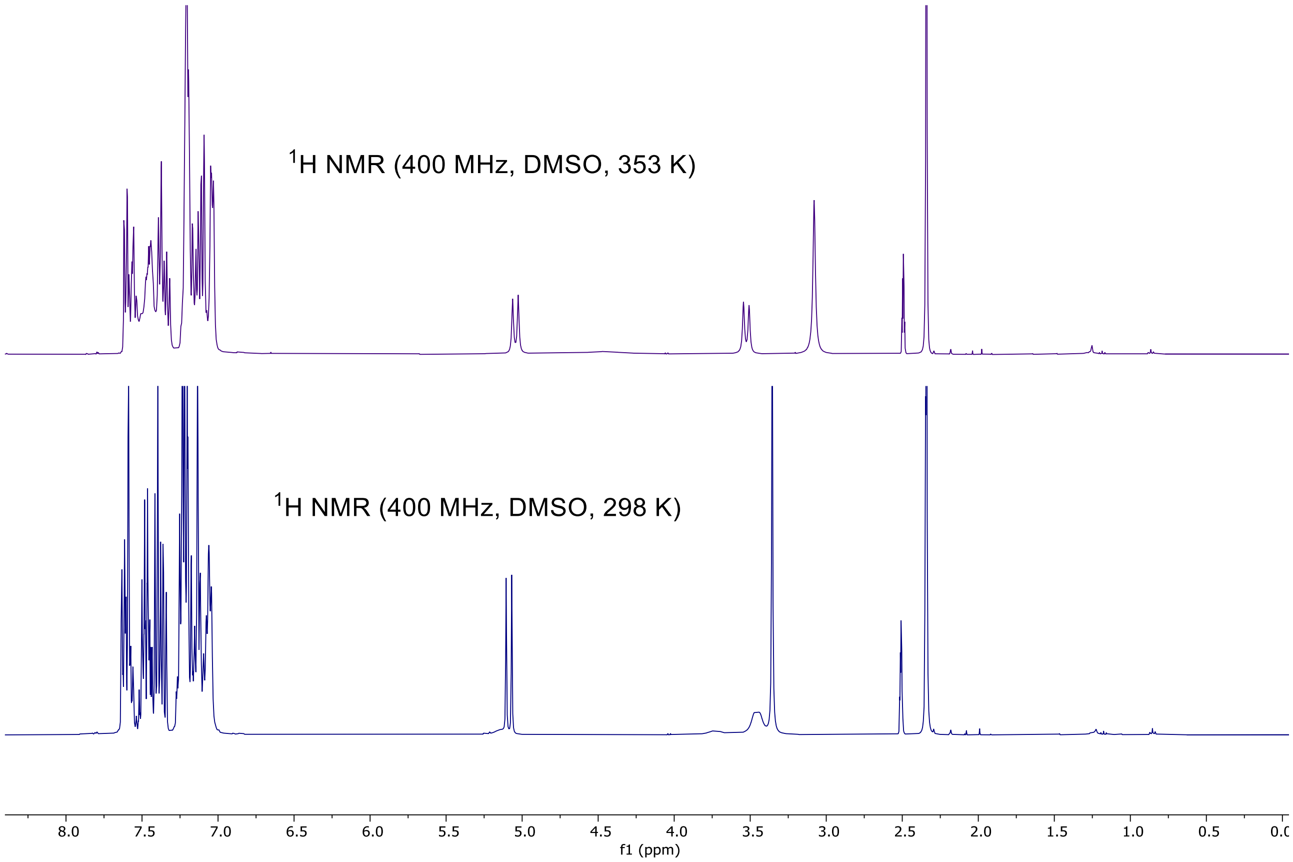
^{1}H NMR (400 MHz, DMSO, 353 K)
^{1}H NMR (400 MHz, DMSO, 298 K)
f1 (ppm)

1aa, ^{13}C NMR (101 MHz, DMSO, 353 K)

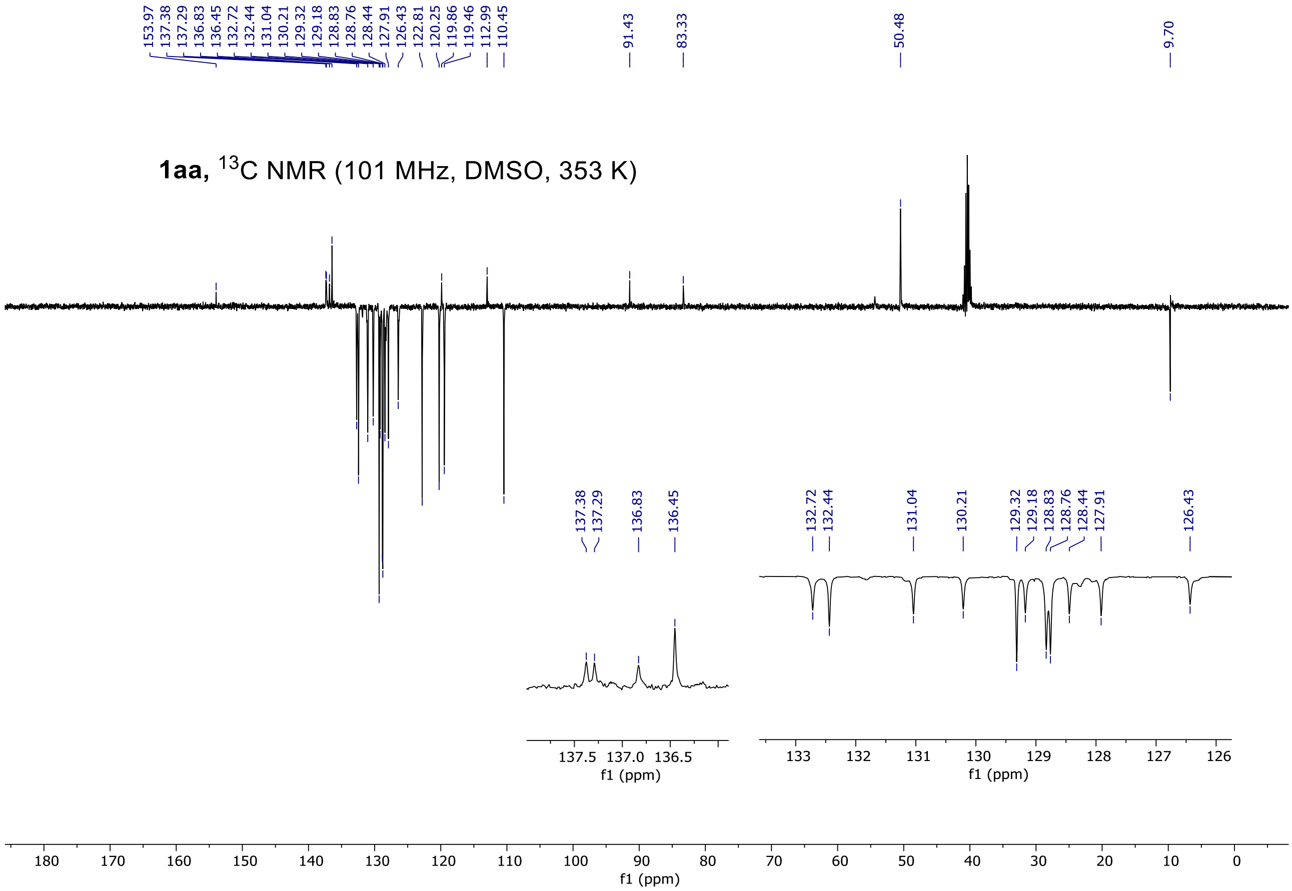

1aa, HMBC (DMSO, 298 K)

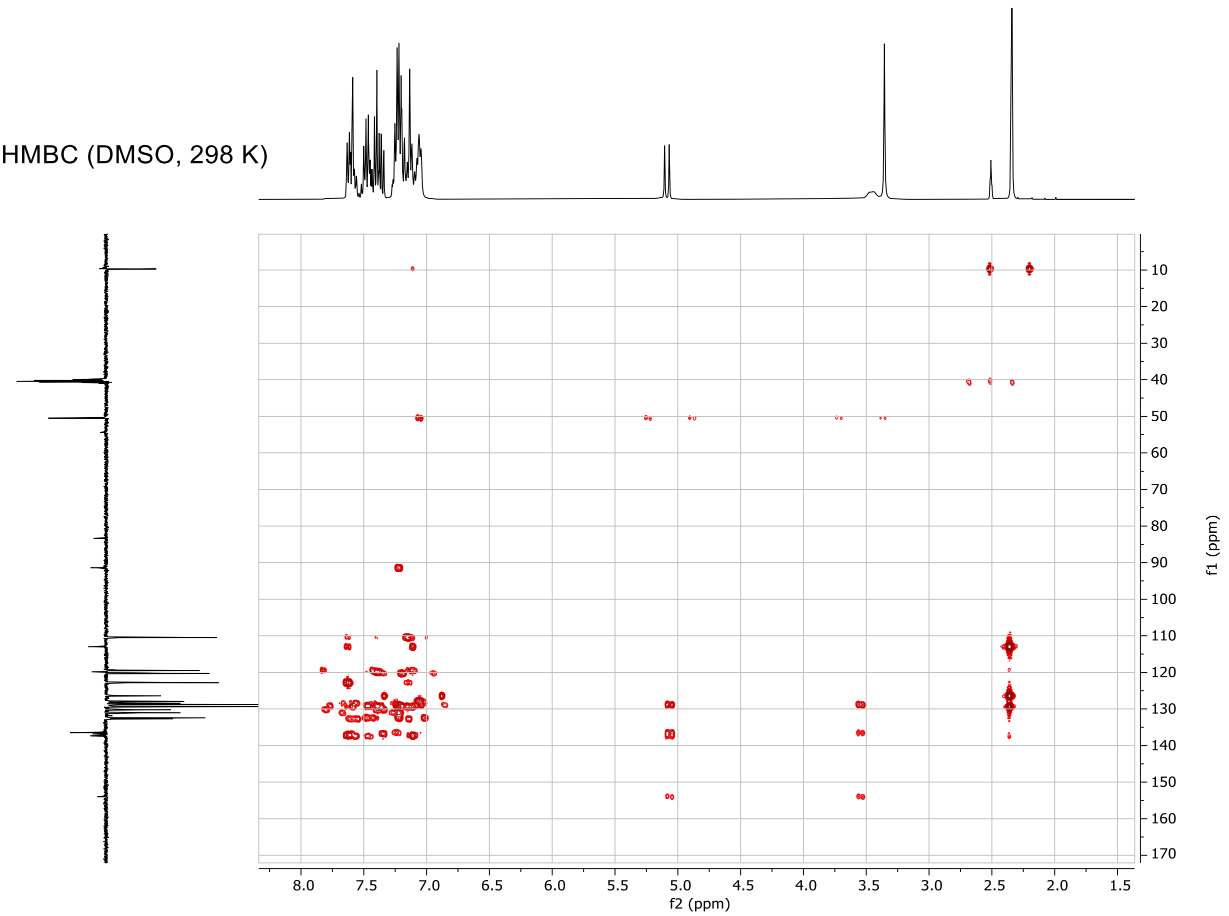

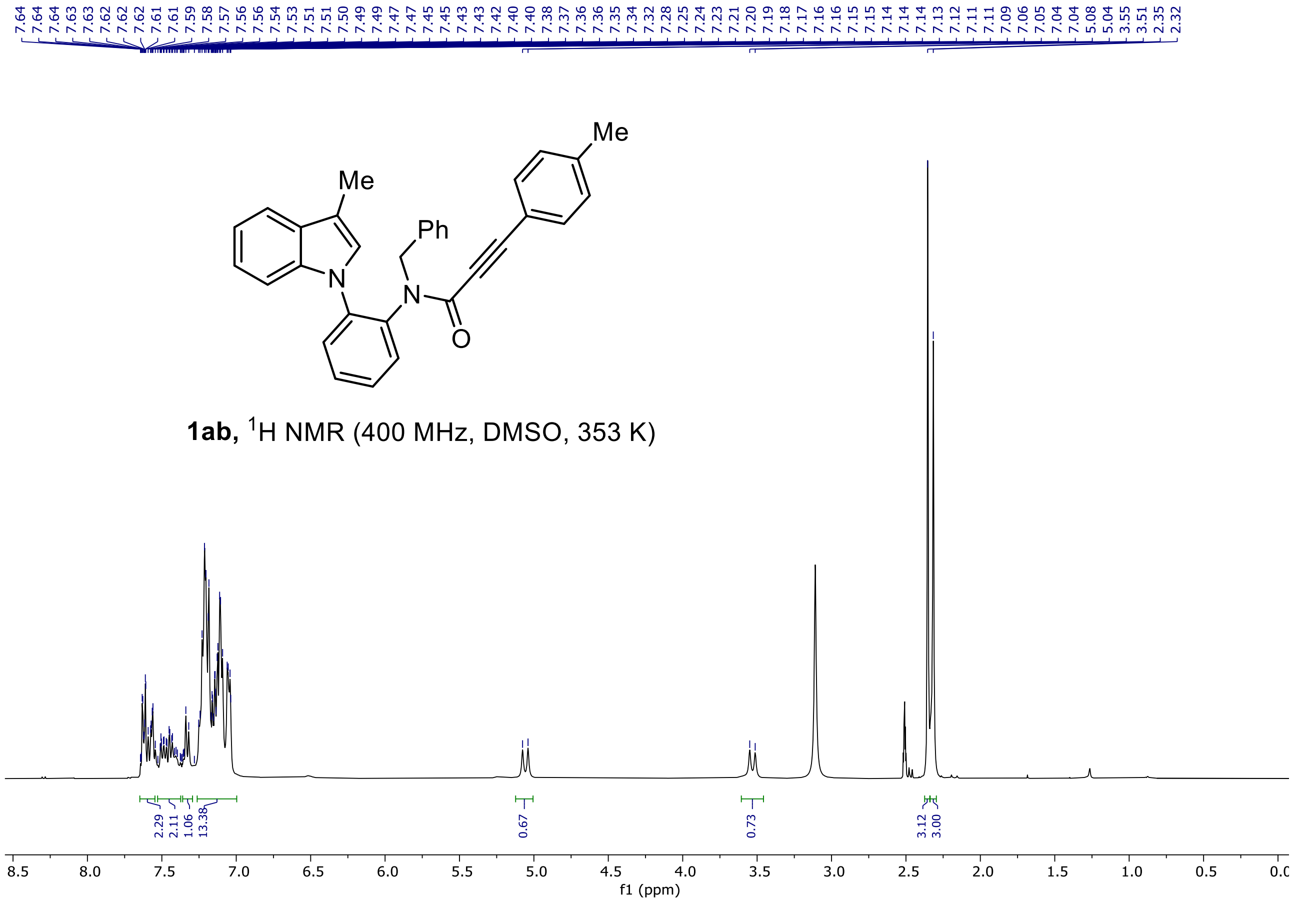

1ab, ^{1}H NMR (400 MHz, DMSO, 353 K)

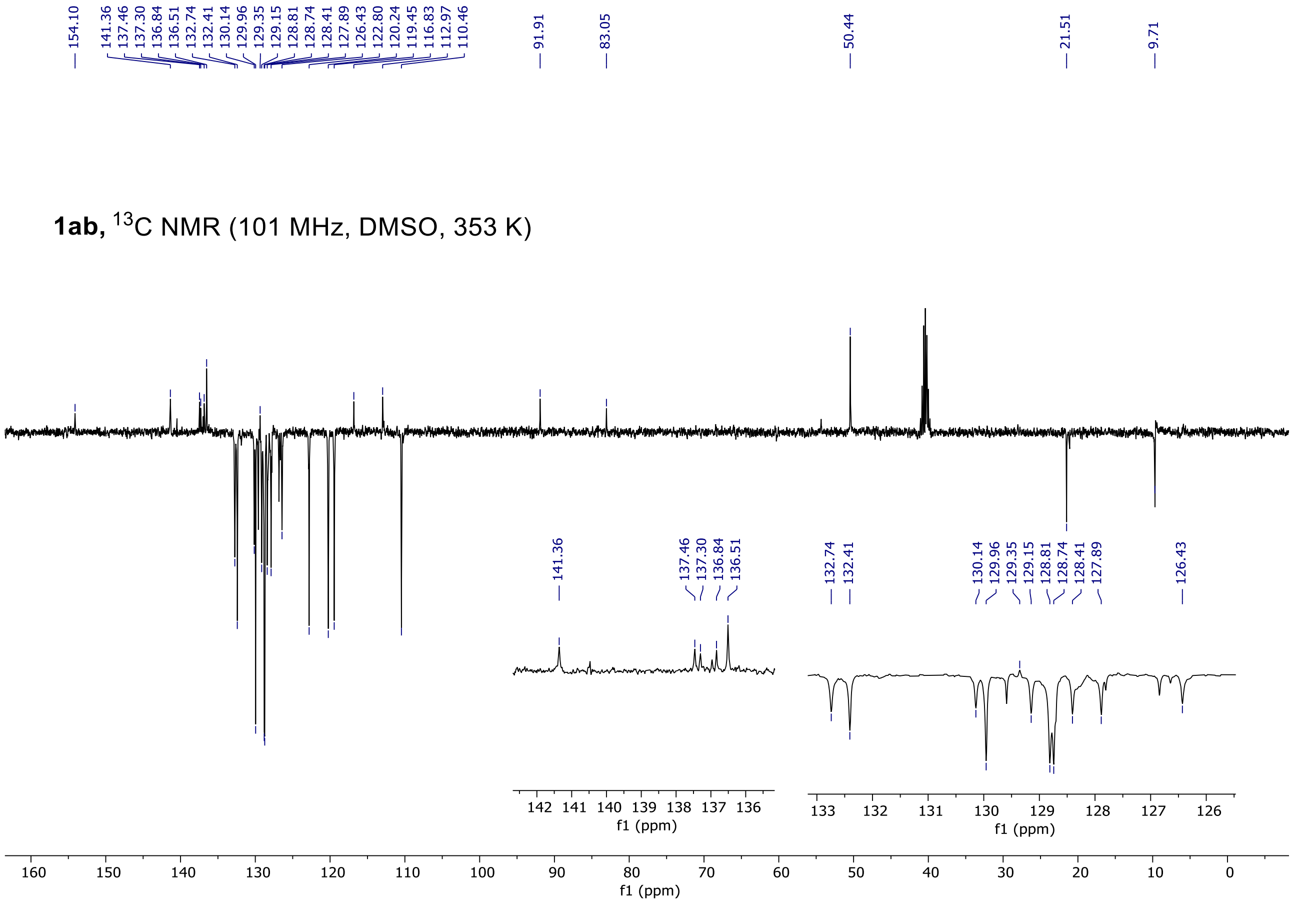

1ab, ^{13}C NMR (101 MHz, DMSO, 353 K)

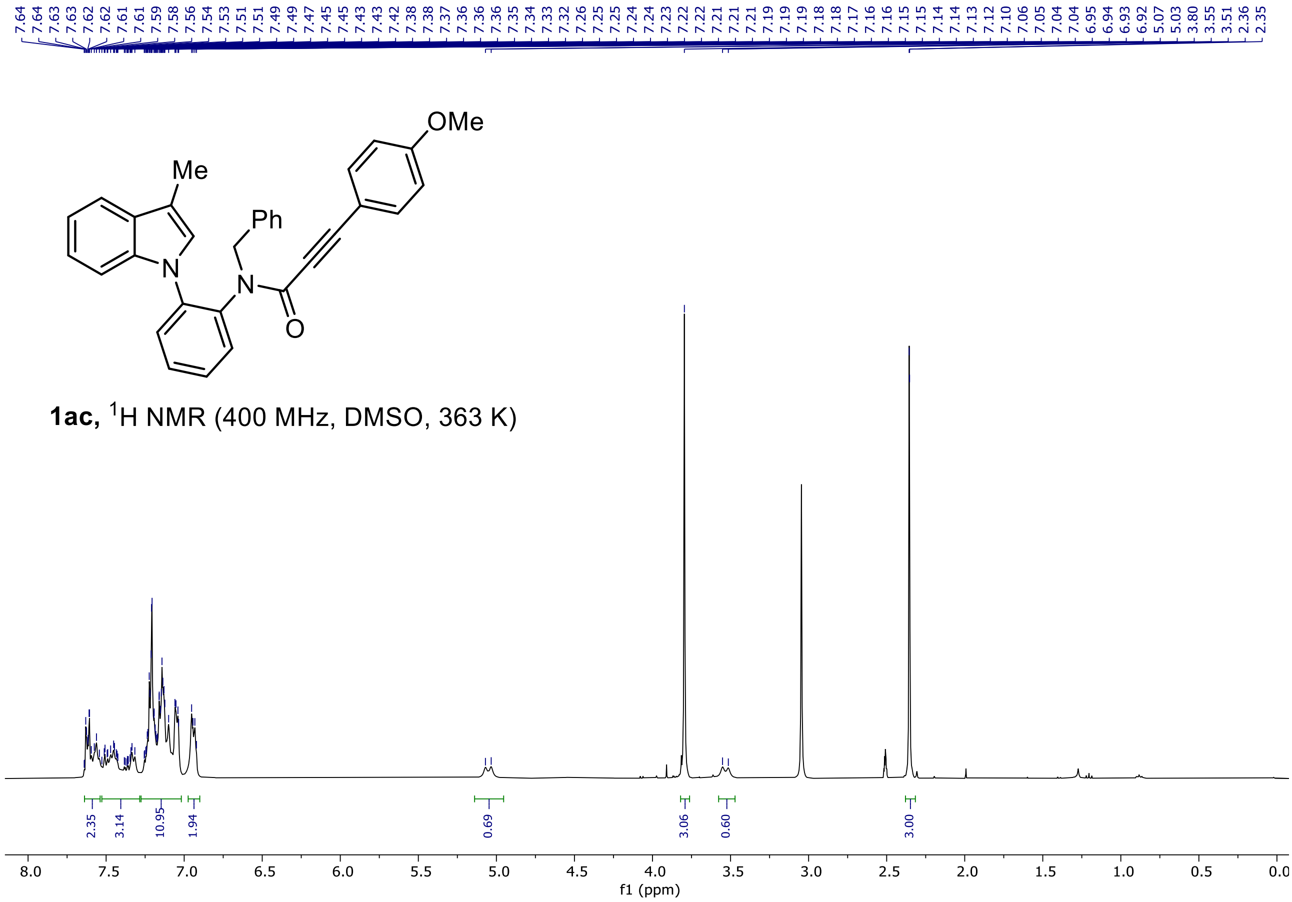

1ac, ^{1}H NMR (400 MHz, DMSO, 363 K)

1ac, ^{13}C NMR (101 MHz, DMSO, 353 K)

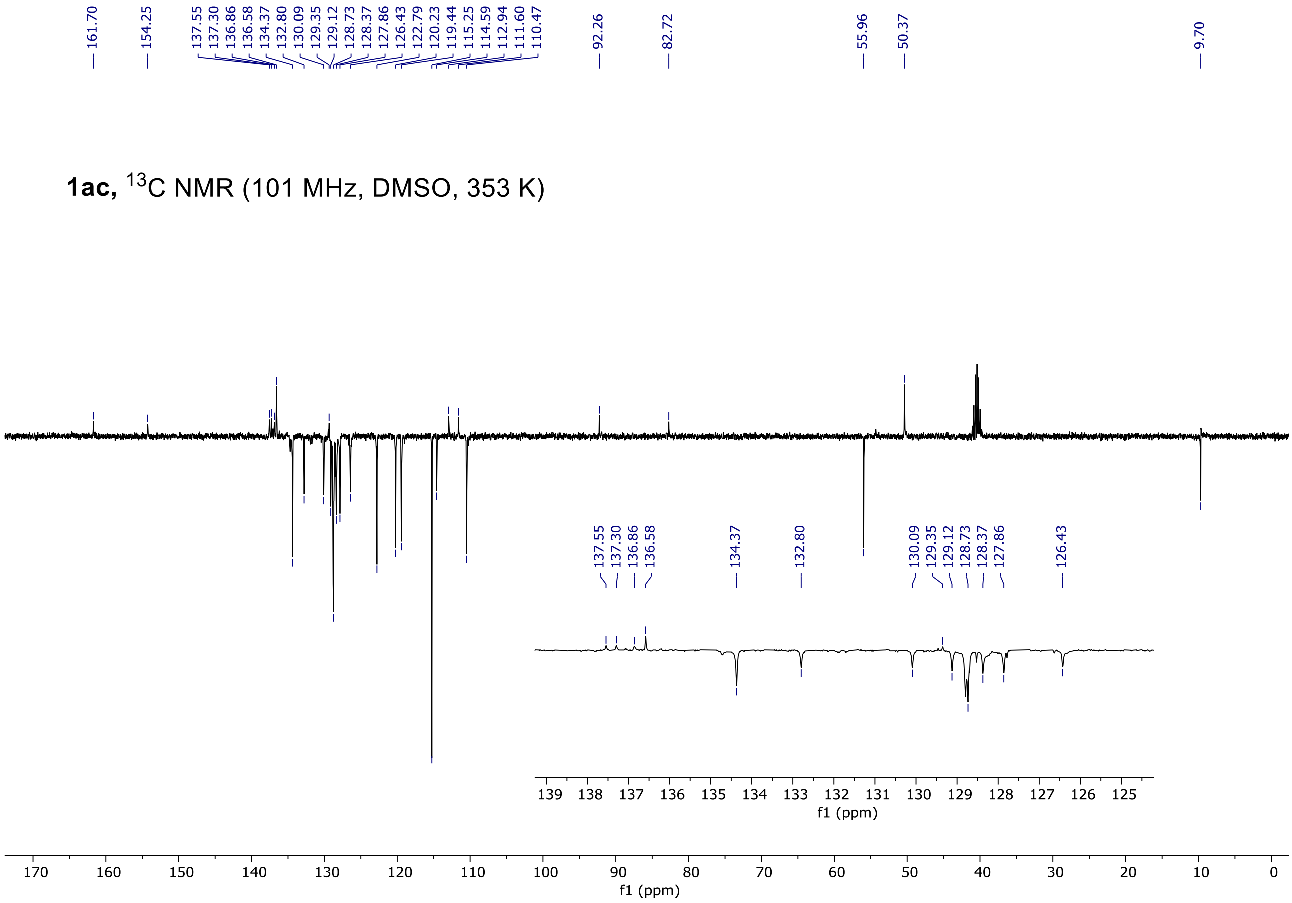

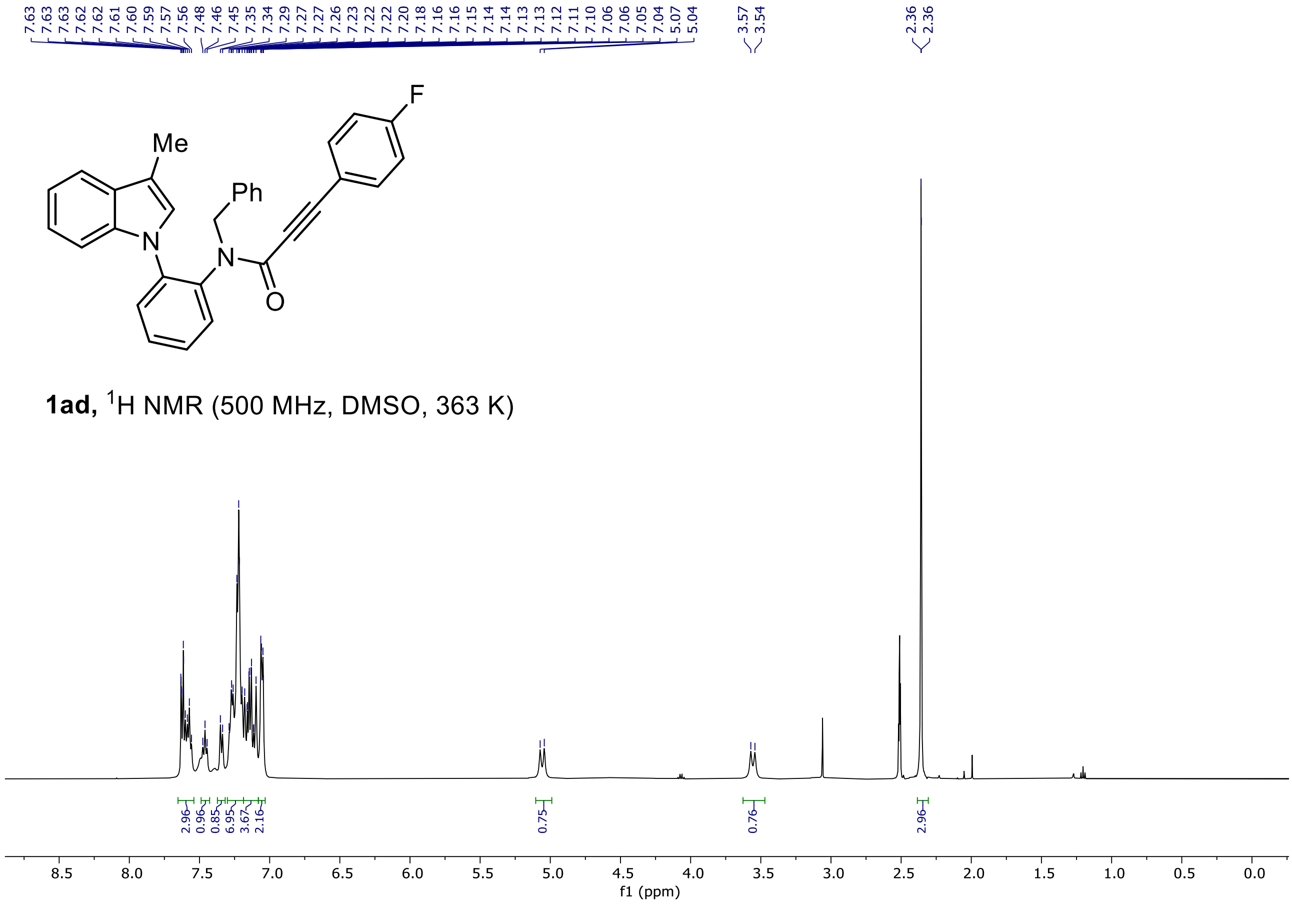

1ad, ^{1}H NMR (500 MHz, DMSO, 363 K)

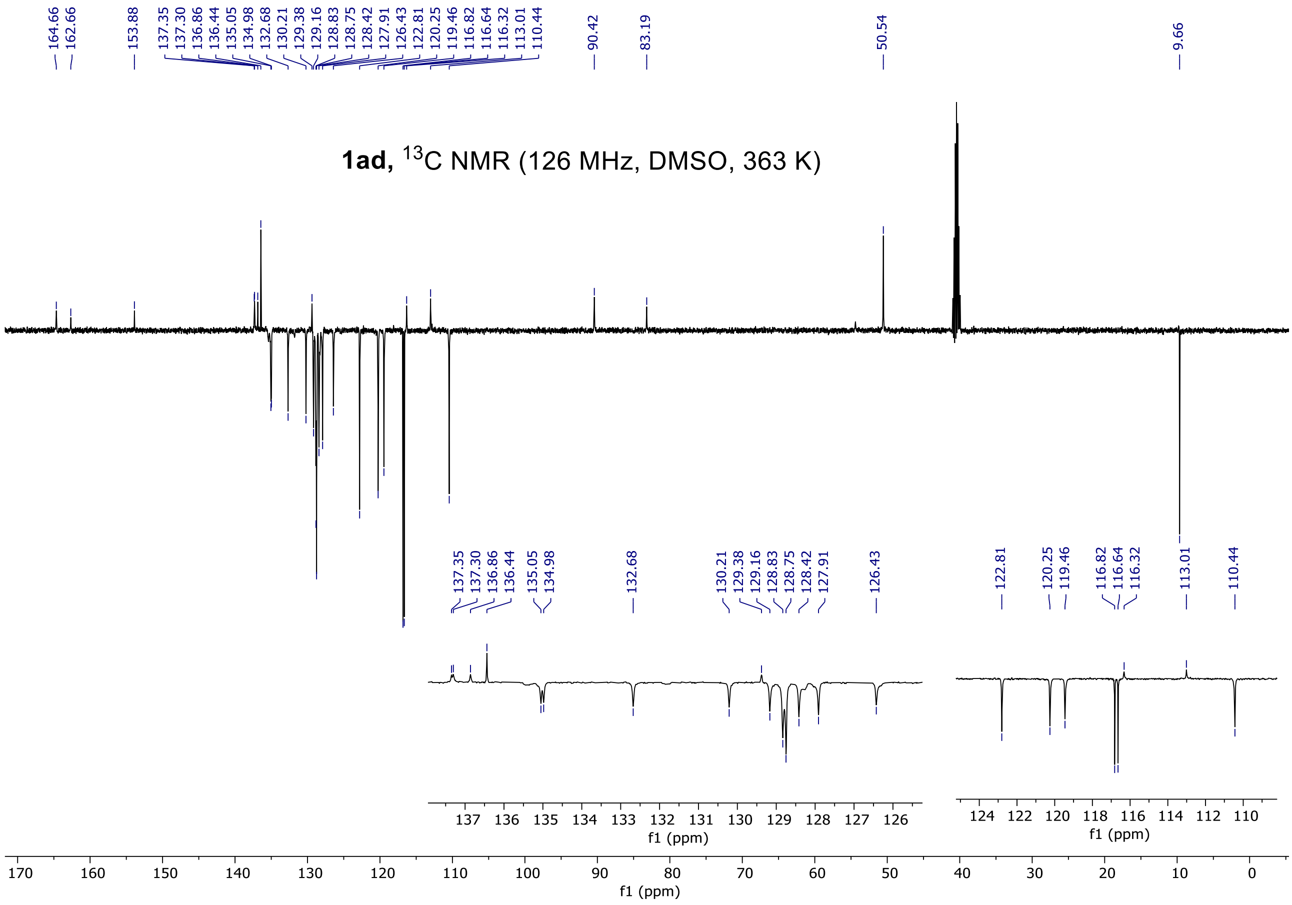

1ad, ^{13}C NMR (126 MHz, DMSO, 363 K)

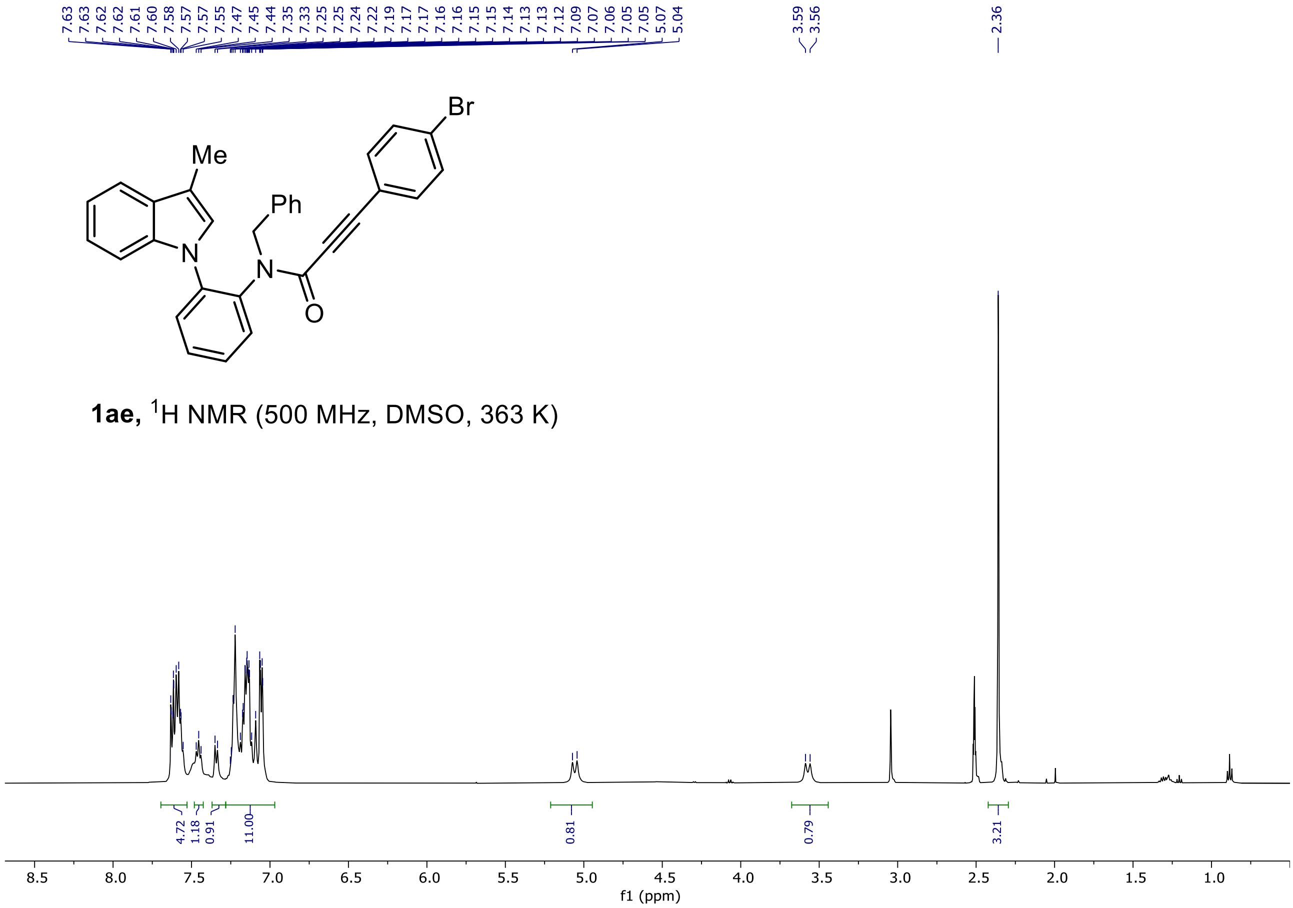

1ae, ^{1}H NMR (500 MHz, DMSO, 363 K)

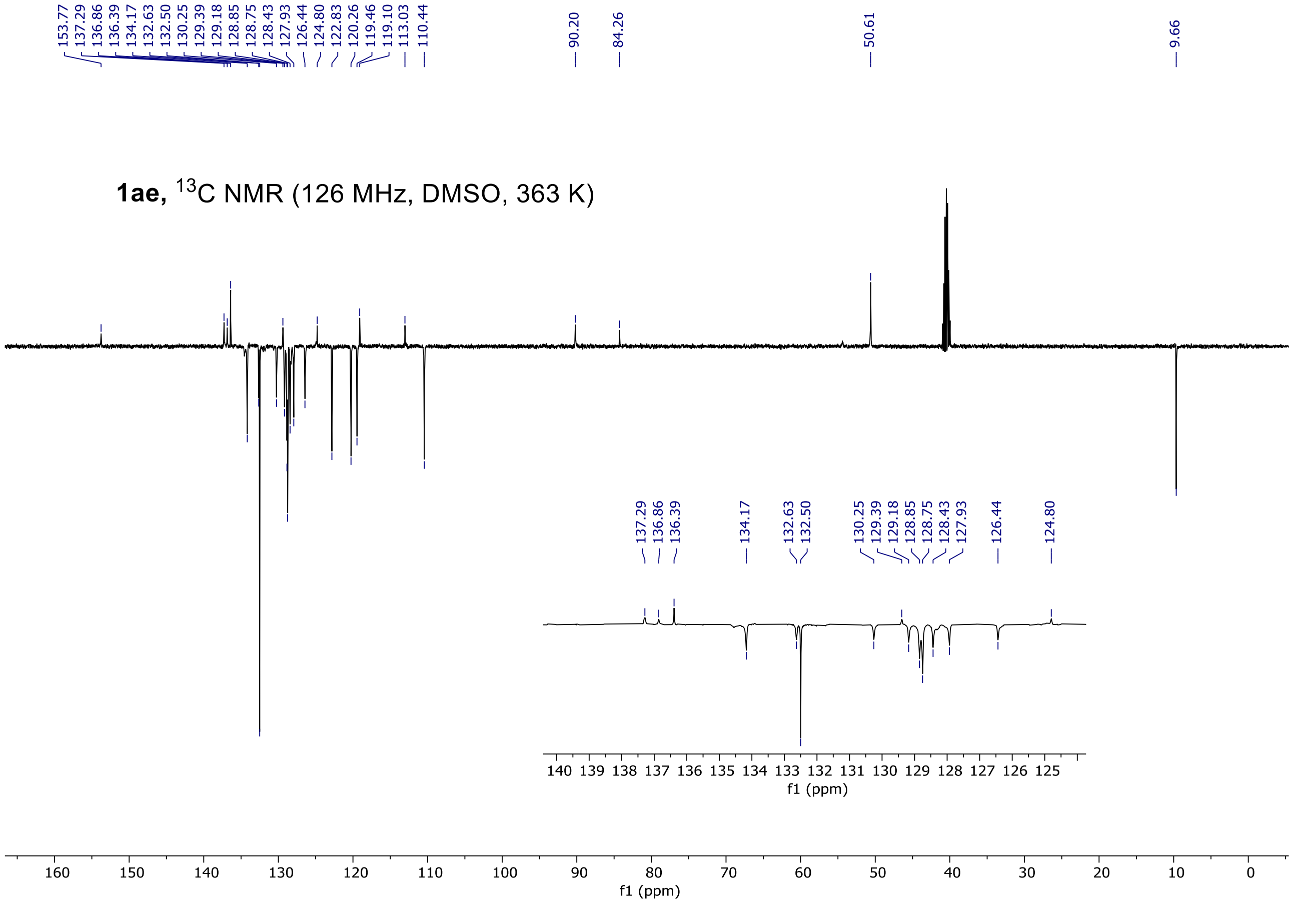

1ae, ^{13}C NMR (126 MHz, DMSO, 363 K)

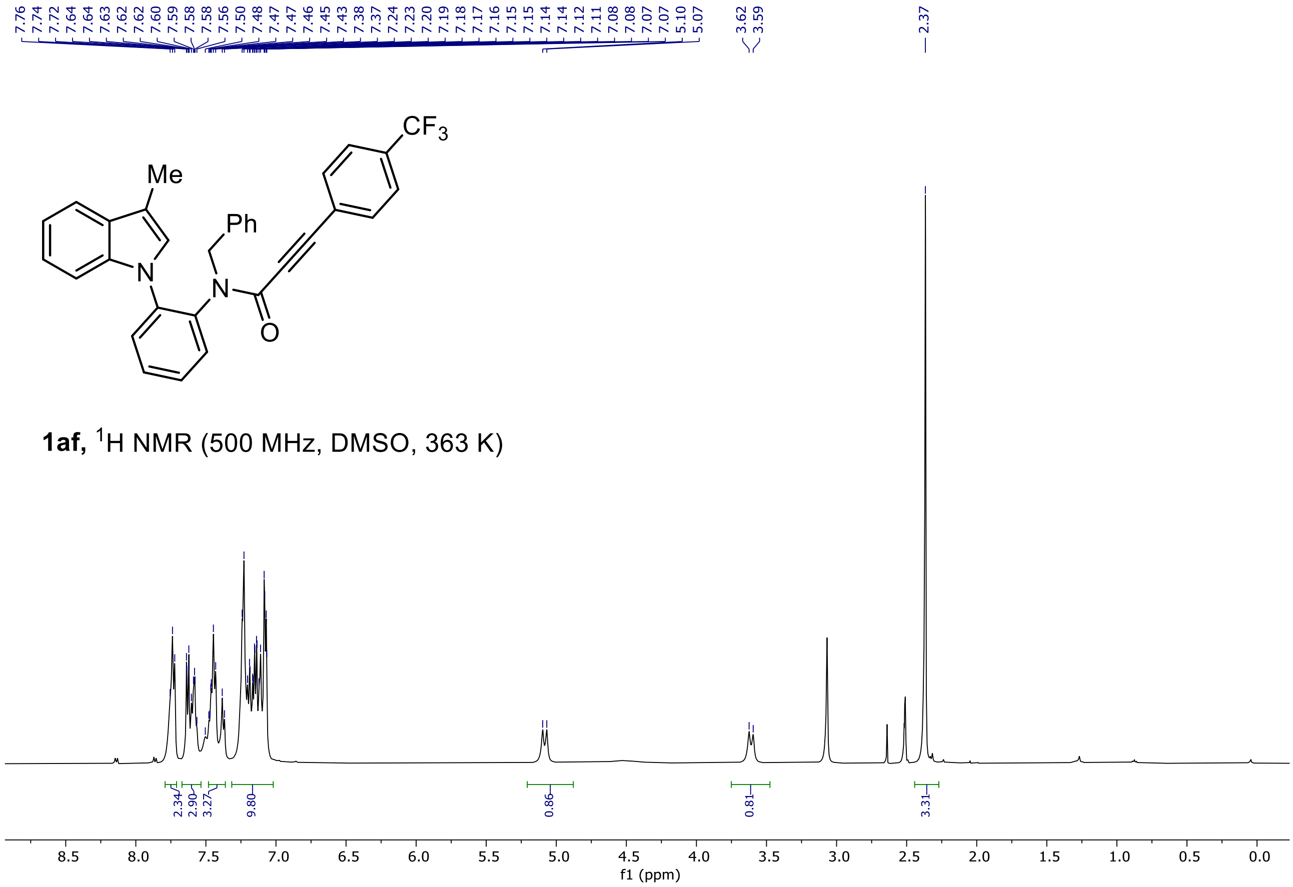

1af, ^{1}H NMR (500 MHz, DMSO, 363 K)

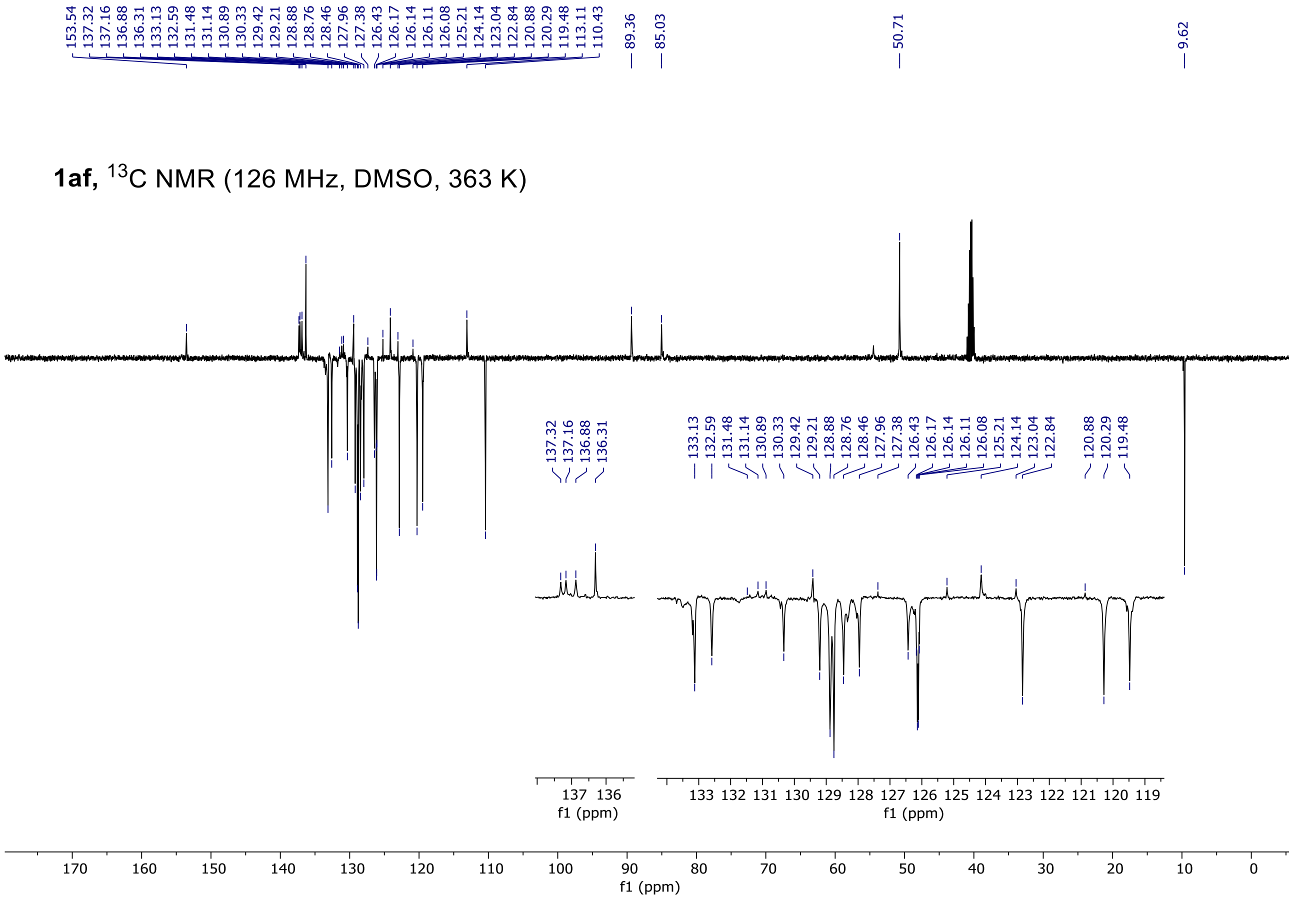

1af, ^{13}C NMR (126 MHz, DMSO, 363 K)

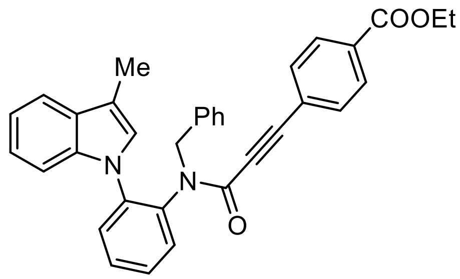

1ag, ^{1}H NMR (500 MHz, DMSO, 363 K)

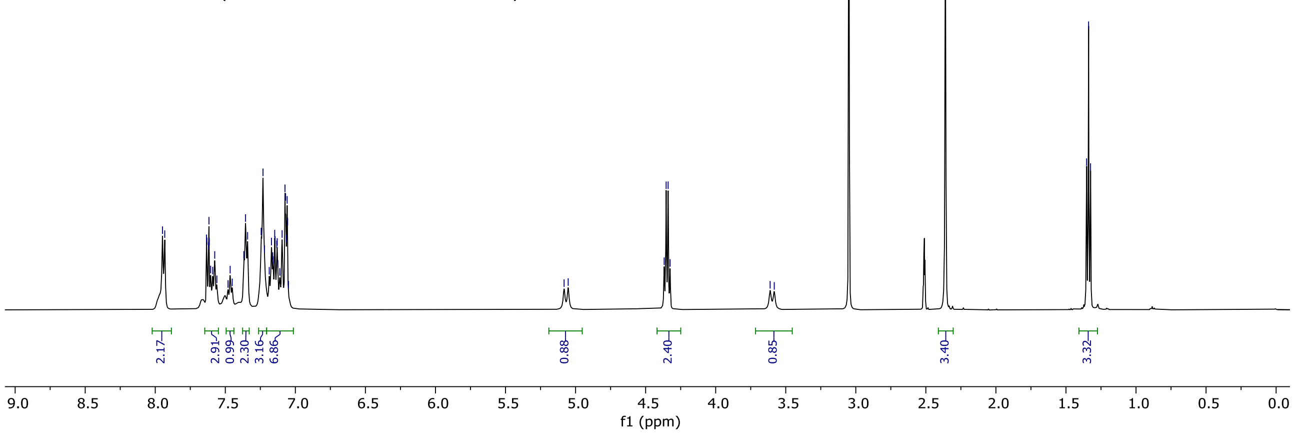

1ag, ^{13}C NMR (126 MHz, DMSO, 363 K)

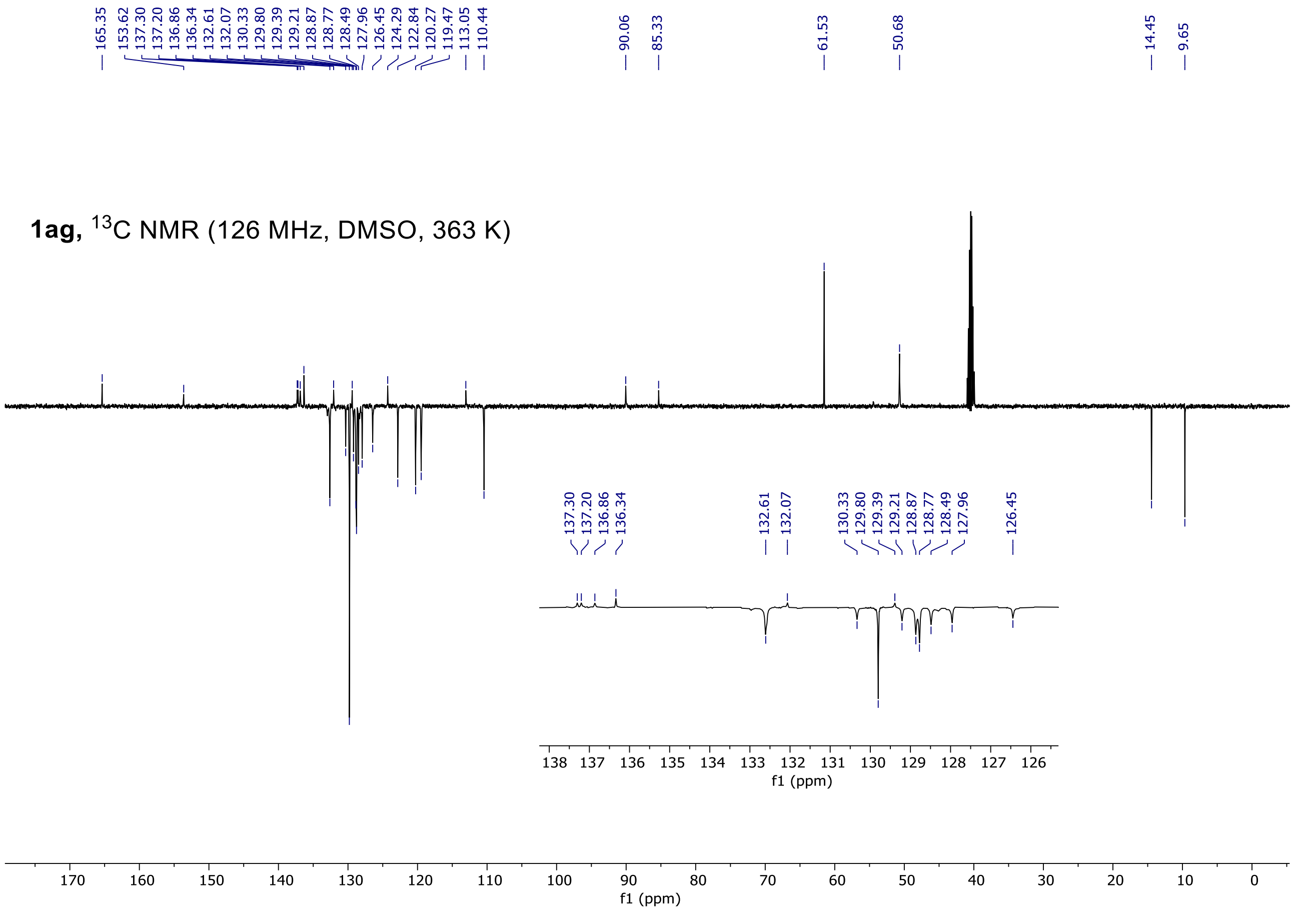

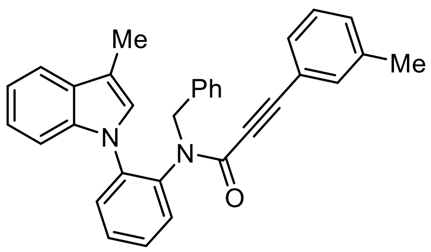

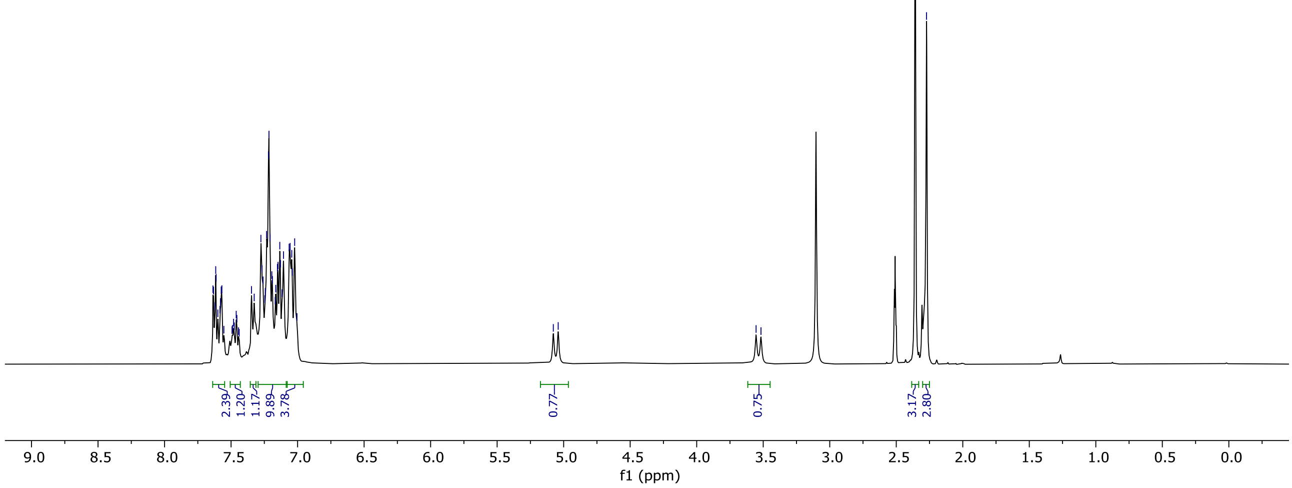

1ah, ^{1}H NMR (400 MHz, DMSO, 353 K)

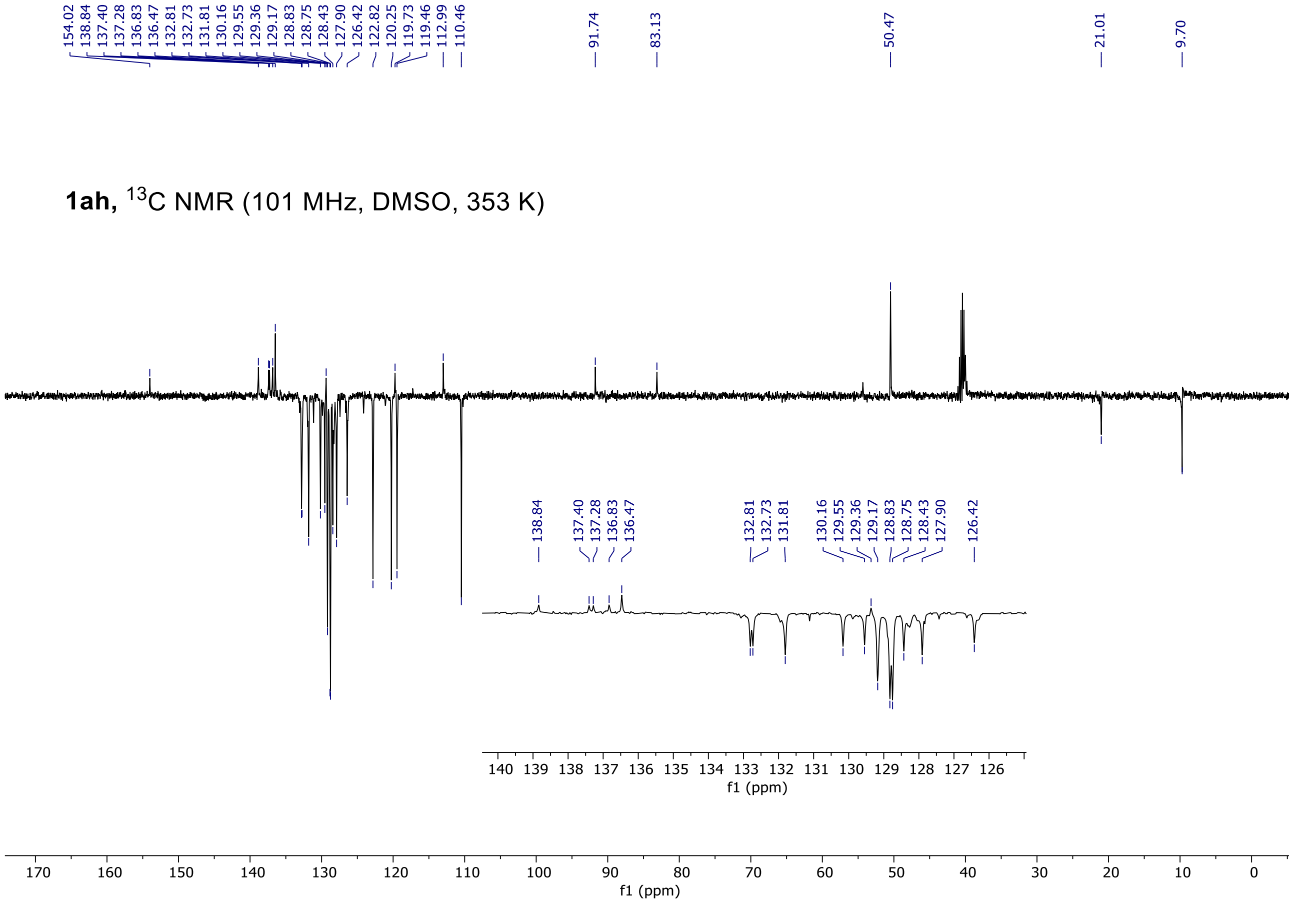

1ah, ^{13}C NMR (101 MHz, DMSO, 353 K)

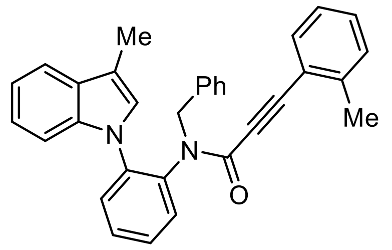

1ai, ^{1}H NMR (500 MHz, DMSO, 363 K)

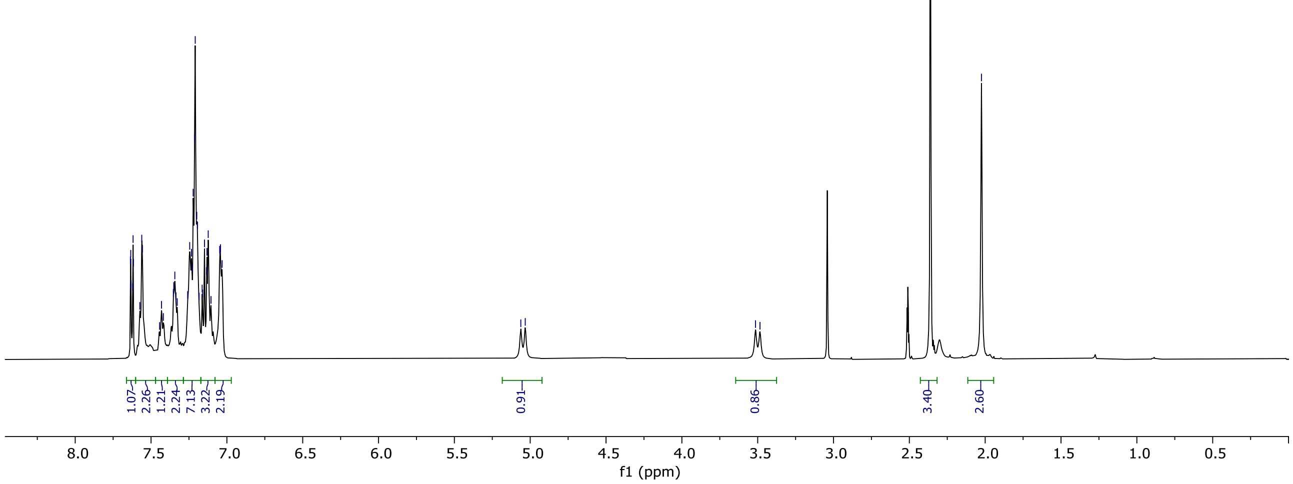

1ai, ^{13}C NMR (126 MHz, DMSO, 363 K)

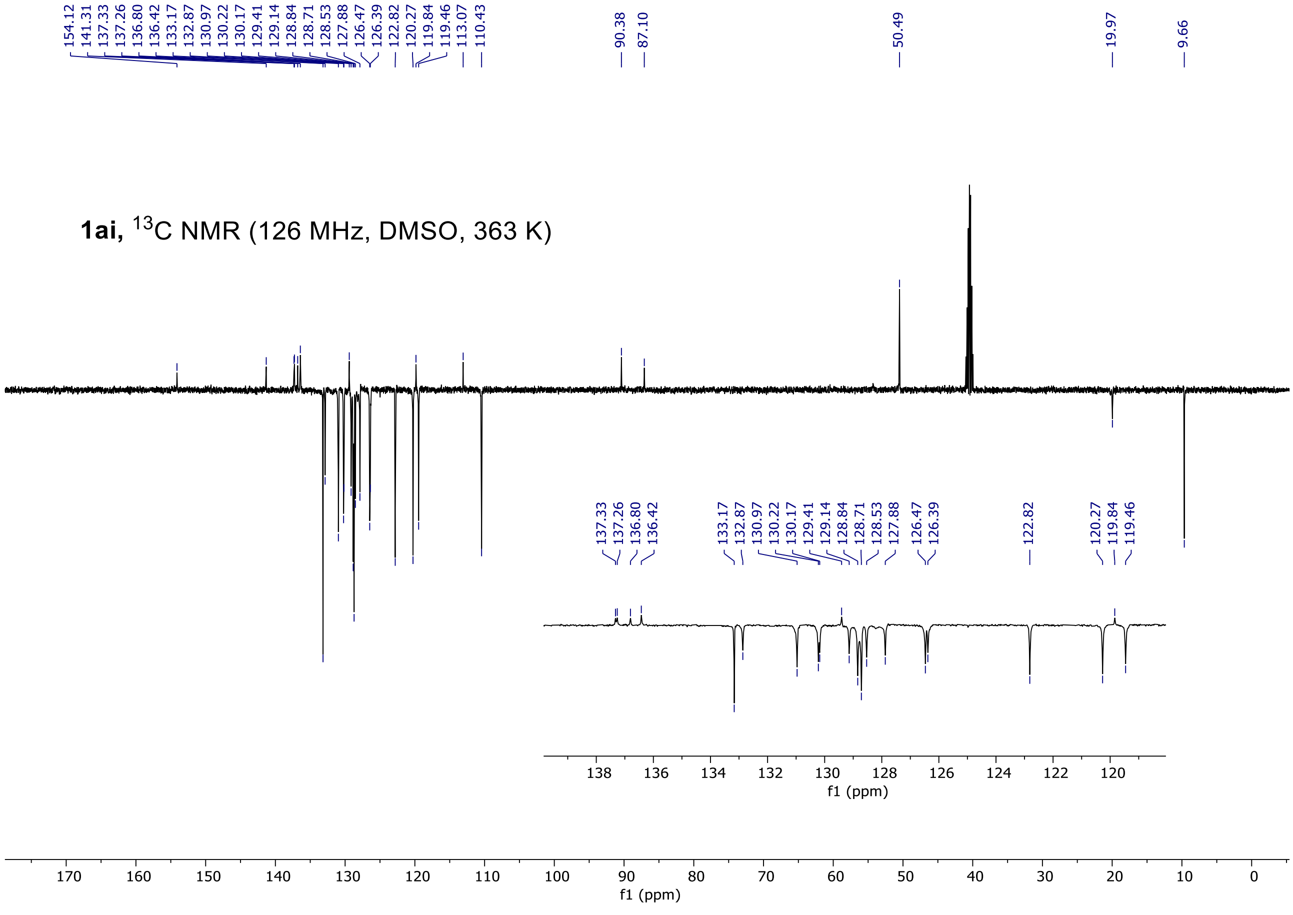

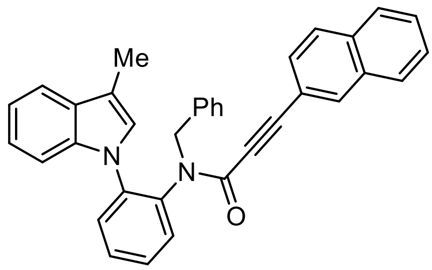

1aj, ^{1}H NMR (500 MHz, DMSO, 363 K)

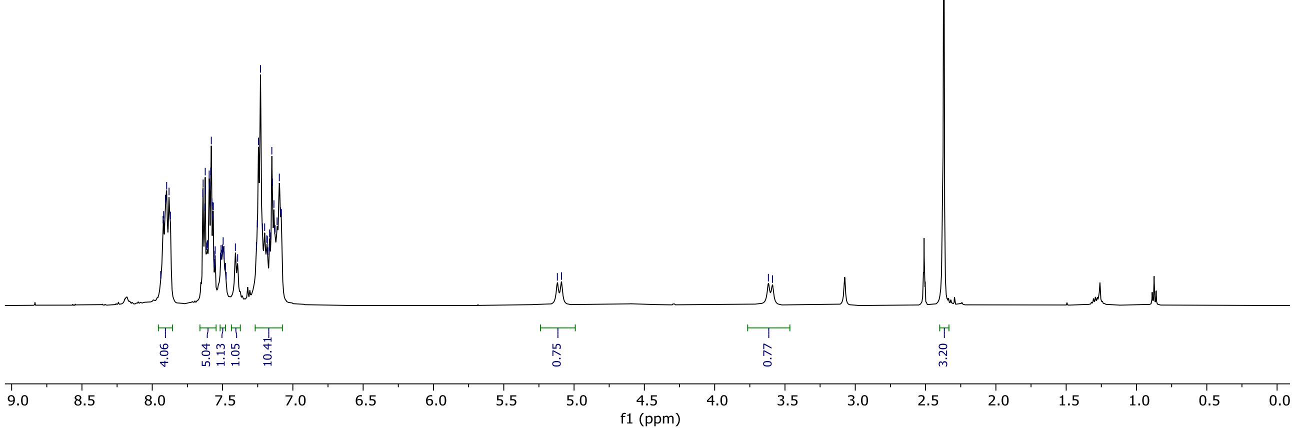

1aj, ^{13}C NMR (126 MHz, DMSO, 363 K)

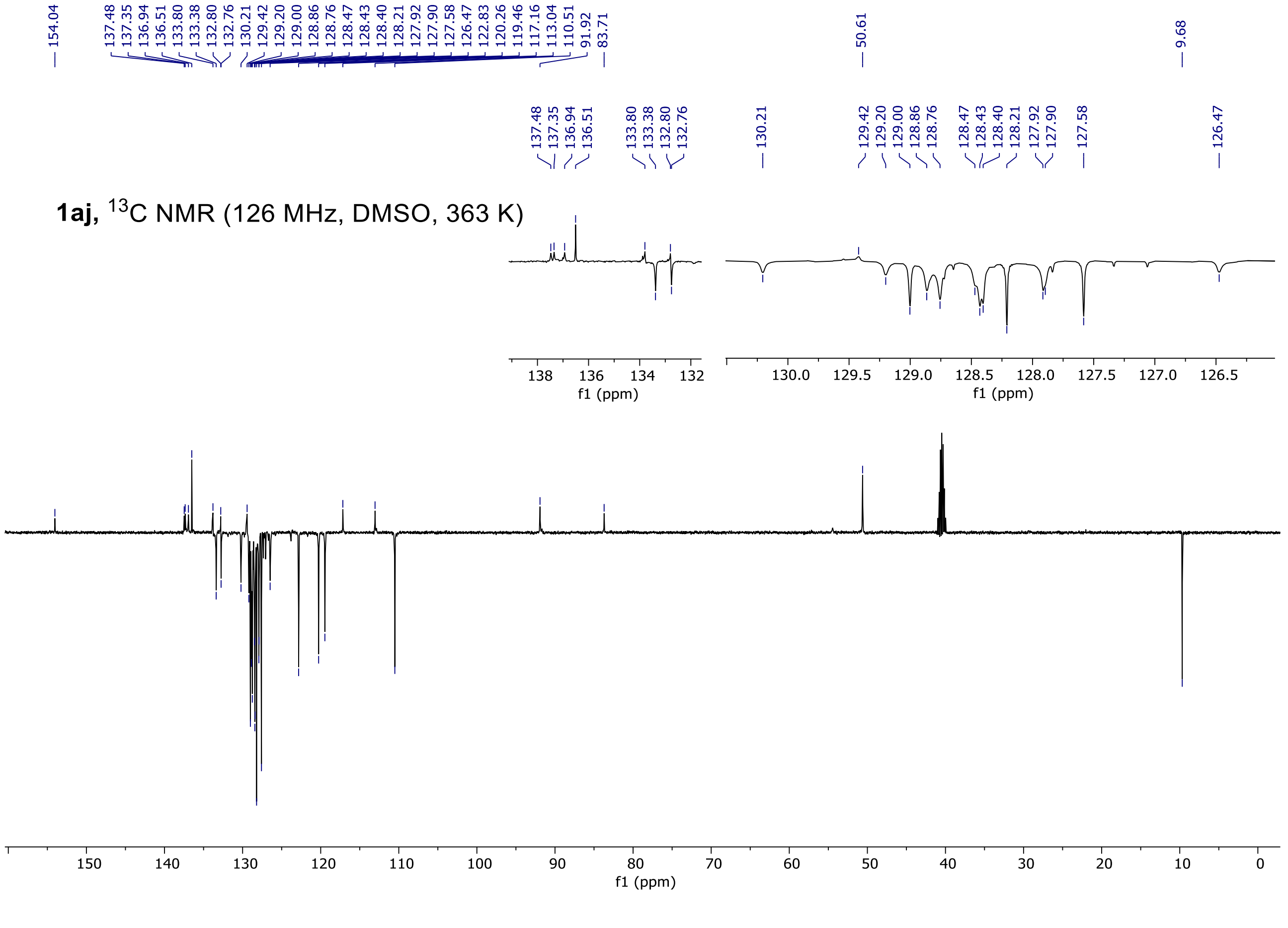

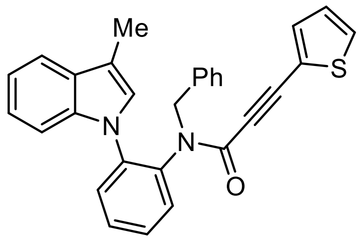

1ak, ^{1}H NMR (400 MHz, DMSO, 363 K)

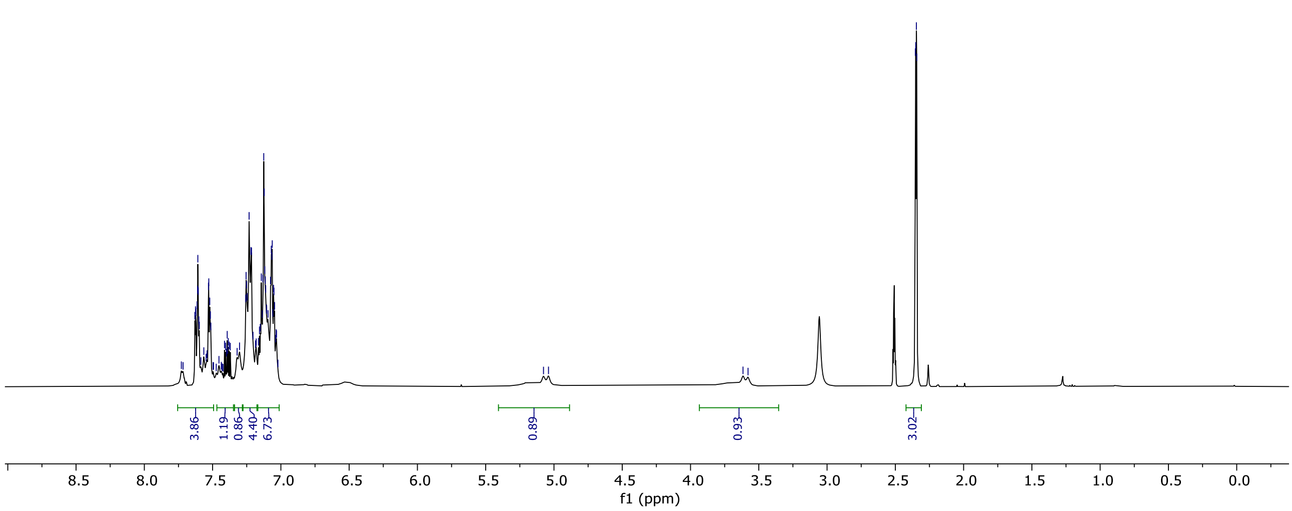

1ak, ^{13}C NMR (101 MHz, DMSO, 363 K)

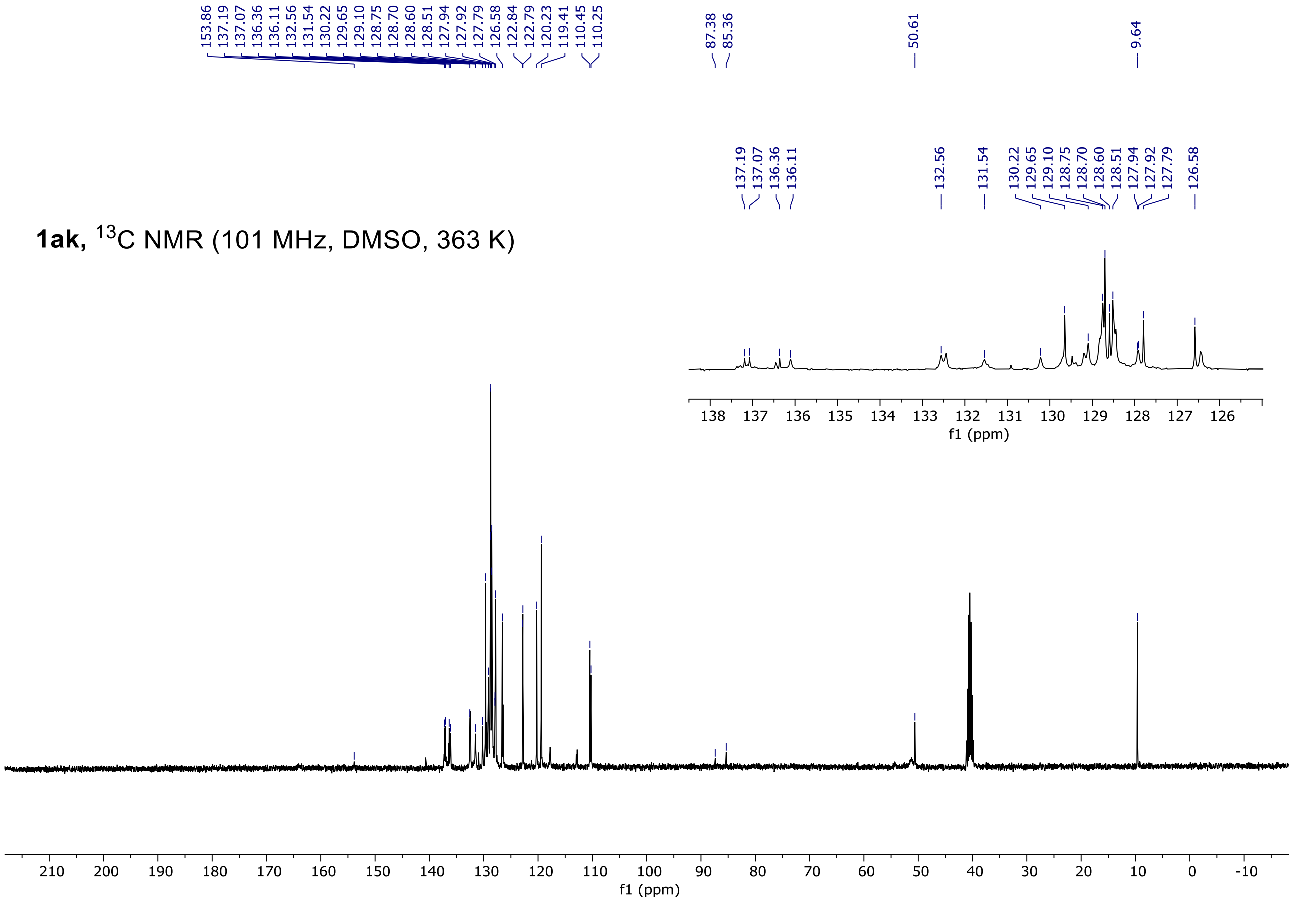

1ak, HSQC (DMSO, 363 K)

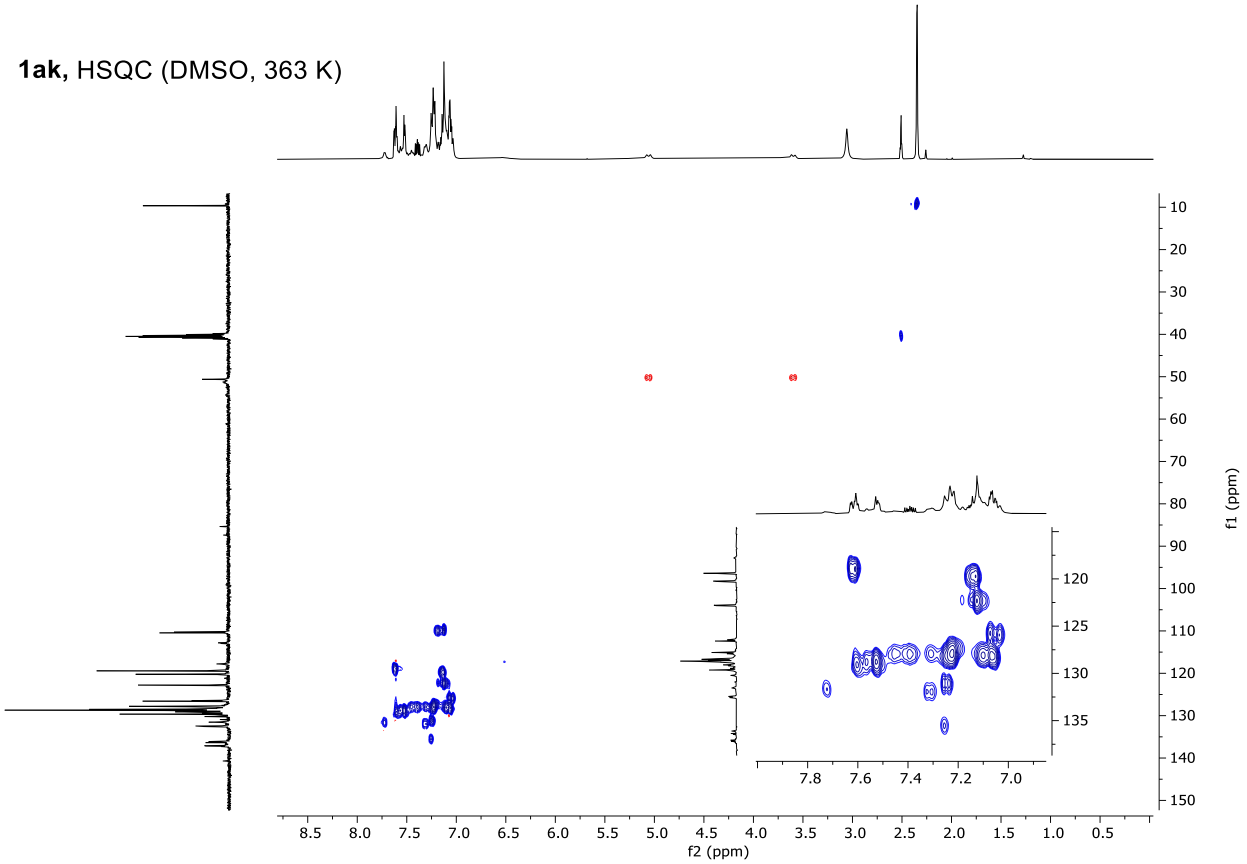

7.67 7.66 7.65 7.64 7.61 7.60 7.59 7.58 7.53 7.52 7.48 7.47 7.46 7.45 7.43 7.40 7.38 7.36 7.34 7.23 7.21 7.20 7.17 7.16 7.15 7.14 7.14 7.12 7.11 7.09 7.05 7.05 7.03 7.03 7.02 5.06 5.03 3.52 3.48 2.84 2.82 2.80 2.78 1.35 1.33 1.31

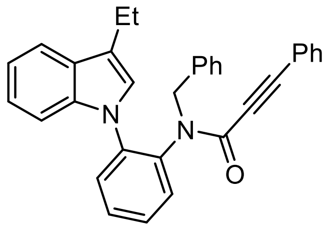

1an, ^{1}H NMR (400 MHz, DMSO, 353 K)

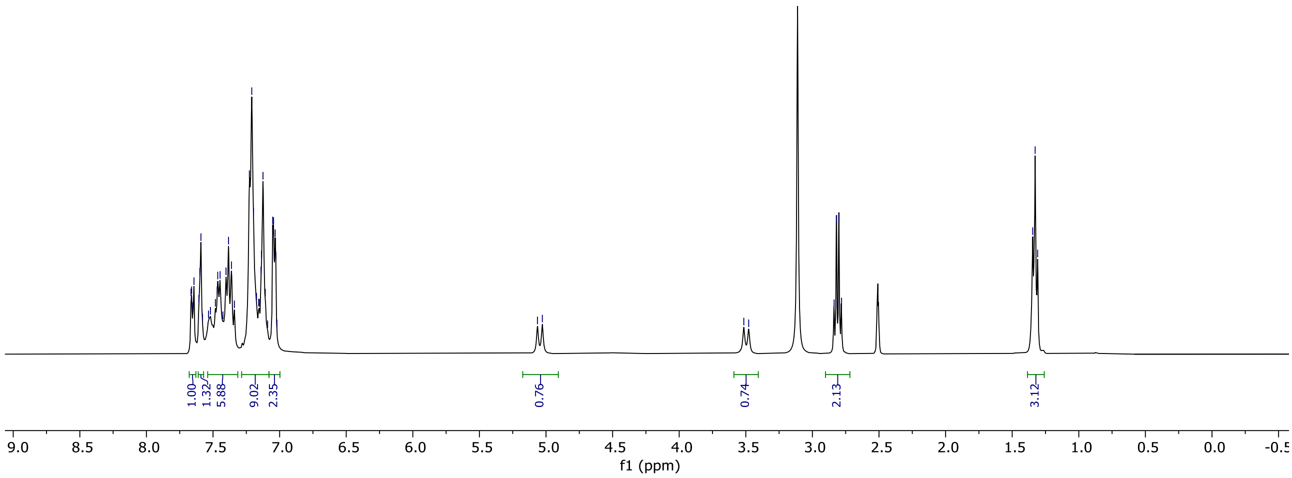

153.96 137.39 136.85 136.42 132.72 132.43 131.04 130.20 129.32 129.10 128.79 128.75 128.46 128.42 127.89 125.42 122.84 120.26 120.16 119.86 119.53 110.51 91.42 83.32 50.44 18.11 14.80

1an, ^{13}C NMR (101 MHz, DMSO, 353 K)

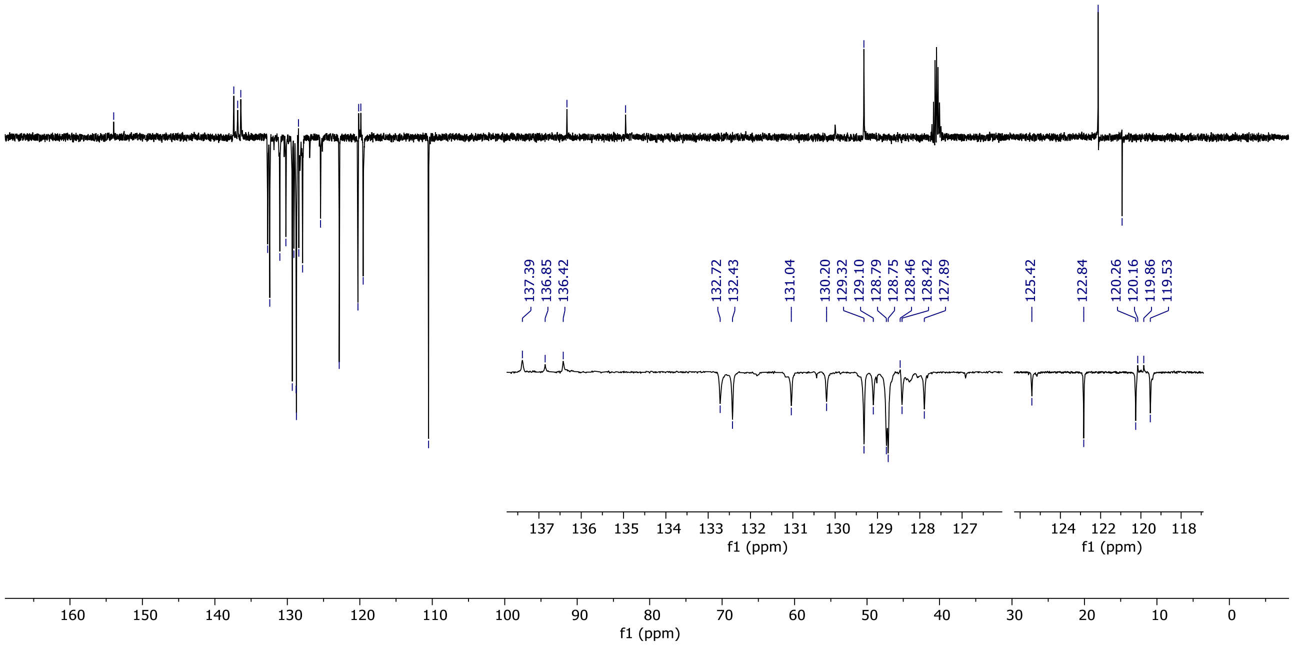

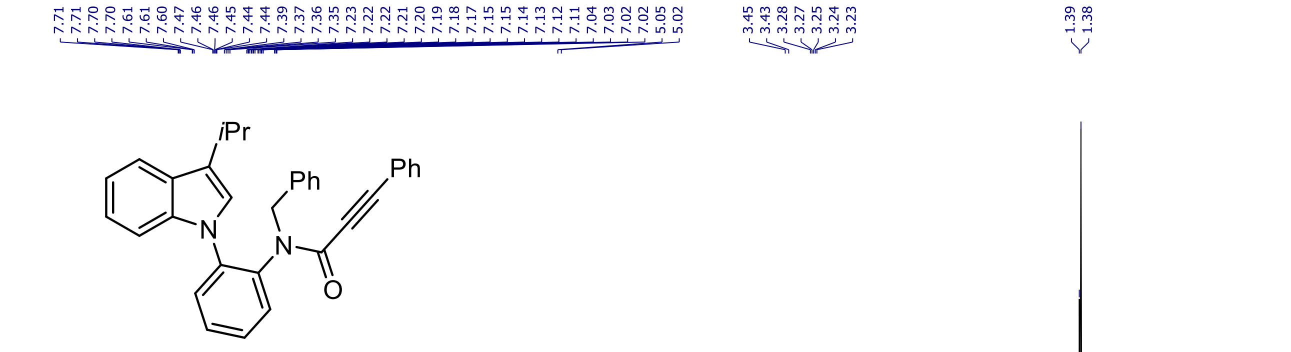

1ao, ^{1}H NMR (600 MHz, DMSO, 353 K)

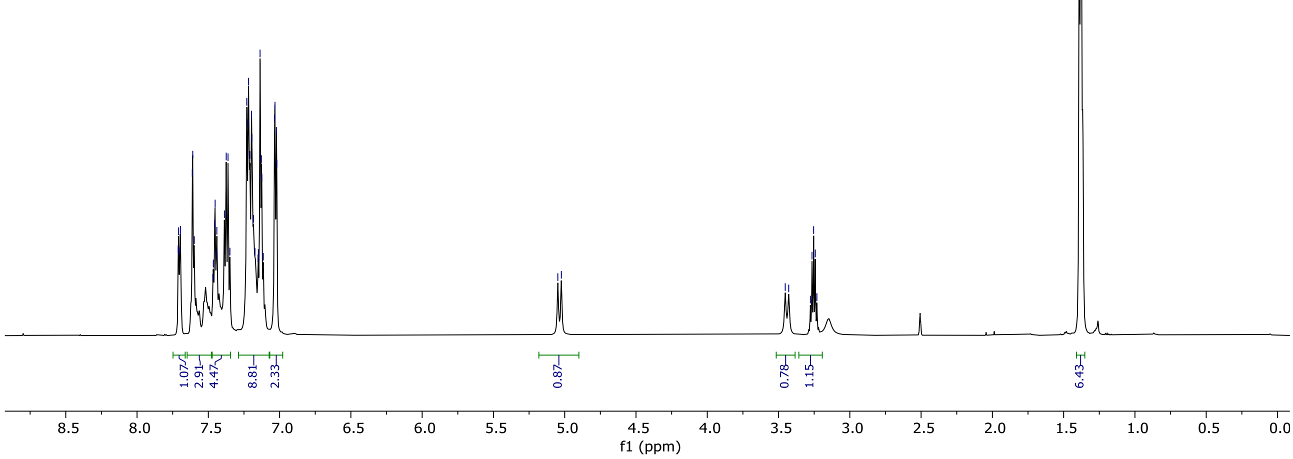

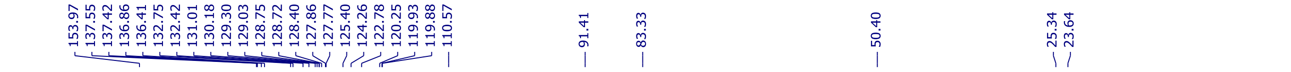

1ao, ^{13}C NMR (151 MHz, DMSO, 353 K)

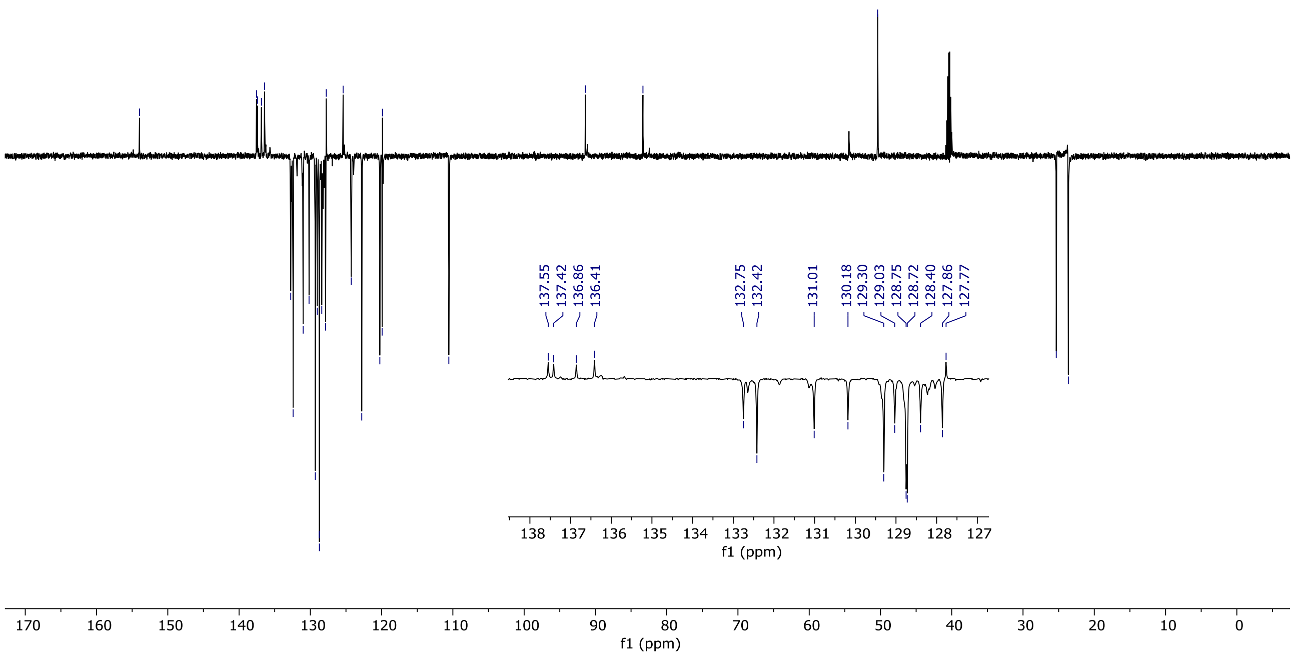

1ao, HSQC ($CDCl_3$, 353 K)

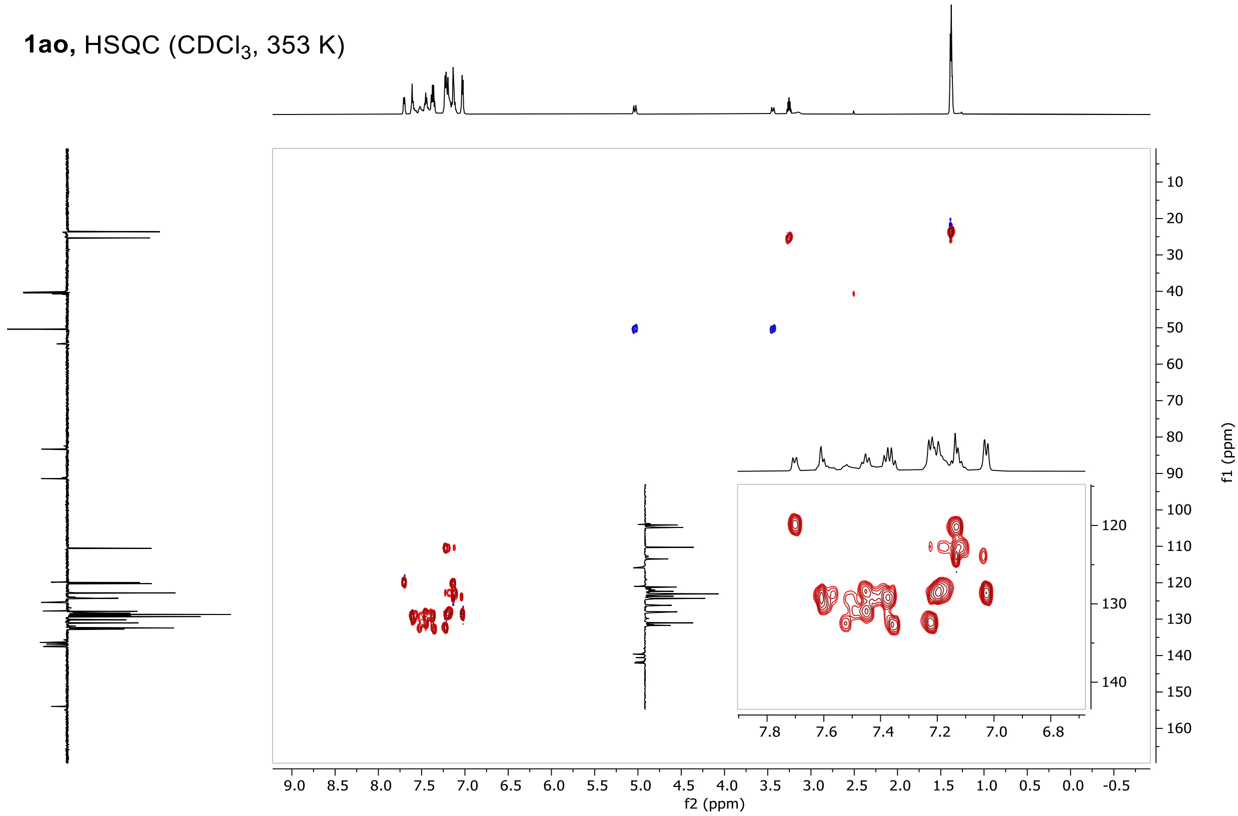

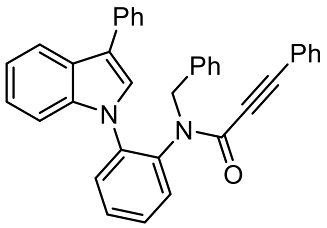

1ap, ^{1}H NMR (400 MHz, DMSO, 353 K)

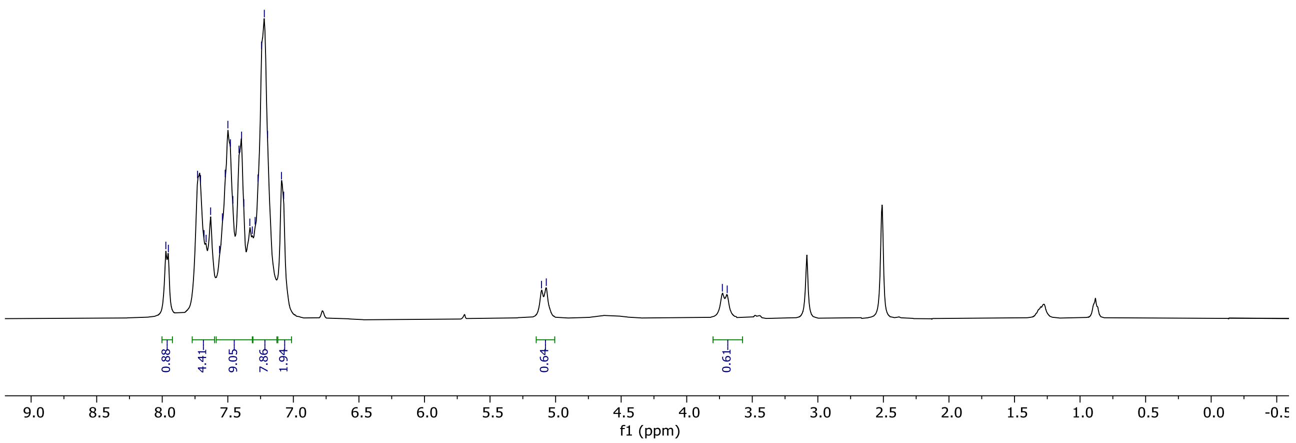

1ap, ^{13}C NMR (101 MHz, DMSO, 353 K)

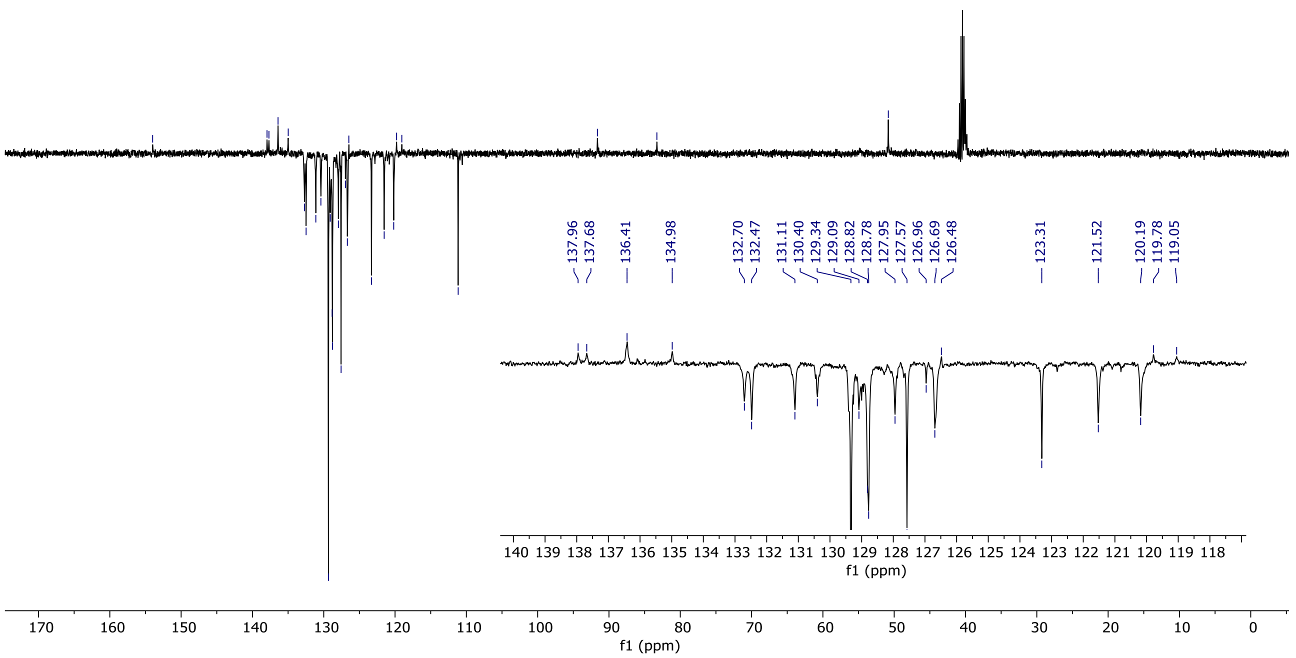

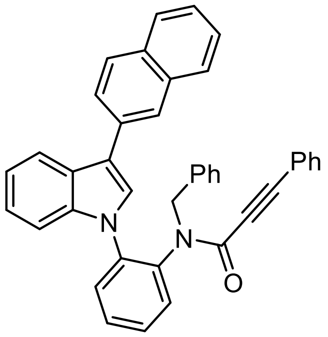

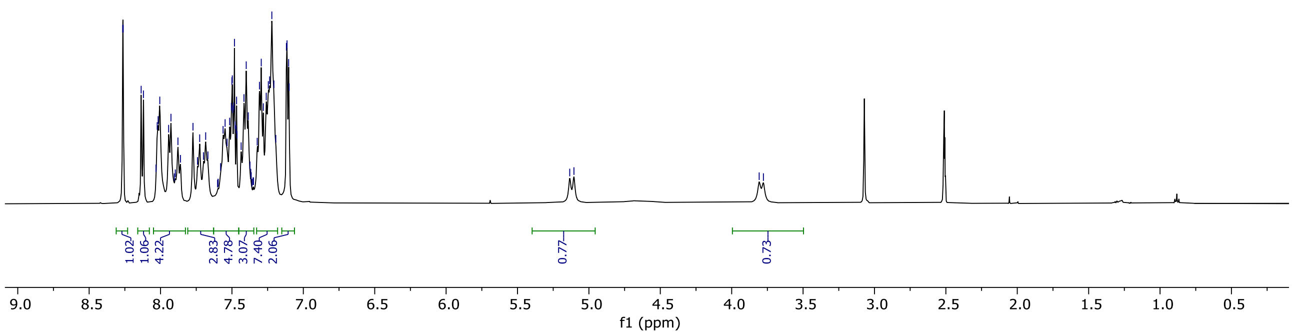

1aq, 1H NMR (500 MHz, DMSO, 363 K)

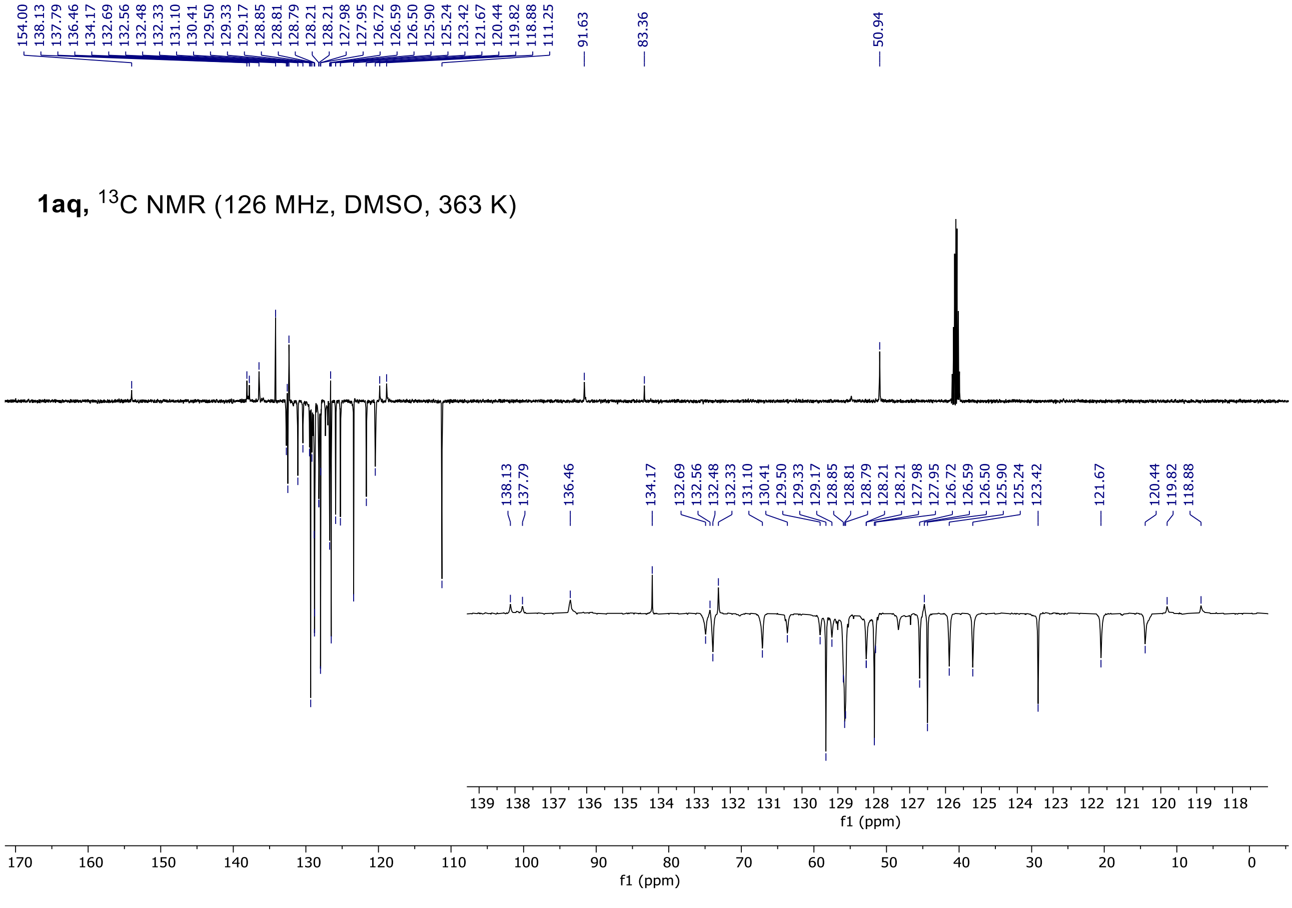

1aq, ^{13}C NMR (126 MHz, DMSO, 363 K)

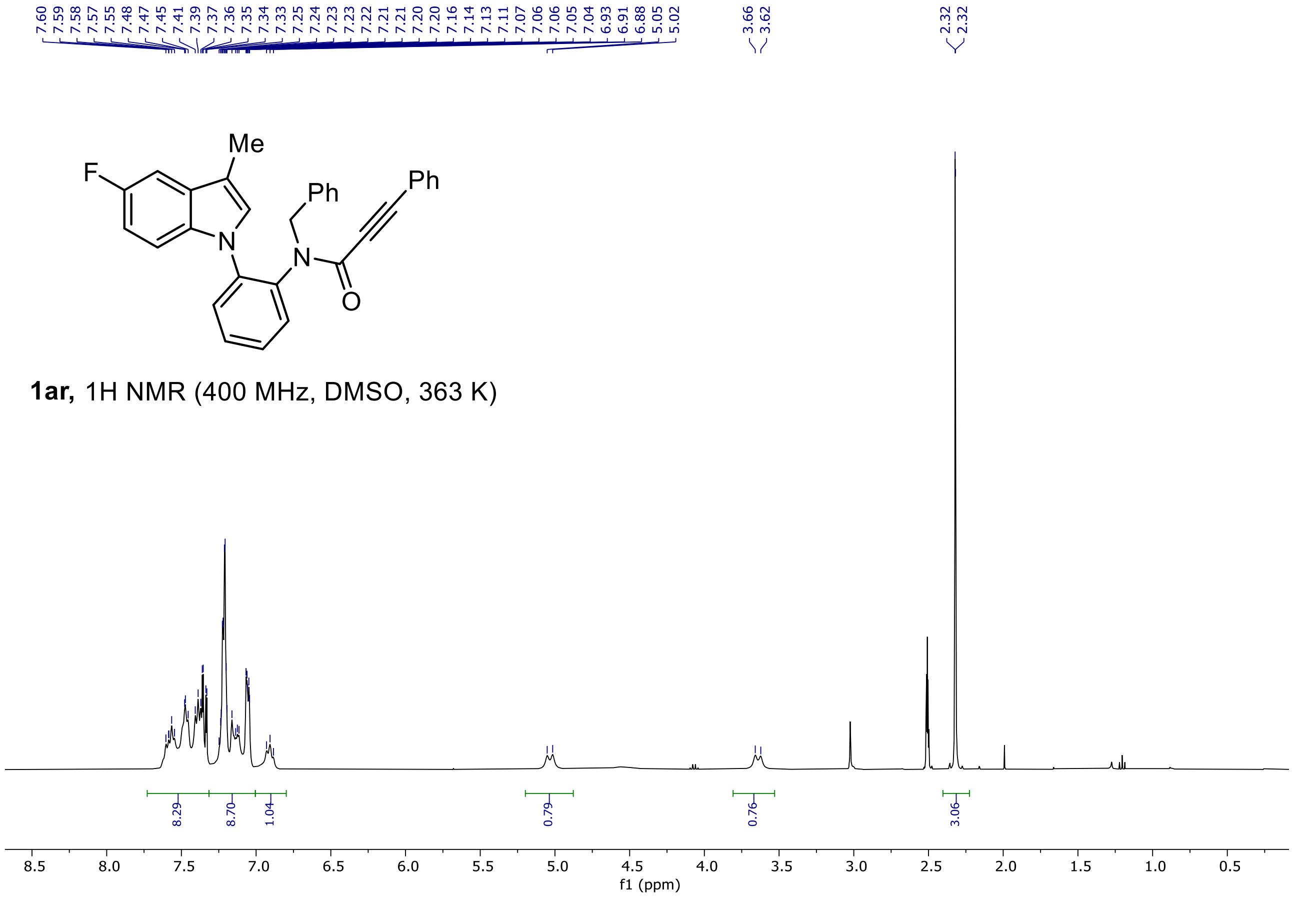

1ar, 1H NMR (400 MHz, DMSO, 363 K)

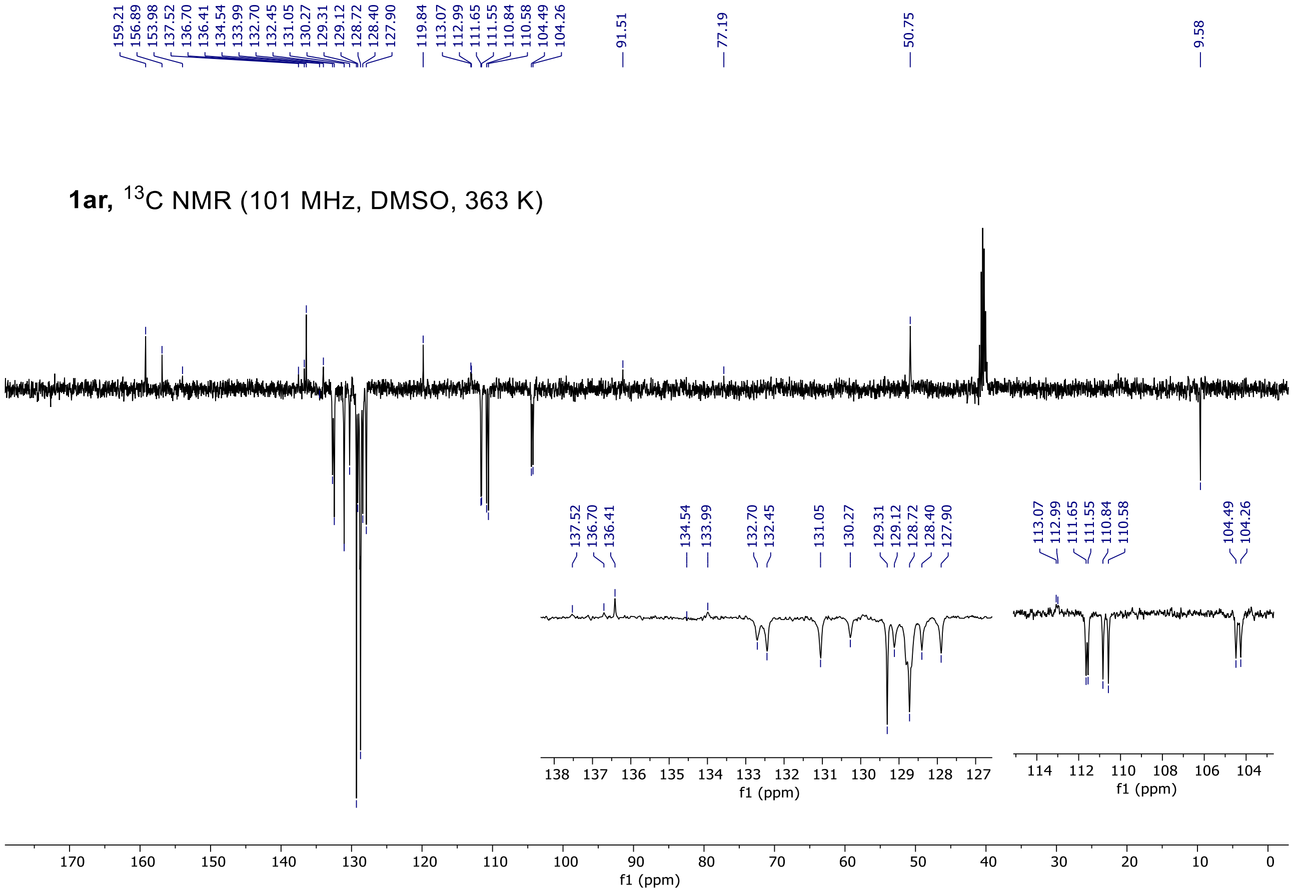

1ar, ^{13}C NMR (101 MHz, DMSO, 363 K)

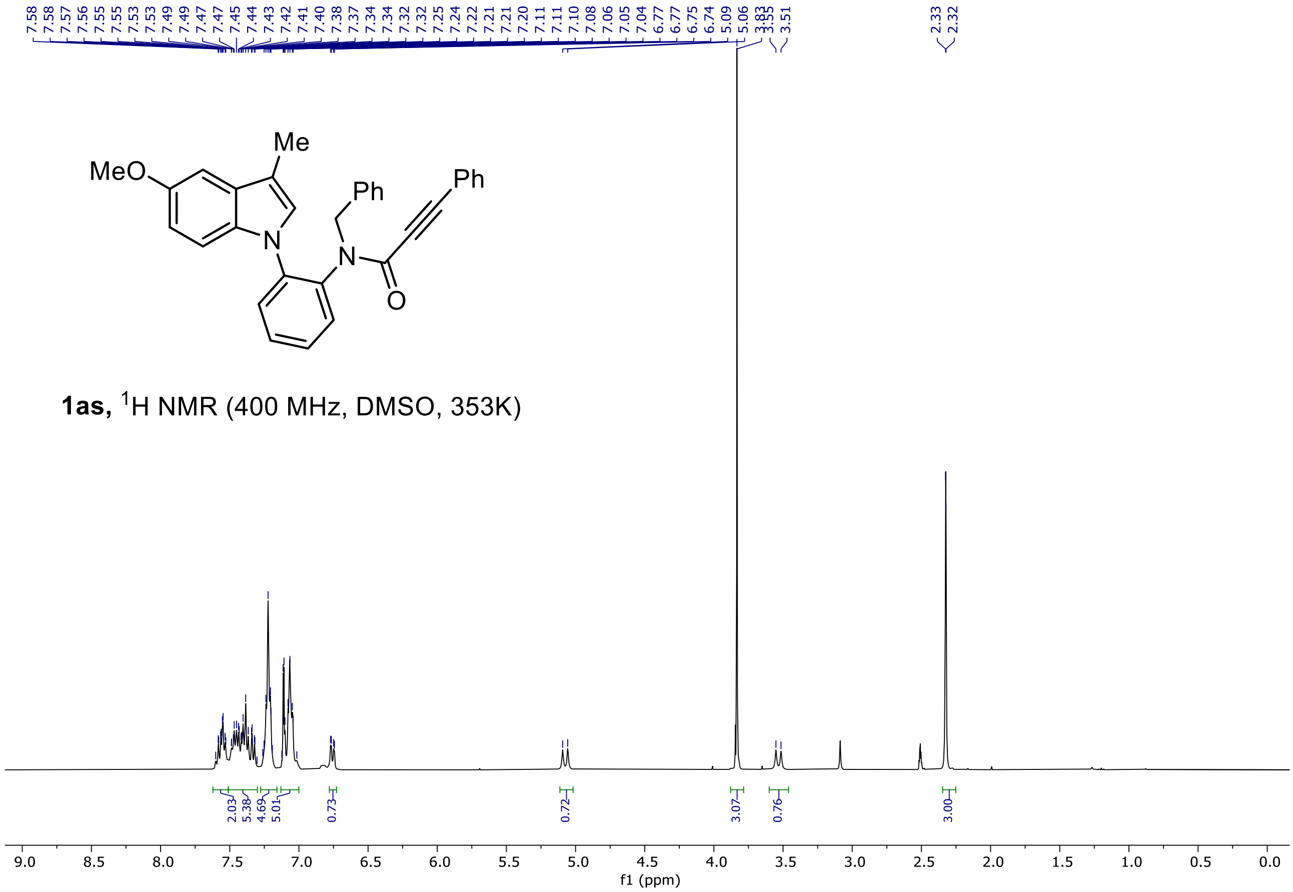

1as, ^{1}H NMR (400 MHz, DMSO, 353K)

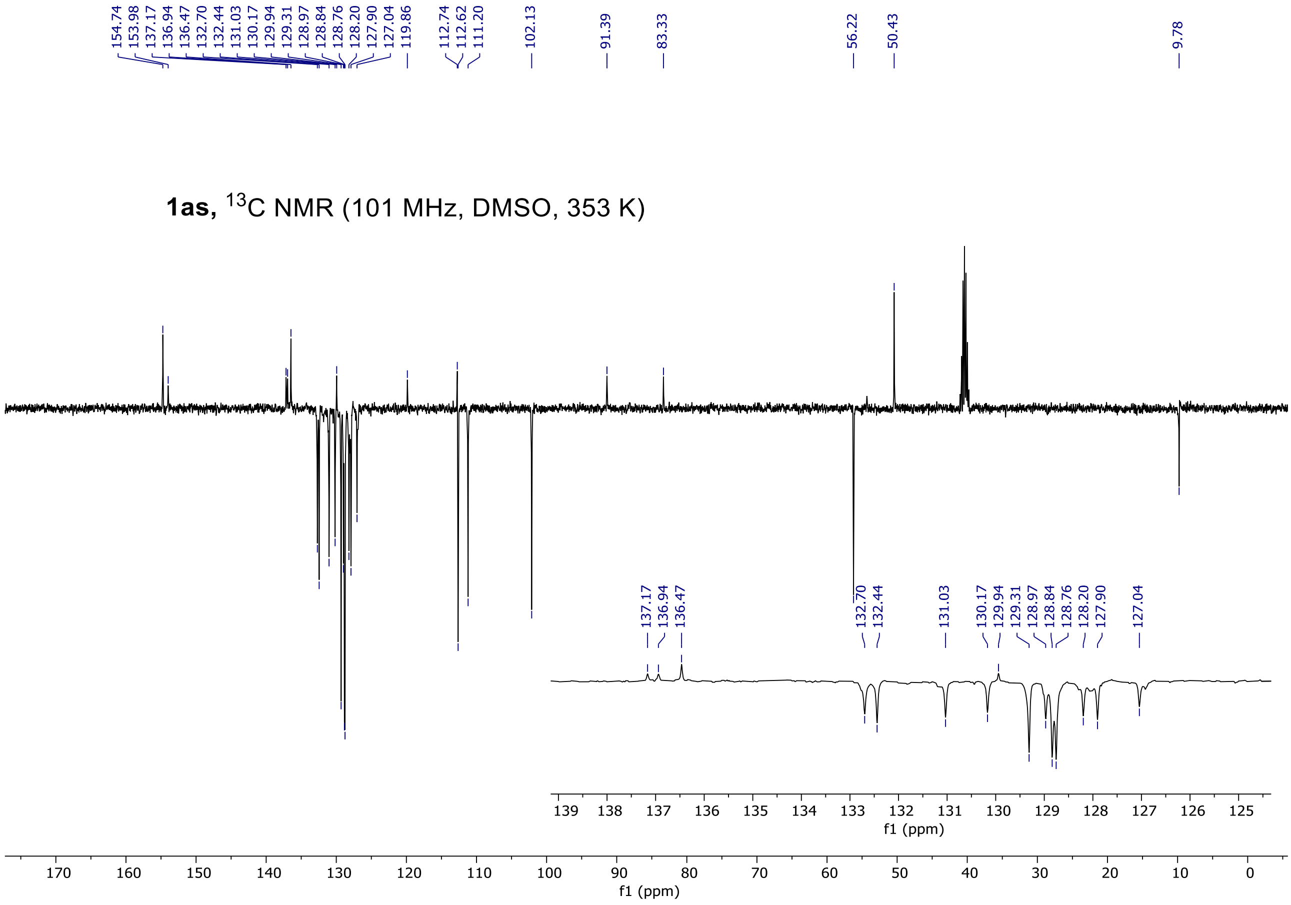

1as, ^{13}C NMR (101 MHz, DMSO, 353 K)

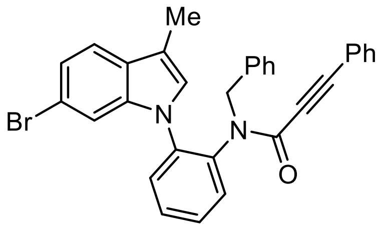

1at, ^{1}H NMR (600 MHz, DMSO, 353 K)

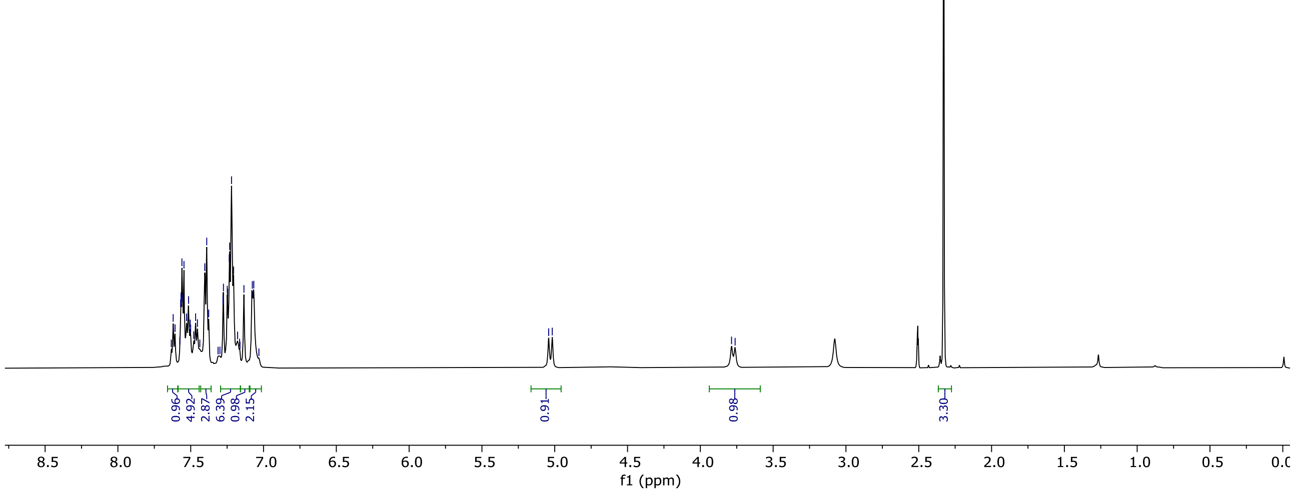

1at, ^{13}C NMR (151 MHz, DMSO, 353 K)

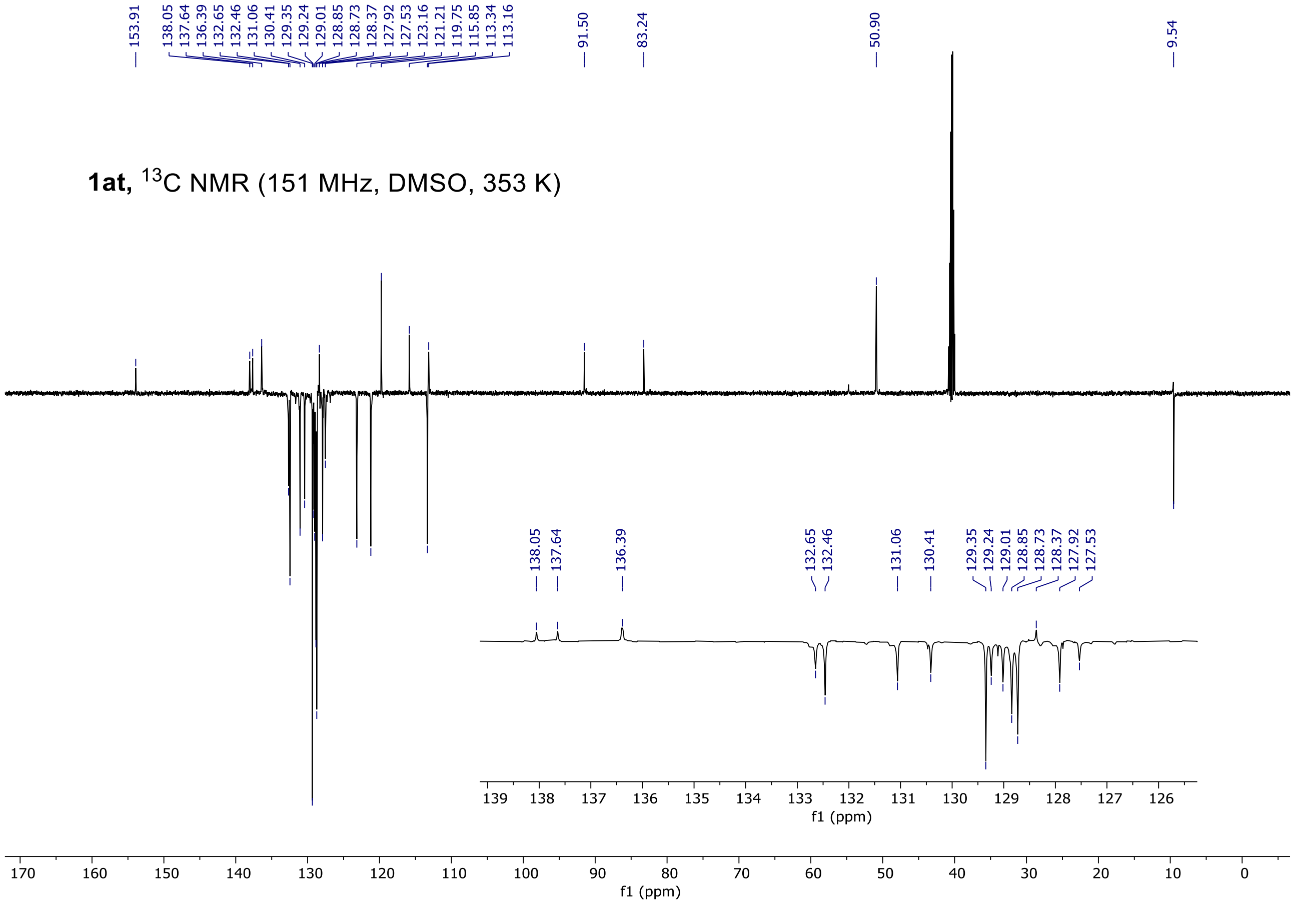

1at, HSQC (DMSO, 353 K)

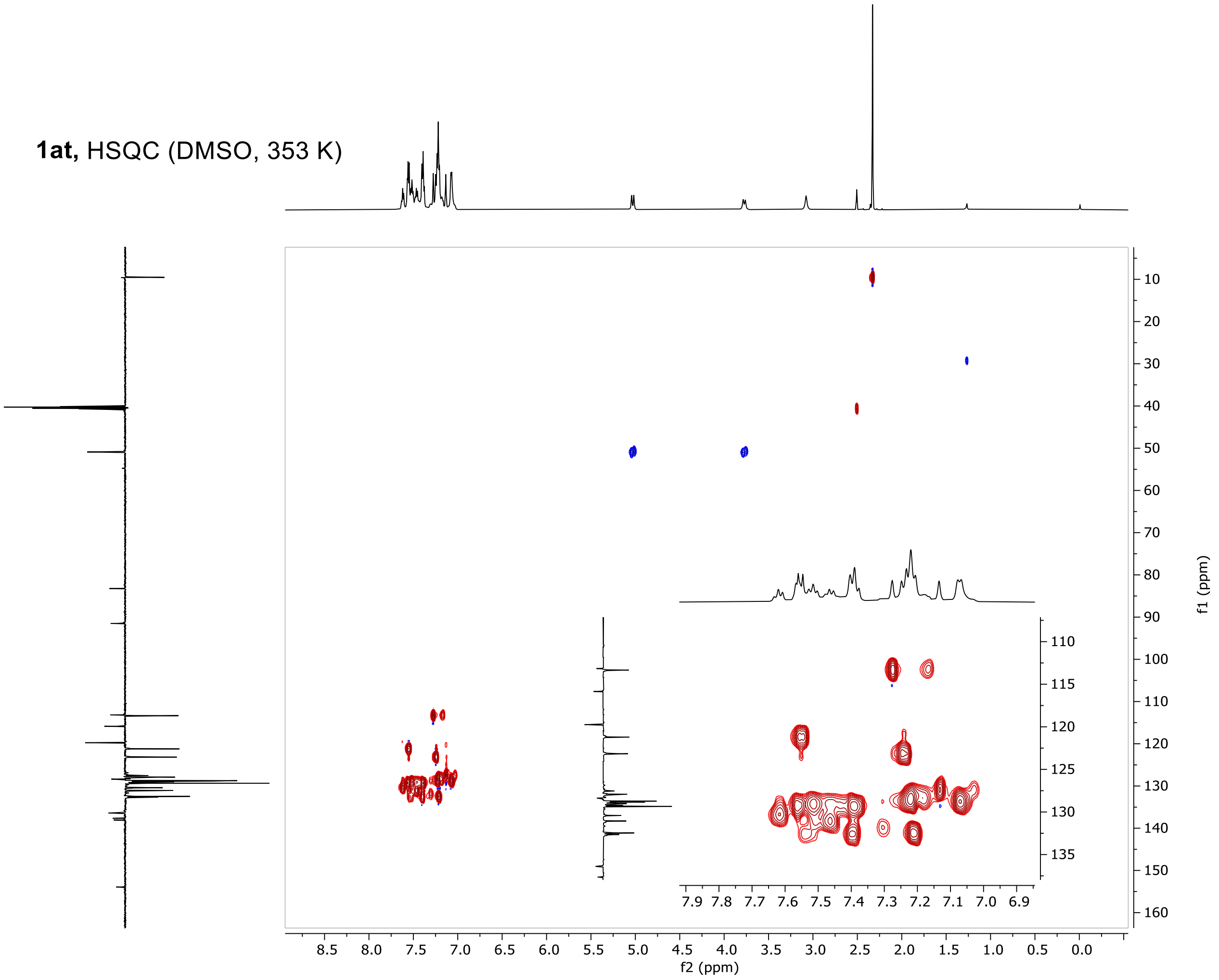

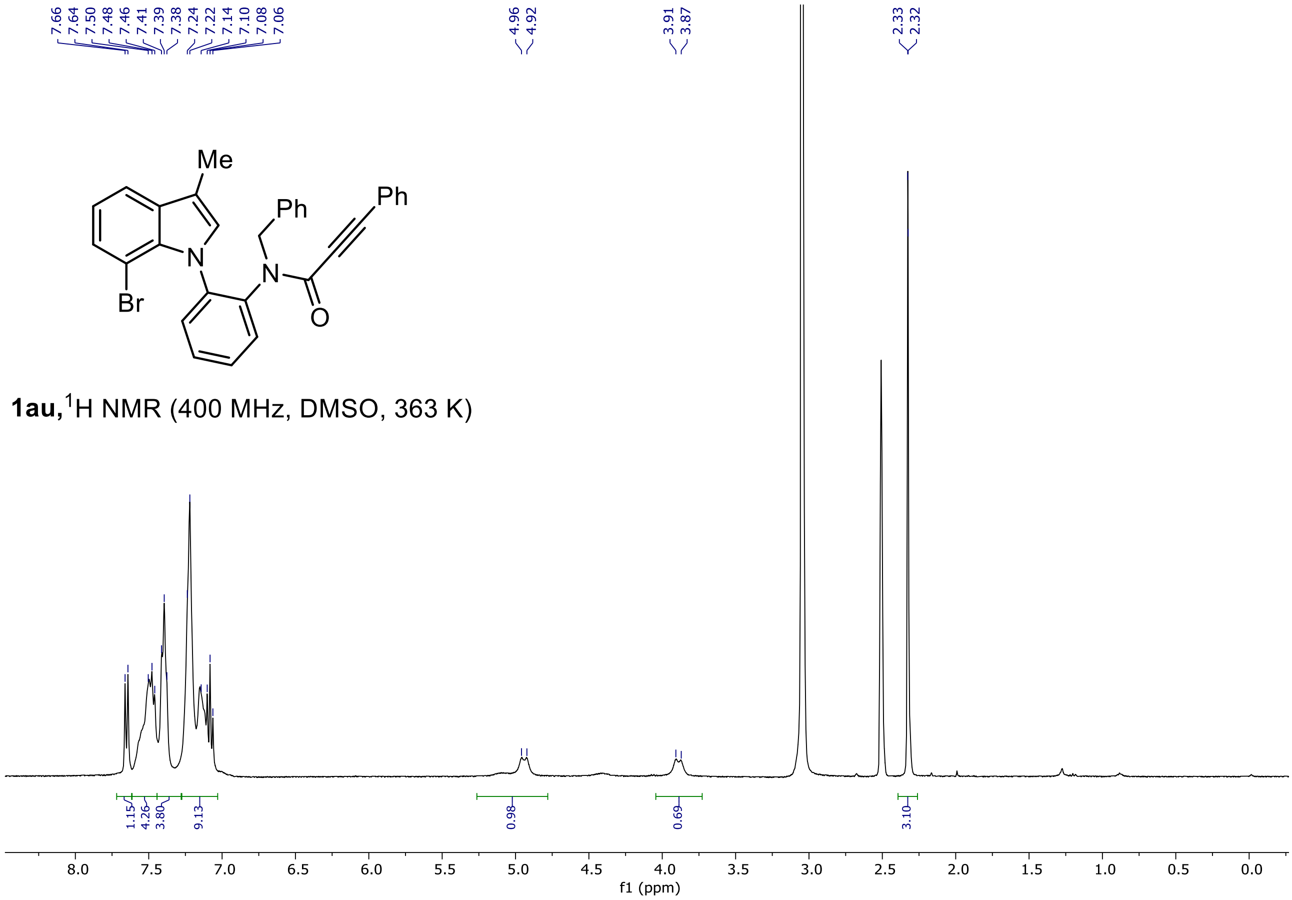
Me
Ph
Ph
N
N
O
Br
1au, 1H NMR (400 MHz, DMSO, 363 K)
7.66 7.64 7.50 7.48 7.46 7.41 7.39 7.38 7.24 7.22 7.14 7.10 7.08 7.06
4.96 4.92
3.91 3.87
2.33 2.32
1.15 4.26 3.80 9.13
0.98
0.69
3.10
8.0 7.5 7.0 6.5 6.0 5.5 5.0 4.5 4.0 3.5 3.0 2.5 2.0 1.5 1.0 0.5 0.0
f1 (ppm)

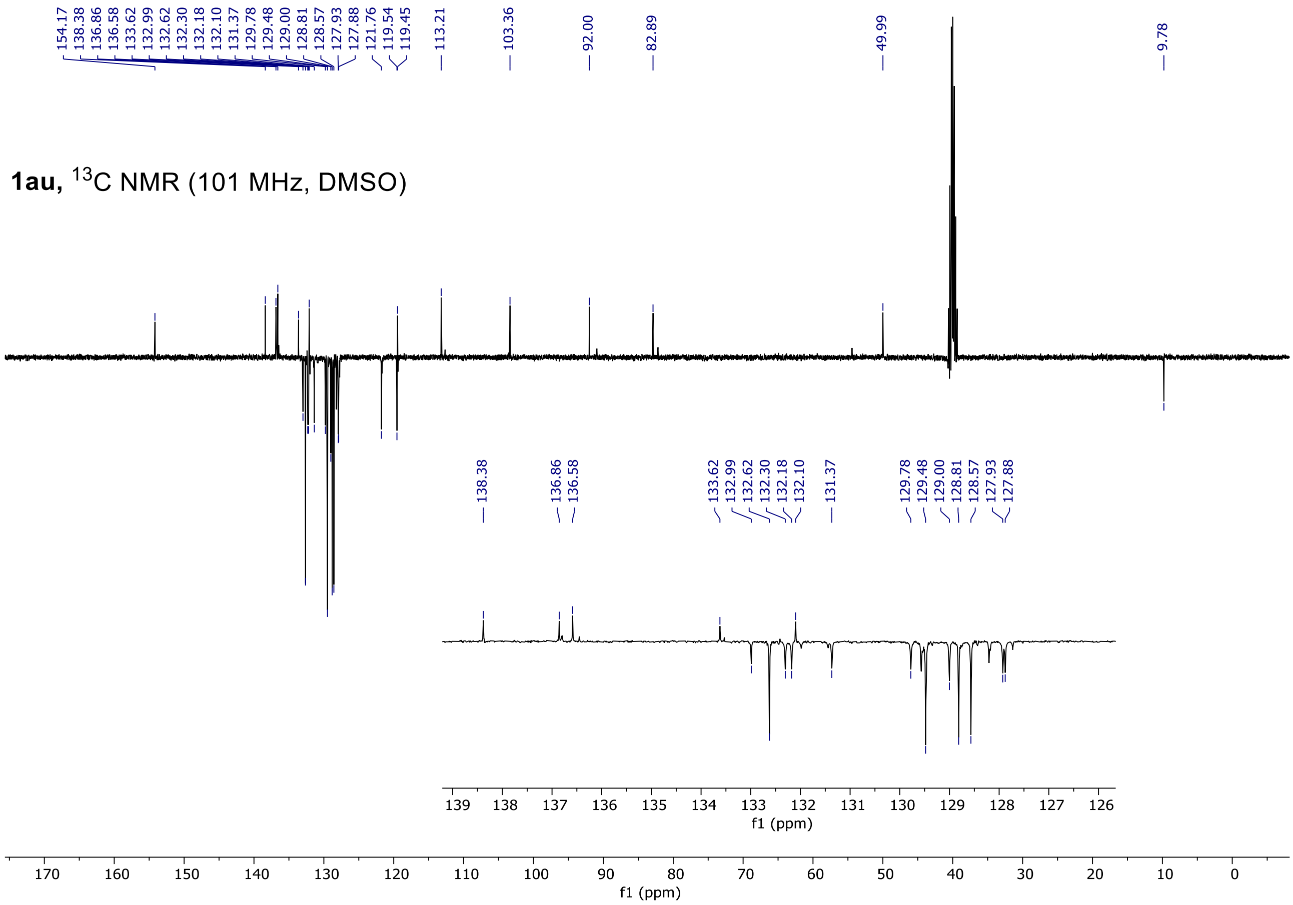
154.17 138.38 136.86 136.58 133.62 132.99 132.62 132.30 132.18 132.10 131.37 129.78 129.48 129.00 128.81 128.57 127.93 127.88 121.76 119.54 119.45
113.21
103.36
92.00
82.89
49.99
9.78
1au, 13C NMR (101 MHz, DMSO)
138.38
136.86 136.58
133.62 132.99 132.62 132.30 132.18 132.10
131.37
129.78 129.48 129.00 128.81 128.57 127.93 127.88
139 138 137 136 135 134 133 132 131 130 129 128 127 126
f1 (ppm)
170 160 150 140 130 120 110 100 90 80 70 60 50 40 30 20 10 0
f1 (ppm)

1au, HSQC (DMSO)

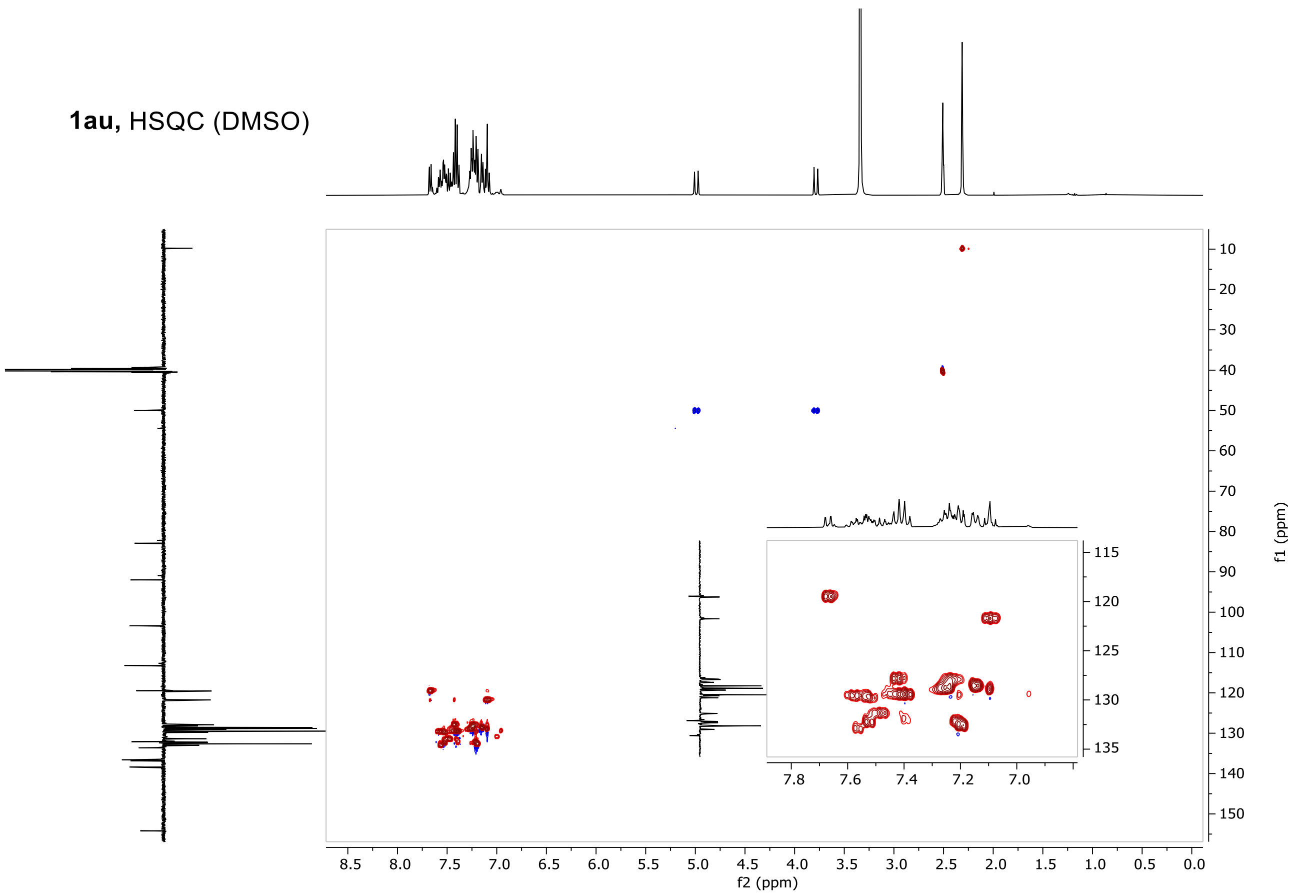

1au, HMBC (DMSO)

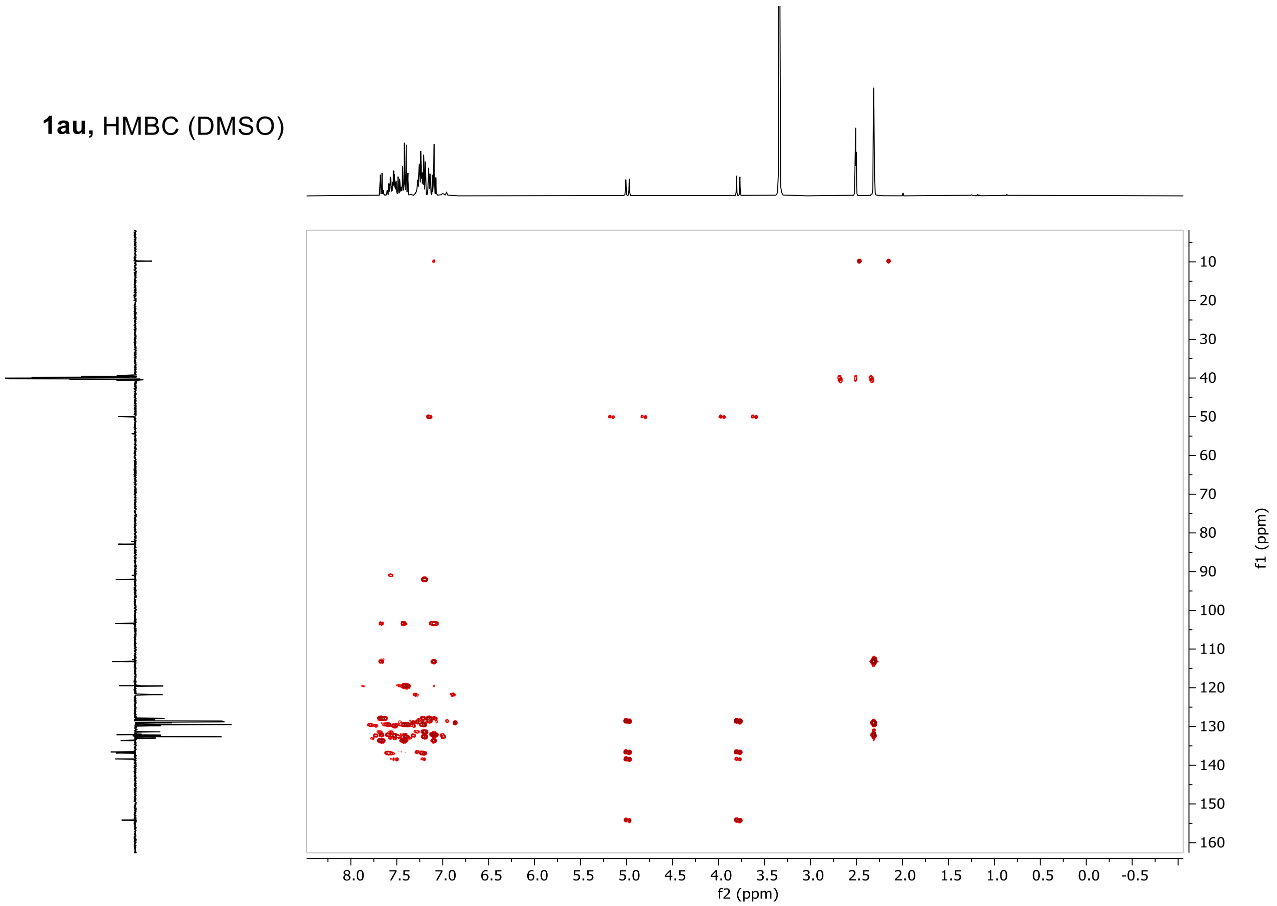

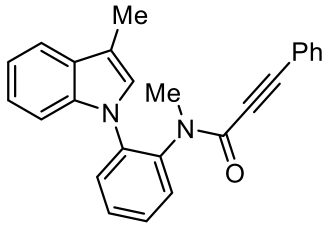

1av, ^{1}H NMR (400 MHz, DMSO, 363 K)

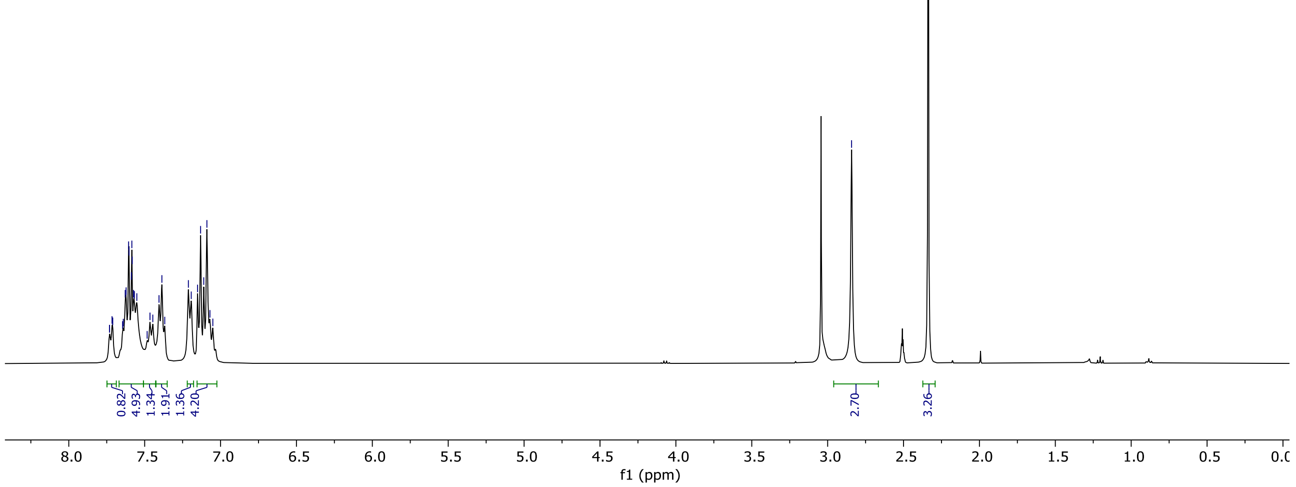

1av, ^{13}C NMR (101 MHz, DMSO, 363 K)

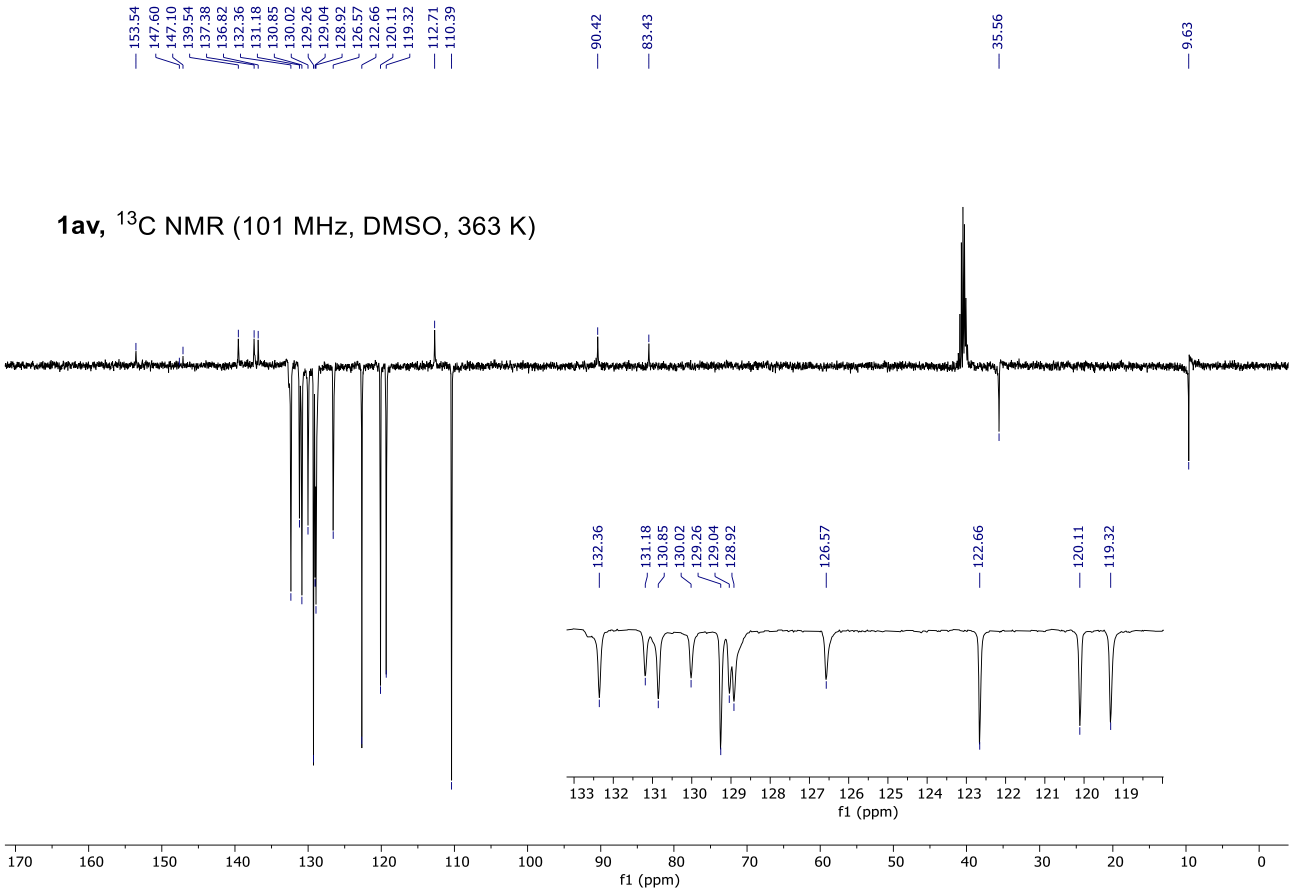

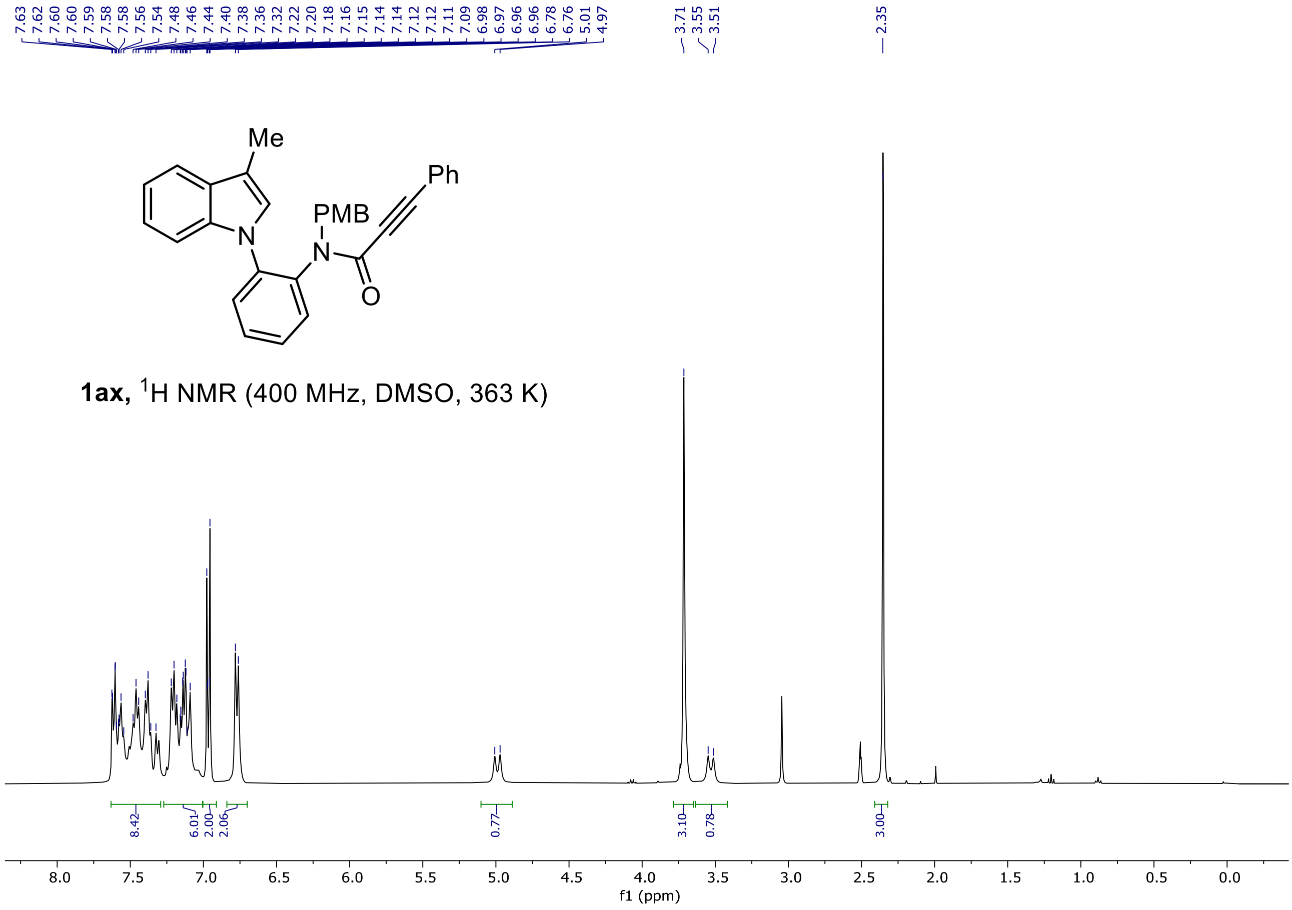

1ax, ^{1}H NMR (400 MHz, DMSO, 363 K)

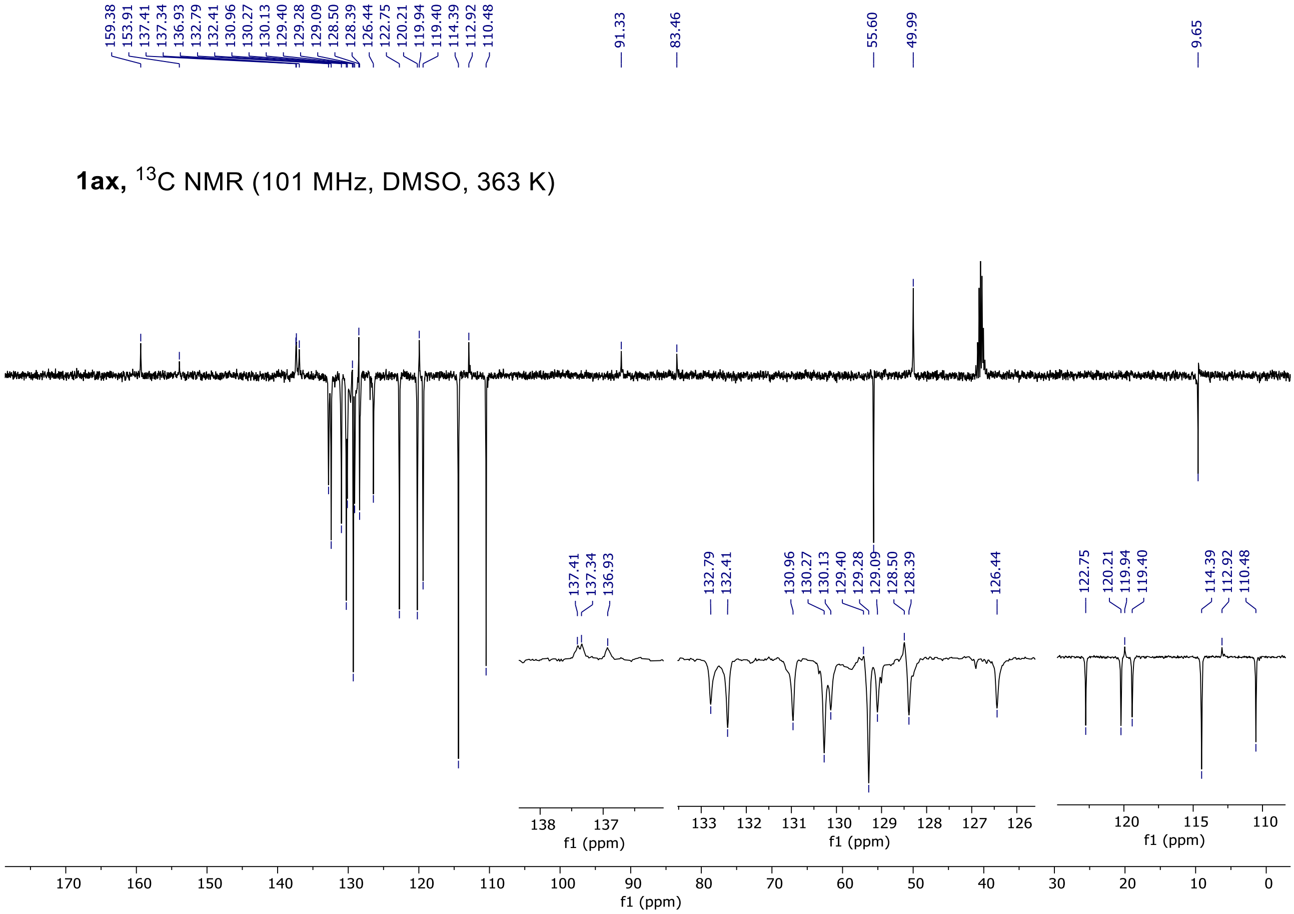

1ax, ^{13}C NMR (101 MHz, DMSO, 363 K)

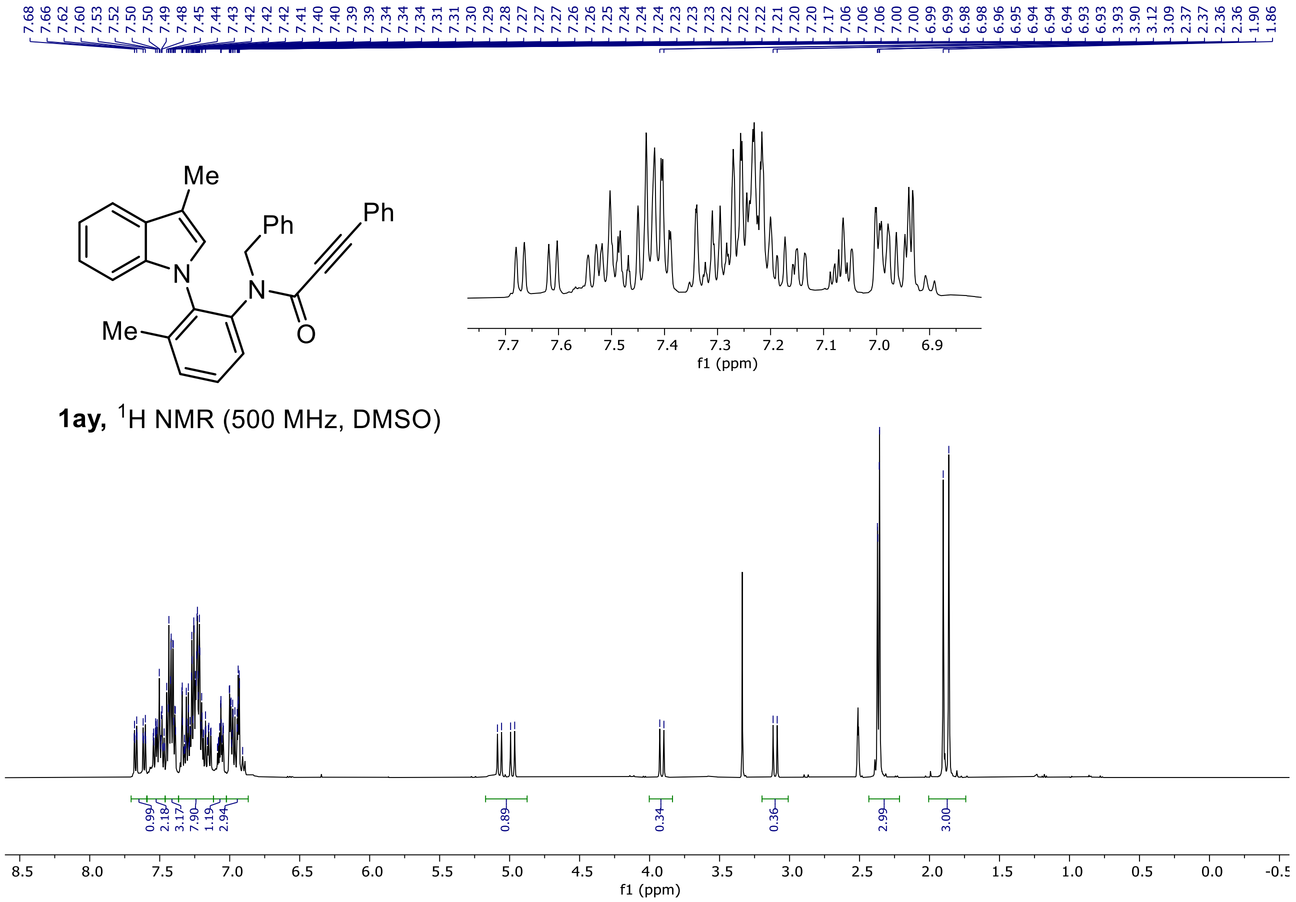

1ay, ^{1}H NMR (500 MHz, DMSO)

1ay, ^{13}C NMR (75 MHz, DMSO)

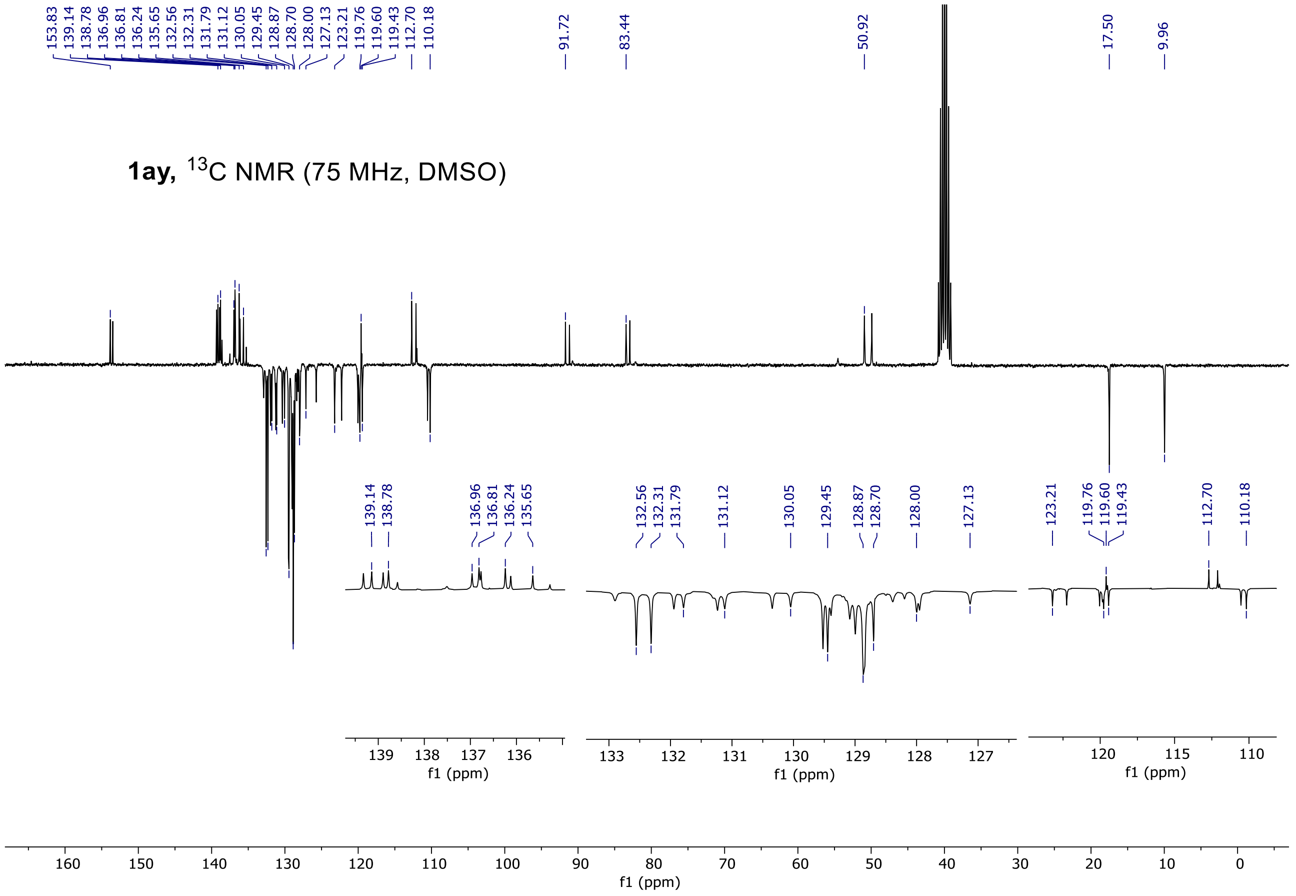

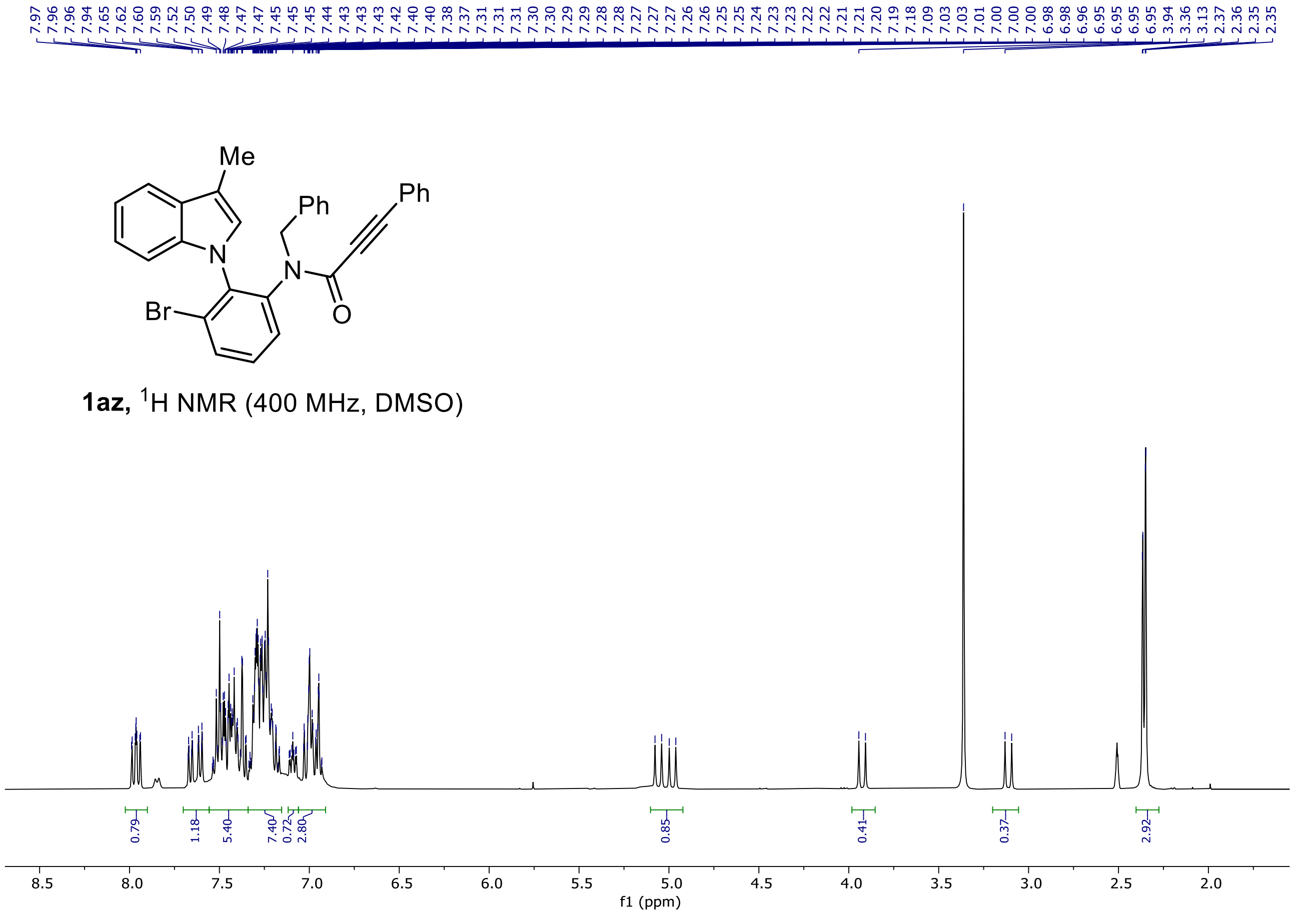
Me
Ph
Ph
N
N
O
Br
1az, 1H NMR (400 MHz, DMSO)
f1 (ppm)

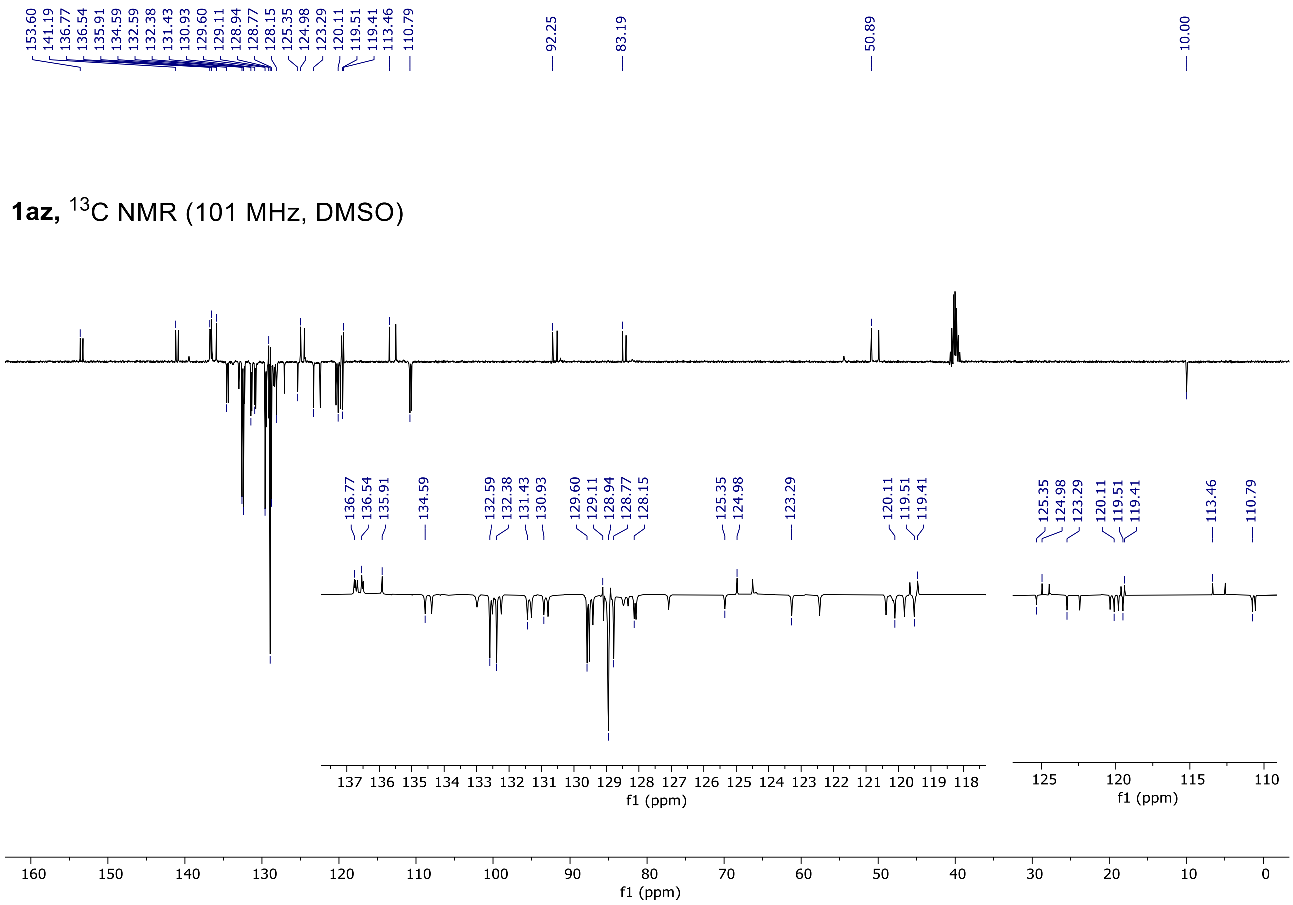
1az, 13C NMR (101 MHz, DMSO)
f1 (ppm)

1az, ^{13}C NMR (101 MHz, DMSO, 363 K)

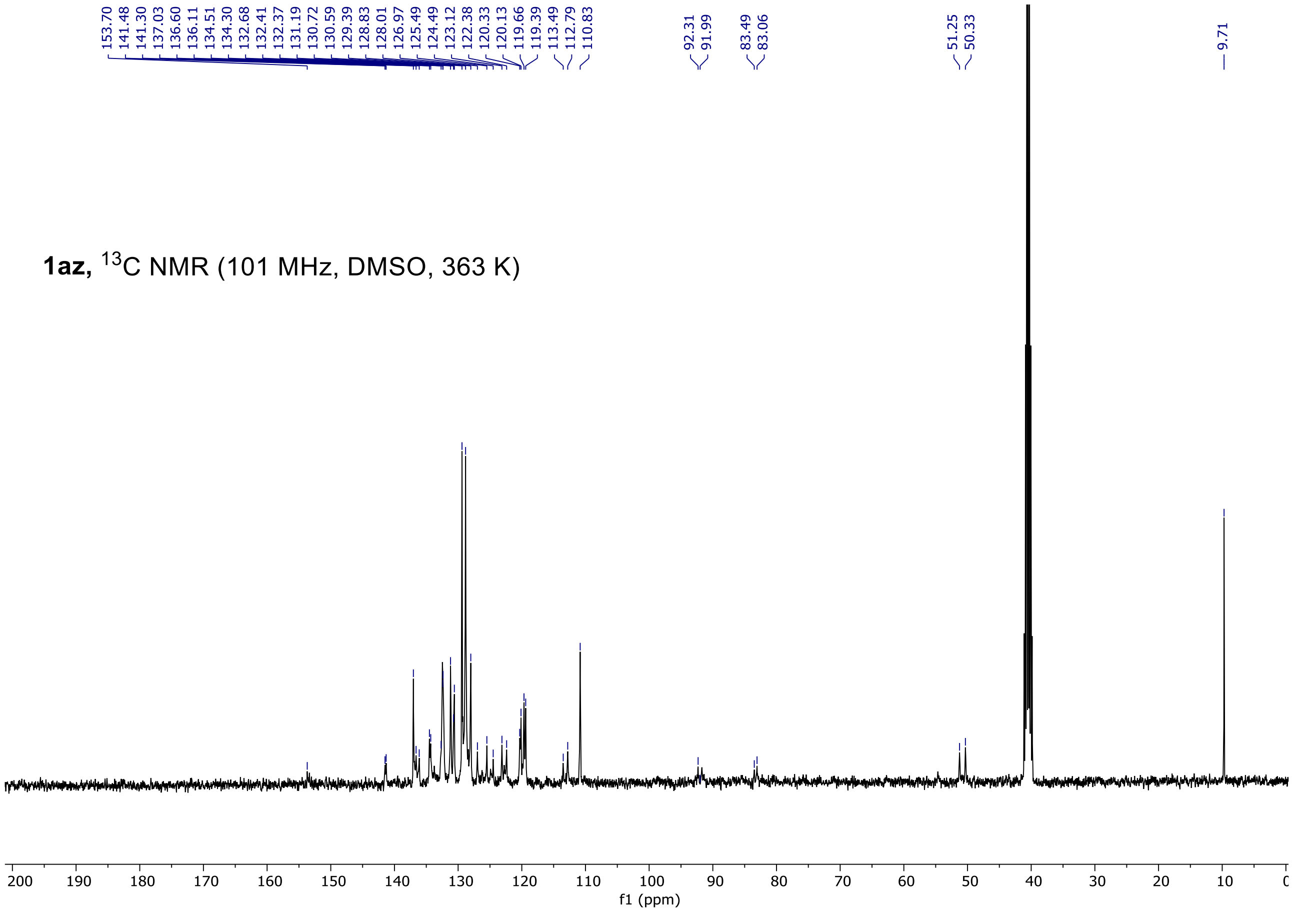

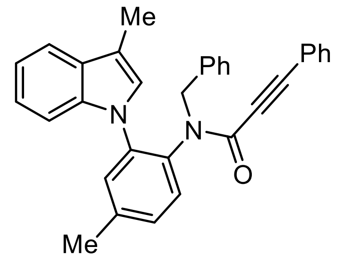

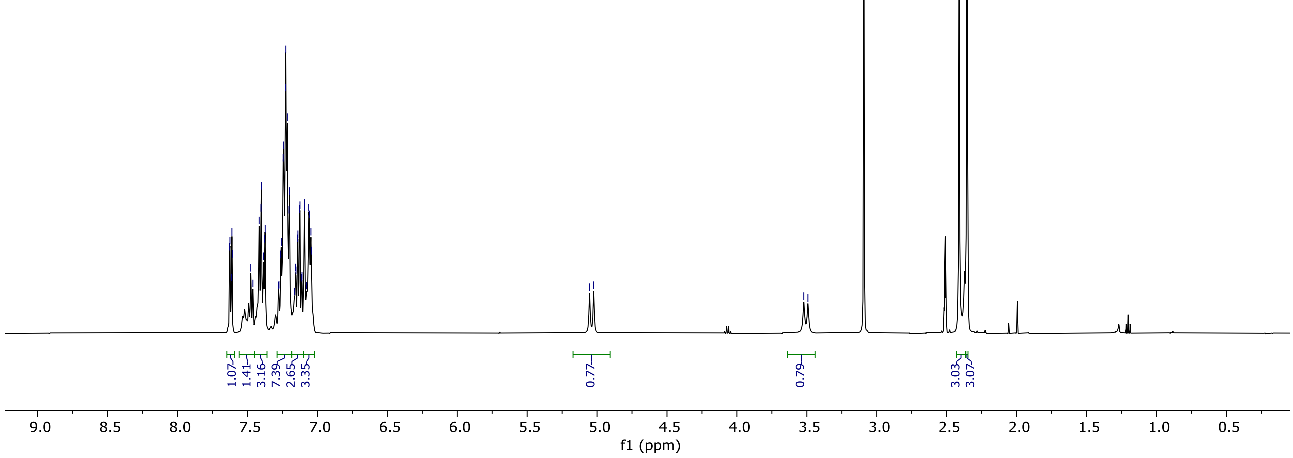

1ba, ^{1}H NMR (500 MHz, DMSO, 353 K)

1ba, ^{13}C NMR (126 MHz, DMSO, 353 K)

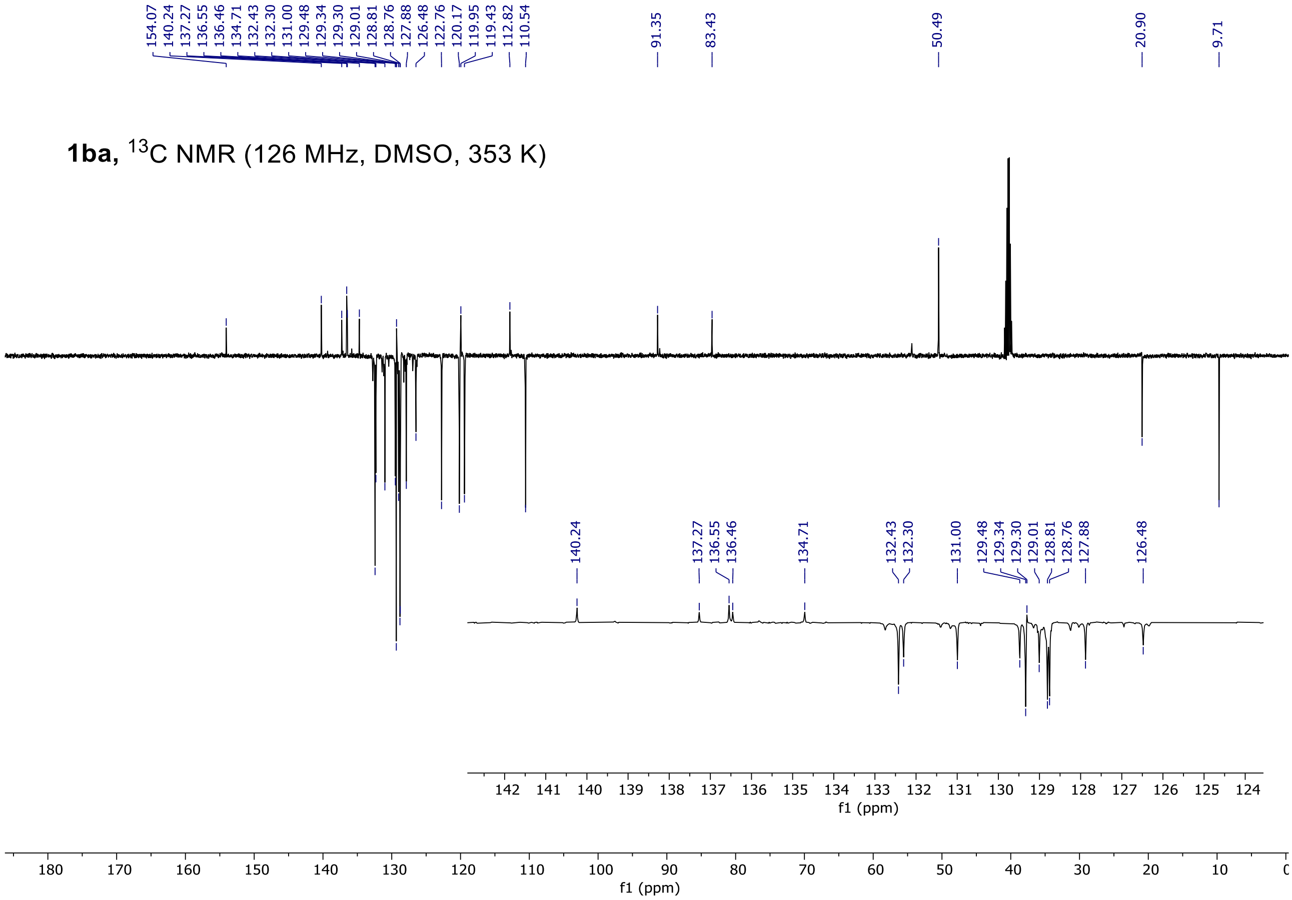

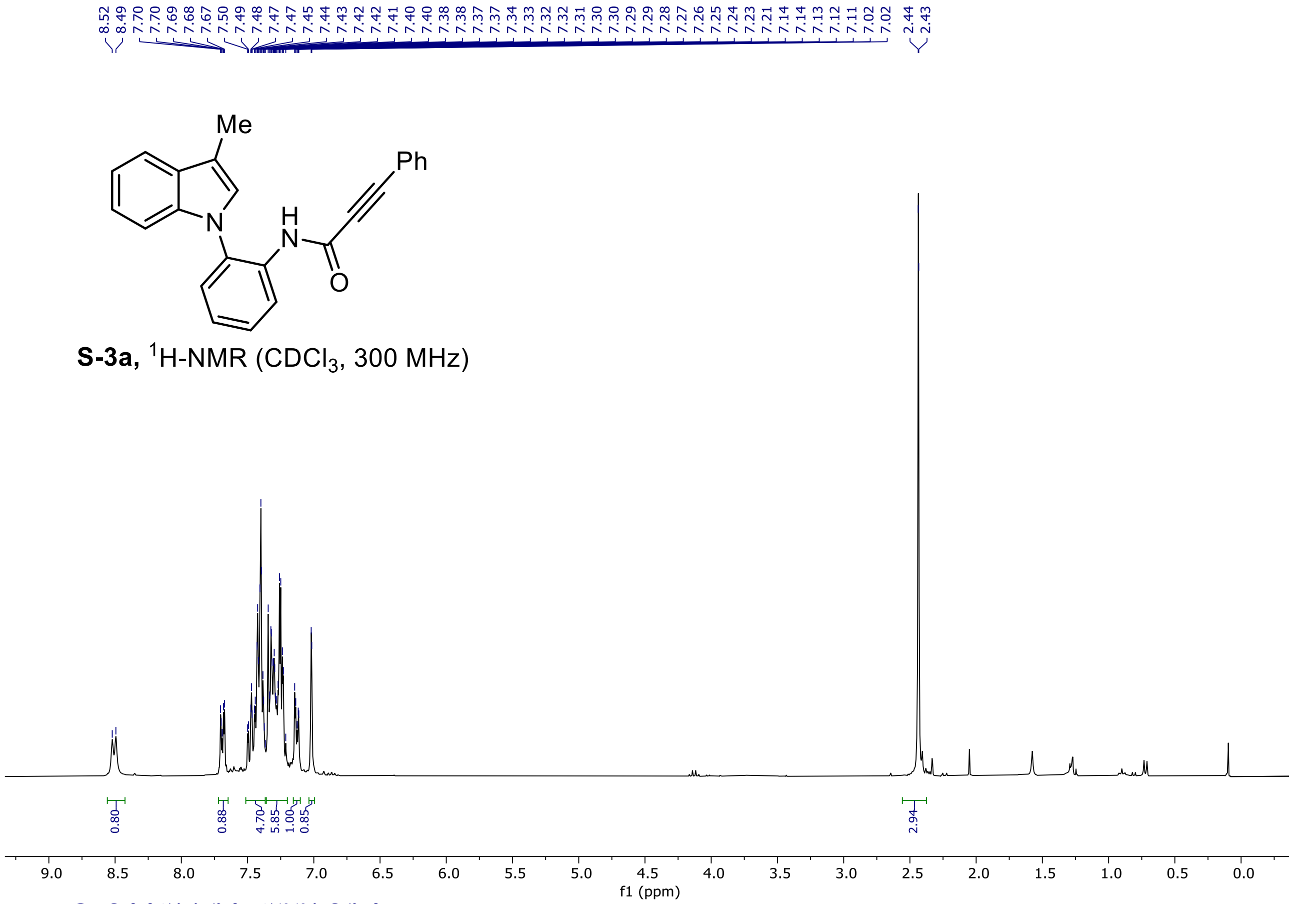
8.52
8.49
7.70
7.70
7.69
7.68
7.67
7.50
7.49
7.48
7.47
7.47
7.45
7.44
7.43
7.42
7.42
7.41
7.40
7.40
7.38
7.38
7.37
7.37
7.34
7.33
7.32
7.32
7.31
7.30
7.30
7.29
7.29
7.28
7.27
7.26
7.25
7.24
7.23
7.21
7.14
7.14
7.13
7.12
7.11
7.02
7.02
2.44
2.43
Me
Ph
H
N
N
O
S-3a, 1H-NMR (CDCl3, 300 MHz)
0.80
0.88
4.70
5.85
1.00
0.85
2.94
f1 (ppm)

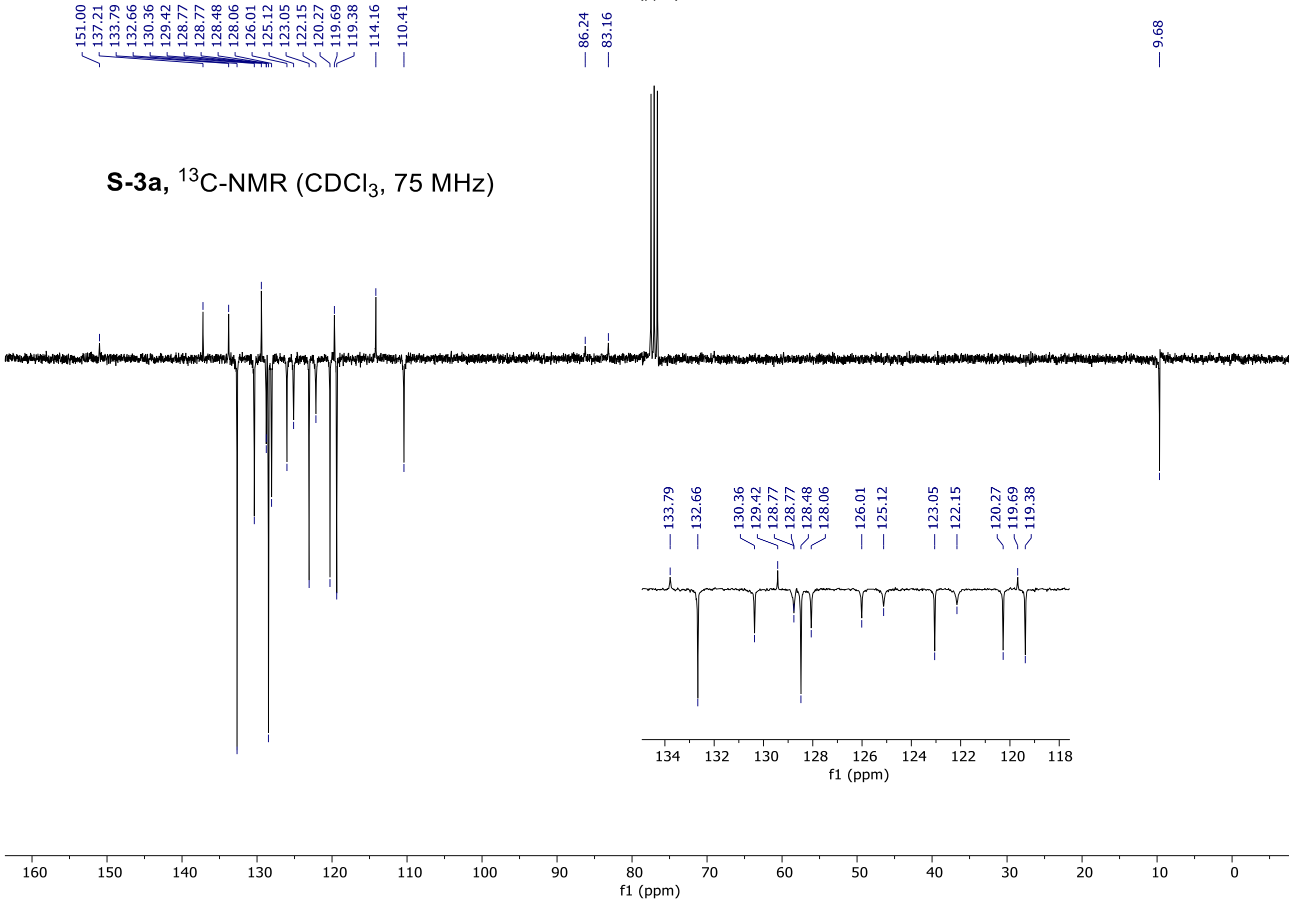
151.00
137.21
133.79
132.66
130.36
129.42
128.77
128.77
128.48
128.06
126.01
125.12
123.05
122.15
120.27
119.69
119.38
114.16
110.41
86.24
83.16
9.68
S-3a, 13C-NMR (CDCl3, 75 MHz)
f1 (ppm)

S-3b, ^{1}H-NMR ($CDCl_3$, 300 MHZ)

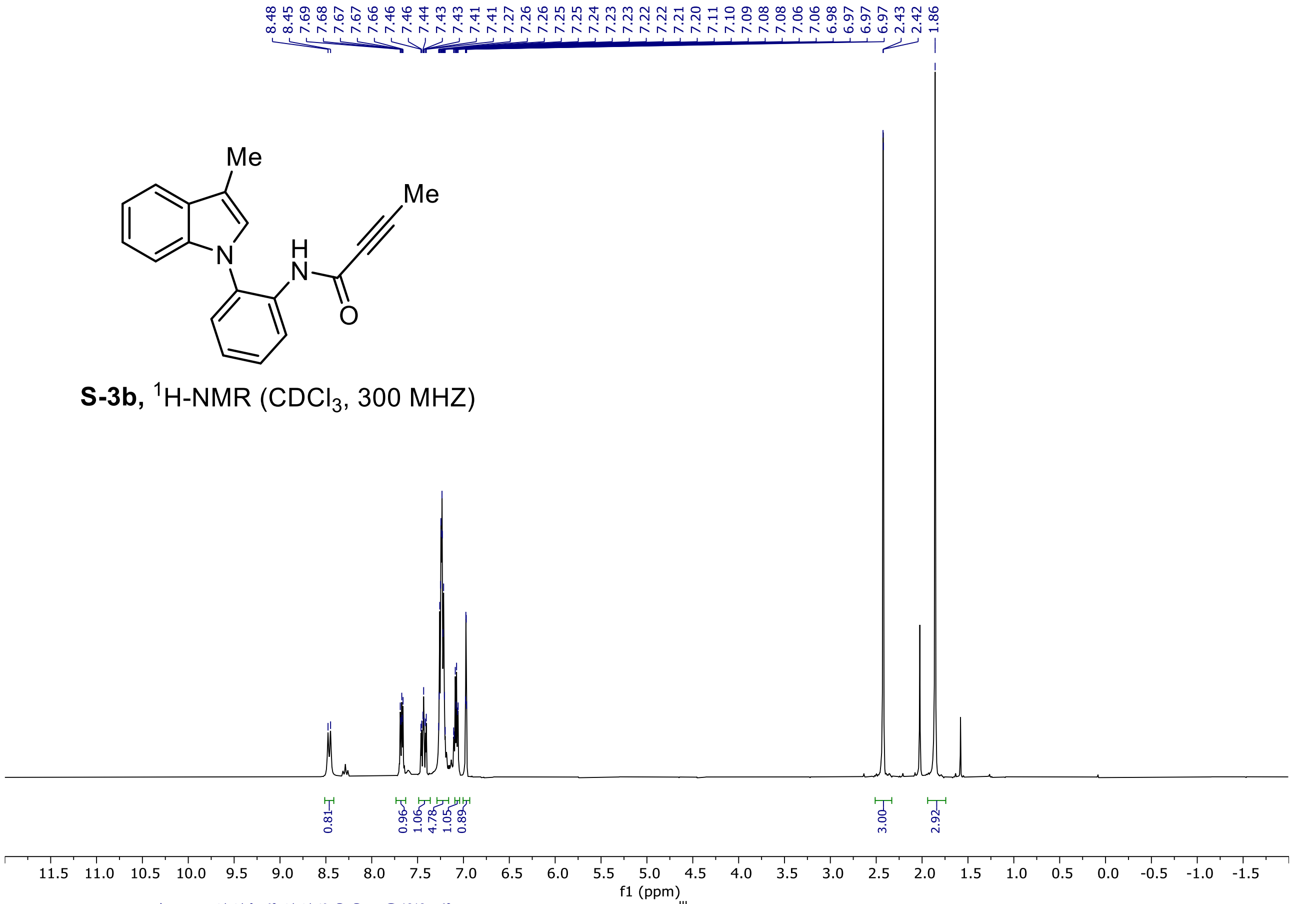

S-3b, ^{13}C-NMR ($CDCl_3$, 75 MHZ)

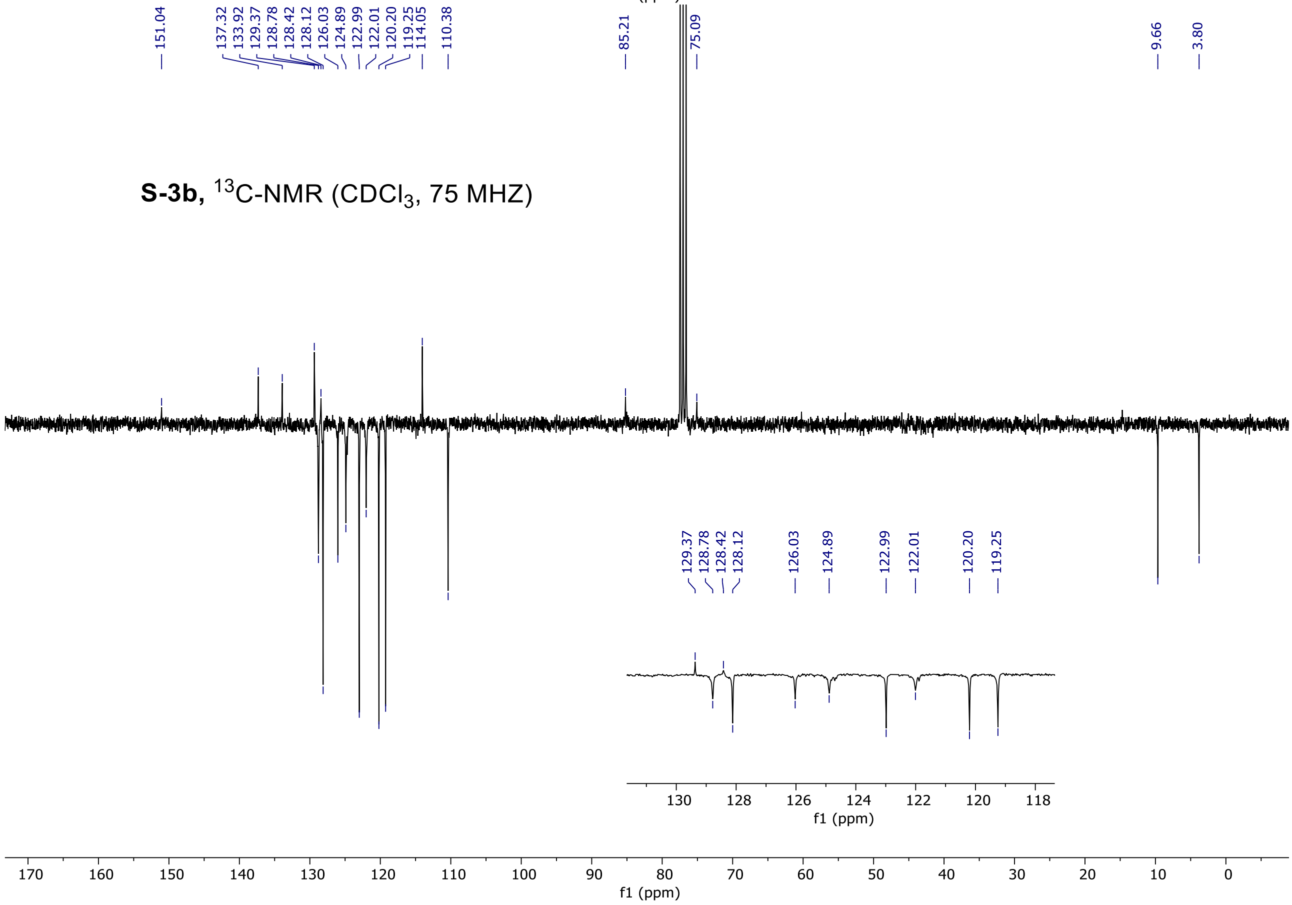

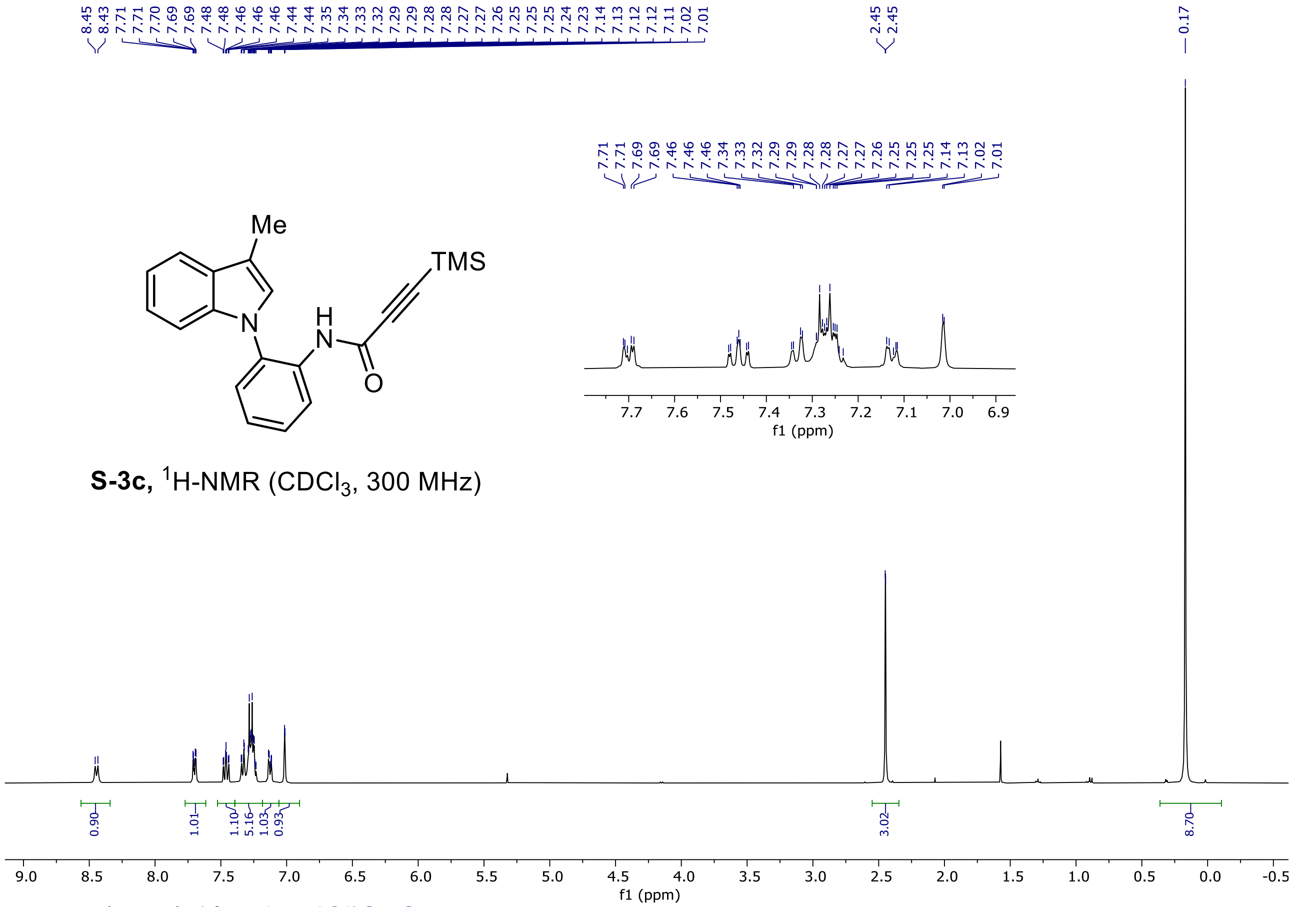

S-3c, ^{1}H-NMR ($CDCl_3$, 300 MHz)

S-3c, ^{13}C-NMR ($CDCl_3$, 101 MHz)

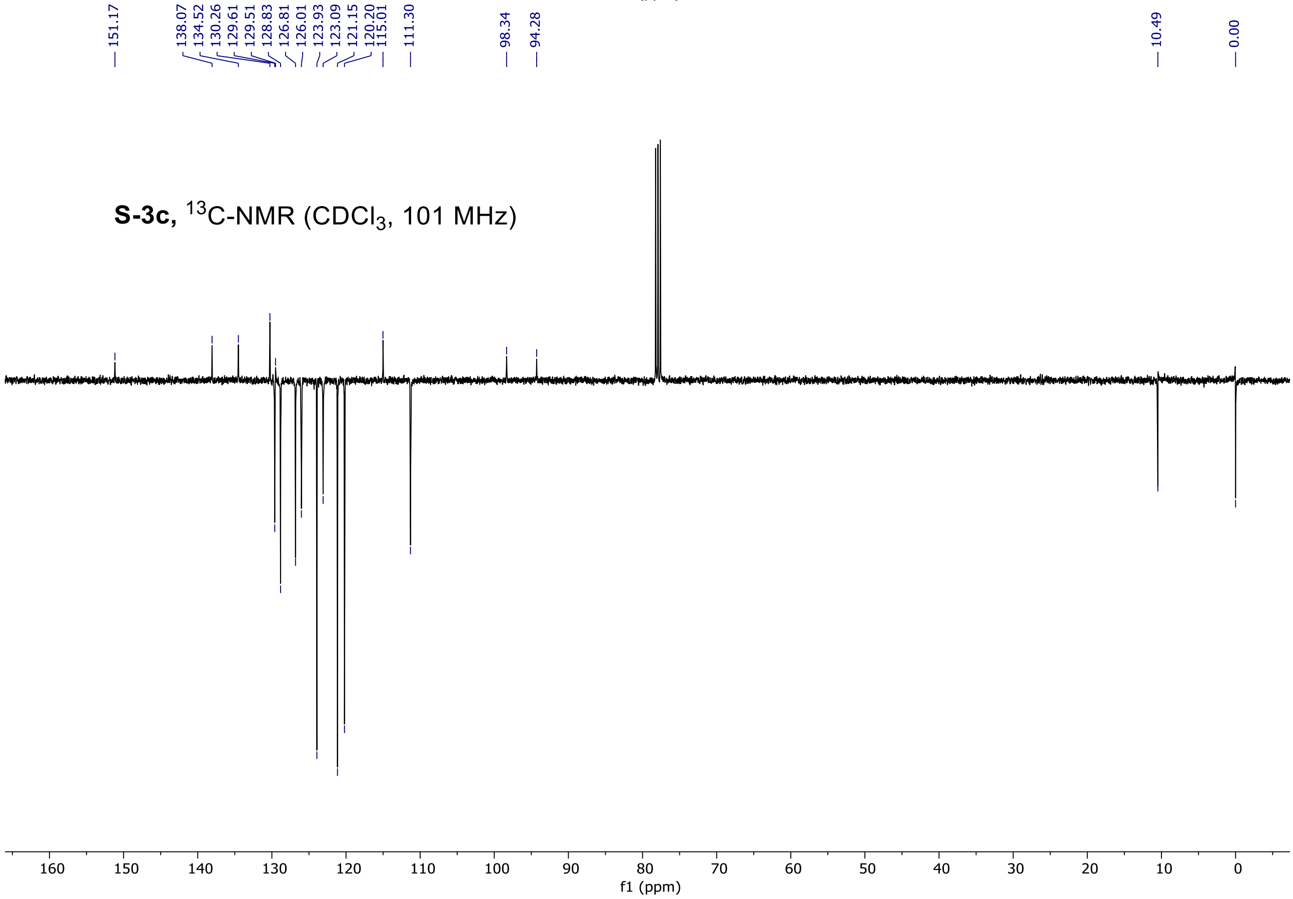

1al, ^{1}H NMR (600 MHz, DMSO, 353 K)

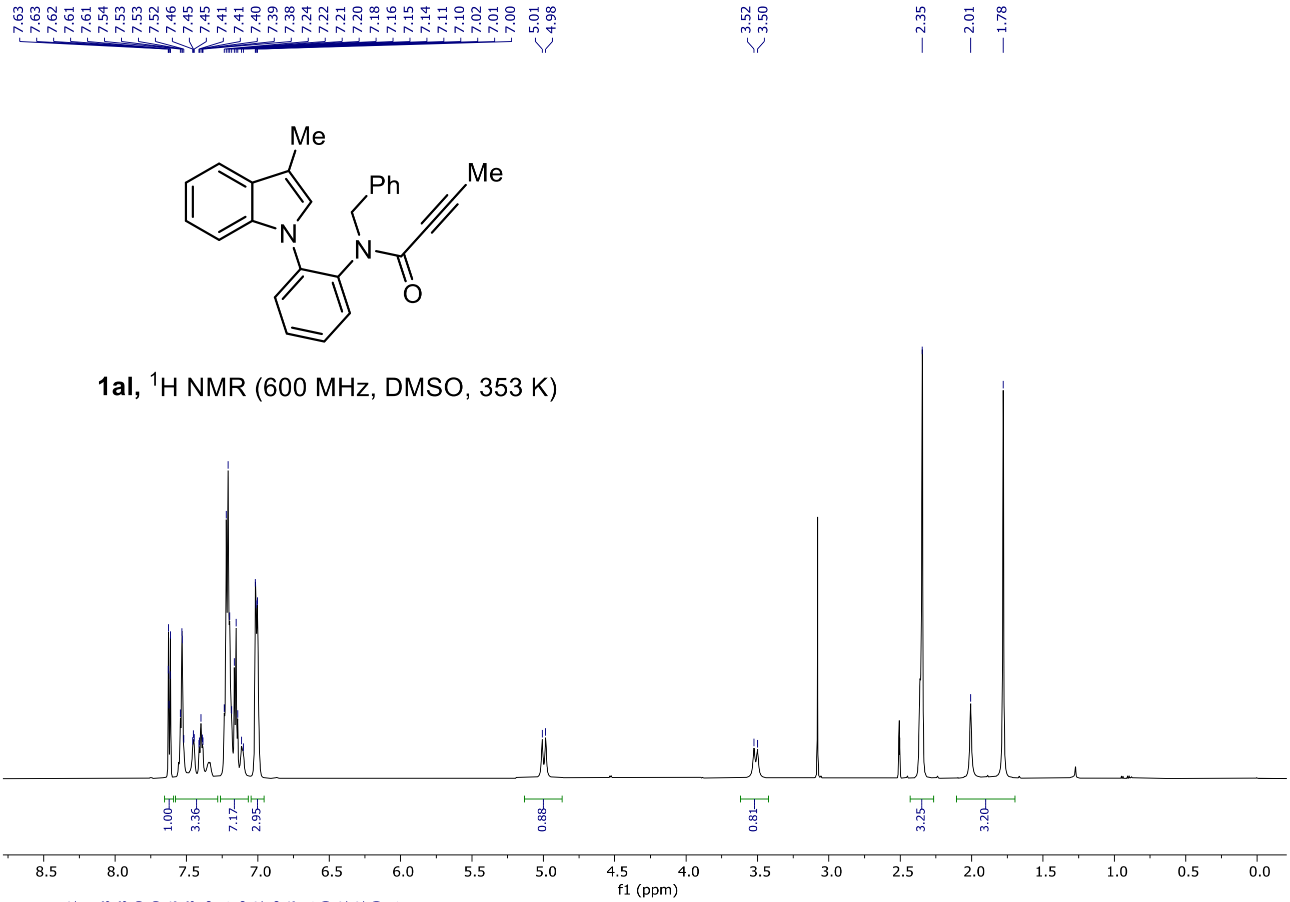

1al, ^{13}C NMR (151 MHz, DMSO, 353 K)

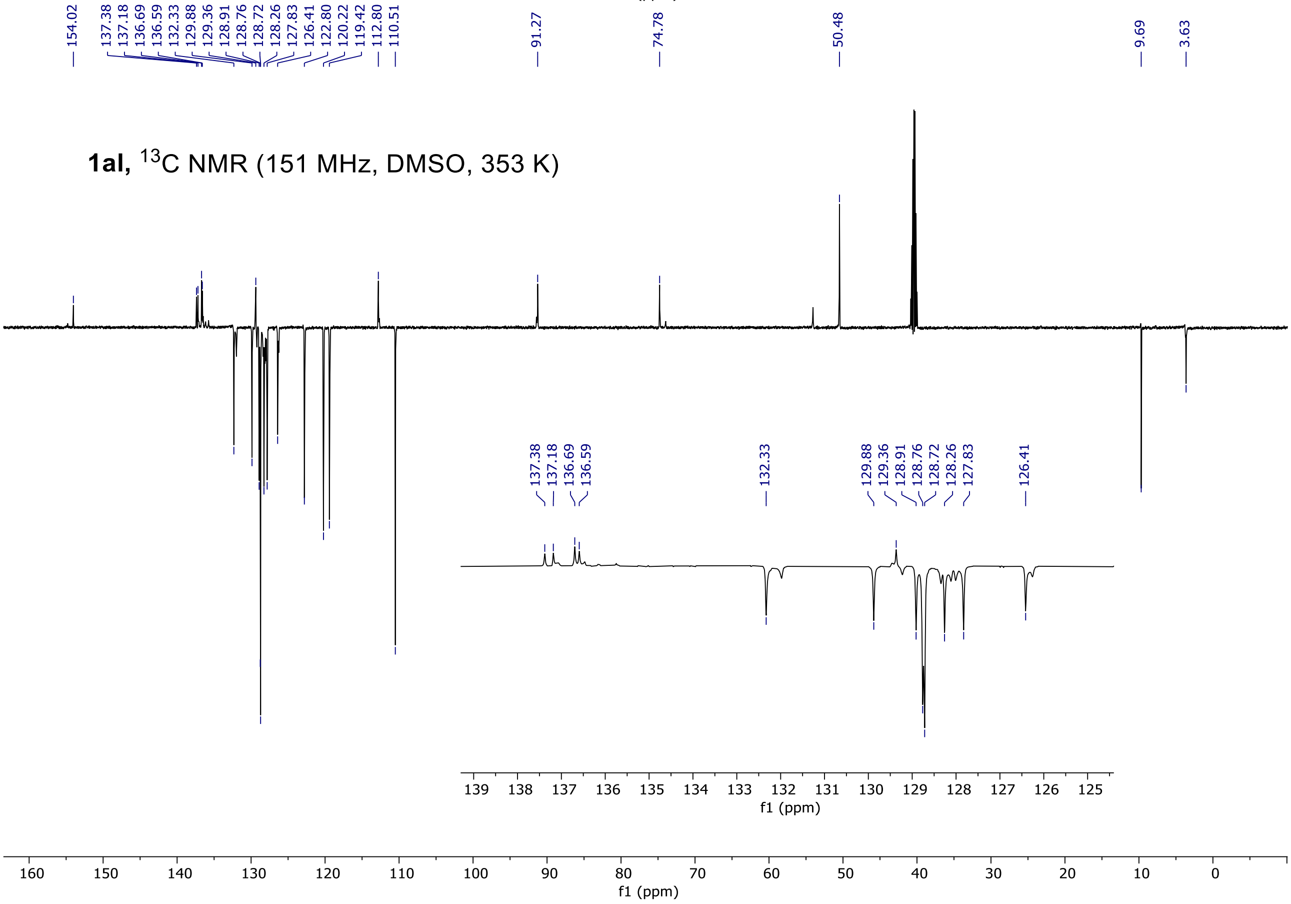

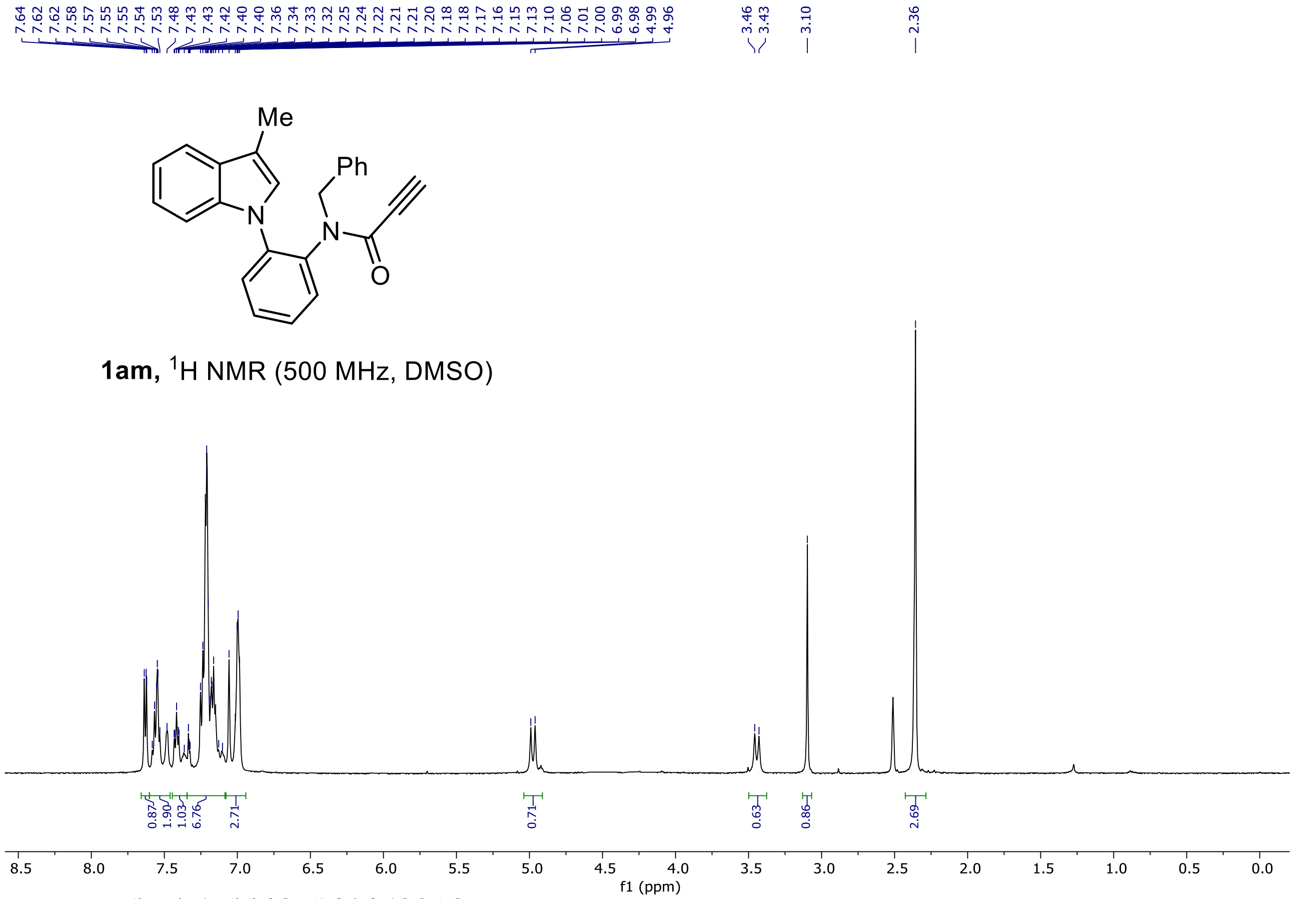

1am, ¹H NMR (500 MHz, DMSO)

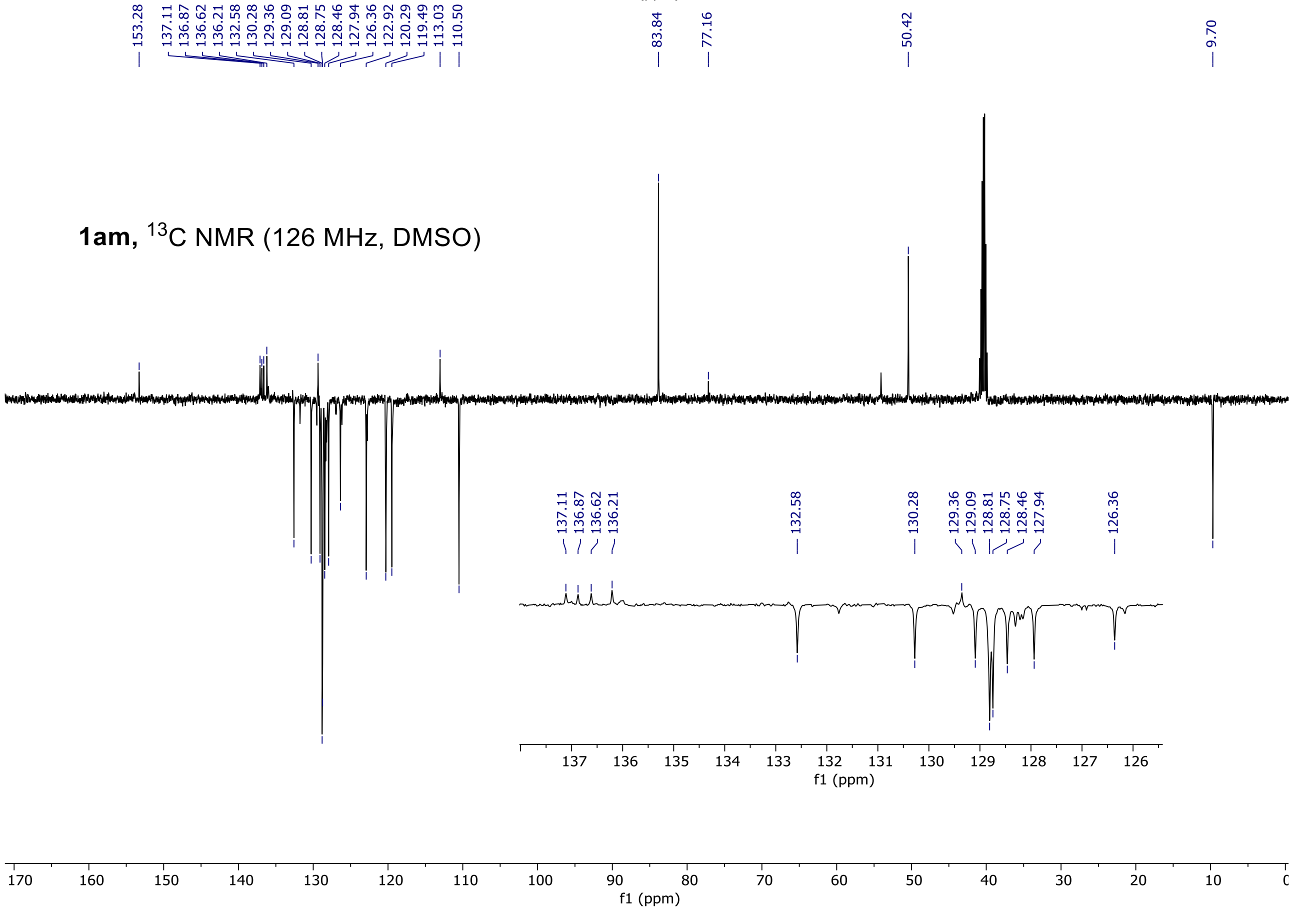

1am, ¹³C NMR (126 MHz, DMSO)

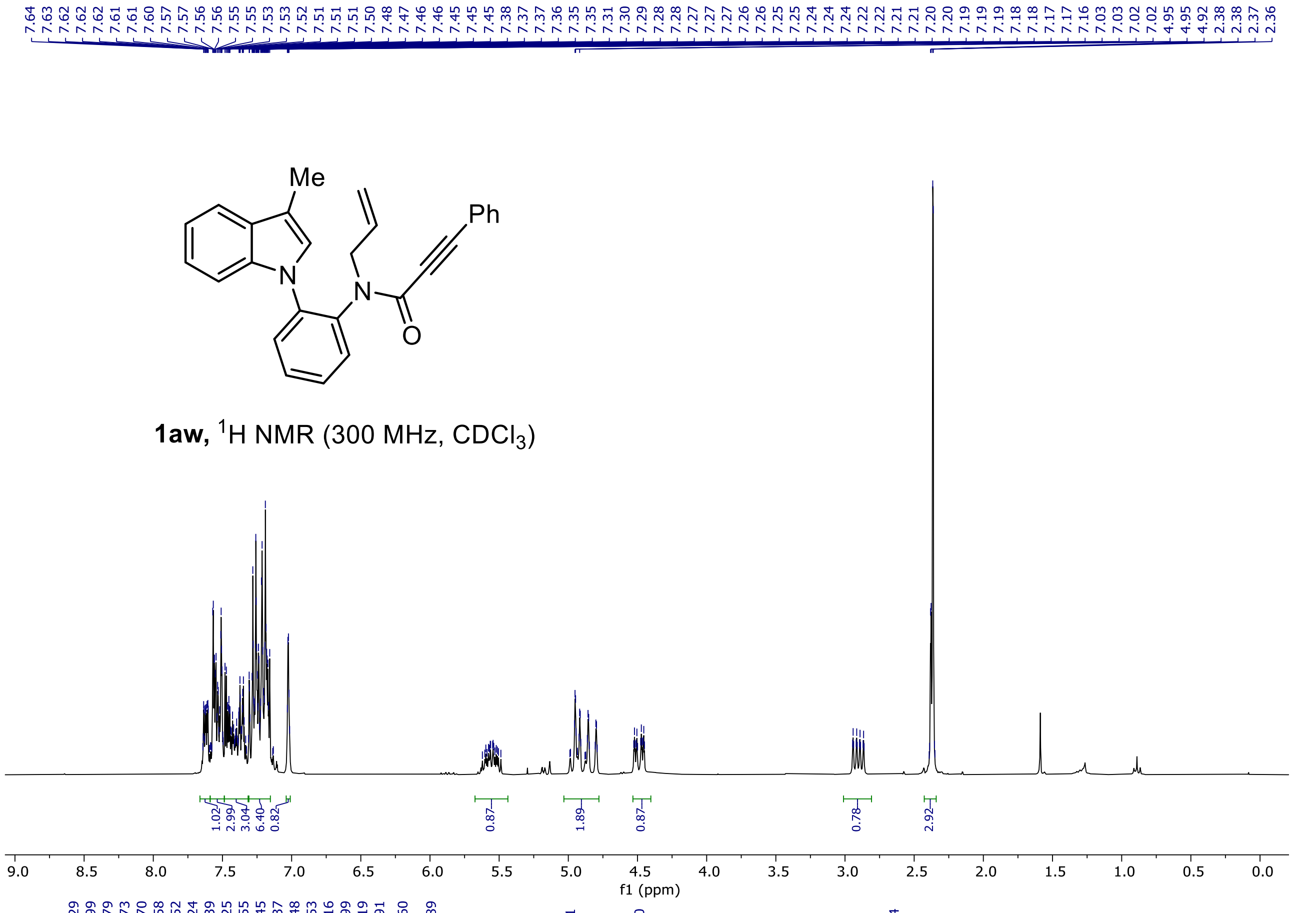
7.64 7.63 7.62 7.62 7.61 7.61 7.60 7.57 7.57 7.56 7.56 7.55 7.55 7.53 7.53 7.52 7.51 7.51 7.51 7.50 7.48 7.47 7.46 7.46 7.45 7.45 7.45 7.38 7.37 7.37 7.36 7.35 7.35 7.31 7.30 7.29 7.28 7.28 7.27 7.27 7.27 7.26 7.26 7.25 7.25 7.24 7.24 7.24 7.22 7.22 7.21 7.21 7.20 7.20 7.19 7.19 7.18 7.18 7.17 7.17 7.16 7.03 7.03 7.02 7.02 4.95 4.95 4.92 2.38 2.37 2.36
Me
Ph
N
N
O
1aw, 1H NMR (300 MHz, CDCl3)
1.02 2.99 3.04 6.40 0.82 0.87 1.89 0.87 0.78 2.92
9.0 8.5 8.0 7.5 7.0 6.5 6.0 5.5 5.0 4.5 4.0 3.5 3.0 2.5 2.0 1.5 1.0 0.5 0.0
f1 (ppm)

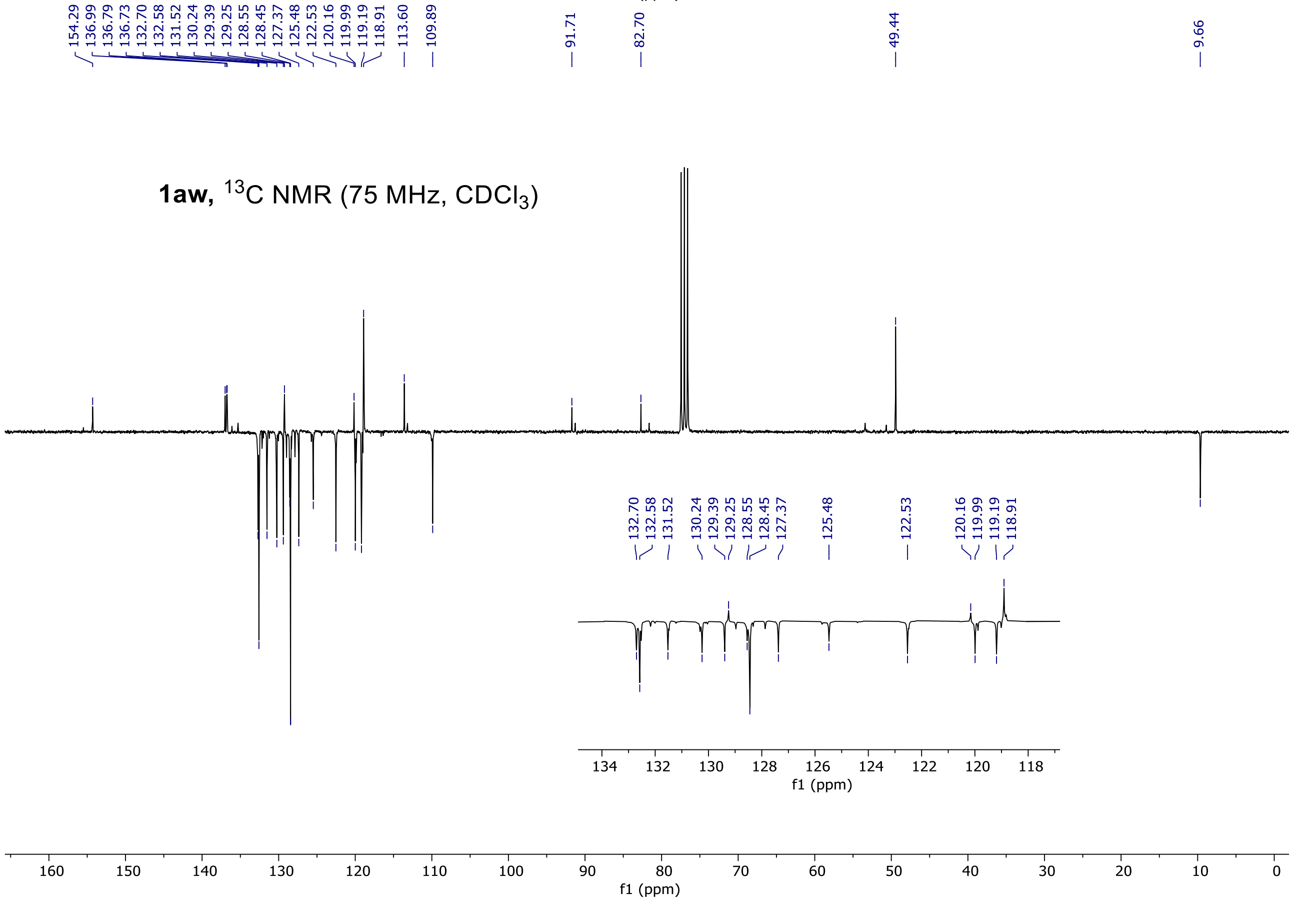
154.29 136.99 136.79 136.73 132.70 132.58 131.52 130.24 129.39 129.25 128.55 128.45 127.37 125.48 122.53 120.16 119.99 119.19 118.91 113.60 109.89
91.71
82.70
49.44
9.66
1aw, 13C NMR (75 MHz, CDCl3)
132.70 132.58 131.52 130.24 129.39 129.25 128.55 128.45 127.37 125.48 122.53 120.16 119.99 119.19 118.91
134 132 130 128 126 124 122 120 118
f1 (ppm)
160 150 140 130 120 110 100 90 80 70 60 50 40 30 20 10 0
f1 (ppm)

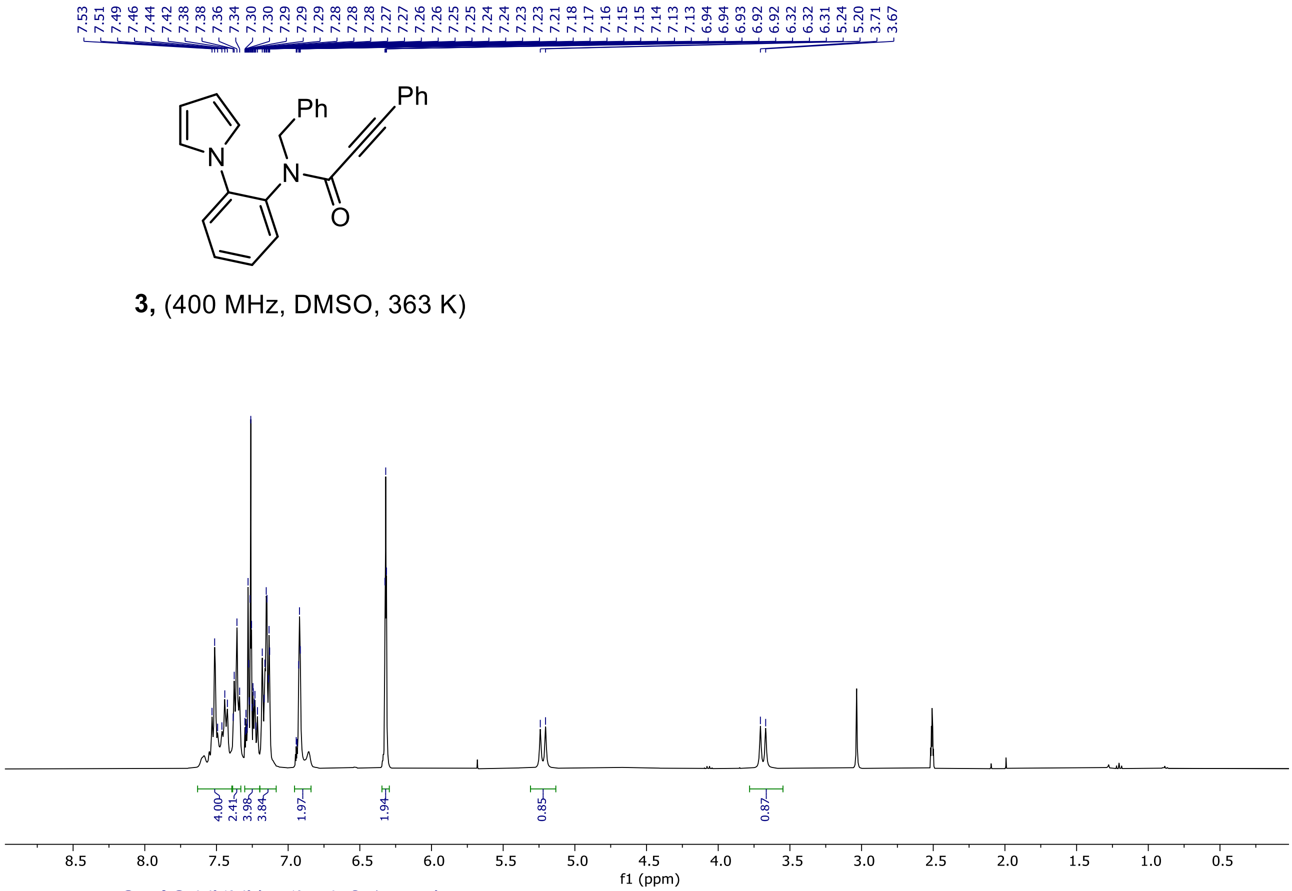

3, (400 MHz, DMSO, 363 K)

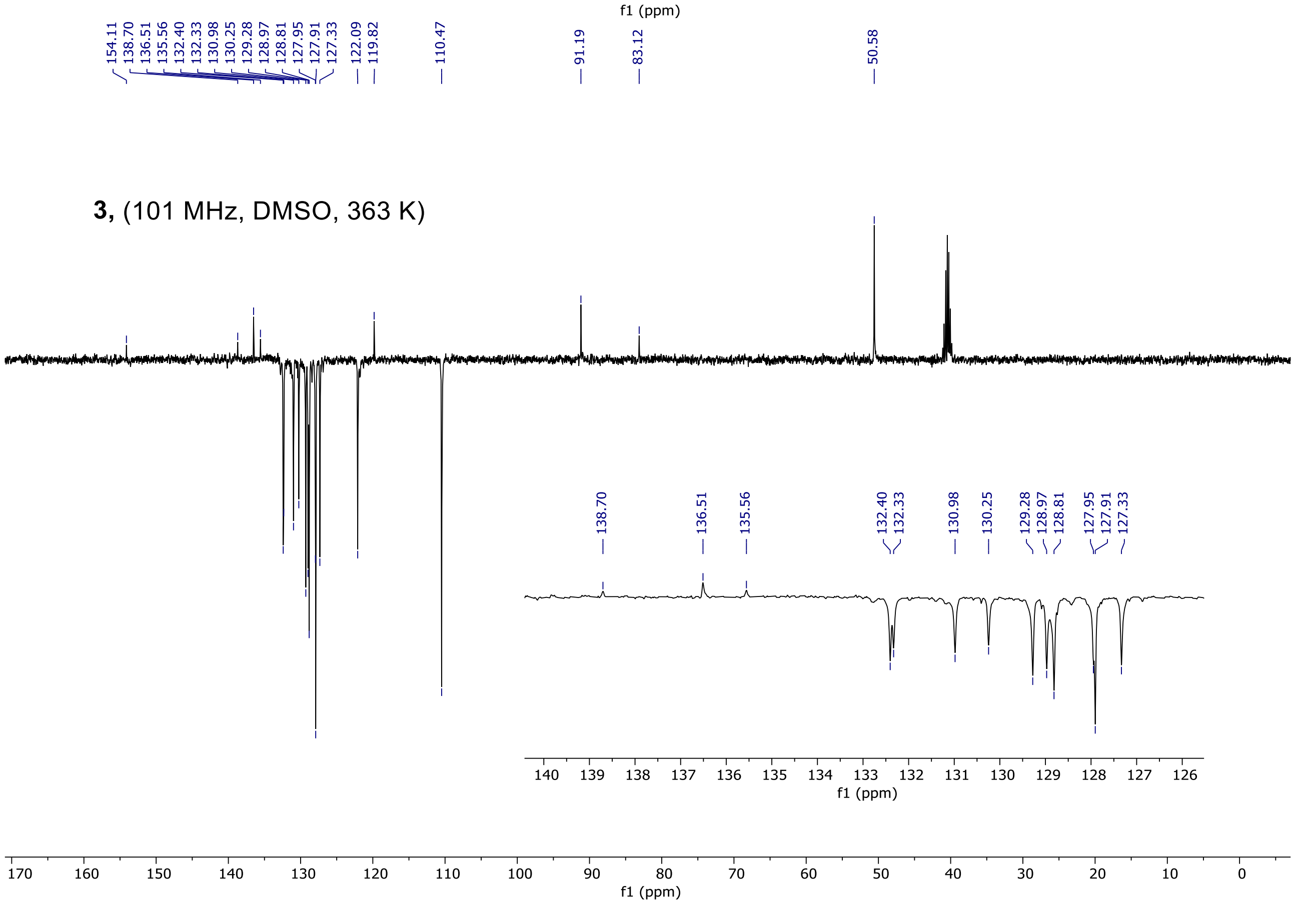

3, (101 MHz, DMSO, 363 K)

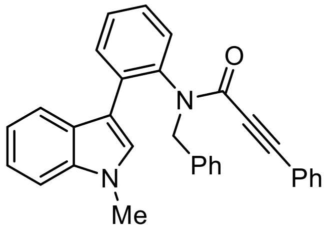

5, ^{1}H NMR (400 MHz, DMSO)

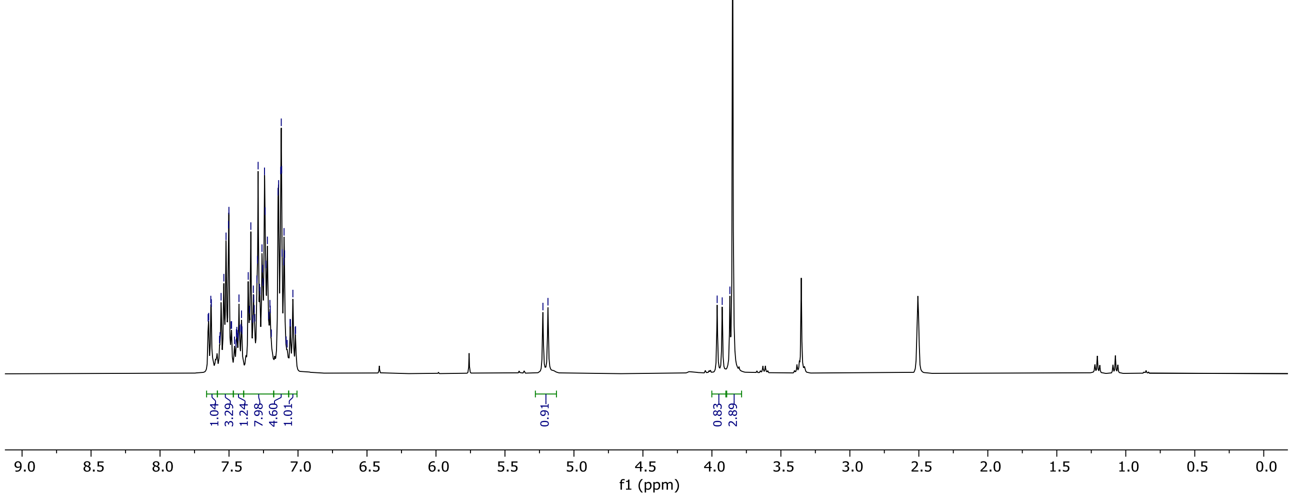

5, ^{13}C NMR (101 MHz, DMSO)

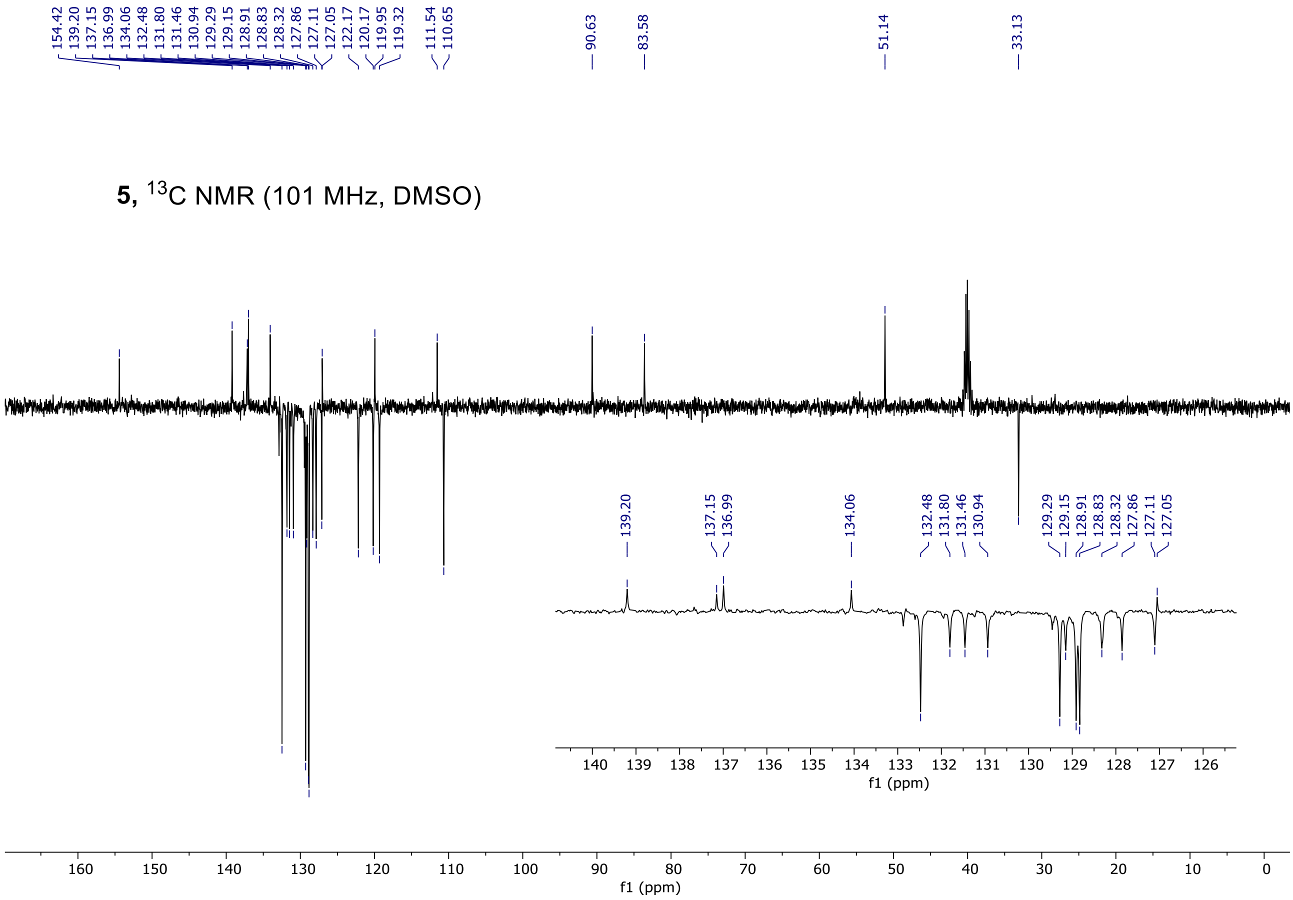

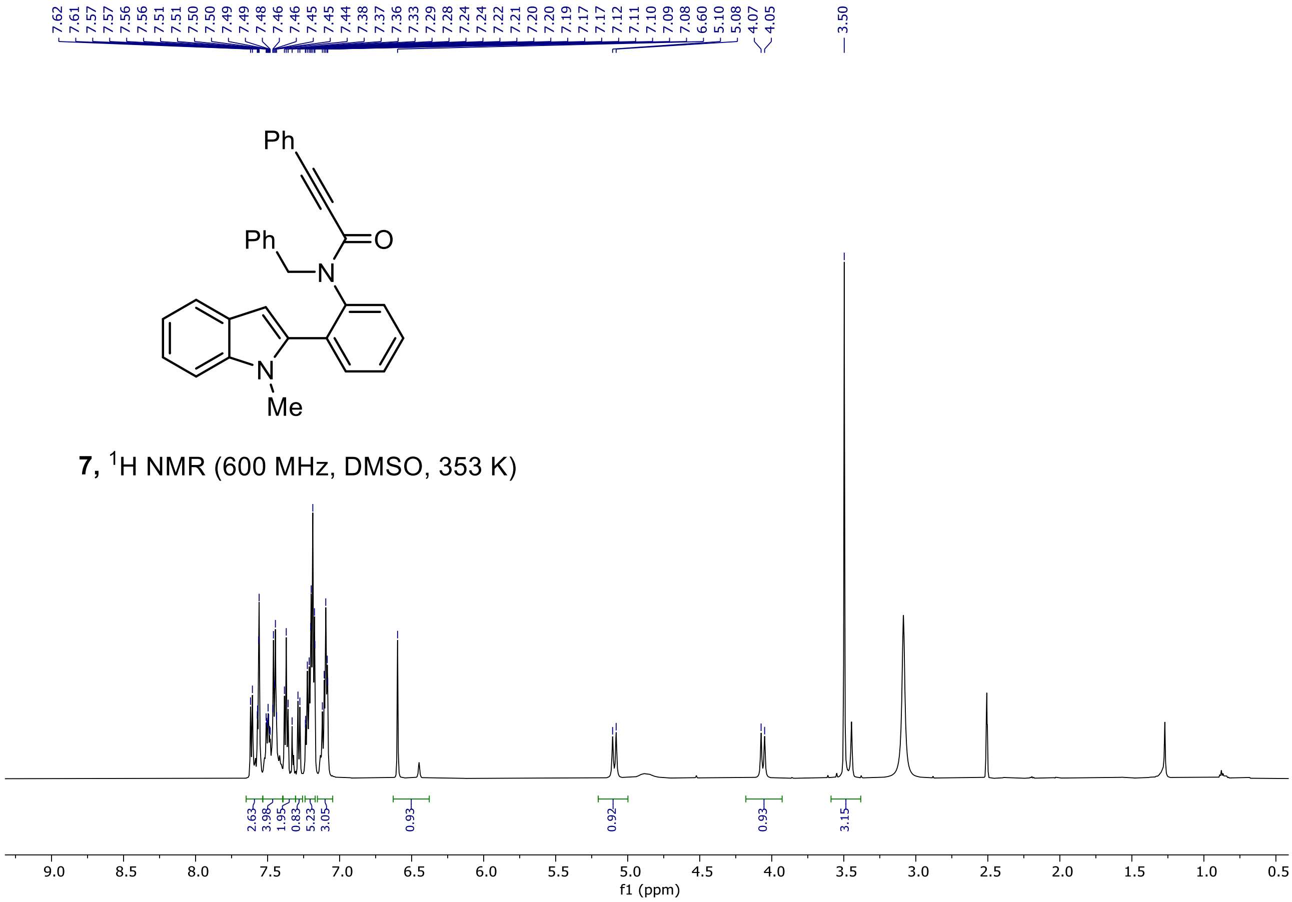

7, ^{1}H NMR (600 MHz, DMSO, 353 K)

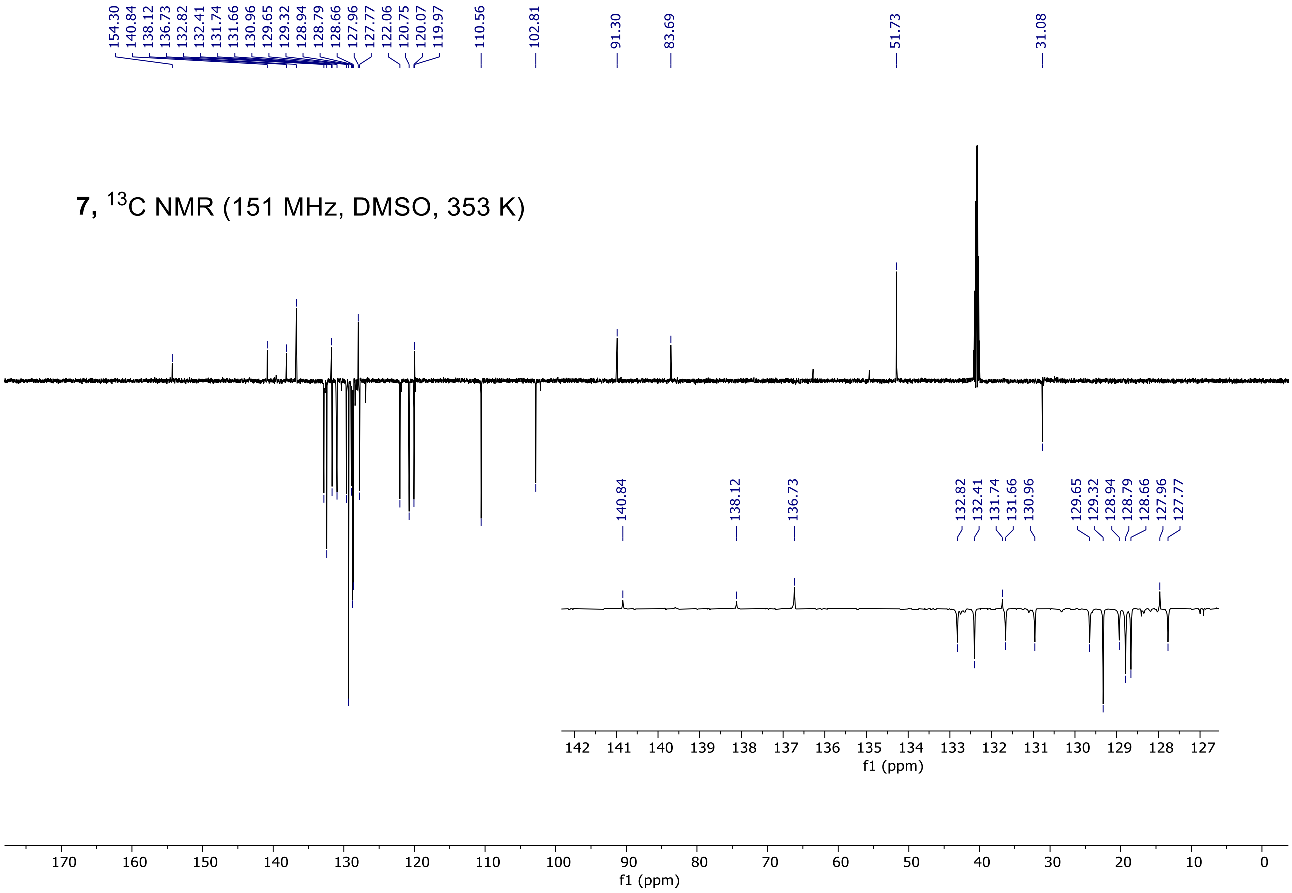

7, ^{13}C NMR (151 MHz, DMSO, 353 K)

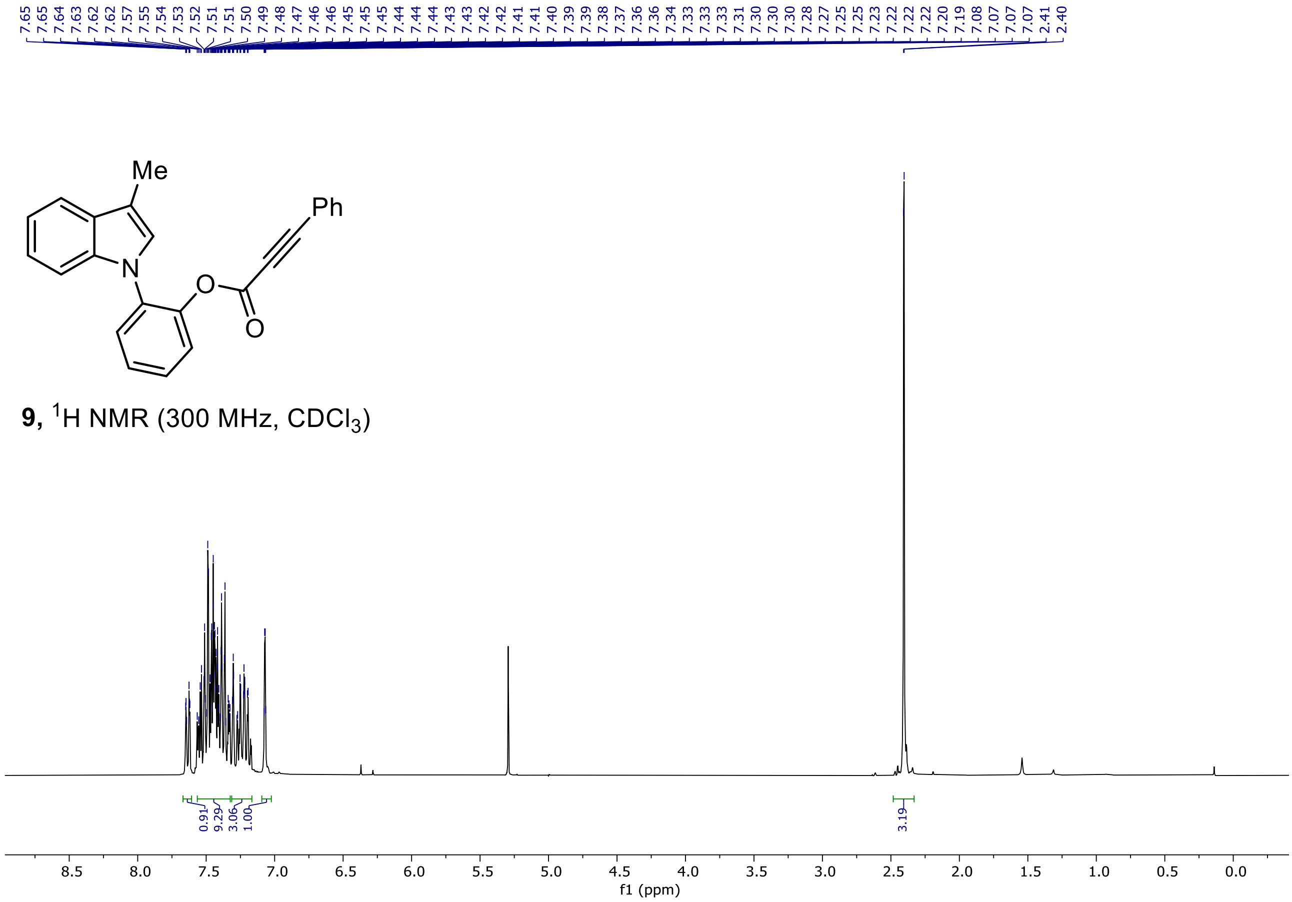

9, ^{1}H NMR (300 MHz, $CDCl_3$)

9, ^{13}C NMR (75 MHz, $CDCl_3$)

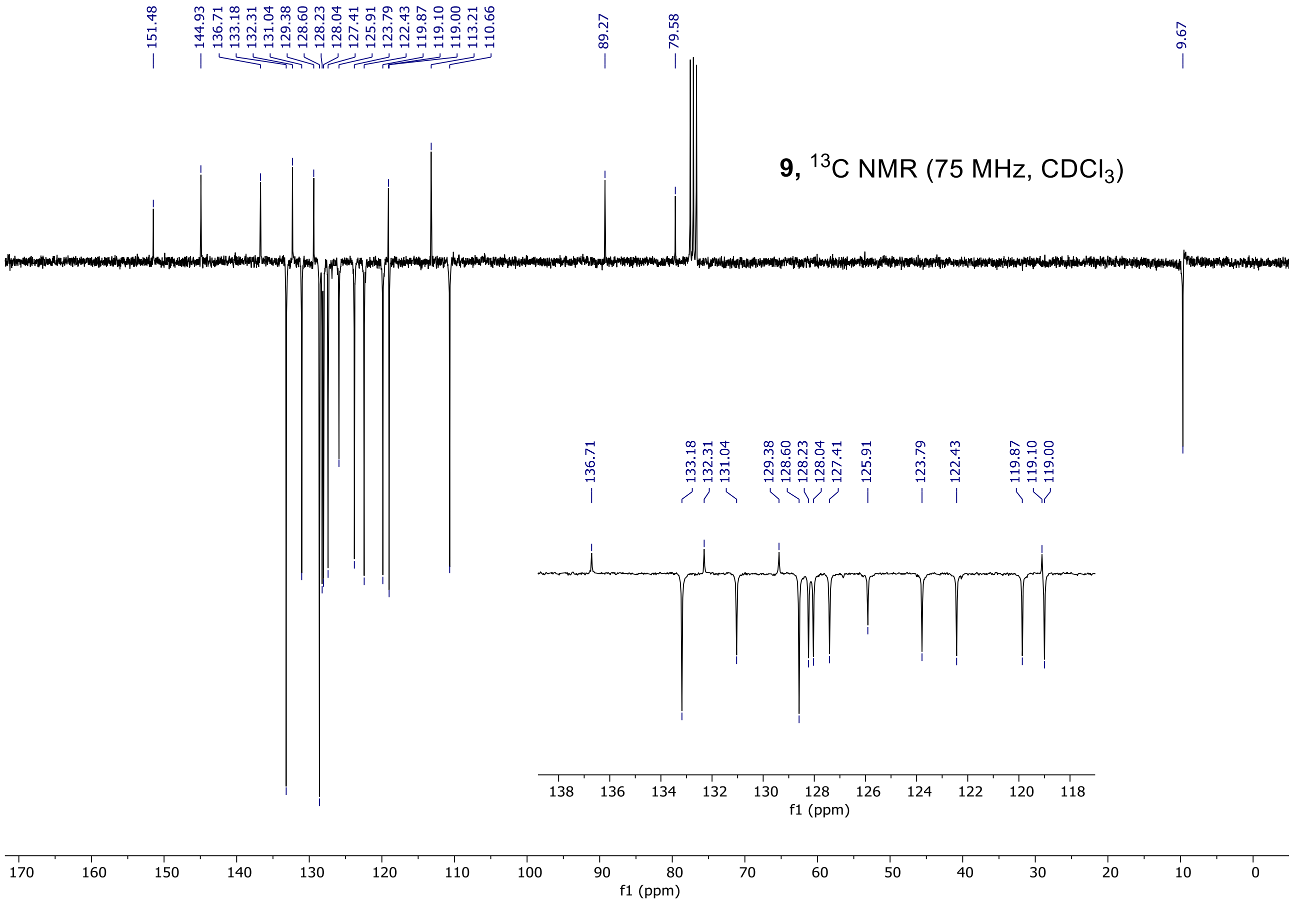

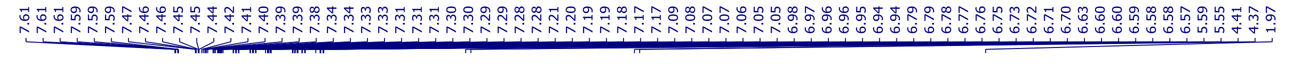

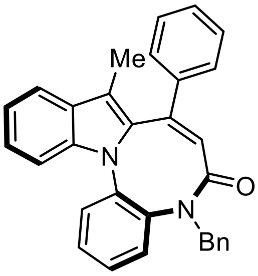

2aa, ^{1}H NMR (300 MHz, $CDCl_3$)

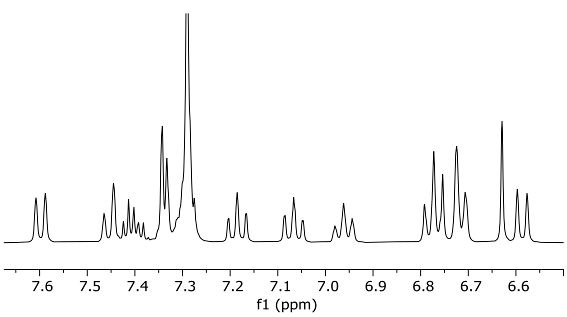

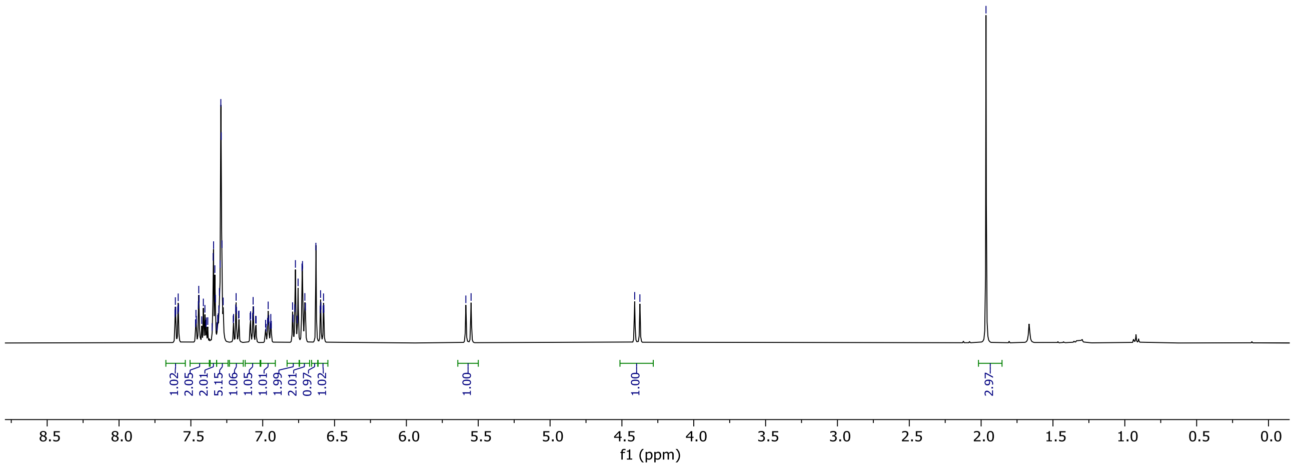

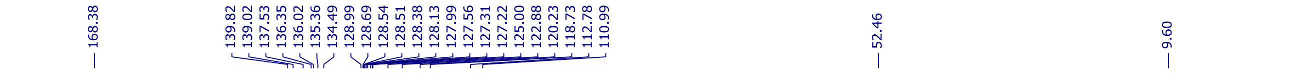

2aa, ^{13}C NMR (101 MHz, $CDCl_3$)

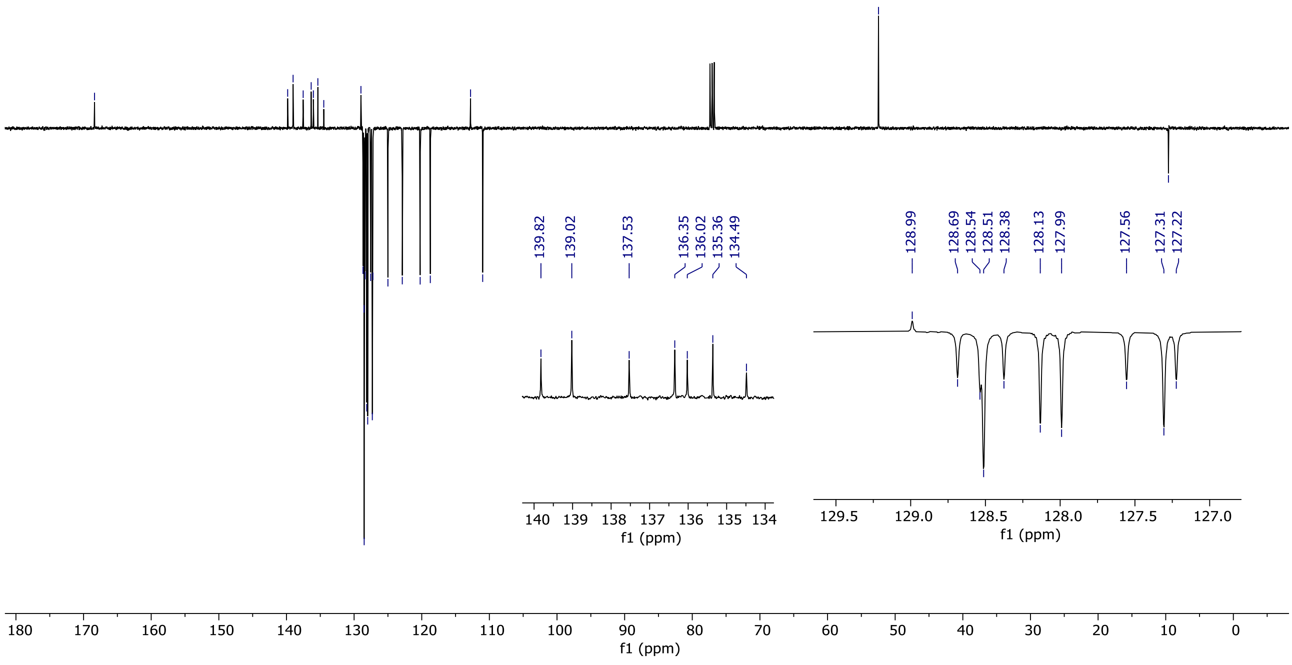

2aa, COSY (400 MHz, $CDCl_3$)

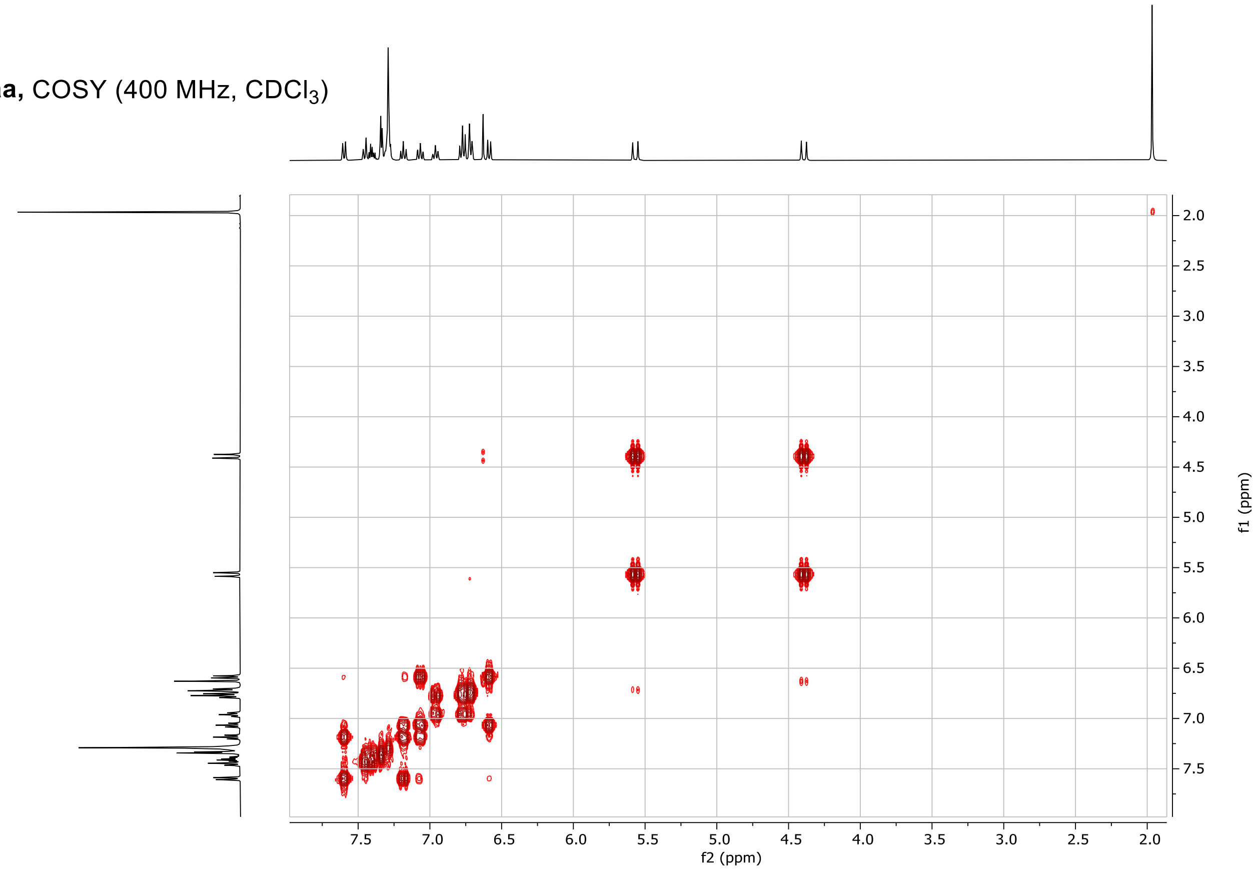

2aa, HSQC ($CDCl_3$)

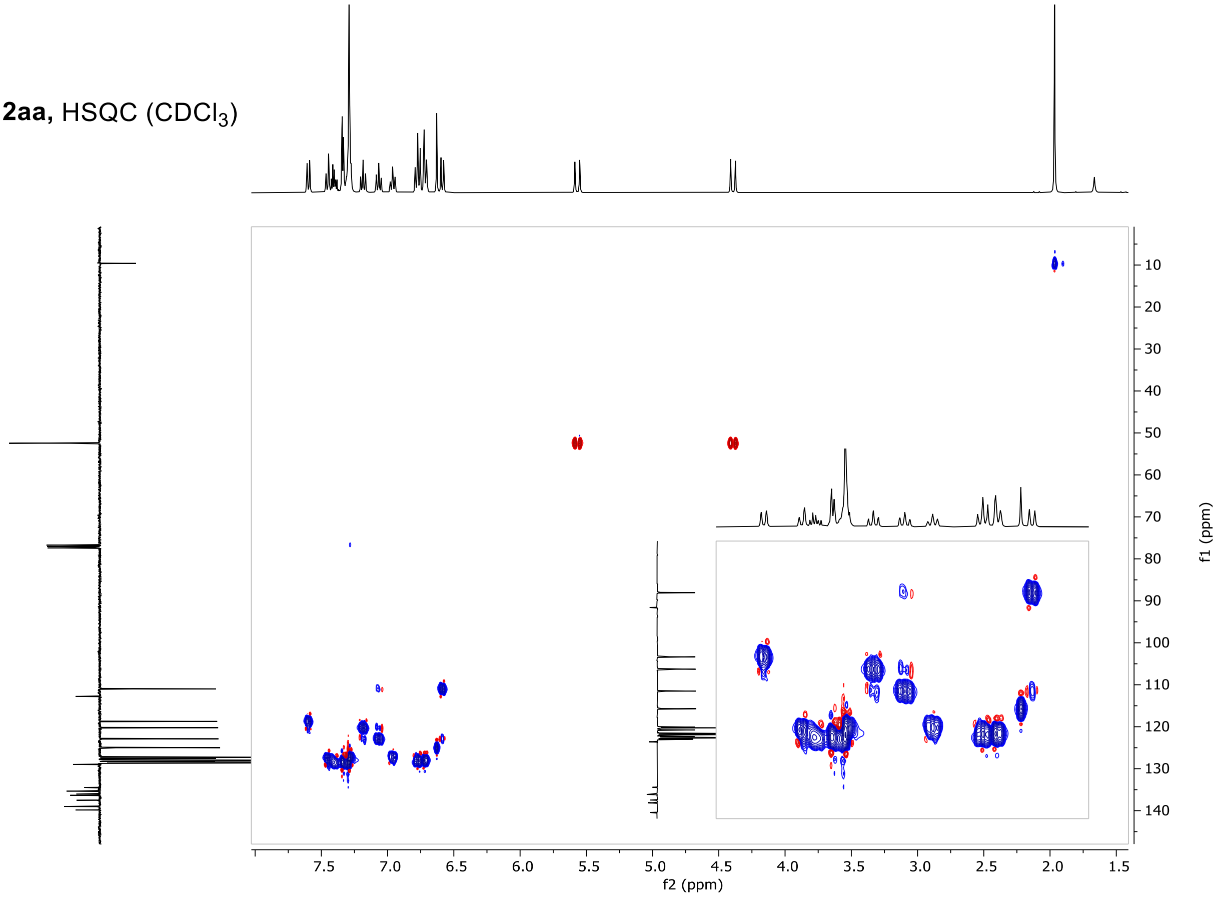

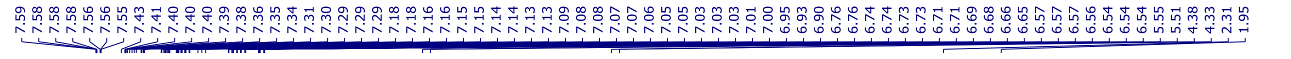

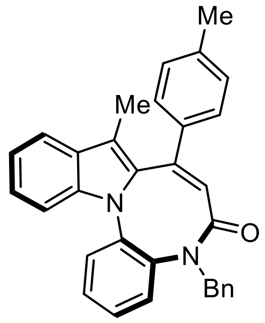

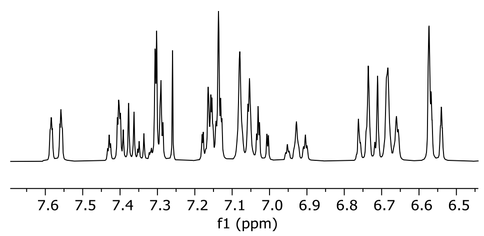

2ab, ^{1}H NMR (300 MHz, $CDCl_3$)

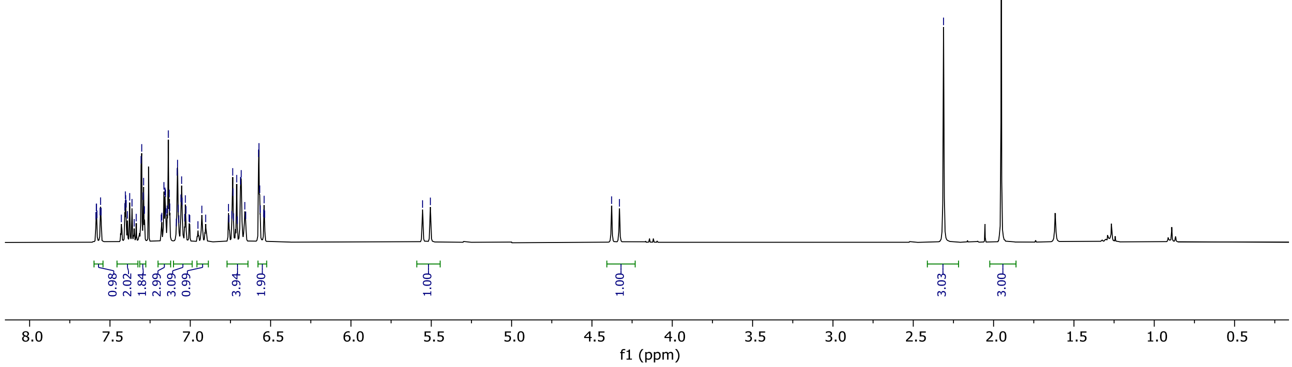

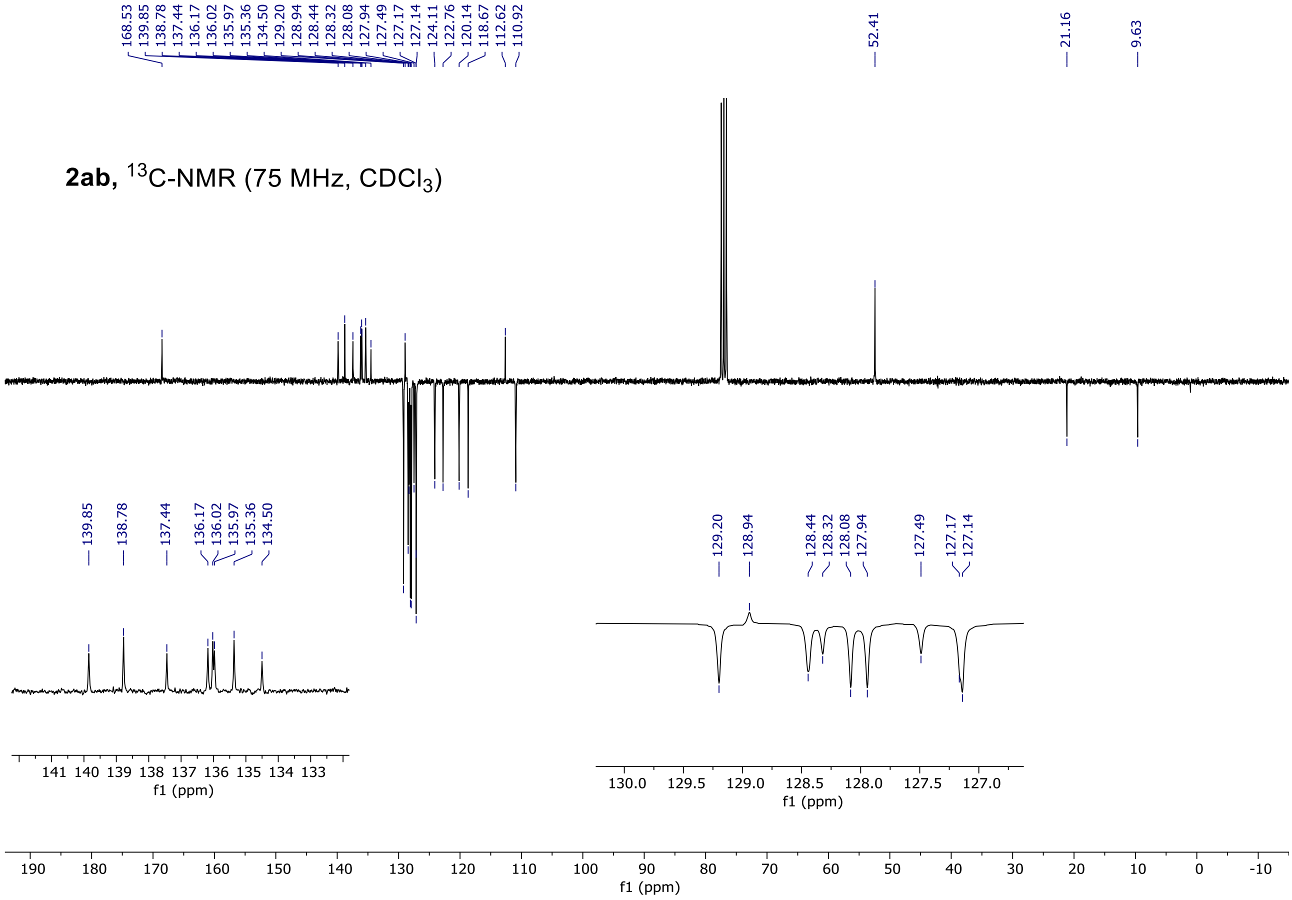

2ab, ^{13}C-NMR (75 MHz, $CDCl_3$)

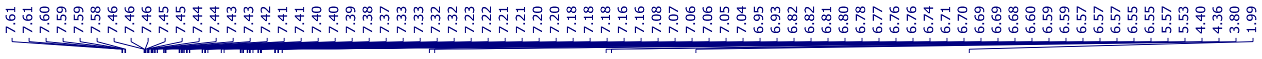

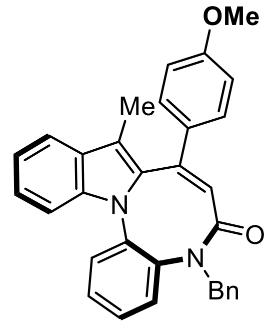

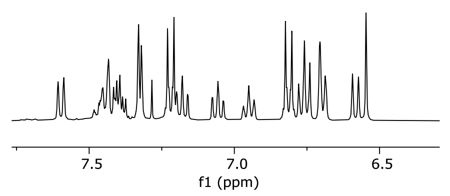

2ac, ^{1}H NMR (400 MHz, $CDCl_3$)

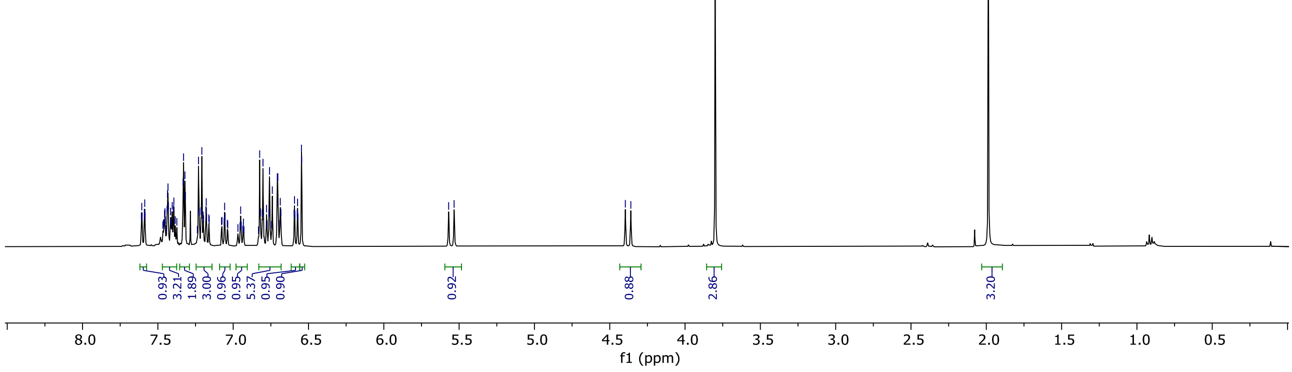

2ac, ^{13}C-NMR (101 MHz, $CDCl_3$)

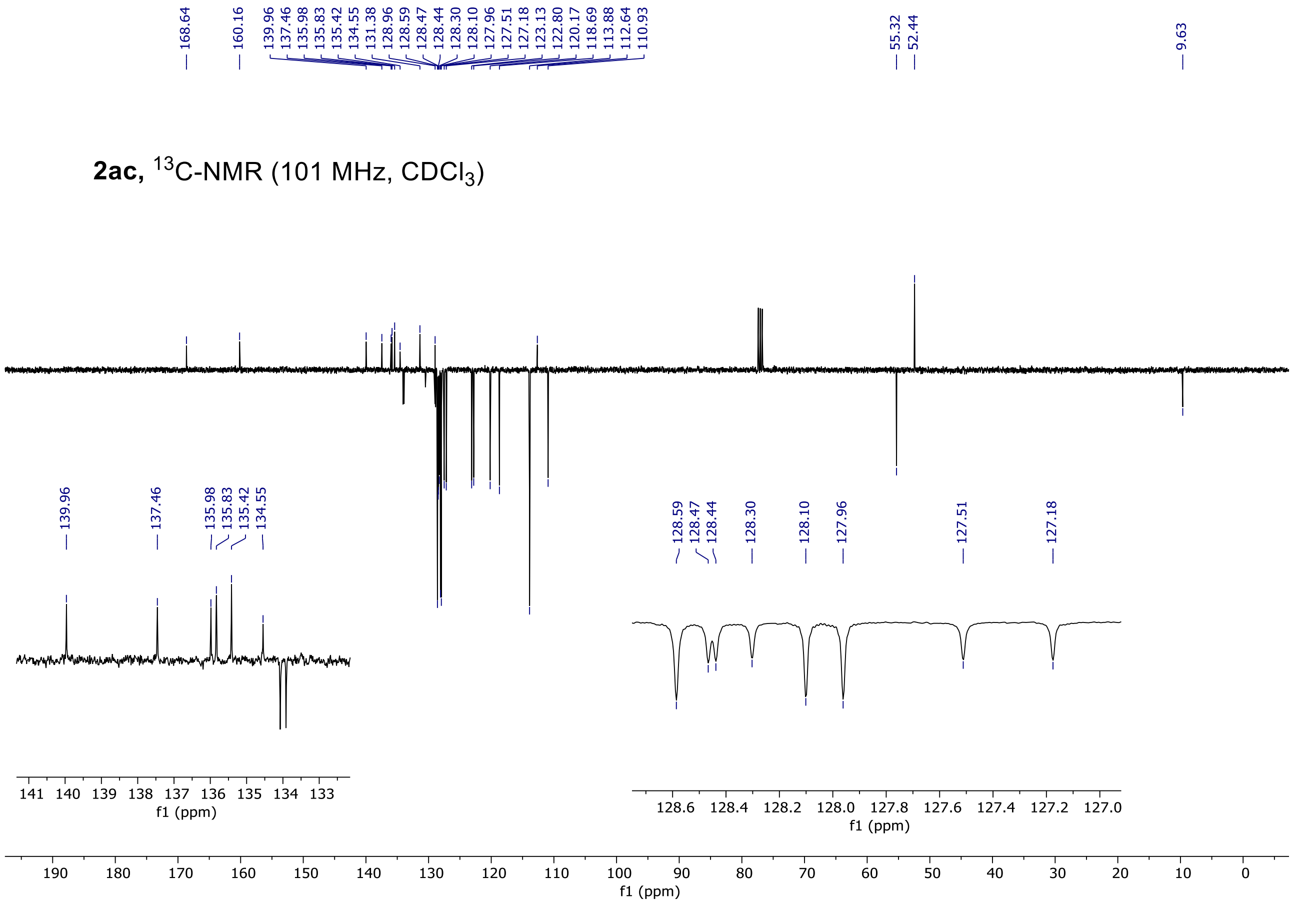

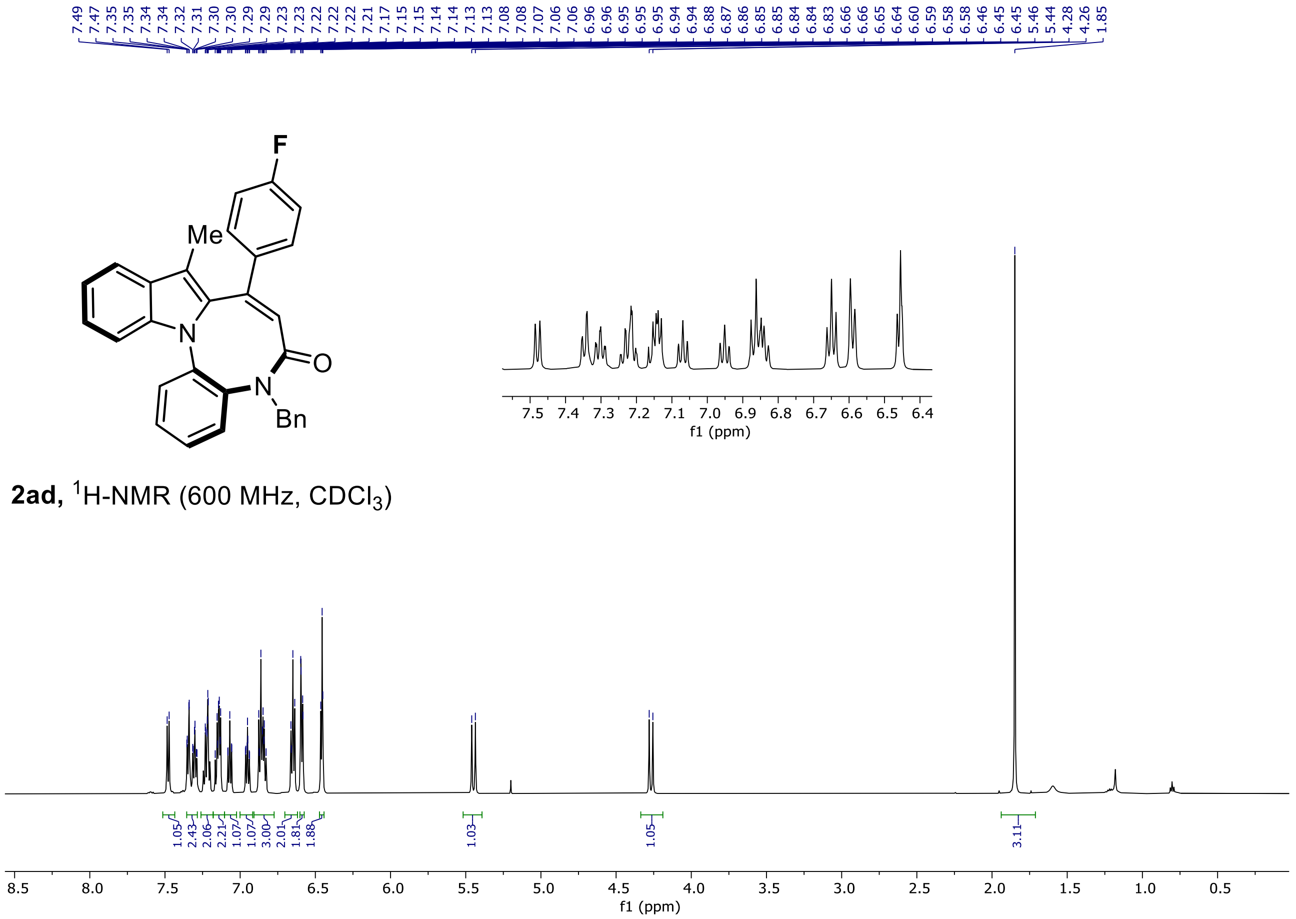

2ad, ^{1}H-NMR (600 MHz, $CDCl_3$)

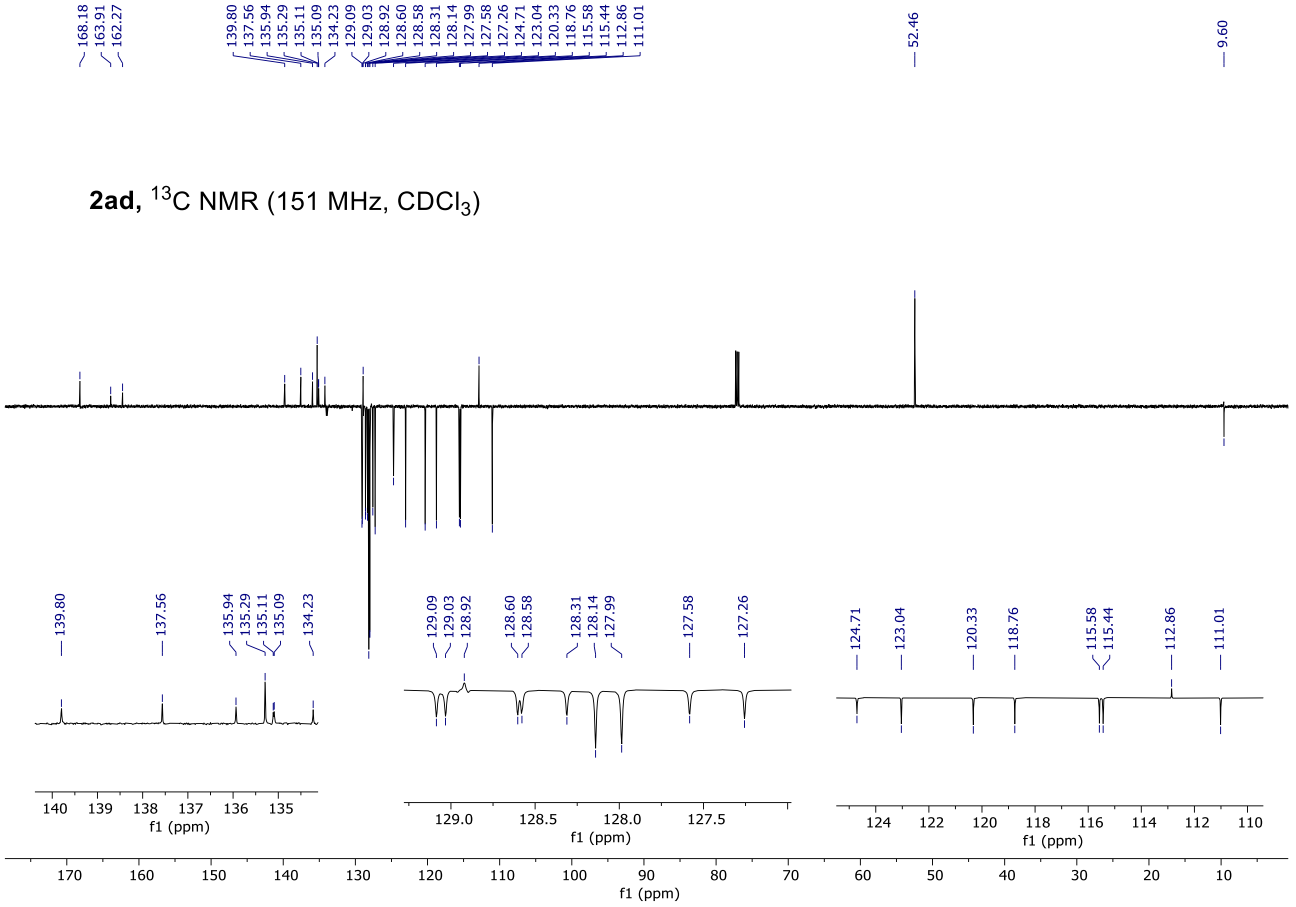

2ad, ^{13}C NMR (151 MHz, $CDCl_3$)

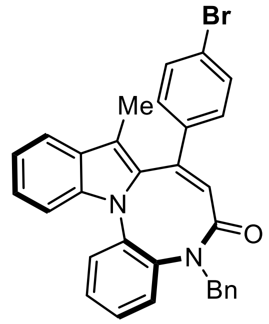

2ae

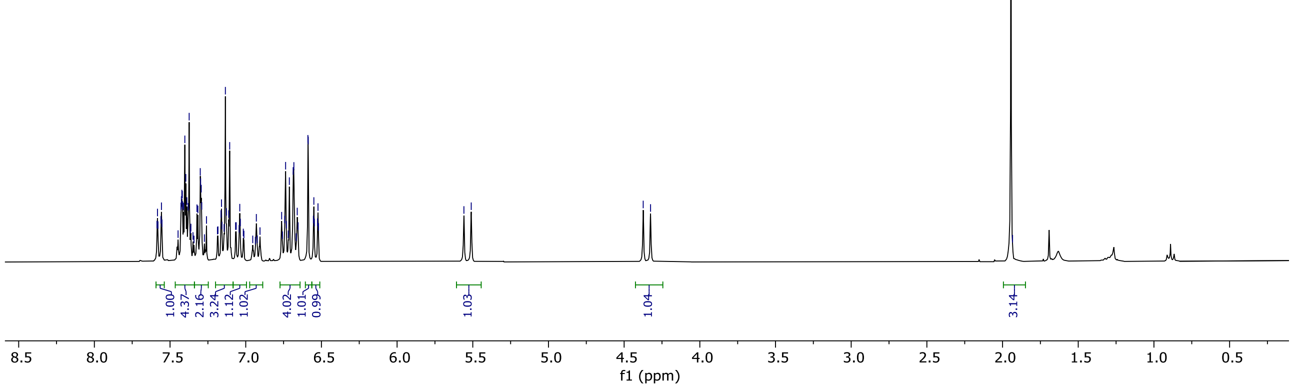

2ae, ^{1}H NMR (300 MHz, $CDCl_3$)

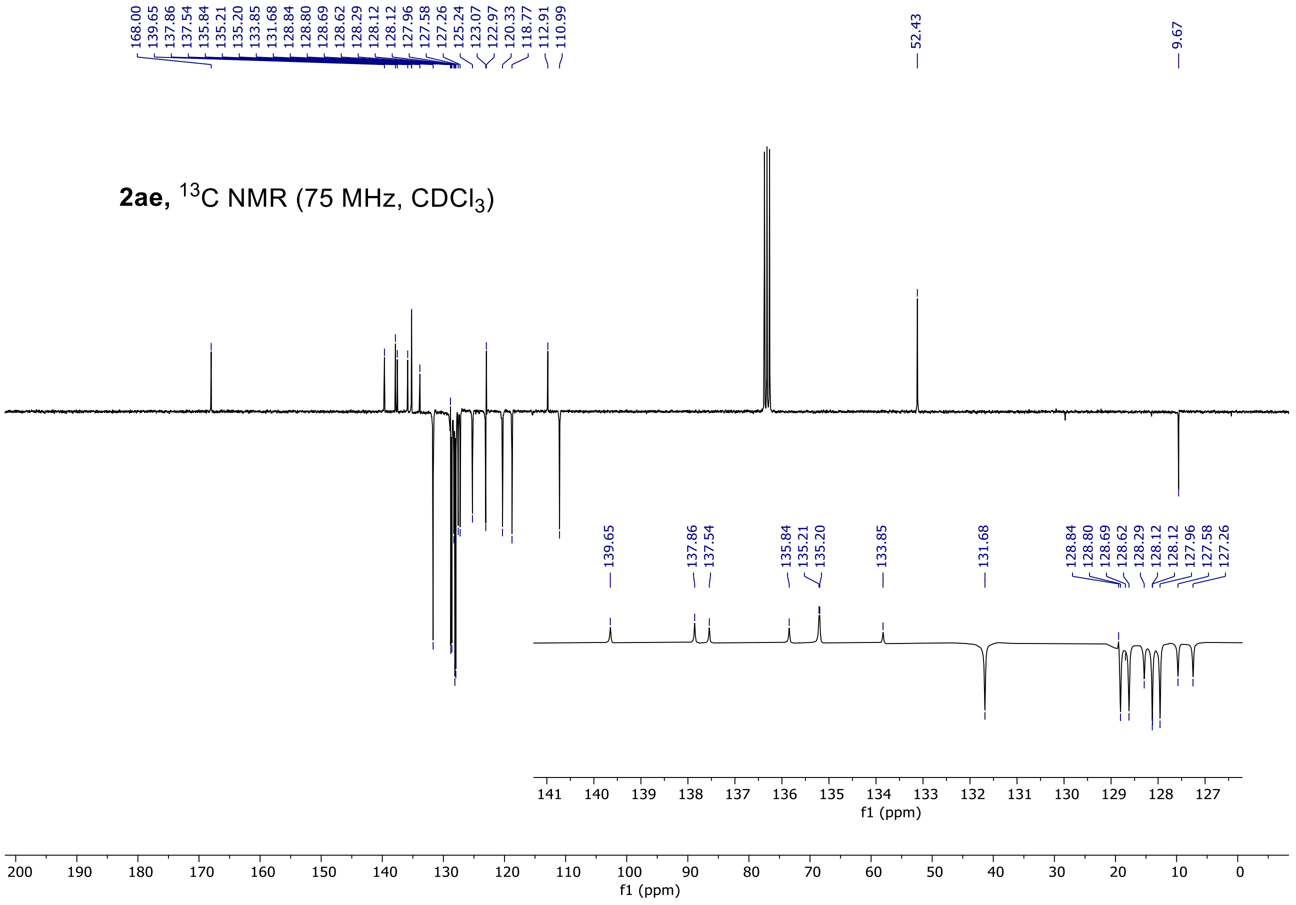

2ae, ^{13}C NMR (75 MHz, $CDCl_3$)

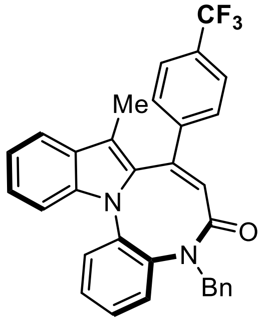

2af, ^{1}H NMR (300 MHz, $CDCl_3$)

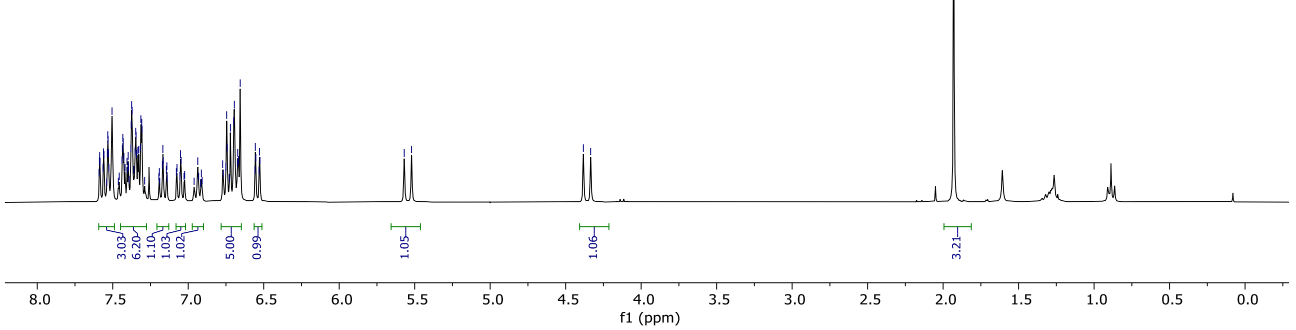

2af, ^{13}C NMR (75 MHz, $CDCl_3$)

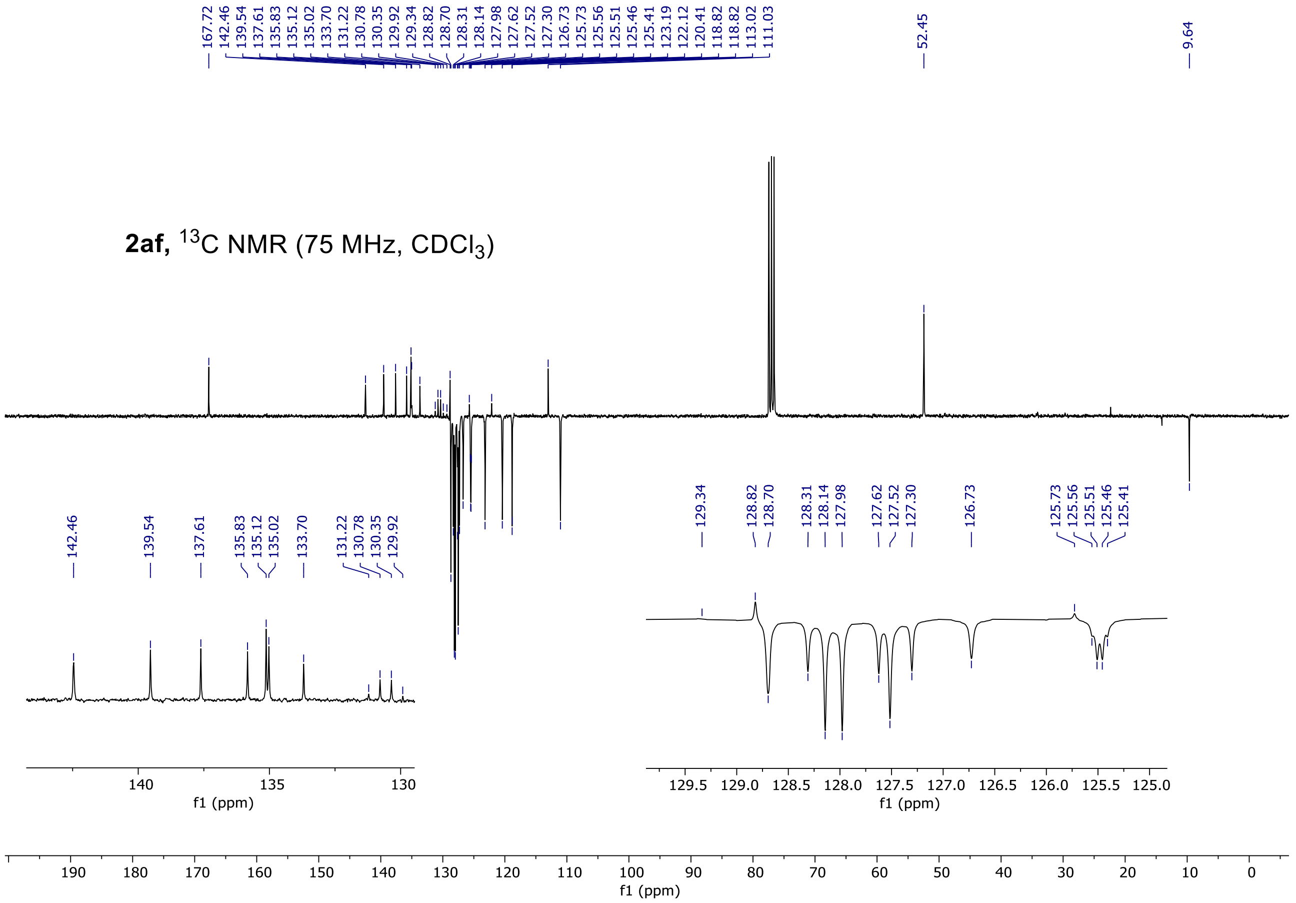

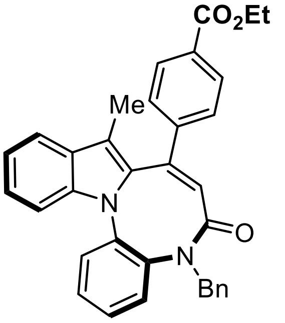

2ag, ^{1}H NMR (400 MHz, $CDCl_3$)

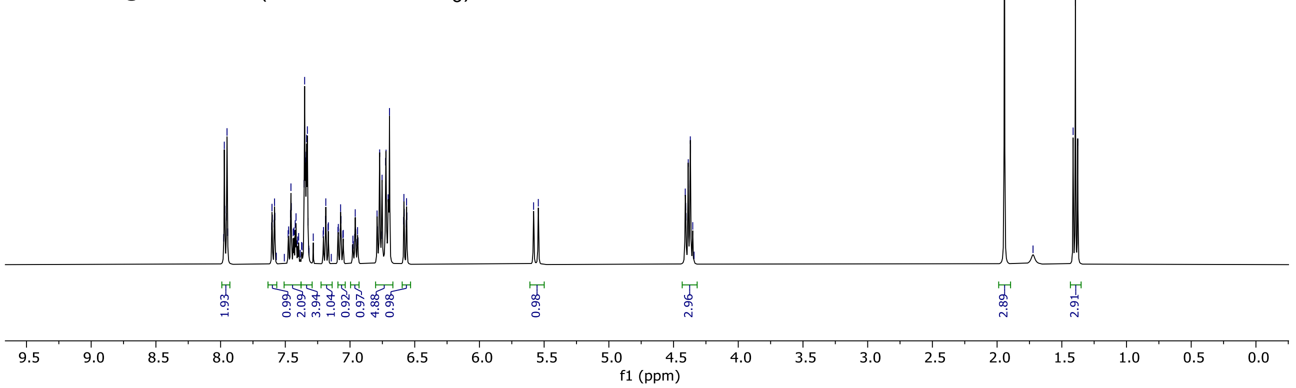

2ag, ^{13}C NMR (101 MHz, $CDCl_3$)

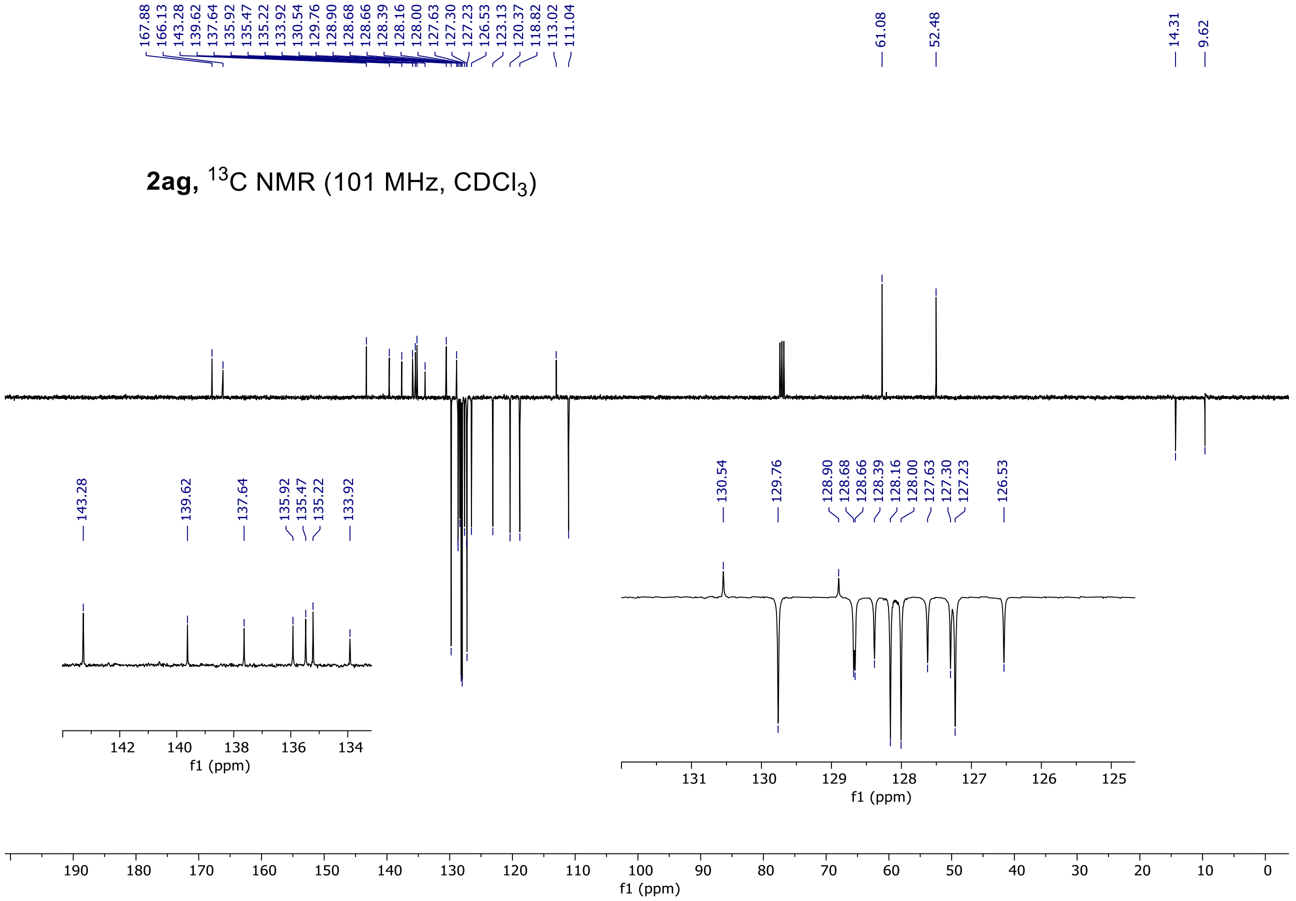

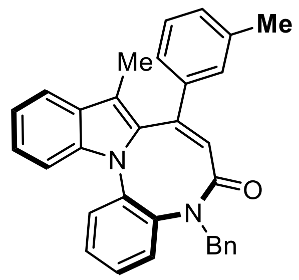

2ah, ^{1}H NMR (300 MHz, $CDCl_3$)

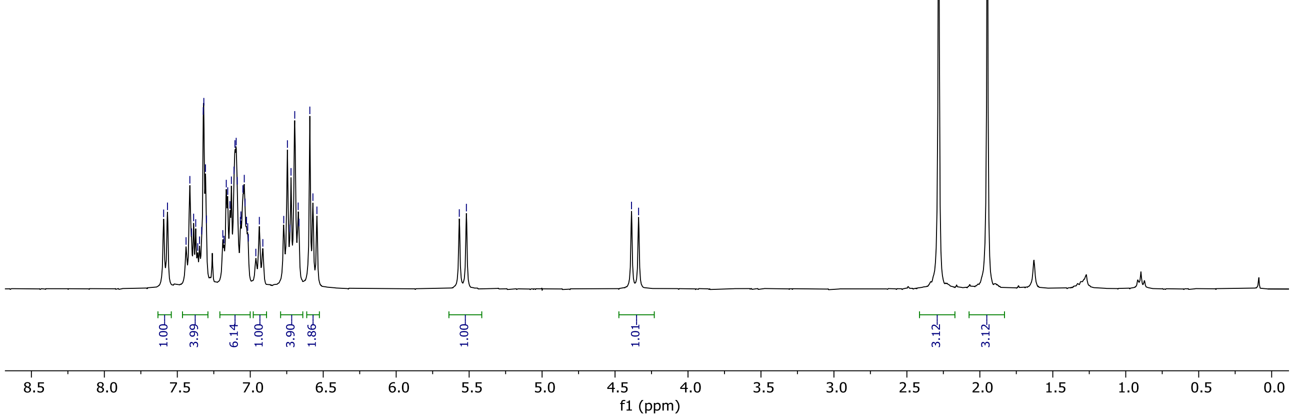

2ah, ^{13}C NMR (75 MHz, $CDCl_3$)

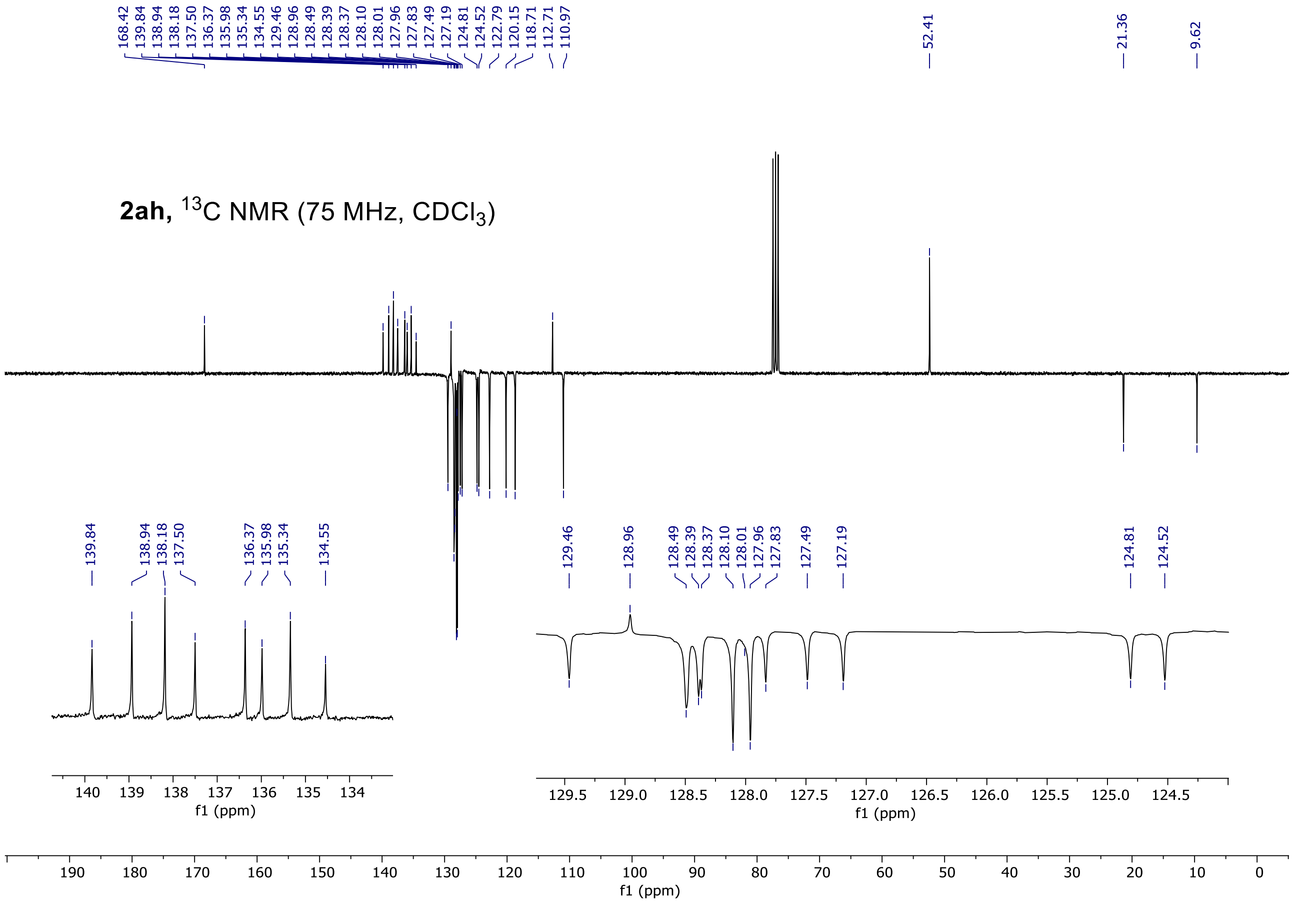

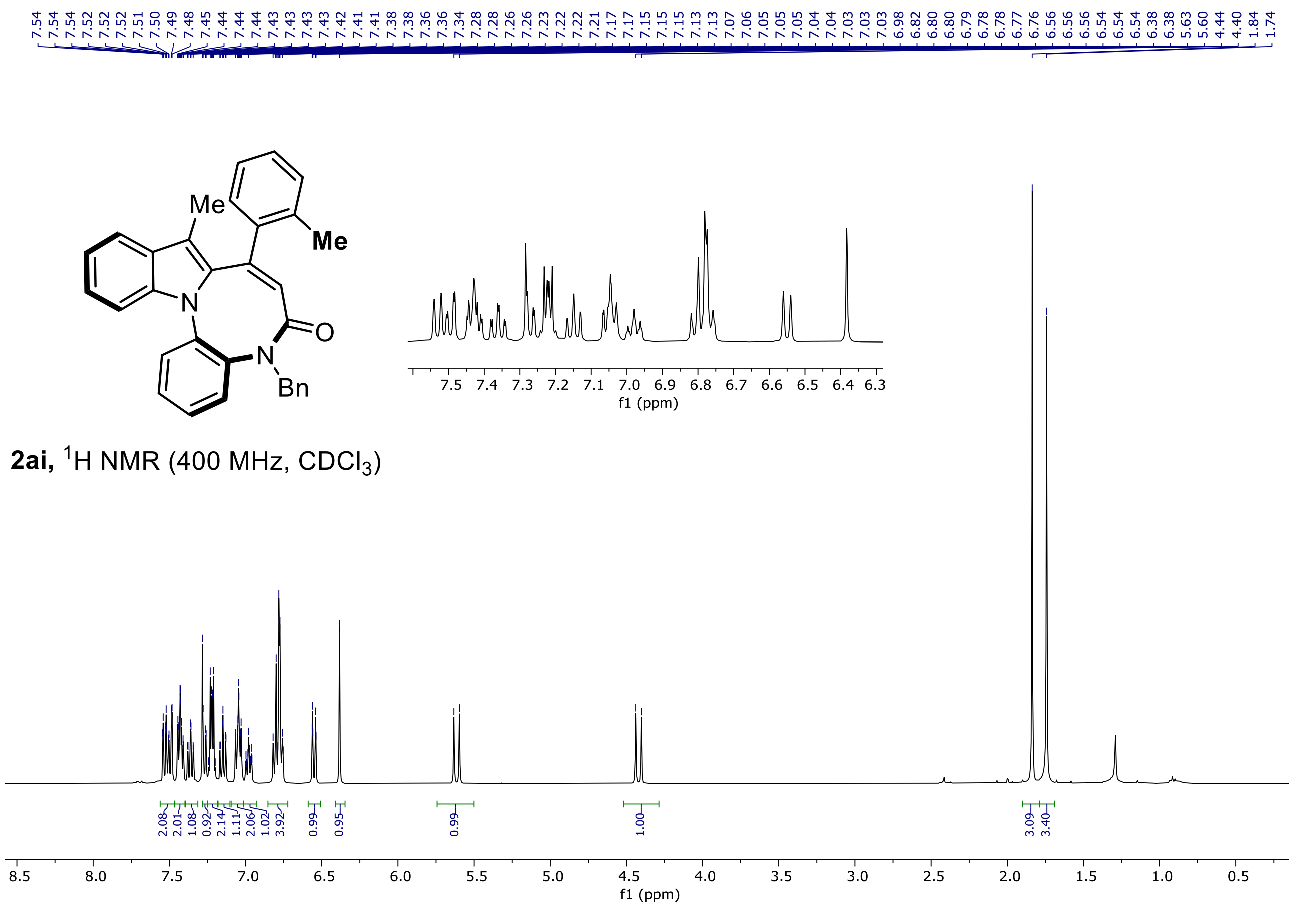

2ai, ^{1}H NMR (400 MHz, $CDCl_3$)

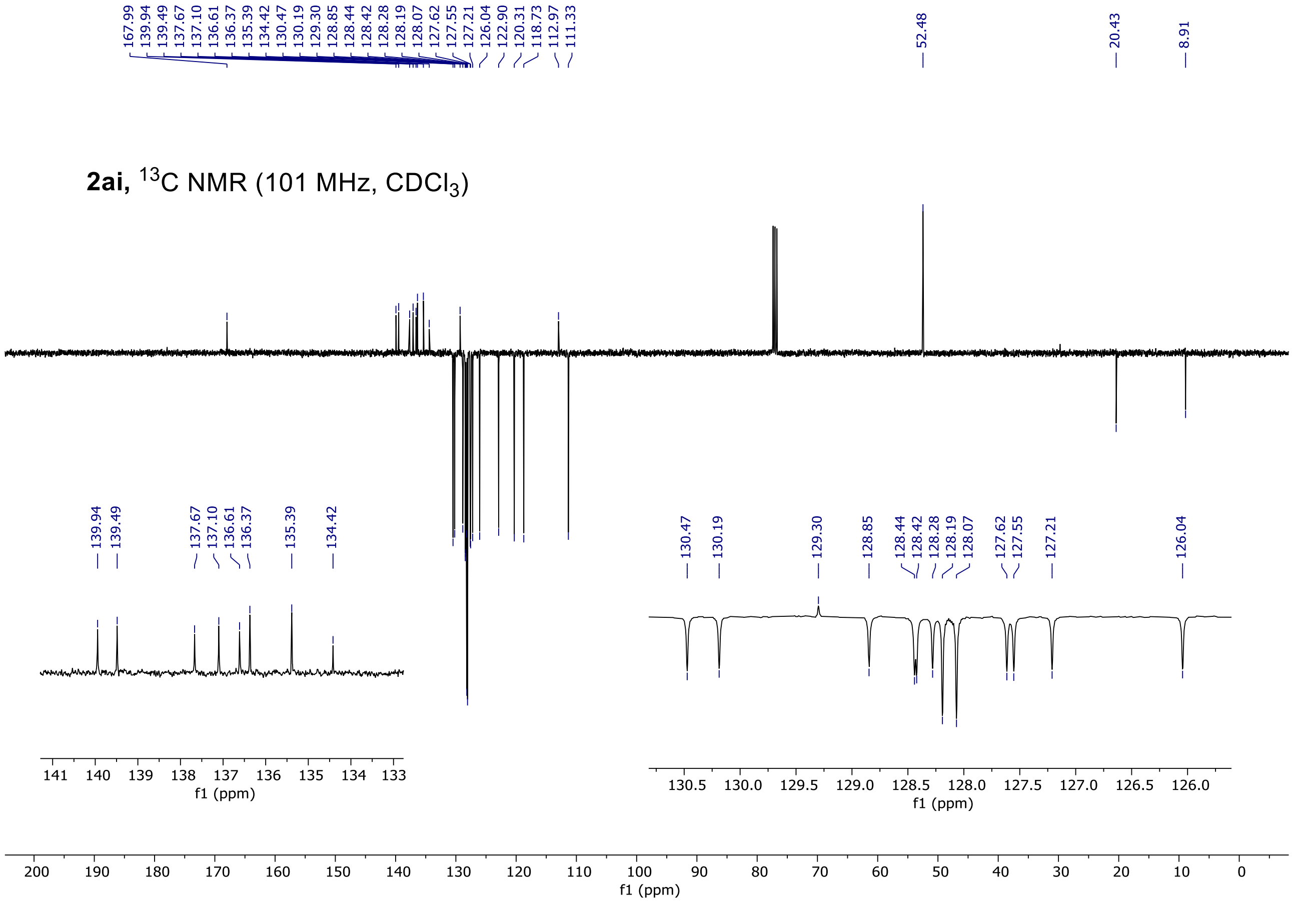

2ai, ^{13}C NMR (101 MHz, $CDCl_3$)

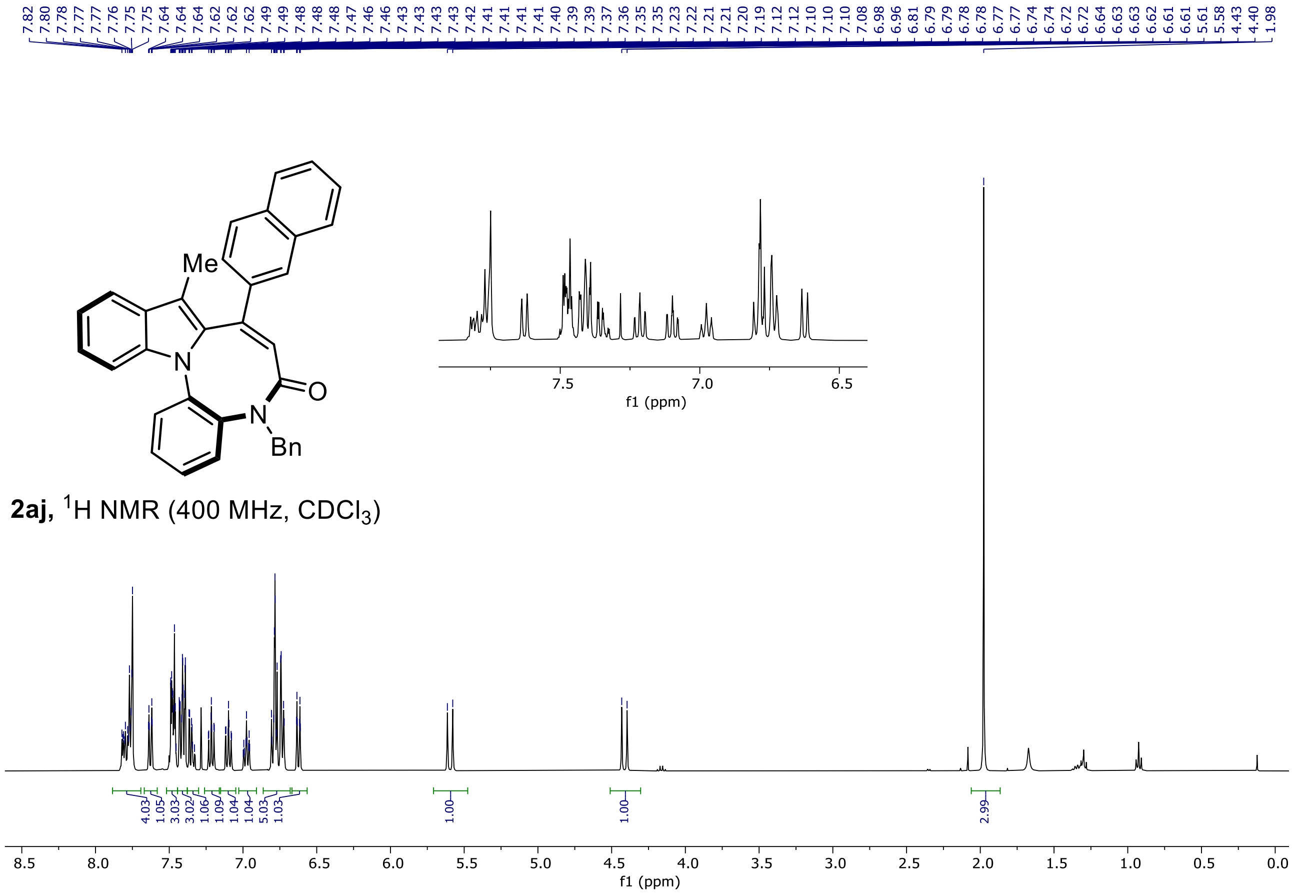

2aj, ^{1}H NMR (400 MHz, $CDCl_3$)

2aj, ^{13}C NMR (101 MHz, $CDCl_3$)

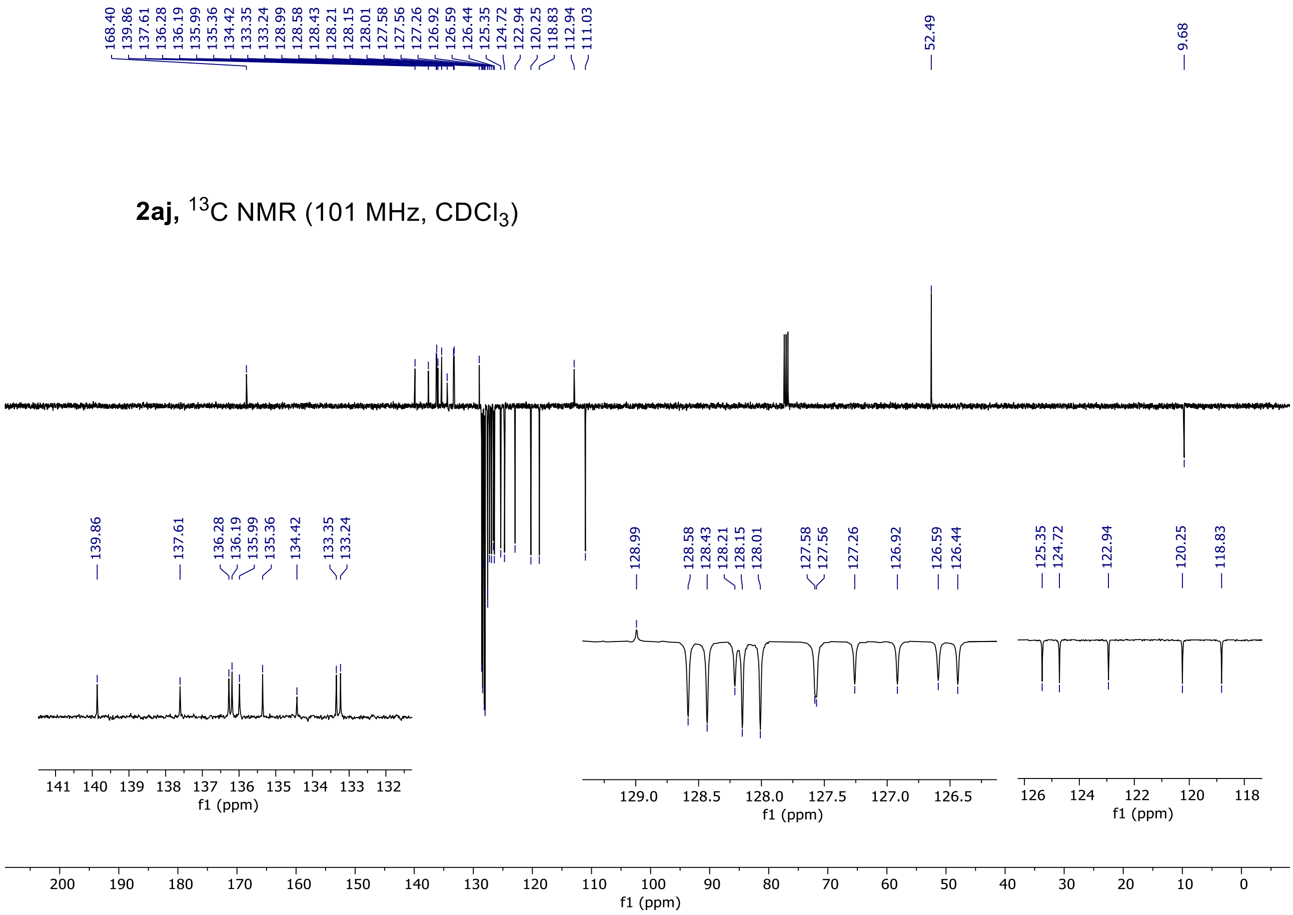

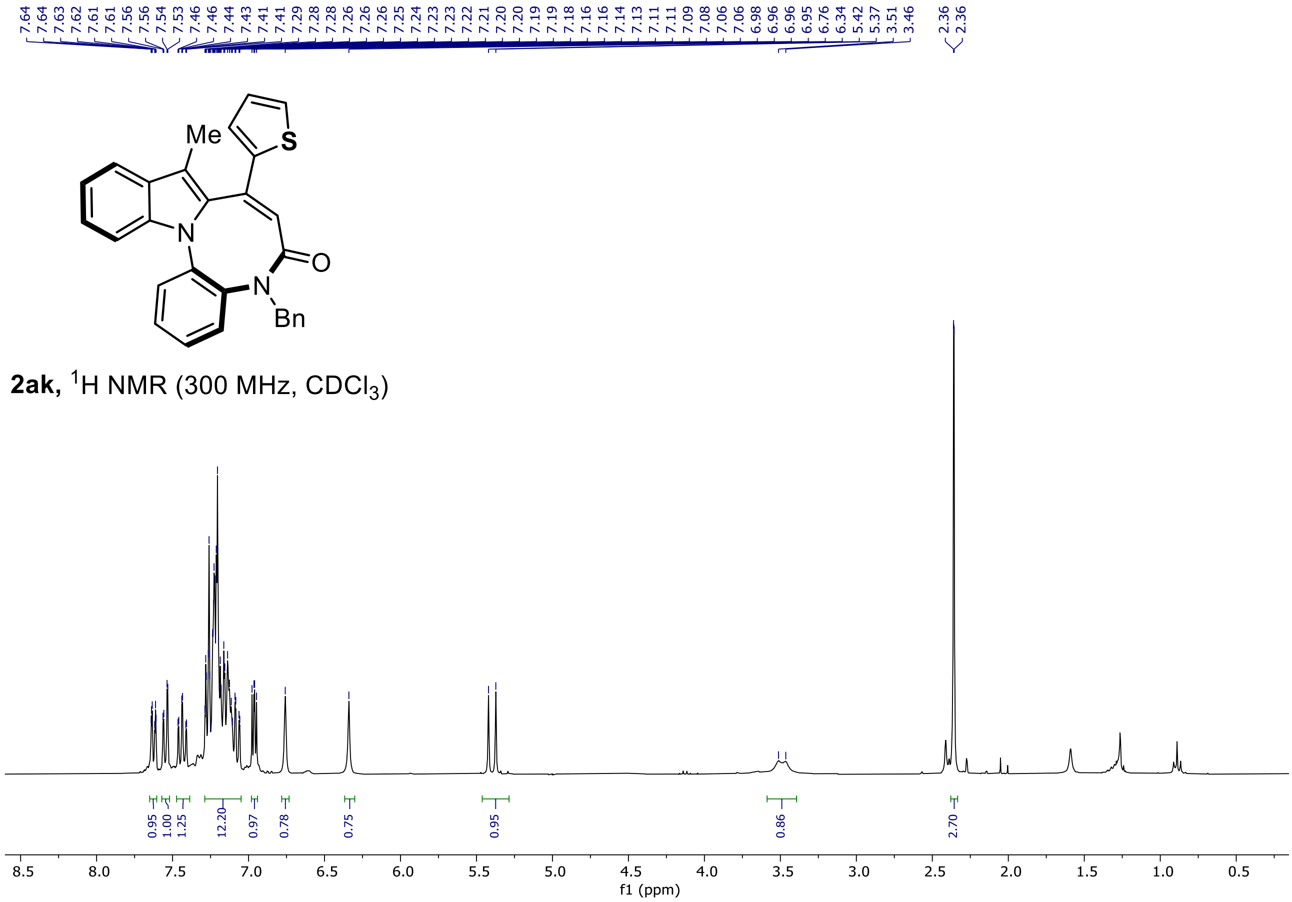

2ak, ^{1}H NMR (300 MHz, $CDCl_3$)

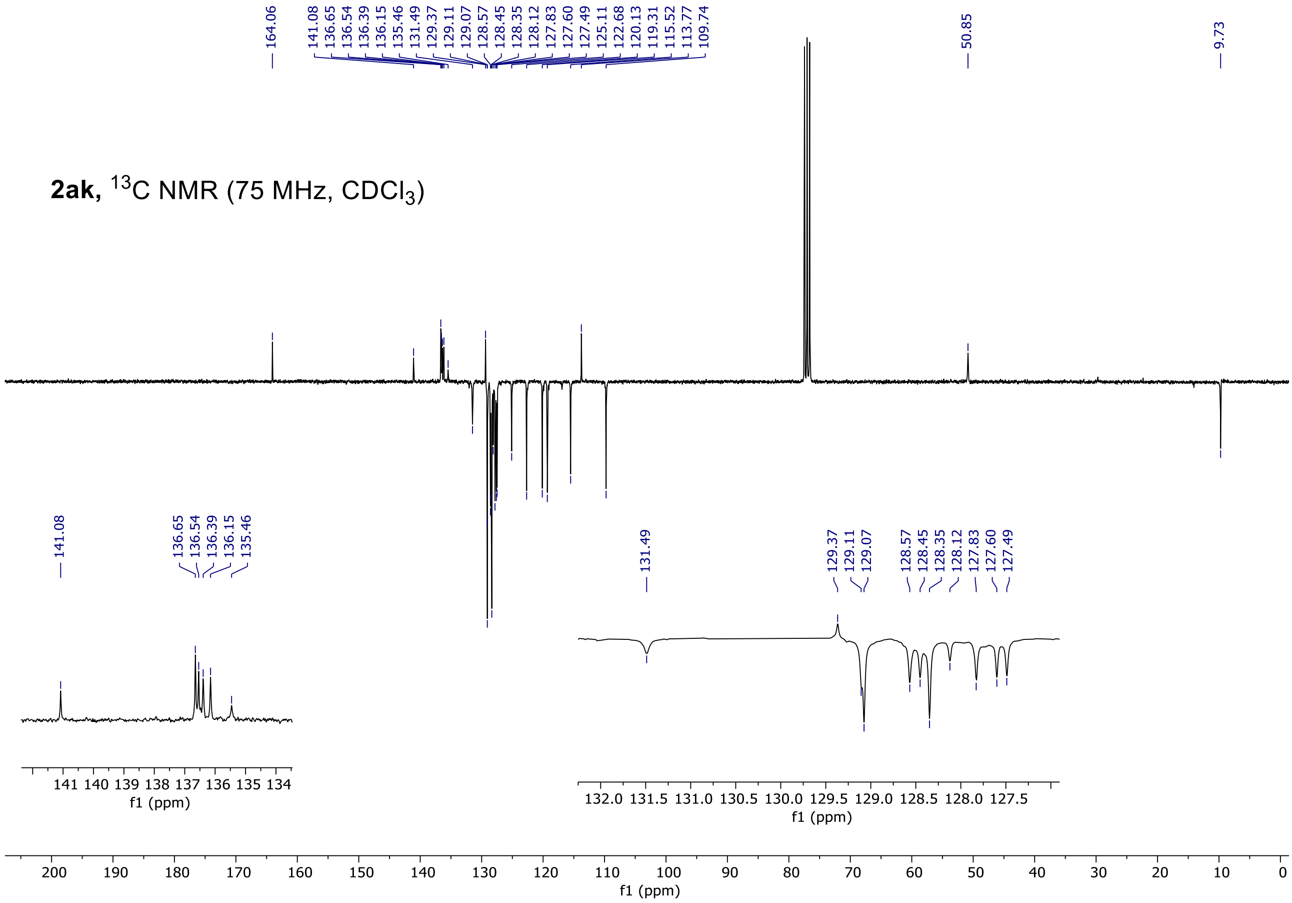

2ak, ^{13}C NMR (75 MHz, $CDCl_3$)

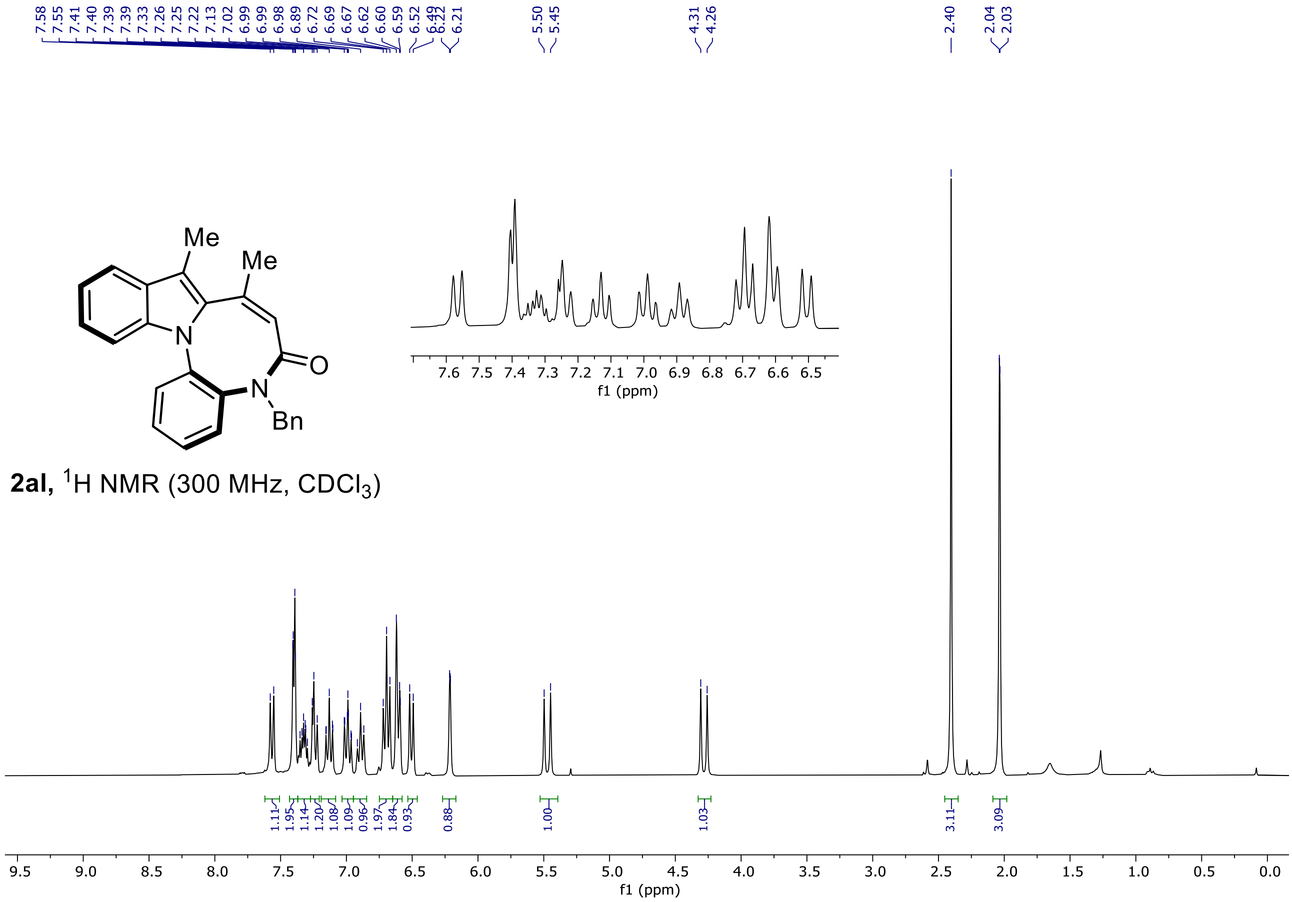

2al, ^{1}H NMR (300 MHz, $CDCl_3$)

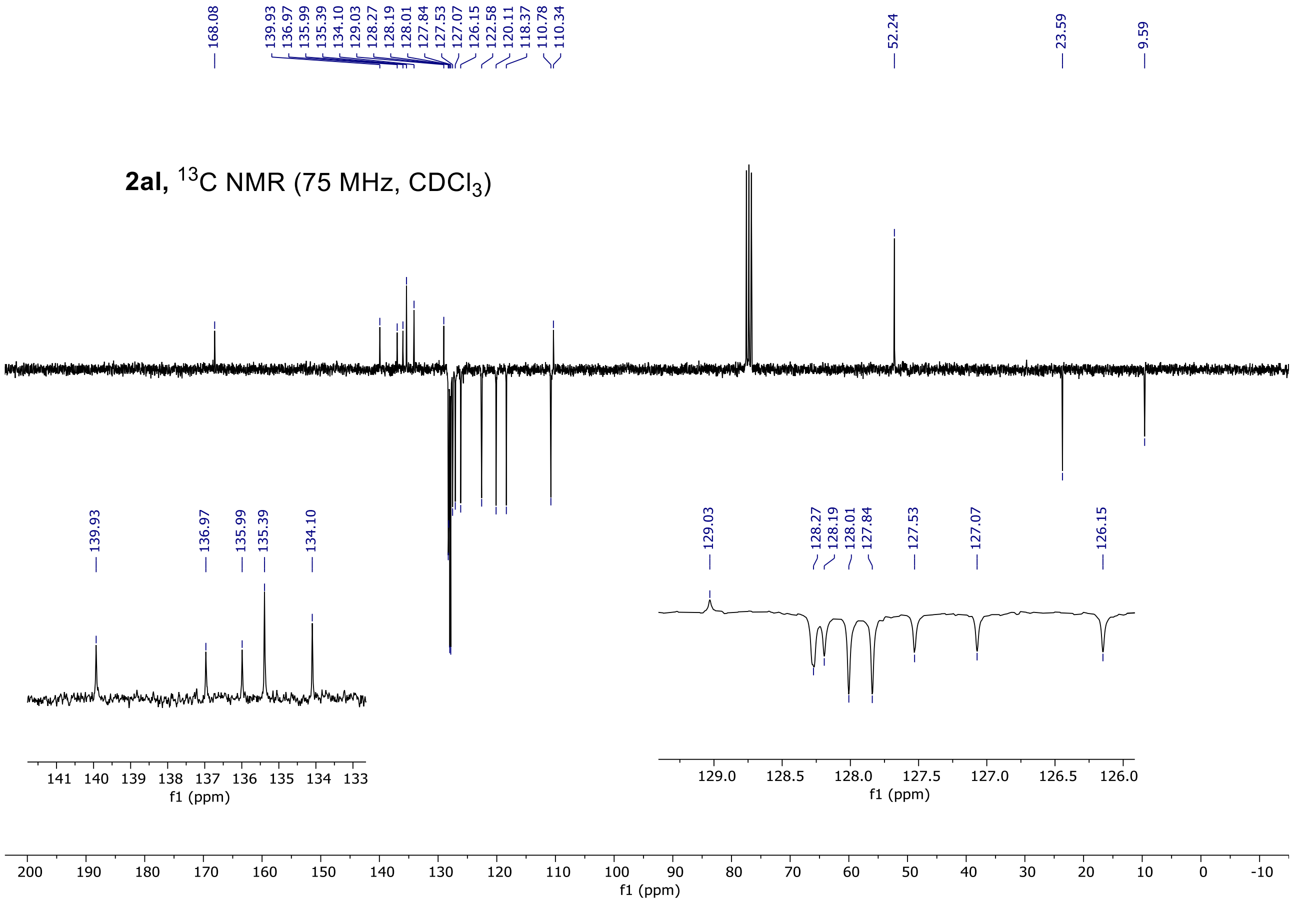

2al, ^{13}C NMR (75 MHz, $CDCl_3$)

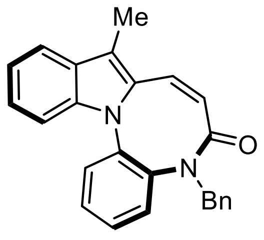

2am, ^{1}H NMR (300 MHz, $CDCl_3$)

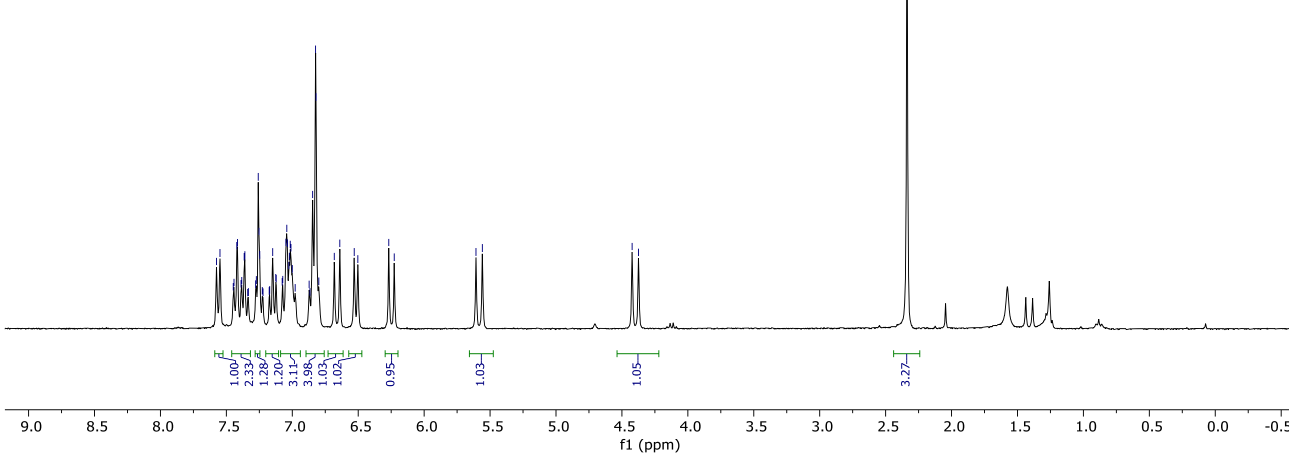

2am, ^{13}C NMR (75 MHz, $CDCl_3$)

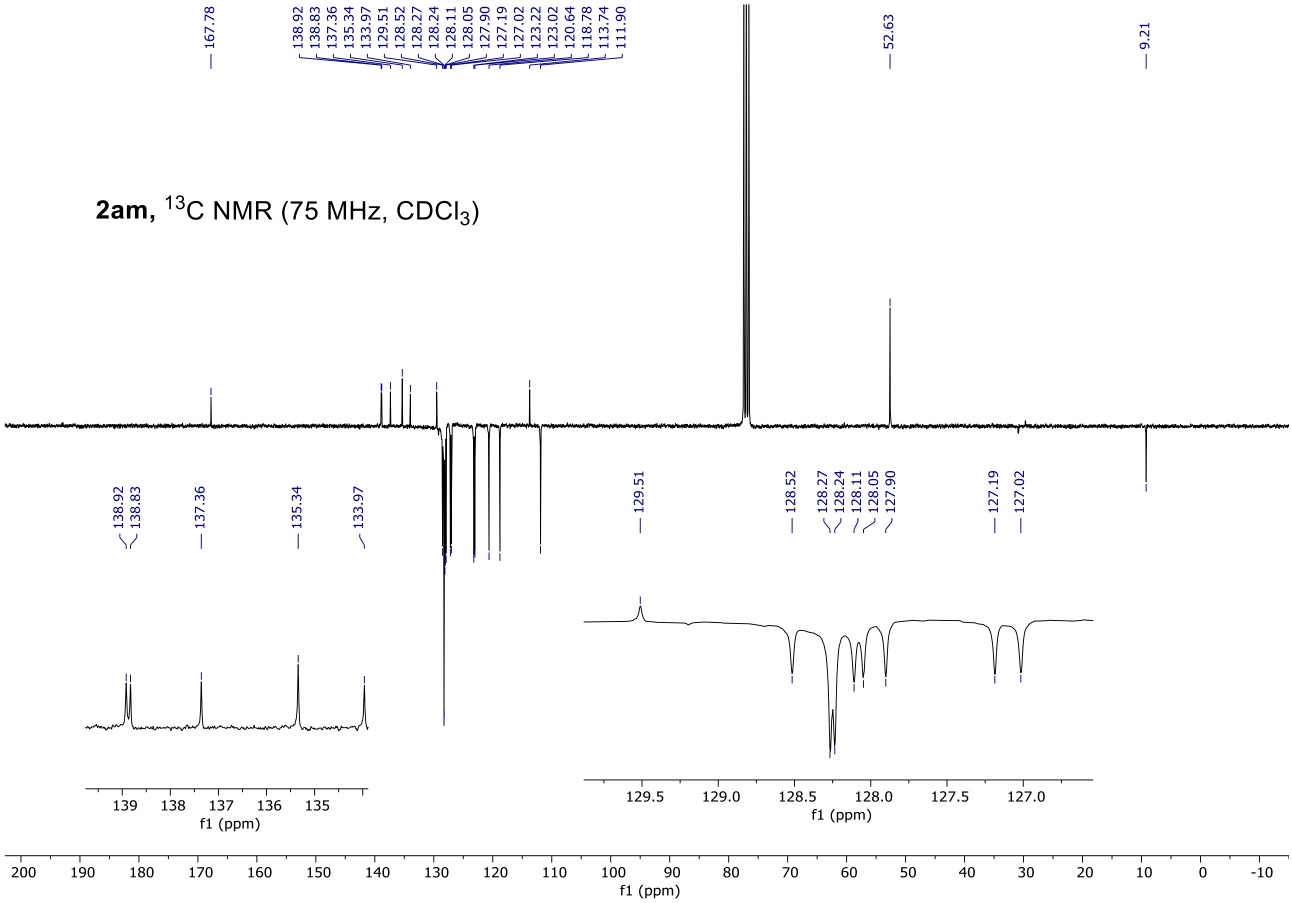

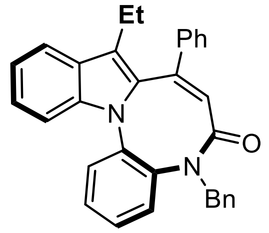

2an, ^{1}H NMR (400 MHz, $CDCl_3$)

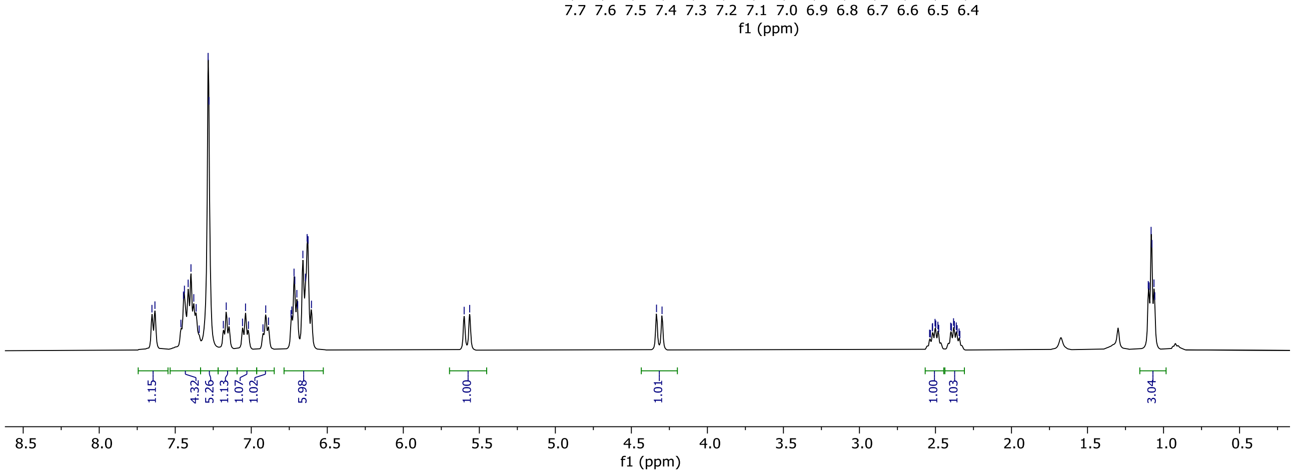

2an, ^{13}C NMR (101 MHz, $CDCl_3$)

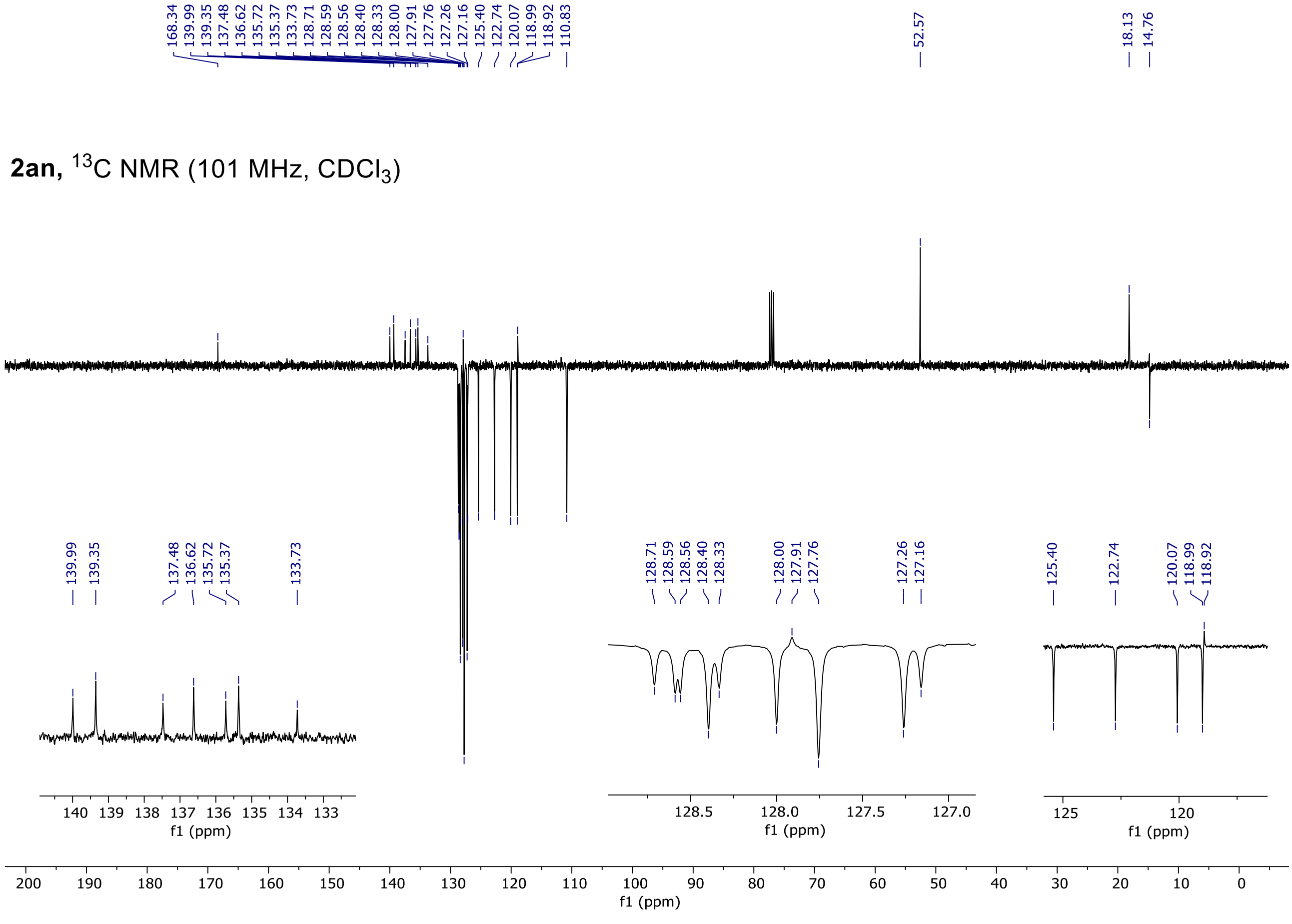

2an, HSQC ($CDCl_3$)

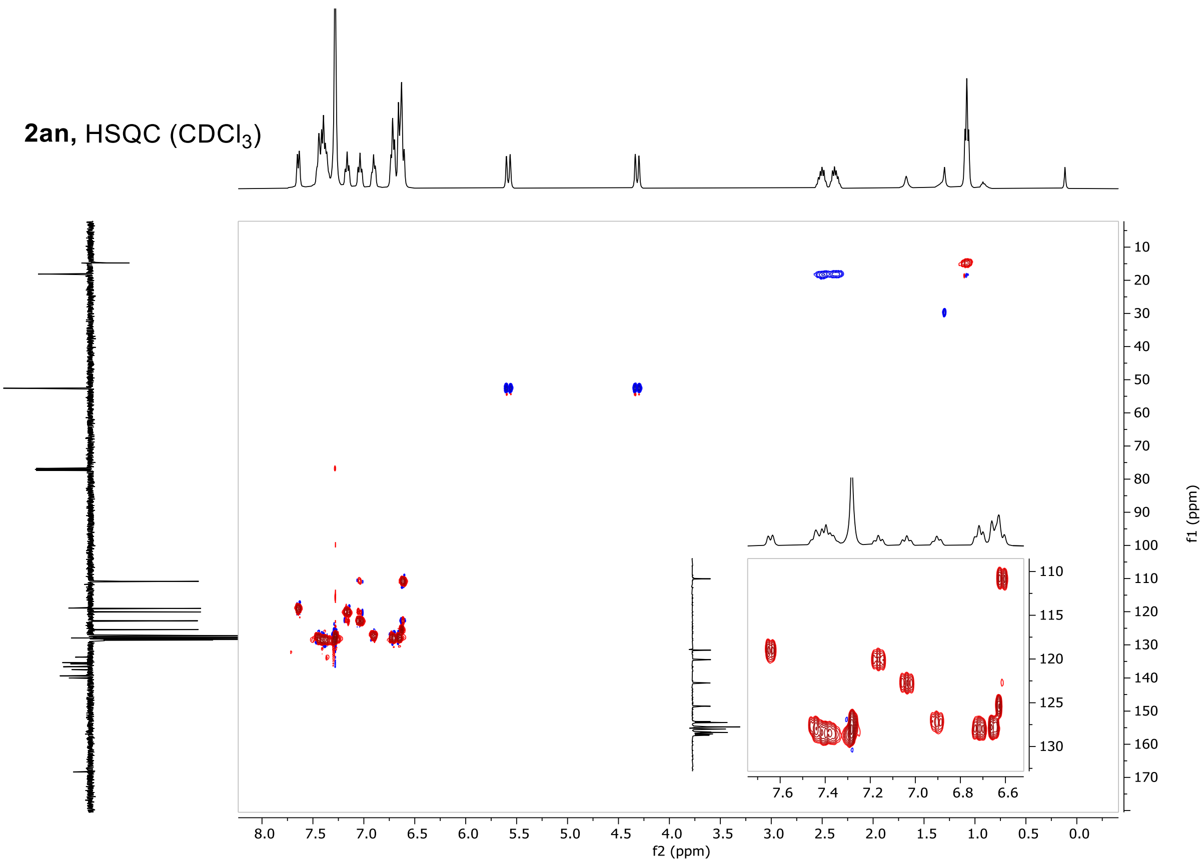

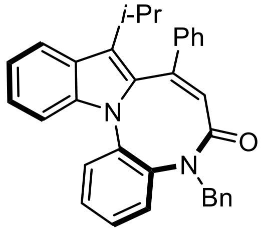

2ao, ^{1}H NMR (300 MHz, $CDCl_3$)

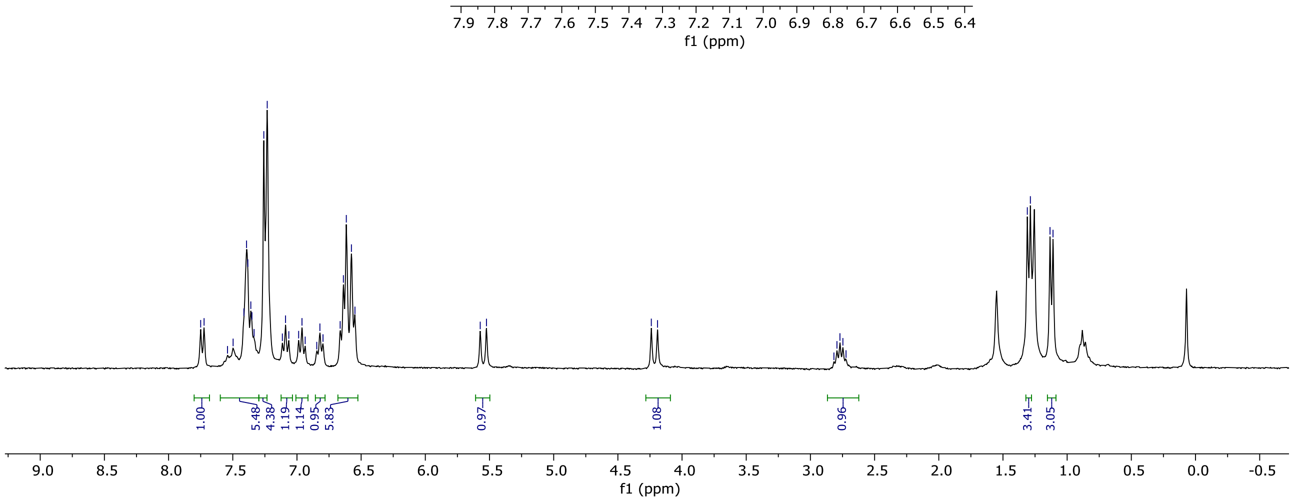

2ao, ^{13}C NMR (75 MHz, $CDCl_3$)

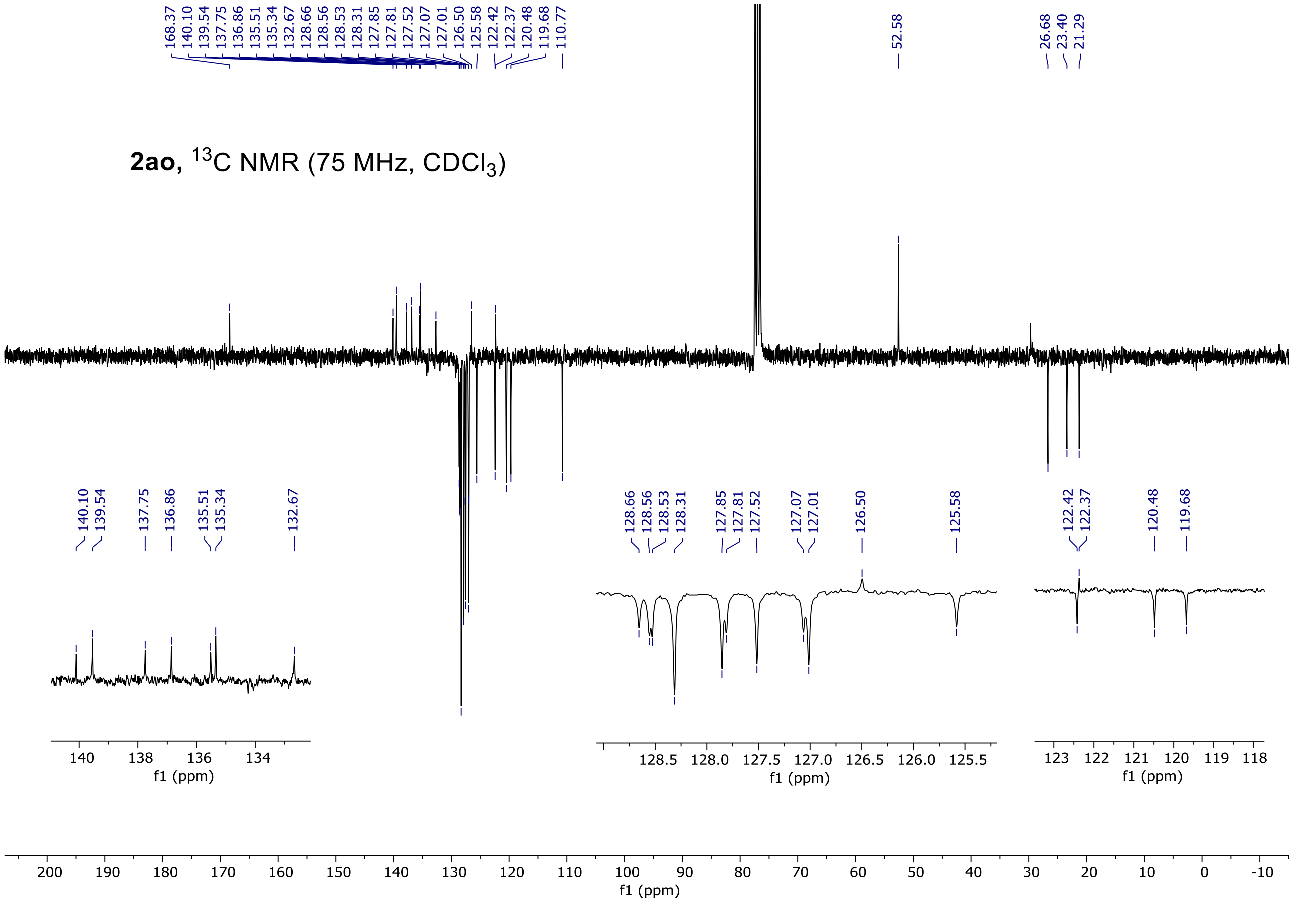

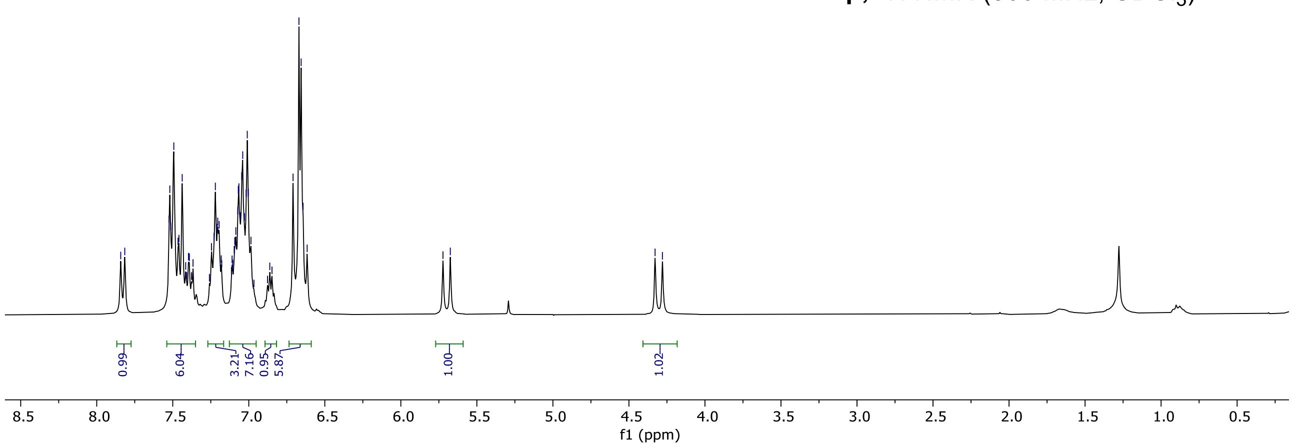

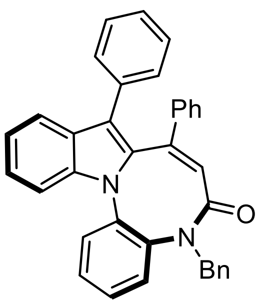

2ap, ^{1}H NMR (300 MHz, $CDCl_3$)

2ap, ^{13}C NMR (75 MHz, $CDCl_3$)

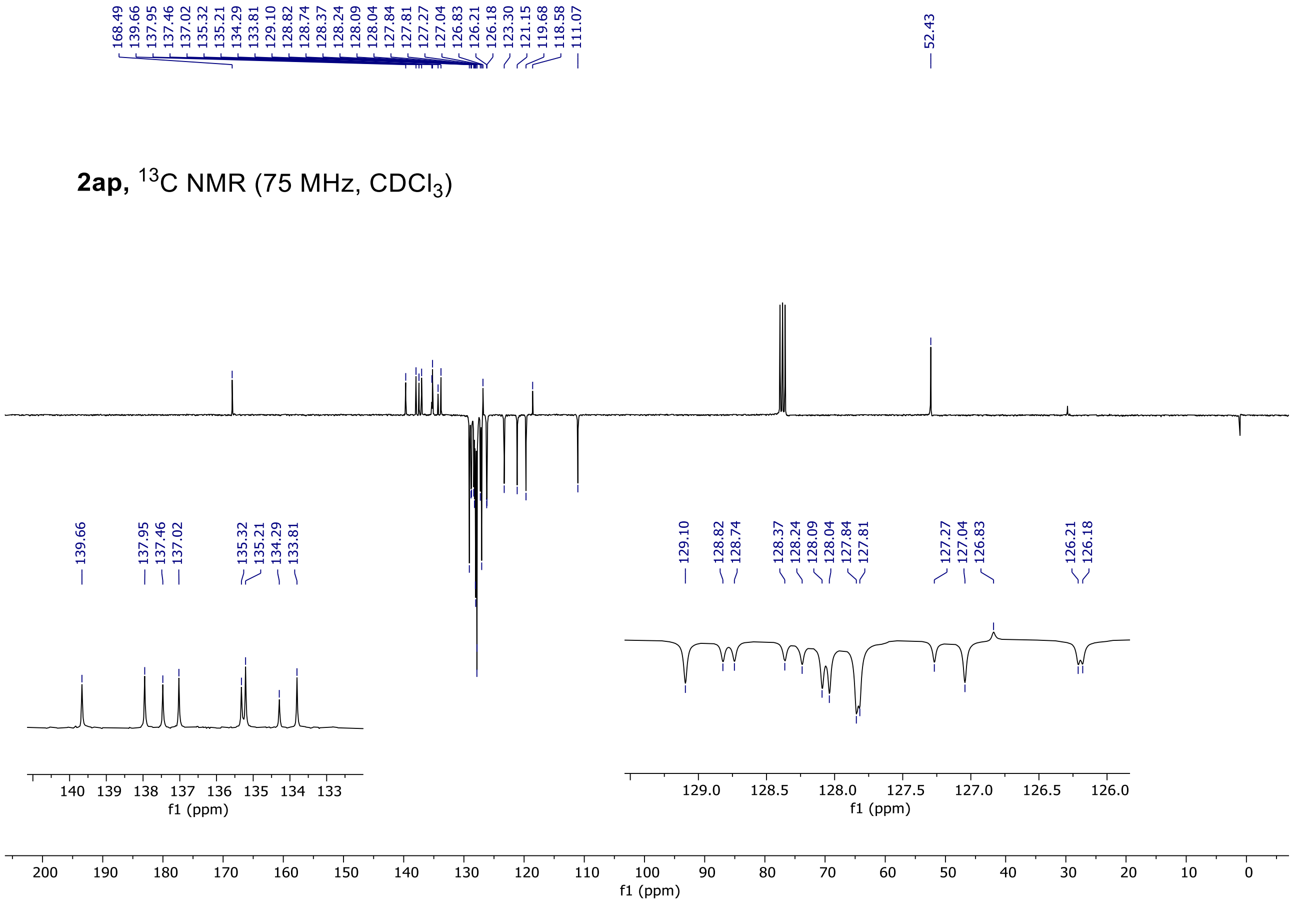

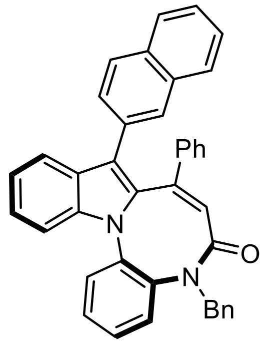

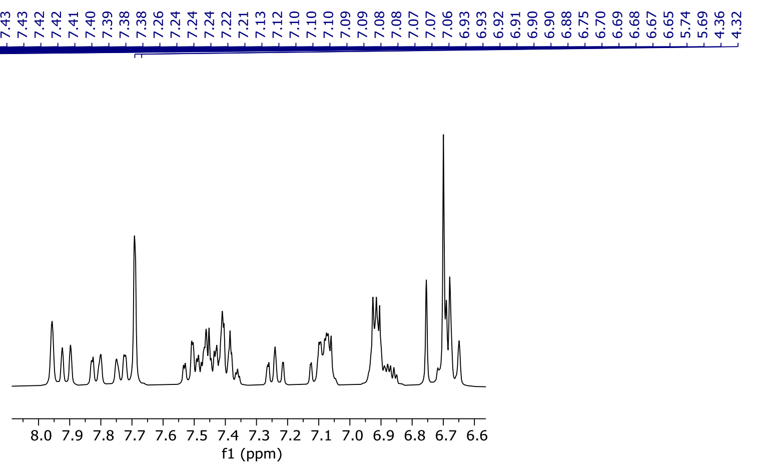

2aq, ^{1}H NMR (300 MHz, $CDCl_3$)

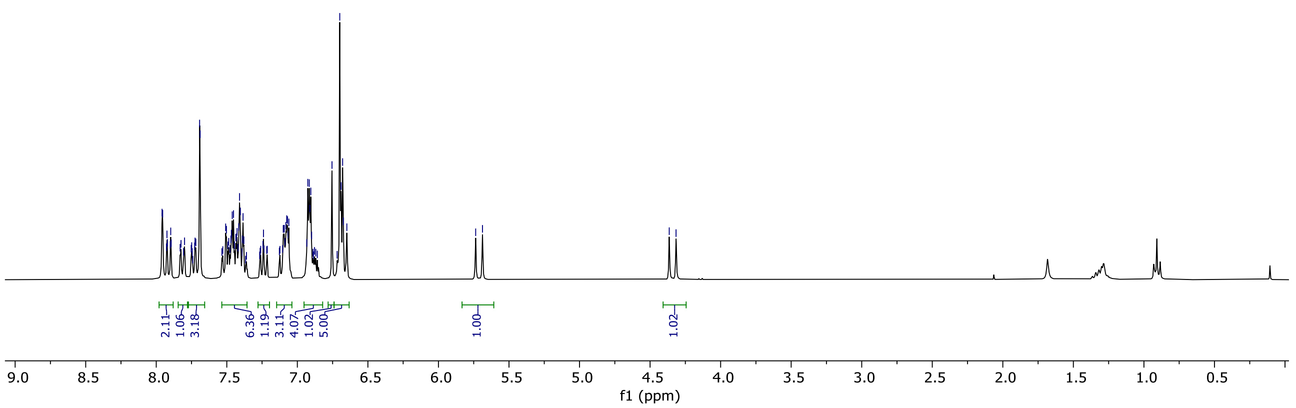

2aq, ^{13}C NMR (75 MHz, $CDCl_3$)

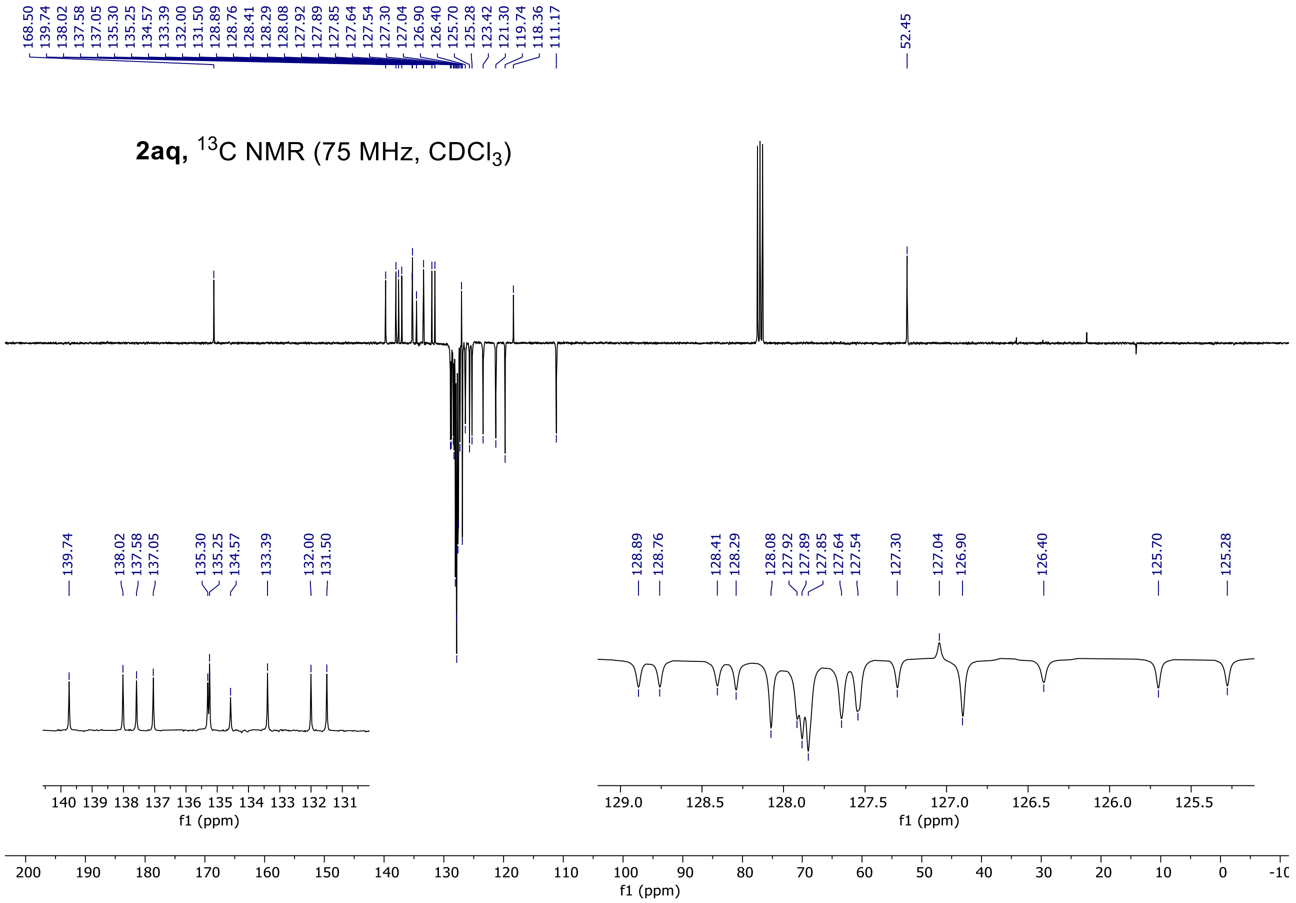

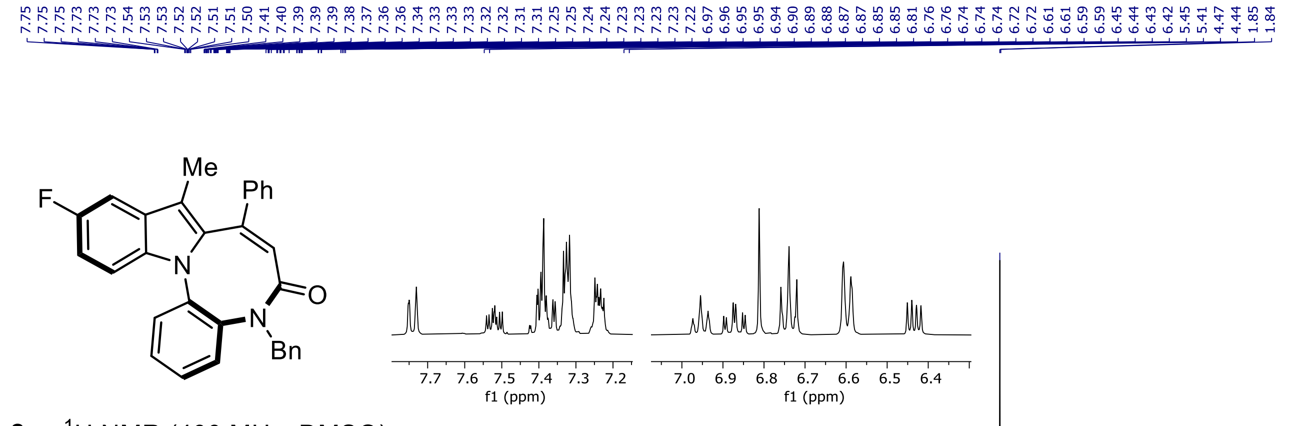

2ar, ^{1}H NMR (400 MHz, DMSO)

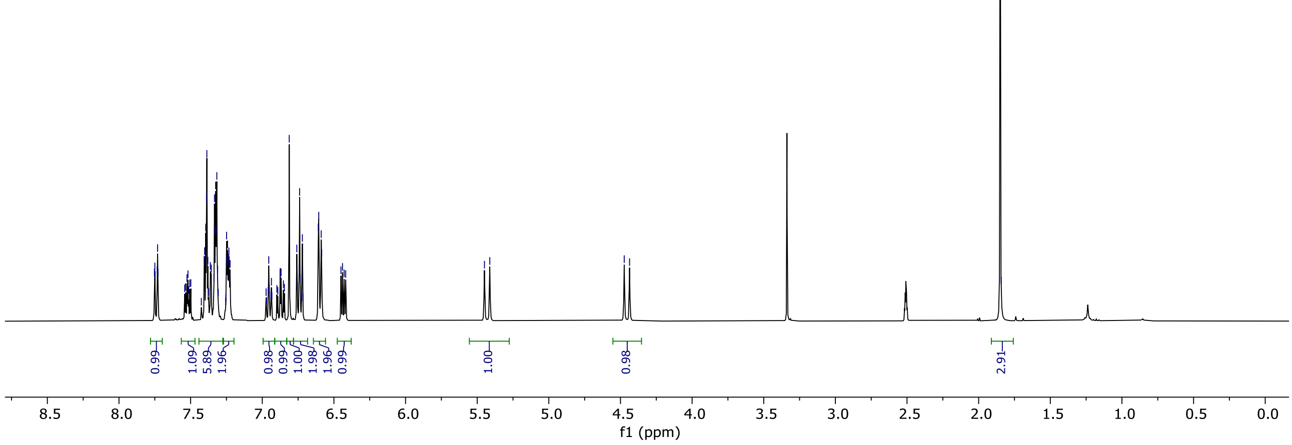

2ar, ^{13}C NMR (101 MHz, DMSO)

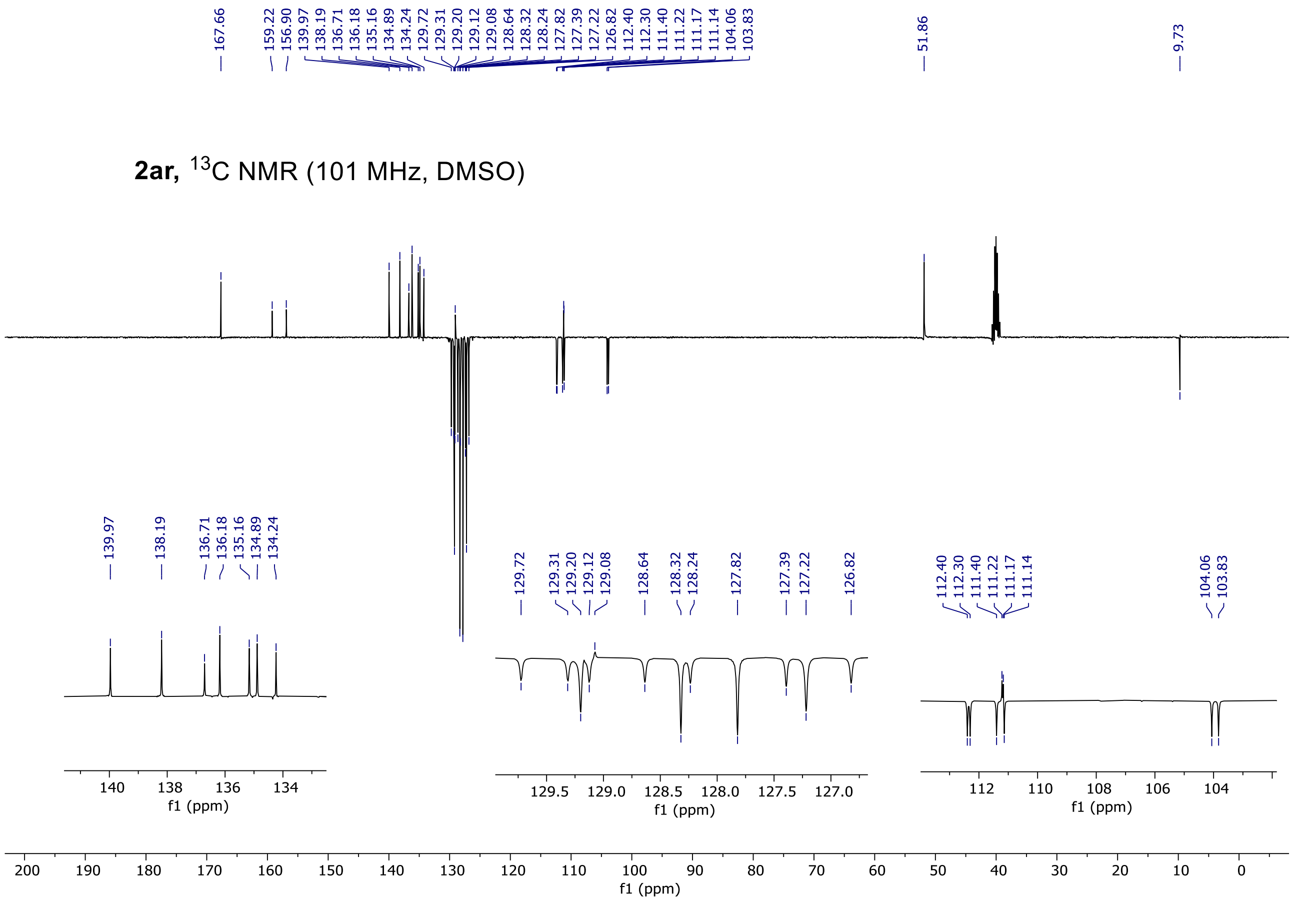

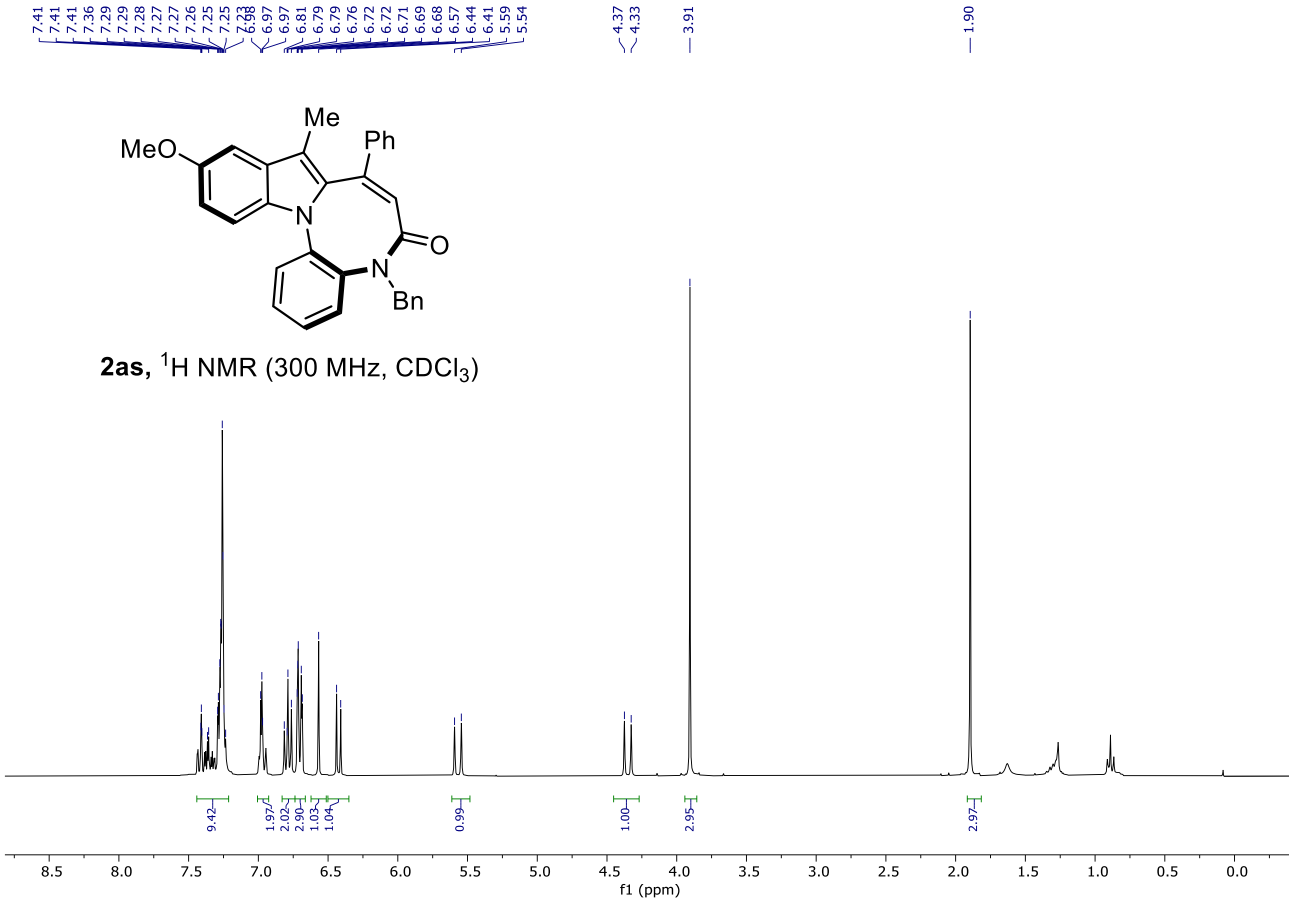

2as, ^{1}H NMR (300 MHz, $CDCl_3$)

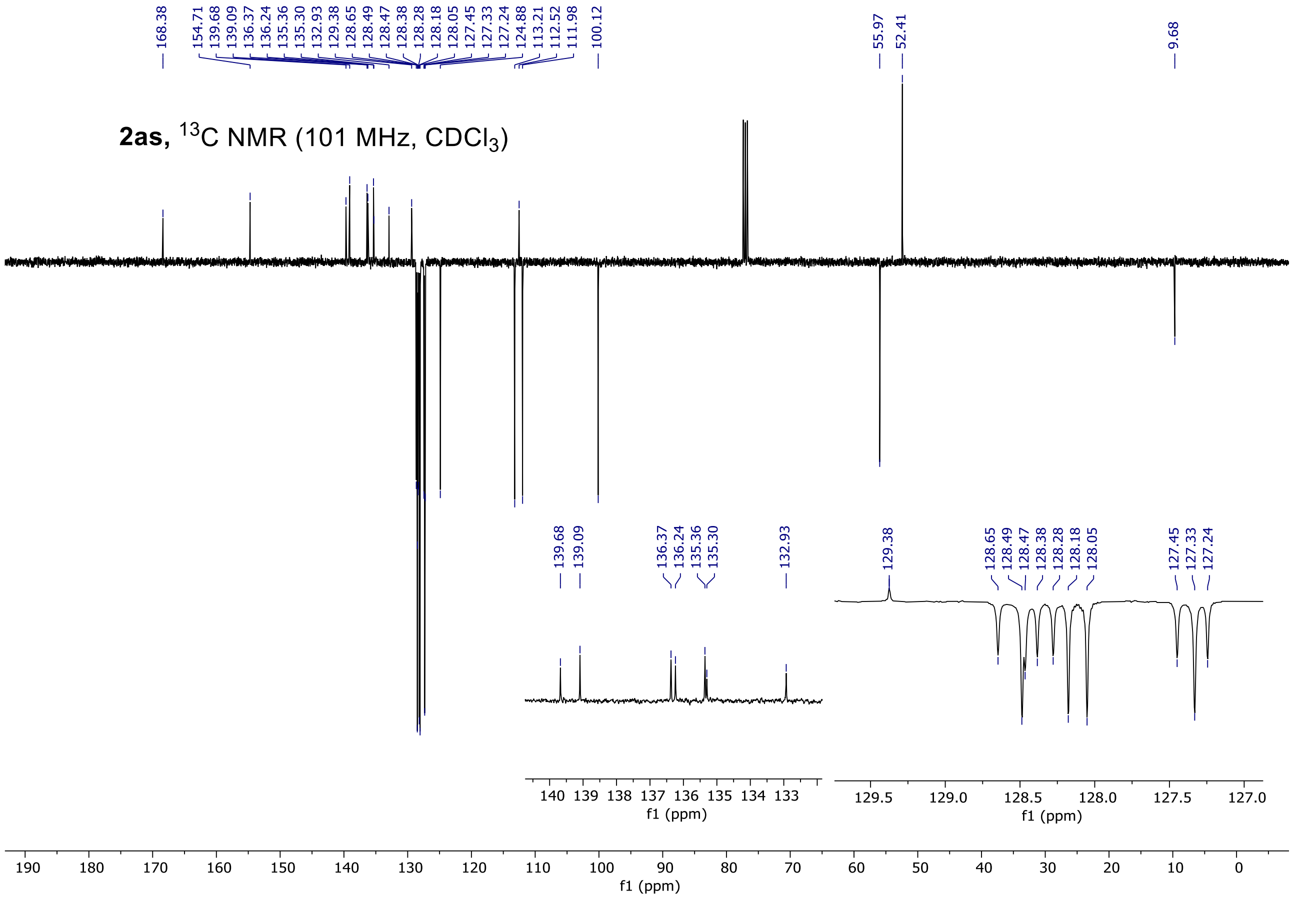

2as, ^{13}C NMR (101 MHz, $CDCl_3$)

7.52, 7.49, 7.46, 7.44, 7.41, 7.40, 7.37, 7.34, 7.31, 7.28, 7.28, 7.26, 7.25, 7.23, 7.20, 7.05, 7.03, 7.00, 6.78, 6.75, 6.72, 6.65, 6.63, 6.60, 6.44, 5.77, 5.72, 4.17, 4.13, 1.90

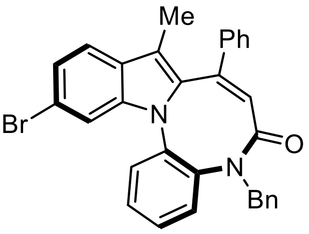

2at, ^{1}H NMR (300 MHz, $CDCl_3$)

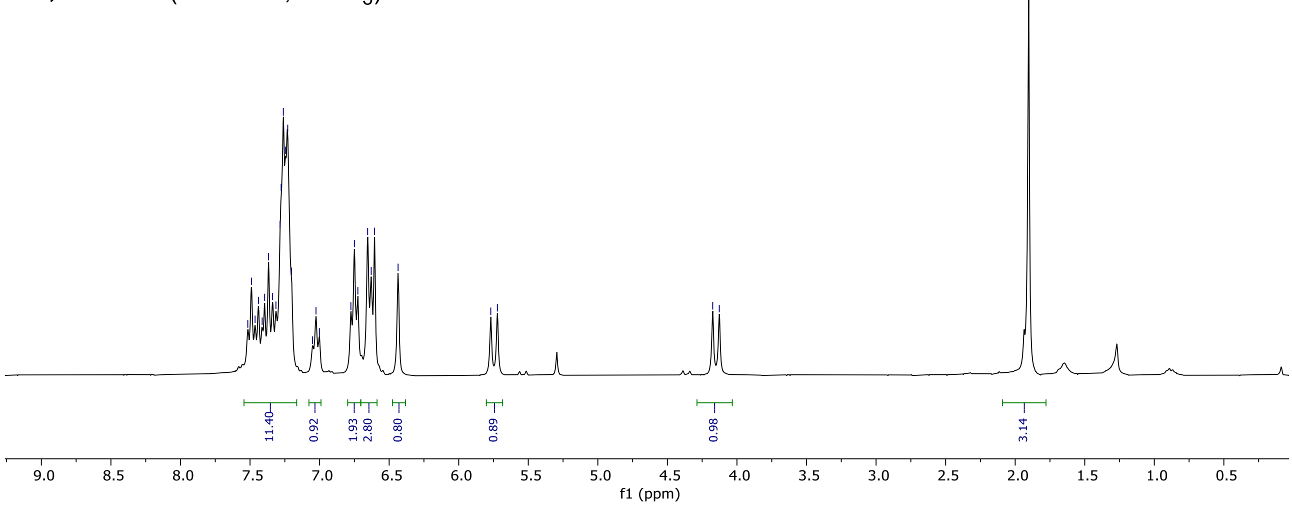

167.90, 139.57, 138.59, 137.92, 135.87, 135.46, 134.97, 134.89, 128.96, 128.79, 128.56, 128.22, 128.12, 128.01, 127.73, 127.63, 127.53, 127.16, 125.29, 123.35, 119.89, 116.55, 113.70, 112.52, 52.27, 9.43

2at, ^{13}C NMR (75 MHz, $CDCl_3$)

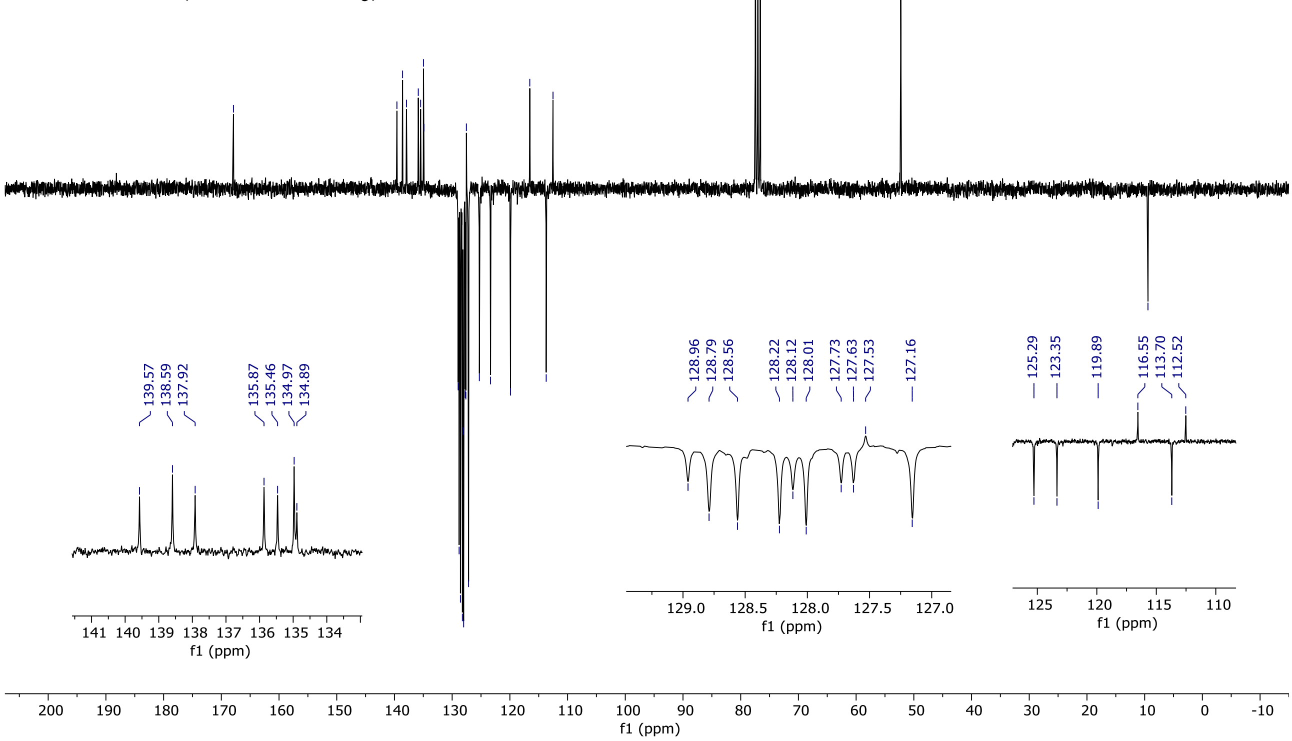

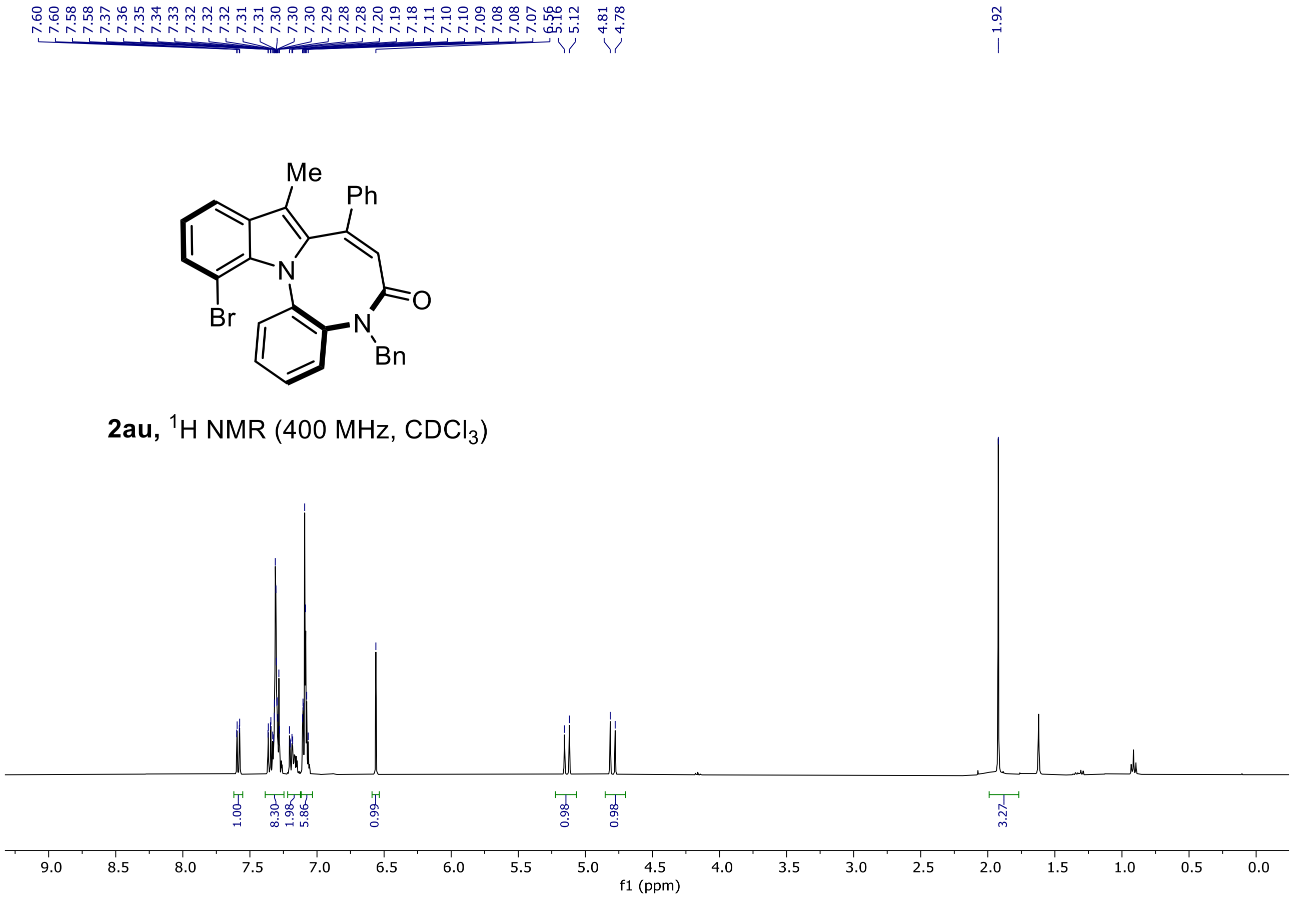

2au, ^{1}H NMR (400 MHz, $CDCl_3$)

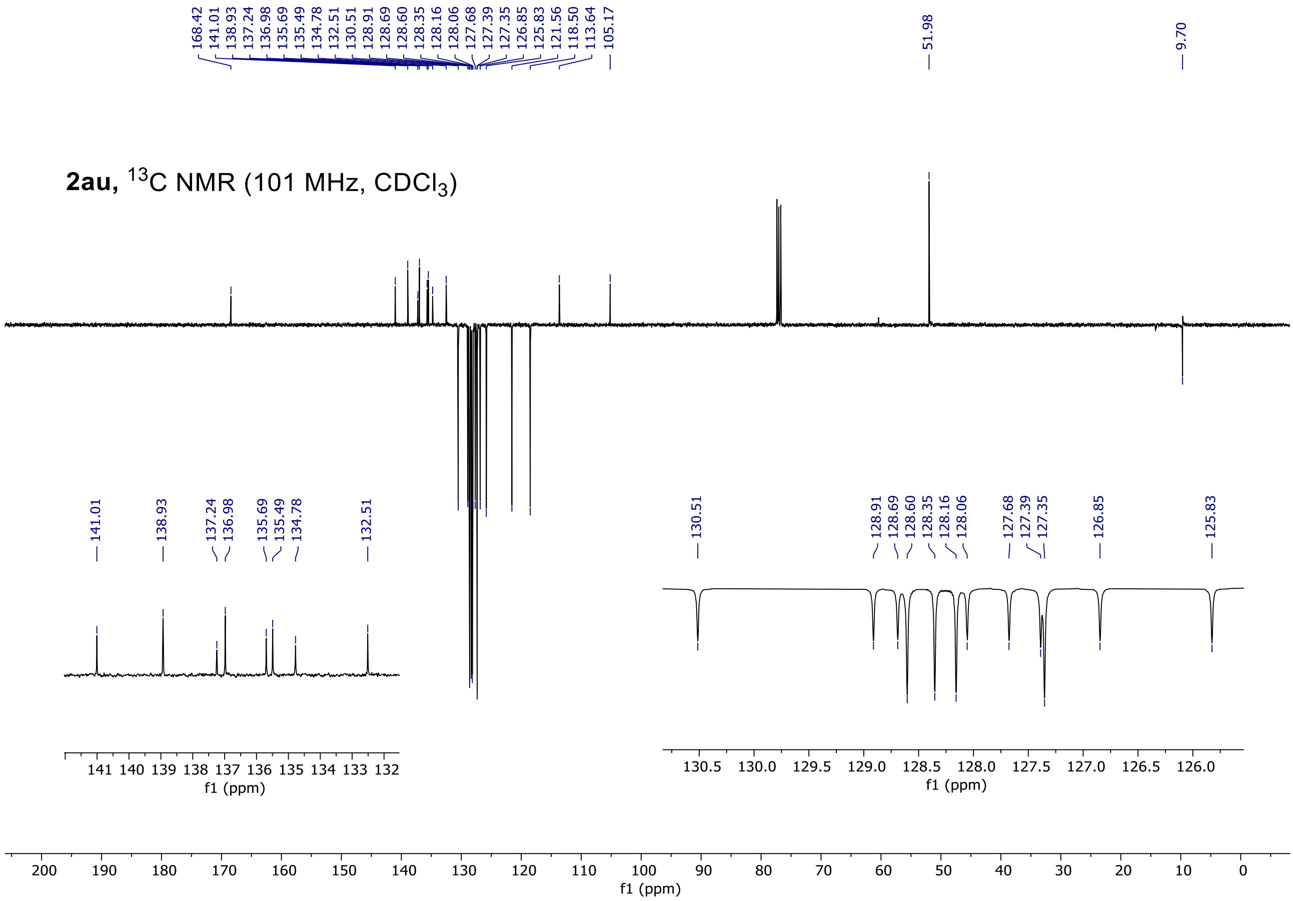

2au, ^{13}C NMR (101 MHz, $CDCl_3$)

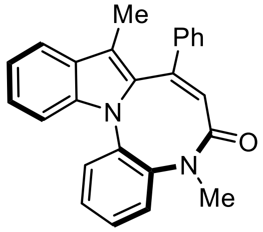

2av, ^{1}H NMR (300 MHz, DMSO)

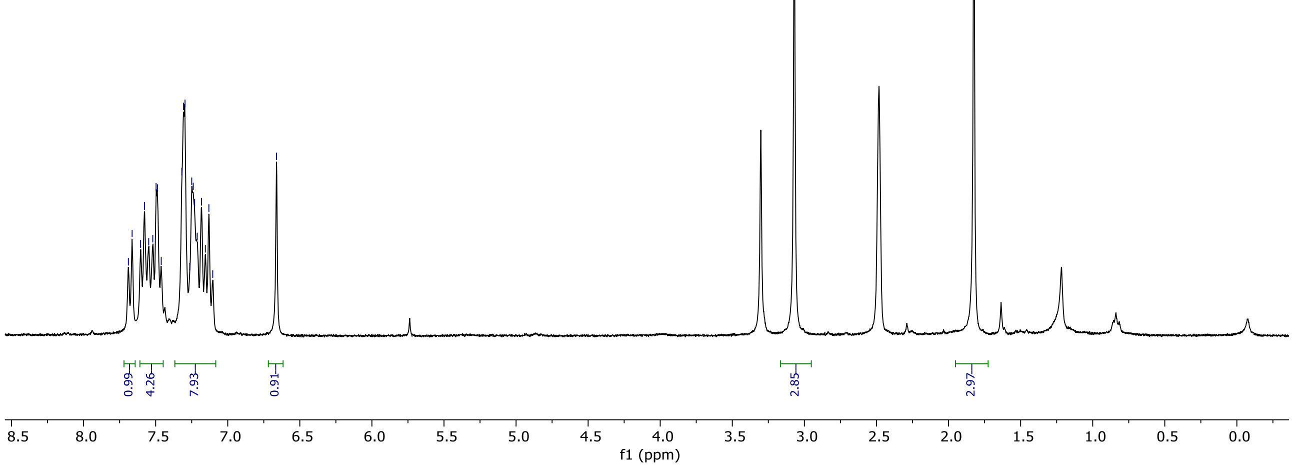

2av, ^{13}C NMR (75 MHz, DMSO)

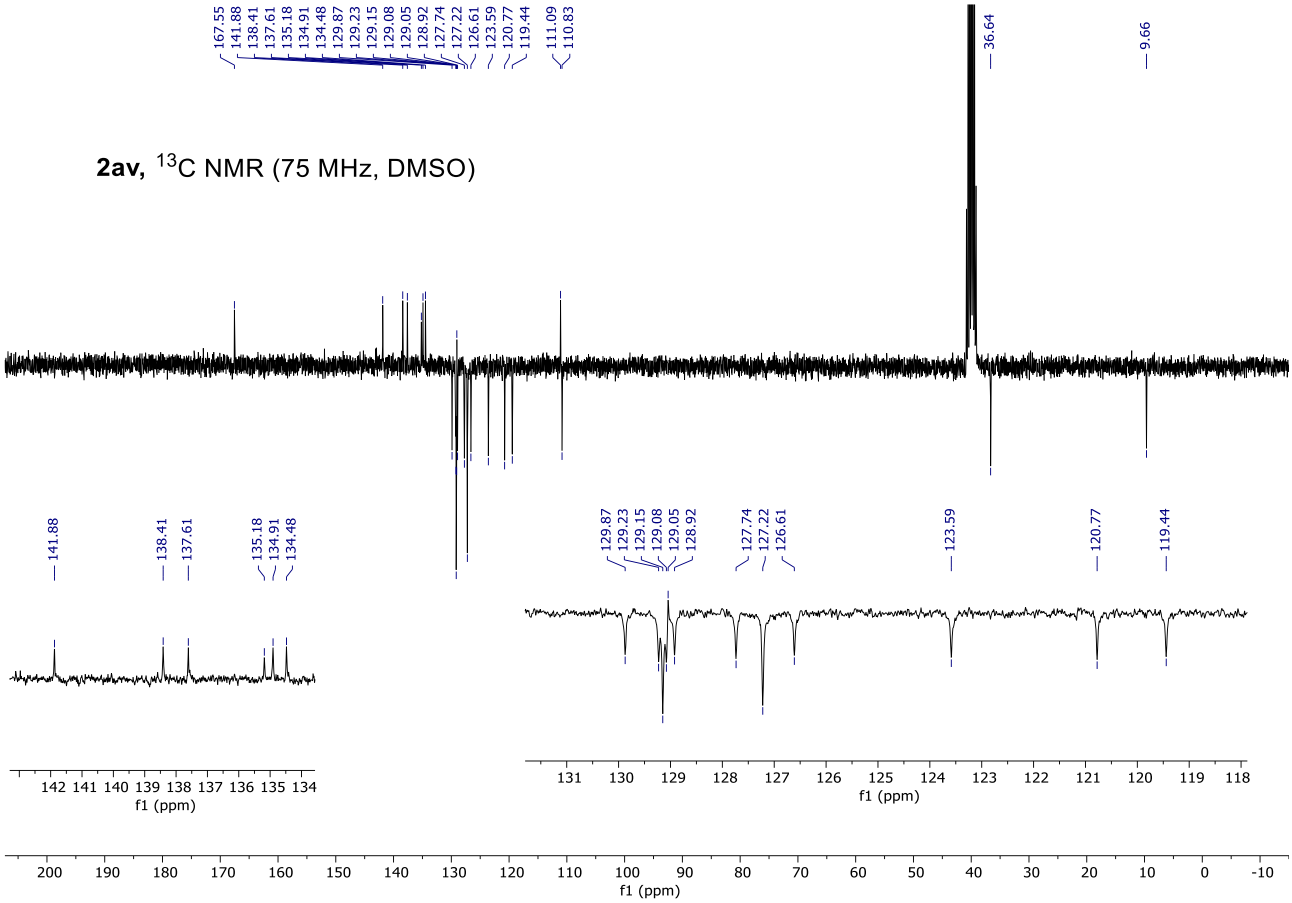

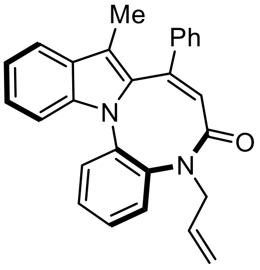

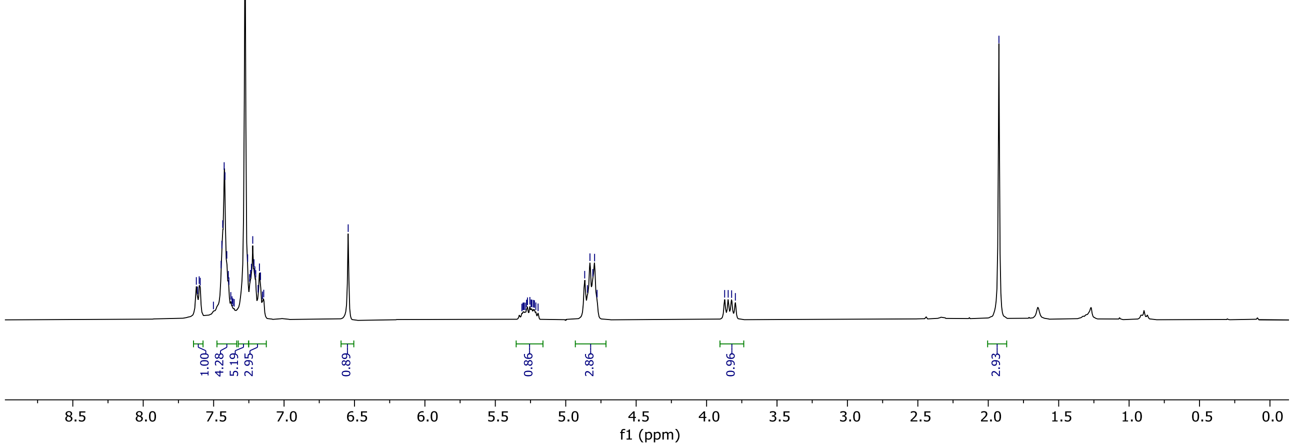

2aw, ^{1}H NMR (300 MHz, $CDCl_3$)

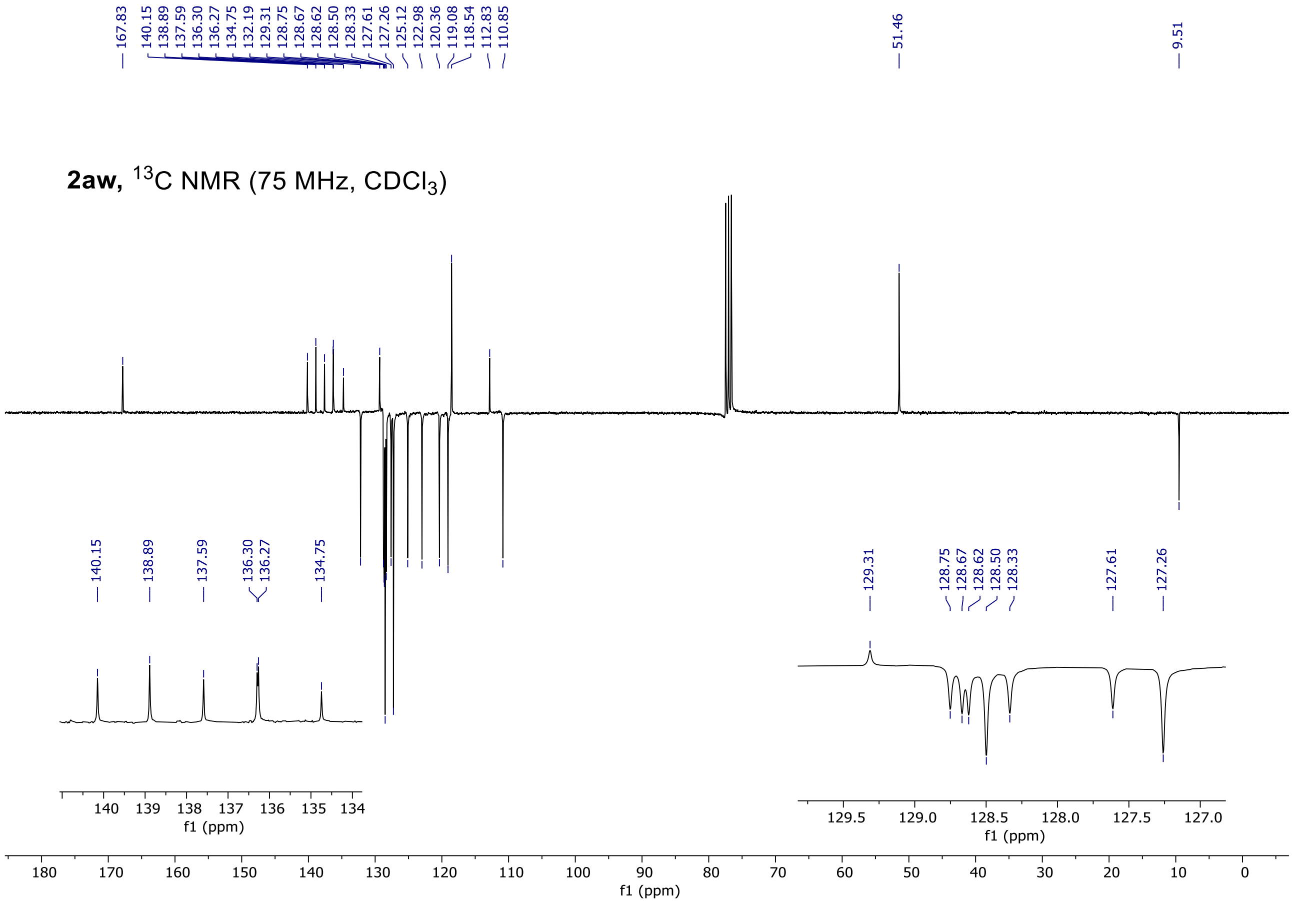

2aw, ^{13}C NMR (75 MHz, $CDCl_3$)

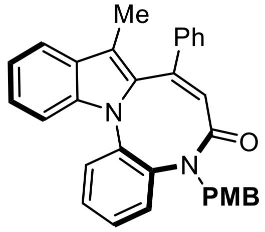

2ax, ^{1}H NMR (300 MHz, $CDCl_3$)

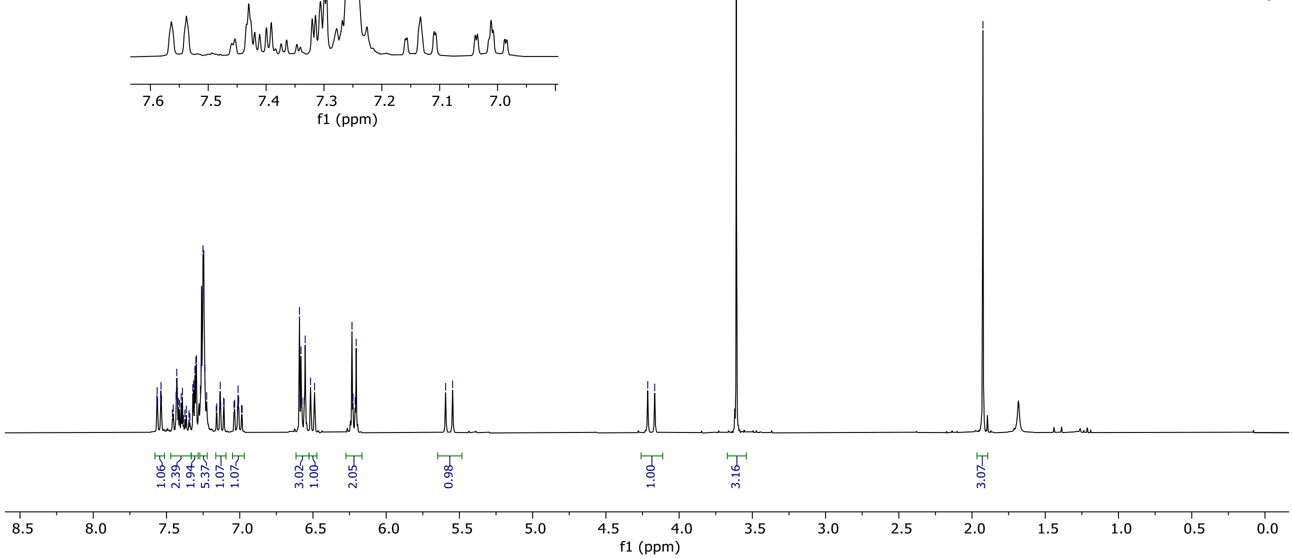

2ax, ^{13}C NMR (75 MHz, $CDCl_3$)

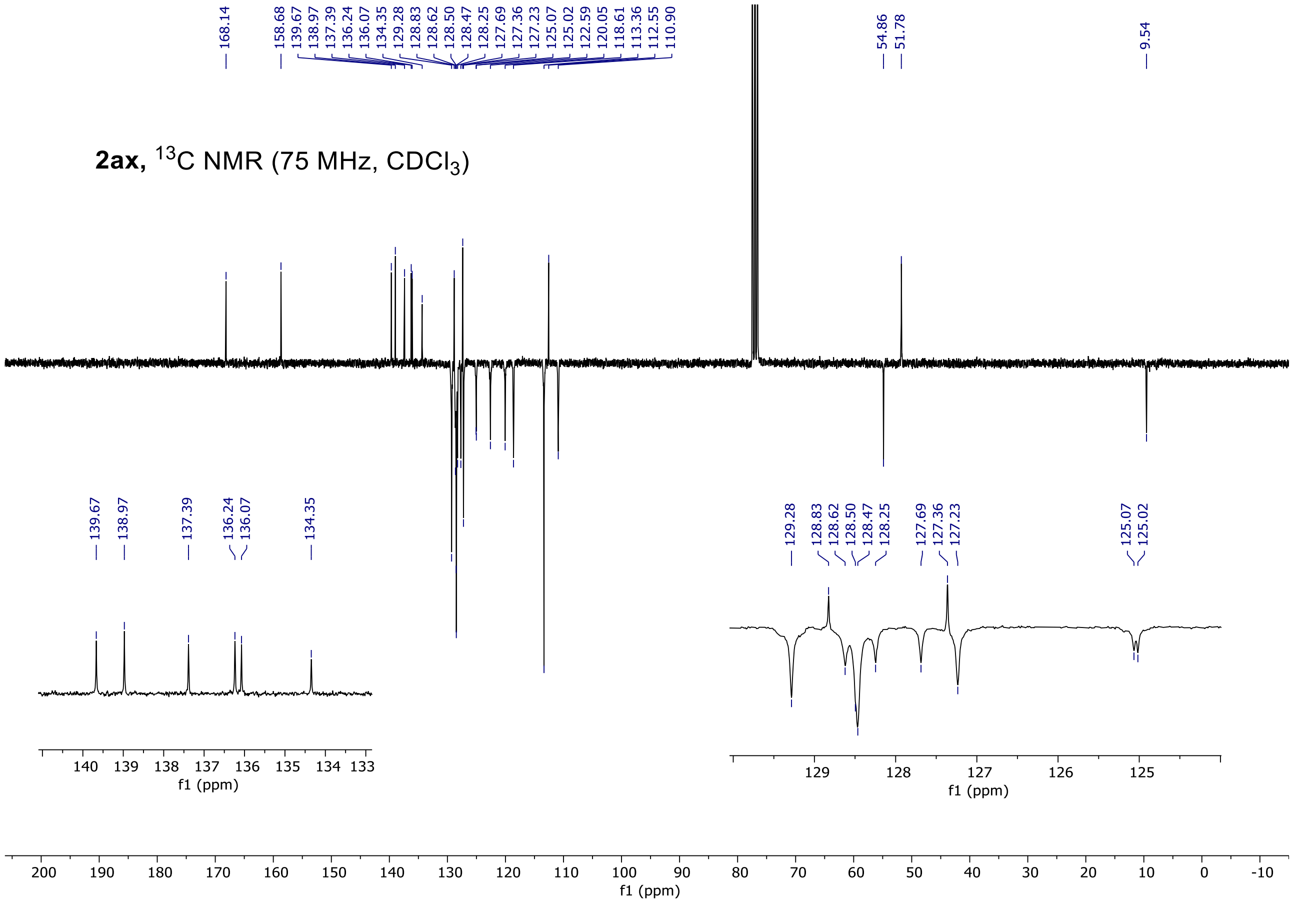

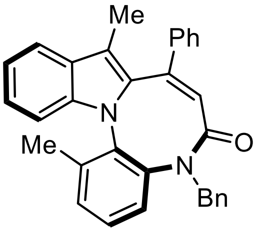

2ay, ^{1}H NMR (400 MHz, $CDCl_3$)

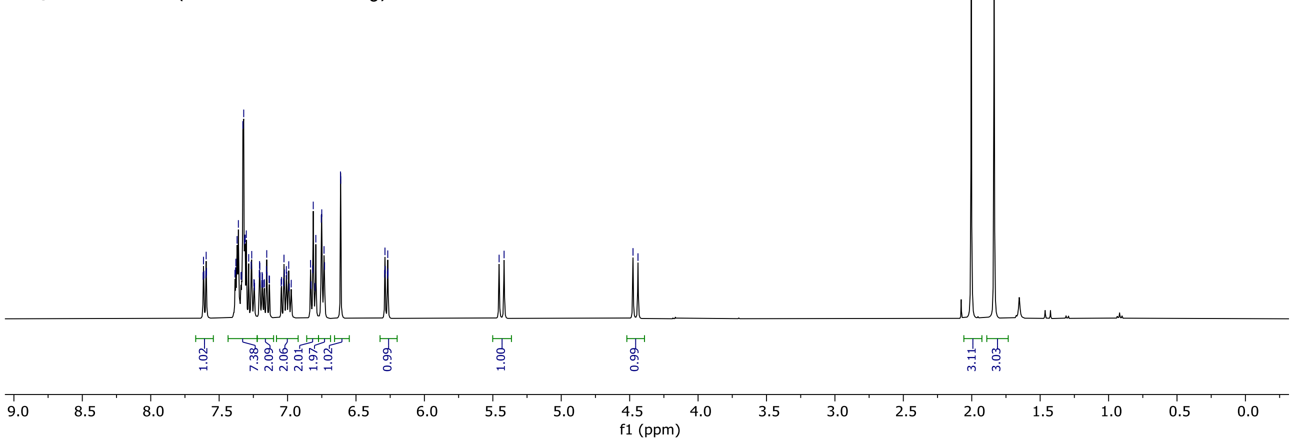

2ay, ^{13}C NMR (101 MHz, $CDCl_3$)

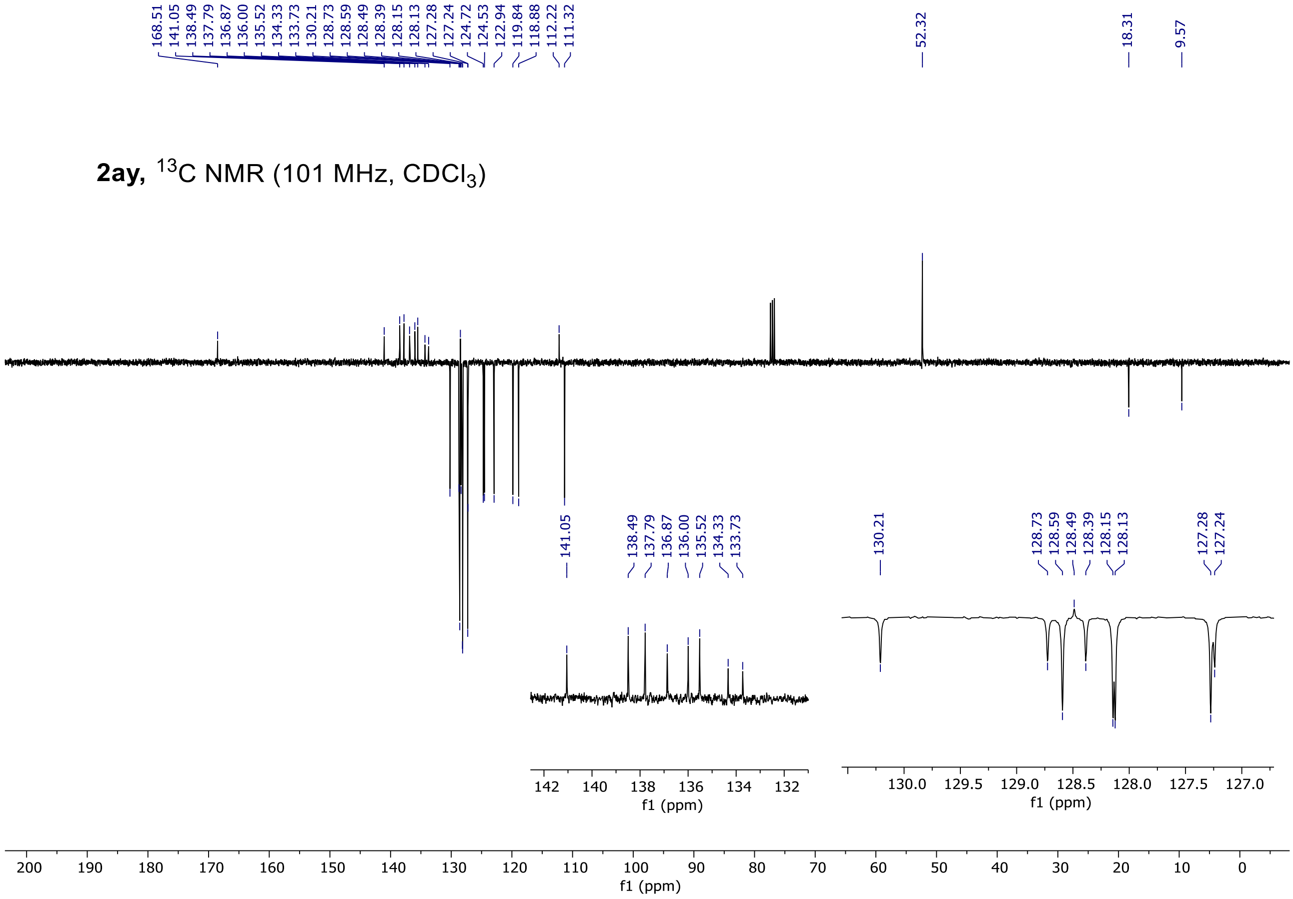

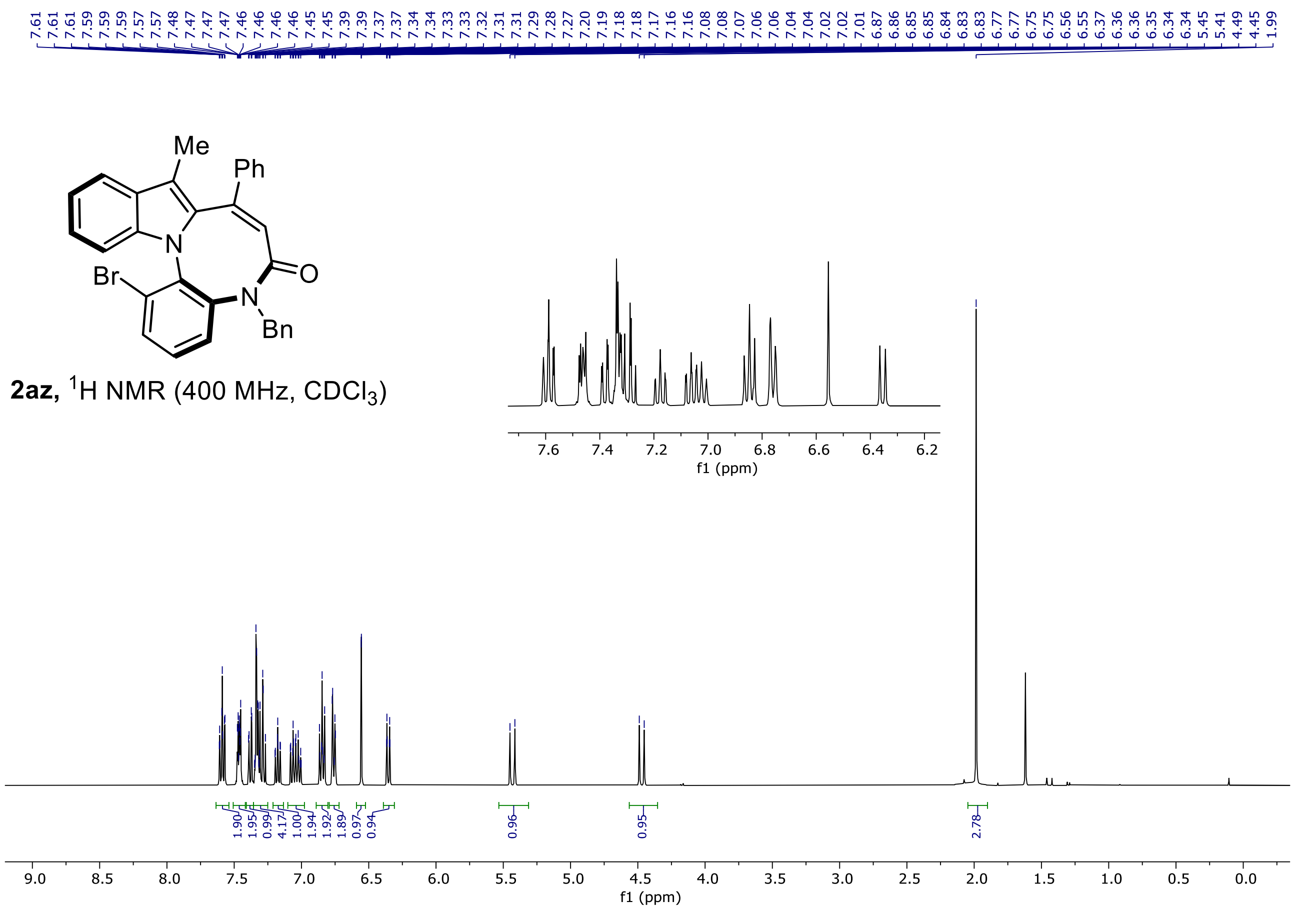

2az, ^{1}H NMR (400 MHz, $CDCl_3$)

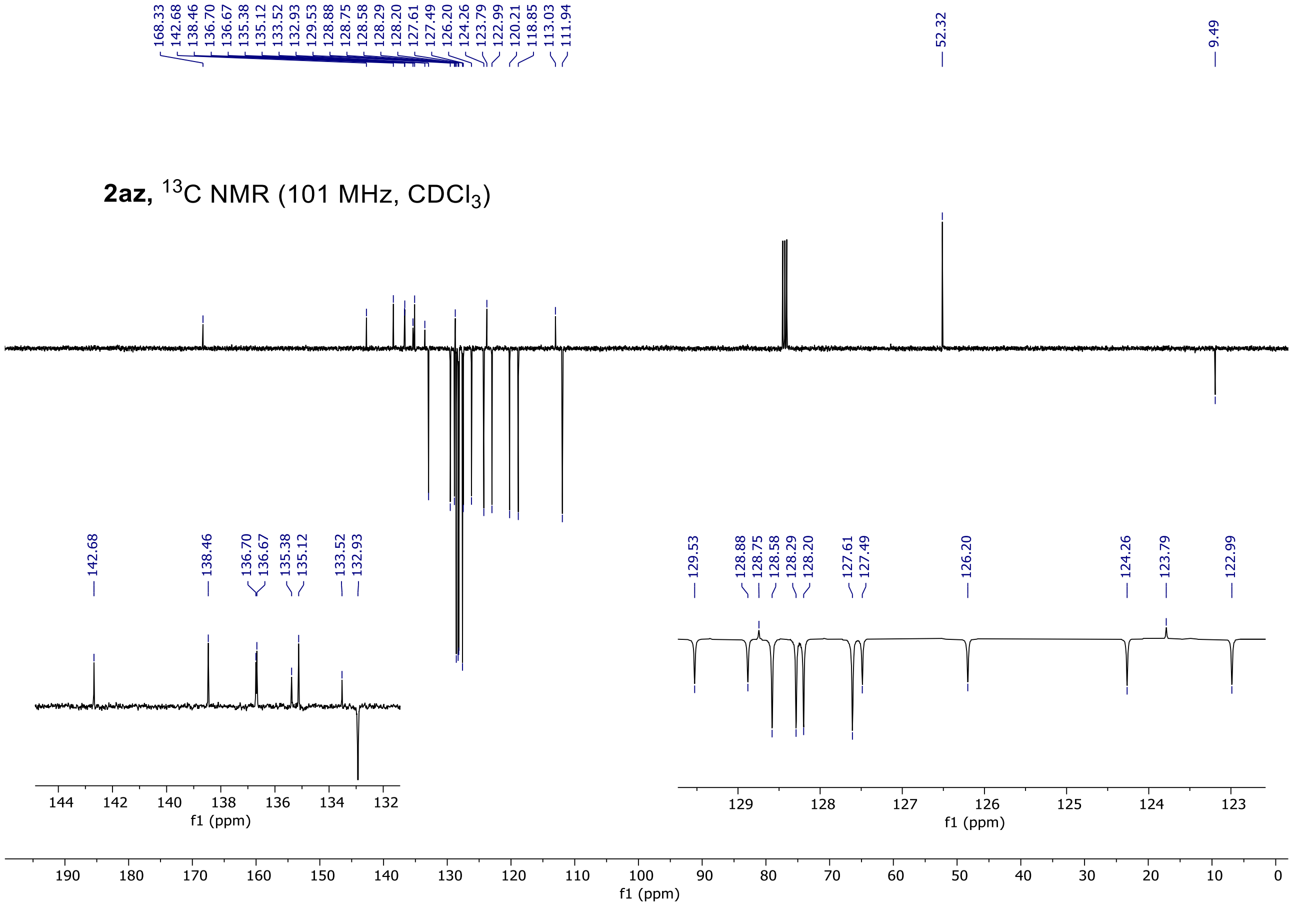

2az, ^{13}C NMR (101 MHz, $CDCl_3$)

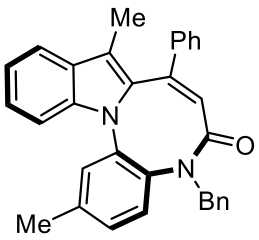

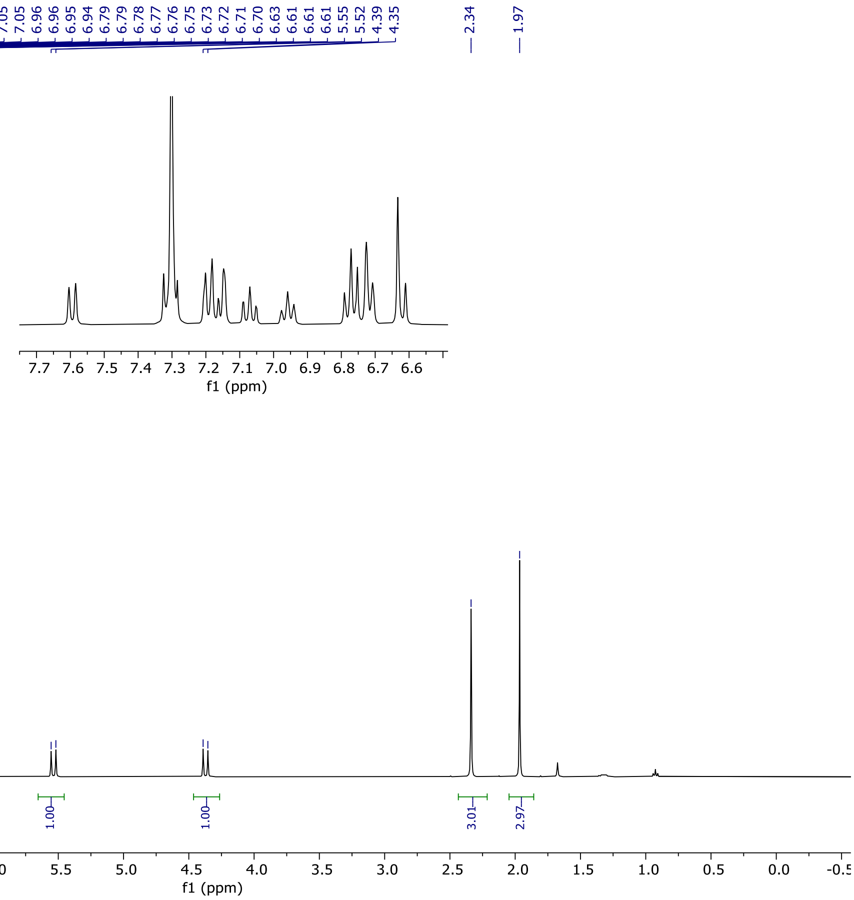

2ba, ^{1}H NMR (400 MHz, $CDCl_3$)

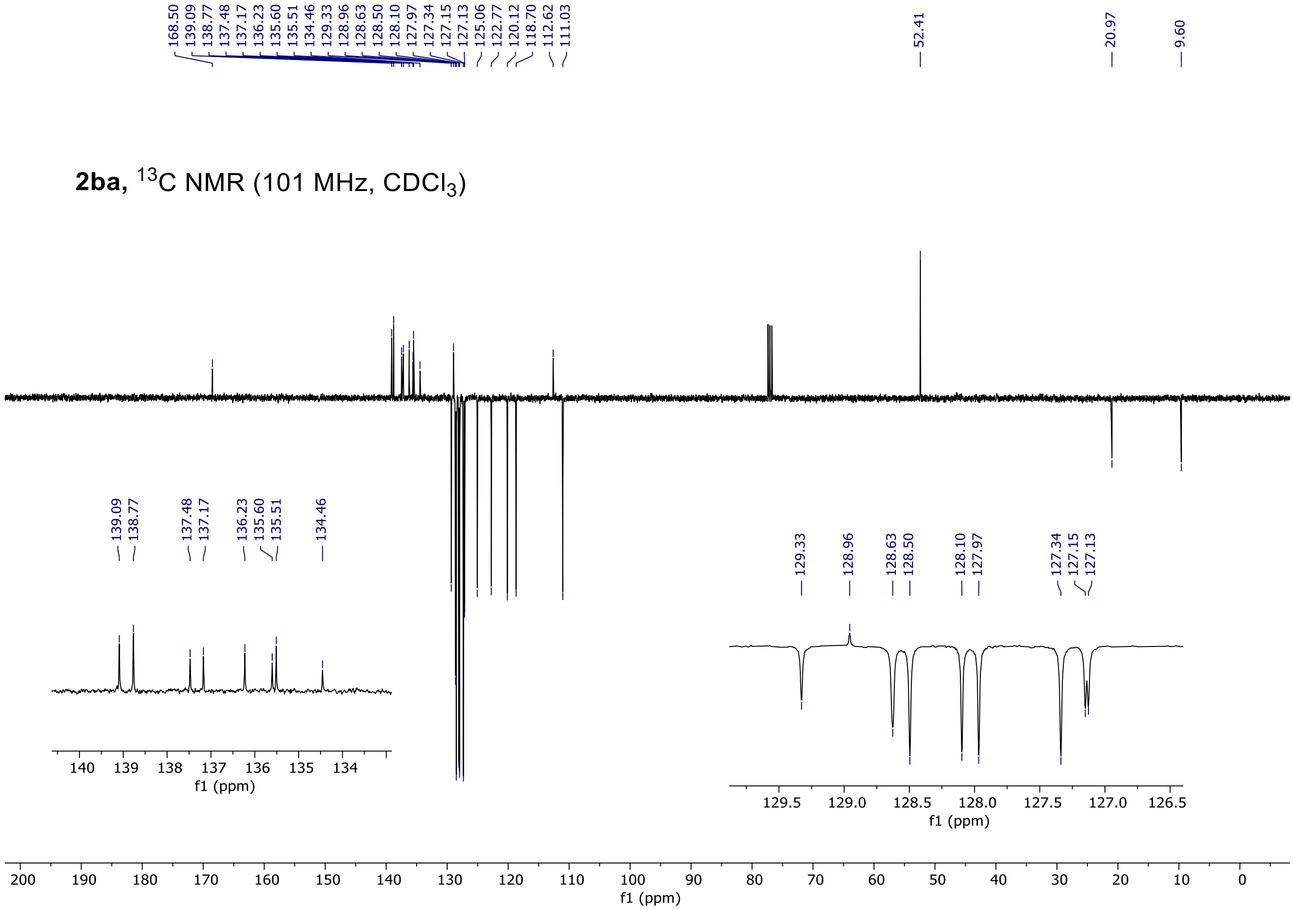

2ba, ^{13}C NMR (101 MHz, $CDCl_3$)

2ba, HSQC ($CDCl_3$)

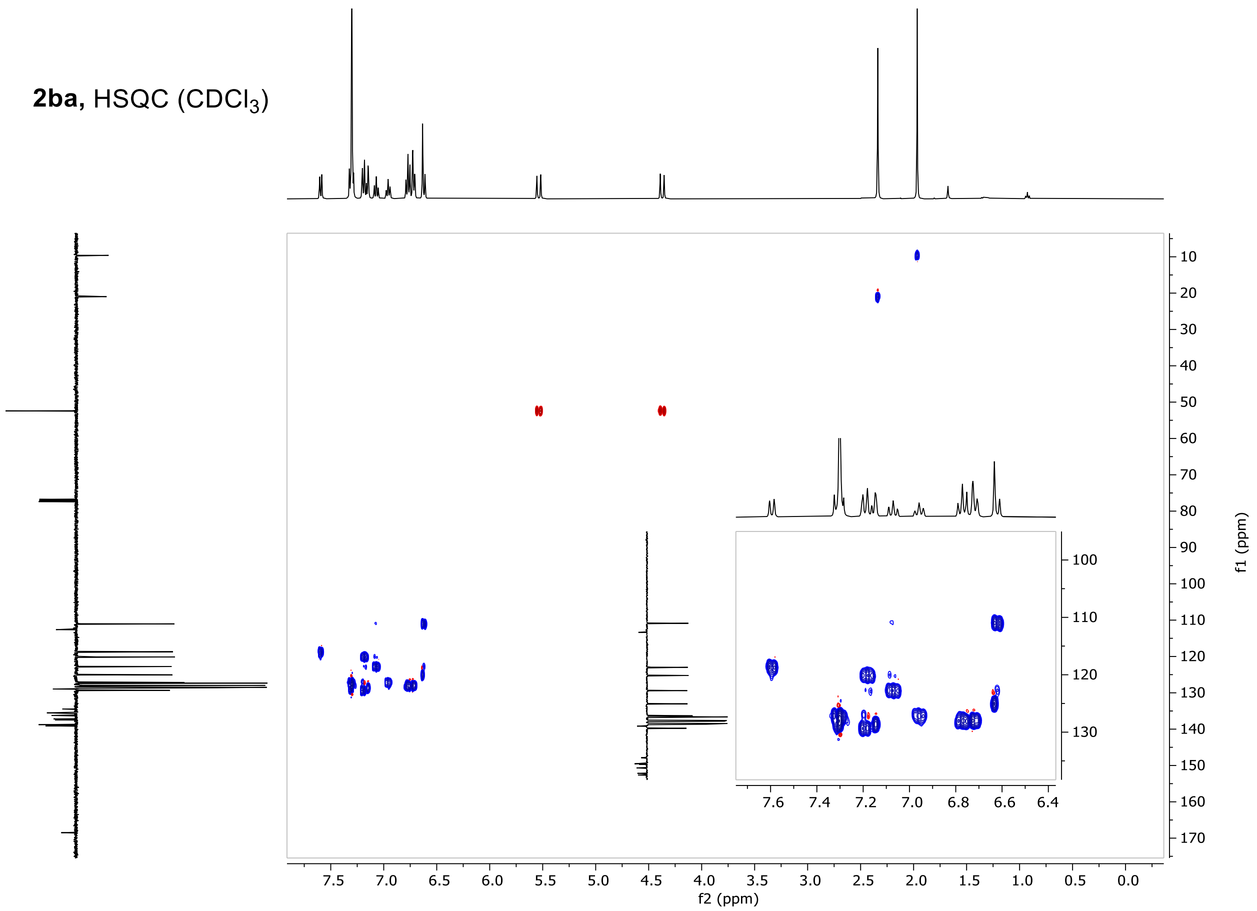

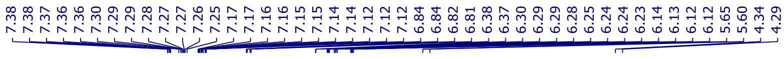

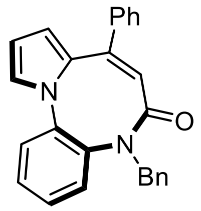

4, ^{1}H NMR (300 MHz, $CDCl_3$)

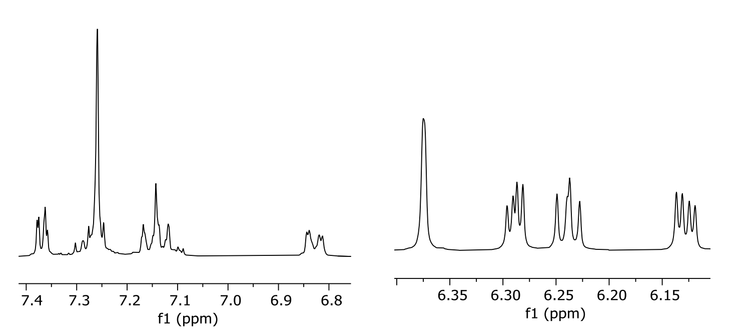

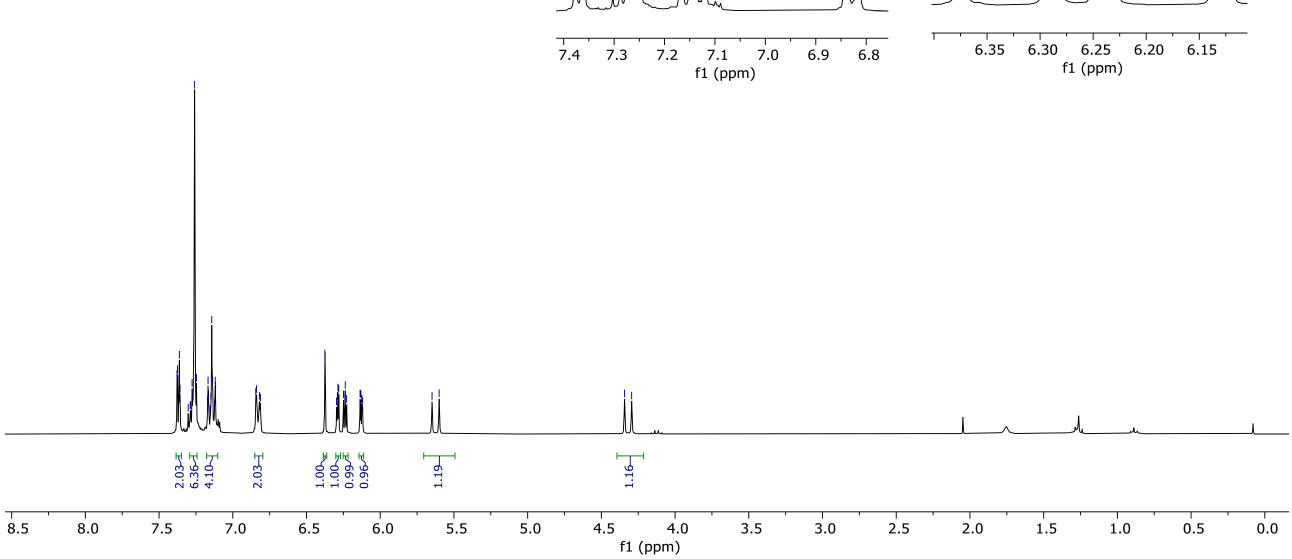

4, ^{13}C NMR (75 MHz, $CDCl_3$)

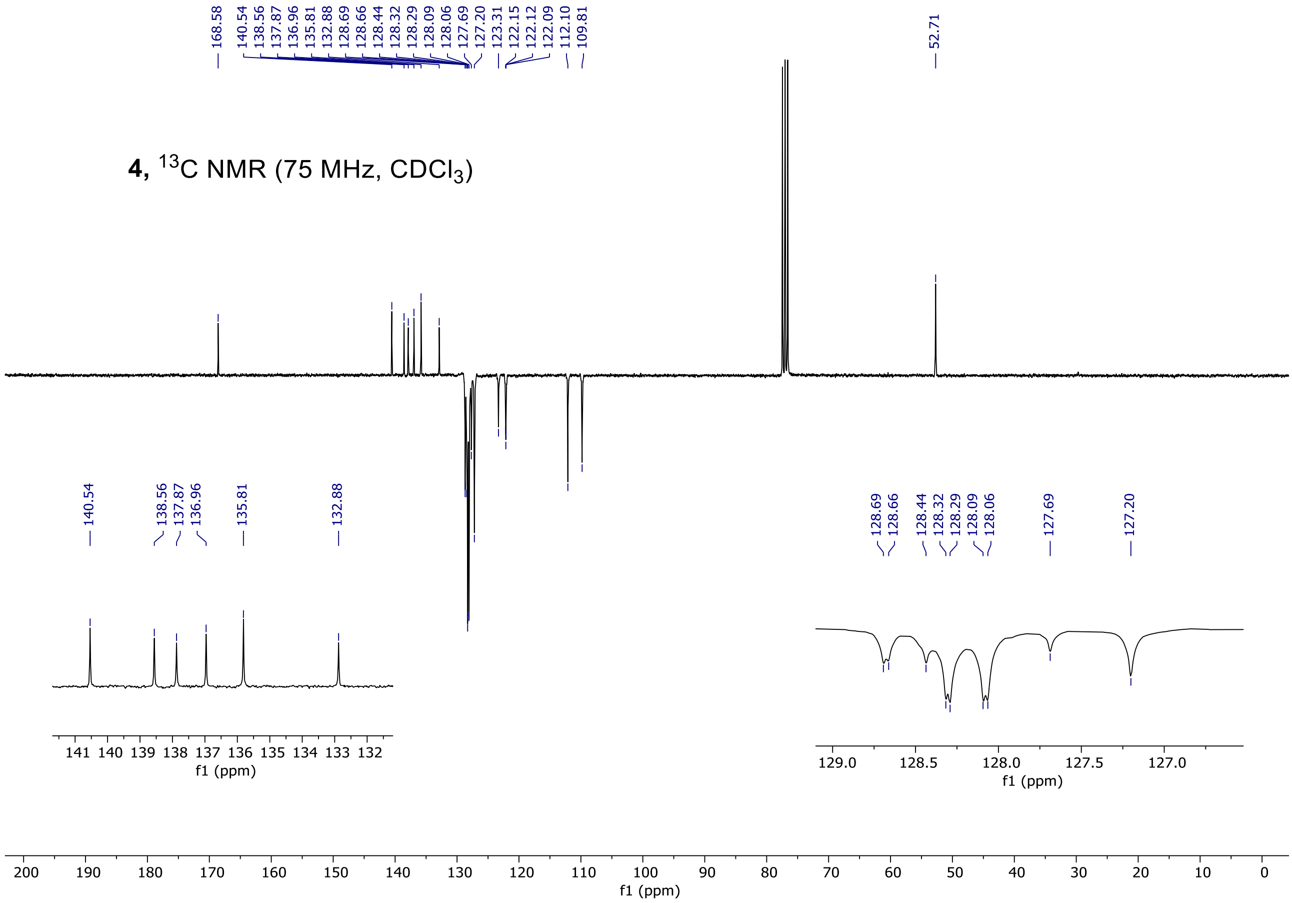

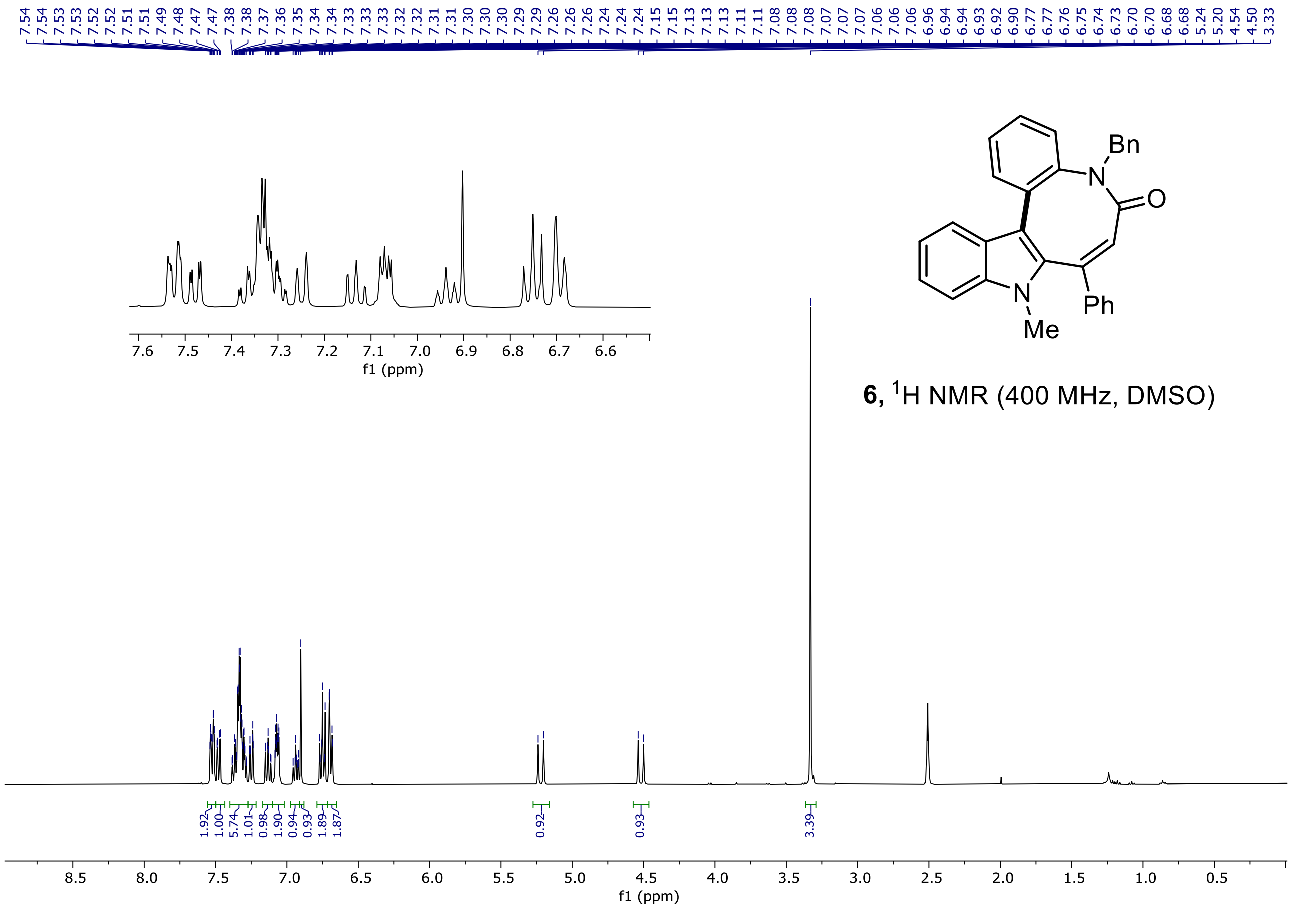

6, ^{1}H NMR (400 MHz, DMSO)

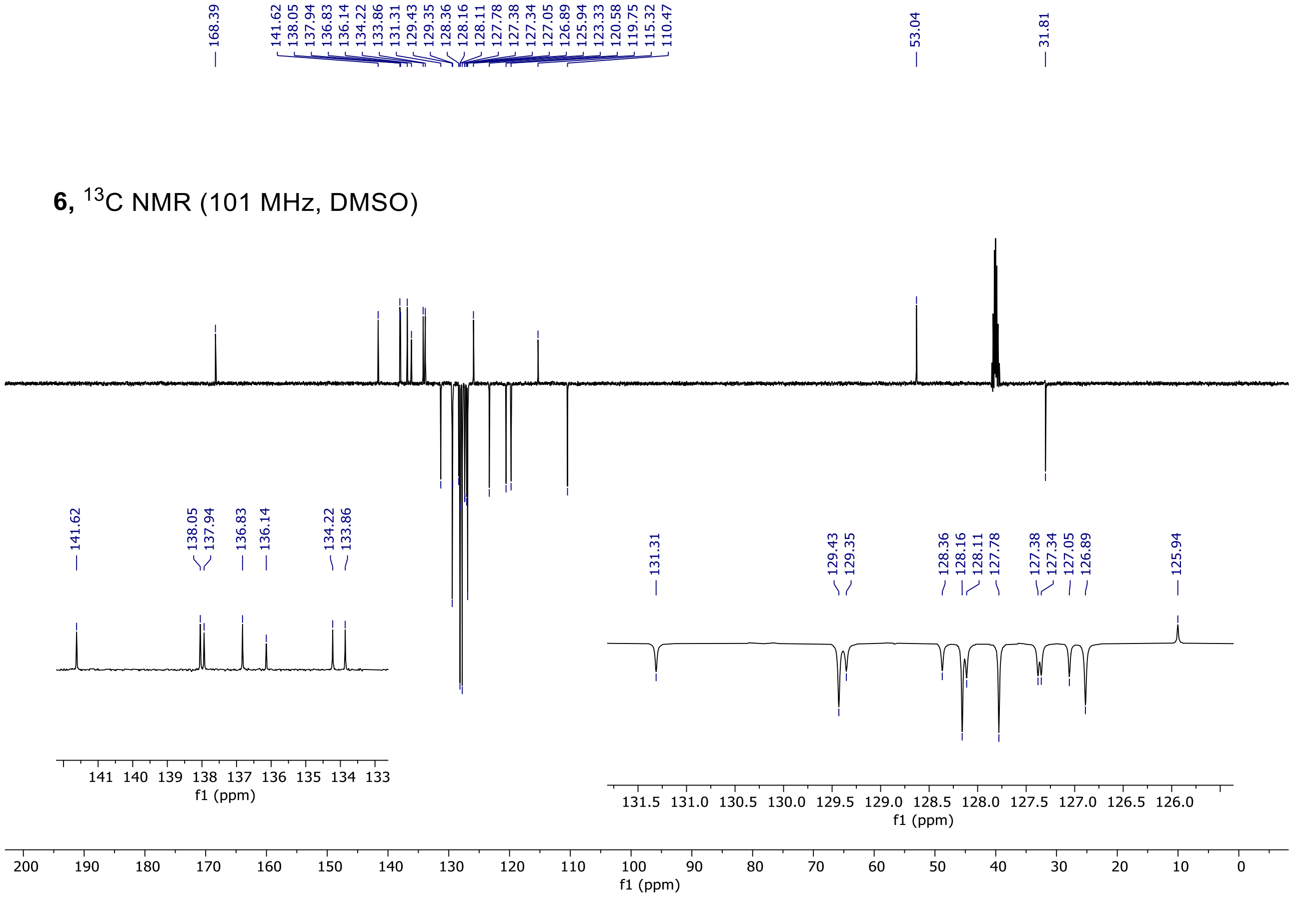

6, ^{13}C NMR (101 MHz, DMSO)

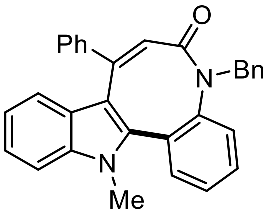

8, ^{1}H NMR (400 MHz, DMSO)

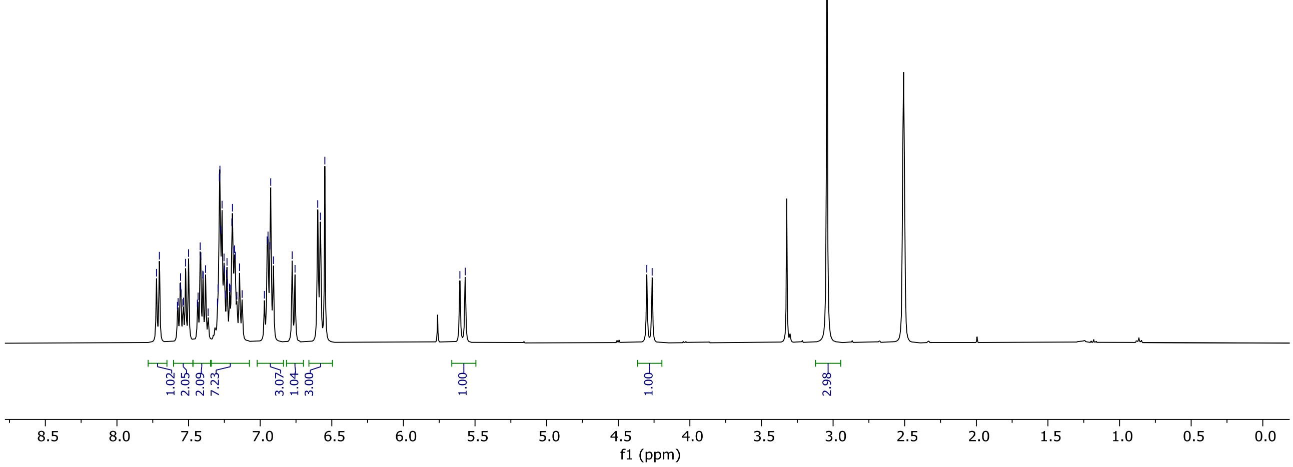

8, ^{13}C NMR (101 MHz, DMSO)

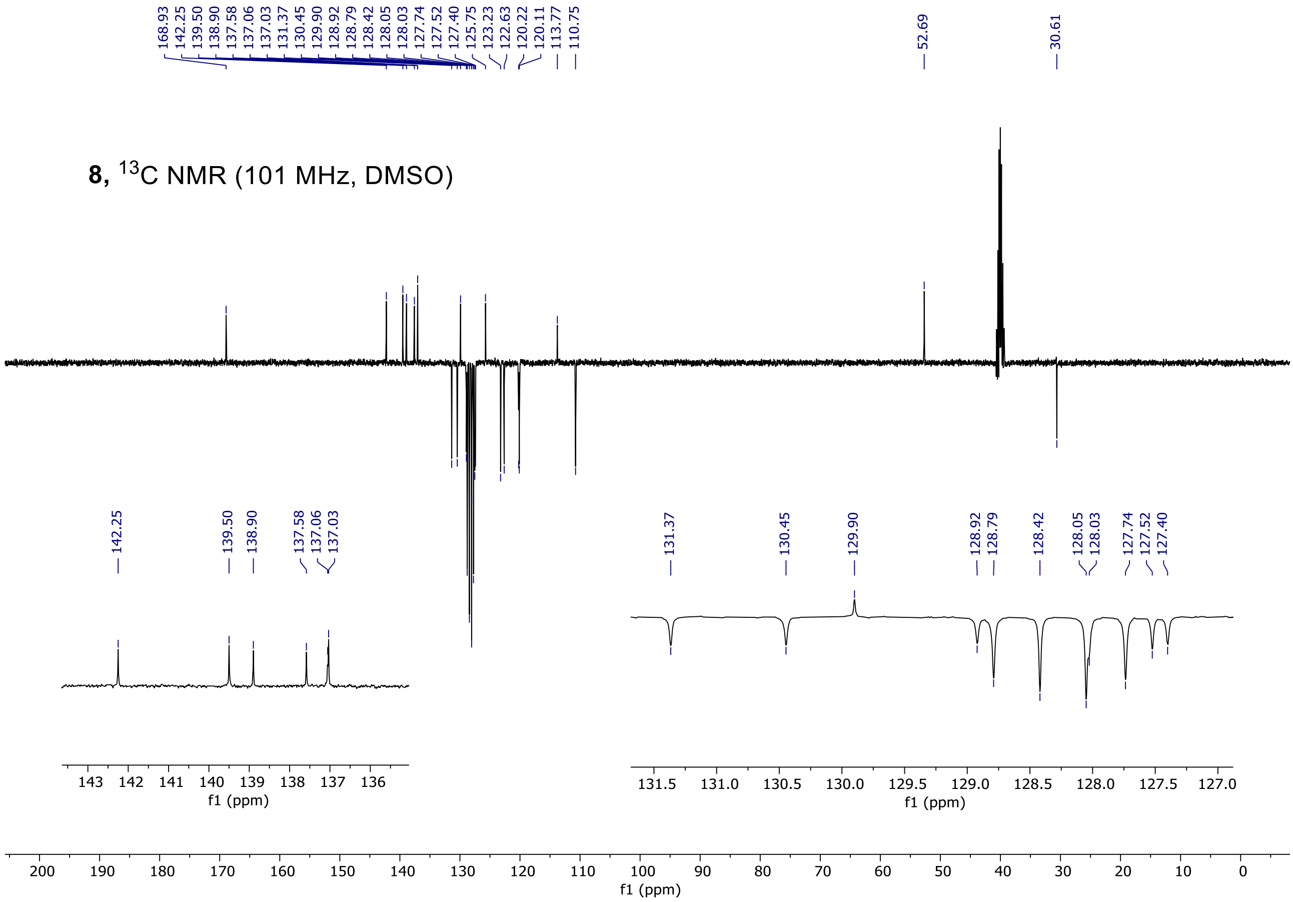

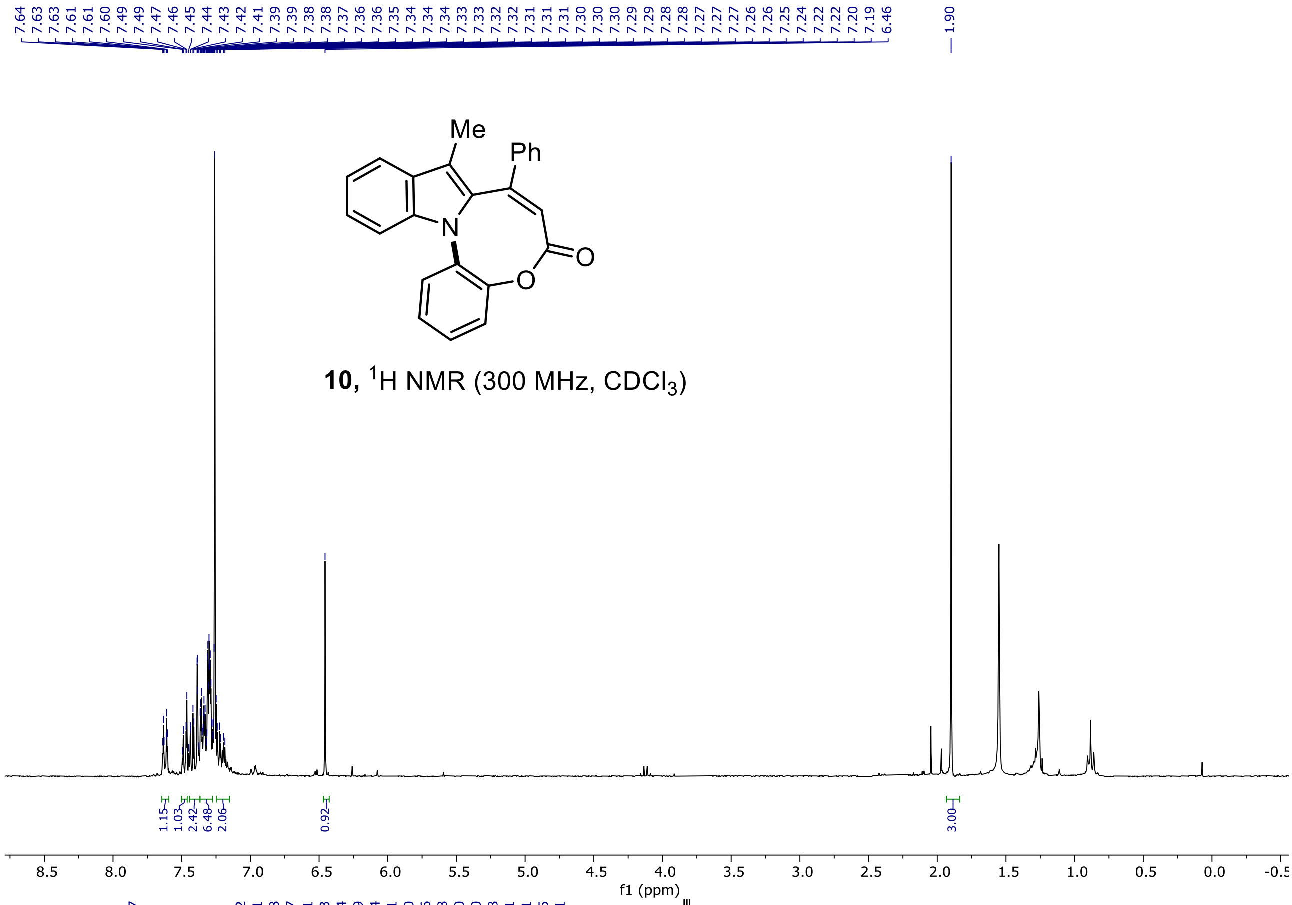
Me
Ph
N
O
O
10, ^{1}H NMR (300 MHz, $CDCl_3$)
f1 (ppm)

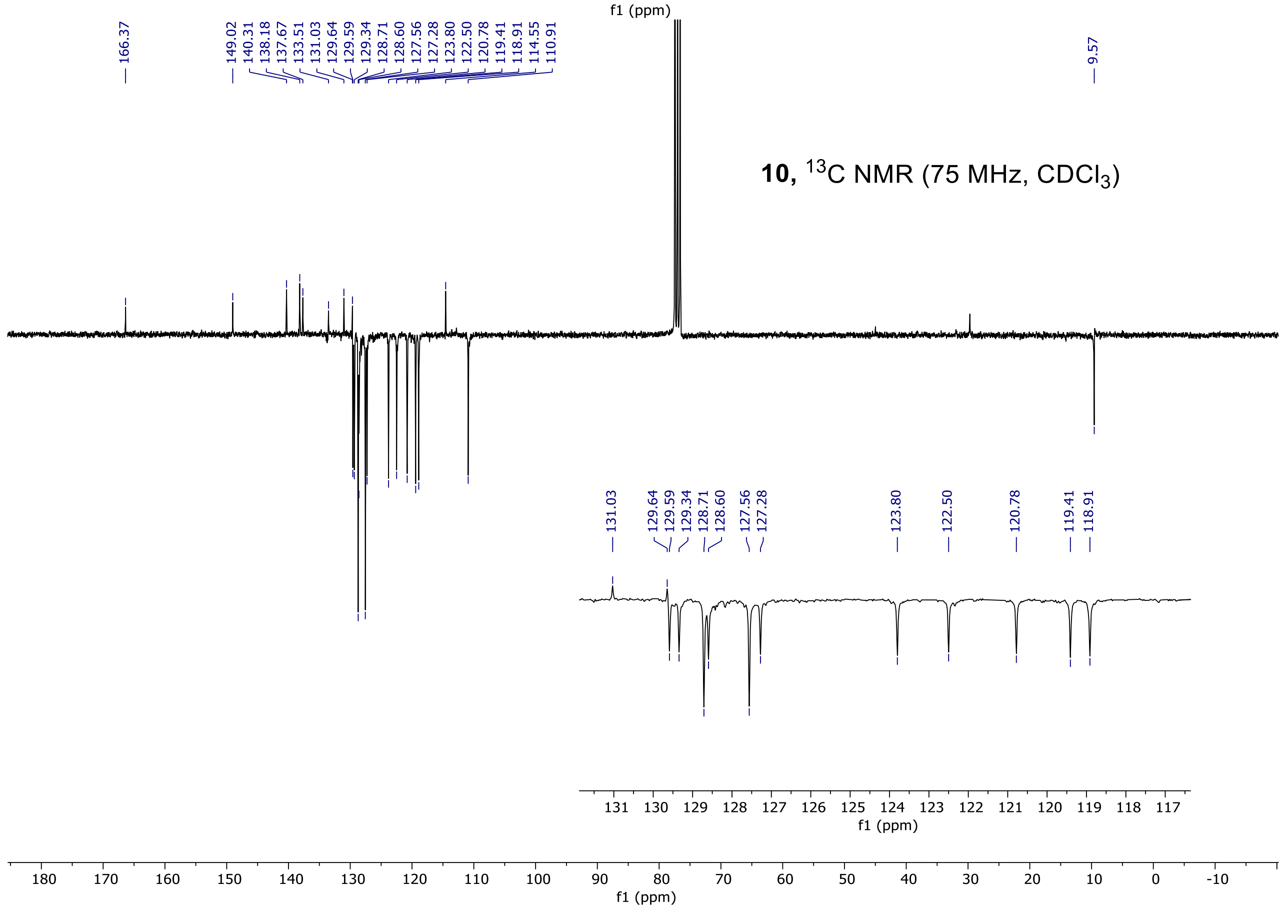
10, ^{13}C NMR (75 MHz, $CDCl_3$)
f1 (ppm)

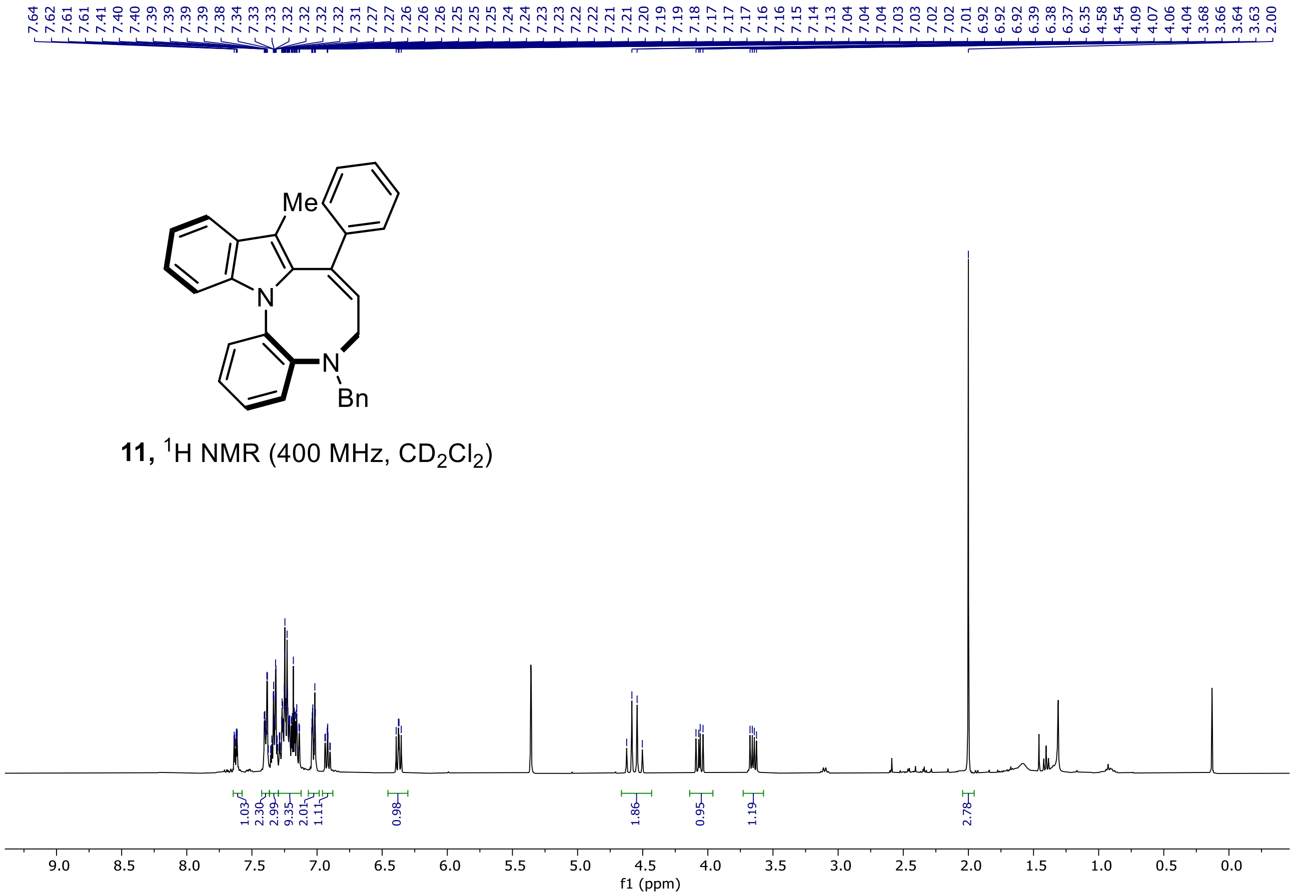

11, ^{1}H NMR (400 MHz, CD_2Cl_2)

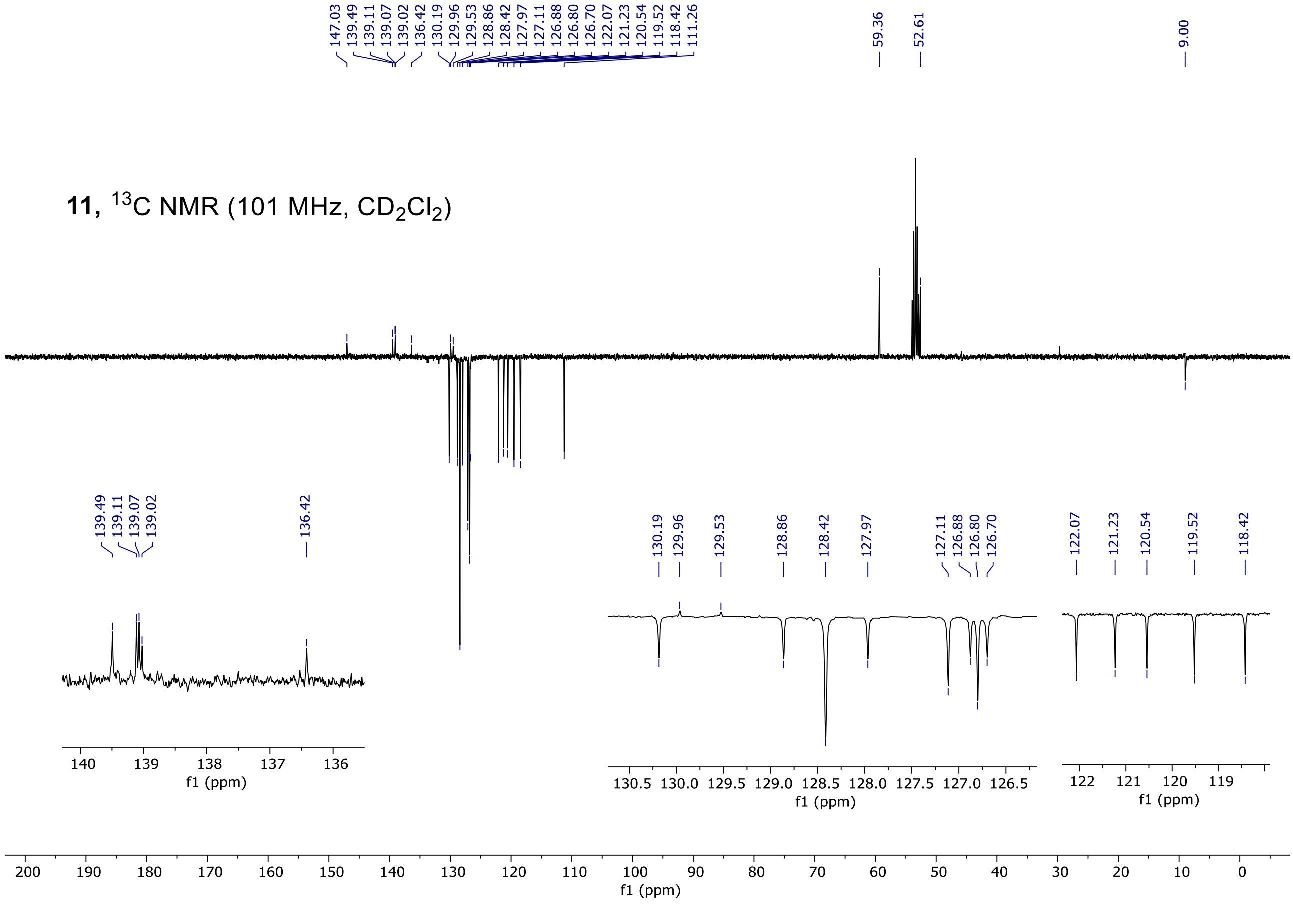

11, ^{13}C NMR (101 MHz, CD_2Cl_2)

11, HSQC (CD_2Cl_2)

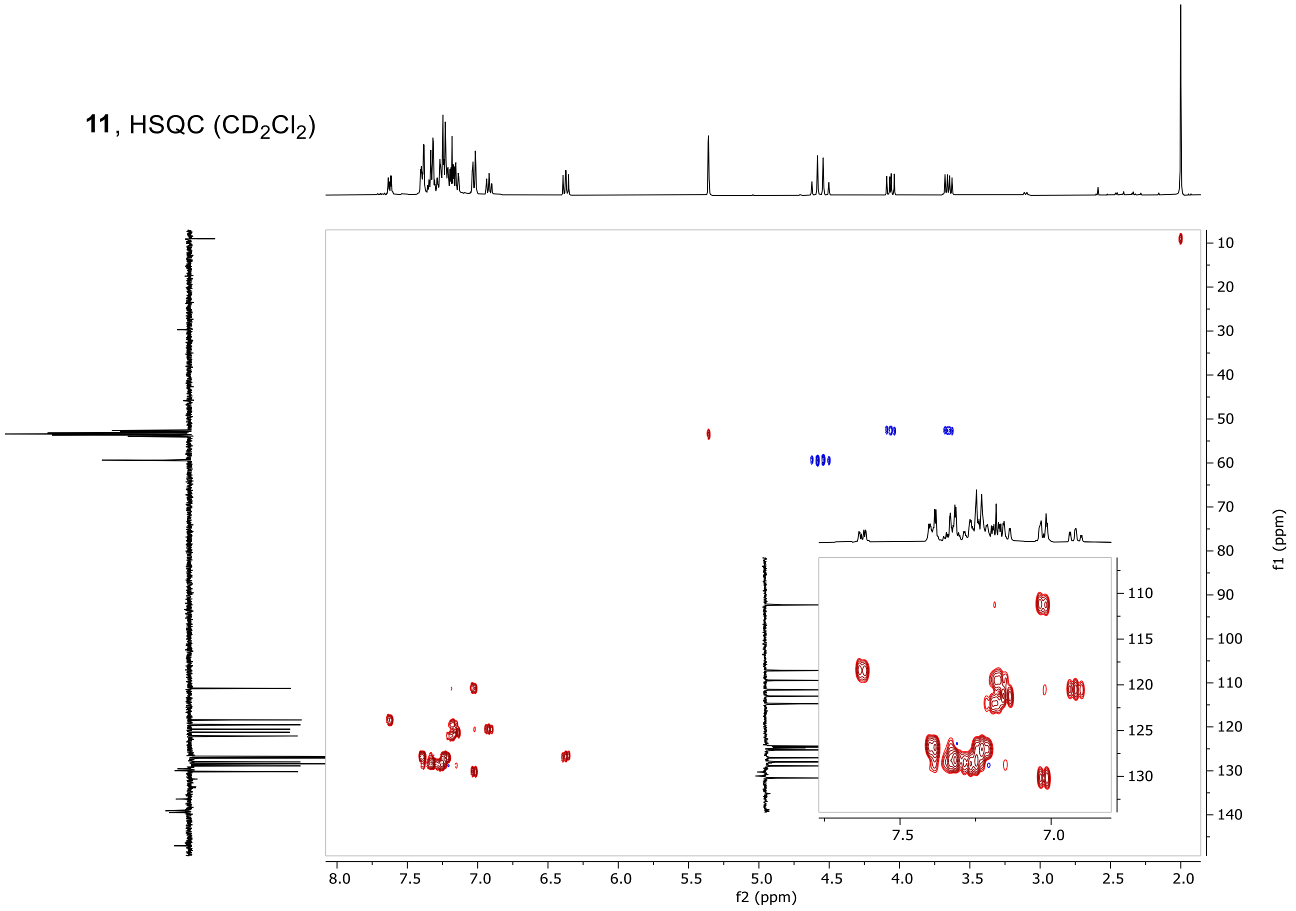

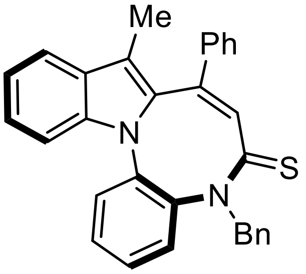

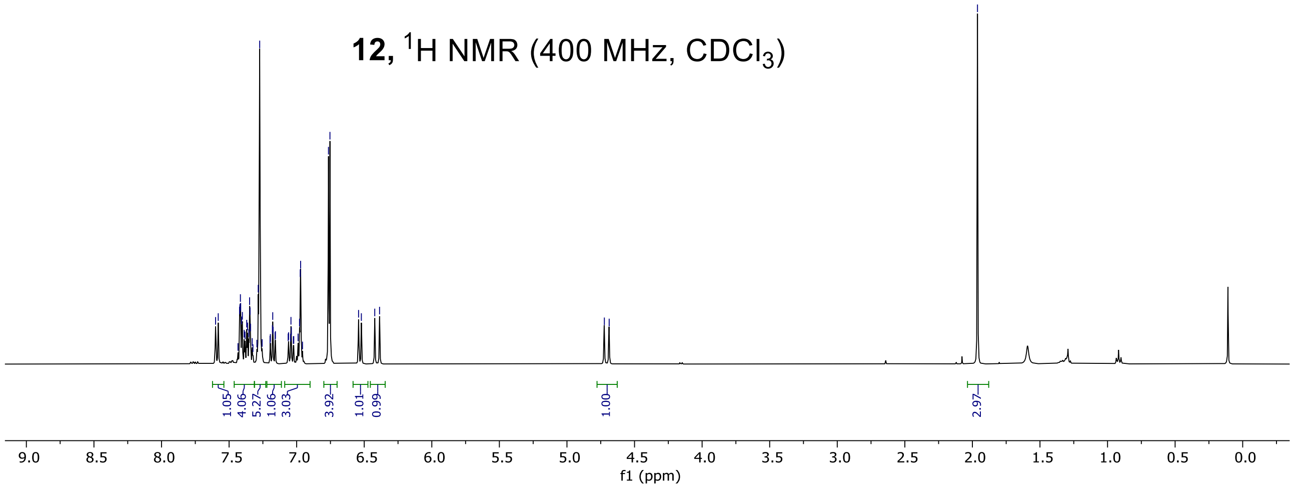

12, ^{1}H NMR (400 MHz, $CDCl_3$)

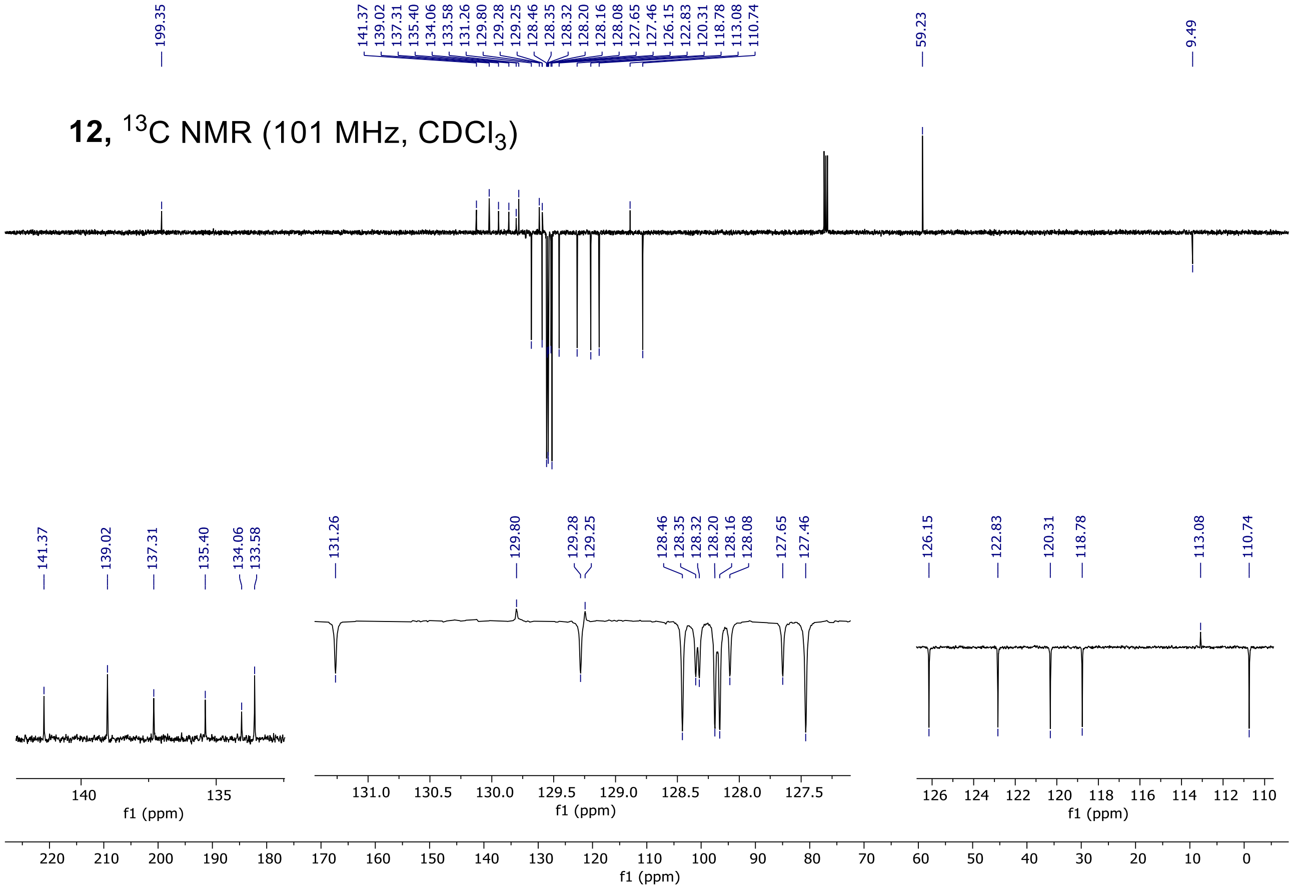

12, ^{13}C NMR (101 MHz, $CDCl_3$)

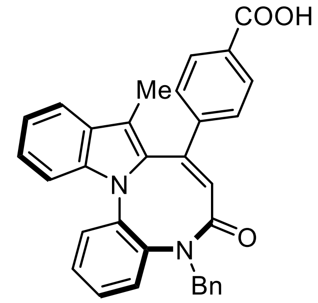

2bb, ^{1}H NMR (400 MHz, $CDCl_3$)

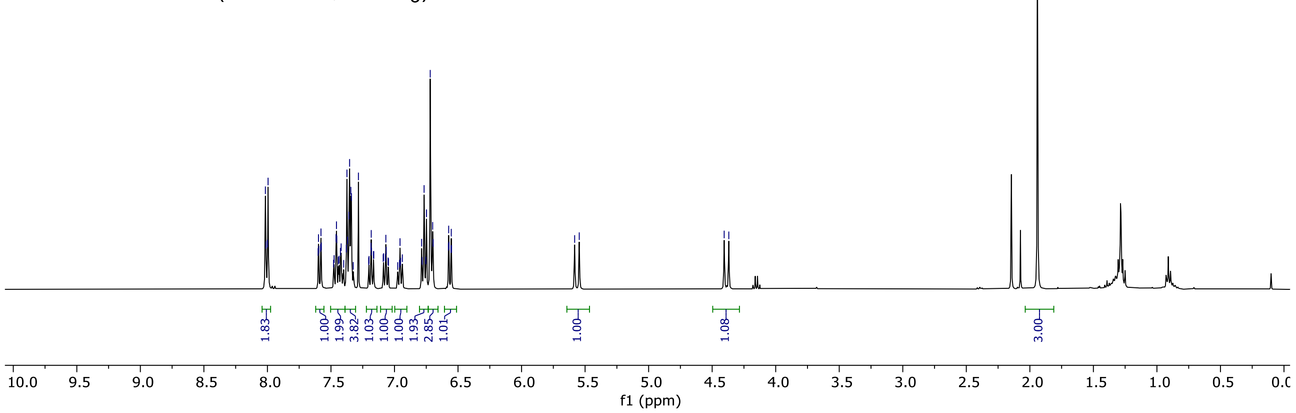

2bb, ^{13}C NMR (101 MHz, $CDCl_3$):

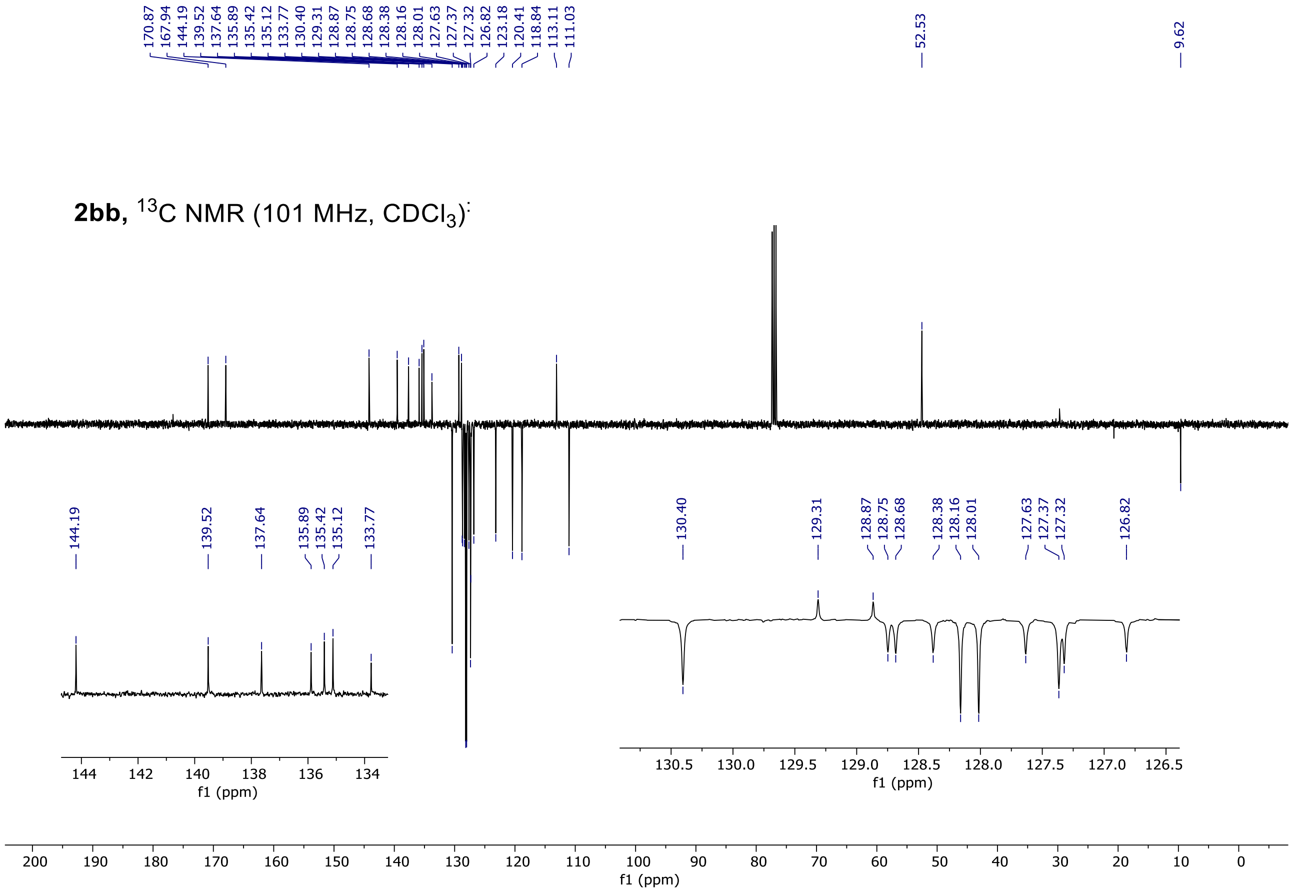

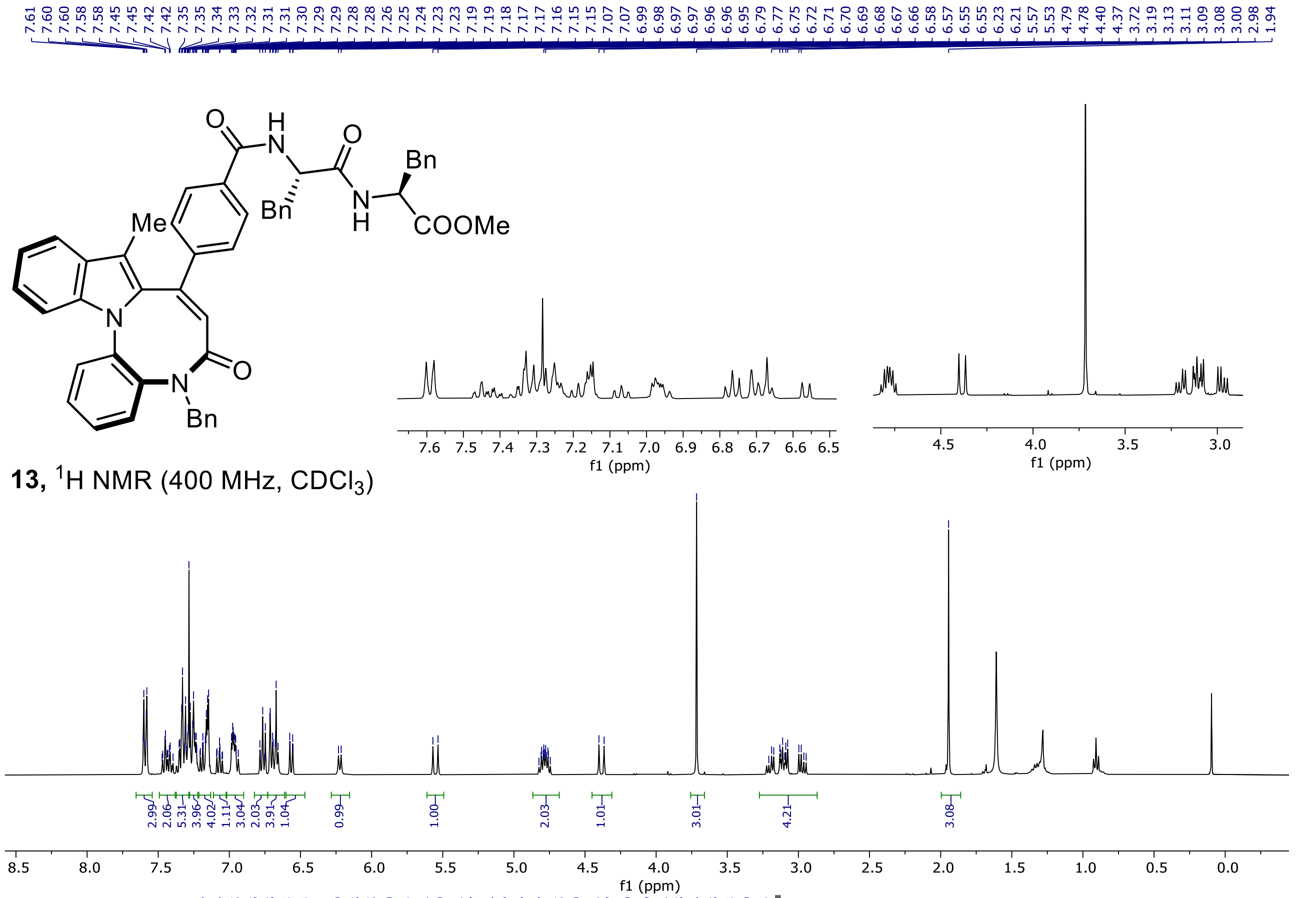

13, ¹H NMR (400 MHz, $CDCl_3$)

13, ¹³C NMR (101 MHz, $CDCl_3$)

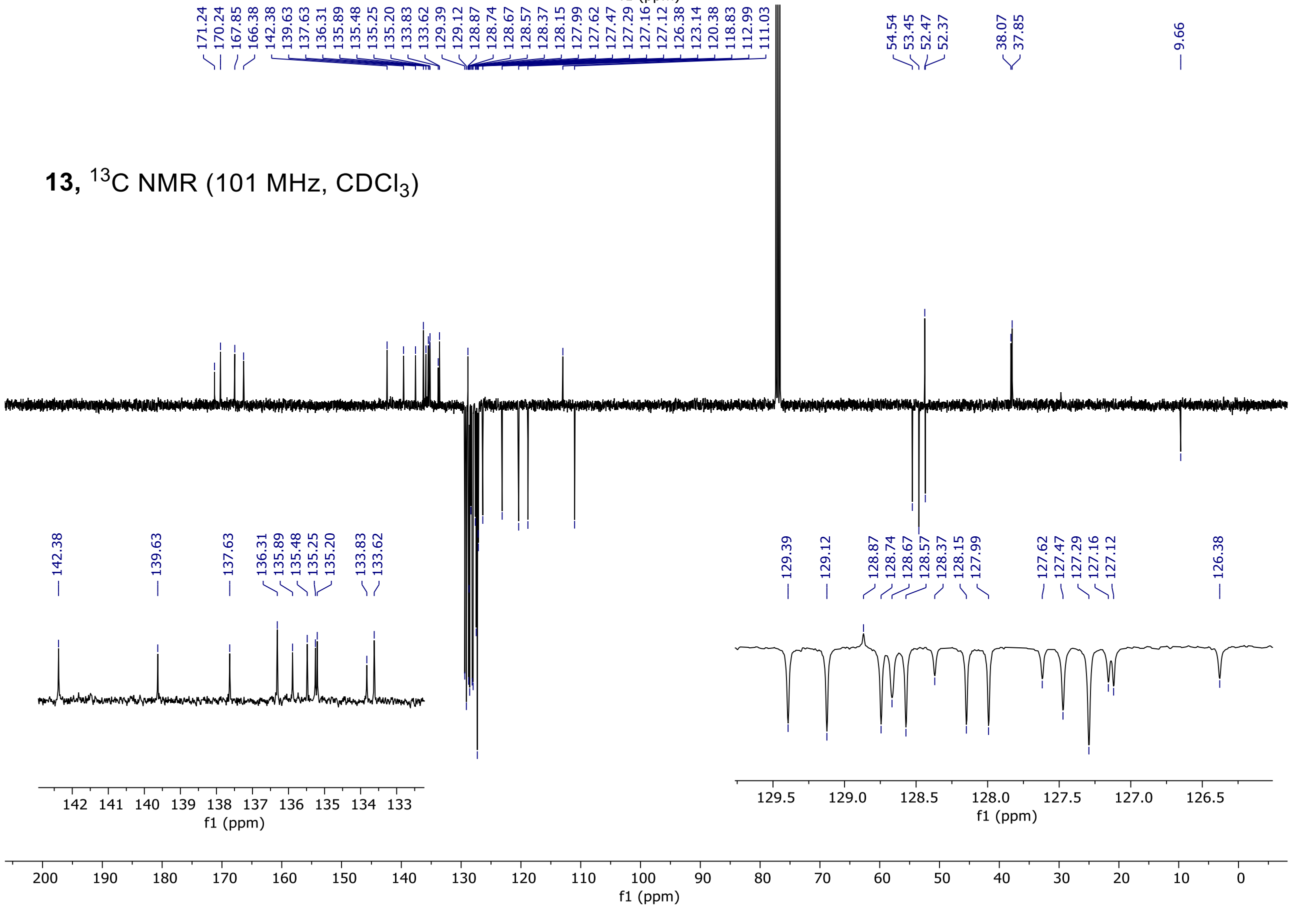

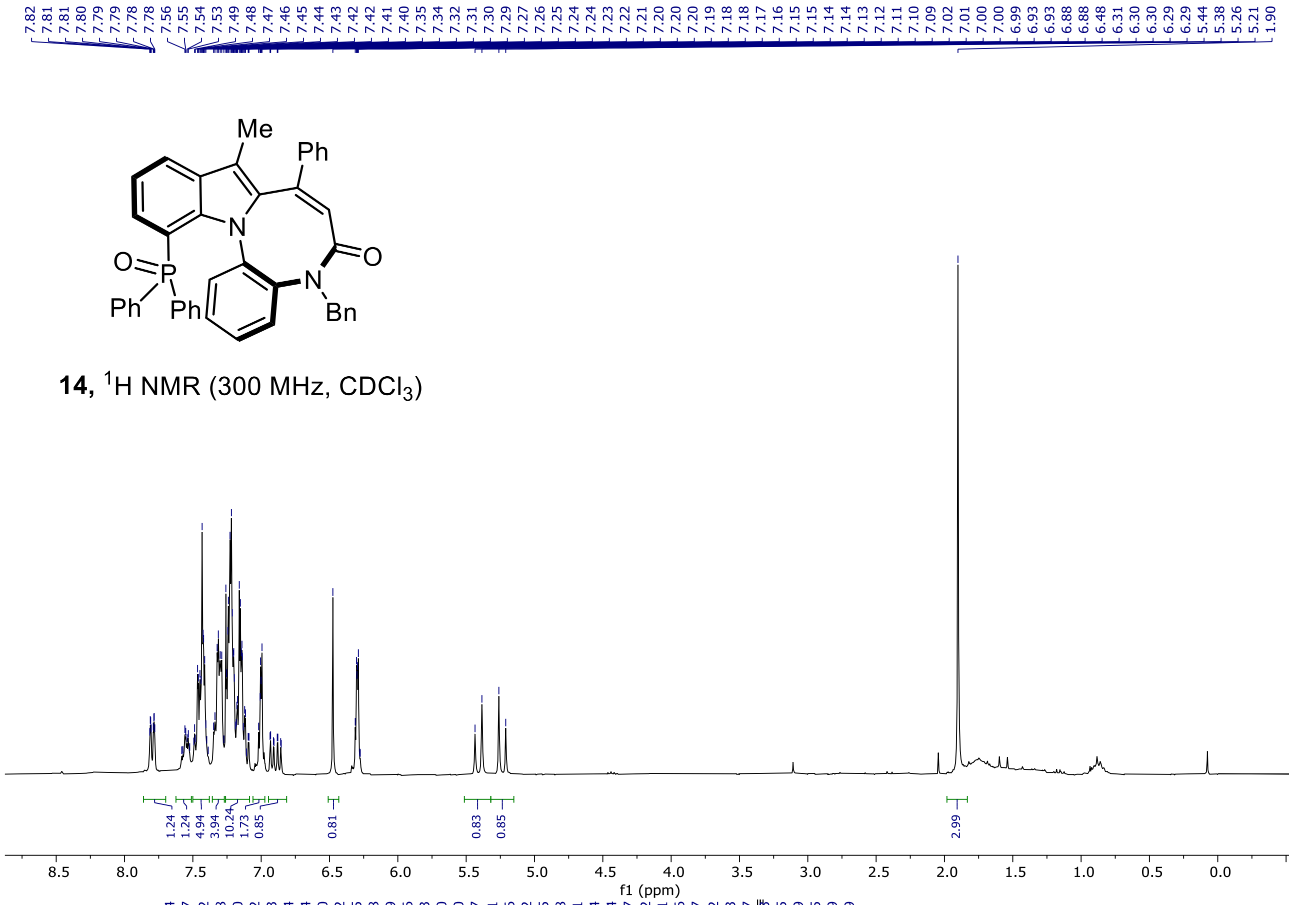

14, ^{1}H NMR (300 MHz, $CDCl_3$)

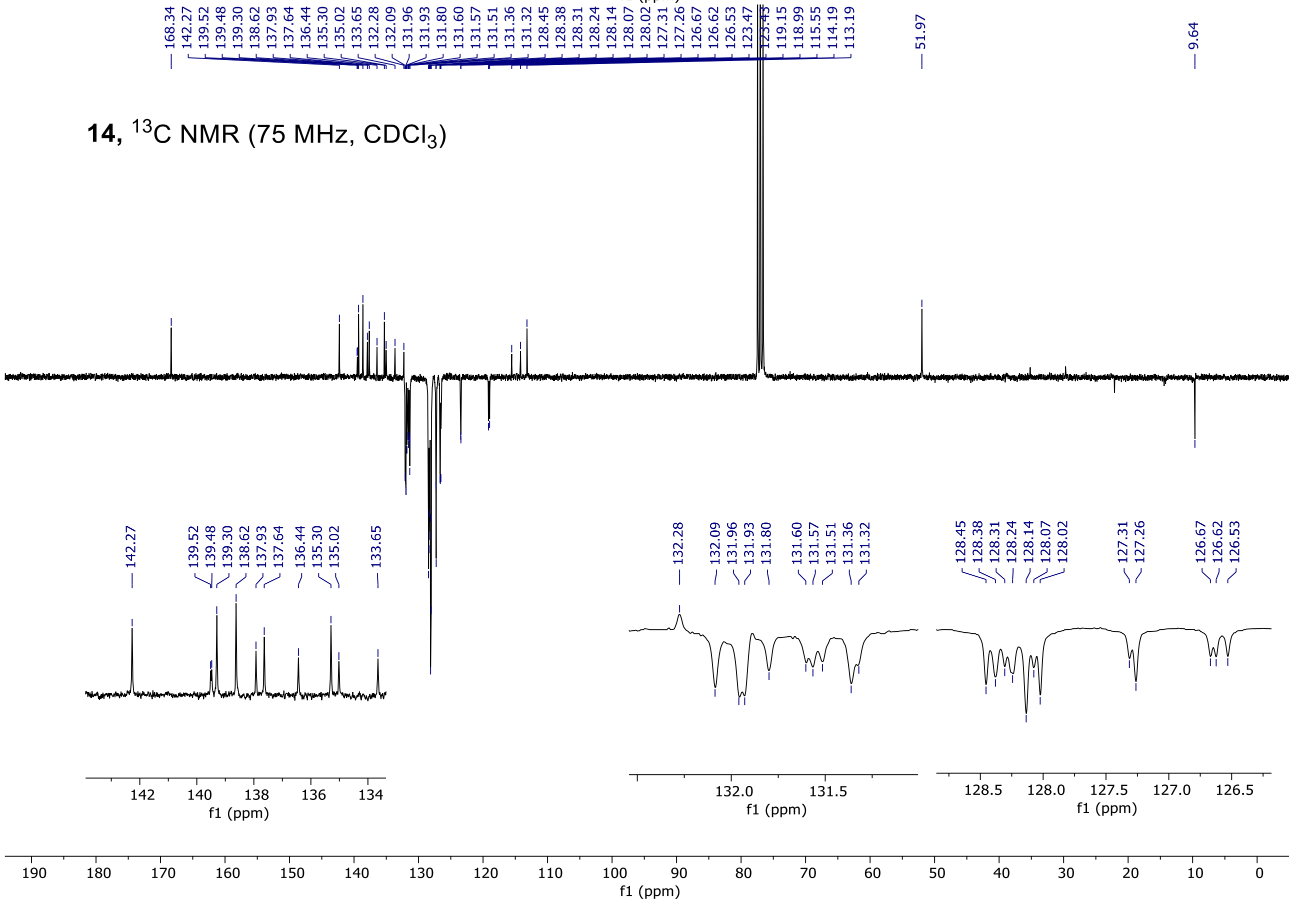

14, ^{13}C NMR (75 MHz, $CDCl_3$)

14, ^{31}P NMR (162 MHz, $CDCl_3$)

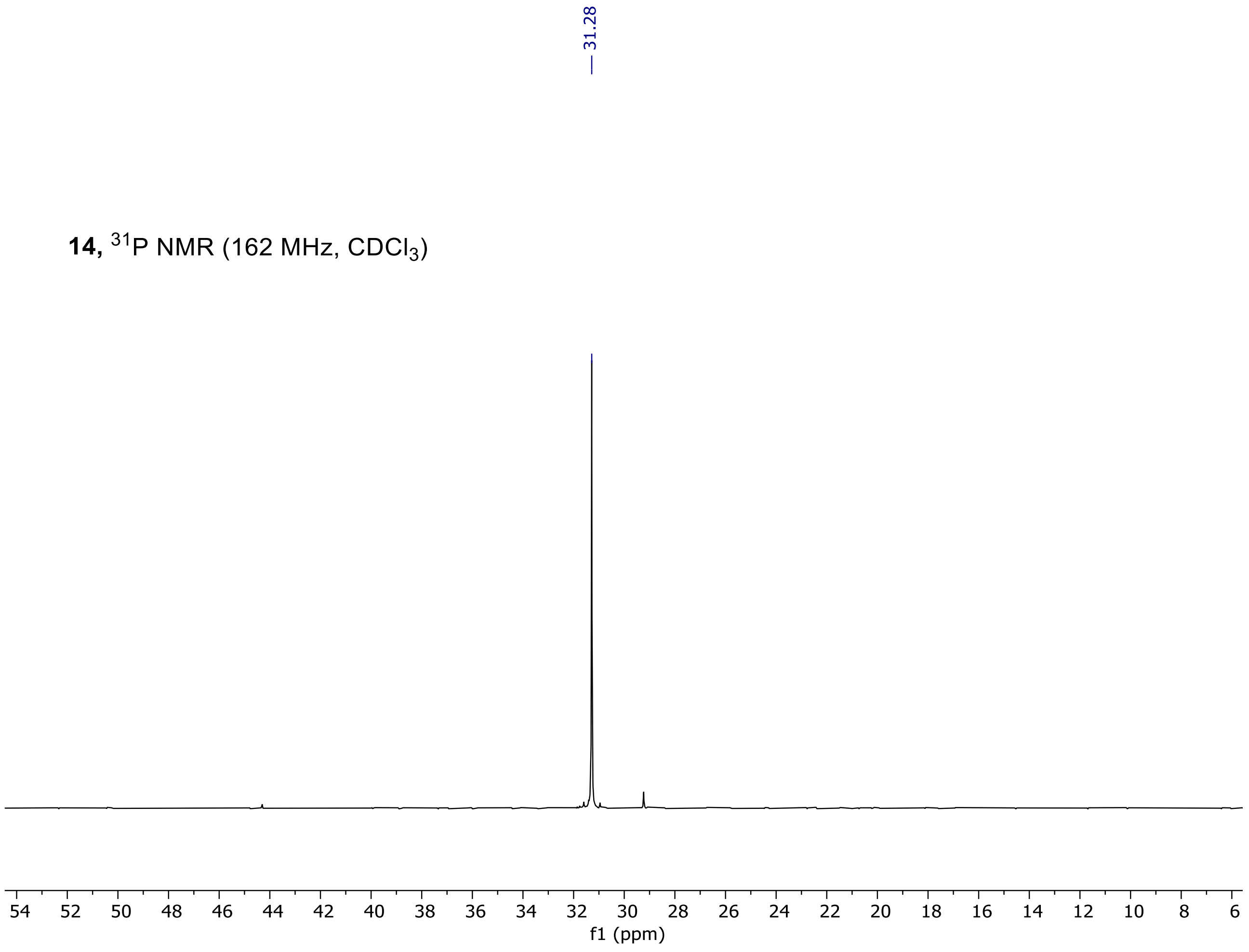

Supplement: SC-017-D6SC00020G-s001 [file SC-017-D6SC00020G-s001.pdf]
